# Combinatorial Synthesis of Structurally Diverse Triazole-Bridged Flavonoid Dimers and Trimers

**DOI:** 10.3390/molecules21091230

**Published:** 2016-09-16

**Authors:** Tze Han Sum, Tze Jing Sum, Warren R. J. D. Galloway, Súil Collins, David G. Twigg, Florian Hollfelder, David R. Spring

**Affiliations:** 1 Department of Chemistry, University of Cambridge, Lensfield Road, Cambridge CB2 1EW, UK; ths29@cam.ac.uk (T.H.S.); tjs76@cam.ac.uk (T.J.S.); wrjdg2@cam.ac.uk (W.R.J.D.G.); sc806@cam.ac.uk (S.C.); dgt24@cam.ac.uk (D.G.T.); 2 Department of Biochemistry, University of Cambridge, Tennis Court Road, Cambridge CB2 1GA, UK; fh111@cam.ac.uk

**Keywords:** flavonoid, triazole, dimer, trimer, hybridization, structural diversity

## Abstract

Flavonoids are a large family of compounds associated with a broad range of biologically useful properties. In recent years, synthetic compounds that contain two flavonoid units linked together have attracted attention in drug discovery and development projects. Numerous flavonoid dimer systems, incorporating a range of monomers attached via different linkers, have been reported to exhibit interesting bioactivities. From a medicinal chemistry perspective, the 1,2,3-triazole ring system has been identified as a particularly attractive linker moiety in dimeric derivatives (owing to several favourable attributes including proven biological relevance and metabolic stability) and triazole-bridged flavonoid dimers possessing anticancer and antimalarial activities have recently been reported. However, there are relatively few examples of libraries of triazole-bridged flavonoid dimers and the diversity of flavonoid subunits present within these is typically limited. Thus, this compound type arguably remains underexplored within drug discovery. Herein, we report a modular strategy for the synthesis of novel and biologically interesting triazole-bridged flavonoid heterodimers and also very rare heterotrimers from readily available starting materials. Application of this strategy has enabled step-efficient and systematic access to a library of structurally diverse compounds of this sort, with a variety of monomer units belonging to six different structural subclasses of flavonoid successfully incorporated.

## 1. Introduction

**Figure 1:**
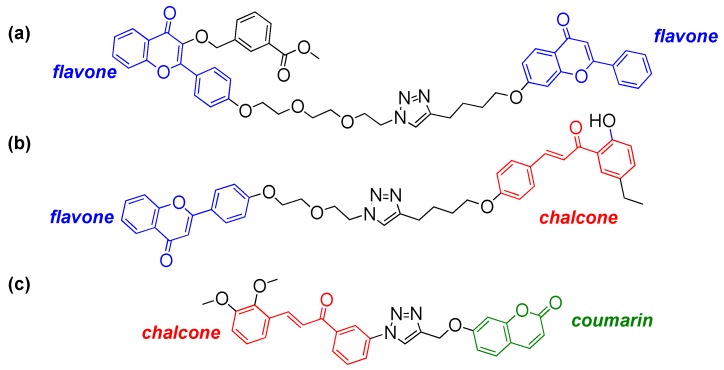
Three examples of biologically active triazole-linked flavonoid dimers. The flavonoid subunits in each example are highlighted in colours. (**a**) A modulator of multidrug resistance in some cancers [10]; (**b**) A modulator of multidrug resistance in some cancers [10]; (**c**) A compound with anticancer activity and antimalarial activity [11].

Flavonoids are a large family of polyphenolic compounds that represent dietary constituents of importance to good health as well as a potentially important new class of pharmaceutical lead substrates [1,2,3]. There are several subclasses of flavonoids, including aurones, chalcones, coumarins, flavones and isoflavones, which serve as the core structural units of numerous biologically active molecules [4,5,6,7]. In recent years, synthetic compounds that contain two such flavonoid units linked together (so-called flavonoid dimers) have garnered attention from the synthetic and medicinal chemistry communities [8,9,10,11,12,13,14,15,16,17]. The generation of species that integrate two pharmacophoric entities (both homo- and hetero-dimers) is a common strategy in drug discovery [18,19] and numerous flavonoid dimer systems, incorporating a range of monomers linked in a variety of ways, have been reported to exhibit biologically useful properties [8,9,10,11,12,13,14,15,16,17]. From a medicinal chemistry perspective, the 1,2,3-triazole ring system has been identified as a particularly attractive linker moiety owing to various favourable properties including ease of synthesis, proven biological relevance and metabolic stability [11,18,20]; indeed, triazole-bridged flavonoid dimers possessing anticancer [10,11] and antimalarial [11] activities have recently been reported (Figure 1). However, there are relatively few examples of libraries of triazole-bridged flavonoid dimers, and the diversity of flavonoid subunits present within these is typically limited. Thus, the triazole-bridged flavonoid dimer compound type arguably remains underexplored within drug discovery. We were interested in investigating the biological potential of triazole-bridged flavonoid dimers further and so sought access to a more structurally diverse collection of such compounds incorporating a wide range of flavonoid units. In addition, we were also interested in accessing triazole-bridged trimeric derivatives, which are also expected to have interesting biological properties. Though the synthesis of triazole-bridged flavonoid trimers has previously been reported [10], compounds of this sort are very rare and they have received relatively little attention from synthetic and medicinal chemists. Herein, we report the development of a modular strategy for the synthesis of novel triazole-bridged flavonoid heterodimers and heterotrimers. Application of this strategy has enabled concise and systematic access to a library of 46 structurally diverse compounds of this sort (41 dimers and five trimers) from readily available starting materials.

## 2. Results and Discussion

### 2.1. Outline of the Synthetic Strategy

**Scheme 1:**
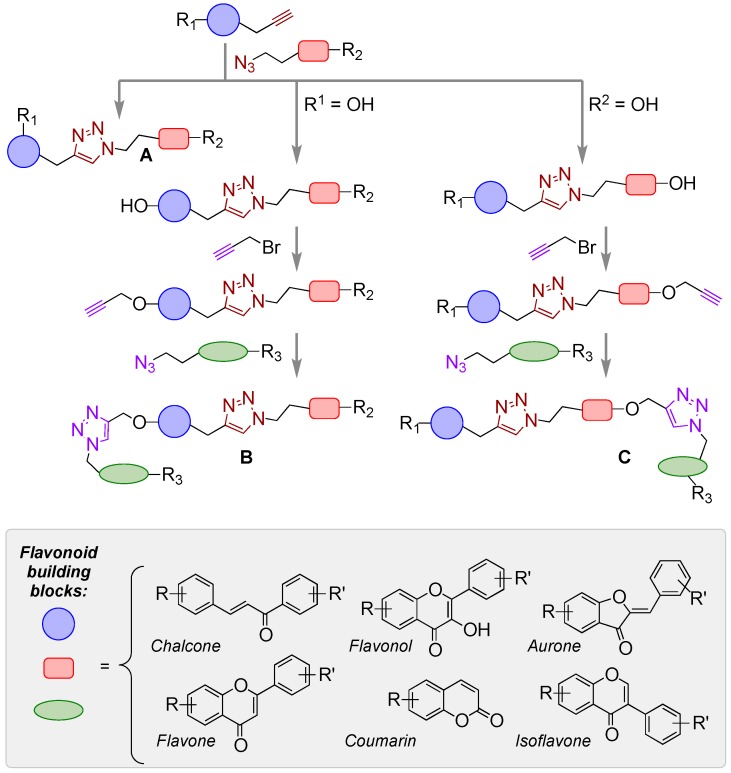
Overview of the branching-type strategy towards structurally diverse triazole-bridged flavonoid heterodimers and heterotrimers. It was anticipated that homo- dimers and trimers could also be accessed through the use of appropriate building blocks.

Inspired by previous studies on the synthesis of triazole-linked flavonoid libraries [10,11] we envisaged a branching-type strategy to access triazole-bridged flavonoid dimers and trimers, based around the use of iterative copper-catalysed “click”-type alkyne-azide 1,3-dipolar cycloadditions (Scheme 1) [21]. It was anticipated that flavonoid monomer units bearing a terminal alkyne group (“alkyne-flavonoid” building blocks) could be reacted with a range of flavonoid monomer units bearing a terminal azide group (“azido-flavonoid” building blocks) to furnish diverse and novel triazole-bridged flavonoid homo- and hetero-dimers (of the general structure **A**, Scheme 1). The presence of a free hydroxyl functionality in either monomer unit would allow for post-cyclisation introduction of a terminal alkyne group in the dimers, thus providing the necessary synthetic handle for a further cycloaddition with varied alkyne-flavonoids to furnish structurally diverse triflavonoid derivatives of the general forms **B** and **C** (Scheme 1). The presence of additional synthetic handles in any given monomer unit should also allow for further elaboration of the dimers and trimers. Overall, it was anticipated that this modular strategy would enable step-efficient and facile access to a structurally diverse library of triazole-bridged flavonoid dimers and trimers through the use of a variety of different flavonoid building blocks belonging to different flavonoid structural subclasses.

### 2.2. Building Block Design and Synthesis

In order to facilitate the generation of structural diversity in the final compound collection, building blocks belonging to a variety of flavonoid structural subclasses (chalcone, coumarin, flavone, aurone, flavonol and isoflavone, see Scheme 1) and derivatives thereof were targeted. Structural diversity, including functional group diversity, within building blocks of some subclasses was also sought in order to further increase the overall structural diversity of the final library (as well as providing a means of introducing additional biomolecular-interacting elements into the library compounds, for example, additional bio-relevant heterocyclic motifs and hydrogen-bonding functionalities). Variation in the position of the key alkyne/azide ligation handles around the flavonoid structures was also envisaged as a strategy to further increase library structural diversity, since this would enable access to different structural isomers of any given dimers/trimers. On the basis of synthetic tractability, various alkyne-chalcones, flavones and isoflavones and azido-chalcone, flavonols and flavones were targeted. Hydroxyl-substituted building blocks were also required in order to allow access to trimeric species (as outlined in Scheme 1). Based on predicted synthetic accessibility, the syntheses of a hydroxyl-substituted alkyne-chalcone, alkyne-flavonol, azido-chalcone and azido-flavonol were targeted. 

#### 2.2.1. Synthesis of the Alkyne-Flavonoid Building-Blocks

##### Alkyne-Chalcones

**Scheme 2:**
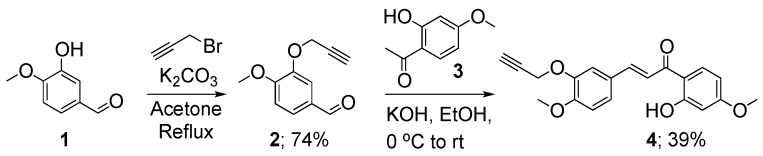
Synthesis of hydroxyl-substituted alkyne-chalcone building block **4**.

Hydroxyl-substituted alkyne-chalcone **4** was accessed from phenol **1** via a two-step sequence: alkylation with propargyl bromide proceeded smoothly to yield aldehyde **2** and subsequent Claisen-Schmidt aldol reaction with acetophenone **3** yielded the target compound **4** (Scheme 2) [22].

**Scheme 3:**
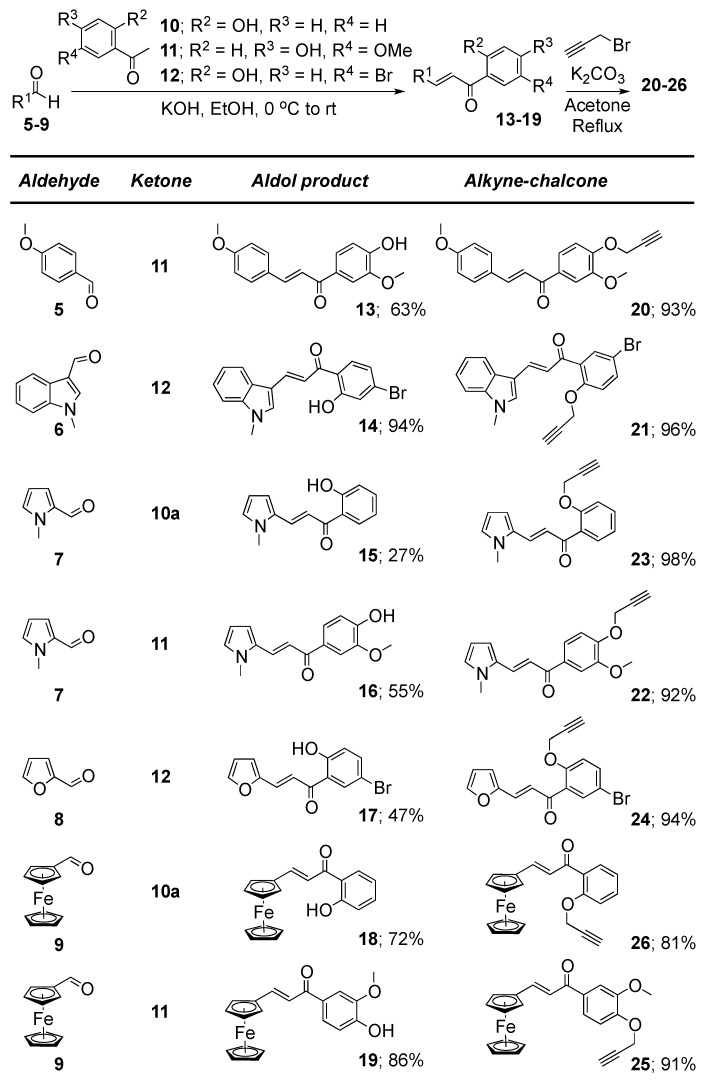
Synthesis of alkyne-chalcone building blocks **20**–**26**.

Structurally diverse alkyne-chalcone building blocks **20**–**26**, including chalconoid derivatives incorporating a range of heteroaromatic scaffolds and an unusual ferrocenyl motif, were generated from aldehydes **5**–**9** respectively by Claisen-Schmidt aldol condensation with various acetophenone derivatives followed by propargylation (Scheme 3).

##### Alkyne-Flavones

**Scheme 4:**
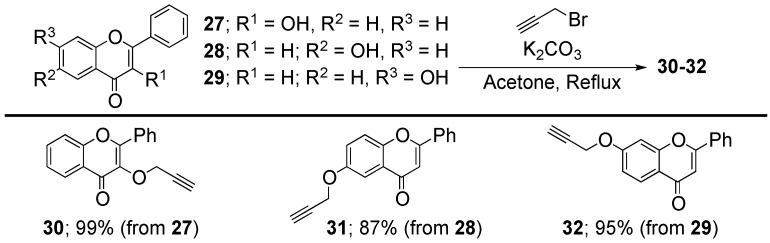
Synthesis of alkyne-flavone building blocks **30**–**32**.

Propargylation of commercially available flavones **27**–**29** proceeded smoothly to furnish alkyne-flavones **30**–**32** with the alkyne synthetic handle appended at various positions on the flavone core unit (Scheme 4) [23,24].

##### Alkyne-Flavonol

**Scheme 5:**
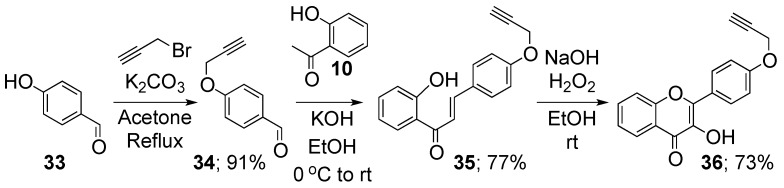
Synthesis of alkyne-flavonol building block **36**.

The preparation of alkyne-flavonol **36** commenced with the synthesis of chalcone **35** via the Claisen-Schmidt reaction of the alkyne-substituted benzaldehyde **34** with acetophenone **10**. Subsequent Algar-Flynn-Oyamada (AFO) oxidation [22] of the chalcone **35** proceeded smoothly to furnish **36** (Scheme 5).

##### Alkyne-Isoflavones

**Scheme 6:**
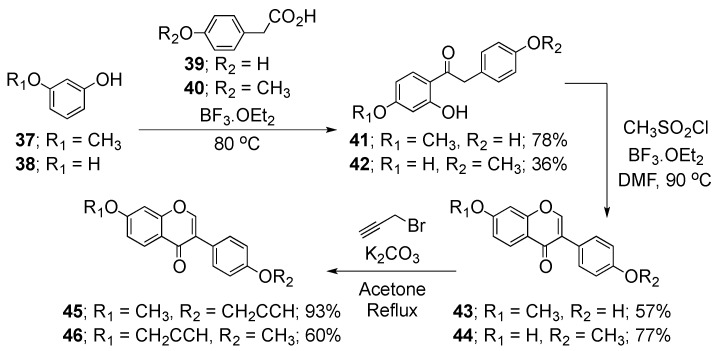
Synthesis of alkyne-isoflavones **45** and **46**.

The preparation of the alkyne-isoflavone building blocks **45** and **46** commenced with the acylation of commercially available substituted phenols **37** and **38** with phenylacetic acids **39** and **40** to afford the deoxybenzoins **41** and **42** [25]. Subsequent cyclization of **41** and **42** in methanesulfonyl chloride afforded the corresponding isoflavones **43** and **44** which then underwent propargylation to yield the desired alkyne-isoflavones **45** and **46** (Scheme 6) [25]. 

##### Alkyne-Coumarin

**Scheme 7:**
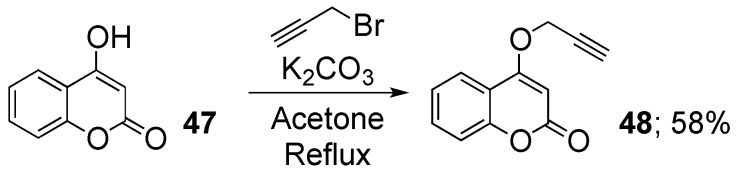
Synthesis of alkyne-coumarin **48**.

Alkyne-coumarin **48** was synthesised by propargylation of hydroxycoumarin **47** in the presence of anhydrous potassium carbonate (Scheme 7) [11].

##### Alkyne-Aurone

**Scheme 8:**
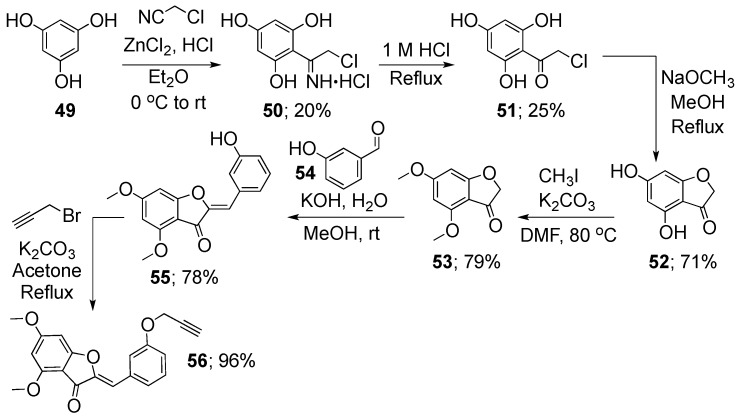
Synthesis of alkyne-aurone **56**.

Alkyne-aurone **56** was prepared from commercially available phloroglucinol **49** (Scheme 8)**.** Condensation with chloroacetonitrile in the presence of ZnCl_2_ furnished imine **50** [26]. Subsequent hydrolysis under acidic conditions afforded ketone **51** which was then treated with methanolic sodium methoxide to give hydroxybenzofuranone **52** [26]. Methyl protection of the free hydroxyl groups afforded benzofuranone **53** which was then condensed with 3-hydroxybenzaldehyde **54** under basic conditions to yield hydroxyaurone **55**. Subsequent propargylation gave the desired alkyne-aurone **56** in an excellent yield (Scheme 8).

#### 2.2.2. Synthesis of the Azido-Flavonoid Building Blocks

##### Azido-Coumarin

**Scheme 9:**
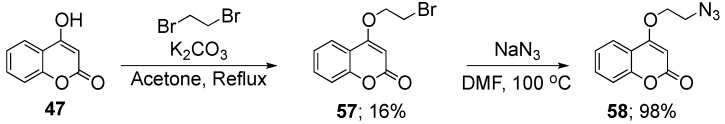
Synthesis of azido-coumarin **58**.

Azido-coumarin **58** was prepared from readily available hydroxycoumarin **47** by alkylation (to form **57**) followed by reaction with sodium azide (Scheme 9) [27].

##### Azido-Chalcones

**Scheme 10:**
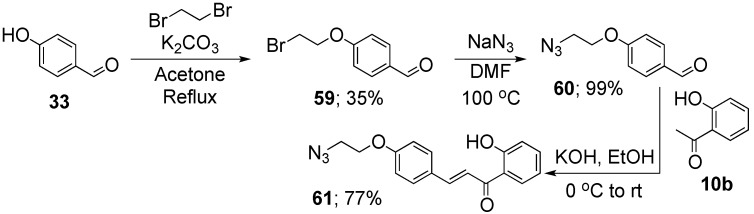
Synthesis of azido-chalcone **61**.

**Scheme 11:**
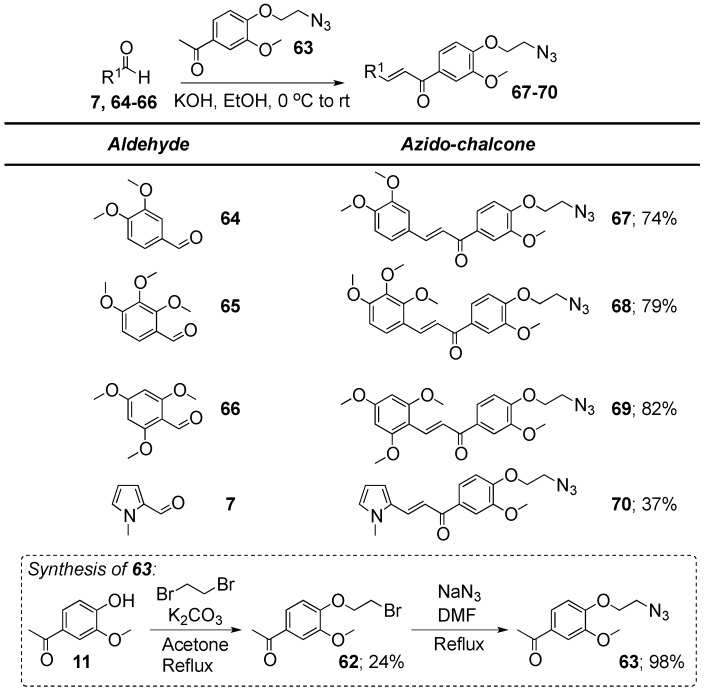
Synthesis of azido-chalcones **67**–**70**.

Hydroxyl-substituted azido-chalcone **61** was prepared by a three step sequence from phenolic aldehyde **33** (Scheme 10). Reaction with 1,2-dibromoethane generated aldehyde **59** and subsequent nucleophilic substitution with sodium azide produced azide **60** in an excellent yield. Claisen-Schmidt aldol condensation with ketone **10b** then yielded the target compound **61** [22]. Alternatively, Claisen-Schmidt aldol condensation of aldehydes **64**–**66** and **7** with readily-prepared azido-ketone **63** furnished azido-chalcone building blocks **67**–**70** respectively (Scheme 11) [22].

##### Azido-Flavonols

**Scheme 12:**
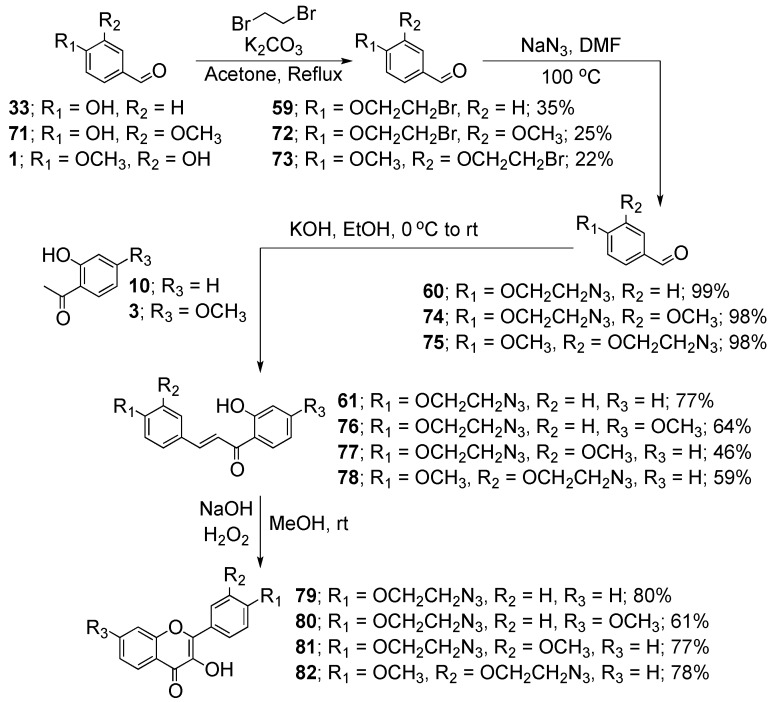
Synthesis of azido-flavonols **79**–**82**.

Aldehydes **59** and **72** and **73**, generated by alkylation of **33**, **71** and **1** respectively with 1,2-dibromoethane, were reacted with sodium azide to form **60** and **74** and **75** respectively (Scheme 12). Subsequent aldol condensation with acetophenones **10** or **3** (see Scheme 12) furnished chalcones **61** and **76**–**78** and AFO proceeded smoothly in all cases to furnish azido-flavonols **79** and **80**–**82** in good yields [22].

##### Azido-Flavones

**Scheme 13:**
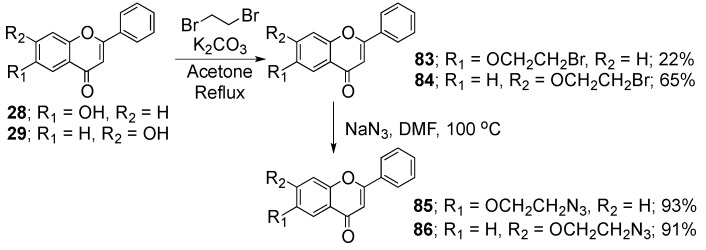
Synthesis of azido-flavones **85**–**86**.

**Scheme 14:**
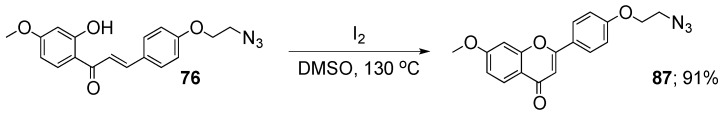
Synthesis of azido-flavone **87**.

Azido-flavones **85** and **86** were readily accessed from commercially available hydroxyflavones **28** and **29** by reaction with 1,2-dibromoethane to forge **83** and **84** followed by nucleophilic substitution with sodium azide (Scheme 13) [10,28]. Azido-flavone **87** was prepared in an excellent yield from chalcone **76** by an iodine-mediated oxidative cyclization (Scheme 14).

##### Azido-Aurones

**Scheme 15:**
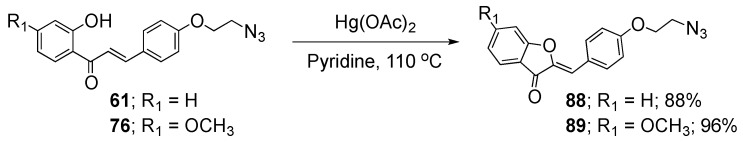
Synthesis of azido-aurones **88**–**89**.

Mercury(II) acetate-mediated oxidative cyclization of chalcones **61** and **76** furnished azido-aurones **88** and **89** respectively in excellent yields (Scheme 15) [29].

#### 2.2.3. Synthesis of Triazole-Bridged Flavonoid Dimers

**Scheme 16:**
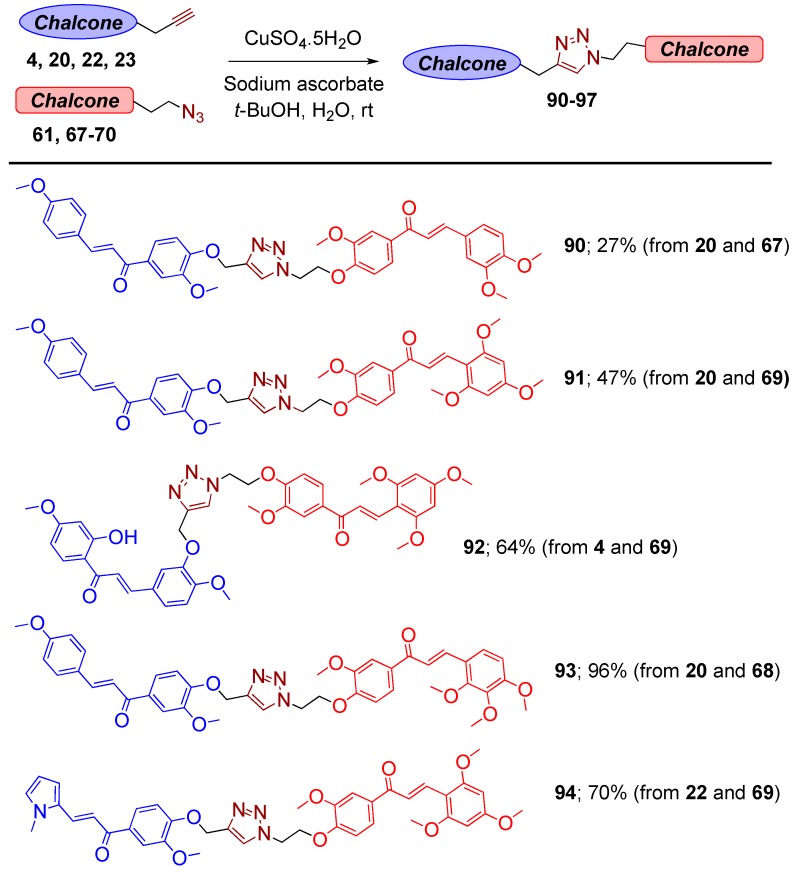

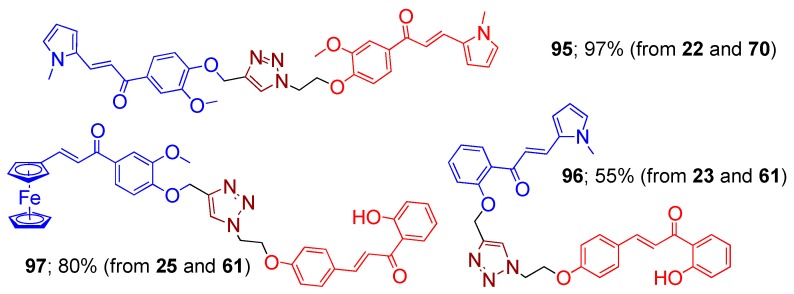
Synthesis of triazole-bridged chalcone-chalcone dimers.

**Scheme 17:**
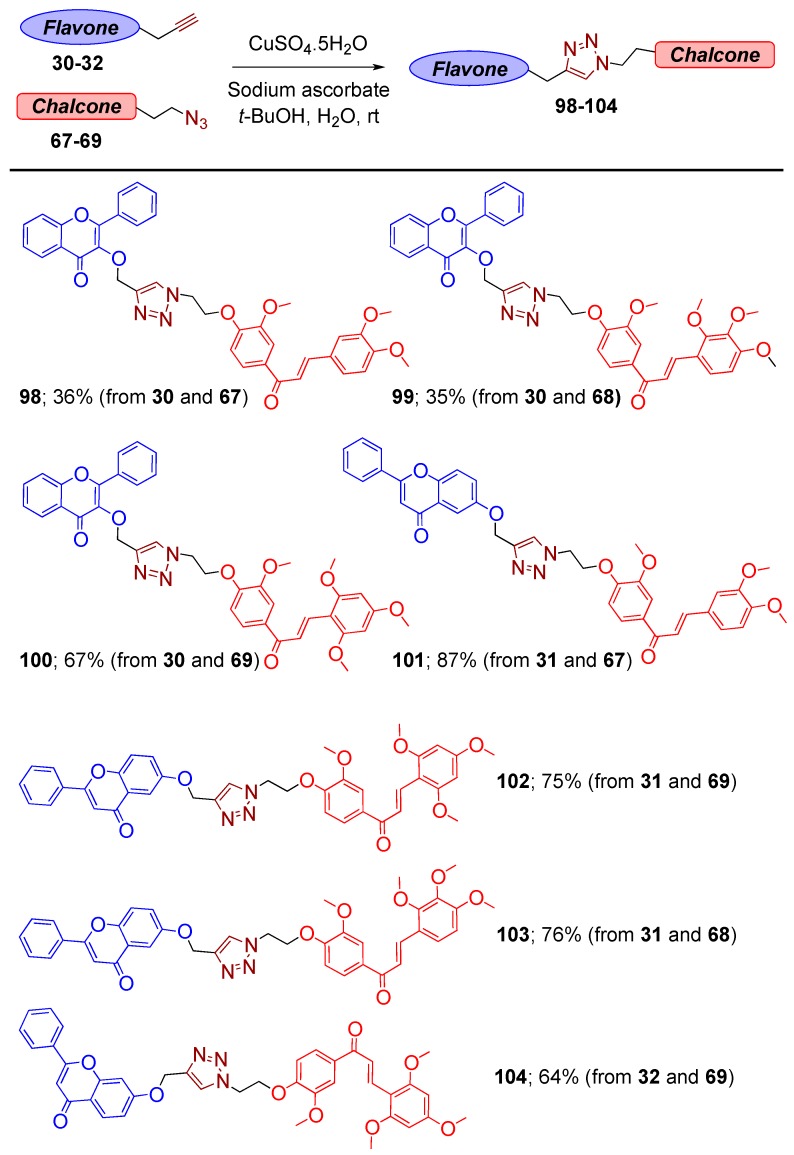
Synthesis of triazole-bridged flavone-chalcone dimers.

**Scheme 18:**
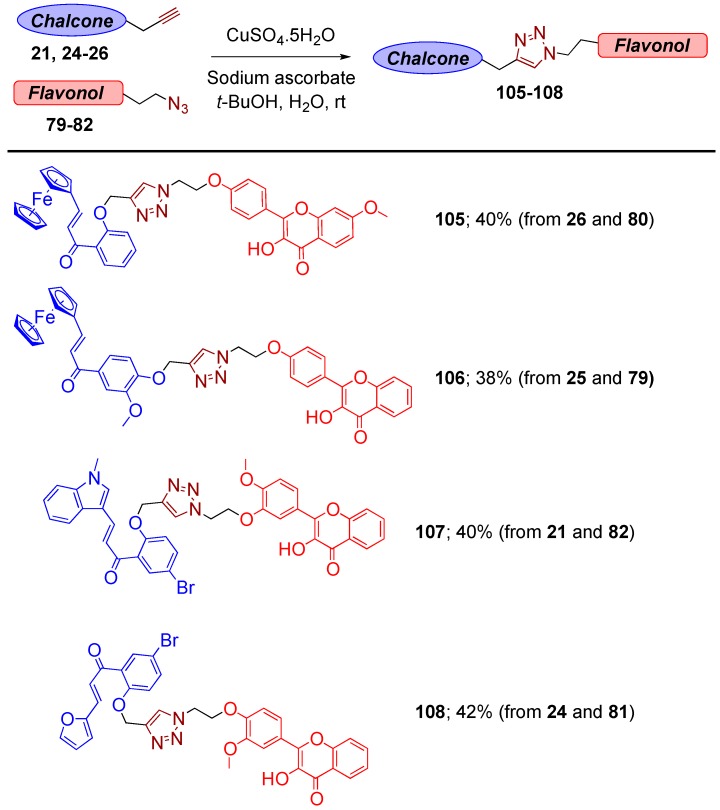
Synthesis of triazole-bridged chalcone-flavonol dimers.

**Scheme 19:**
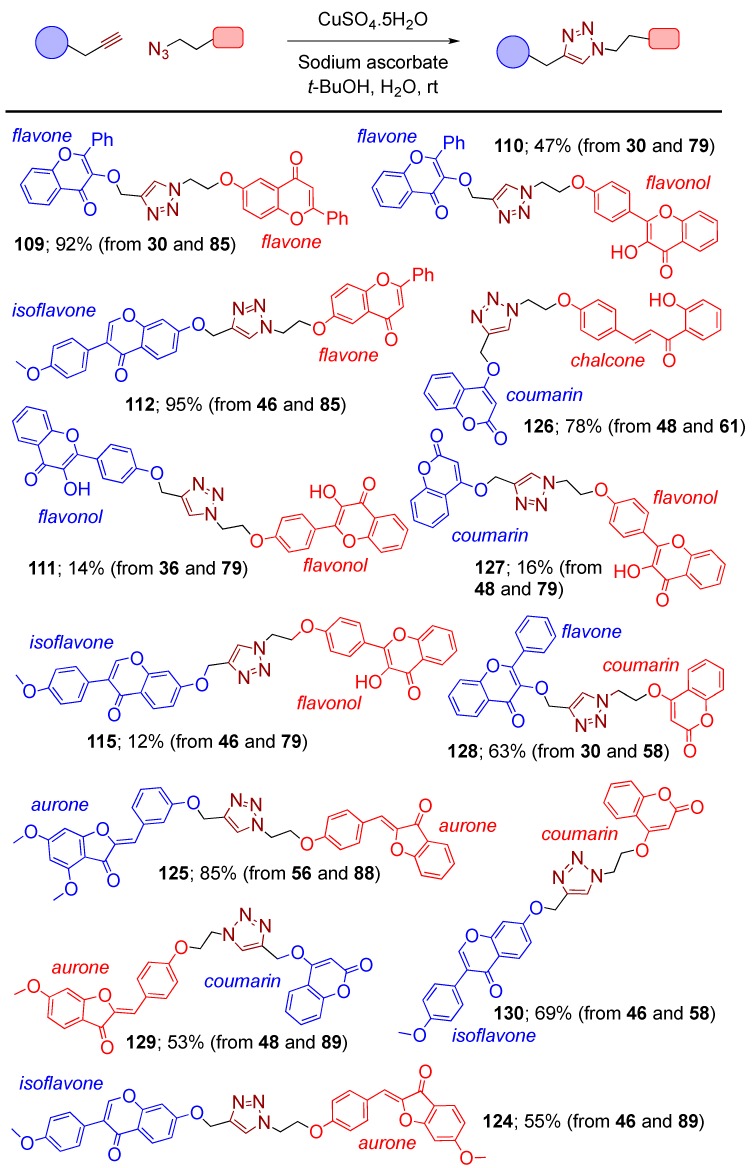
Synthesis of some triazole-bridged dimers.

**Scheme 20:**
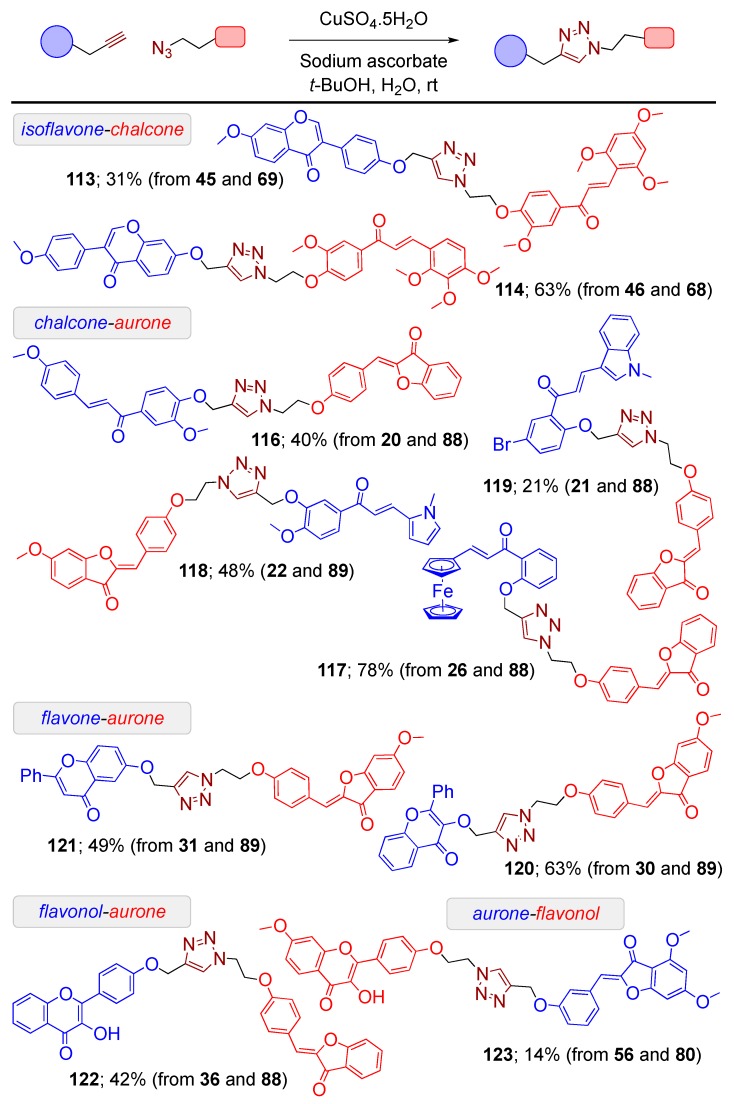
Synthesis of some triazole-bridged dimers.

With the alkyne- and azido-flavonoid building blocks in hand, we were ready to forge a series of dimeric combinations via triazole formation. Thus, various pairs of building blocks were subjected to standard copper-mediated “click” cycloaddition conditions to generate 41 distinct and diverse triazole-bridged flavonoid dimers (compounds **90**–**130**, Scheme 16, Scheme 17, Scheme 18, Scheme 19 and Scheme 20). The reactions generally proceeded smoothly and with high levels of regioselectivity and isolated yields of the target compounds were typically moderate-to-good. Six different biologically-relevant flavonoid structural subclasses (chalcone, flavonol, aurone, flavone, coumarin and isoflavone) were successfully incorporated into the dimer library together with other biologically-relevant features, and variation within building blocks belonging to certain subclasses allowed for the generation of additional structural diversity in the library and the concomitant introduction of additional biomolecule-interacting elements (for example, the varied heterocyclic motifs exhibited by the chalcone-chalcone dimers **90**–**97**). Several compounds also featured groups that could provide synthetic handles for further elaboration or diversification (for example, compounds **96** and **97** and **105** and **106** contain a hydroxyl group and the aryl-bromide group present in **107** and **108** could conceivably be exploited in various metal-catalysed cross-coupling processes).

#### 2.2.4. Synthesis of Triazole-Bridged Flavonoid Trimers

**Scheme 21:**
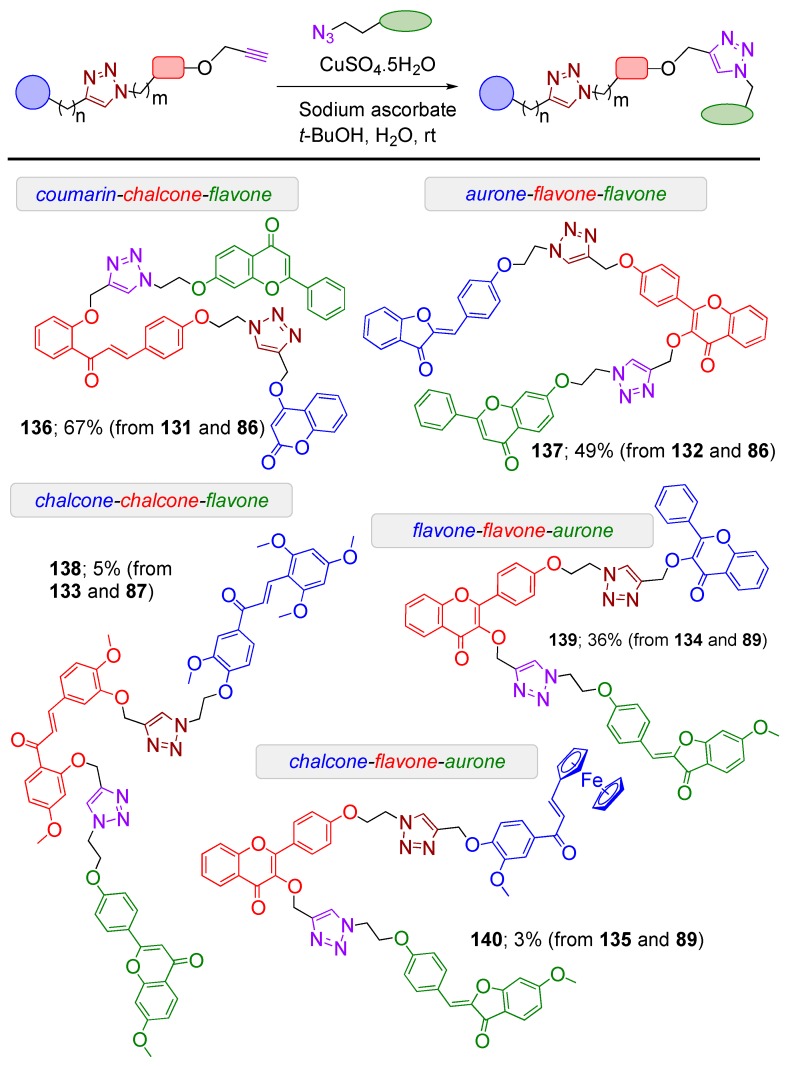
Synthesis of triazole-bridged trimers.

Propargylation of the free phenolic hydroxyl groups of triazole-bridged flavonoid dimers **126**, **122**, **92** and **110** and **106** led to the formation of alkyne-capped derivatives **131**–**135** respectively. These were successfully coupled with three azido-flavonoid building blocks (**86** for **131** and **132**; **87** for **133**; and **89** for **134** and **135**) via copper-catalysed triazole formation to furnish five structurally diverse triazole-bridged flavonoid trimers **136**–**140** (Scheme 21).

### 2.3. Preliminary Biological Screening

**Figure 2:**
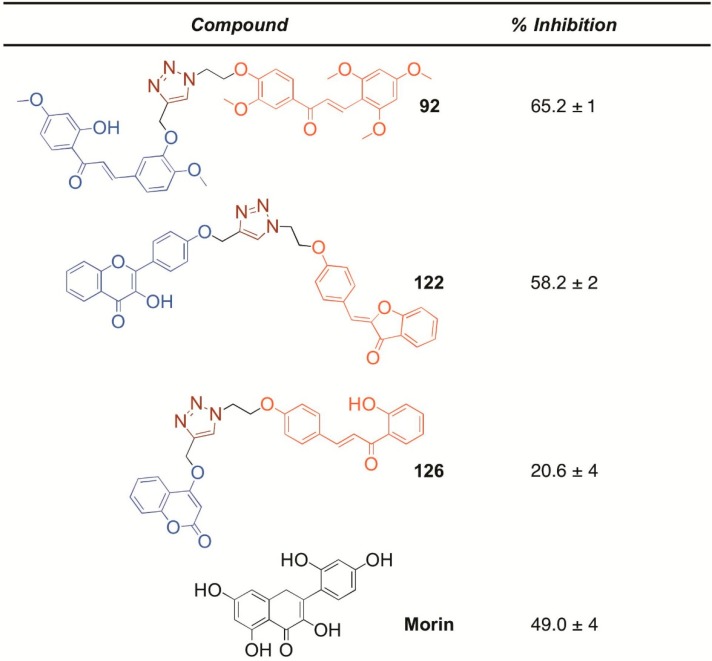
Percentage inhibition of Aβ_42_ aggregation achieved by compounds **92**, **122** and **126** (50 μM concentration) relative to that of Aβ_42_ alone (10 μM), where 100% represents complete aggregation inhibition and 0% shows no inhibition. The data represents the averages and standard error from the results of three independent biological repeats. Inhibitory effect of morin determined under identical assay conditions.

A representative sample of 13 final triazole-bridged dimers (**90**–**93**, **112**–**114**, **122**, **123**, **125**, **126**, **129** and **130**) was screened for inhibitory activity against the aggregation of amyloid beta (**1**–**42**) (Aβ_42_), a pathological hallmark of Alzheimer’s disease [30]. Aggregation of the monomeric form of the peptide into oligomeric and fibrillar species is associated with disease onset and progression. As such, the identification of compounds capable of inhibiting the aggregation process holds great potential for the development of therapeutic agents [31]. Flavonoid and chalcone derivatives have previously shown activity in perturbing the aggregation of Aβ, with compounds such as EGCG myricetin and morin displaying inhibitory activity in a variety of biophysical and in vivo tests [32,33,34,35]. It has also been shown that dimeric flavonoids can display enhanced inhibitory activity than their monomeric counterparts [36], suggesting that the libraries synthesised may be effective at targeting this peptide aggregation pathway. The ability of the triazole-linked dimers to inhibit the Aβ_42_ aggregation was assessed using a thioflavin T (THT) assay (Figure 2). Three of the compounds screened were found to have moderate inhibitory activity, with **92** found to be the most potent and comparable to the inhibitor morin.

It is difficult to draw any firm conclusions at this time regarding structure-activity relationships in the triazole-bridged dimer compound class due to the relatively small sample size and some issues with the solubility and fluorescence behaviour of some compounds under the assay conditions. Nevertheless, this preliminary screen has identified structurally novel Aβ_42_ aggregation inhibitors which could represent interesting scaffolds for further study in this regard.

## 3. Materials and Methods

### 3.1. Chemical Synthesis

#### 3.1.1. General Information

All non-aqueous reactions were performed under a constant stream of dry nitrogen using oven-dried glassware. Standard practices were employed when handling moisture and air-sensitive materials. All reagents and solvents were purchased from commercial sources and used without further purification unless otherwise stated. Room temperature refers to ambient temperature. Temperatures of 0 °C were maintained using an ice-water bath. Petroleum ether was distilled before use. Ethyl acetate and methanol were distilled from calcium hydride. Melting points were measured using a Büchi B545 melting point apparatus and are uncorrected. Thin layer chromatography (TLC) was performed on pre-coated silica gel GF254 plates (Merck, Kenilworth, NJ, USA). Infrared (IR) spectra were recorded on a Spectrum One (FT-IR) spectrophotometer (Perkin-Elmer, Waltham, MA, USA) with internal referencing. Absorption maxima (ν_max_) are reported in wavenumbers (cm^−1^). Flash column chromatography was performed on silica gel (230–400 mesh). ^1^H-NMR and ^13^C-NMR were recorded on an Avance 500 MHz instrument (Bruker, Billerica, MA, USA) in CDCl_3_ or (CD_3_)_2_CO. Chemical shifts (δ) are quoted in ppm, to the nearest 0.01 ppm (^1^H-NMR) or 0.1 ppm (^13^C-NMR) and are referenced to the residual non-deuterated solvent peak. ^1^H-NMR and ^13^C-NMR data for all compounds can be found in Supplementary Materials. LCMS analysis was performed on an ACQUITY H-Class UPLC (Waters, Milford, MA, USA) with an ESCi Multi-Mode Ionisation Waters SQ Detector 2 spectrometer using MassLynx 4.1 software. LC system: solvent A: 2 mM NH_4_OAc in H_2_O/MeCN (95:5); solvent B: MeCN; solvent C: 2% aqueous formic acid; gradient: 5%–95% B with constant 5% C over 1 min at flow rate of 0.6 mL/min. High resolution mass spectrometry (HRMS) measurements were recorded on a Q-TOF mass spectrometer (Micromass, Cary, NC, USA) or a Waters LCT Premier Time of Flight mass spectrometer. Mass values are quoted within the error limits of ±5 ppm mass units. ESI+ refers to the mass ionisation technique.

#### 3.1.2. General Synthetic Procedures

General Procedure A: Synthesis of Biflavonoid Triazole Hybrids (GP-A). To a stirred solution of alkyne flavonoid (1.0 equiv.) and azide flavonoid (1.0 equiv.) in *t*-BuOH/H_2_O (1:1, 40 mL) were added CuSO_4_·5H_2_O (1.1 equiv.) and sodium ascorbate (2.5 equiv.). The reaction mixture was stirred at room temperature for 24 h or until TLC analysis indicated complete consumption of starting material. The resulting mixture was poured into H_2_O (100 mL) and the aqueous solution was extracted with CHCl_3_ (3 × 100 mL). The combined organic layer was washed with H_2_O (2 × 100 mL), brine (2 × 100 mL), dried over anhydrous MgSO_4_, filtered and the solvent removed under reduced pressure. The crude residue was purified by flash column chromatography over silica and recrystallized from MeOH to afford the corresponding biflavonoid triazole hybrids.

General Procedure B: Synthesis of Alkyne Biflavonoid Triazole Hybrids (GP-B). To a stirred solution of the corresponding biflavonoid triazole hybrid (1.0 equiv.) in dry acetone (50 mL) were added propargyl bromide (3.0 equiv.) and anhydrous K_2_CO_3_ (3.0 equiv.). The reaction mixture was heated at reflux with stirring for 24 h under a nitrogen atmosphere or until TLC analysis indicated complete consumption of starting material. The resulting mixture was allowed to cool to room temperature and the solvent removed in vacuo. The crude residue was re-suspended in CHCl_3_ (50 mL) and the organic layer was washed with H_2_O (2 × 100 mL), brine (2 × 100 mL), dried over anhydrous MgSO_4_, filtered and evaporated to dryness. The crude residue was purified by flash column chromatography over silica to afford the corresponding propynyloxy biflavonoid triazole hybrid.

General Procedure C: Synthesis of Propynyloxy flavonoids or benzaldehydes (GP-C). To a stirred solution of the corresponding flavonoid or benzaldehyde (1.0 equiv.) in dry acetone (50 mL) were added anhydrous K_2_CO_3_ (3.0 equiv.) and propargyl bromide (3.0 equiv.). The reaction mixture was heated at reflux with stirring for 24 h under a nitrogen atmosphere or until TLC analysis indicated complete consumption of starting material. The resulting mixture was allowed to cool to room temperature and the solvent removed in vacuo. The crude residue was re-suspended in CHCl_3_ (100 mL) and the organic layer was washed with H_2_O (2 × 100 mL), brine (2 × 100 mL), dried over anhydrous MgSO_4_, filtered and evaporated to dryness. The crude residue was purified by flash column chromatography over silica to afford the corresponding propynyloxy flavonoids or benzaldehydes.

General Procedure D: Synthesis of Chalcones (GP-D). To a stirred solution of KOH (12.0 equiv.) in absolute EtOH (100 mL) cooled to 0 °C in an ice-bath were added dropwise a solution of the corresponding acetophenone (1.0 equiv.) and aldehyde (1.0 equiv.) in EtOH (20 mL). The reaction mixture was stirred at 0 °C for 1 h and then at room temperature for 72 h under a nitrogen atmosphere or until TLC analysis indicated complete consumption of starting material. The resulting mixture was then poured into ice-water (100 mL) and acidified to pH 3–4 with 3 M HCl. The aqueous solution was extracted with CHCl_3_ (3 × 100 mL) and the combined organic layer was washed with satd NaHCO_3_ (2 × 100 mL), brine (2 × 100 mL), dried over anhydrous MgSO_4_, filtered and the solvent removed under reduced pressure. The crude residue was purified by flash column chromatography over silica and/or recrystallized from MeOH or absolute EtOH to afford the corresponding chalcones.

General Procedure E: Synthesis of Indole or Pyrrole 2-hydroxychalcones (GP-E). To a stirred solution of indole or pyrrole aldehyde (1.0 equiv.) and the corresponding 2-hydroxyacetophenone (1.0 equiv.) in absolute EtOH (100 mL) was added piperidine (1.0 equiv.). The reaction mixture was heated at reflux for 24 h under a nitrogen atmosphere or until TLC analysis indicated complete consumption of starting material. The reaction mixture was allowed to cool to room temperature, poured into ice-water (100 mL) and then acidified to pH 3–4 with 3 M HCl. The resulting suspension was filtered and the precipitate washed with ice-water (2 × 100 mL), suction-dried and recrystallized from MeOH to afford the corresponding indole or pyrrole chalcones.

General Procedure F: Synthesis of Flavonols (GP-F). To a stirred solution of the corresponding chalcone (0.30 mmol) in MeOH (20 mL) were added 16% NaOH (aq) (0.60 mL) and 15% H_2_O_2_ (0.30 mL). The reaction mixture was stirred at room temperature for 24 h under a nitrogen atmosphere or until TLC analysis indicated complete consumption of starting material. The resulting mixture was then poured into ice-water (50 mL) and acidified to pH 3–4 with 3 M HCl. The aqueous solution was extracted with CHCl_3_ (3 × 50 mL) and the combined organic layer was washed with satd NaHCO_3_ (2 × 50 mL), brine (2 × 50 mL), dried over anhydrous MgSO_4_, filtered and the solvent removed under reduced pressure. The crude residue was purified by flash column chromatography over silica to afford the corresponding flavonols.

General Procedure G: Synthesis of Phenylethanones (GP-G). To a stirred solution of substituted phenol (1.2 equiv.) in BF_3_·OEt_2_ (50 mL) was added the corresponding phenylacetic acid (1.0 equiv.) and the reaction mixture was heated at 80 °C for 8 h under a nitrogen atmosphere. The resulting dark solution was allowed to cool to room temperature and slowly poured into ice-water (100 mL). The organic layer was separated and the aqueous layer was extracted with EtOAc (3 × 100 mL). The combined organic layer was washed with satd NaHCO_3_ (2 × 100 mL), brine (2 × 100 mL), dried over anhydrous MgSO_4_, filtered and evaporated to dryness. The crude residue was purified by flash column chromatography over silica to afford the corresponding phenylethanones.

General Procedure H: Synthesis of Isoflavones (GP-H). To a stirred solution of the corresponding phenylethanone (1.0 equiv.) in dry DMF (15 mL) was carefully added BF_3_·OEt_2_ (4.0 equiv.) over 10 min under a nitrogen atmosphere. To this mixture, methanesulfonyl chloride (3.0 equiv.) was added at 55 °C, stirred for 1 h and then heated at 80 °C for 24 h. The resulting dark solution was allowed to cool to room temperature and then poured with rapid stirring into ice-water (100 mL). The resulting precipitate was filtered, washed with H_2_O (2 × 100 mL), suction-dried and re-dissolved in EtOAc (100 mL). The organic solution was washed with H_2_O (2 × 100 mL), brine (2 × 100 mL), dried over anhydrous MgSO_4_, filtered and evaporated to dryness. The crude residue was purified by flash column chromatography over silica to afford the corresponding isoflavones.

General Procedure I: Synthesis of Bromoalkylated flavonoids or benzaldehdyes (GP-I). To a stirred solution of the corresponding flavonoid or benzaldehyde (1.0 equiv.) in dry acetone (50 mL) or dry DMF (50 mL) were added anhydrous K_2_CO_3_ (3.0 equiv.) and 1,2-dibromoethane (3.0 equiv.). The reaction mixture was heated at reflux with stirring for 24 h under a nitrogen atmosphere or until TLC analysis indicated complete consumption of starting material. The resulting mixture was allowed to cool to room temperature and the solvent removed in vacuo. The crude residue was re-suspended in CHCl_3_ (100 mL) and the organic layer was washed with H_2_O (2 × 100 mL), brine (2 × 100 mL), dried over anhydrous MgSO_4_, filtered and evaporated to dryness. The crude residue was purified by flash column chromatography over silica to afford the corresponding bromoalkylated flavonoids or benzaldehydes.

General Procedure J: Synthesis of Azido flavonoids or benzaldehydes (GP-J). To a stirred solution of the corresponding bromoalkylated flavonoids or benzaldehydes (1.0 equiv.) in dry DMF (30 mL) was added NaN_3_ (3.0 equiv.). The reaction mixture was heated at 100 °C with stirring for 3 h under a nitrogen atmosphere. The resulting mixture was allowed to cool to room temperature and poured into H_2_O (100 mL). The aqueous solution was extracted with CHCl_3_ (3 × 100 mL) and the combined organic layer was washed with H_2_O (2 × 100 mL), brine (2 × 100 mL), dried over anhydrous MgSO_4_, filtered and evaporated to dryness to afford the corresponding azido flavonoids or benzaldehydes and were used without further purification.

General Procedure K: Synthesis of Aurones (GP-K). To a stirred solution of the corresponding chalcone (1.0 equiv.) in pyridine (10 mL) was added Hg(OAc)_2_ (1.0 equiv.). The reaction mixture was heated at 110 °C with stirring for 1 h under a nitrogen atmosphere. The resulting mixture was then poured into ice-water (50 mL) and acidified to pH 3–4 with 3 M HCl. The aqueous solution was extracted with CHCl_3_ (3 × 50 mL) and the combined organic layer was washed with H_2_O (2 × 50 mL), brine (2 × 50 mL), dried over anhydrous MgSO_4_, filtered and the solvent removed under reduced pressure. The crude residue was purified by flash column chromatography over silica to afford the corresponding aurones.

### 3.2. Synthetic Procedures

#### 3.2.1. Building Block Synthesis

*4-Methoxy-3-(prop-2-yn-1-yloxy)benzaldehyde* (**2**). A mixture of isovanillin (**1**, 10.0 g, 65.7 mmol), propargyl bromide (8.78 mL, 98.6 mmol) and anhydrous K_2_CO_3_ (18.2 g, 131 mmol) in dry acetone (100 mL) was reacted according to GP-C. The crude residue was purified by flash column chromatography (SiO_2_, CH_2_Cl_2_) to afford benzaldehyde **2** (9.23 g, 74%) as a white fluffy solid. m.p. 78–80 °C. TLC *R*_f_ = 0.21 (CH_2_Cl_2_). IR ν_max_ (neat)/cm^−1^: 3229m (C≡C-H str), 3096w (C-H str), 2839w (C-H str), 2124w (C≡C str), 1671s (C=O str), 1599s (C=C str), 1586s (C=C str), 1508s (C=C str), 1436m, 1407m, 1387m, 1261s, 1229s, 1161s, 1129s, 1012s. ^1^H-NMR (500 MHz, CDCl_3_): δ 2.55 (1H, t, *J* = 2.4 Hz, -OCH_2_C≡C*H*), 3.97 (3H, s, -OCH_3_), 4.83 (2H, d, *J* = 2.4 Hz, -OC*H*_2_C≡CH), 7.01 (1H, d, *J* = 8.0 Hz, ArH), 7.53 (1H, dd, *J* = 8.0, 1.6 H z, ArH), 7.55 (1H, d, *J* = 1.6 Hz, ArH), 9.87 (1H, s, CHO). ^13^C-NMR (500 MHz, CDCl_3_): δ 56.2, 56.6, 76.4, 77.7, 110.9, 112.0, 127.3, 129.9, 147.3, 154.9, 190.7. LCMS (ES+) *m*/*z* = 191.2 ([M + H]^+^, *t*_R_ = 3.09 min). These characterisation data are in accordance with that previously reported in the literature [37].

*(E)-1-(2-Hydroxy-4-methoxyphenyl)-3-(4-methoxy-3-(prop-2-yn-1-yloxy)phenyl)prop-2-en-1-one* (**4**). A mixture of benzaldehyde **2** (2.05 g, 10.8 mmol), acetophenone **3** (1.80 g, 10.8 mmol) and KOH (3.02 g, 53.8 mmol) in absolute EtOH (100 mL) was reacted according to GP-D. The crude residue was purified by flash column chromatography (SiO_2_, PE/EtOAc; 5:1) and recrystallized from MeOH to afford chalcone **4** (1.42 g, 39%) as a bright yellow-orange fluffy solid. m.p. 144–146 °C. TLC *R*_f_ = 0.37 (PE/EtOAc; 2:1). IR ν_max_ (neat)/cm^−1^: 3506w (br) (O-H str), 3229m (C≡C-H str), 3081w (C-H str), 2973w (C-H str), 2111w (C≡C str), 1633m (C=O str), 1572m (C=C str), 1556s (C=C str), 1507s (C=C str), 1466m, 1440s, 1361s, 1339s, 1260s, 1206s, 1170m, 1126s, 1018s. ^1^H-NMR (500 MHz, CDCl_3_): δ 2.58 (1H, t, *J* = 2.0 Hz, -OCH_2_C≡C*H*), 3.87 (3H, s, -OCH_3_), 3.94 (3H, s, -OCH_3_), 4.85 (2H, d, *J* = 2.4 Hz, -OC*H*_2_C≡CH), 6.49–6.52 (2H, m, ArH), 6.94 (1H, d, *J* = 8.4 Hz, ArH), 7.32 (1H, dd, *J* = 8.4, 2.0 Hz, ArH), 7.35 (1H, d, *J* = 1.2 Hz, ArH), 7.45 (1H, d, *J* = 15.2 Hz, -C*H*=CHCO-), 7.83 (1H, d, *J* = 8.4 Hz, ArH), 7.85 (1H, d, *J* = 15.6 Hz, -CH=C*H*CO-), 13.53 (1H, s, OH). ^13^C-NMR (500 MHz, CDCl_3_): δ 55.6, 56.0, 56.9, 76.3, 78.1, 101.0, 107.7, 111.7, 113.8, 114.1, 118.3, 124.3, 127.7, 131.1, 144.3, 146.9, 152.2, 166.1, 166.6, 191.7. LCMS (ES+) *m*/*z* = 339.2 ([M + H]^+^, *t*_R_ = 1.70 min). HRMS (ESI+) *m*/*z* = 339.1224 [M + H]^+^ found, C_20_H_19_O_5_^+^ required 339.1227.

*(E)-1-(4-Hydroxy-3-methoxyphenyl)-3-(4-methoxyphenyl)prop-2-en-1-one* (**13**). A mixture of aldehyde **5** (7.40 mL, 60.8 mmol), acetophenone **11** (10.0 g, 60.3 mmol) and KOH (16.9 g, 302 mmol) in absolute EtOH (100 mL) was reacted according to GP-D. The crude residue was purified by flash column chromatography (SiO_2_, PE/EtOAc; 5:1) to afford chalcone **13** (10.7 g, 63%) as a bright yellow-orange powdery solid. m.p. 148–150 °C. TLC *R*_f_ = 0.40 (PE/EtOAc; 1:1). IR ν_max_ (neat)/cm^−1^: 3180m (br) (O-H str), 2991w (C-H str), 2836w (C-H str), 1643m (C=O str), 1602m (C=C str), 1560s (C=C str), 1508s (C=C str), 1461w, 1424s, 1338m, 1296s, 1278s, 1248s, 1166s, 1048m, 1026s. ^1^H-NMR (500 MHz, CDCl_3_): δ 3.86 (3H, s, -OCH_3_), 3.98 (3H, s, -OCH_3_), 6.23 (1H, s, OH), 6.94 (2H, d, *J* = 9.0 Hz, ArH), 7.00 (1H, d, *J* = 8.0 Hz, ArH), 7.45 (1H, d, *J* = 15.5 Hz, -C*H*=CHCO-), 7.61 (2H, d, *J* = 8.5 Hz, ArH), 7.64–7.66 (2H, m, ArH), 7.79 (1H, d, *J* = 16.0 Hz, -CH=C*H*CO-). ^13^C-NMR (500 MHz, CDCl_3_): δ 55.4, 56.1, 110.5, 113.7, 114.3, 119.3, 123.5, 127.8, 130.1, 131.2, 143.8, 146.8, 150.2, 161.5, 188.6. LCMS (ES+) *m*/*z* = 285.2 ([M + H]^+^, *t*_R_ = 1.75 min). These characterisation data are in accordance with that previously reported in the literature [38].

*(E)-1-(5-Bromo-2-hydroxyphenyl)-3-(1-methyl-1H-indol-3-yl)prop-2-en-1-one* (**14**). A mixture of indole aldehyde **6** (1.03 g, 6.47 mmol), acetophenone **12** (1.38 g, 6.42 mmol) and piperidine (0.62 mL, 6.28 mmol) in absolute EtOH (100 mL) was reacted according to GP-E. The crude residue was purified by recrystallization from MeOH to afford chalcone **14** (2.15 g, 94%) as a bright orange fluffy solid. m.p. 250–252 °C. TLC *R*_f_ = 0.45 (PE/EtOAc 2:1). IR ν_max_ (neat)/cm^−1^: 3101w (C-H str), 2885w (C-H str), 1626s (C=O str), 1541m (C=C str), 1513s (C=C str), 1462m, 1387m, 1375m, 1344m, 1295m, 1252s, 1177s, 1126s, 1074s, 1028s, 1014m. ^1^H-NMR (500 MHz, CDCl_3_): δ 3.88 (3H, s, -NCH_3_), 6.93 (1H, d, *J* = 8.8 Hz, ArH), 7.38–7.40 (3H, m, ArH), 7.49–7.56 (2H, m, ArH), 7.55 (1H, d, *J* = 15.6 Hz, -C*H*=CHCO-), 8.00–8.03 (2H, m, ArH), 8.23 (1H, d, *J* = 15.2 Hz, -CH=C*H*CO-), 13.25 (1H, br s, OH). ^13^C-NMR (500 MHz, CDCl_3_): δ 33.5, 110.2, 110.3, 113.1, 113.6, 120.5, 120.8, 121.7, 122.1, 123.6, 126.1, 131.5, 135.6, 138.1, 138.4, 140.4, 162.4, 192.5. LCMS (ES+) *m*/*z* = 358.0 ([M + H]^+^, *t*_R_ = 4.96 min). HRMS (ESI+) *m*/*z* = 356.0279 [M + H]^+^ found, C_18_H_15_O_2_NBr^+^ required 356.0281.

*(E)-1-(2-Hydroxyphenyl)-3-(1-methyl-1H-pyrrol-2-yl)prop-2-en-1-one* (**15**). A mixture of pyrrole aldehyde **7** (4.00 mL, 37.2 mmol), acetophenone **10** (4.50 mL, 37.4 mmol) and piperidine (3.80 mL, 38.5 mmol) in absolute EtOH (100 mL) was reacted according to GP-E. The crude residue was purified by recrystallization from MeOH to afford chalcone **15** (2.28 g, 27%) as a bright yellow-orange powdery solid. m.p. 98–100 °C. TLC *R*_f_ = 0.45 (PE/EtOAc; 3:1). IR ν_max_ (neat)/cm^−1^: 3112w (C-H str), 2937w (C-H str), 1627s (C=O str), 1579w (C=C str), 1549s (C=C str), 1479s, 1440m, 1412m, 1384m, 1356w, 1338m, 1290m, 1260s, 1249s, 1203s, 1182w, 1155s, 1092w, 1061s, 1025s. ^1^H-NMR (500 MHz, CDCl_3_): δ 3.81 (3H, s, -NCH_3_), 6.26–6.27 (1H, m, ArH), 6.87 (1H, t, *J* = 2.0 Hz, ArH), 6.91–6.96 (2H, m, ArH), 7.02 (1H, dd, *J* = 8.4, 0.8 Hz, ArH), 7.40 (1H, d, *J* = 14.8 Hz, -C*H*=CHCO-), 7.48 (1H, t, *J* = 8.4 Hz, ArH), 7.89 (1H, dd, *J* = 8.0, 2.0 Hz, ArH), 7.92 (1H, d, *J* = 14.8 Hz, -CH=C*H*CO-), 13.14 (1H, s, OH). ^13^C-NMR (500 MHz, CDCl_3_): δ 34.4, 110.2, 113.3, 114.5, 118.5, 118.7, 120.2, 128.6, 129.2, 130.2, 132.8, 135.8, 163.4, 193.1. LCMS (ES+) *m*/*z* = 228.1 ([M + H]^+^, *t*_R_ = 1.68 min). HRMS (ESI+) *m*/*z* = 228.1020 [M + H]^+^ found, C_14_H_14_O_2_N^+^ required 228.1019.

*(E)-1-(4-Hydroxy-3-methoxyphenyl)-3-(1-methyl-1H-pyrrol-2-yl)prop-2-en-1-one* (**16**). A mixture of pyrrole aldehyde **7** (3.30 mL, 30.7 mmol), acetophenone **11** (5.02 g, 30.2 mmol) and KOH (10.2 g, 182 mmol) in absolute EtOH (100 mL) was reacted according to GP-D. The crude residue was purified by flash column chromatography (SiO_2_, PE/EtOAc; 5:1) and recrystallized from absolute EtOH to afford chalcone **16** (4.28 g, 55%) as a dark red-brown powdery solid. m.p. 150–152 °C. TLC *R*_f_ = 0.31 (PE/EtOAc; 1:1). IR ν_max_ (neat)/cm^−1^: 3177m(br) (O-H str), 2958w (C-H str), 2930w (C-H str), 1630m (C=O str), 1600m (C=C str), 1587m (C=C str), 1543s (C=C str), 1508s (C=C str), 1478m, 1380w, 1333m, 1270s, 1218m, 1196s, 1169s, 1052s, 1031s. ^1^H-NMR (500 MHz, CDCl_3_): δ 3.77 (3H, s, -NCH_3_), 3.99 (3H, s, -OCH_3_), 6.18 (1H, s, OH), 6.23 (1H, t, *J* = 3.2 Hz, ArH), 6.81 (1H, t, *J* = 2.0 Hz, ArH), 6.84 (1H, dd, *J* = 4.0, 1.6 Hz, ArH), 6.99 (1H, d, *J* = 8.8 Hz, ArH), 7.32 (1H, d, *J* = 15.2 Hz, -C*H*=CHCO-), 7.62–7.64 (2H, m, ArH), 7.81 (1H, d, *J* = 14.8 Hz, -CH=C*H*CO-). **^1^**^3^C-NMR (500 MHz, CDCl_3_): δ 34.3, 56.1, 109.6, 110.4, 112.0, 113.7, 116.4, 123.1, 127.5, 130.3, 131.5, 131.5, 146.8, 150.0, 188.1. LCMS (ES+) *m*/*z* = 258.1 ([M + H]^+^, *t*_R_ = 1.40 min). HRMS (ESI+) *m*/*z* =280.0935 [M + Na]^+^ found, C_15_H_15_O_3_NNa^+^ required 280.0944.

*(E)-1-(5-Bromo-2-hydroxyphenyl)-3-(furan-2-yl)prop-2-en-1-one* (**17**). A mixture of furan aldehyde **8** (1.20 mL, 14.5 mmol), acetophenone **12** (3.06 g, 14.2 mmol) and KOH (4.70 g, 83.8 mmol) in absolute EtOH (100 mL) was reacted according to GP-D. The crude residue was purified by recrystallization from MeOH to afford chalcone **17** (1.97 g, 47%) as a bright yellow-orange fluffy solid. m.p. 80–82 °C. TLC *R*_f_ = 0.50 (PE/EtOAc; 3:1). IR ν_max_ (neat)/cm^−1^: 3128w (C-H str), 3046w (C-H str), 2868w (C-H str), 1638m (C=O str), 1567s (C=C str), 1548s (C=C str), 1467s, 1364m, 1335s, 1296m, 1258s, 1202s, 1177s, 1077w, 1013s. ^1^H-NMR (500 MHz, CDCl_3_): δ 6.56–6.57 (1H, m, ArH), 6.82 (1H, d, *J* = 3.6 Hz, ArH), 6.93 (1H, d, *J* = 8.8 Hz, ArH), 7.45 (1H, d, *J* = 15.2 Hz, -C*H*=CHCO-), 7.56 (1H, dd, *J* = 9.2, 2.4 Hz, ArH), 7.60 (1H, d, *J* = 0.8 Hz, ArH), 7.70 (1H, d, *J* = 15.2 Hz, -CH=C*H*CO-), 8.00 (1H, d, *J* = 2.4 Hz, ArH), 12.83 (1H, s, OH). ^13^C-NMR (500 MHz, CDCl_3_): δ 110.4, 113.0, 116.8, 117.8, 120.5, 121.2, 131.8, 131.9, 138.8, 145.8, 151.3, 162.4, 192.3. LCMS (ES+) *m*/*z* = 293.1 ([M + H]^+^, *t*_R_ = 1.85 min). These characterisation data are in accordance with that previously reported in the literature [39].

*(E)-3-(Ferrocenyl)-1-(2-hydroxyphenyl)prop-2-en-1-one* (**18**). A mixture of ferrocene aldehyde **9** (4.77 g, 22.3 mmol), acetophenone **10** (2.70 mL, 22.4 mmol) and KOH (7.63 g, 136 mmol) in absolute EtOH (100 mL) was reacted according to GP-D. The crude residue was purified by recrystallization from MeOH to afford chalcone **18** (4.99 g, 67%) as a dark purple microcrystalline solid. m.p. 162–164 °C. TLC *R*_f_ = 0.43 (PE/EtOAc; 7:1). IR ν_max_ (neat)/cm^−1^: 3315w(br) (O-H str), 3088w (C-H str), 1742w (C=O str), 1627m, 1554s (C=C str), 1489s, 1440m, 1383w, 1347m, 1302s, 1271m, 1246m, 1206s, 1158m, 1104m, 1048w, 1023m, 1002w. ^1^H-NMR (500 MHz, CDCl_3_): δ 4.21 (5H, s, -C_5_H_5_), 4.55 (2H, t, *J* = 2.0 Hz, -C_5_H_4_), 4.65 (2H, t, *J* = 2.0 Hz, -C_5_H_4_), 6.94 (1H, t, *J* = 8.0 Hz, ArH), 7.03 (1H, dd, *J* = 8.5, 1.0 Hz, ArH), 7.26 (1H, d, *J* = 15.0 Hz, -C*H*=CHCO-, overlain by CDCl_3_), 7.49 (1H, t, *J* = 8.5 Hz, ArH), 7.87 (1H, dd, *J* = 8.0, 1.0 Hz, ArH), 7.91 (1H, d, *J* = 15.0 Hz, -CH=C*H*CO-), 13.07 (1H, s, OH). ^13^C-NMR (500 MHz, CDCl_3_): δ 69.3, 69.9, 71.8, 78.8, 116.7, 118.6, 118.6, 120.0, 129.3, 135.9, 147.9, 163.6, 192.7. LCMS (ES+) *m*/*z* = 333.1 ([M + H]^+^, *t*_R_ = 1.87 min). These characterisation data are in accordance with that previously reported in the literature [40].

*(E)-3-(Ferrocenyl)-1-(4-hydroxy-3-methoxyphenyl)prop-2-en-1-one* (**19**). A mixture of ferrocene aldehyde **9** (3.86 g, 18.0 mmol), acetophenone **11** (3.05 g, 18.4 mmol) and KOH (6.10 g, 109 mmol) in absolute EtOH (100 mL) was reacted according to GP-D. The crude residue was purified by flash column chromatography (SiO_2_, PE/EtOAc; 5:1) to afford chalcone **19** (5.61 g, 86%) as a dark purple microcrystalline solid. m.p. 78–80 °C. TLC *R*_f_ = 0.29 (PE/EtOAc; 2:1). IR ν_max_ (neat)/cm^−1^: 3098w(C-H str), 2835w (C-H str), 1743w, 1642m, (C=O str) 1588s (C=C str), 1563s (C=C str), 1511s (C=C str), 1462m, 1424s, 1359m, 1347m, 1281s, 1264s, 1189s, 1162s, 1026s, 1001w. ^1^H-NMR (500 MHz, CDCl_3_): δ. 3.95 (3H, s, -OCH_3_), 4.17 (5H, s, -C_5_H_5_), 4.47 (2H, t, *J* = 2.0 Hz, -C_5_H_4_), 4.59 (2H, t, *J* = 2.0 Hz, -C_5_H_4_), 6.54 (1H, br s, OH), 7.00 (1H, d, *J* = 8.0 Hz, ArH), 7.16 (1H, d, *J* = 15.0 Hz, -C*H*=CHCO-), 7.60 (1H, dd, *J* = 8.5, 1.5 Hz, ArH), 7.63 (1H, d, *J* = 1.5 Hz, ArH), 7.76 (1H, d, *J* = 15.5 Hz, -CH=C*H*CO-). ^13^C-NMR (500 MHz, CDCl_3_): δ 56.0, 68.8, 69.7, 71.2, 79.3, 110.5, 113.8, 118.5, 123.2, 131.1, 145.8, 146.8, 150.1, 187.9. LCMS (ES+) *m*/*z* = 363.1 ([M + H]^+^, *t*_R_ = 1.66 min). HRMS (ESI+) *m*/*z* = 363.0595 [M + H]^+^ found, C_20_H_19_O_3_Fe^+^ required 363.0600.

*(E)-1-(3-Methoxy-4-(prop-2-yn-1-yloxy)phenyl)-3-(4-methoxyphenyl)prop-2-en-1-one* (**20**). A mixture of chalcone **13** (1.02 g, 3.57 mmol), propargyl bromide (0.63 mL, 7.07 mmol) and anhydrous K_2_CO_3_ (1.49 g, 10.8 mmol) in dry acetone (50 mL) was reacted according to GP-C. The crude residue was purified by flash column chromatography (SiO_2_, CH_2_Cl_2_) to afford chalcone **20** (1.07 g, 93%) as a pale yellow powdery solid. m.p. 168–170 °C. TLC *R*_f_ = 0.41 (CH_2_Cl_2_). IR ν_max_ (neat)/cm^−1^: 3251w (C≡C-H str), 2990w (C-H str), 2835w (C-H str), 2116w (C≡C str), 1651s (C=O str), 1598s (C=C str), 1577s (C=C str), 1508s (C=C str), 1466m, 1421s, 1382w, 1323m, 1257s, 1229s, 1165s, 1145s, 1056w, 1015s. ^1^H-NMR (500 MHz, CDCl_3_): δ 2.57 (1H, t, *J* = 2.4 Hz, -OCH_2_C≡C*H*), 3.87 (3H, s, -OCH_3_), 3.98 (3H, s, -OCH_3_), 4.87 (2H, d, *J* = 2.4 Hz, -OC*H*_2_C≡CH), 6.95 (2H, d, *J* = 8.8 Hz, ArH), 7.11 (2H, d, *J* = 8.4 Hz, ArH), 7.44 (1H, d, *J* = 15.6 Hz, -C*H*=CHCO-), 7.62 (2H, d, *J* = 8.8 Hz, ArH), 7.65 (1H, d, *J* = 2.0 Hz, ArH), 7.68 (1H, dd, *J* = 8.4, 2.0 Hz, ArH), 7.80 (1H, d, *J* = 15.6 Hz, -CH=C*H*CO-). ^13^C-NMR (500 MHz, CDCl_3_): δ 55.4, 56.1, 56.6, 76.4, 77.8, 111.3, 112.3, 114.4, 119.3, 122.3, 127.7, 130.1, 132.6, 144.0, 149.7, 150.7, 161.6, 188.7. LCMS (ES+) *m*/*z* = 323.2 ([M + H]^+^, *t*_R_ = 1.56 min). HRMS (ESI+) *m*/*z* = 323.1265 [M + H]^+^ found, C_20_H_19_O_4_^+^ required 323.1278.

*(E)-1-(5-Bromo-2-(prop-2-yn-1-yloxy)phenyl)-3-(1-methyl-1H-indol-3-yl)prop-2-en-1-one* (**21**). A mixture of indole chalcone **14** (2.07 g, 5.81 mmol), propargyl bromide (1.50 mL, 16.8 mmol) and anhydrous K_2_CO_3_ (2.52 g, 18.2 mmol) in dry acetone (100 mL) was reacted according to GP-C. The crude residue was purified by flash column chromatography (SiO_2_, CH_2_Cl_2_) to afford chalcone **21** (2.20 g, 96%) as a bright yellow powdery solid. m.p. 126–128 °C. TLC *R*_f_ = 0.20 (CH_2_Cl_2_). IR ν_max_ (neat)/cm^−1^: 3257w (C≡C-H str), 3070w (C-H str), 2942w (C-H str), 2114w (C≡C str), 1638s (C=O str), 1587m (C=C str), 1544s (C=C str), 1524s (C=C str), 1473m, 1373s, 1269s, 1213m, 1176s, 1133m, 1073m, 1006s. ^1^H-NMR (500 MHz, CDCl_3_): δ 2.55 (1H, t, *J* = 2.4 Hz, -OCH_2_C≡C*H*), 3.82 (3H, s, -NCH_3_), 4.79 (2H, d, *J* = 2.4 Hz, -OC*H*_2_C≡CH), 7.03 (1H, d, *J* = 9.2 Hz, ArH), 7.26–7.38 (3H, m, ArH), 7.38 (1H, d, *J* = 16.0 Hz, -C*H*=CHCO-), 7.41 (1H, s, ArH), 7.55 (1H, dd, *J* = 8.8, 2.4 Hz, ArH), 7.77 (1H, d, *J* = 2.4 Hz, ArH), 7.88 (1H, d, *J* = 15.6 Hz, -CH=C*H*CO-), 8.01 (1H, d, *J* = 8.0 Hz, ArH). ^13^C-NMR (500 MHz, CDCl_3_): δ 33.3, 56.6, 76.4, 77.8, 110.0, 112.9, 114.3, 115.3, 121.0, 121.5, 121.6, 123.2, 126.0, 132.6, 132.9, 134.3, 134.8, 138.2, 138.6, 154.6, 191.0. LCMS (ES+) *m*/*z* = 396.1 ([M + H]^+^, *t*_R_ = 1.98 min). HRMS (ESI+) *m*/*z* = 394.0425 [M + H]^+^ found, C_21_H_17_NO_2_Br^+^ required 394.0443.

*(E)-1-(3-Methoxy-4-(prop-2-yn-1-yloxy)phenyl)-3-(1-methyl-1H-pyrrol-2-yl)prop-2-en-1-one* (**22**). A mixture of pyrrole chalcone **16** (1.00 g, 3.89 mmol), propargyl bromide (0.70 mL, 7.86 mmol) and anhydrous K_2_CO_3_ (1.61 g, 1.17 mmol) in dry acetone (50 mL) was reacted according to GP-C. The crude residue was purified by flash column chromatography (SiO_2_, PE/EtOAc; 3:1) to afford chalcone **22** (1.06 g, 92%) as a bright yellow powdery solid. m.p. 164–166 °C. TLC *R*_f_ = 0.41 (PE/EtOAc; 1:1). IR ν_max_ (neat)/cm^−1^: 3234m (C≡C-H str), 2995w (C-H str), 2866w (C-H str), 1739m (C=O str), 1645m, 1598s, (C=C str), 1561s (C=C str), 1515m (C=C str), 1483m, 1416w, 1374m, 1346s, 1272s, 1179m, 1129s, 1087w, 1042m, 1012m. ^1^H-NMR (500 MHz, CDCl_3_): δ 2.56 (1H, t, *J* = 2.5 Hz, -OCH_2_C≡C*H*), 3.78 (3H, s, -NCH_3_), 3.98 (3H, s, -OCH_3_), 4.86 (1H, d, *J* = 2.5 Hz, -OC*H*_2_C≡CH), 6.23–6.24 (1H, m, ArH), 6.82 (1H, t, *J* = 2.0 Hz, ArH), 6.85 (1H, dd, *J* = 4.0, 1.0 Hz, ArH), 7.10 (1H, d, *J* = 8.0 Hz, ArH), 7.32 (1H, d, *J* = 15.0 Hz, -C*H*=CHCO-), 7.64–7.67 (2H, m, ArH), 7.82 (1H, d, *J* = 15.0 Hz, -CH=C*H*CO-). ^13^C-NMR (500 MHz, CDCl_3_): δ 34.4, 56.1, 56.6, 76.4, 77.8, 109.7, 111.2, 112.1, 112.4, 116.4, 122.0, 127.6, 130.3, 131.7, 132.9, 149.6, 150.5, 188.1. LCMS (ES+) *m*/*z* = 296.2 ([M + H]^+^, *t*_R_ = 1.57 min). HRMS (ESI+) *m*/*z* = 294.1119 [M − H]^+^ found, C_18_H_16_O_3_N^+^ required 294.1125.

*(E)-3-(1-Methyl-1H-pyrrol-2-yl)-1-(2-(prop-2-yn-1-yloxy)phenyl)prop-2-en-1-one* (**23**). A mixture of pyrrole chalcone **15** (308 mg, 1.32 mmol), propargyl bromide (0.25 mL, 2.81 mmol) and anhydrous K_2_CO_3_ (568 mg, 4.11 mmol) in dry acetone (20 mL) was reacted according to GP-C. The crude residue was purified by flash column chromatography (SiO_2_, PE/EtOAc; 3:1) to afford chalcone **23** (352 mg, 98%) as a bright yellow viscous oil. TLC *R*_f_ = 0.34 (PE/EtOAc; 2:1). IR ν_max_ (neat)/cm^−1^: 3283w (C≡C-H str), 3072w (C-H str), 2923w (C-H str), 2120w (C≡C str), 1698w (C=O str), 1647m, 1598s (C=C str), 1580s (C=C str), 1564s (C=C str), 1480s, 1450m, 1330m, 1287s, 1217s, 1113w, 1057m, 1018s. ^1^H-NMR (500 MHz, CDCl_3_): δ 2.54 (1H, t, *J* = 2.4 Hz, -OCH_2_C≡C*H*), 3.74 (3H, s, -NCH_3_), 4.79 (2H, d, *J* = 2.4 Hz, -OC*H*_2_C≡CH), 6.20–6.21 (1H, m, ArH), 6.78–6.80 (2H, m, ArH), 7.08–7.12 (2H, m, ArH), 7.19 (1H, d, *J* = 15.2 Hz, -C*H*=CHCO-), 7.47 (1H, t, *J* = 8.8 Hz, ArH), 7.65 (1H, d, *J* = 15.6 Hz, -CH=C*H*CO-), 7.66 (1H, dd, *J* = 7.6, 2.0 Hz, ArH). ^13^C-NMR (500 MHz, CDCl_3_): δ 34.5, 56.4, 75.9, 78.2, 109.6, 112.8, 113.3, 121.8, 121.9, 127.7, 130.2, 130.5, 130.5, 131.1, 132.2, 155.7, 191.9. LCMS (ES+) *m*/*z* = 266.1 ([M + H]^+^, *t*_R_ = 1.57 min). HRMS (ESI+) *m*/*z* = 266.1183 [M + H]^+^ found, C_17_H_16_NO_2_^+^ required 266.1181.

*(E)-1-(5-Bromo-2-(prop-2-yn-1-yloxy)phenyl)-3-(furan-2-yl)prop-2-en-1-one* (**24**). A mixture of furan chalcone **17** (2.02 g, 6.89 mmol), propargyl bromide (1.30 mL, 14.6 mmol) and anhydrous K_2_CO_3_ (2.90 g, 21.0 mmol) in dry acetone (100 mL) was reacted according to GP-C. The crude residue was purified by flash column chromatography (SiO_2_, CH_2_Cl_2_) to afford chalcone **24** (2.14 g, 94%) as a pale yellow-brown powdery solid. m.p. 82–84 °C. TLC *R*_f_ = 0.21 (PE/CH_2_Cl_2_; 1:1). IR ν_max_ (neat)/cm^−1^: 3224m (C≡C-H str), 3118w (C-H str), 2118w (C≡C str), 1660m (C=O str), 1600s (C=C str), 1586m (C=C str), 1554m (C=C str), 1476m, 1394m, 1313w, 1276m, 1211s, 1180m, 1131m, 1079w, 1048m, 1012s. ^1^H-NMR (500 MHz, CDCl_3_): δ 2.54 (1H, t, *J* = 2.4 Hz, -OCH_2_C≡C*H*), 4.78 (2H, d, *J* = 2.4 Hz, -OC*H*_2_C≡CH), 6.50–6.51 (1H, m, ArH), 6.70 (1H, d, *J* = 3.6 Hz, ArH), 7.03 (1H, d, *J* = 8.8 Hz, ArH), 7.21 (1H, d, *J* = 15.6 Hz, -C*H*=CHCO-), 7.40 (1H, d, *J* = 15.6 Hz, -CH=C*H*CO-), 7.52 (1H, d, *J* = 1.2 Hz, ArH), 7.56 (1H, dd, *J* = 8.8, 2.4 Hz, ArH), 7.73 (1H, d, *J* = 2.4 Hz, ArH). ^13^C-NMR (500 MHz, CDCl_3_): δ 56.6, 76.5, 77.6, 112.6, 114.3, 115.4, 116.2, 123.8, 130.4, 131.6, 133.0, 135.0, 145.1, 151.5, 154.8, 190.5. LCMS (ES+) *m*/*z* = 333.0 ([M + H]^+^, *t*_R_ = 1.89 min). HRMS (ESI+) *m*/*z* = 330.9973 [M + H]^+^ found, C_16_H_12_O_3_Br^+^ required 330.9970.

*(E)-3-(Ferrocenyl)-1-(3-methoxy-4-(prop-2-yn-1-yloxy)phenyl)prop-2-en-1-one* (**25**). A mixture of ferrocene chalcone **19** (1.01 g, 2.79 mmol), propargyl bromide (0.50 mL, 5.61 mmol) and anhydrous K_2_CO_3_ (1.18 g, 8.56 mmol) in dry acetone (50 mL) was reacted according to GP-C. The crude residue was purified by flash column chromatography (SiO_2_, CH_2_Cl_2_) to afford chalcone **25** (1.01 g, 91%) as a dark red-purple microcrystalline solid. m.p. 128–130 °C. TLC *R*_f_ = 0.47 (0.5% MeOH/CH_2_Cl_2_). IR ν_max_ (neat)/cm^−1^: 3225m (C≡C-H str), 3008w (C-H str), 2939w (C-H str), 2114w (C≡C str), 1646s (C=O str), 1595m (C=C str), 1567s (C=C str), 1506m (C=C str), 1456m, 1413s, 1345w, 1295s, 1259s, 1194m, 1159s, 1136s, 1107m, 1060w, 1044m, 1020s. ^1^H-NMR (500 MHz, CDCl_3_): δ 2.56 (1H, t, *J* = 2.5 Hz, -OCH_2_C≡C*H*), 3.96 (3H, s, -OCH_3_), 4.18 (5H, s, -C_5_H_5_), 4.48 (2H, t, *J* = 2.0 Hz, -C_5_H_4_), 4.60 (2H, t, *J* = 2.0 Hz, -C_5_H_4_), 4.85 (2H, d, *J* = 2.0 Hz, -OC*H*_2_C≡CH), 7.09 (1H, d, *J* = 8.5 Hz, ArH), 7.15 (1H, d, *J* = 15.5 Hz, -C*H*=CHCO-), 7.61–7.63 (2H, m, ArH), 7.75 (1H, d, *J* = 15.0 Hz, -CH=C*H*CO-). ^13^C-NMR (500 MHz, CDCl_3_): δ 56.0, 56.5, 68.9, 69.7, 71.2, 76.3, 77.8, 79.3, 111.2, 112.3, 118.5, 122.1, 132.6, 146.0, 149.5, 150.4, 187.9. LCMS (ES+) *m*/*z* = 401.1 ([M + H]^+^, *t*_R_ = 1.81 min). HRMS (ESI+) *m*/*z* = 401.0823 [M + H]^+^ found, C_23_H_21_O_3_Fe^+^ required 401.0840.

*(E)-3-(Ferrocenyl)-1-(2-(prop-2-yn-1-yloxy)phenyl)prop-2-en-1-one* (**26**). A mixture of chalcone **18** (1.02 g, 3.07 mmol), propargyl bromide (0.54 mL, 6.02 mmol) and anhydrous K_2_CO_3_ (1.20 g, 8.68 mmol) in dry acetone (50 mL) was reacted according to GP-C. The crude residue was purified by flash column chromatography (SiO_2_, PE/CH_2_Cl_2_; 1:1) to afford chalcone **26** (919 mg, 81%) as a bright red powdery solid. m.p. 90–92 °C. TLC *R*_f_ = 0.38 (CH_2_Cl_2_). IR ν_max_ (neat)/cm^−1^: 3257m (C≡C-H str), 2924w (C-H str), 2850w (C-H str), 2117w (C≡C str), 1654s (C=O str), 1587s (C=C str), 1480m, 1448m, 1356w, 1345w, 1289m, 1211s, 1159w, 1104m, 1058m, 1016s, 1001s. ^1^H-NMR (500 MHz, CDCl_3_): δ 2.56 (1H, t, *J* = 2.5 Hz, -OCH_2_C≡C*H*), 4.19 (5H, s, -C_5_H_5_), 4.46 (2H, t, *J* = 2.0 Hz, -C_5_H_4_), 4.56 (2H, t, *J* = 2.0 Hz, -C_5_H_4_), 4.79 (2H, d, *J* = 2.5 Hz, -OC*H*_2_C≡CH), 6.95 (1H, d, *J* = 16.0 Hz, -C*H*=CHCO-), 7.09–7.13 (2H, m, ArH), 7.47 (1H, t, *J* = 9.0 Hz, ArH), 7.50 (1H, d, *J* = 15.5 Hz, -CH=C*H*CO-), 7.58 (1H, dd, *J* = 7.5, 1.5 Hz, ArH). ^13^C-NMR (500 MHz, CDCl_3_): δ 56.3, 69.0, 69.8, 71.2, 76.0, 78.2, 79.0, 113.3, 121.7, 124.6, 130.1, 130.5, 131.9, 146.2, 155.4, 192.5. LCMS (ES+) *m*/*z* = 371.1 ([M + H]^+^, *t*_R_ = 1.96 min). HRMS (ESI+) *m*/*z* = 370.0639 [M]^+^ found, C_22_H_18_O_2_Fe^+^ required 370.0651.

*3-(Prop-2-yn-1-yloxy)flavone* (**30**). A mixture of 3-hydroxyflavone **27** (2.06 g, 8.65 mmol), propargyl bromide (1.50 mL, 16.8 mmol) and anhydrous K_2_CO_3_ (3.48 g, 25.2 mmol) in dry acetone (100 mL) was reacted according to GP-C. The crude residue was purified by flash column chromatography (SiO_2_, CH_2_Cl_2_) to afford flavone **30** (2.38 g, 99%) as a pale yellow-white powdery solid. m.p. 104–106 °C. TLC *R*_f_ = 0.24 (CH_2_Cl_2_). IR ν_max_ (neat)/cm^−1^: 3254m (C≡C-H str), 2946w (C-H str), 2114w (C≡C str), 1612s (C=O str), 1600s, 1557s (C=C str), 1468s, 1445m, 1399s, 1354m, 1345m, 1279w, 1234w, 1195s, 1185s, 1148s, 1110m, 1079w, 1036m. ^1^H-NMR (500 MHz, CDCl_3_): δ 2.33 (1H, t, *J* = 2.4 Hz, -OCH_2_C≡C*H*), 5.00 (2H, d, *J* = 2.4 Hz, -OC*H*_2_C≡CH), 7.42 (1H, t, *J* = 8.0 Hz, ArH), 7.50–7.54 (3H, m, ArH), 7.56 (1H, d, *J* = 8.4 Hz, ArH), 7.70 (1H, t, *J* = 8.4 Hz, ArH), 8.14–8.16 (2H, m, ArH), 8.27 (1H, dd, *J* = 8.0, 1.6 Hz, ArH). ^13^C-NMR (500 MHz, CDCl_3_): δ 59.1, 76.1, 78.5, 118.0, 123.9, 124.7, 125.7, 128.3, 128.9, 130.7, 130.9, 133.5, 138.5, 155.2, 156.7, 174.8. LCMS (ES+) *m*/*z* = 277.5 ([M + H]^+^, *t*_R_ = 4.47 min). These characterisation data are in accordance with that previously reported in the literature [23].

*6-(Prop-2-yn-1-yloxy)flavone* (**31**). A mixture of 6-hydroxyflavone **28** (2.00 g, 8.39 mmol), propargyl bromide (1.50 mL, 16.8 mmol) and anhydrous K_2_CO_3_ (3.50 g, 25.3 mmol) in dry acetone (100 mL) was reacted according to GP-C. The crude residue was purified by flash column chromatography (SiO_2_, 1% MeOH/CH_2_Cl_2_) to afford flavone **31** (2.03 g, 87%) as a white crystalline solid. m.p. 170–172 °C. TLC *R*_f_ = 0.44 (1% MeOH/CH_2_Cl_2_). IR ν_max_ (neat)/cm^−1^: 3266m (C≡C-H str), 3059w (C-H str), 2116w (C≡C str), 1623s (C=O str), 1606s, 1582s (C=C str), 1568s (C=C str), 1497m, 1482m, 1455s, 1365s, 1283m, 1259m, 1234m, 1186s, 1144s, 1075m, 1048m, 1007s. ^1^H-NMR (500 MHz, CDCl_3_): δ 2.57 (1H, t, *J* = 2.4 Hz, -OCH_2_C≡C*H*), 4.76 (2H, d, *J* = 2.4 Hz, -OC*H*_2_C≡CH), 6.77 (1H, s, -C=CH), 7.31 (1H, dd, *J* = 9.2, 3.2 Hz, ArH), 7.46–7.51 (4H, m, ArH), 7.65 (1H, d, *J* = 3.2 Hz, ArH), 7.86–7.88 (2H, m, ArH). ^13^C-NMR (500 MHz, CDCl_3_): δ 56.3, 76.0, 77.7, 106.4, 106.7, 119.6, 123.9, 124.4, 126.1, 128.9, 131.4, 131.6, 151.3, 154.7, 163.0, 177.9. LCMS (ES+) *m*/*z* = 277.0 ([M + H]^+^, *t*_R_ = 1.56 min). These characterisation data are in accordance with that previously reported in the literature [24].

*7-(Prop-2-yn-1-yloxy)flavone* (**32**). A mixture of 7-hydroxyflavone **29** (990 mg, 4.16 mmol), propargyl bromide (0.748 mL, 8.39 mmol) and anhydrous K_2_CO_3_ (1.75 g, 12.7 mmol) and in dry acetone (100 mL) was reacted according to GP-C. The crude residue was purified by flash column chromatography (SiO_2_, 1% MeOH/CH_2_Cl_2_) to afford flavone **32** (1.10 g, 95%) as an off-white powdery solid. m.p. 216–218 °C. TLC *R*_f_ = 0.44 (2% MeOH/CH_2_Cl_2_). IR ν_max_ (neat)/cm^−1^: 3194w (C≡C-H str), 2921w (C-H str), 2115w (C≡C str), 1619s (C=O str), 1591s (C=C str), 1568s (C=C str), 1496m, 1451s, 1435s, 1378s, 1350m, 1268m, 1229m, 1168s, 1135s, 1091s, 1017s. ^1^H-NMR (500 MHz, CDCl_3_): δ 2.62 (1H, t, *J* = 2.4 Hz, -OCH_2_C≡C*H*), 4.83 (2H, d, *J* = 2.4 Hz, -OC*H*_2_C≡CH), 6.77 (1H, s, -C=CH), 7.05 (1H, dd, *J* = 8.8, 2.4 Hz, ArH), 7.09 (1H, d, *J* = 2.4 Hz, ArH), 7.51–7.55 (3H, m, ArH), 7.90–7.93 (2H, m, ArH), 8.17 (1H, d, *J* = 8.8 Hz, ArH). ^13^C-NMR (500 MHz, CDCl_3_): δ 56.2, 76.6, 77.3, 101.8, 107.6, 114.7, 118.4, 126.2, 127.2, 129.0, 131.5, 131.8, 157.7, 161.9, 163.1, 177.7. LCMS (ES+) *m*/*z* = 277.0 ([M + H]^+^, *t*_R_ = 1.54 min). These characterisation data are in accordance with that previously reported in the literature [24].

*4-(Prop-2-yn-1-yloxy)benzaldehyde* (**34**). A mixture of benzaldehyde **33** (10.2 g, 83.7 mmol), propargyl bromide (14.6 mL, 164 mmol) and anhydrous K_2_CO_3_ (22.7 g, 165 mmol) in dry acetone (200 mL) was reacted according to GP-C. The crude residue was purified by flash column chromatography (SiO_2_, CH_2_Cl_2_) to afford benzaldehyde **34** (12.2 g, 91%) as a white crystalline solid. m.p. 88–90 °C. TLC *R*_f_ = 0.49 (CH_2_Cl_2_). IR ν_max_ (neat)/cm^−1^: 3205m (C≡C-H str), 2971w (C-H str), 2837w (C-H str), 2123w (C≡C str), 1739s (C=O str), 1679s, 1601s, 1576s (C=C str), 1508m (C=C str), 1428w, 1384s, 1245s, 1217s, 1170s, 1009s. ^1^H-NMR (500 MHz, CDCl_3_): δ 2.58 (1H, t, *J* = 2.4 Hz, -OCH_2_C≡C*H*), 4.77 (2H, d, *J* = 2.4 Hz, -OC*H*_2_C≡CH), 7.08 (2H, d, *J* = 8.8 Hz, ArH), 7.85 (2H, d, *J* = 8.8 Hz, ArH), 9.89 (1H, s, CHO). ^13^C-NMR (500 MHz, CDCl_3_): δ 55.9, 76.3, 77.5, 115.1, 130.5, 131.8, 162.3, 190.7. LCMS (ES+) *m*/*z* = 161.1 ([M + H]^+^, *t*_R_ = 1.65 min). These characterisation data are in accordance with that previously reported in the literature [41].

*(E)-1-(2-Hydroxyphenyl)-3-(4-(prop-2-yn-1-yloxy)phenyl)prop-2-en-1-one* (**35**). A mixture of benzaldehyde **34** (5.04 g, 31.5 mmol), acetophenone **10** (3.76 mL, 31.2 mmol), and KOH (10.6 g, 189 mmol) in absolute EtOH (100 mL) was reacted according to GP-D. The crude residue was purified by recrystallization from MeOH to afford chalcone **35** (6.67 g, 77%) as a bright yellow powdery solid. m.p. 144–146 °C. TLC *R*_f_ = 0.45 (PE/EtOAc; 3:1). IR ν_max_ (neat)/cm^−1^: 3487w (O-H str), 3240m (C≡C-H str), 2923w (C-H str), 2124w (C≡C str), 1634s (C=O str), 1604m, 1559s (C=C str), 1509s (C=C str), 1488s, 1442m, 1368m, 1299s, 1270m, 1203m, 1177s, 1159s, 1027s. ^1^H-NMR (500 MHz, CDCl_3_): δ 2.58 (1H, t, *J* = 2.4 Hz, -OCH_2_C≡C*H*), 4.75 (2H, d, *J* = 2.4 Hz, -OC*H*_2_C≡CH), 6.94 (1H, t, *J* = 7.6 Hz, ArH), 7.03 (3H, d, *J* = 8.4 Hz, ArH), 7.49 (1H, t, *J* = 8.4 Hz, ArH), 7.54 (1H, d, *J* = 15.2 Hz, -C*H*=CHCO-), 7.63 (2H, d, *J* = 8.8 Hz, ArH), 7.89 (1H, d, *J* = 15.2 Hz, -CH=C*H*CO-), 7.90–7.92 (1H, m, ArH), 12.93 (1H, s, OH). ^13^C-NMR (500 MHz, CDCl_3_): δ 55.8, 76.1, 77.9, 115.3, 118.0, 118.5, 118.7, 120.0, 128.0, 129.5, 130.4, 136.1, 145.0, 159.7, 163.5, 193.5. LCMS (ES+) *m*/*z* = 279.1 ([M + H]^+^, *t*_R_ = 1.75 min). HRMS (ESI+) *m*/*z* = 279.1005 [M + H]^+^ found, C_18_H_15_O_3_^+^ required 279.1016.

*3-Hydroxy-4′-(prop-2-yn-1-yloxy)flavone* (**36**). A mixture of chalcone **35** (2.02 g, 7.26 mmol), 16% NaOH (14.4 mL) and 15% H_2_O_2_ (7.19 mL) in MeOH (50 mL) was reacted according to GP-F. The crude residue was purified by flash column chromatography (SiO_2_, 1% MeOH/CH_2_Cl_2_) to afford flavonol **36** (1.56 g, 73%) as a pale yellow-white powdery solid. m.p. 194–196 °C. TLC *R*_f_ = 0.44 (0.5% MeOH/CH_2_Cl_2_). IR ν_max_ (neat)/cm^−1^: 3271s (C≡C-H str), 3260w (O-H str), 2979w (C-H str), 2119w (C≡C str), 1599s (C=O str), 1562s (C=C str), 1507m (C=C str), 1483m, 1470m, 1427m, 1411m, 1233s, 1216m, 1180s, 1120s, 1109s, 1017s. ^1^H-NMR (500 MHz, CDCl_3_): δ 2.58 (1H, t, *J* = 2.4 Hz, -OCH_2_C≡C*H*), 4.79 (2H, d, *J* = 2.4 Hz, -OC*H*_2_C≡CH), 7.00 (1H, br s, OH), 7.14 (2H, d, *J* = 9.2 Hz, ArH), 7.42 (1H, t, *J* = 8.0 Hz, ArH), 7.58 (1H, d, *J* = 8.4 Hz, ArH), 7.70 (1H, t, *J* = 8.4 Hz, ArH), 8.25–8.28 (3H, m, ArH). ^13^C-NMR (500 MHz, CDCl_3_): δ 55.8, 76.0, 78.0, 115.0, 118.2, 120.7, 124.4, 124.4, 125.4, 129.5, 133.4, 137.8, 145.0, 155.3, 158.9, 173.2. LCMS (ES+) *m*/*z* = 293.1 ([M + H]^+^, *t*_R_ = 1.62 min). HRMS (ESI+) *m*/*z* = 315.0618 [M + Na]^+^ found, C_18_H_12_O_4_Na^+^ required 315.0628.

*1-(2-Hydroxy-4-methoxyphenyl)-2-(4-hydroxyphenyl)ethan-1-one* (**41**). A mixture of phenol **37** (8.70 mL, 79.32 mmol) and phenylacetic acid **39** (10.1 g, 66.3 mmol) in BF_3_·OEt_2_ (85.0 mL, 677 mmol) was reacted according to GP-G. The crude residue was purified by flash column chromatography (SiO_2_, CH_2_Cl_2_) to afford phenylethanone **41** (13.2 g, 78%) as a white powdery solid. m.p. 158–160 °C. TLC *R*_f_ = 0.38 (1% MeOH/CH_2_Cl_2_). IR ν_max_ (neat)/cm^−1^: 3469w(br) (O-H str), 3221w(br ) (O-H str), 2984w (C-H str), 2904w (C-H str), 1706w (C=O str), 1613s (C=C str), 1518s (C=C str), 1450m, 1437s, 1381m, 1354s, 1291s, 1224s, 1202s, 1127s, 1021m. ^1^H-NMR (500 MHz, (CD_3_)_2_CO): δ 3.85 (3H, s, -OCH_3_), 4.19 (2H, s, -COCH_2_), 6.42 (1H, d, *J* = 2.8 Hz, ArH), 6.49 (1H, dd, *J* = 8.8, 2.4 Hz, ArH), 6.80 (2H, d, *J* = 8.8 Hz, ArH), 7.16 (2H, d, *J* = 8.4 Hz, ArH), 7.98 (1H, d, *J* = 9.2 Hz, ArH), 8.29 (1H, br s, OH), 12.80 (1H, s, OH). ^13^C-NMR (500 MHz, (CD_3_)_2_CO): δ 43.7, 55.4, 101.0, 107.5, 113.2, 115.6, 125.9, 130.6, 133.1, 156.6, 166.0, 166.5, 203.5. LCMS (ES+) *m*/*z* = 259.1 ([M + H]^+^, *t*_R_ = 1.47 min). These characterisation data are in accordance with that previously reported in the literature [42].

*1-(2,4-Dihydroxyphenyl)-2-(4-methoxyphenyl)ethan-1-one* (**42**). A mixture of phenol **38** (8.00 g, 72.7 mmol) and phenylacetic acid **40** (10.2 g, 61.5 mmol) in BF_3_·OEt_2_ (70.0 mL, 557 mmol) was reacted according to GP-G. The crude residue was purified by recrystallization from CHCl_3_ to afford phenylethanone **42** (5.67 g, 36%) as a pale yellow-white powdery solid. m.p. 168–170 °C. TLC *R*_f_ = 0.50 (1% MeOH/CH_2_Cl_2_). IR ν_max_ (neat)/cm^−1^: 3349s(br) (O-H str), 2917w (C-H str), 2838w (C-H str), 1614s (C=O str), 1607s (C=C str), 1590s (C=C str), 1510s (C=C str), 1500m (C=C str), 1436m, 1412w, 1351s, 1299m, 1239m, 1174s, 1129m, 1105w, 1023s. ^1^H-NMR (500 MHz, (CD_3_)_2_CO): δ 3.76 (3H, s, -OCH_3_), 4.22 (2H, s, -COCH_2_), 6.33 (1H, d, *J* = 2.0 Hz, ArH), 6.44 (1H, dd, *J* = 9.2, 2.4 Hz, ArH), 6.88 (2H, d, *J* = 8.8 Hz, ArH), 7.26 (2H, d, *J* = 8.8 Hz, ArH), 7.96 (1H, d, *J* = 8.8 Hz, ArH), 9.46 (1H, s, OH), 12.73 (1H, s, OH). ^13^C-NMR (500 MHz, (CD_3_)_2_CO): δ 43.6, 54.8, 103.0, 108.2, 112.8, 114.1, 127.3, 130.7, 133.7, 159.0, 165.0, 166.1, 203.1. LCMS (ES+) *m*/*z* = 259.1 ([M + H]^+^, *t*_R_ = 1.52 min). These characterisation data are in accordance with that previously reported in the literature [43].

*4′-Hydroxy-7-methoxyisoflavone* (**43**). A mixture of phenylethanone **41** (2.00 g, 7.74 mmol), BF_3_·OEt_2_ (3.90 mL, 31.1 mmol) and MeSO_2_Cl (1.80 mL, 23.3 mmol) in dry DMF (50 mL) was reacted according to GP-H. The crude residue was purified by flash column chromatography (SiO_2_, PE/EtOAc; 2:1) to afford isoflavone **43** (1.18 g, 57%) as a white powdery solid. m.p. 232–234 °C. TLC *R*_f_ = 0.39 (PE/EtOAc; 1:1). IR ν_max_ (neat)/cm^−1^: 3210m(br) (O-H str), 3014w (C-H str), 1621s (C=O str), 1583s (C=C str), 1563m (C=C str), 1517s (C=C str), 1439s, 1373w, 1253s, 1205w, 1170w, 1095m, 1050m, 1019m. ^1^H-NMR (500 MHz, (CD_3_)_2_CO): δ 3.97 (3H, s, -OCH_3_), 6.89 (2H, d, *J* = 8.4 Hz, ArH), 7.04–7.07 (2H, m, ArH), 7.48 (2H, d, *J* = 8.8 Hz, ArH), 8.10 (1H, d, *J* = 8.8 Hz, ArH), 8.19 (1H, s, -C=CH), 8.44 (1H, s, OH). ^13^C-NMR (500 MHz, (CD_3_)_2_CO): δ 55.8, 100.4, 114.7, 115.2, 118.5, 123.7, 124.7, 127.4, 130.4, 152.7, 157.6, 158.2, 164.4, 175.1. LCMS (ES+) *m*/*z* = 269.1 ([M + H]^+^, *t*_R_ = 1.41 min). These characterisation data are in accordance with that previously reported in the literature [42].

*7-Hydroxy-4’-methoxyisoflavone* (**44**). A mixture of phenylethanone **42** (2.02 g, 7.82 mmol), BF_3_·OEt_2_ (3.90 mL, 31.1 mmol) and MeSO_2_Cl (1.80 mL, 23.2 mmol) in dry DMF (50 mL) was reacted according to GP-H. The crude residue was purified by flash column chromatography (SiO_2_, PE/EtOAc; 1:1) and recrystallized from CHCl_3_ to afford isoflavone **44** (1.62 g, 77%) as a dark yellow powdery solid. m.p. 242–244 °C. TLC *R*_f_ = 0.26 (PE/EtOAc; 1:1). IR ν_max_ (neat)/cm^−1^: 3126s(br) (O-H str), 2993w (C-H str), 2836w (C-H str), 1637m (C=O str), 1621m, 1594s (C=C str), 1568m (C=C str), 1512s (C=C str), 1451s, 1384m, 1273m, 1178s, 1099m, 1045m, 1025s. ^1^H-NMR (500 MHz, (CD_3_)_2_CO): δ 3.20 (1H, br s, OH), 3.83 (3H, s, -OCH_3_), 6.92 (1H, d, *J* = 2.0 Hz, ArH), 6.98 (2H, d, *J* = 9.2 Hz, ArH), 7.01 (1H, dd, *J* = 8.8, 2.4 Hz, ArH), 7.56 (2H, d, *J* = 8.8 Hz, ArH), 8.07 (1H, d, *J* = 8.4 Hz, ArH), 8.18 (1H, s, -C=CH). ^13^C-NMR (500 MHz, (CD_3_)_2_CO): δ 54.9, 102.5, 113.7, 115.1, 117.9, 124.3, 124.9, 127.8, 130.4, 152.8, 158.2, 159.8, 162.6, 175.0. LCMS (ES+) *m*/*z* = 269.1 ([M + H]^+^, *t*_R_ = 1.47 min). These characterisation data are in accordance with that previously reported in the literature [44].

*7-Methoxy-(4′-(prop-2-yn-1-yloxy)isoflavone* (**45**). A mixture of isoflavone **43** (602 mg, 2.25 mmol), propargyl bromide (0.50 mL, 5.61 mmol) and anhydrous K_2_CO_3_ (1.09 g, 7.91 mmol) in dry acetone (50 mL) was reacted according to GP-C. The crude residue was purified by flash column chromatography (SiO_2_, 1% MeOH/CH_2_Cl_2_) to afford isoflavone **45** (641 mg, 93%) as a white powdery solid. m.p. 162–164 °C. TLC *R*_f_ = 0.45 (1% MeOH/CH_2_Cl_2_). IR ν_max_ (neat)/cm^−1^: 3289m (C≡C-H str), 3083w (C-H str), 2953w (C-H str), 1645s (C=O str), 1624s, 1606s, 1579m (C=C str), 1513s (C=C str), 1456m, 1442s, 1378s, 1285m, 1262s, 1245s, 1192s, 1176s, 1103s, 1050w, 1017s. ^1^H-NMR (500 MHz, CDCl_3_): δ 2.54 (1H, t, *J* = 2.4 Hz, -OCH_2_C≡C*H*), 3.92 (3H, s, -OCH_3_), 4.73 (2H, d, *J* = 2.4 Hz, -OC*H*_2_C≡CH), 6.86 (2H, d, *J* = 2.0 Hz, ArH), 7.00 (1H, dd, *J* = 8.8, 2.0 Hz, ArH), 7.05 (2H, d, *J* = 8.8 Hz, ArH), 7.52 (2H, d, *J* = 8.8 Hz, ArH), 7.93 (1H, s, -C=CH), 8.21 (2H, d, *J* = 8.8 Hz, ArH). ^13^C-NMR (500 MHz, CDCl_3_): δ 55.8, 75.6, 78.5, 100.1, 114.5, 114.9, 118.4, 124.7, 125.2, 127.8, 130.1, 152.1, 157.5, 157.9, 164.0, 175.7. LCMS (ES+) *m*/*z* = 307.1 ([M + H]^+^, *t*_R_ = 1.59 min). HRMS (ESI+) *m*/*z* =307.0959 [M + H]^+^ found, C_19_H_15_O_4_^+^ required 307.0965.

*4′-Methoxy-7-(prop-2-yn-1-yloxy)isoflavone* (**46**). A mixture of isoflavone **44** (810 mg, 3.02 mmol), propargyl bromide (0.55 mL, 6.17 mmol) and anhydrous K_2_CO_3_ (1.25 g, 9.01 mmol) in dry acetone (50 mL) was reacted according to GP-C. The crude residue was purified by flash column chromatography (SiO_2_, CH_2_Cl_2_) to afford isoflavone **46** (550 mg, 60%) as a white powdery solid. m.p. 150–152 °C. TLC *R*_f_ = 0.32 (0.5% MeOH/CH_2_Cl_2_). IR ν_max_ (neat)/cm^−1^: 3281m (C≡C-H str), 3259m, 3078w (C-H str), 2950w (C-H str), 2116w (C≡C str), 1624s (C=O str), 1608s, 1597s (C=C str), 1565m (C=C str), 1513s (C=C str), 1440s, 1373m, 1326w, 1295m, 1271s, 1237s, 1176s, 1109w, 1095s, 1052m, 1033s, 1016s. ^1^H-NMR (500 MHz, CDCl_3_): δ 2.61 (1H, t, *J* = 2.0 Hz, -OCH_2_C≡C*H*), 3.85 (3H, s, -OCH_3_), 4.81 (1H, d, *J* = 2.4 Hz, -OC*H*_2_C≡CH), 6.98 (2H, d, *J* = 8.4 Hz, ArH), 6.98 (1H, d, *J* = 2.4 Hz, ArH), 7.05 (1H, dd, *J* = 8.8, 2.4 Hz, ArH), 7.51 (2H, d, *J* = 8.8 Hz, ArH), 7.93 (1H, s, -C=CH), 8.24 (1H, d, *J* = 8.8 Hz, ArH). ^13^C-NMR (500 MHz, CDCl_3_): δ 55.3, 56.2, 76.6, 77.3, 101.5, 113.9, 114.8, 119.0, 124.1, 124.9, 127.9, 130.1, 152.1, 157.6, 159.6, 161.6, 175.8. LCMS (ES+) *m*/*z* = 307.1 ([M + H]^+^, *t*_R_ = 1.62 min). These characterisation data are in accordance with that previously reported in the literature [45].

*4-(Prop-2-yn-1-yloxy)-2H-chromen-2-one* (**48**). A mixture of 4-hydroxycoumarin **47** (5.06 g, 31.2 mmol), propargyl bromide (6.20 mL, 69.6 mmol) and anhydrous K_2_CO_3_ (8.74 g, 63.2 mmol) in dry acetone (100 mL) was reacted according to GP-C. The crude residue was purified by flash column chromatography (SiO_2_, CH_2_Cl_2_) to afford coumarin **48** (3.60 g, 58%) as a white fluffy solid. m.p. 154–156 °C. TLC *R*_f_ = 0.24 (CH_2_Cl_2_). IR ν_max_ (neat)/cm^−1^: 3281m (C≡C-H str), 3240m, 3078w (C-H str), 2131w (C≡C str), 1714s (C=O str), 1688m, 1622s, 1610m (C=C str), 1568m (C=C str), 1493m, 1453m, 1409m, 1361m, 1329w, 1274s, 1248s, 1194w, 1179m, 1155w, 1145m, 1107s, 1032w. ^1^H-NMR (500 MHz, CDCl_3_): δ 2.68 (1H, t, *J* = 2.4 Hz, -OCH_2_C≡C*H*), 4.88 (2H, d, *J* = 2.4 Hz, -OC*H*_2_C≡CH), 5.84 (1H, s, -C=C*H*), 7.29–7.34 (2H, m, ArH), 7.57 (1H, t, *J* = 8.4 Hz, ArH), 7.84 (1H, dd, *J* = 8.0, 1.2 Hz, ArH). ^13^C-NMR (500 MHz, CDCl_3_): δ 56.8, 75.7, 77.9, 91.7, 115.4, 116.8, 123.1, 124.0, 132.6, 153.3, 162.4, 164.2. LCMS (ES+) *m*/*z* = 201.1 ([M + H]^+^, *t*_R_ = 1.47 min). These characterisation data are in accordance with that previously reported in the literature [46].

*2-(2-Chloro-1-iminoethyl)-1,3,5-benzentriol hydrochloride* (**50**). To a stirred solution of phloroglucinol **49** (5.02 g, 39.8 mmol) and chloroacetonitrile (2.50 mL, 39.5 mmol) in Et_2_O (100 mL) was added anhydrous ZnCl_2_ (0.558 g, 4.09 mmol). The reaction mixture was cooled to 0 °C and HCl gas was bubbled through the solution for 30 min. The resulting mixture was stirred at 0 °C for 3 h and further 24 h at room temperature. The resulting suspension was filtered and the precipitate was washed with ice-cold Et_2_O (2 × 50 mL) and suction-dried to afford hydrochloric salt **50** (1.87 g, 20%) as a pale yellow-white powdery solid and was used without further purification in the next step. m.p. 240–242 °C. IR ν_max_ (neat)/cm^−1^: 3389m (N-H str), 3257m(br) (O-H str), 3184s(br) (O-H str), 2968w (C-H str), 1646m, 1616s (C=C str), 1591s (C =C str), 1531w (C=C str), 1457m, 1383m, 1366s, 1291m, 1248s, 1175s, 1128w, 1065m, 1050s, 1024w. ^1^H-NMR (500 MHz, DMSO-*d*_6_): δ 5.46 (2H, s, -CH_2_-), 6.07 (1H, d, *J* = 1.2 Hz, ArH), 6.26 (1H, d, *J* = 1.2 Hz, ArH), 9.90 (1H, s, OH), 10.84 (1H, s, OH), 11.80 (1H, br s, NH), 12.54 (1H, s, OH). ^13^C-NMR (500 MHz, DMSO-*d*_6_): δ 75.3, 90.1, 96.9, 99.3, 160.4, 172.8, 173.7, 176.0. These characterisation data are in accordance with that previously reported in the literature [47].

*2,4,6-Trihydroxy-2-chloroacetophenone* (**51**). A mixture of imine salt **50** (1.80 g, 7.56 mmol) and 1 M HCl (100 mL) were heated at reflux with stirring for 1 h. The resulting red solution was blown under a steady stream of nitrogen and the residual solid was re-suspended in H_2_O (50 mL). The precipitate was filtered, washed with ice-water (2 × 50 mL), suction-dried and re-dissolved in EtOAc (50 mL). The organic solution was washed with H_2_O (2 × 50 mL), brine (2 × 50 mL), dried over anhydrous Na_2_SO_4_, filtered and evaporated to dryness. The crude residue was purified by flash column chromatography (SiO_2_, PE/EtOAc; 1:1) to afford acetophenone **51** (389 mg, 25%) as a white powdery solid. m.p. 236–238 °C. TLC *R*_f_ = 0.32 (PE/EtOAc; 1:1). IR ν_max_ (neat)/cm^−1^: 3422m (br) (O-H str), 3372s(br) (O-H str), 3068w (C-H str), 2962w (C-H str), 1640m (C=O str), 1598s (C=C str), 1521m (C=C str), 1456s, 1376s, 1331w, 1279m, 1213s, 1164s, 1073s, 1017m. ^1^H-NMR (500 MHz, DMSO-*d*_6_): δ 4.98 (2H, s, -COCH_2_-), 5.84 (2H, s, ArH), 10.55 (1H, s, OH), 12.07 (2H, s, OH). ^13^C-NMR (500 MHz, DMSO-*d*_6_): δ 51.0, 94.7, 102.5, 163.9, 165.5, 194.7. LCMS (ES+) *m*/*z* = 203.0 ([M + H]^+^, *t*_R_ = 1.26 min). These characterisation data are in accordance with that previously reported in the literature [26].

*Dihydroxybenzofuran-3(2H)-one* (**52**). To a stirred solution of acetophenone **51** (5.00 g, 24.7 mmol) in MeOH (100 mL) was added NaOMe (4.88 g, 90.3 mmol) and the mixture was heated at reflux for 2 h under nitrogen. The reaction mixture was allowed to cool to room temperature, acidified with 1 M HCl and the solvent removed under reduced pressure. The resulting dark residue was then re-dissolved in EtOAc (100 mL). The organic solution was washed with H_2_O (2 × 100 mL), brine (2 × 100 mL), dried over anhydrous MgSO_4_, filtered and evaporated to dryness. The crude residue was purified by flash column chromatography (SiO_2_, PE/EtOAc; 1:1) to afford benzofuranone **52** (2.90 g, 71%) as a pale brown-white powdery solid. m.p. 280–282 °C. TLC *R*_f_ = 0.16 (PE/EtOAc; 1:1). IR ν_max_ (neat)/cm^−1^: 3331s(br) (O-H str), 3164m(br) (O-H str), 3062w (C-H str), 1671m (C=O str), 1607s (C=C str), 1533w (C=C str), 1457m, 1422w, 1399m, 1369m, 1336m, 1261w, 1227m, 1157s, 1064s, 1042m, 1012m. ^1^H-NMR (500 MHz, DMSO-*d*_6_): δ 4.55 (2H, s, -OCH_2_CO-), 5.91 (2H, s, ArH), 10.59 (2H, br s, OH). ^13^C-NMR (500 MHz, DMSO-*d*_6_): δ 74.9, 90.1, 96.2, 102.7, 157.5, 167.6, 175.6, 194.0. LCMS (ES+) *m*/*z* = 167.1 ([M + H]^+^, *t*_R_ = 1.27 min). These characterisation data are in accordance with that previously reported in the literature [26].

*4,6-Dimethoxybenzofuran-3(2H)-one* (**53**). To a stirred solution of dihydroxybenzofuranone **52** (2.02 g, 12.2 mmol) in dry DMF (50 mL) were added CH_3_I (2.30 mL, 37.0 mmol) and anhydrous K_2_CO_3_ (3.35 g, 24.2 mmol). The resulting dark red-brown suspension was heated at 80 °C for 1 h under a nitrogen atmosphere. The reaction mixture was then allowed to cool to room temperature, poured into ice-water (100 mL) and extracted with EtOAc (3 × 100 mL). The combined organic layer was washed with H_2_O (3 × 100 mL), brine (2 × 100 mL), dried over anhydrous MgSO_4_, filtered and evaporated to dryness. The crude residue was purified by flash column chromatography (SiO_2_, PE/EtOAc; 1:1) to afford benzofuranone **53** (1.87 g, 79%) as a pale yellow-white powdery solid. m.p. 148–150 °C. TLC *R*_f_ = 0.23 (PE/EtOAc; 1:1). IR ν_max_ (neat)/cm^−1^: 2979w (C-H str), 2949w (C-H str), 1699s (C=O str), 1616s (C=C str), 1585s (C=C str), 1500m (C=C str), 1463m, 1431m, 1366m, 1342m, 1288m, 1217s, 1186s, 1160s, 1099s, 1052m, 1021m. ^1^H-NMR (500 MHz, CDCl_3_): δ 3.83 (3H, s, -OCH_3_), 3.87 (3H, s, -OCH_3_), 4.55 (2H, s, -OCH_2_CO-), 5.97 (1H, d, *J* = 1.6 Hz, ArH), 6.11 (1H, d, *J* = 2.0 Hz, ArH). ^13^C-NMR (500 MHz, CDCl_3_): δ 55.9, 55.9, 75.4, 88.8, 92.9, 104.7, 158.7, 169.7, 177.0, 194.9. LCMS (ES+) *m*/*z* = 195.1 ([M + H]^+^, *t*_R_ = 1.52 min). These characterisation data are in accordance with that previously reported in the literature [26].

*4,6-Dimethoxy-3′-hydroxyaurone* (**55**). To a stirred solution of benzofuranone **53** (1.01 g, 5.20 mmol) in MeOH (20 mL) was added 3-hydroxybenzaldehyde **54** (0.760 g, 6.22 mmol) followed by the addition of KOH (1.50 g, 26.6 mmol) in H_2_O (20 mL). The reaction mixture was stirred at room temperature for 2 h and then poured into H_2_O (2 × 100 mL). The resulting suspension was neutralized to pH 7 with 3 M HCl and extracted with CHCl_3_ (3 × 50 mL). The combined organic layer was washed with H_2_O (3 × 100 mL), brine (100 mL), dried over anhydrous MgSO_4_, filtered and evaporated to dryness under reduced pressure. The crude residue was purified by flash column chromatography (SiO_2_, PE/EtOAc; 1:1) and recrystallized from MeOH to afford aurone **55** (1.21 g, 78%) as a bright yellow powdery solid. m.p. 178–180 °C. TLC *R*_f_ = 0.35 (PE/EtOAc; 1:2). IR ν_max_ (neat)/cm^−1^: 3254m(br) (O-H str), 2944w (C-H str), 2842w (C-H str), 1688m (C=O str), 1649m, 1612s (C=C str), 1586s (C=C str), 1501m (C=C str), 1447s, 1430w, 1338m, 1303m, 1249m, 1214s, 1153s, 1138m, 1087s, 1038w. ^1^H-NMR (500 MHz, (CD_3_)_2_CO): δ 3.94 (3H, s, -OCH_3_), 3.98 (3H, s, -OCH_3_), 6.32 (1H, d, *J* = 2.0 Hz, ArH), 6.56 (1H, d, *J* = 1.6 Hz, ArH), 6.56 (1H, s, -C=CH), 6.91 (1H, ddd, *J* = 8.0, 2.4, 0.8 Hz, ArH), 7.30 (1H, t, *J* = 8.0 Hz, ArH), 7.40 (1H, d, *J* = 7.6 Hz, ArH), 7.47 (1H, t, *J* = 2.0 Hz, ArH), 8.57 (1H, s, OH). ^13^C-NMR (500 MHz, (CD_3_)_2_CO): δ 55.9, 56.2, 89.7, 94.4, 104.8, 109.4, 116.8, 117.5, 122.8, 130.0, 134.1, 148.1, 157.9, 159.7, 169.2, 169.5, 179.4. LCMS (ES+) *m*/*z* = 299.2 ([M + H]^+^, *t*_R_ = 1.71 min). These characterisation data are in accordance with that previously reported in the literature [48].

*4,6-Dimethoxy-3′-(prop-2-yn-1-yloxy)aurone* (**56**). A mixture of aurone **55** (505 mg, 1.69 mmol), propargyl bromide (0.30 mL, 3.37 mmol) and anhydrous K_2_CO_3_ (701 mg, 5.07 mmol) in dry acetone (30 mL) was reacted according to GP-C. The crude residue was purified by flash column chromatography (SiO_2_, 0.5% MeOH/CH_2_Cl_2_) to afford aurone **56** (545 mg, 96%) as a pale yellow-white powdery solid. m.p. 152–154 °C. TLC *R*_f_ = 0.46 (1% MeOH/CH_2_Cl_2_). IR ν_max_ (neat)/cm^−1^: 3219m (C≡C-H str), 3007w (C-H str), 2940w (C-H str), 2110w (C≡C str), 1686s (C=O str), 1654m, 1612s (C=C str), 1589s (C=C str), 1504s (C=C str), 1454m, 1423m, 1347m, 1313m, 1266m, 1215s, 1154m, 1094s, 1040m, 1019w. ^1^H-NMR (500 MHz, CDCl_3_): δ 2.57 (1H, t, *J* = 2.5 Hz, -OCH_2_C≡C*H*), 3.91 (3H, s, -OCH_3_), 3.95 (3H, s, -OCH_3_), 4.75 (2H, d, *J* = 2.5 Hz, -OC*H*_2_C≡CH), 6.12 (1H, d, *J* = 2.0 Hz, ArH), 6.37 (1H, d, *J* = 2.0 Hz, ArH), 6.72 (1H, s, -C=CH), 6.99 (1H, ddd, *J* = 8.0, 2.5, 1.0 Hz, ArH), 7.35 (1H, t, *J* = 8.0 Hz, ArH), 7.46 (1H, d, *J* = 7.5 Hz, ArH), 7.54 (1H, t, *J* = 1.5 Hz, ArH). ^13^C-NMR (500 MHz, CDCl_3_): δ 55.9, 56.1, 56.2, 75.7, 78.3, 89.2, 94.0, 105.1, 110.3, 115.9, 117.1, 124.6, 129.7, 133.9, 148.0, 157.6, 159.4, 169.0, 180.6. LCMS (ES+) *m*/*z* = 337.2 ([M + H]^+^, *t*_R_ = 1.83 min). HRMS (ESI+) *m*/*z* = 337.1062 [M + H]^+^ found, C_20_H_17_O_5_^+^ required 337.1071.

*4-(2-Bromoethoxy)-2H-chromen-2-one* (**57**). A mixture of 4-hydroxycoumarin **47** (5.14 g, 31.7 mmol), 1,2-dibromoethane (3.19 mL, 37.0 mmol) and anhydrous K_2_CO_3_ (8.56 g, 61.9 mmol) in dry acetone (100 mL) was reacted according to GP-I. The crude residue was purified by flash column chromatography (SiO_2_, CH_2_Cl_2_) to afford coumarin **57** (1.33g, 16%) as a white powdery solid. m.p. 176–178 °C. TLC *R*_f_ = 0.41 (PE/EtOAc 1:1). IR ν_max_ (neat)/cm^−1^: 3044w (C-H str), 2982w (C-H str), 1716s (C=O str), 1627s, 1607s, 1566m (C=C str), 1496m, 1454m, 1407s, 1369s, 1330m, 1272m, 1246s, 1225s, 1184s, 1147m, 1111s, 1067w, 1034s. ^1^H-NMR (500 MHz, CDCl_3_): δ 3.77 (2H, t, *J* = 6.0 Hz, -OCH_2_C*H*_2_Br), 4.46 (2H, t, *J* = 6.0 Hz, -OC*H*_2_CH_2_Br), 5.68 (1H, s, -C=CH), 7.31 (1H, t, *J* = 8.0 Hz, ArH), 7.34 (1H, dd, *J* = 8.5, 0.5 Hz, ArH), 7.58 (1H, t, *J* = 8.5 Hz, ArH), 7.88 (1H, dd, *J* = 7.5, 1.5 Hz, ArH). ^13^C-NMR (500 MHz, CDCl_3_): δ 27.6, 68.4, 90.9, 115.3, 116.8, 123.1, 124.0, 132.7, 153.3, 162.5, 164.9. LCMS (ES+) *m*/*z* = 271.0 ([M + H]^+^, *t*_R_ = 1.53 min). These characterisation data are in accordance with that previously reported in the literature [49].

*4-(2-Azidoethoxy)-2H-chromen-2-one* (**58**). A mixture of coumarin **57** (679 mg, 2.52 mmol) and NaN_3_ (370 mg, 5.69 mmol) in dry DMF (20 mL) was reacted according to GP-J. The reaction mixture was worked up to afford coumarin **58** (571 mg, 98%) as an off-white powdery solid and was used without further purification. m.p. 150–152 °C. TLC *R*_f_ = 0.40 (1% MeOH/CH_2_Cl_2_). IR ν_max_ (neat)/cm^−1^: 3075w (C-H str), 2947w (C-H str), 2125s (N_3_ str), 1732s (C=O str), 1623s, 1609s, 1566s (C=C str), 1495s, 1453m, 1417s, 1371s, 1277m, 1236s, 1180s, 1147s, 1109s, 1032s. ^1^H-NMR (500 MHz, CDCl_3_): δ 3.75 (2H, t, *J* = 5.0 Hz, -OCH_2_C*H*_2_N_3_), 4.32 (2H, t, *J* = 5.0 Hz, -OC*H*_2_CH_2_N_3_), 5.70 (1H, s, -C=CH), 7.31 (1H, t, *J* = 8.0 Hz, ArH), 7.34 (1H, dd, *J* = 8.5, 0.5 Hz, ArH), 7.58 (1H, t, *J* = 8.5 Hz, ArH), 7.84 (1H, dd, *J* = 8.0, 1.5 Hz, ArH). ^13^C-NMR (500 MHz, CDCl_3_): δ 49.6, 68.2, 90.9, 115.2, 116.8, 123.0, 124.1, 132.7, 153.3, 162.5, 165.1. LCMS (ES+) *m*/*z* = 232.1 ([M + H]^+^, *t*_R_ = 1.70 min). These characterisation data are in accordance with that previously reported in the literature [27].

*4-(2-Bomoethoxy)benzaldehyde* (**59**). A mixture of benzaldehyde **33** (20.0 g, 164 mmol), 1,2-dibromoethane (28.5 mL, 331 mmol) and anhydrous K_2_CO_3_ (46.0 g, 333 mmol) in dry acetone (100 mL) was reacted according to GP-I. The crude residue was purified by flash column chromatography (SiO_2_, CH_2_Cl_2_) to afford benzaldehyde **59** (13.2 g, 35%) as a white powdery solid. m.p. 56–58 °C. TLC *R*_f_ = 0.41 (CH_2_Cl_2_). IR ν_max_ (neat)/cm^−1^: 2967w (C-H str), 1679s (C=O str), 1601s (C=C str), 1577s (C=C str), 1508m (C=C str), 1458m, 1422m, 1392m, 1317w, 1300m, 1282m, 1249s, 1229s, 1211s, 1160s, 1107w, 1068s, 1008s. ^1^H-NMR (500 MHz, CDCl_3_): δ 3.68 (2H, t, *J* = 6.4 Hz, -OCH_2_C*H*_2_Br), 4.39 (2H, t, *J* = 6.4 Hz, -OC*H*_2_CH_2_Br), 7.03 (2H, d, *J* = 8.8 Hz, ArH), 7.86 (2H, d, *J* = 8.8 Hz, ArH), 9.91 (1H, s, CHO). ^13^C-NMR (500 MHz, CDCl_3_): δ 28.4, 67.9, 114.8, 130.5, 132.0, 163.0, 190.7. LCMS (ES+) *m*/*z* = 231.0 ([M + H]^+^, *t*_R_ = 1.56 min). These characterisation data are in accordance with that previously reported in the literature [50].

*4-(2-Azidoethoxy)benzaldehyde* (**60**). A mixture of benzaldehyde **59** (12.8 g, 56.0 mmol) and NaN_3_ (7.36 g, 113 mmol) in dry DMF (100 mL) was reacted according to GP-J. The reaction mixture was worked up to afford benzaldehyde **60** (10.6 g, 99%) as a pale yellow viscous oil and was used without further purification. TLC *R*_f_ = 0.38 (CH_2_Cl_2_). IR ν_max_ (neat)/cm^−1^: 2942w (C-H str), 2837w (C-H str), 2100s (N_3_ str), 1682s (C=O str), 1598s (C=C str), 1578s (C=C str), 1508s (C=C str), 1427w, 1395w, 1304m, 1247s, 1213s, 1157s, 1110m, 1052m, 1008w. ^1^H-NMR (500 MHz, CDCl_3_): δ 3.64 (2H, t, *J* = 5.0 Hz, -OCH_2_C*H*_2_N_3_), 4.22 (2H, t, *J* = 5.0 Hz, -OC*H*_2_CH_2_N_3_), 7.02 (2H, d, *J* = 8.5 Hz, ArH), 7.84 (2H, d, *J* = 9.0 Hz, ArH), 9.88 (1H, s, CHO). ^13^C-NMR (500 MHz, CDCl_3_): δ 49.9, 67.1, 114.7, 130.3, 131.9, 163.0, 190.7. LCMS (ES+) *m*/*z* = 193.2 ([M + H]^+^, *t*_R_ = 1.62 min). These characterisation data are in accordance with that previously reported in the literature [50].

*(E)-3-(4-(2-Azidoethoxy)phenyl)-1-(2-hydroxyphenyl)prop-2-en-1-one* (**61**). A mixture of benzaldehyde **60** (4.90 g, 25.6 mmol), acetophenone **10** (3.09 mL, 25.7 mmol) and KOH (8.70 g, 155 mmol) in absolute EtOH (100 mL) was reacted according to GP-D. The crude residue was purified by recrystallization from MeOH to afford chalcone **61** (6.13 g, 77%) as a bright yellow powdery solid. m.p. 142–144 °C. TLC *R*_f_ = 0.48 (PE/EtOAc; 2:1). IR ν_max_ (neat)/cm^−1^: 2932w (C-H str), 2874w (C-H str), 2107m (N_3_ str), 2070m, 1636s (C=O str), 1602s, 1575s (C=C str), 1560s (C=C str), 1508s (C=C str), 1488s, 1424m, 1345m, 1299m, 1271m, 1244m, 1201m, 1059m, 1031m. ^1^H-NMR (500 MHz, CDCl_3_): δ 3.63 (2H, t, *J* = 5.0 Hz, -OCH_2_C*H*_2_N_3_), 4.19 (2H, t, *J* = 5.0 Hz, -OC*H*_2_CH_2_N_3_), 6.93–6.98 (3H, m, ArH), 7.03 (1H, dd, *J* = 8.5, 1.0 Hz, ArH), 7.49 (1H, t, *J* = 8.5 Hz, ArH), 7.54 (1H, d, *J* = 15.5 Hz, -C*H*=CHCO-), 7.63 (2H, d, *J* = 8.5 Hz, ArH), 7.89 (1H, d, *J* = 15.5 Hz, -CH=C*H*CO-), 7.92 (1H, dd, *J* = 8.5, 2.0 Hz, ArH), 12.94 (1H, s, OH). ^13^C-NMR (500 MHz, CDCl_3_): δ 50.0, 67.0, 115.0, 117.9, 118.5, 118.7, 120.0, 127.9, 129.5, 130.5, 136.2, 145.0, 160.5, 163.5, 193.5. LCMS (ES+) *m*/*z* = 310.1 ([M + H]^+^, *t*_R_ = 1.80 min). These characterisation data are in accordance with that previously reported in the literature [51].

*1-(4-(2-Bromoethoxy)-3-methoxyphenyl)ethan-1-one* (**62**). A mixture of acetophenone **11** (10.0 g, 60.3 mmol), 1,2-dibromoethane (10.5 mL, 122 mmol) and anhydrous K_2_CO_3_ (12.6, 91.5 mmol) in dry DMF (100 mL) was reacted according to GP-I. The crude residue was purified by flash column chromatography (SiO_2_, CH_2_Cl_2_) to afford acetophenone **62** (3.90 g, 24%) as a white powdery solid. m.p. 98–100 °C. TLC *R*_f_ = 0.16 (CH_2_Cl_2_). IR ν_max_ (neat)/cm^−1^: 3075w (C-H str), 2971w (C-H str), 1760w, 1671s (C=O str), 1585s (C=C str), 1507s (C=C str), 1460m, 1412s, 1385w, 1359m, 1263s, 1220s, 1171m, 1144s, 1076s, 1030s, 1008s. ^1^H-NMR (500 MHz, CDCl_3_): δ 2.58 (3H, s, -COCH_3_), 3.70 (2H, t, *J* = 6.8 Hz, -OCH_2_C*H*_2_Br), 3.94 (3H, s, -OCH_3_), 4.41 (2H, t, *J* = 6.8 Hz, -OC*H*_2_CH_2_Br), 6.91 (1H, d, *J* = 8.8 Hz, ArH), 7.55–7.57 (2H, m, ArH). ^13^C-NMR (500 MHz, CDCl_3_): δ 26.3, 28.2, 56.1, 68.7, 111.0, 112.2, 123.0, 131.4, 149.5, 151.7, 196.7. LCMS (ES+) *m*/*z* = 275.0 ([M + H]^+^, *t*_R_ = 1.43 min). These characterisation data are in accordance with that previously reported in the literature [52].

*1-(4-(2-Azidoethoxy)-3-methoxyphenyl)ethan-1-one* (**63**). A mixture of acetophenone **62** (3.50 g, 12.8 mmol) and NaN_3_ (1.25 g, 19.2 mol) in DMF (30 mL) was reacted according to GP-J. The reaction mixture was worked up to afford phenylethanone **63** (2.94 g, 98%) as a pale brown residual oil which solidified upon standing to give a pale brown crystalline solid and used without further purification. m.p. 58–60 °C. TLC *R*_f_ = 0.30 (0.5% MeOH/CH_2_Cl_2_). IR ν_max_ (neat)/cm^−1^: 3088w (C-H str), 2956w (C-H str), 2110s (N_3_ str), 2067m, 1742w, 1671s (C=O str), 1589s (C=C str), 1508s (C=C str), 1471m, 1419s, 1355m, 1270s, 1216s, 1177m, 1151s, 1080m, 1036s. ^1^H-NMR (500 MHz, CDCl_3_): δ 2.56 (3H, s, -COC*H*_3_), 3.68 (2H, t, *J* = 5.2 Hz, -OCH_2_C*H*_2_N_3_), 3.91 (3H, s, -OCH_3_), 4.23 (2H, t, *J* = 5.2 Hz, -OC*H*_2_CH_2_N_3_), 6.89 (1H, d, *J* = 8.0 Hz, ArH), 7.53–7.55 (2H, m, ArH). ^13^C-NMR (500 MHz, CDCl_3_): δ 26.2, 50.0, 56.0, 67.7, 110.7, 111.9, 122.9, 131.2, 149.4, 151.9, 196.7. LCMS (ES+) *m*/*z* = 236.0 ([M + H]^+^, *t*_R_ = 1.45 min). These characterisation data are in accordance with that previously reported in the literature [52].

*(E)-1-(4-(2-Azidoethoxy)-3-methoxyphenyl)-3-(3,4-dimethoxyphenyl)prop-2-en-1-one* (**67**). A mixture of benzaldehyde **64** (1.48 g, 8.91 mmol), acetophenone **63** (2.04 g, 8.67 mmol) and KOH (2.41 g, 43.0 mmol) in absolute EtOH (100 mL) was reacted according to GP-D. The crude residue was purified by flash column chromatography (SiO_2_, PE/EtOAc; 5:1) and recrystallized from MeOH to afford chalcone **67** (2.61 g, 79%) as a pale yellow-green powdery solid. m.p. 106–108 °C. TLC *R*_f_ = 0.28 (PE/EtOAc; 1:1). IR ν_max_ (neat)/cm^−1^: 2940w (C-H str), 2838w (C-H str), 2112s (N_3_ str), 2068m, 1648s (C=O str), 1595s (C=C str), 1568s (C=C str), 1509s (C=C str), 1458m, 1419m, 1355w, 1312m, 1261s, 1242s, 1199m, 1159m, 1139s, 1036s, 1021s. ^1^H-NMR (500 MHz, CDCl_3_): δ 3.67 (2H, t, *J* = 5.2 Hz, -OCH_2_C*H*_2_N_3_), 3.91 (3H, s, -OCH_3_), 3.93 (3H, s, -OCH_3_), 3.94 (3H, s, -OCH_3_), 4.23 (2H, t, *J* = 5.2 Hz, -OC*H*_2_CH_2_N_3_), 6.88 (1H, d, *J* = 8.4 Hz, ArH), 6.92 (1H, d, *J* = 8.0 Hz, ArH), 7.15 (1H, d, *J* = 2.0 Hz, ArH), 7.22 (1H, dd, *J* = 8.4, 2.0 Hz, ArH), 7.39 (1H, d, *J* = 15.6 Hz, -C*H*=CHCO-), 7.61–7.65 (2H, m, ArH), 7.75 (1H, d, *J* = 15.2 Hz, -CH=C*H*CO-). ^13^C-NMR (500 MHz, CDCl_3_): δ 49.9, 55.8, 56.0, 67.7, 110.1, 111.0, 111.4, 111.9, 119.4, 122.4, 122.8, 127.8, 132.2, 144.2, 149.1, 149.6, 151.2, 151.7, 188.5. LCMS (ES+) *m*/*z* = 384.2 ([M + H]^+^, *t*_R_ = 3.98 min). These characterisation data are in accordance with that previously reported in the literature [52].

*(E)-1-(4-(2-Azidoethoxy)-3-methoxyphenyl)-3-(2,3,4-trimethoxyphenyl)prop-2-en-1-one* (**68**). A mixture of benzaldehyde **65** (1.70 g, 8.66 mmol), acetophenone **63** (2.02 g, 8.59 mmol) and KOH (2.39 g, 42.6 mmol) in absolute EtOH (100 mL) was reacted according to GP-D. The crude residue was purified by flash column chromatography (SiO_2_, PE/EtOAc; 5:1) and recrystallized from MeOH to afford chalcone **68** (2.61 g, 74%) as a pale yellow-green powdery solid. m.p. 114–116 °C. TLC *R*_f_ = 0.35 (PE/EtOAc; 1:1). IR ν_max_ (neat)/cm^−1^: 2945w (C-H str), 2836w (C-H str), 2119s (N_3_ str), 2072m, 1743w, 1644m (C=O str), 1588m (C=C str), 1560s (C=C str), 1519m (C=C str), 1494s, 1466m, 1420s, 1246s, 1149s, 1093s, 1042s. ^1^H-NMR (500 MHz, CDCl_3_): δ 3.66 (2H, t, *J* = 5.2 Hz, -OCH_2_C*H*_2_N_3_), 3.87 (3H, s, -OCH_3_), 3.88 (3H, s, -OCH_3_), 3.92 (3H, s, -OCH_3_), 3.93 (3H, s, -OCH_3_), 4.23 (2H, t, *J* = 5.0 Hz, -OC*H*_2_CH_2_N_3_), 6.70 (1H, d, *J* = 8.8 Hz, ArH), 6.91 (1H, d, *J* = 8.8 Hz, ArH), 7.37 (1H, d, *J* = 8.8 Hz, ArH), 7.55 (1H, d, *J* = 16.0 Hz, -C*H*=CHCO-), 7.61–7.63 (2H, m, ArH), 7.97 (1H, d, *J* = 15.6 Hz, -CH=C*H*CO-). ^13^C-NMR (500 MHz, CDCl_3_): δ 49.9, 55.9, 60.8, 61.2, 67.7, 107.5, 111.5, 112.0, 120.8, 121.9, 122.4, 123.7, 132.4, 139.3, 142.3, 149.6, 151.6, 153.6, 155.6, 188.9. LCMS (ES+) *m*/*z* = 414.1 ([M + H]^+^, *t*_R_ = 1.64 min). HRMS (ESI+) *m*/*z* = 414.1653 [M + H]^+^ found, C_21_H_24_O_6_N_3_^+^ required 414.1660.

*(E)-1-(4-(2-Azidoethoxy)-3-methoxyphenyl)-3-(2,4,6-trimethoxyphenyl)prop-2-en-1-one* (**69**). A mixture of benzaldehyde **66** (1.69 g, 8.61 mmol), acetophenone **63** (2.06 g, 8.76 mmol) and KOH (2.44 g, 43.5 mmol) in absolute EtOH (100 mL) was reacted according to GP-D. The crude residue was purified by recrystallization from MeOH to afford chalcone **69** (2.91 g, 82%) as a pale yellow-green powdery solid. m.p. 132–134 °C. TLC *R*_f_ = 0.29 (PE/EtOAc; 1:1). IR ν_max_ (neat)/cm^−1^: 3005w (C-H str), 2939w (C-H str), 2840w (C-H str), 2112m (N_3_ str), 1647m (C=O str), 1596m (C=C str), 1567s (C=C str), 1518m (C=C str), 1493w, 1451m, 1417m, 1320s, 1266m, 1213s, 1147s, 1119s, 1026s. ^1^H-NMR (500 MHz, CDCl_3_): δ 3.70 (2H, t, *J* = 5.2 Hz, -OCH_2_C*H*_2_N_3_), 3.87 (3H, s, -OCH_3_), 3.92 (6H, s, 2 × -OCH_3_), 3.96 (3H, s, -OCH_3_), 4.27 (2H, t, *J* = 5.2 Hz, -OC*H*_2_CH_2_N_3_), 6.16 (2H, s, ArH), 6.95 (1H, d, *J* = 8.4 Hz, ArH), 7.62–7.65 (2H, m, ArH), 7.88 (1H, d, *J* = 16.0 Hz, -C*H*=CHCO-), 8.24 (1H, d, *J* = 16.0 Hz, -CH=C*H*CO-). ^13^C-NMR (500 MHz, CDCl_3_): δ 50.1, 55.4, 55.8, 56.1, 67.8, 90.5, 106.6, 111.8, 112.2, 121.8, 122.4, 133.3, 135.4, 149.6, 151.2, 161.6, 163.0, 190.5. LCMS (ES+) *m*/*z* = 414.1 ([M + H]^+^, *t*_R_ = 1.67 min). HRMS (ESI+) *m*/*z* = 414.1654 [M + H]^+^ found, C_21_H_24_O_6_N_3_^+^ required 414.1660.

*(E)-1-(4-(2-Azidoethoxy)-3-methoxyphenyl)-3-(1-methyl-1H-pyrrol-2-yl)prop-2-en-1-one* (**70**). A mixture of pyrrole aldehyde **7** (1.40 mL, 13.0 mmol), acetophenone **63** (3.03 g, 12.9 mmol) and KOH (4.33 g, 77.2 mmol) in absolute EtOH (100 mL) was reacted according to GP-D. The crude product was purified by flash column chromatography (SiO_2_, PE/EtOAc; 5:1) and recrystallized from MeOH to afford chalcone **70** as a bright yellow powdery solid (1.56 g, 37%). m.p. 124–126 °C. TLC *R*_f_ = 0.32 (PE/EtOAc; 1:1). IR ν_max_ (neat)/cm^−1^: 2946w (C-H str), 2107m (N_3_ str), 2064w, 1739w, 1644m (C=O str), 1595s (C=C str), 1562s (C=C str), 1515m (C=C str), 1485m, 1342s, 1272s, 1238s, 1196s, 1174s, 1125s, 1059m, 1034s. ^1^H-NMR (500 MHz, CDCl_3_): δ 3.70 (2H, t, *J* = 5.2 Hz, -OCH_2_C*H*_2_N_3_), 3.78 (3H, s, -NCH_3_), 3.96 (3H, s, -OCH_3_), 4.27 (2H, t, *J* = 4.8 Hz, -OC*H*_2_CH_2_N_3_), 6.23–6.24 (1H, m, ArH), 6.82 (1H, t, *J* = 1.6 Hz, ArH), 6.85 (1H, dd, *J* = 4.0, 1.6 Hz, ArH), 6.94 (1H, d, *J* = 8.8 Hz, ArH), 7.31 (1H, d, *J* = 14.8 Hz, -C*H*=CHCO-), 7.62–7.64 (2H, m, ArH), 7.81 (1H, d, *J* = 15.2 Hz, -CH=C*H*CO-). ^13^C-NMR (500 MHz, CDCl_3_): δ 34.4, 50.1, 56.1, 67.8, 109.7, 111.5, 112.1, 112.1, 116.4, 122.2, 127.6, 130.3, 131.7, 132.7, 149.7, 151.6, 188.1. LCMS (ES+) *m*/*z* = 327.2 ([M + H]^+^, *t*_R_ = 1.82 min). HRMS (ESI+) *m*/*z* = 327.1447 [M + H]^+^ found, C_17_H_19_O_3_N_4_^+^ required 327.1452.

*4-(2-Bromoethoxy)-3-methoxy benzaldehyde* (**72**). A mixture of benzaldehyde **71** (10.0 g, 65.9 mmol), 1,2-dibromoethane (7.40 mL, 85.9 mmol) and anhydrous K_2_CO_3_ (11.8 g, 85.5 mmol) in dry acetone (100 mL) was reacted according to GP-I. The crude residue was purified by flash column chromatography (SiO_2_, CH_2_Cl_2_) to afford benzaldehyde **72** (4.24 g, 25%) as a white crystalline solid. m.p. 84–86 °C. TLC *R*_f_ = 0.37 (CH_2_Cl_2_). IR ν_max_ (neat)/cm^−1^: 2967w (C-H str), 2849w (C-H str), 1697m (C=O str), 1682s, 1672s, 1585s (C=C str), 1508s (C=C str), 1459m, 1444m, 1427m, 1397m, 1349w, 1279m, 1266s, 1233s, 1215w, 1159w, 1134s, 1015s, 1003m. ^1^H-NMR (500 MHz, CDCl_3_): δ 3.70 (2H, t, *J* = 6.5 Hz, -OCH_2_C*H*_2_Br), 3.94 (3H, s, -OCH_3_), 4.42 (2H, t, *J* = 6.5 Hz, -OC*H*_2_CH_2_Br), 6.99 (1H, d, *J* = 8.0 Hz, ArH), 7.43–7.46 (2H, m, ArH), 9.87 (1H, s, CHO). ^13^C-NMR (500 MHz, CDCl_3_): δ 28.1, 56.1, 68.7, 109.8, 112.4, 126.4, 130.8, 150.0, 152.9, 190.8. LCMS (ES+) *m*/*z* = 261.0 ([M + H]^+^, *t*_R_ = 1.49 min). These characterisation data are in accordance with that previously reported in the literature [53].

*3-(2-Bromoethoxy)-4-methoxy benzaldehyde* (**73**). A mixture of isovanillin **1** (10.3 g, 68.0 mmol), 1,2-dibromoethane (7.36 mL, 85.4 mmol) and anhydrous K_2_CO_3_ (11.8 g, 85.7 mmol) in dry acetone (100 mL) was reacted according to GP-I. The crude residue was purified by flash column chromatography (SiO_2_, CH_2_Cl_2_) to afford benzaldehyde **73** (3.81 g, 22%) as a white powdery solid. m.p. 88–90 °C. TLC *R*_f_ = 0.38 (CH_2_Cl_2_). IR *ν*_max_ (neat)/cm^−1^: 2978w (C-H str), 2840w (C-H str), 1677s (C=O str), 1595m (C=C str), 1582s (C=C str), 1509s (C=C str), 1462w, 1434s, 1392m, 1259s, 1238s, 1162s, 1131s, 1072w, 1013s. ^1^H-NMR (500 MHz, CDCl_3_): δ 3.69 (2H, t, *J* = 6.4 Hz, -OCH_2_C*H*_2_Br), 3.96 (3H, s, -OCH_3_), 4.39 (2H, t, *J* = 6.4 Hz, -OC*H*_2_CH_2_Br), 7.00 (1H, d, *J* = 8.0 Hz, ArH), 7.41 (1H, d, *J* = 1.6 Hz, ArH), 7.50 (1H, dd, *J* = 8.0, 1.6 Hz, ArH), 9.84 (1H, s, CHO). ^13^C-NMR (500 MHz, CDCl_3_): δ 28.5, 56.2, 68.8, 111.1, 111.6, 127.4, 130.0, 148.0, 155.0, 190.6. LCMS (ES+) *m*/*z* = 261.0 ([M + H]^+^, *t*_R_ = 1.47 min). These characterisation data are in accordance with that previously reported in the literature [53].

*4-(2-Azidoethoxy)-3-methoxybenzaldehyde* (**74**). A mixture of benzaldehyde **72** (3.40 g, 13.1 mmol) and NaN_3_ (1.74 g, 26.8 mmol) in dry DMF (20 mL) was reacted according to GP-J. The reaction mixture was worked up to afford benzaldehyde **74** (2.84 g, 98%) as a pale yellow viscous oil and was used without further purification. TLC *R*_f_ = 0.21 (CH_2_Cl_2_). IR ν_max_ (neat)/cm^−1^: 2938w (C-H str), 2831w (C-H str), 2106s (N_3_ str), 1679s (C=O str), 1586s (C=C str), 1507s (C=C str), 1457m, 1424m, 1396w, 1339w, 1263s, 1236m, 1194w, 1158w, 1134s, 1051w, 1030m. ^1^H-NMR (500 MHz, CDCl_3_): δ 3.66 (2H, t, *J* = 5.2 Hz, -OCH_2_C*H*_2_N_3_), 3.88 (3H, s, -OCH_3_), 4.22 (2H, t, *J* = 5.2 Hz, -OC*H*_2_CH_2_N_3_), 6.95 (1H, d, *J* = 8.0 Hz, ArH), 7.38–7.41 (2H, m, ArH), 9.81 (1H, s, CHO). ^13^C-NMR (500 MHz, CDCl_3_): δ 49.8, 55.8, 67.7, 109.5, 112.0, 126.1, 130.6, 149.9, 153.0, 190.7. LCMS (ES+) *m*/*z* = 222.0 ([M + H]^+^, *t*_R_ = 1.66 min). HRMS (ESI+) *m*/*z* = 222.0863 [M + H]^+^ found, C_10_H_12_O_3_N_3_^+^ required 222.0834.

*3-(2-Azidoethoxy)-4-methoxybenzaldehyde* (**75**). A mixture of benzaldehyde **73** (3.63 g, 14.0 mmol) and NaN_3_ (1.80 g, 27.7 mmol) in dry DMF (20 mL) was reacted according to GP-J. The reaction mixture was worked up to afford benzaldehyde **75** (3.03 g, 98%) as a pale yellow viscous oil and was used without further purification. TLC *R*_f_ = 0.26 (CH_2_Cl_2_). IR ν_max_ (neat)/cm^−1^: 2940w (C-H str), 2838w (C-H str), 2094s (N_3_ str), 1682s (C=O str), 1596s (C=C str), 1583s (C=C str), 1508s (C=C str), 1435s, 1397w, 1339w, 1261s, 1236s, 1161m, 1134s, 1125s, 1051w, 1019s. ^1^H-NMR (500 MHz, CDCl_3_): δ 3.68 (2H, t, *J* = 5.2 Hz, -OCH_2_C*H*_2_N_3_), 3.96 (3H, s, -OCH_3_), 4.25 (2H, t, *J* = 5.2 Hz, -OC*H*_2_CH_2_N_3_), 7.00 (1H, d, *J* = 8.0 Hz, ArH), 7.42 (1H, d, *J* = 2.0 Hz, ArH), 7.50 (1H, dd, *J* = 8.4, 2.0 Hz, ArH), 9.85 (1H, s, CHO). ^13^C-NMR (500 MHz, CDCl_3_): δ 50.0, 56.1, 67.9, 110.9, 111.1, 127.5, 129.9, 148.3, 155.1, 190.7. LCMS (ES+) *m*/*z* = 222.3 ([M + H]^+^, *t*_R_ = 1.69 min). HRMS (ESI+) *m*/*z* = 244.0683 [M + Na]^+^ found, C_10_H_11_O_3_N_3_Na^+^ required 244.0693.

*(E)-3-(4-(2-Azidoethoxy)phenyl)-1-(2-hydroxy-4-methoxyphenyl)prop-2-en-1-one* (**76**). A mixture of benzaldehyde **60** (5.00 g, 26.2 mmol), acetophenone **3** (4.39 g, 26.4 mmol) and KOH (9.42 g, 168 mmol) in absolute EtOH (100 mL) was reacted according to GP-D. The crude residue was purified by recrystallization from MeOH to afford chalcone **76** (5.72 g, 64%) as a bright yellow-orange powdery solid. m.p. 124–126 °C. TLC *R*_f_ = 0.36 (PE/EtOAc; 2:1). IR ν_max_ (neat)/cm^−1^: 2940w (C-H str), 2873w (C-H str), 2109m (N_3_ str), 2073m, 1634m (C=O str), 1604m, 1573s (C=C str), 1513s (C=C str), 1445m, 1426m, 1361s, 1328m, 1307w, 1279s, 1250w, 1209s, 1184s, 1119s, 1057m, 1015s. ^1^H-NMR (500 MHz, CDCl_3_): δ 3.65 (2H, t, *J* = 5.2 Hz, -OCH_2_C*H*_2_N_3_), 3.87 (3H, s, -OCH_3_), 4.22 (2H, t, *J* = 5.2 Hz, -OC*H*_2_CH_2_N_3_), 6.49–6.51 (2H, m, ArH), 6.98 (2H, d, *J* = 8.8 Hz, ArH), 7.48 (1H, d, *J* = 15.2 Hz, -C*H*=CHCO-), 7.63 (2H, d, *J* = 8.8 Hz, ArH), 7.84 (1H, d, *J* = 9.2 Hz, ArH), 7.87 (1H, d, *J* = 15.6 Hz, -CH=C*H*CO-), 13.52 (1H, s, OH). ^13^C-NMR (500 MHz, CDCl_3_): δ 50.6, 55.6, 67.1, 101.0, 107.7, 114.1, 115.0, 118.3, 128.2, 130.4, 131.1, 144.0, 160.3, 166.1, 166.6, 191.8. LCMS (ES+) *m*/*z* = 340.2 ([M + H]^+^, *t*_R_ = 1.74 min). HRMS (ESI+) *m*/*z* = 340.1295 [M + H]^+^ found, C_18_H_18_N_3_O_4_^+^ required 340.1297.

*(E)-3-(4-(2-Azidoethoxy)-3-methoxyphenyl)-1-(2-hydroxyphenyl)prop-2-en-1-one* (**77**). A mixture of benzaldehyde **74** (2.70 g, 12.2 mmol), acetophenone **10** (1.50 mL, 12.5 mmol) and KOH (4.30 g, 76.6 mmol) in absolute EtOH (100 mL) was reacted according to GP-D. The crude residue was purified by flash column chromatography (SiO_2_, PE/EtOAc; 5:1) to afford chalcone **77** (1.91 g, 46%) as a bright yellow-orange powdery solid. m.p. 84–86 °C. TLC *R*_f_ = 0.25 (PE/EtOAc; 3:1). IR ν_max_ (neat)/cm^−1^: 2935w (C-H str), 2876w (C-H str), 2106s (N_3_ str), 1635s (C=O str), 1580m (C=C str), 1562s (C=C str), 1508s (C=C str), 1489s, 1462w, 1441w, 1421w, 1372w, 1313w, 1255s, 1201s, 1168w, 1138s, 1057w, 1028s. ^1^H-NMR (500 MHz, CDCl_3_): δ 3.68 (2H, t, *J* = 5.5 Hz, -OCH_2_C*H*_2_N_3_), 3.95 (3H, s, -OCH_3_), 4.24 (2H, t, *J* = 5.5 Hz, -OC*H*_2_CH_2_N_3_), 6.92 (1H, d, *J* = 8.0 Hz, ArH), 6.93–6.96 (1H, m, ArH), 7.03 (1H, dd, *J* = 8.5, 1.5 Hz, ArH), 7.19 (1H, d, *J* = 2.0 Hz, ArH), 7.25 (1H, dd, *J* = 8.5, 1.5 Hz, ArH), 7.50 (1H, t, *J* = 8.5 Hz, ArH), 7.53 (1H, d, *J* = 15.5 Hz, -C*H*=CHCO-), 7.87 (1H, d, *J* = 15.5 Hz, -CH=C*H*CO-), 7.93 (1H, dd, *J* = 8.0, 1.5 Hz, ArH), 12.91 (1H, s, OH). ^13^C-NMR (500 MHz, CDCl_3_): δ 50.1, 56.1, 67.9, 111.2, 113.4, 118.2, 118.5, 118.7, 120.0, 123.1, 128.5, 129.5, 136.2, 145.4, 149.9, 150.5, 163.5, 193.5. LCMS (ES+) *m*/*z* = 340.3 ([M + H]^+^, *t*_R_ = 1.96 min). HRMS (ESI+) *m*/*z* = 340.1296 [M + H]^+^ found, C_18_H_18_N_3_O_4_^+^ required 340.1297.

*(E)-3-(3-(2-Azidoethoxy)-4-methoxyphenyl)-1-(2-hydroxyphenyl)prop-2-en-1-one* (**78**). A mixture of benzaldehyde **75** (3.00 g, 13.6 mmol), acetophenone **10** (1.63 mL, 13.6 mmol) and KOH (4.57 g, 81.4 mmol) in absolute EtOH (100 mL) was reacted according to GP-D. The crude residue was purified by flash column chromatography (SiO_2_, PE/EtOAc; 5:1) to afford chalcone **78** (2.72 g, 59%) as a bright yellow powdery solid. m.p. 98–100 °C. TLC *R*_f_ = 0.33 (PE/EtOAc; 3:1). IR ν_max_ (neat)/cm^−1^: 3009w (C-H str), 2970w (C-H str), 2112m (N_3_ str), 2067w, 1738s (C=O str), 1634s, 1567s (C=C str), 1511s (C=C str), 1490s, 1440m, 1374s, 1315m, 1266s, 1227s, 1204s, 1142s, 1024s. ^1^H-NMR (500 MHz, CDCl_3_): δ 3.66 (2H, t, *J* = 5.0 Hz, -OCH_2_C*H*_2_N_3_), 3.89 (3H, s, -OCH_3_), 4.22 (2H, t, *J* = 5.0 Hz, -OC*H*_2_CH_2_N_3_), 6.90 (1H, d, *J* = 8.5 Hz, ArH), 6.91 (1H, t, *J* = 8.5 Hz, ArH), 7.00 (1H, dd, *J* = 8.5, 1.0 Hz, ArH), 7.19 (1H, d, *J* = 2.5 Hz, ArH), 7.27 (1H, dd, *J* = 8.0, 2.0 Hz, ArH, overlain by CDCl_3_), 7.46 (1H, t, *J* = 7.5 Hz, ArH), 7.48 (1H, d, *J* = 15.0 Hz, -C*H*=CHCO-), 7.82 (1H, d, *J* = 15.0 Hz, -CH=C*H*CO-), 7.90 (1H, dd, *J* = 8.0, 1.5 Hz, ArH), 12.95 (1H, s, OH). ^13^C-NMR (500 MHz, CDCl_3_): δ 50.1, 55.8, 68.2, 111.7, 113.4, 117.7, 118.4, 118.6, 119.9, 124.6, 127.3, 129.4, 136.1, 145.2, 147.9, 152.5, 163.4, 193.3. LCMS (ES+) *m*/*z* = 340.3 ([M + H]^+^, *t*_R_ = 1.97 min). HRMS (ESI+) *m*/*z* = 340.1301 [M + H]^+^ found, C_18_H_18_N_3_O_4_^+^ required 340.1297.

*4′-(2-Azidoethoxy)-3-hydroxyflavone* (**79**). A mixture of chalcone **61** (2.02 g, 6.53 mmol), 16% NaOH (12.9 mL) and 15% H_2_O_2_ (6.47 mL) in MeOH (50 mL) was reacted according to GP-F. The crude residue was purified by flash column chromatography (SiO_2_, 1% MeOH/CH_2_Cl_2_) to afford flavonol **79** (1.68 g, 80%) as an off-white fluffy solid. m.p. 158–160 °C. TLC *R*_f_ = 0.39 (0.5% MeOH/CH_2_Cl_2_). IR ν_max_ (neat)/cm^−1^: 3235w(br) (O-H str), 2957w (C-H str), 2942w (C-H str), 2140m (N_3_ str), 2091m, 1739w, 1604s (C=O str), 1574m (C=C str), 1512s (C=C str), 1478m, 1425m, 1406m, 1346m, 1254s, 1201s, 1182s, 1117s, 1107s, 1057s. ^1^H-NMR (500 MHz, CDCl_3_): δ 3.66 (2H, t, *J* = 5.2 Hz, -OCH_2_C*H*_2_N_3_), 4.24 (2H, t, *J* = 5.2 Hz, -OC*H*_2_CH_2_N_3_), 7.03 (1H, br s, OH), 7.07 (2H, d, *J* = 8.8 Hz, ArH), 7.41 (1H, t, *J* = 7.6 Hz, ArH), 7.58 (1H, d, *J* = 8.4 Hz, ArH), 7.70 (1H, t, *J* = 8.4 Hz, ArH), 8.25 (3H, d, *J* = 8.8 Hz, ArH). ^13^C-NMR (500 MHz, CDCl_3_): δ 50.1, 67.0, 114.6, 118.1, 120.7, 124.2, 124.4, 125.4, 129.6, 133.4, 137.7, 145.0, 155.2, 159.6, 173.1. LCMS (ES+) *m*/*z* = 324.1 ([M + H]^+^, *t*_R_ = 1.66 min). These characterisation data are in accordance with that previously reported in the literature [51].

*4′-(2-Azidoethoxy)-3-hydroxy-7-methoxyflavone* (**80**). A mixture of chalcone **74** (1.06 g, 3.12 mmol), 16% NaOH (5.89 mL) and 15% H_2_O_2_ (2.95 mL) in MeOH (30 mL) was reacted according to GP-F. The crude residue was purified by flash column chromatography (SiO_2_, 1% MeOH/CH_2_Cl_2_) to afford flavonol **80** (677 mg, 61%) as an off-white powdery solid. m.p. 172–174 °C. TLC *R*_f_ = 0.39 (1% MeOH/CH_2_Cl_2_). IR *ν*_max_ (neat)/cm^−1^: 3269w(br) (O-H str), 2932w (C-H str), 2116s (N_3_ str), 2073m, 1597s (C=O str), 1561s (C=C str), 1504s (C=C str), 1451m, 1403m, 1256s, 1239s, 1203s, 1171s, 1114m, 1098m, 1050m, 1026m. ^1^H-NMR (500 MHz, CDCl_3_): δ 3.66 (2H, t, *J* = 5.2 Hz, -OCH_2_C*H*_2_N_3_), 3.94 (3H, s, -OCH_3_), 4.23 (2H, t, *J* = 5.2 Hz, -OC*H*_2_CH_2_N_3_), 6.94 (1H, d, *J* = 2.0 Hz, ArH), 6.98 (1H, dd, *J* = 8.8, 2.0 Hz, ArH), 7.04 (1H, br s, OH), 7.05 (2H, d, *J* = 9.2 Hz, ArH), 8.12 (1H, d, *J* = 8.8 Hz, ArH), 8.21 (2H, d, *J* = 8.8 Hz, ArH). ^13^C-NMR (500 MHz, CDCl_3_): δ 50.1, 55.8, 67.0, 99.8, 114.5, 114.6, 114.7, 124.3, 126.6, 129.2, 137.4, 144.3, 157.1, 159.3, 164.1, 172.6. LCMS (ES+) *m*/*z* = 354.2 ([M + H]^+^, *t*_R_ = 1.90 min). HRMS (ESI+) *m*/*z* = 354.1074 [M + H]^+^ found, C_18_H_16_O_5_N_3_^+^ required 354.1084.

*4′-(2-Azidoethoxy)-3-hydroxy-3′-methoxyflavone* (**81**). A mixture of chalcone **77** (519 mg, 1.53 mmol), 16% NaOH (2.95 mL) and 15% H_2_O_2_ (1.47 mL) in MeOH (20 mL) was reacted according to GP-F. The crude residue was purified by flash column chromatography (SiO_2_, 1% MeOH/CH_2_Cl_2_) to afford flavonol **81** (418 mg, 77%) as a pale yellow-white powdery solid. m.p. 118–120 °C. TLC *R*_f_ = 0.45 (0.5% MeOH/CH_2_Cl_2_). IR ν_max_ (neat)/cm^−1^: 3224w(br) (O-H str), 2924w (C-H str), 2125s (N_3_ str), 1614s (C=O str), 1567m (C=C str), 1514s (C=C str), 1481m, 1470m, 1409s, 1269s, 1202s, 1183m, 1146s, 1121m, 1035m, 1006m. ^1^H-NMR (500 MHz, CDCl_3_): δ 3.71 (2H, t, *J* = 5.2 Hz, -OCH_2_C*H*_2_N_3_), 3.99 (3H, s, -OCH_3_), 4.29 (2H, t, *J* = 5.2 Hz, -OC*H*_2_CH_2_N_3_), 7.01 (1H, br s, OH), 7.05 (1H, d, *J* = 9.2 Hz, ArH), 7.43 (1H, t, *J* = 7.2 Hz, ArH), 7.60 (1H, d, *J* = 8.4 Hz, ArH), 7.72 (1H, t, *J* = 8.4 Hz, ArH), 7.87–7.89 (2H, m, ArH), 8.26 (1H, dd, *J* = 8.0, 1.2 Hz, ArH). ^13^C-NMR (500 MHz, CDCl_3_): δ 50.1, 56.2, 67.9, 111.6, 113.3, 118.2, 120.6, 121.3, 124.5, 124.8, 125.4, 133.5, 137.9, 144.8, 149.5, 149.5, 155.3, 173.2. LCMS (ES+) *m*/*z* = 354.2 ([M + H]^+^, *t*_R_ = 1.86 min). HRMS (ESI+) *m*/*z* = 354.1095 [M + H]^+^ found, C_18_H_16_N_3_O_5_^+^ required 354.1090.

*3′-(2-Azidoethoxy)-3-hydroxy-4′-methoxyflavone* (**82**). A mixture of chalcone **78** (513 mg, 1.51 mmol), 16% NaOH (2.95 mL) and 15% H_2_O_2_ (1.47 mL) in MeOH (20 mL) was reacted according to GP-F. The crude residue was purified by flash column chromatography (SiO_2_, 1% MeOH/CH_2_Cl_2_) to afford flavonol **82** (419 mg, 78%) as a pale yellow-white powdery solid. m.p. 164–166 °C. TLC *R*_f_ = 0.31 (0.5% MeOH/CH_2_Cl_2_). IR ν_max_ (neat)/cm^−1^: 3199m(br) (O-H str), 2974w (C-H str), 2942w (C-H str), 2105s (N_3_ str), 1611s (C=O str), 1599m (C=O str), 1564s (C=C str), 1516s (C=C str), 1480s, 1463m, 1412s, 1350m, 1264s, 1203s, 1179s, 1141s, 1012s. ^1^H-NMR (500 MHz, CDCl_3_): δ 3.71 (2H, t, *J* = 5.2 Hz, -OCH_2_C*H*_2_N_3_), 3.97 (3H, s, -OCH_3_), 4.31 (2H, t, *J* = 5.2 Hz, -OC*H*_2_CH_2_N_3_), 7.01 (1H, br s, OH), 7.06 (1H, d, *J* = 8.4 Hz, ArH), 7.43 (1H, t, *J* = 7.6 Hz, ArH), 7.60 (1H, d, *J* = 8.4 Hz, ArH), 7.71 (1H, t, *J* = 8.4 Hz, ArH), 7.91 (1H, d, *J* = 2.0 Hz, ArH), 7.96 (1H, dd, *J* = 8.8, 2.0 Hz, ArH), 8.26 (1H, dd, *J* = 8.0, 1.2 Hz, ArH). ^13^C-NMR (500 MHz, CDCl_3_): δ 50.2, 56.0, 68.3, 111.7, 113.8, 118.2, 120.6, 122.6, 123.7, 124.5, 125.4, 133.5, 137.8, 144.8, 147.6, 151.6, 155.2, 173.1. LCMS (ES+) *m*/*z* = 354.2 ([M + H]^+^, *t*_R_ = 1.86 min). HRMS (ESI+) *m*/*z* = 354.1096 [M + H]^+^ found, C_18_H_16_N_3_O_5_^+^ required 354.1090.

*6-(2-Bromoethoxy)flavone* (**83**). A mixture of flavone **28** (1.02 g, 4.28 mmol), 1,2-dibromoethane (0.47 mL, 5.46 mmol) and anhydrous K_2_CO_3_ (782 mg, 5.66 mmol) in dry acetone (50 mL) was reacted according to GP-I. The crude residue was purified by flash column chromatography (SiO_2_, CH_2_Cl_2_) to afford flavone **83** (328 mg, 22%) as a white powdery solid. m.p. 180–182 °C. TLC *R*_f_ = 0.33 (1% MeOH/CH_2_Cl_2_). IR ν_max_ (neat)/cm^−1^: 3026w (C-H str), 2938w (C-H str), 2887w (C-H str), 1637s (C=O str), 1617m, 1586m (C=C str), 1568m (C=C str), 1482m, 1468m, 1447s, 1358s, 1313w, 1254m, 1200m, 1084m, 1030m, 1017s. ^1^H-NMR (500 MHz, CDCl_3_): δ 3.69 (2H, t, *J* = 6.0 Hz, -OCH_2_C*H*_2_Br), 4.41 (2H, t, *J* = 6.0 Hz, -OC*H*_2_CH_2_Br), 6.82 (1H, s, -C=CH), 7.34 (1H, dd, *J* = 9.2, 3.2 Hz, ArH), 7.50–7.54 (4H, m, ArH), 7.58 (1H, d, *J* = 3.2 Hz, ArH), 7.91–7.93 (2H, m, ArH). ^13^C-NMR (500 MHz, CDCl_3_): δ 28.9, 68.4, 105.9, 106.8, 119.8, 124.1, 124.5, 126.2, 129.0, 131.5, 131.7, 151.3, 155.4, 163.2, 178.1. LCMS (ES+) *m*/*z* = 347.0 ([M + H]^+^, *t*_R_ = 1.66 min). These characterisation data are in accordance with that previously reported in the literature [54].

*7-(2-Bromoethoxy)flavone* (**84**). A mixture of flavone **29** (1.59 g, 6.67 mmol), 1,2-dibromoethane (0.71 mL, 8.19 mmol) and anhydrous K_2_CO_3_ (1.20 g, 8.68 mmol) in dry acetone (50 mL) was reacted according to GP-I. The crude residue was purified by flash column chromatography (SiO_2_, 1% MeOH/CH_2_Cl_2_) to afford flavone **84** (1.50 g, 65%) as a white powdery solid. m.p. 150–152 °C. TLC *R*_f_ = 0.22 (1% MeOH/CH_2_Cl_2_). IR ν_max_ (neat)/cm^−1^: 3045w (C-H str), 2934w (C-H str), 2864w (C-H str), 1628s (C=O str), 1604s, 1594s (C=C str), 1567m (C=C str), 1494m, 1439m, 1372s, 1356s, 1282s, 1250s, 1227s, 1171s, 1131m, 1089s, 1013m. ^1^H-NMR (500 MHz, CDCl_3_): δ 3.70 (2H, t, *J* = 6.0 Hz, -OCH_2_C*H*_2_Br), 4.41 (2H, t, *J* = 6.0 Hz, -OC*H*_2_CH_2_Br), 6.76 (1H, s, -C=CH), 6.97 (1H, d, *J* = 2.4 Hz, ArH), 7.00 (1H, dd, *J* = 8.8, 2.4 Hz, ArH), 7.49–7.54 (3H, m, ArH), 7.88–7.91 (2H, m, ArH), 8.14 (1H, d, *J* = 8.8 Hz, ArH). ^13^C-NMR (500 MHz, CDCl_3_): δ 28.3, 68.2, 101.3, 107.5, 114.4, 118.3, 126.1, 127.3, 129.0, 131.4, 131.7, 157.8, 162.4, 163.0, 177.7. LCMS (ES+) *m*/*z* = 347.0 ([M + H]^+^, *t*_R_ = 1.69 min). These characterisation data are in accordance with that previously reported in the literature [28].

*6-(2-Azidoethoxy)flavone* (**85**). A mixture of flavone **83** (824 mg, 2.39 mmol) and NaN_3_ (336 mg, 5.17 mmol) in dry DMF (20 mL) was reacted according to GP-J. The reaction mixture was worked up to afford flavone **85** (686 mg, 93%) as a white powdery solid and was used without further purification. m.p. 138–140 °C. TLC *R*_f_ = 0.27 (1% MeOH/CH_2_Cl_2_). IR *ν*_max_ (neat)/cm^−1^: 3073w (C-H str), 2930w (C-H str), 2107s (N_3_ str), 2063m, 1625s, 1616s (C=O str), 1603s, 1582s (C=C str), 1569s (C=C str), 1480s, 1455s, 1363s, 1291s, 1262m, 1242s, 1203s, 1130s, 1083m. ^1^H-NMR (500 MHz, CDCl_3_): δ 3.65 (2H, t, *J* = 4.8 Hz, -OCH_2_C*H*_2_N_3_), 4.26 (2H, t, *J* = 4.8 Hz, -OC*H*_2_CH_2_N_3_), 6.81 (1H, s, -C=CH), 7.34 (1H, dd, *J* = 8.8, 2.8 Hz, ArH), 7.51–7.54 (4H, m, ArH), 7.59 (1H, d, *J* = 2.8 Hz, ArH), 7.90–7.93 (2H, m, ArH). ^13^C-NMR (500 MHz, CDCl_3_): δ 50.0, 67.5, 105.6, 106.9, 119.8, 124.2, 124.5, 126.2, 129.0, 131.5, 131.8, 151.3, 155.5, 163.3, 178.1. LCMS (ES+) *m*/*z* = 308.2 ([M + H]^+^, *t*_R_ = 1.68 min). HRMS (ESI+) *m*/*z* = 330.0836 [M + Na]^+^ found, C_17_H_13_O_3_N_3_Na^+^ required 330.0849.

*7-(2-Azidoethoxy)flavone* (**86**). A mixture of flavone **84** (1.03 g, 3.00 mmol) and NaN_3_ (444 mg, 6.83 mmol) in dry DMF (20 mL) was reacted according to GP-J. The reaction mixture was worked up to afford flavone **86** (838 mg, 91%) as an off-white powdery solid and was used without further purification. m.p. 108–110 °C. TLC *R*_f_ = 0.23 (1% MeOH/CH_2_Cl_2_). IR ν_max_ (neat)/cm^−1^: 3073w (C-H str), 2979w (C-H str), 2926w (C-H str), 2105m (N_3_ str), 1631s (C=O str), 1601s, 1578m (C=C str), 1569m (C=C str), 1493w, 1447s, 1375m, 1358m, 1303m, 1244s, 1177s, 1130m, 1085s. ^1^H-NMR (500 MHz, CDCl_3_): δ 3.68 (2H, t, *J* = 5.0 Hz, -OCH_2_C*H*_2_N_3_), 4.26 (2H, t, *J* = 5.0 Hz, -OC*H*_2_CH_2_N_3_), 6.75 (1H, s, -C=CH), 6.97 (1H, d, *J* = 2.0 Hz, ArH), 6.99 (1H, dd, *J* = 8.5, 2.0 Hz, ArH), 7.48–7.54 (3H, m, ArH), 7.88 (2H, dd, *J* = 8.0, 2.0 Hz, ArH), 8.13 (1H, d, *J* = 8.5 Hz, ArH). ^13^C-NMR (500 MHz, CDCl_3_): δ 49.8, 67.4, 101.3, 107.5, 114.3, 118.2, 126.1, 127.2, 128.9, 131.4, 131.6, 157.7, 162.6, 163.0, 177.6. LCMS (ES+) *m*/*z* = 308.0 ([M + H]^+^, *t*_R_ = 4.43 min). These characterisation data are in accordance with that previously reported in the literature [10].

*4’-(2-Azidoethoxy)-7-methoxyflavone* (**87**). To a stirred solution of chalcone **76** (1.02 g, 3.01 mmol) in DMSO (20 mL) was added a catalytic amount of I_2_ (103 mg, 0.407 mmol). The reaction mixture was heated at 130 °C with stirring for 24 h under a nitrogen atmosphere. The resulting mixture was allowed to cool to room temperature and poured into H_2_O (50 mL). The aqueous solution was extracted with CHCl_3_ (2 × 100 mL) and the combined organic layer was washed with H_2_O (2 × 50 mL), brine (2 × 50 mL), dried over anhydrous MgSO_4_, filtered and the solvent removed under reduced pressure. The crude residue was purified by flash column chromatography (SiO_2_, 1% MeOH/CH_2_Cl_2_) to afford flavone **87** (924 mg, 91%) as a pale yellow-white powdery solid. m.p. 128–130 °C. TLC *R*_f_ = 0.35 (3% MeOH/CH_2_Cl_2_). IR ν_max_ (neat)/cm^−1^: 2946w (C-H str), 2839w (C-H str), 2097s (N_3_ str), 1644s (C=O str), 1626s, 1598s (C=C str), 1510s (C=C str), 1503s (C=C str), 1440s, 1376m, 1355s, 1247s, 1182s, 1164m, 1141w, 1091m, 1023m. ^1^H-NMR (500 MHz, CDCl_3_): δ 3.65 (2H, t, *J* = 5.2 Hz, -OCH_2_C*H*_2_N_3_), 3.92 (3H, s, -OCH_3_), 4.22 (2H, t, *J* = 5.2 Hz, -OC*H*_2_CH_2_N_3_), 6.66 (1H, s, -C=CH), 6.94 (1H, d, *J* = 2.4 Hz, ArH), 6.96 (1H, dd, *J* = 8.8, 2.4 Hz, ArH), 7.02 (2H, d, *J* = 9.2 Hz, ArH), 7.85 (2H, d, *J* = 9.2 Hz, ArH), 8.11 (1H, d, *J* = 8.8 Hz, ArH). ^13^C-NMR (500 MHz, CDCl_3_): δ 50.0, 55.8, 67.1, 100.3, 106.2, 114.2, 114.9, 117.7, 124.7, 126.9, 127.8, 157.8, 160.7, 162.7, 164.0, 177.7. LCMS (ES+) *m*/*z* = 338.2 ([M + H]^+^, *t*_R_ = 1.84 min). HRMS (ESI+) *m*/*z* = 338.1147 [M + H]^+^ found, C_18_H_16_N_3_O_4_^+^ required 338.1141. 

*4′-(2-Azidoethoxy)aurone* (**88**). A mixture of chalcone **61** (403 mg, 1.30 mmol) and Hg(OAc)_2_ (440 mg, 1.38 mmol) in dry pyridine (15 mL) was reacted according to GP-K. The crude residue was purified by flash column chromatography (SiO_2_, PE/EtOAc; 3:1) to afford aurone **88** (354 mg, 88%) as a bright yellow powdery solid. m.p. 114–116 °C. TLC *R*_f_ = 0.37 (PE/EtOAc; 2:1). IR ν_max_ (neat)/cm^−1^: 2945w (C-H str), 2888w (C-H str), 2096s (N_3_ str), 2062m, 1699s (C=O str), 1649s, 1592s (C=C str), 1510m (C=C str), 1474m, 1461m, 1395w, 1347m, 1301s, 1257s, 1177s, 1130m, 1111m, 1097w, 1048s, 1009w. ^1^H-NMR (500 MHz, CDCl_3_): δ 3.65 (2H, t, *J* = 4.8 Hz, -OCH_2_C*H*_2_N_3_), 4.22 (2H, t, *J* = 4.8 Hz, -OC*H*_2_CH_2_N_3_), 6.89 (1H, s, -C=CH), 7.01 (2H, d, *J* = 8.8 Hz, ArH), 7.22 (1H, t, *J* = 7.6 Hz, ArH), 7.33 (1H, d, *J* = 8.4 Hz, ArH), 7.65 (1H, t, *J* = 8.4 Hz, ArH), 7.82 (1H, dd, *J* = 7.6, 0.4 Hz, ArH), 7.91 (2H, d, *J* = 8.8 Hz, ArH). ^13^C-NMR (500 MHz, CDCl_3_): δ 50.0, 67.0, 112.9, 113.0, 115.0, 121.8, 123.3, 124.6, 125.7, 133.4, 136.6, 146.0, 159.6, 165.9, 184.6. LCMS (ES+) *m*/*z* = 308.2 ([M + H]^+^, *t*_R_ = 1.73 min). HRMS (ESI+) *m*/*z* = 308.1035 [M + H]^+^ found, C_17_H_14_O_3_N_3_^+^ required 308.1035.

*4′-(2-Azidoethoxy)-6-methoxyaurone* (**89**). A mixture of chalcone **76** (2.01 g, 5.91 mmol) and Hg(OAc)_2_ (1.95 g, 6.13 mmol) in dry pyridine (50 mL) was reacted according to GP-K. The crude residue was purified by flash column chromatography (SiO_2_, 0.5% MeOH/CH_2_Cl_2_) to afford aurone **89** (1.91 g, 96%) as a bright yellow-orange powdery solid. m.p. 108–110 °C. TLC *R*_f_ = 0.36 (0.5% MeOH/CH_2_Cl_2_). IR ν_max_ (neat)/cm^−1^: 3008w (C-H str), 2970w (C-H str), 2092s (N_3_ str), 1694m (C=O str), 1652m, 1594s (C=C str), 1570m (C=C str), 1512m (C=C str), 1443m, 1414w, 1349m, 1315w, 1271m, 1248s, 1185m, 1131s, 1110s, 1068w, 1035m. ^1^H-NMR (500 MHz, CDCl_3_): δ 3.63 (2H, t, *J* = 4.8 Hz, -OCH_2_C*H*_2_N_3_), 3.91 (3H, s, -OCH_3_), 4.19 (2H, t, *J* = 4.8 Hz, -OC*H*_2_CH_2_N_3_), 6.72–6.74 (2H, m, ArH), 6.76 (1H, s, -C=CH), 6.97 (2H, d, *J* = 8.8 Hz, ArH), 7.68 (1H, d, *J* = 8.8 Hz, ArH), 7.83 (2H, d, *J* = 8.8 Hz, ArH). ^13^C-NMR (500 MHz, CDCl_3_): δ 50.0, 55.9, 66.9, 96.5, 111.7, 112.0, 114.9, 115.0, 125.6, 125.7, 133.1, 146.8, 159.3, 167.2, 168.2, 182.8. LCMS (ES+) *m*/*z* = 338.2 ([M + H]^+^, *t*_R_ = 1.65 min). HRMS (ESI+) *m*/*z* = 338.1146 [M + H]^+^ found, C_18_H_16_N_3_O_4_^+^ required 338.1141.

### 3.3. Synthesis of Triazole-Bridged Flavonoid Dimers and Trimers

*(E)-3-(3,4-Dimethoxyphenyl)-1-(3-methoxy-4-(2-(4-((2-methoxy-4-((E)-3-(4-methoxyphenyl)acryloyl))methyl)-1H-1,2,3-triazol-1-yl)ethoxy)phenyl)prop-2-en-1-one* (**90**). A mixture of alkyne chalcone **20** (254 mg, 0.789 mmol), azide chalcone **67** (305 mg, 0.796 mmol), CuSO_4_·5H_2_O (225 mg, 0.900 mmol) and sodium ascorbate (389 mg, 1.97 mmol) in *t*-BuOH/H_2_O (1:1, 40 mL) was reacted according to GP-A. The crude residue was purified by flash column chromatography (SiO_2_, 1%–5% MeOH/CH_2_Cl_2_) and recrystallized from MeOH to afford triazole hybrid **90** (151 mg, 27%) as a pale yellow-brown powdery solid. m.p. 108–110 °C. TLC *R*_f_ = 0.41 (5% MeOH/CH_2_Cl_2_). IR ν_max_ (neat)/cm^−1^: 2942w (C-H str), 2837w (C-H str), 1652m (C=O str), 1595m (C=C str), 1572m (C=C str), 1509s (C=C str), 1464m, 1420m, 1335w, 1307w, 1255s, 1195w, 1143s, 1021s. ^1^H-NMR (500 MHz, CDCl_3_): δ 3.86 (3H, s, -OCH_3_), 3.92 (3H, s, -OCH_3_), 3.94 (3H, s, -OCH_3_), 3.95 (3H, s, -OCH_3_), 3.96 (3H, s, -OCH_3_), 4.46 (2H, t, *J* = 5.2 Hz, -OCH_2_C*H*_2_N-), 4.84 (2H, t, *J* = 5.2 Hz, -OC*H*_2_CH_2_N-), 5.40 (2H, s, -OC*H*_2_CN-), 6.82 (1H, d, *J* = 8.0 Hz, ArH), 6.90 (1H, d, *J* = 8.4 Hz, ArH), 6.93 (2H, d, *J* = 8.8 Hz, ArH), 7.14–7.17 (2H, m, ArH), 7.24 (1H, dd, *J* = 8.4, 2.0 Hz, ArH), 7.37 (1H, d, *J* = 15.2 Hz, -C*H*=CHCO-), 7.41 (1H, d, *J* = 15.6 Hz, -C*H*=CHCO-), 7.57–7.62 (6H, m, ArH), 7.75 (1H, d, *J* = 15.6 Hz, -CH=C*H*CO-), 7.77 (1H, d, *J* = 15.6 Hz, -CH=C*H*CO-), 8.04 (1H, s, -C*H*N-). ^13^C-NMR (500 MHz, CDCl_3_): δ 49.8, 55.4, 56.0, 56.0, 62.9, 67.6, 110.2, 111.1, 111.1, 111.5, 112.3, 112.4, 114.4, 119.2, 119.4, 122.5, 122.6, 123.0, 124.7, 127.7, 127.9, 130.1, 132.1, 132.8, 143.7, 144.0, 144.5, 149.2, 149.5, 149.7, 151.2, 151.4, 151.6, 161.5, 188.6. LCMS (ES+) *m*/*z* = 706.2 ([M + H]^+^, *t*_R_ = 1.65 min). HRMS (ESI+) *m*/*z* = 728.2576 [M + Na]^+^ found, C_40_H_39_O_9_N_3_Na^+^ required 728.2579.

*(E)-1-(3-Methoxy-4-((1-(2-(2-methoxy-4-((E)-3-(2,4,6-trimethoxyphenyl)acryloyl)phenoxy)ethyl)-1H-1,2,3-triazol-4-yl)methoxy)phenyl)-3-(4-methoxyphenyl)prop-2-en-1-one* (**91**). A mixture of alkyne chalcone **20** (234 mg, 0.727 mmol), azide chalcone **69** (303 mg, 0.732 mmol), CuSO_4_·5H_2_O (203 mg, 0.813 mmol) and sodium ascorbate (361 mg, 1.82 mmol) in *t*-BuOH/H_2_O (1:1, 40 mL) was reacted according to GP-A. The crude residue was purified by flash column chromatography (SiO_2_, 1%–5% MeOH/CH_2_Cl_2_) and recrystallized from MeOH to afford triazole hybrid **91** (250 mg, 47%) as a pale yellow-white flaky solid. m.p. 206–208 °C. TLC *R*_f_ = 0.44 (5% MeOH/CH_2_Cl_2_). IR ν_max_ (neat)/cm^−1^: 3150w (C-H str), 2942w (C-H str), 1661m (C=O str), 1601s (C=C str), 1572s (C=C str), 1514s (C=C str), 1459m, 1416m, 1324s, 1295m, 1262s, 1228m, 1166s, 1150s, 1126s, 1053w, 1015s. ^1^H-NMR (500 MHz, CDCl_3_): δ 3.86 (3H, s, -OCH_3_), 3.87 (3H, s, -OCH_3_), 3.91 (6H, s, 2 × -OCH_3_), 3.92 (3H, s, -OCH_3_), 3.95 (3H, s, -OCH_3_), 4.46 (2H, t, *J* = 5.2 Hz, -OCH_2_C*H*_2_N-), 4.84 (2H, t, *J* = 5.2 Hz, -OC*H*_2_CH_2_N-), 5.40 (2H, s, -OC*H*_2_CN-), 6.15 (2H, s, ArH), 6.85 (1H, d, *J* = 8.4 Hz, ArH), 6.94 (2H, d, *J* = 8.8 Hz, ArH), 7.17 (1H, d, *J* = 8.8 Hz, ArH), 7.42 (1H, d, *J* = 15.6 Hz, -C*H*=CHCO-), 7.58–7.65 (6H, m, ArH), 7.78 (1H, d, *J* = 15.2 Hz, -CH=C*H*CO-), 7.85 (1H, d, *J* = 16.0 Hz, -C*H*=CHCO-), 8.05 (1H, s, -C*H*N-), 8.23 (1H, d, *J* = 16.0 Hz, -CH=C*H*CO-). ^13^C-NMR (500 MHz, CDCl_3_): δ 49.8, 55.4, 55.8, 55.9, 56.0, 62.9, 67.5, 90.5, 106.6, 111.1, 111.7, 112.3, 112.4, 114.4, 119.3, 121.6, 122.3, 122.6, 124.7, 127.8, 130.1, 132.1, 133.7, 135.6, 143.7, 143.9, 149.5, 149.5, 150.6, 151.6, 161.5, 161.7, 163.0, 188.6, 190.3. LCMS (ES+) *m*/*z* = 736.2 ([M + H]^+^, *t*_R_ = 1.67 min). HRMS (ESI+) *m*/*z* = 736.2892 [M + H]^+^ found, C_41_H_42_O_10_N_3_^+^ required 736.2865.

*(E)-1-(2-Hydroxy-4-methoxyphenyl)-3-(4-methoxy-3-((1-(2-(2-methoxy-4-((E)-3-(2,4,6-trimethoxyphenyl)acryloyl)phenoxy)ethyl)-1H-1,2,3-triazol-4-yl)methoxy)phenyl)prop-2-en-1-one* (**92**). A mixture of alkyne chalcone **4** (167 mg, 0.494 mmol), azide chalcone **69** (206 mg, 0.498 mmol), CuSO_4_·5H_2_O (140 mg, 0.560 mmol) and sodium ascorbate (253 mg, 1.28 mmol) in *t*-BuOH/H_2_O (1:1, 40 mL) was reacted according to GP-A. The crude residue was purified by flash column chromatography (SiO_2_, 1%–5% MeOH/CH_2_Cl_2_) and recrystallized from MeOH to afford triazole hybrid **92** (236 mg, 64%) as a bright yellow flaky solid. m.p. 208–210 °C. TLC *R*_f_ = 0.48 (5% MeOH/CH_2_Cl_2_). IR ν_max_ (neat)/cm^−1^: 2931w (C-H str), 2842w (C-H str), 1632m (C=O str), 1597m (C=C str), 1568s (C=C str), 1509s (C=C str), 1455w, 1419w, 1371m, 1318m, 1259s, 1232s, 1211s, 1153s, 1123s, 1021m, 1004m. ^1^H-NMR (500 MHz, CDCl_3_): δ 3.86 (3H, s, -OCH_3_), 3.87 (3H, s, -OCH_3_), 3.91 (3H, s, -OCH_3_), 3.91 (9H, s, 3 × -OCH_3_), 4.45 (2H, t, *J* = 4.8 Hz, -OCH_2_C*H*_2_N-), 4.84 (2H, t, *J* = 4.8 Hz, -OC*H*_2_CH_2_N-), 5.38 (2H, s, -OC*H*_2_CN-), 6.14 (2H, s, ArH), 6.46 (1H, d, *J* = 2.4 Hz, ArH), 6.50 (1H, dd, *J* = 8.8, 2.4 Hz, ArH), 6.81 (1H, d, *J* = 8.4 Hz, ArH), 6.89 (1H, d, *J* = 2.4 Hz, ArH), 7.21 (1H, dd, *J* = 8.0, 2.0 Hz, ArH), 7.45 (1H, d, *J* = 15.2 Hz, -C*H*=CHCO-), 7.46 (1H, d, *J* = 1.6 Hz, ArH), 7.55 (1H, dd, *J* = 8.4, 1.6 Hz, ArH), 7.61 (1H, d, *J* = 1.6 Hz, ArH), 7.77 (1H, d, *J* = 15.6 Hz, -CH=C*H*CO-), 7.85 (1H, d, *J* = 16.0 Hz, -C*H*=CHCO-), 7.89 (1H, d, *J* = 8.8 Hz, ArH), 8.05 (1H, s, -C*H*N-), 8.23 (1H, d, *J* = 16.0 Hz, -CH=C*H*CO-), 13.55 (1H, s, OH). ^13^C-NMR (500 MHz, CDCl_3_): δ 49.8, 55.4, 55.5, 55.8, 55.9, 56.0, 63.2, 67.6, 90.5, 101.0, 106.6, 107.6, 111.5, 111.6, 112.3, 113.0, 114.2, 118.4, 121.5, 122.3, 124.3, 124.7, 127.8, 131.3, 133.7, 135.6, 144.0, 144.1, 147.8, 149.4, 150.6, 152.0, 161.7, 163.0, 166.0, 166.6, 190.3, 191.8. LCMS (ES+) *m*/*z* = 752.2 ([M + H]^+^, *t*_R_ = 1.74 min). HRMS (ESI+) *m*/*z* = 752.2822 [M + H]^+^ found, C_41_H_42_O_11_N_3_^+^ required 752.2814.

*(E)-1-(3-Methoxy-4-((1-(2-(2-methoxy-4-((E)-3-(2,3,4-trimethoxyphenyl)acryloyl)phenoxy)ethyl)-1H-1,2,3-triazol-4-yl)methoxy)phenyl)-3-(4-methoxyphenyl)prop-2-en-1-one* (**93**). A mixture of alkyne chalcone **20** (235 mg, 0.729 mmol), azide chalcone **68** (303 mg, 0.733 mmol), CuSO_4_·5H_2_O (271 mg, 1.09 mmol) and sodium ascorbate (414 mg, 2.09 mmol) in *t*-BuOH/H_2_O (1:1, 40 mL) was reacted according to GP-A. The crude residue was purified by flash column chromatography (SiO_2_, 1%–5% MeOH/CH_2_Cl_2_) and recrystallized from MeOH to afford triazole hybrid **93** (515 mg, 96%) as a pale yellow-green flaky solid. m.p. 128–130 °C. TLC *R*_f_ = 0.30 (4% MeOH/CH_2_Cl_2_). IR ν_max_ (neat)/cm^−1^: 2937w (C-H str), 2836w (C-H str), 1652m (C=O str), 1595s (C=C str), 1572s (C=C str), 1510s (C=C str), 1494s, 1463s, 1415s, 1326w, 1255s, 1193w, 1148s, 1095s, 1021s. ^1^H-NMR (500 MHz, CDCl_3_): δ 3.86 (3H, s, -OCH_3_), 3.90 (3H, s, -OCH_3_), 3.92 (6H, s, 2 × -OCH_3_), 3.95 (6H, s, 2 × -OCH_3_), 4.47 (2H, t, *J* = 4.8 Hz, -OCH_2_C*H*_2_N-), 4.84 (2H, t, *J* = 4.8 Hz, -OC*H*_2_CH_2_N-), 5.40 (2H, s, -OC*H*_2_CN-), 6.73 (1H, d, *J* = 8.8 Hz, ArH), 6.84 (1H, d, *J* = 8.4 Hz, ArH), 6.94 (2H, d, *J* = 8.8 Hz, ArH), 7.17 (1H, d, *J* = 8.8 Hz, ArH), 7.39 (1H, d, *J* = 8.8 Hz, ArH), 7.42 (1H, d, *J* = 15.6 Hz, -C*H*=CHCO-), 7.54 (1H, d, *J* = 16.0 Hz, -C*H*=CHCO-), 7.59–7.64 (6H, m, ArH), 7.78 (1H, d, *J* = 15.6 Hz, -CH=C*H*CO-), 7.98 (1H, d, *J* = 16.0 Hz, -CH=C*H*CO-), 8.05 (1H, s, -C*H*N-). ^13^C-NMR (500 MHz, CDCl_3_): δ 49.7, 55.4, 56.0, 56.0, 56.1, 60.9, 61.4, 62.9, 67.5, 107.6, 111.1, 111.6, 112.3, 112.4, 114.4, 119.2, 120.8, 122.0, 122.5, 122.6, 123.9, 124.7, 127.7, 130.1, 132.1, 133.0, 139.7, 142.5, 143.7, 144.0, 149.5, 149.7, 151.1, 151.6, 153.8, 155.7, 161.5, 188.6, 189.0. LCMS (ES+) *m*/*z* = 736.2 ([M + H]^+^, *t*_R_ = 1.66 min). HRMS (ESI+) *m*/*z* = 736.2855 [M + H]^+^ found, C_41_H_42_O_10_N_3_^+^ required 736.2870.

*(E)-1-(3-Methoxy-4-((1-(2-(2-methoxy-4-((E)-3-(2,4,6-trimethoxyphenyl)acryloyl)phenoxy)ethyl)-1H-1,2,3-triazol-4-yl)methoxy)phenyl)-3-(1-methyl-1H-pyrrol-2-yl)prop-2-en-1-one* (**94**). A mixture of alkyne chalcone **22** (217 mg, 0.734 mmol), azide chalcone **69** (305 mg, 0.737 mmol), CuSO_4_·5H_2_O (214 mg, 0.857 mmol) and sodium ascorbate (365 mg, 1.84 mmol) in *t*-BuOH/H_2_O (1:1, 40 mL) was reacted according to GP-A. The crude residue was purified by flash column chromatography (SiO_2_, 1%–5% MeOH/CH_2_Cl_2_) and recrystallized from MeOH to afford triazole hybrid **94** (367 mg, 70%) as a bright yellow flaky solid. m.p. 138–140 °C. TLC *R*_f_ = 0.31 (5% MeOH/CH_2_Cl_2_). IR ν_max_ (neat)/cm^−1^: 2941w (C-H str), 2838w (C-H str), 1646m (C=O str), 1596m (C=C str), 1566s (C=C str), 1512m (C=C str), 1470m, 1457m, 1414m, 1338w, 1321m, 1257s, 1198m, 1155s, 1146s, 1118s, 1027m. ^1^H-NMR (500 MHz, CDCl_3_): δ 3.77 (3H, s, -NCH_3_), 3.86 (3H, s, -OCH_3_), 3.91 (6H, s, 2 × -OCH_3_), 3.91 (3H, s, -OCH_3_), 3.94 (3H, s, -OCH_3_), 4.45 (2H, t, *J* = 4.8 Hz, -OCH_2_C*H*_2_N-), 4.83 (2H, t, *J* = 4.8 Hz, -OC*H*_2_CH_2_N-), 5.38 (2H, s, -OC*H*_2_CN-), 6.14 (2H, s, ArH), 6.21–6.23 (1H, m, ArH), 6.80–6.86 (3H, m, ArH), 7.16 (1H, d, *J* = 8.8 Hz, ArH), 7.29 (1H, d, *J* = 15.6 Hz, -C*H*=CHCO-), 7.57–7.63 (4H, m, ArH), 7.79 (1H, d, *J* = 15.2 Hz, -CH=C*H*CO-), 7.85 (1H, d, *J* = 16.0 Hz, -C*H*=CHCO-), 8.05 (1H, s, -C*H*N-), 8.23 (1H, d, *J* = 15.6 Hz, -CH=C*H*CO-). ^13^C-NMR (500 MHz, CDCl_3_): δ 34.4, 49.7, 55.4, 55.8, 55.9, 56.0, 62.8, 67.5, 90.5, 106.5, 109.6, 111.1, 111.7, 112.1, 112.3, 112.4, 116.3, 121.5, 122.3, 122.3, 124.7, 127.6, 130.3, 131.6, 132.4, 133.7, 135.6, 143.7, 149.4, 149.5, 150.6, 151.4, 161.6, 163.0, 188.1, 190.3. LCMS (ES+) *m*/*z* = 709.3 ([M + H]^+^, *t*_R_ = 1.63 min). HRMS (ESI+) *m*/*z* = 709.2854 [M + H]^+^ found, C_39_H_41_O_9_N_4_^+^ required 709.2868.

*(E)-1-(3-Methoxy-4-((1-(2-(2-methoxy-4-((E)-3-(1-methyl-1H-pyrrol-2-yl)acryloyl)phenoxy)ethyl)-1H-1,2,3-triazol-4-yl)methoxy)phenyl)-3-(1-methyl-1H-pyrrol-2-yl)prop-2-en-1-one* (**95**). A mixture of alkyne chalcone **22** (271 mg, 0.918 mmol), azide chalcone **70** (301 mg, 0.924 mmol), CuSO_4_·5H_2_O (316 g, 1.26 mmol) and sodium ascorbate (499 mg, 2.52 mmol) in *t*-BuOH/H_2_O (1:1, 40 mL) was reacted according to GP-A. The crude residue was purified by flash column chromatography (SiO_2_, 1%–5% MeOH/CH_2_Cl_2_) and recrystallized from MeOH to afford triazole hybrid **95** (555 mg, 97%) as a dark yellow-brown flaky solid. m.p. 118–120 °C. TLC *R*_f_ = 0.38 (5% MeOH/CH_2_Cl_2_). IR ν_max_ (neat)/cm^−1^: 2941w (C-H str), 2844w (C-H str), 1644m (C=O str), 1594m (C=C str), 1564s (C=C str), 1511m (C=C str), 1480m, 1412m, 1381w, 1330m, 1258s, 1196m, 1153s, 1129m, 1055w, 1025m. ^1^H-NMR (500 MHz, CDCl_3_): δ 3.76 (3H, s, -NCH_3_), 3.77 (3H, s, -NCH_3_), 3.92 (3H, s, -OCH_3_), 3.95 (3H, s, -OCH_3_), 4.46 (2H, t, *J* = 4.8 Hz, -OCH_2_C*H*_2_N-), 4.84 (2H, t, *J* = 4.8 Hz, -OC*H*_2_CH_2_N-), 5.39 (2H, s, -OC*H*_2_CN-), 6.22–6.24 (2H, m, ArH), 6.82–6.86 (5H, m, ArH), 7.15 (1H, d, *J* = 8.0 Hz, ArH), 7.27 (1H, d, *J* = 15.2 Hz, -C*H*=CHCO-, overlain by CDCl_3_), 7.29 (1H, d, *J* = 14.0 Hz, -C*H*=CHCO-), 7.57–7.62 (4H, m, ArH), 7.79 (2H, d, *J* = 14.8 Hz, 2 × -CH=C*H*CO-), 8.04 (1H, s, -C*H*N-). ^13^C-NMR (500 MHz, CDCl_3_): δ 34.4, 49.7, 55.9, 56.0, 62.9, 67.5, 109.6, 109.7, 111.1, 111.4, 112.1, 112.2, 112.3, 112.4, 116.2, 116.3, 122.1, 122.3, 124.7, 127.6, 127.7, 130.3, 130.3, 131.6, 131.8, 132.4, 133.1, 143.7, 149.4, 149.6, 150.9, 151.4, 188.0, 188.1. LCMS (ES+) *m*/*z* = 622.3 ([M + H]^+^, *t*_R_ = 1.62 min). HRMS (ESI+) *m*/*z* = 622.2648 [M + H]^+^ found, C_35_H_36_O_6_N_5_^+^ required 622.2660.

*(E)-1-(2-Hydroxyphenyl)-3-(4-(2-(4-((2-((E)-3-(1-methyl-1H-pyrrol-2-yl)acryloyl)phenoxy)methyl)-1H-1,2,3-triazol-1-yl)ethoxy)phenyl)prop-2-en-1-one* (**96**). A mixture of alkyne chalcone **23** (337 mg, 1.27 mmol), azide chalcone **61** (400 mg, 1.29 mmol), CuSO_4_·5H_2_O (429 mg, 1.72 mmol) and sodium ascorbate (641 mg, 3.23 mmol) in *t*-BuOH/H_2_O (1:1, 40 mL) was reacted according to GP-A. The crude residue was purified by flash column chromatography (SiO_2_, 1%–5% MeOH/CH_2_Cl_2_) to afford triazole hybrid **96** (399 mg, 55%) as a bright yellow-orange powdery solid. m.p. 98–100 °C. TLC *R*_f_ = 0.40 (3% MeOH/CH_2_Cl_2_). IR ν_max_ (neat)/cm^−1^: 2933w (C-H str), 2884w (C-H str), 1635m (C=O str), 1597m (C=C str), 1562s (C=C str), 1509m (C=C str), 1480m, 1447m, 1329m, 1286m, 1268m, 1233m, 1202s, 1174s, 1156s, 1113w, 1055m, 1025s. ^1^H-NMR (500 MHz, CDCl_3_): δ 3.65 (3H, s, -NCH_3_), 4.32 (2H, t, *J* = 5.2 Hz, -OCH_2_C*H*_2_N-), 4.65 (2H, t, *J* = 5.2 Hz, -OC*H*_2_CH_2_N-), 5.33 (2H, s, -OC*H*_2_CN-), 6.16–6.18 (1H, m, ArH), 6.63 (1H, dd, *J* = 4.0, 1.2 Hz, ArH), 6.76 (1H, t, *J* = 2.0 Hz, ArH), 6.85 (2H, d, *J* = 8.8 Hz, ArH), 6.95 (1H, t, *J* = 8.0 Hz, ArH), 7.03 (1H, dd, *J* = 8.4, 0.8 Hz, ArH), 7.08 (1H, d, *J* = 7.6 Hz, ArH), 7.12 (1H, d, *J* = 8.4 Hz, ArH), 7.16 (1H, d, *J* = 15.6 Hz, -C*H*=CHCO-), 7.44–7.59 (4H, m, ArH), 7.54 (1H, d, *J* = 15.6 Hz, -C*H*=CHCO-), 7.60 (1H, d, *J* = 15.2 Hz, -CH=C*H*CO-), 7.66 (1H, dd, *J* = 8.0, 2.0 Hz, ArH), 7.76 (1H, s, -C*H*N-), 7.86 (1H, d, *J* = 15.6 Hz, -CH=C*H*CO-), 7.93 (1H, dd, *J* = 8.4, 1.6 Hz, ArH), 12.89 (1H, s, OH). ^13^C-NMR (500 MHz, CDCl_3_): δ 34.3, 49.5, 63.0, 66.2, 109.6, 112.4, 113.0, 114.9, 118.2, 118.5, 118.8, 120.0, 121.4, 122.2, 123.9, 127.7, 128.2, 129.5, 130.0, 130.1, 130.5, 130.6, 132.7, 136.2, 144.1, 144.8, 156.5, 159.9, 163.5, 191.9, 193.5. LCMS (ES+) *m*/*z* = 575.3 ([M + H]^+^, *t*_R_ = 1.72 min). HRMS (ESI+) *m*/*z* = 575.2266 [M + H]^+^ found, C_34_H_31_O_5_N_4_^+^ required 575.2289.

*(E)-3-(Ferrocenyl)-1-(4-((1-(2-(4-((E)-3-(2-hydroxyphenyl)-3-oxoprop-1-en-1-yl)phenoxy)ethyl)-1H-1,2,3-triazol-4-yl)methoxy)-3-methoxyphenyl)prop-2-en-1-one* (**97**). A mixture of alkyne chalcone **25** (301 mg, 0.753 mmol), azide chalcone **61** (235 mg, 0.760 mmol), CuSO_4_·5H_2_O (225 mg, 0.900 mmol) and sodium ascorbate (398 mg, 2.01 mmol) in *t*-BuOH/H_2_O (1:1, 40 mL) was reacted according to GP-A. The crude residue was purified by flash column chromatography (SiO_2_, 1%–5% MeOH/CH_2_Cl_2_) to afford triazole hybrid **97** (427 mg, 80%) as a dark red-brown powdery solid. m.p. 178–180 °C. TLC *R*_f_ = 0.28 (2% MeOH/CH_2_Cl_2_). IR ν_max_ (neat)/cm^−1^: 2937w (C-H str), 1740m (C=O str), 1651m, 1637s, 1596s (C=C str), 1571s (C=C str), 1510s (C=C str), 1487m, 1444m, 1341w, 1300m, 1266s, 1203s, 1179s, 1159s, 1146s, 1048m, 1020s. ^1^H-NMR (500 MHz, CDCl_3_): δ 3.98 (3H, s, -OCH_3_), 4.16 (5H, s, -C_5_H_5_), 4.42 (2H, t, *J* = 5.2 Hz, -OCH_2_C*H*_2_N-), 4.47 (2H, t, *J* = 2.0 Hz, -C_5_H_4_), 4.56 (2H, t, *J* = 2.0 Hz, -C_5_H_4_), 4.80 (2H, t, *J* = 5.2 Hz, -OC*H*_2_CH_2_N-), 5.43 (2H, s, -OC*H*_2_CN-), 6.84 (2H, d, *J* = 8.8 Hz, ArH), 6.97 (1H, t, *J* = 8.0 Hz, ArH), 7.04 (1H, dd, *J* = 8.4, 0.8 Hz, ArH), 7.12 (1H, d, *J* = 15.2 Hz, -C*H*=CHCO-), 7.12 (1H, d, *J* = 8.4 Hz, ArH), 7.51 (1H, t, *J* = 8.4 Hz, ArH), 7.54–7.61 (3H, m, ArH), 7.59 (1H, d, *J* = 16.0 Hz, -C*H*=CHCO-), 7.64 (1H, d, *J* = 1.6 Hz, ArH), 7.74 (1H, d, *J* = 15.6 Hz, -CH=C*H*CO-), 7.86 (1H, s, -C*H*N-), 7.86 (1H, d, *J* = 15.6 Hz, -CH=C*H*CO-), 7.97 (1H, dd, *J* = 8.0, 1.6 Hz, ArH), 12.90 (1H, s, OH). ^13^C-NMR (500 MHz, CDCl_3_): δ 49.7, 56.1, 62.8, 66.3, 68.9, 69.7, 71.3, 79.2, 111.1, 112.3, 114.9, 118.3, 118.5, 118.6, 118.8, 120.0, 122.4, 124.3, 128.4, 129.6, 130.6, 132.3, 136.3, 143.8, 144.7, 146.1, 149.5, 151.2, 159.8, 163.6, 187.9, 193.6. LCMS (ES+) *m*/*z* = 710.2 ([M + H]^+^, *t*_R_ = 2.09 min). HRMS (ESI+) *m*/*z* = 710.1889 [M + H]^+^ found, C_40_H_36_N_3_O_6_Fe^+^ required 710.1875.

*(E)-3-((1-(2-(4-(3-(3,4-Dimethoxyphenyl)acryloyl)-2-methoxyphenoxy)ethyl)-1H-1,2,3-triazol-4-yl)methoxy)-2-phenyl-4H-chromen-4-one* (**98**). A mixture of alkyne flavone **30** (806 mg, 2.92 mmol), azide chalcone **67** (1.01 g, 2.64 mmol), CuSO_4_·5H_2_O (716 mg, 2.87 mmol) and sodium ascorbate (1.29 g, 6.51 mmol) in *t*-BuOH/H_2_O (1:1, 40 mL) was reacted according to GP-A. The crude residue was purified by flash column chromatography (SiO_2_, 1%–5% MeOH/CH_2_Cl_2_) to afford triazole hybrid **98** (622 mg, 36%) as a pale yellow-green powdery solid. m.p. 102–104 °C. TLC *R*_f_ = 0.24 (3% MeOH/CH_2_Cl_2_). IR ν_max_ (neat)/cm^−1^: 2940w (C-H str), 2836w (C-H str), 1644m (C=O str), 1596m (C=C str), 1579m (C=C str), 1510s (C=C str), 1467m, 1420w, 1398w, 1260s, 1237s, 1196m, 1146s, 1138s, 1022s. ^1^H-NMR (500 MHz, CDCl_3_): δ 3.87 (3H, s, -OCH_3_), 3.94 (3H, s, -OCH_3_), 3.96 (3H, s, -OCH_3_), 4.38 (2H, t, *J* = 5.2 Hz, -OCH_2_C*H*_2_N-), 4.74 (2H, t, *J* = 5.2 Hz, -OC*H*_2_CH_2_N-), 5.32 (2H, s, -OC*H*_2_CN-), 6.84 (1H, d, *J* = 8.4 Hz, ArH), 6.90 (1H, d, *J* = 8.4 Hz, ArH), 7.16 (1H, d, *J* = 1.6 Hz, ArH), 7.24 (1H, dd, *J* = 8.4, 1.6 Hz, ArH), 7.39 (1H, d, *J* = 15.6 Hz, -C*H*=CHCO-), 7.39–7.44 (4H, m, ArH), 7.50 (1H, d, *J* = 8.4 Hz, ArH), 7.59 (1H, d, *J* = 1.6 Hz, ArH), 7.62 (1H, dd, *J* = 8.4, 2.0 Hz, ArH), 7.69 (1H, t, *J* = 8.4 Hz, ArH), 7.77 (1H, d, *J* = 15.6 Hz, -CH=C*H*CO-), 7.96 (1H, s, -C*H*N-), 7.97–8.00 (2H, m, ArH), 8.27 (1H, dd, *J* = 8.0, 1.6 Hz, ArH). ^13^C-NMR (500 MHz, CDCl_3_): δ 49.4, 56.0, 56.0, 65.1, 67.4, 110.2, 111.1, 111.5, 112.2, 118.1, 119.5, 122.4, 123.0, 124.1, 124.8, 125.3, 125.7, 127.9, 128.3, 128.7, 130.6, 130.7, 132.7, 133.5, 139.4, 143.9, 144.5, 149.2, 149.7, 151.3, 151.3, 155.3, 156.4, 175.0, 188.6. LCMS (ES+) *m*/*z* = 660.2 ([M + H]^+^, *t*_R_ = 1.64 min). HRMS (ESI+) *m*/*z* = 660.2325 [M + H]^+^ found, C_38_H_34_O_8_N_3_^+^ required 660.2340.

*(E)-3-((1-(2-(2-Methoxy-4-(3-(2,3,4-trimethoxyphenyl)acryloyl)phenoxy)ethyl)-1H-1,2,3-triazol-4-yl)methoxy)-2-phenyl-4H-chromen-4-one* (**99**). A mixture of alkyne flavone **30** (205 mg, 0.742 mmol), azide chalcone **68** (306 mg, 0.739 mmol), CuSO_4_·5H_2_O (204 mg, 0.818 mmol) and sodium ascorbate (365 mg, 1.84 mmol) in *t*-BuOH/H_2_O (1:1, 40 mL) was reacted according to GP-A. The crude residue was purified by flash column chromatography (SiO_2_, 1%–5% MeOH/CH_2_Cl_2_) and recrystallized from MeOH to afford triazole hybrid **99** (178 mg, 35%) as an off-white powdery solid. m.p. 120–122 °C. TLC *R*_f_ = 0.28 (5% MeOH/CH_2_Cl_2_). IR ν_max_ (neat)/cm^−1^: 2941w (C-H str), 2838w (C-H str), 1740w, 1645s (C=O str), 1581s (C=C str), 1563m (C=C str), 1516w (C=C str), 1494s, 1466s, 1414m, 1403m, 1264s, 1238m, 1195m, 1148m, 1096s, 1039m, 1027m. ^1^H-NMR (500 MHz, CDCl_3_): δ 3.86 (3H, s, -OCH_3_), 3.90 (3H, s, -OCH_3_), 3.92 (3H, s, -OCH_3_), 3.95 (3H, s, -OCH_3_), 4.36 (2H, t, *J* = 5.2 Hz, -OCH_2_C*H*_2_N-), 4.73 (2H, t, *J* = 5.2 Hz, -OC*H*_2_CH_2_N-), 5.33 (2H, s, -OC*H*_2_CN-), 6.73 (1H, d, *J* = 8.8 Hz, ArH), 6.84 (1H, d, *J* = 8.0 Hz, ArH), 7.38–7.44 (5H, m, ArH), 7.50 (1H, d, *J* = 8.4 Hz, ArH), 7.55 (1H, d, *J* = 15.6 Hz, -C*H*=CHCO-), 7.59–7.62 (2H, m, ArH), 7.69 (1H, t, *J* = 8.4 Hz, ArH), 7.95 (1H, s, -C*H*N-), 7.96–7.99 (2H, m, ArH), 7.99 (1H, d, *J* = 15.6 Hz, -CH=C*H*CO-), 8.28 (1H, dd, *J* = 8.0, 1.2 Hz, ArH). ^13^C-NMR (500 MHz, CDCl_3_): δ 49.4, 55.9, 56.1, 60.9, 61.4, 65.1, 67.4, 107.6, 111.5, 112.2, 118.1, 120.9, 122.0, 122.4, 123.9, 124.1, 124.8, 125.3, 125.7, 128.3, 128.7, 130.6, 130.7, 132.8, 133.5, 139.4, 139.7, 142.5, 143.9, 149.6, 151.2, 153.7, 155.3, 155.7, 156.4, 175.0, 189.0. LCMS (ES+) *m*/*z* = 690.2 ([M + H]^+^, *t*_R_ = 1.95 min). HRMS (ESI+) *m*/*z* = 690.2425 [M + H]^+^ found, C_39_H_36_O_9_N_3_^+^ required 690.2446.

*(E)-3-((1-(2-(2-Methoxy-4-(3-(2,4,6-trimethoxyphenyl)acryloyl)phenoxy)ethyl)-1H-1,2,3-triazol-4-yl)methoxy)-2-phenyl-4H-chromen-4-one* (**100**). A mixture of alkyne flavone **30** (207 mg, 0.749 mmol), azide chalcone **69** (306 mg, 0.741 mmol), CuSO_4_·5H_2_O (203 mg, 0.814 mmol) and sodium ascorbate (369 mg, 1.86 mmol) in *t*-BuOH/H_2_O (1:1, 40 mL) was reacted according to GP-A. The crude residue was purified by flash column chromatography (SiO_2_, 1%–5% MeOH/CH_2_Cl_2_) and recrystallized from MeOH to afford triazole hybrid **100** (342 mg, 67%) as a white powdery solid. m.p. 172–174 °C. TLC *R*_f_ = 0.30 (5% MeOH/CH_2_Cl_2_). IR ν_max_ (neat)/cm^−1^: 2941w (C-H str), 2843w (C-H str), 1737w, 1643s (C=O str), 1599s (C=C str), 1575s (C=C str), 1515m (C=C str), 1465m, 1416m, 1322m, 1261m, 1231s, 1216m, 1192m, 1149s, 1125s, 1027s. ^1^H-NMR (500 MHz, CDCl_3_): δ 3.85 (3H, s, -OCH_3_), 3.88 (3H, s, -OCH_3_), 3.92 (6H, s, 2 × -OCH_3_), 4.35 (2H, t, *J* = 5.2 Hz, -OCH_2_C*H*_2_N-), 4.72 (2H, t, *J* = 5.2 Hz, -OC*H*_2_CH_2_N-), 5.35 (2H, s, -OC*H*_2_CN-), 6.16 (2H, s, ArH), 6.84 (1H, d, *J* = 8.8 Hz, ArH), 7.38–7.44 (4H, m, ArH), 7.50 (1H, d, *J* = 8.4 Hz, ArH), 7.60–7.62 (2H, m, ArH), 7.70 (1H, t, *J* = 8.8 Hz, ArH), 7.87 (1H, d, *J* = 16.0 Hz, -C*H*=CHCO-), 7.94 (1H, s -C*H*N-), 7.95–7.98 (2H, m, ArH), 8.25 (1H, d, *J* = 16.0 Hz, -CH=C*H*CO-), 8.29 (1H, dd, *J* = 8.4, 1.6 Hz, ArH). ^13^C-NMR (500 MHz, CDCl_3_): δ 49.4, 55.4, 55.8, 55.8, 65.0, 67.3, 90.5, 106.5, 111.7, 112.3, 118.1, 121.6, 122.3, 124.1, 124.7, 125.2, 125.7, 128.2, 128.7, 130.6, 130.7, 133.5, 133.5, 135.6, 139.3, 143.8, 149.4, 150.7, 155.2, 156.4, 161.6, 163.0, 175.0, 190.4. LCMS (ES+) *m*/*z* = 690.2 ([M + H]^+^, *t*_R_ = 1.73 min). HRMS (ESI+) *m*/*z* = 690.2476 [M + H]^+^ found, C_39_H_36_O_9_N_3_^+^ required 690.2446.

*(E)-6-((1-(2-(4-(3-(3,4-Dimethoxyphenyl)acryloyl)-2-methoxyphenoxy)ethyl)-1H-1,2,3-triazol-4-yl)methoxy)-2-phenyl-4H-chromen-4-one* (**101**). A mixture of alkyne flavone **31** (179 mg, 0.648 mmol), azide chalcone **67** (246 mg, 0.641 mmol), CuSO_4_·5H_2_O (188 mg, 0.753 mmol) and sodium ascorbate (322 mg, 1.63 mmol) in *t*-BuOH/H_2_O (1:1, 40 mL) was reacted according to GP-A. The crude residue was purified by flash column chromatography (SiO_2_, 1%–5% MeOH/CH_2_Cl_2_) and recrystallized from MeOH to afford triazole hybrid **101** (366 mg, 87%) as a bright orange crystalline solid. m.p. 128–130 °C. TLC *R*_f_ = 0.34 (5% MeOH/CH_2_Cl_2_). IR ν_max_ (neat)/cm^−1^: 2940w (C-H str), 2831w (C-H str), 1637s (C=O str), 1618m, 1595s (C=C str), 1571s (C=C str), 1510s (C=C str), 1481m, 1454s, 1419m, 1360s, 1257s, 1235m, 1198m, 1140s, 1023s. ^1^H-NMR (500 MHz, CDCl_3_): δ 3.92 (3H, s, -OCH_3_), 3.93 (3H, s, -OCH_3_), 3.94 (3H, s, -OCH_3_), 4.47 (2H, t, *J* = 4.8 Hz, -OCH_2_C*H*_2_N-), 4.86 (2H, t, *J* = 4.8 Hz, -OC*H*_2_CH_2_N-), 5.29 (2H, s, -OC*H*_2_CN-), 6.79 (1H, s, -C=CH), 6.85 (1H, d, *J* = 8.8 Hz, ArH), 6.89 (1H, d, *J* = 8.4 Hz, ArH), 7.14 (1H, d, *J* = 1.6 Hz, ArH), 7.22 (1H, dd, *J* = 8.4, 2.0 Hz, ArH), 7.33 (1H, dd, *J* = 9.2, 3.2 Hz, ArH), 7.36 (1H, d, *J* = 15.2 Hz, -C*H*=CHCO-), 7.49–7.52 (4H, m, ArH), 7.59–7.61 (2H, m, ArH), 7.71 (1H, d, *J* = 3.2 Hz, ArH), 7.73 (1H, d, *J* = 16.0 Hz, -CH=C*H*CO-), 7.88–7.91 (2H, m, ArH), 8.04 (1H, s, -C*H*N-). ^13^C-NMR (500 MHz, CDCl_3_): δ 49.7, 55.9, 56.0, 62.3, 67.5, 106.3, 106.7, 110.1, 111.1, 111.5, 112.3, 119.4, 119.7, 122.5, 123.0, 123.9, 124.5, 124.6, 126.2, 127.8, 129.0, 131.5, 131.7, 132.7, 143.3, 144.5, 149.2, 149.7, 151.2, 151.2, 151.3, 155.5, 163.2, 178.1, 188.5. LCMS (ES+) *m*/*z* = 660.2 ([M + H]^+^, *t*_R_ = 1.81 min). HRMS (ESI+) *m*/*z* = 682.2153 [M + Na]^+^ found, C_38_H_33_O_8_N_3_Na^+^ required 682.2160.

*(E)-6-((1-(2-(2-Methoxy-4-(3-(2,4,6-trimethoxyphenyl)acryloyl)phenoxy)ethyl)-1H-1,2,3-triazol-4-yl)methoxy)-2-phenyl-4H-chromen-4-one* (**102**). A mixture of alkyne flavone **31** (211 mg, 0.763 mmol), azide chalcone **69** (317 mg, 0.767 mmol), CuSO_4_·5H_2_O (211 mg, 0.843 mmol) and sodium ascorbate (382 mg, 1.93 mmol) in *t*-BuOH/H_2_O (1:1, 40 mL) was reacted according to GP-A. The crude residue was purified by flash column chromatography (SiO_2_, 1%–5% MeOH/CH_2_Cl_2_) and recrystallized from MeOH to afford triazole hybrid **102** (397 mg, 75%) as a bright yellow-orange flaky solid. m.p. 128–130 °C. TLC *R*_f_ = 0.25 (5% MeOH/CH_2_Cl_2_). IR ν_max_ (neat)/cm^−1^: 2941w (C-H str), 2838w (C-H str), 1739w, 1640s (C=O str), 1598s (C=C str), 1569s (C=C str), 1517m (C=C str), 1455s, 1363m, 1320m, 1279m, 1261m, 1201s, 1154s, 1122s, 1035s, 1025s. ^1^H-NMR (500 MHz, CDCl_3_): δ 3.84 (3H, s, -OCH_3_), 3.89 (6H, s, 2 × -OCH_3_), 3.92 (3H, s, -OCH_3_), 4.45 (2H, t, *J* = 4.8 Hz, -OCH_2_C*H*_2_N-), 4.85 (2H, t, *J* = 4.8 Hz, -OC*H*_2_CH_2_N-), 5.28 (2H, s, -OC*H*_2_CN-), 6.12 (2H, s, ArH), 6.79 (1H, s, -C=CH), 6.84 (1H, d, *J* = 8.4 Hz, ArH), 7.33 (1H, dd, *J* = 9.2, 3.2 Hz, ArH), 7.48–7.51 (4H, m, ArH), 7.57 (1H, dd, *J* = 8.0, 1.6 Hz, ArH), 7.61 (1H, d, *J* = 1.6 Hz, ArH), 7.71 (1H, d, *J* = 2.8 Hz, ArH), 7.84 (1H, d, *J* = 16.0 Hz, -C*H*=CHCO-), 7.88–7.91 (2H, m, ArH), 8.06 (1H, s, -C*H*N-), 8.21 (1H, d, *J* = 15.6 Hz, -CH=C*H*CO-). ^13^C-NMR (500 MHz, CDCl_3_): δ 49.7, 55.3, 55.7, 55.9, 62.3, 67.5, 90.5, 106.2, 106.5, 106.7, 111.6, 112.4, 119.6, 121.4, 122.3, 123.9, 124.4, 124.6, 126.2, 128.9, 131.5, 131.7, 133.6, 135.5, 143.3, 149.4, 150.6, 151.2, 155.6, 161.6, 163.0, 163.2, 178.1, 190.3. LCMS (ES+) *m*/*z* = 690.3 ([M + H]^+^, *t*_R_ = 1.88 min). HRMS (ESI+) *m*/*z* = 690.2426 [M + H]^+^ found, C_39_H_36_O_9_N_3_^+^ required 690.2446.

*(E)-6-((1-(2-(2-Methoxy-4-(3-(2,3,4-trimethoxyphenyl)acryloyl)phenoxy)ethyl)-1H-1,2,3-triazol-4-yl)-methoxy)-2-phenyl-4H-chromen-4-one* (**103**). A mixture of alkyne flavone **31** (201 mg, 0.729 mmol), azide chalcone **68** (304 mg, 0.735 mmol), CuSO_4_·5H_2_O (208 mg, 0.831 mmol) and sodium ascorbate (383 mg, 1.93 mmol) in *t*-BuOH/H_2_O (1:1, 40 mL) was reacted according to GP-A. The crude residue was purified by flash column chromatography (SiO_2_, 1%–5% MeOH/CH_2_Cl_2_) and recrystallized from MeOH to afford triazole hybrid **103** (382 mg, 76%) as a pale yellow flaky solid. m.p. 138–140 °C. TLC *R*_f_ = 0.32 (5% MeOH/CH_2_Cl_2_). IR ν_max_ (neat)/cm^−1^: 2939w (C-H str), 2839w (C-H str), 1740w, 1640s (C=O str), 1619m, 1593m (C=C str), 1573s (C=C str), 1516m (C=C str), 1495m, 1482m, 1455s, 1415m, 1360s, 1259s, 1200m, 1157m, 1095s, 1026s. ^1^H-NMR (500 MHz, CDCl_3_): δ 3.89 (3H, s, -OCH_3_), 3.90 (3H, s, -OCH_3_), 3.93 (3H, s, -OCH_3_), 3.94 (3H, s, -OCH_3_), 4.47 (2H, t, *J* = 4.8 Hz, -OCH_2_C*H*_2_N-), 4.86 (2H, t, *J* = 4.8 Hz, -OC*H*_2_CH_2_N-), 5.29 (2H, s, -OC*H*_2_CN-), 6.72 (1H, d, *J* = 8.8 Hz, ArH), 6.80 (1H, s, -C=CH), 6.86 (1H, d, *J* = 8.0 Hz, ArH), 7.34 (1H, dd, *J* = 9.2, 3.2 Hz, ArH), 7.37 (1H, d, *J* = 8.8 Hz, ArH), 7.49–7.52 (4H, m, ArH), 7.53 (1H, d, *J* = 16.0 Hz, -C*H*=CHCO-), 7.58–7.61 (2H, m, ArH), 7.71 (1H, d, *J* = 3.2 Hz, ArH), 7.89–7.92 (2H, m, ArH), 7.96 (1H, d, *J* = 16.0 Hz, -CH=C*H*CO-), 8.05 (1H, s, -C*H*N-). ^13^C-NMR (500 MHz, CDCl_3_): δ 49.7, 56.0, 56.0, 60.9, 61.3, 62.3, 67.5, 106.3, 106.8, 107.5, 111.5, 112.3, 119.7, 120.8, 122.0, 122.4, 123.8, 123.9, 124.5, 124.6, 126.2, 129.0, 131.5, 131.7, 132.8, 139.6, 142.4, 143.3, 149.6, 151.1, 151.2, 153.7, 155.6, 155.7, 163.2, 178.1, 188.9. LCMS (ES+) *m*/*z* = 690.2 ([M + H]^+^, *t*_R_ = 1.94 min). HRMS (ESI+) *m*/*z* = 690.2452 [M + H]^+^ found, C_39_H_36_N_3_O_9_^+^ required 690.2452.

*(E)-7-((1-(2-(2-Methoxy-4-(3-(2,4,6-trimethoxyphenyl)acryloyl)phenoxy)ethyl)-1H-1,2,3-triazol-4-yl)-methoxy)-2-phenyl-4H-chromen-4-one* (**104**). A mixture of alkyne flavone **32** (202 mg, 0.730 mmol), azide chalcone **69** (304 mg, 0.735 mmol), CuSO_4_·5H_2_O (218 mg, 0.872 mmol) and sodium ascorbate (386 mg, 1.95 mmol) in *t*-BuOH/H_2_O (1:1, 40 mL) was reacted according to GP-A. The crude residue was purified by flash column chromatography (SiO_2_, 1%–5% MeOH/CH_2_Cl_2_) and recrystallized from MeOH to afford triazole hybrid **104** (324 mg, 64%) as a pale yellow-white powdery solid. m.p. 168–170 °C. TLC *R*_f_ = 0.25 (5% MeOH/CH_2_Cl_2_). IR ν_max_ (neat)/cm^−1^: 2941w (C-H str), 2841w (C-H str), 1736w, 1634s (C=O str), 1599s (C=C str), 1566s (C=C str), 1512m (C=C str), 1451m, 1441m, 1336s, 1302w, 1279m, 1261m, 1204m, 1175m, 1160w, 1122s, 1092w, 1053w, 1037m, 1016w. ^1^H-NMR (500 MHz, CDCl_3_): δ 3.87 (3H, s, -OCH_3_), 3.91 (6H, s, 2 × -OCH_3_), 3.93 (3H, s, -OCH_3_), 4.48 (2H, t, *J* = 5.2 Hz, -OCH_2_C*H*_2_N-), 4.87 (2H, t, *J* = 4.8 Hz, -OC*H*_2_CH_2_N-), 5.35 (2H, s, -OC*H*_2_CN-), 6.14 (2H, s, ArH), 6.77 (1H, s, -C=CH), 6.86 (1H, d, *J* = 8.0 Hz, ArH), 7.06 (1H, dd, *J* = 8.8, 2.0 Hz, ArH), 7.14 (1H, d, *J* = 2.0 Hz, ArH), 7.49–7.53 (3H, m, ArH), 7.59 (1H, dd, *J* = 8.4, 2.0 Hz, ArH), 7.63 (1H, d, *J* = 1.6 Hz, ArH), 7.84 (1H, d, *J* = 16.0 Hz, -C*H*=CHCO-), 7.90–7.92 (2H, m, ArH), 8.07 (1H, s, -C*H*N-), 8.15 (1H, d, *J* = 8.8 Hz, ArH), 8.23 (1H, d, *J* = 16.0 Hz, -CH=C*H*CO-). ^13^C-NMR (500 MHz, CDCl_3_): δ 49.8, 55.3, 55.7, 55.8, 62.3, 67.4, 90.4, 101.4, 106.4, 107.3, 111.6, 112.4, 114.8, 118.0, 121.3, 122.3, 124.7, 126.1, 127.0, 128.9, 131.4, 131.6, 133.6, 135.6, 143.0, 149.4, 150.5, 157.8, 161.6, 162.6, 163.0, 163.1, 177.7, 190.2. LCMS (ES+) *m*/*z* = 690.2 ([M + H]^+^, *t*_R_ = 1.70 min). HRMS (ESI+) *m*/*z* = 690.2471 [M + H]^+^ found, C_39_H_36_O_9_N_3_^+^ required 690.2446.

*(E)-2-(4-(2-(4-((2-(3-(Ferrocenyl)acryloyl)phenoxy)methyl)-1H-1,2,3-triazol-1-yl)ethoxy)phenyl)-3-hydroxy-7-methoxy-4H-chromen-4-one* (**105**). A mixture of alkyne chalcone **26** (310 mg, 0.838 mmol), azide flavonol **80** (296 mg, 0.838 mmol), CuSO_4_·5H_2_O (273 mg, 1.09 mmol) and sodium ascorbate (496 mg, 2.50 mmol) in *t*-BuOH/H_2_O (1:1, 40 mL) was reacted according to GP-A. The crude residue was purified by flash column chromatography (SiO_2_, 1%–5% MeOH/CH_2_Cl_2_) to afford triazole hybrid **105** (238 mg, 40%) as a dark red powdery solid. m.p. 118–120 °C. TLC *R*_f_ = 0.44 (5% MeOH/CH_2_Cl_2_). IR ν_max_ (neat)/cm^−1^: 3340w (O-H str), 3088w (C-H str), 2929w (C-H str), 1735m (C=O str), 1597s (C=C str), 1541m (C=C str), 1506m (C=C str), 1484m, 1449m, 1403m, 1235s, 1206s, 1171s, 1117m, 1106m, 1044w, 1025s. ^1^H-NMR (500 MHz, CDCl_3_): δ 3.96 (3H, s, -OCH_3_), 4.12 (5H, s, -C_5_H_5_), 4.35 (2H, t, *J* = 4.5 Hz, -OCH_2_C*H*_2_N-), 4.43 (2H, t, *J* = 1.5 Hz, -C_5_H_4_), 4.48 (2H, t, *J* = 1.5 Hz, -C_5_H_4_), 4.67 (2H, t, *J* = 5.0 Hz, -OC*H*_2_CH_2_N-), 5.33 (2H, br s, -OC*H*_2_CN-), 6.95–6.97 (4H, m, ArH and OH), 6.96 (1H, d, *J* = 15.5 Hz, -C*H*=CHCO-), 7.01 (1H, dd, *J* = 9.0, 2.0 Hz, ArH), 7.09 (1H, t, *J* = 7.5 Hz, ArH), 7.14 (1H, d, *J* = 8.5 Hz, ArH), 7.46 (1H, dd, *J* = 7.5, 1.0 Hz, ArH), 7.50 (1H, d, *J* = 16.0 Hz, -CH=C*H*CO-), 7.60 (1H, dd, *J* = 7.5, 1.5 Hz, ArH), 7.77 (1H, br s, -C*H*N-), 8.14 (1H, d, *J* = 9.0 Hz, ArH), 8.19 (2H, d, *J* = 8.5 Hz, ArH). ^13^C-NMR (500 MHz, CDCl_3_): δ 49.6, 55.8, 63.0, 66.1, 68.9, 69.8, 71.3, 79.0, 99.9, 113.1, 114.5, 114.6, 114.7, 121.4, 124.0, 124.7, 126.7, 129.3, 130.1, 130.2, 132.4, 137.4, 144.1, 145.5, 156.3, 157.2, 158.8, 164.2, 172.6, 192.3. LCMS (ES+) *m*/*z* = 724.2 ([M + H]^+^, *t*_R_ = 2.01 min). HRMS (ESI+) *m*/*z* = 724.1711 [M + H]^+^ found, C_40_H_34_N_3_O_7_Fe^+^ required 724.1746.

*(E)-2-(4-(2-(4-((4-(3-(Ferrocenyl)acryloyl)-2-methoxyphenoxy)methyl)-1H-1,2,3-triazol-1-yl)ethoxy)phenyl)-3-hydroxy-4H-chromen-4-one* (**106**). A mixture of alkyne chalcone **25** (308 mg, 0.769 mmol), azide flavonol **79** (252 mg, 0.779 mmol), CuSO_4_·5H_2_O (209 mg, 0.835 mmol) and sodium ascorbate (395 mg, 1.99 mmol) in *t*-BuOH/H_2_O (1:1, 40 mL) was reacted according to GP-A. The crude residue was purified by flash column chromatography (SiO_2_, 1%–5% MeOH/CH_2_Cl_2_) to afford triazole hybrid **106** (212 mg, 38%) as a dark-red powdery solid. m.p. 158–160 °C. TLC *R*_f_ = 0.26 (3% MeOH/CH_2_Cl_2_). IR ν_max_ (neat)/cm^−1^: 3429w (O-H str), 2943w (C-H str), 2873w (C-H str), 1735m (C=O str), 1645w, 1596s (C=C str), 1568s (C=C str), 1509s (C=C str), 1469m, 1418m, 1409m, 1348w, 1292w, 1254s, 1200w, 1180m, 1151m, 1108m, 1027m. ^1^H-NMR (500 MHz, CDCl_3_): δ 3.97 (3H, s, -OCH_3_), 4.17 (5H, s, -C_5_H_5_), 4.46–4.48 (4H, m, -C_5_H_4_ and -OCH_2_C*H*_2_N-), 4.57 (2H, t, *J* = 2.0 Hz, -C_5_H_4_), 4.83 (2H, t, *J* = 5.0 Hz, -OC*H*_2_CH_2_N-), 5.43 (2H, s, -OC*H*_2_CN-), 6.94 (1H, br s, OH), 6.98 (2H, d, *J* = 9.0 Hz, ArH), 7.12 (1H, d, *J* = 15.0 Hz, -C*H*=CHCO-), 7.15 (1H, d, *J* = 8.5 Hz, ArH), 7.43 (1H, t, *J* = 8.0 Hz, ArH), 7.57 (1H, dd, *J* = 8.0, 1.5 Hz, ArH), 7.59 (1H, d, *J* = 8.5 Hz, ArH), 7.63 (1H, d, *J* = 2.0 Hz, ArH), 7.71 (1H, t, *J* = 8.0 Hz, ArH), 7.73 (1H, d, *J* = 15.5 Hz, -CH=C*H*CO-), 7.90 (1H, br s, -C*H*N-), 8.22 (2H, d, *J* = 9.0 Hz, ArH), 8.25 (1H, dd, *J* = 8.0, 1.5 Hz, ArH). ^13^C-NMR (500 MHz, CDCl_3_): δ 49.7, 56.1, 62.9, 66.3, 68.9, 69.7, 71.2, 79.3, 111.2, 112.3, 114.5, 118.2, 118.5, 120.7, 122.4, 124.3, 124.5, 124.6, 125.4, 129.6, 132.3, 133.5, 137.8, 143.8, 144.7, 146.0, 149.5, 151.3, 155.2, 159.0, 173.2, 187.9. LCMS (ES+) *m*/*z* = 724.2 ([M + H]^+^, *t*_R_ = 2.04 min). HRMS (ESI+) *m*/*z* = 724.1723 [M + H]^+^ found, C_40_H_34_N_3_O_7_Fe^+^ required 724.1746.

*(E)-2-(3-(2-(4-((4-Bromo-2-(3-(1-methyl-1H-indol-3-yl)acryloyl)phenoxy)methyl)-1H-1,2,3-triazol-1-yl)-ethoxy)-4-methoxyphenyl)-3-hydroxy-4H-chromen-4-one* (**107**). A mixture of alkyne chalcone **21** (339 mg, 0.859 mmol), azide flavonol **82** (301 mg, 0.853 mmol), CuSO_4_·5H_2_O (542 mg, 2.17 mmol) and sodium ascorbate (202 mg, 1.02 mmol) in *t*-BuOH/H_2_O (1:1, 40 mL) was reacted according to GP-A. The crude residue was purified by flash column chromatography (SiO_2_, 1%–5% MeOH/CH_2_Cl_2_) and recrystallized from MeOH to afford triazole hybrid **107** (258 mg, 40%) as a brown powdery solid. m.p. 158–160 °C. TLC *R*_f_ = 0.44 (5% MeOH/CH_2_Cl_2_). IR ν_max_ (neat)/cm^−1^: 3351w (O-H str), 2920m (C-H str), 2852w (C-H str), 1640m (C=O str), 1589s (C=C str), 1559m (C=C str), 1514m (C=C str), 1492m, 1471m, 1397m, 1374m, 1333w, 1269s, 1204m, 1180m, 1130s, 1074w, 1047m, 1020m. ^1^H-NMR (500 MHz, CDCl_3_): δ 3.64 (3H, s, -NCH_3_), 3.83 (3H, s, -OCH_3_), 4.02 (2H, t, *J* = 4.5 Hz, -OCH_2_C*H*_2_N-), 4.49 (2H, t, *J* = 4.5 Hz, -OC*H*_2_CH_2_N-), 5.36 (2H, br s, -OC*H*_2_CN-), 6.96 (1H, d, *J* = 9.0 Hz, ArH), 7.02 (1H, br s, OH), 7.07 (1H, d, *J* = 8.5 Hz, ArH), 7.10–7.13 (1H, m, ArH), 7.18 (1H, s, ArH), 7.23 (2H, d, *J* = 3.5 Hz, ArH), 7.38 (1H, d, *J* = 15.5 Hz, -C*H*=CHCO-), 7.45 (1H, t, *J* = 7.5 Hz, ArH), 7.50 (1H, d, *J* = 1.5 Hz, ArH), 7.53 (1H, d, *J* = 7.5 Hz, ArH), 7.56 (1H, dd, *J* = 8.5, 2.5 Hz, ArH), 7.60 (1H, d, *J* = 8.5 Hz, ArH), 7.73 (1H, t, *J* = 7.0 Hz, ArH), 7.77 (1H, d, *J* = 16.0 Hz, -CH=C*H*CO-), 7.83 (1H, d, *J* = 2.5 Hz, ArH), 7.90 (1H, dd, *J* = 8.5, 1.5 Hz, ArH), 8.06 (1H, br s, -C*H*N-), 8.27 (1H, d, *J* = 8.0 Hz, ArH). ^13^C-NMR (500 MHz, CDCl_3_): δ 33.0, 49.5, 55.9, 63.5, 67.1, 109.9, 111.4, 112.6, 113.2, 114.0, 114.8, 118.1, 120.5, 120.6, 121.4, 121.6, 122.6, 123.0, 123.3, 123.7, 124.6, 124.8, 125.4, 125.7, 131.9, 133.3, 133.6, 134.8, 134.9, 137.7, 137.8, 138.0, 144.5, 146.9, 151.3, 155.1, 155.8, 173.0, 190.5. LCMS (ES+) *m*/*z* = 749.2 ([M + H]^+^, *t*_R_ = 1.99 min). HRMS (ESI+) *m*/*z* = 769.1232 [M + Na]^+^ found, C_39_H_31_O_7_N_4_BrNa^+^ required 769.1268.

*(E)-2-(4-(2-(4-((4-Bromo-2-(3-(furan-2-yl)acryloyl)phenoxy)methyl)-1H-1,2,3-triazol-1-yl)ethoxy)-3-methoxyphenyl)-3-hydroxy-4H-chromen-4-one* (**108**). A mixture of alkyne chalcone **24** (289 mg, 0.872 mmol), azide flavonol **81** (304 mg, 0.860 mmol), CuSO_4_·5H_2_O (234 mg, 0.936 mmol) and sodium ascorbate (476 mg, 2.40 mmol) in *t*-BuOH/H_2_O (1:1, 40 mL) was reacted according to GP-A. The crude residue was purified by flash column chromatography (SiO_2_, 1%–5% MeOH/CH_2_Cl_2_) and recrystallized from MeOH to afford triazole hybrid **108** (245 mg, 42%) as a dark yellow-brown powdery solid. m.p. 118–120 °C. TLC *R*_f_ = 0.41 (5% MeOH/CH_2_Cl_2_). IR ν_max_ (neat)/cm^−1^: 3275w (O-H str), 3110w (C-H str), 2926w (C-H str), 1650m (C=O str), 1598s (C=C str), 1549m (C=C str), 1515s (C=C str), 1481s, 1422w, 1398m, 1267s, 1232m, 1207s, 1177m, 1145s, 1111m, 1041m, 1017m, 1008m. ^1^H-NMR (500 MHz, CDCl_3_): δ 3.90 (3H, s, -OCH_3_), 4.43 (2H, t, *J* = 4.0 Hz, -OCH_2_C*H*_2_N-), 4.75 (2H, t, *J* = 4.0 Hz, -OC*H*_2_CH_2_N-), 5.33 (2H, br s, -OC*H*_2_CN-), 6.47 (1H, d, *J* = 1.5 Hz, ArH), 6.64 (1H, d, *J* = 3.0 Hz, ArH), 6.89 (1H, d, *J* = 8.5 Hz, ArH), 7.01 (1H, s, ArH), 7.06–7.08 (1H, m, ArH), 7.25 (1H, d, *J* = 16.0 Hz, -C*H*=CHCO-, overlain by CDCl_3_), 7.36 (1H, d, *J* = 15.5 Hz, -CH=C*H*CO-), 7.44 (1H, t, *J* = 7.5 Hz, ArH), 7.52 (1H, s, ArH), 7.54 (1H, d, *J* = 6.5 Hz, ArH), 7.61 (1H, d, *J* = 8.5 Hz, ArH), 7.72 (1H, d, *J* = 7.0 Hz, ArH), 7.74 (1H, d, *J* = 2.0 Hz, ArH), 7.82 (1H, d, *J* = 8.5 Hz, ArH), 7.84 (1H, s, -C*H*N-), 8.09 (1H, br s, OH), 8.26 (1H, d, *J* = 7.5 Hz, ArH). ^13^C-NMR (500 MHz, CDCl_3_): δ 49.9, 55.8, 63.2, 67.5, 111.4, 112.7, 113.6, 113.9, 115.1, 116.1, 118.2, 120.6, 121.1, 124.0, 124.5, 125.2, 125.4, 129.6, 131.1, 133.0, 133.5, 135.4, 137.9, 144.6, 145.0, 148.7, 149.4, 151.4, 155.2, 155.7, 173.1, 190.2. LCMS (ES+) *m*/*z* = 686.2 ([M + H]^+^, *t*_R_ = 2.00 min). HRMS (ESI+) *m*/*z* = 684.0955 [M + H]^+^ found, C_34_H_27_O_8_N_3_Br^+^ required 684.0976.

*6-(2-(4-(((4-Oxo-2-phenyl-4H-chromen-3-yl)oxy)methyl)-1H-1,2,3-triazol-1-yl)ethoxy)-2-phenyl-4H-chromen-4-one* (**109**). A mixture of alkyne flavone **30** (277 mg, 1.00 mmol), azide flavone **85** (307 mg, 0.998 mmol), CuSO_4_·5H_2_O (303 mg, 1.21 mmol) and sodium ascorbate (492 mg, 2.48 mmol) in *t*-BuOH/H_2_O (1:1, 40 mL) was reacted according to GP-A. The crude residue was purified by flash column chromatography (SiO_2_, 1%–5% MeOH/CH_2_Cl_2_) to afford triazole hybrid **109** (534 mg, 92%) as a white powdery solid. m.p. 234–236 °C. TLC *R*_f_ = 0.36 (5% MeOH/CH_2_Cl_2_). IR ν_max_ (neat)/cm^−1^: 3145w (C-H str), 2940w (C-H str), 1736w, 1643s (C=O str), 1627s, 1600m, 1570m (C=C str), 1561m (C=C str), 1482m, 1470m, 1455s, 1398m, 1361s, 1293m, 1197s, 1187m, 1148m, 1087m, 1047m, 1026w. ^1^H-NMR (500 MHz, CDCl_3_): δ 4.41 (2H, t, *J* = 4.8 Hz, -OCH_2_C*H*_2_N-), 4.73 (2H, t, *J* = 4.8 Hz, -OC*H*_2_CH_2_N-), 5.34 (2H, s, -OC*H*_2_CN-), 6.83 (1H, s, -C=CH), 7.25 (1H, dd, *J* = 9.2, 3.2 Hz, ArH, overlain by CDCl_3_), 7.41–7.46 (4H, m, ArH), 7.51–7.55 (5H, m, ArH), 7.58 (1H, d, *J* = 3.2 Hz, ArH), 7.70 (1H, t, *J* = 7.2 Hz, ArH), 7.81 (1H, s, -C*H*N-), 7.92–7.94 (2H, m, ArH), 8.01–8.04 (2H, m, ArH), 8.29 (1H, d, *J* = 7.2 Hz, ArH). ^13^C-NMR (500 MHz, CDCl_3_): δ 49.4, 65.1, 66.7, 105.9, 106.9, 118.0, 119.9, 123.8, 124.1, 124.5, 124.8, 125.7, 126.2, 128.3, 128.7, 129.0, 130.7, 131.6, 131.7, 133.5, 139.5, 144.0, 151.4, 155.1, 155.3, 156.4, 163.3, 175.1, 178.0. LCMS (ES+) *m*/*z* = 584.1 ([M + H]^+^, *t*_R_ = 1.91 min). HRMS (ESI+) *m*/*z* = 606.1610 [M + Na]^+^ found, C_35_H_25_O_6_N_3_Na^+^ required 606.1636.

*3-Hydroxy-2-(4-(2-(4-(((4-oxo-2-phenyl-4H-chromen-3-yl)oxy)methyl)-1H-1,2,3-triazol-1-yl)ethoxy)phenyl) -4H-chromen-4-one* (**110**). A mixture of alkyne flavone **30** (272 mg, 0.984 mmol), azide flavonol **79** (311 mg, 0.961 mmol), CuSO_4_·5H_2_O (274 mg, 1.10 mmol) and sodium ascorbate (557 mg, 2.81 mmol) in *t*-BuOH/H_2_O (1:1, 40 mL) was reacted according to GP-A. The crude residue was purified by flash column chromatography (SiO_2_, 1%–5% MeOH/CH_2_Cl_2_) and recrystallized from MeOH to afford triazole hybrid **110** (272 mg, 47%) as an off-white powdery solid. m.p. 148–150 °C. TLC *R*_f_ = 0.34 (5% MeOH/CH_2_Cl_2_). IR ν_max_ (neat)/cm^−1^: 3254w(br) (O-H str), 2964w (C-H str), 1602s (C=O str), 1564m (C=C str), 1549m (C=C str), 1509s (C=C str), 1481m, 1469s, 1427m, 1403s, 1282m, 1250s, 1198m, 1183s, 1150w, 1116m, 1109m, 1041m. ^1^H-NMR (500 MHz, CDCl_3_): δ 4.39 (2H, t, *J* = 5.5 Hz, -OCH_2_C*H*_2_N-), 4.73 (2H, t, *J* = 5.5 Hz, -OC*H*_2_CH_2_N-), 5.33 (2H, s, -OC*H*_2_CN-), 6.99 (3H, d, *J* = 9.0 Hz, ArH and OH), 7.41–7.46 (5H, m, ArH), 7.53 (1H, dd, *J* = 8.0, 0.5 Hz, ArH), 7.59 (1H, d, *J* = 8.0 Hz, ArH), 7.68–7.72 (2H, m, ArH), 7.85 (1H, s, -C*H*N-), 8.01–8.03 (2H, m, ArH), 8.23 (2H, d, *J* = 9.0 Hz, ArH), 8.25 (1H, dd, *J* = 8.0, 1.5 Hz, ArH), 8.29 (1H, dd, *J* = 8.0, 1.5 Hz, ArH). ^13^C-NMR (500 MHz, CDCl_3_): δ 49.5, 65.1, 66.2, 114.5, 118.1, 118.2, 120.7, 124.1, 124.5, 124.5, 124.8, 125.0, 125.4, 125.7, 128.4, 128.7, 129.6, 130.7, 133.5, 133.6, 137.8, 139.5, 144.1, 144.8, 155.3, 155.3, 156.4, 159.1, 173.2, 175.1. LCMS (ES+) *m*/*z* = 600.0 ([M + H]^+^, *t*_R_ = 1.75 min). HRMS (ESI+) *m*/*z* = 600.1755 [M + H]^+^ found, C_35_H_26_O_7_N_3_^+^ required 600.1765.

*3-Hydroxy-2-(4-((1-(2-(4-(3-hydroxy-4-oxo-4H-chromen-2-yl)phenoxy)ethyl)-1H-1,2,3-triazol-4-yl)-methoxy)phenyl)-4H-chromen-4-one* (**111**). A mixture of alkyne flavonol **36** (273 mg, 0.933 mmol), azide flavonol **79** (310 mg, 0.958 mmol), CuSO_4_·5H_2_O (296 mg, 1.19 mmol) and sodium ascorbate (461 mg, 2.33 mmol) in *t*-BuOH/H_2_O (1:1, 40 mL) was reacted according to GP-A. The crude residue was purified by flash column chromatography (SiO_2_, 1%–5% MeOH/CH_2_Cl_2_) and recrystallized from MeOH to afford triazole hybrid **111** (82.7 mg, 14%) as a dark brown powdery solid. m.p. 218–220 °C. TLC *R*_f_ = 0.41 (5% MeOH/CH_2_Cl_2_). IR ν_max_ (neat)/cm^−1^: 3284w (O-H str), 3087w (C-H str), 2924w (C-H str), 1600s (C=O str), 1563m (C=C str), 1543m (C=C str), 1508s (C=C str), 1491s, 1424s, 1409s, 1248s, 1209m, 1180s, 1108s, 1043m, 1014w. ^1^H-NMR (500 MHz, DMSO-*d*_6_): δ 4.53 (2H, t, *J* = 4.5 Hz, -OCH_2_C*H*_2_N-), 4.85 (2H, t, *J* = 4.5 Hz, -OC*H*_2_CH_2_N-), 5.29 (2H, s, -OC*H*_2_CN-), 7.11 (2H, d, *J* = 8.5 Hz, ArH), 7.25 (2H, d, *J* = 8.5 Hz, ArH), 7.43–7.47 (2H, m, ArH), 7.71–7.80 (4H, m, ArH), 8.09 (2H, t, *J* = 6.5 Hz, ArH), 8.17 (2H, d, *J* = 9.0 Hz, ArH), 8.21 (2H, d, *J* = 9.0 Hz, ArH), 8.39 (1H, s, -C*H*N-), 9.47 (1H, s, OH), 9.48 (1H, s, OH). ^13^C-NMR (500 MHz, DMSO-*d*_6_): δ 49.0, 61.1, 66.3, 114.6, 114.8, 115.4, 118.3, 121.3, 123.9, 124.1, 124.5, 124.7, 125.4, 129.4, 129.6, 133.4, 133.5, 138.2, 142.4, 145.3, 145.4, 154.4, 158.9, 159.1, 172.6. LCMS (ES+) *m*/*z* = 616.1 ([M + H]^+^, *t*_R_ = 1.68 min). HRMS (ESI+) *m*/*z* = 616.1722 [M + H]^+^ found, C_35_H_26_N_3_O_8_^+^ required 616.1720.

*3-(4-Methoxyphenyl)-7-((1-(2-((4-oxo-2-phenyl-4H-chromen-6-yl)oxy)ethyl)-1H-1,2,3-triazol-4-yl)-methoxy)-4H-chromen-4-one* (**112**). A mixture of alkyne isoflavone **46** (280 mg, 0.914 mmol), azide flavone **85** (282 mg, 0.919 mmol), CuSO_4_·5H_2_O (279 mg, 1.12 mmol) and sodium ascorbate (503 mg, 2.54 mmol) in *t*-BuOH/H_2_O (1:1, 40 mL) was reacted according to GP-A. The crude residue was purified by flash column chromatography (SiO_2_, 1%–5% MeOH/CH_2_Cl_2_) and recrystallized from MeOH to afford triazole hybrid **112** (531 mg, 95%) as a white powdery solid. m.p. 234–236 °C. TLC *R*_f_ = (3% MeOH/CH_2_Cl_2_). IR ν_max_ (neat)/cm^−1^: 3082w (C-H str), 2941w (C-H str), 1641s, 1625s (C=O str), 1608s, 1567s (C=C str), 1515s (C=C str), 1497w, 1483w, 1456s, 1443s, 1359s, 1292s, 1252s, 1204m, 1192m, 1185s, 1137w, 1099m, 1084m, 1047s, 1032s. ^1^H-NMR (500 MHz, CDCl_3_): δ 3.83 (3H, s, -OCH_3_), 4.50 (2H, t, *J* = 4.8 Hz, -OCH_2_C*H*_2_N-), 4.86 (2H, t, *J* = 4.8 Hz, -OC*H*_2_CH_2_N-), 5.36 (2H, s, -OC*H*_2_CN-), 6.82 (1H, s, -C=CH), 6.95 (2H, d, *J* = 8.4 Hz, ArH), 7.01 (1H, d, *J* = 2.0 Hz, ArH), 7.07 (1H, dd, *J* = 8.8, 2.0 Hz, ArH), 7.18 (1H, dd, *J* = 8.8, 3.2 Hz, ArH), 7.48–7.56 (6H, m, ArH), 7.59 (1H, d, *J* = 3.2 Hz, ArH), 7.86 (1H, s, -C*H*N-), 7.90–7.92 (3H, m, -C=CH and ArH), 8.22 (1H, d, *J* = 8.4 Hz, ArH). ^13^C-NMR (500 MHz, CDCl_3_): δ 49.8, 55.3, 62.4, 66.8, 101.3, 106.2, 106.9, 113.9, 115.0, 118.8, 119.9, 123.4, 124.0, 124.1, 124.6, 124.9, 126.3, 127.9, 129.1, 130.1, 131.7, 143.3, 151.5, 152.1, 155.0, 157.7, 159.6, 162.3, 163.5, 175.8, 178.0. LCMS (ES+) *m*/*z* = 614.2 ([M + H]^+^, *t*_R_ = 1.94 min). HRMS (ESI+) *m*/*z* = 614.1942 [M + H]^+^ found, C_36_H_28_N_3_O_7_^+^ required 614.1927.

*(E)-7-Methoxy-3-(4-((1-(2-(2-methoxy-4-(3-(2,4,6-trimethoxyphenyl)acryloyl)phenoxy)ethyl)-1H-1,2,3-triazol-4-yl)methoxy)phenyl)-4H-chromen-4-one* (**113**). A mixture of alkyne isoflavone **45** (236 mg, 0.770 mmol), azide chalcone **69** (308 mg, 0.744 mmol), CuSO_4_·5H_2_O (210 mg, 0.843 mmol) and sodium ascorbate (363 mg, 1.83 mmol) in *t*-BuOH/H_2_O (1:1, 40 mL) was reacted according to GP-A. The crude residue was purified by flash column chromatography (SiO_2_, 1%–5% MeOH/CH_2_Cl_2_) and recrystallized from MeOH/PE to afford triazole hybrid **113** (167 mg, 31%) as a pale yellow-orange powdery solid. m.p. 118–120 °C. TLC *R*_f_ = 0.33 (5% MeOH/CH_2_Cl_2_). IR ν_max_ (neat)/cm^−1^: 2942w (C-H str), 2840w (C-H str), 1637m (C=O str), 1595s (C=C str), 1559s (C=C str), 1511s (C=C str), 1438m, 1418m, 1336m, 1300m, 1244s, 1201m, 1153s, 1177s, 1118s, 1037s, 1024s. ^1^H-NMR (500 MHz, CDCl_3_): δ 3.86 (3H, s, -OCH_3_), 3.90 (6H, s, 2 × -OCH_3_), 3.90 (3H, s, -OCH_3_), 3.93 (3H, s, -OCH_3_), 4.46 (2H, t, *J* = 5.2 Hz, -OCH_2_C*H*_2_N-), 4.84 (2H, t, *J* = 5.2 Hz, -OC*H*_2_CH_2_N-), 5.26 (2H, s, -OC*H*_2_CN-), 6.13 (2H, s, ArH), 6.83 (1H, d, *J* = 0.4 Hz, ArH), 6.84 (1H, d, *J* = 6.0 Hz, ArH), 6.97 (1H, dd, *J* = 8.8, 2.4 Hz, ArH), 7.04 (2H, d, *J* = 8.8 Hz, ArH), 7.48 (2H, d, *J* = 8.8 Hz, ArH), 7.58 (1H, dd, *J* = 8.4, 2.0 Hz, ArH), 7.62 (1H, d, *J* = 2.0 Hz, ArH), 7.84 (1H, d, *J* = 15.6 Hz, -C*H*=CHCO-), 7.88 (1H, s, -C*H*N-), 8.02 (1H, s, -C=CH), 8.19 (1H, d, *J* = 8.8 Hz, ArH), 8.21 (1H, d, *J* = 15.6 Hz, -CH=C*H*CO-). ^13^C-NMR (500 MHz, CDCl_3_): δ 49.7, 55.0, 55.4, 55.8, 55.9, 62.1, 67.6, 90.5, 100.0, 106.6, 111.7, 112.5, 114.5, 114.8, 118.4, 121.6, 122.3, 122.9, 124.4, 127.7, 130.1, 133.7, 135.6, 144.3, 149.5, 150.7, 152.1, 157.9, 158.2, 158.3, 161.6, 163.0, 163.9, 175.7, 190.3. LCMS (ES+) *m*/*z* = 720.3 ([M + H]^+^, *t*_R_ = 1.69 min). HRMS (ESI+) *m*/*z* = 720.2529 [M + H]^+^ found, C_40_H_38_O_10_N_3_^+^ required 720.2552.

*(E)-7-((1-(2-(2-Methoxy-4-(3-(2,3,4-trimethoxyphenyl)acryloyl)phenoxy)ethyl)-1H-1,2,3-triazol-4-yl)-methoxy)-3-(4-methoxyphenyl)-4H-chromen-4-one* (**114**). A mixture of alkyne isoflavone **46** (226 mg, 0.738 mmol), azide chalcone **68** (301 mg, 0.728 mmol), CuSO_4_·5H_2_O (204 mg, 0.818 mmol) and sodium ascorbate (393 mg, 1.98 mmol) in *t*-BuOH/H_2_O (1:1, 40 mL) was reacted according to GP-A. The crude residue was purified by flash column chromatography (SiO_2_, 1%–5% MeOH/CH_2_Cl_2_) and recrystallized from MeOH to afford triazole hybrid **114** (331 mg, 63%) as a pale yellow-green powdery solid. m.p. 98–100 °C. TLC *R*_f_ = 0.39 (5% MeOH/CH_2_Cl_2_). IR ν_max_ (neat)/cm^−1^: 2937w (C-H str), 2838w (C-H str), 1623s (C=O str), 1594s (C=C str), 1576s (C=C str), 1512s (C=C str), 1494s, 1463m, 1442m, 1416m, 1247s, 1196m, 1178m, 1094s, 1027m. ^1^H-NMR (500 MHz, CDCl_3_): δ 3.83 (3H, s, -OCH_3_), 3.89 (3H, s, -OCH_3_), 3.91 (3H, s, -OCH_3_), 3.93 (3H, s, -OCH_3_), 3.95 (3H, s, -OCH_3_), 4.47 (2H, t, *J* = 4.8 Hz, -OCH_2_C*H*_2_N-), 4.86 (2H, t, *J* = 4.8 Hz, -OC*H*_2_CH_2_N-), 5.32 (2H, s, -OC*H*_2_CN-), 6.72 (1H, d, *J* = 8.4 Hz, ArH), 6.85 (1H, d, *J* = 8.4 Hz, ArH), 6.95 (2H, d, *J* = 8.8 Hz, ArH), 7.01 (1H, d, *J* = 2.0 Hz, ArH), 7.04 (1H, dd, *J* = 8.8, 2.0 Hz, ArH), 7.38 (1H, d, *J* = 8.8 Hz, ArH), 7.49 (2H, d, *J* = 8.8 Hz, ArH), 7.53 (1H, d, *J* = 15.6 Hz, -C*H*=CHCO-), 7.60 (1H, d, *J* = 8.4, 1.6 Hz, ArH), 7.62 (1H, d, *J* = 0.8 Hz, ArH), 7.90 (1H, s, -C=CH), 7.98 (1H, d, *J* = 16.0 Hz, -CH=C*H*CO-), 8.06 (1H, s, -C*H*N-), 8.20 (1H, d, *J* = 9.2 Hz, ArH). ^13^C-NMR (500 MHz, CDCl_3_): δ 49.8, 55.3, 56.0, 56.0, 60.9, 61.4, 62.4, 67.5, 101.2, 107.6, 111.5, 112.4, 113.9, 114.8, 118.7, 120.7, 122.0, 122.4, 123.9, 124.1, 124.7, 124.8, 127.9, 130.1, 133.0, 139.7, 142.4, 143.1, 149.6, 151.0, 152.1, 153.7, 155.7, 157.7, 159.5, 162.4, 175.7, 188.9. LCMS (ES+) *m*/*z* = 720.3 ([M + H]^+^, *t*_R_ = 1.84 min). HRMS (ESI+) *m*/*z* = 720.2533 [M + H]^+^ found, C_40_H_38_O_10_N_3_^+^ required 720.2552.

*3-Hydroxy-2-(4-(2-(4-(((3-(4-methoxyphenyl)-4-oxo-4H-chromen-7-yl)oxy)methyl)-1H-1,2,3-triazol-1-yl)-ethoxy)phenyl)-4H-chromen-4-one* (**115**). A mixture of alkyne isoflavone **46** (183 mg, 0.597 mmol), azide flavonol **79** (189 mg, 0.585 mmol), CuSO_4_·5H_2_O (271 mg, 1.09 mmol) and sodium ascorbate (493 mg, 2.49 mmol) in *t*-BuOH/H_2_O (1:1, 40 mL) was reacted according to GP-A. The crude residue was purified by flash column chromatography (SiO_2_, 1%–5% MeOH/CH_2_Cl_2_) and recrystallized from MeOH to afford triazole hybrid **115** (42.9 mg, 12%) as a yellow-green powdery solid. m.p. 252–254 °C. TLC *R*_f_ = 0.30 (3% MeOH/CH_2_Cl_2_). IR ν_max_ (neat)/cm^−1^: 3371w(br) (O-H str), 3064w (C-H str), 2928w (C-H str), 1640m, 1626m (C=O str), 1601s, 1563m (C=C str), 1512m (C=C str), 1484w, 1444m, 1409m, 1290w, 1247s, 1201m, 1183m, 1121m, 1049m, 1027m. ^1^H-NMR (500 MHz, CDCl_3_): δ 3.83 (3H, s, -OCH_3_), 4.48 (2H, t, *J* = 5.0 Hz, -OCH_2_C*H*_2_N-), 4.86 (2H, t, *J* = 5.0 Hz, -OC*H*_2_CH_2_N-), 5.36 (2H, s, -OC*H*_2_CN-), 6.92 (2H, d, *J* = 9.0 Hz, ArH), 6.97 (3H, d, *J* = 9.0 Hz, ArH and OH), 7.00 (1H, d, *J* = 1.5 Hz, ArH), 7.07 (1H, dd, *J* = 8.5, 2.0 Hz, ArH), 7.42 (1H, t, *J* = 7.5 Hz, ArH), 7.47 (2H, d, *J* = 8.5 Hz, ArH), 7.57 (1H, d, *J* = 8.0 Hz, ArH), 7.70 (1H, t, *J* = 8.5 Hz, ArH), 7.89 (2H, s, -C=CH and -C*H*N-), 8.21–8.25 (4H, m, ArH). ^13^C-NMR (500 MHz, CDCl_3_): δ 49.8, 55.3, 62.4, 66.3, 101.3, 113.9, 114.5, 115.0, 118.2, 118.8, 120.6, 124.1, 124.5, 124.7, 124.9, 125.4, 127.9, 129.6, 130.0, 133.5, 137.8, 144.6, 152.1, 155.3, 157.7, 159.0, 159.5, 162.3, 173.2, 175.8. LCMS (ES+) *m*/*z* = 630.2 ([M + H]^+^, *t*_R_ = 1.77 min). HRMS (ESI+) *m*/*z* = 630.1861 [M + H]^+^ found, C_36_H_28_O_8_N_3_^+^ required 630.1871.

*2-((Z)-4-(2-(4-((2-Methoxy-4-((E)-3-(4-methoxyphenyl)acryloyl)phenoxy)methyl)-1H-1,2,3-triazol-1-yl)-ethoxy)benzylidene)benzofuran-3(2H)-one* (**116**). A mixture of alkyne chalcone **20** (192 mg, 0.595 mmol), azide aurone **88** (187 mg, 0.609 mmol), CuSO_4_·5H_2_O (289 mg, 1.16 mmol) and sodium ascorbate (548 mg, 2.77 mmol) in *t*-BuOH/H_2_O (1:1, 40 mL) was reacted according to GP-A. The crude residue was purified by flash column chromatography (SiO_2_, 1%–5% MeOH/CH_2_Cl_2_) and recrystallized from MeOH to afford triazole hybrid **116** (148 mg, 40%) as a bright yellow powdery solid. m.p. 128–130 °C. TLC *R*_f_ = 0.33 (3% MeOH/CH_2_Cl_2_). IR ν_max_ (neat)/cm^−1^: 2941w (C-H str), 2835w (C-H str), 1695m (C=O str), 1647m, 1590s (C=C str), 1571s (C=C str), 1509s (C=C str), 1461m, 1422m, 1296m, 1250s, 1174s, 1147s, 1128s, 1110s, 1098m, 1025s. ^1^H-NMR (500 MHz, CDCl_3_): δ 3.85 (3H, s, -OCH_3_), 3.97 (3H, s, -OCH_3_), 4.43 (2H, t, *J* = 5.0 Hz, -OCH_2_C*H*_2_N-), 4.80 (2H, t, *J* = 5.0 Hz, -OC*H*_2_CH_2_N-), 5.42 (2H, s, -OC*H*_2_CN-), 6.83 (1H, s, -C=CH), 6.89 (2H, d, *J* = 9.0 Hz, ArH), 6.91 (2H, d, *J* = 9.0 Hz, ArH), 7.12 (1H, d, *J* = 8.0 Hz, ArH), 7.22 (1H, t, *J* = 8.0 Hz, ArH), 7.33 (1H, d, *J* = 8.0 Hz, ArH), 7.39 (1H, d, *J* = 15.5 Hz, -C*H*=CHCO-), 7.56 (2H, d, *J* = 8.5 Hz, ArH), 7.60 (1H, dd, *J* = 8.5, 2.0 Hz, ArH), 7.64–7.67 (2H, m, ArH), 7.77 (1H, d, *J* = 16.0 Hz, -CH=C*H*CO-), 7.79–7.81 (1H, m, ArH), 7.86 (2H, d, *J* = 9.0 Hz, ArH), 7.87 (1H, br s, -C*H*N-). ^13^C-NMR (500 MHz, CDCl_3_): δ 49.7, 55.4, 56.1, 62.9, 66.3, 111.1, 112.3, 112.6, 112.9, 114.3, 114.9, 119.1, 121.8, 122.6, 123.3, 124.3, 124.6, 126.1, 127.7, 130.1, 132.2, 133.1, 133.4, 136.6, 144.0, 146.1, 149.6, 151.4, 158.9, 161.5, 165.9, 184.5, 188.6. LCMS (ES+) *m*/*z* = 630.0 ([M + H]^+^, *t*_R_ = 1.72 min). HRMS (ESI+) *m*/*z* = 630.2217 [M + H]^+^ found, C_37_H_32_O_7_N_3_^+^ required 630.2235.

*2-((Z)-4-(2-(4-((2-((E)-3-(Ferrocenyl)acryloyl)phenoxy)methyl)-1H-1,2,3-triazol-1-yl)ethoxy)benzylidene)benzofuran-3(2H)-one* (**117**). A mixture of alkyne chalcone **26** (286 mg, 0.772 mmol), azide aurone **88** (248 mg, 0.806 mmol), CuSO_4_·5H_2_O (236 mg, 0.943 mmol) and sodium ascorbate (426 mg, 2.15 mmol) in *t*-BuOH/H_2_O (1:1, 40 mL) was reacted according to GP-A. The crude residue was purified by flash column chromatography (SiO_2_, 1%–5% MeOH/CH_2_Cl_2_) to afford triazole hybrid **117** (409 mg, 78%) as a dark red-purple microcrystalline solid. m.p. 108–110 °C. TLC *R*_f_ = 0.48 (3% MeOH/CH_2_Cl_2_). IR ν_max_ (neat)/cm^−1^: 3090w (C-H str), 1699m (C=O str), 1646m, 1594s (C=C str), 1569m (C=C str), 1509s (C=C str), 1476w, 1459m, 1347w, 1297m, 1247m, 1208m, 1178s, 1127s, 1109s, 1097s, 1044m, 1025m, 1001w. ^1^H-NMR (500 MHz, CDCl_3_): δ 4.11 (5H, s, -C_5_H_5_), 4.32 (2H, t, *J* = 5.2 Hz, -OCH_2_C*H*_2_N-), 4.42 (2H, t, *J* = 2.0 Hz, -C_5_H_4_), 4.47 (2H, t, *J* = 2.0 Hz, -C_5_H_4_), 4.65 (2H, t, *J* = 5.2 Hz, -OC*H*_2_CH_2_N-), 5.32 (2H, s, -OC*H*_2_CN-), 6.85 (1H, s, -C=CH), 6.88 (2H, d, *J* = 8.8 Hz, ArH), 6.95 (1H, d, *J* = 15.6 Hz, -C*H*=CHCO-), 7.08 (1H, t, *J* = 7.6 Hz, ArH), 7.13 (1H, d, *J* = 8.4 Hz, ArH), 7.22 (1H, t, *J* = 7.6 Hz, ArH), 7.34 (1H, d, *J* = 8.4 Hz, ArH), 7.44–7.46 (1H, m, ArH), 7.49 (1H, d, *J* = 15.6 Hz, -CH=C*H*CO-), 7.60 (1H, dd, *J* = 7.6, 1.6 Hz, ArH), 7.65 (1H, t, *J* = 8.4 Hz, ArH), 7.74 (1H, s, -C*H*N-), 7.81 (1H, dd, *J* = 7.6, 0.8 Hz, ArH), 7.86 (2H, d, *J* = 8.8 Hz, ArH). ^13^C-NMR (500 MHz, CDCl_3_): δ 49.5, 62.9, 66.1, 68.9, 69.8, 71.2, 79.0, 112.8, 112.9, 113.1, 115.0, 121.4, 121.8, 123.3, 123.9, 124.6, 124.7, 125.9, 130.1, 130.2, 132.4, 133.4, 136.6, 144.1, 145.4, 146.0, 156.3, 159.0, 165.8, 184.5, 192.3. LCMS (ES+) *m*/*z* = 678.2 ([M + H]^+^, *t*_R_ = 2.11 min). HRMS (ESI+) *m*/*z* = 678.1677 [M + H]^+^ found, C_39_H_32_N_3_O_5_Fe^+^ required 678.1691.

*6-Methoxy-2-((Z)-4-(2-(4-((2-methoxy-5-((E)-3-(1-methyl-1H-pyrrol-2-yl)acryloyl)phenoxy)methyl)-1H-1,2,3-triazol-1-yl)ethoxy)benzylidene)benzofuran-3(2H)-one* (**118**). A mixture of alkyne chalcone **22** (265 mg, 0.898 mmol), azide aurone **89** (304 mg, 0.900 mmol), CuSO_4_·5H_2_O (264 mg, 1.06 mmol) and sodium ascorbate (474 mg, 2.39 mmol) in *t*-BuOH/H_2_O (1:1, 40 mL) was reacted according to GP-A. The crude residue was purified by flash column chromatography (SiO_2_, 1%–5% MeOH/CH_2_Cl_2_) to afford triazole hybrid **118** (270 mg, 48%) as a bright yellow powdery solid. m.p. 138–140 °C. TLC *R*_f_ = 0.46 (5% MeOH/CH_2_Cl_2_). IR ν_max_ (neat)/cm^−1^: 2934w (C-H str), 2884w (C-H str), 1700m (C=O str), 1656m, 1638m, 1596s (C=C str), 1562s (C=C str), 1510m (C=C str), 1483m, 1412m, 1330m, 1267s, 1198m, 1152s, 1132s, 1099s, 1056m, 1042s, 1022s. ^1^H-NMR (500 MHz, CDCl_3_): δ 3.74 (3H, s, -NCH_3_), 3.94 (3H, s, -OCH_3_), 3.96 (3H, s, -OCH_3_), 4.42 (2H, t, *J* = 4.8 Hz, -OCH_2_C*H*_2_N-), 4.79 (2H, t, *J* = 4.8 Hz, -OC*H*_2_CH_2_N-), 5.41 (2H, s, -OC*H*_2_CN-), 6.19–6.21 (1H, m, ArH), 6.74–6.80 (4H, m, ArH), 6.78 (1H, s, -C=CH), 6.87 (2H, d, *J* = 8.8 Hz, ArH), 7.11 (1H, d, *J* = 8.4 Hz, ArH), 7.27 (1H, d, *J* = 15.2 Hz, -C*H*=CHCO-, overlain by CDCl_3_), 7.58 (1H, dd, *J* = 8.4, 1.6 Hz, ArH), 7.64 (1H, d, *J* = 2.0 Hz, ArH), 7.69 (1H, d, *J* = 8.8 Hz, ArH), 7.78 (1H, d, *J* = 15.2 Hz, -CH=C*H*CO-), 7.82 (2H, d, *J* = 8.8 Hz, ArH), 7.87 (1H, s, -C*H*N-). ^13^C-NMR (500 MHz, CDCl_3_): δ 34.3, 49.7, 56.0, 56.0, 62.8, 66.2, 96.6, 109.6, 111.0, 111.4, 112.0, 112.1, 112.3, 114.8, 114.9, 116.2, 122.3, 124.3, 125.6, 126.1, 127.6, 130.2, 131.6, 132.4, 133.1, 143.8, 147.0, 149.4, 151.2, 158.6, 167.2, 168.2, 182.8, 188.0. LCMS (ES+) *m*/*z* = 633.3 ([M + H]^+^, *t*_R_ = 1.94 min). HRMS (ESI+) *m*/*z* = 633.2352 [M + H]^+^ found, C_36_H_33_N_4_O_7_^+^ required 633.2349.

*2-((Z)-4-(2-(4-((4-Bromo-2-((E)-3-(1-methyl-1H-indol-3-yl)acryloyl)phenoxy)methyl)-1H-1,2,3-triazol-1-yl)ethoxy)benzylidene)benzofuran-3(2H)-one* (**119**). A mixture of alkyne chalcone **21** (308 mg, 0.781 mmol), azide aurone **88** (239 mg, 0.779 mmol), CuSO_4_·5H_2_O (261 mg, 1.04 mmol) and sodium ascorbate (397 mg, 2.00 mmol) in *t*-BuOH/H_2_O (1:1, 40 mL) was reacted according to GP-A. The crude residue was purified by flash column chromatography (SiO_2_, 1%–5% MeOH/CH_2_Cl_2_) and recrystallized from MeOH to afford triazole hybrid **119** (117 mg, 21%) as a pale yellow-brown powdery solid. m.p. 138–140 °C. TLC *R*_f_ = 0.44 (5% MeOH/CH_2_Cl_2_). IR ν_max_ (neat)/cm^−1^: 2930w (C-H str), 1699m (C=O str), 1644m, 1590s (C=C str), 1525m (C=C str), 1508m (C=C str), 1460m, 1395w, 1373m, 1249m, 1177s, 1126m, 1096w, 1045w. ^1^H-NMR (500 MHz, CDCl_3_): δ 3.74 (3H, s, -NCH_3_), 4.03 (2H, t, *J* = 5.2 Hz, -OCH_2_C*H*_2_N-), 4.37 (2H, t, *J* = 5.2 Hz, -OC*H*_2_CH_2_N-), 5.32 (2H, s, -OC*H*_2_CN-), 6.71 (2H, d, *J* = 8.8 Hz, ArH), 6.82 (1H, s, -C=CH), 7.04 (1H, d, *J* = 8.8 Hz, ArH), 7.16–7.21 (1H, m, ArH), 7.23 (1H, t, *J* = 7.6 Hz, ArH), 7.29–7.30 (2H, m, ArH), 7.33–7.35 (2H, m, ArH), 7.38 (1H, d, *J* = 16.0 Hz, -C*H*=CHCO-), 7.54 (1H, dd, *J* = 8.8, 2.4 Hz, ArH), 7.64–7.69 (2H, m, ArH), 7.73 (1H, s, -C*H*N-), 7.77 (2H, d, *J* = 8.8 ArH), 7.80–7.82 (2H, m, ArH), 7.85 (1H, d, *J* = 15.6 Hz, -CH=C*H*CO-). ^13^C-NMR (500 MHz, CDCl_3_): δ 33.2, 49.2, 63.3, 65.7, 110.0, 112.7, 112.7, 112.9, 114.0, 114.7, 114.9, 120.6, 121.7, 121.7, 121.8, 123.1, 123.4, 124.1, 124.6, 125.7, 125.8, 132.0, 133.1, 133.2, 134.8, 135.0, 136.7, 138.1, 138.1, 143.7, 146.0, 155.5, 158.8, 165.8, 184.5, 190.8. LCMS (ES+) *m*/*z* = 703.1 ([M + H]^+^, *t*_R_ = 5.05 min). HRMS (ESI+) *m*/*z* = 723.1189 [M + Na]^+^ found, C_38_H_29_O_5_N_4_BrNa^+^ required 723.1214.

*(Z)-3-((1-(2-(4-((6-Methoxy-3-oxobenzofuran-2(3H)-ylidene)methyl)phenoxy)ethyl)-1H-1,2,3-triazol-4-yl)-methoxy)-2-phenyl-4H-chromen-4-one* (**120**). A mixture of alkyne flavone **30** (250 mg, 0.906 mmol), azide aurone **89** (316 mg, 0.936 mmol), CuSO_4_·5H_2_O (284 mg, 1.14 mmol) and sodium ascorbate (447 mg, 2.26 mmol) in *t*-BuOH/H_2_O (1:1, 40 mL) was reacted according to GP-A. The crude residue was purified by flash column chromatography (SiO_2_, 1%–5% MeOH/CH_2_Cl_2_) and recrystallized from MeOH to afford triazole hybrid **120** (351 mg, 63%) as a pale yellow-white powdery solid. m.p. 186–188 °C. TLC *R*_f_ = 0.38 (5% MeOH/CH_2_Cl_2_). IR ν_max_ (neat)/cm^−1^: 3056w (C-H str), 1698m (C=O str), 1654w, 1608s, 1595s (C=C str), 1509m (C=C str), 1470m, 1434m, 1397m, 1342m, 1272s, 1245s, 1198s, 1176m, 1147m, 1128s, 1109s, 1091s, 1042m. ^1^H-NMR (500 MHz, CDCl_3_): δ 3.93 (3H, s, -OCH_3_), 4.35 (2H, t, *J* = 5.0 Hz, -OCH_2_C*H*_2_N-), 4.71 (2H, t, *J* = 4.5 Hz, -OC*H*_2_CH_2_N-), 5.33 (2H, s, -OC*H*_2_CN-), 6.74–6.77 (3H, m, ArH and -C=CH), 6.90 (2H, d, *J* = 9.0 Hz, ArH), 7.40–7.45 (4H, m, ArH), 7.52 (1H, dd, *J* = 8.5, 0.5 Hz, ArH), 7.69 (1H, t, *J* = 8.5 Hz, ArH), 7.70 (1H, d, *J* = 8.5 Hz, ArH), 7.83 (2H, d, *J* = 9.0 Hz, ArH), 7.83 (1H, s, -C*H*N-), 8.01–8.03 (2H, m, ArH), 8.28 (1H, dd, *J* = 8.0, 1.5 Hz, ArH). ^13^C-NMR (500 MHz, CDCl_3_): δ 49.5, 56.0, 65.1, 66.2, 96.6, 111.5, 112.0, 114.9, 115.0, 118.1, 124.1, 124.8, 124.9, 125.7, 126.1, 128.3, 128.7, 130.7, 133.1, 133.5, 139.5, 144.0, 147.0, 155.3, 156.4, 158.8, 167.3, 168.3, 175.1, 182.8. LCMS (ES+) *m*/*z* = 614.2 ([M + H]^+^, *t*_R_ = 1.91 min). HRMS (ESI+) *m*/*z* = 614.1926 [M + H]^+^ found, C_36_H_28_N_3_O_7_^+^ required 614.1927.

*(Z)-6-((1-(2-(4-((6-Methoxy-3-oxobenzofuran-2(3H)-ylidene)methyl)phenoxy)ethyl)-1H-1,2,3-triazol-4-yl)methoxy)-2-phenyl-4H-chromen-4-one* (**121**). A mixture of alkyne flavone **31** (249 mg, 0.900 mmol), azide aurone **89** (309 mg, 0.916 mmol), CuSO_4_·5H_2_O (247 mg, 0.989 mmol) and sodium ascorbate (482 mg, 2.43 mmol) in *t*-BuOH/H_2_O (1:1, 40 mL) was reacted according to GP-A. The crude residue was purified by flash column chromatography (SiO_2_, 1%–5% MeOH/CH_2_Cl_2_) and recrystallized from MeOH to afford triazole hybrid **121** (269 mg, 49%) as a bright yellow-orange powdery solid. m.p. 148–150 °C. TLC *R*_f_ = 0.30 (5% MeOH/CH_2_Cl_2_). IR ν_max_ (neat)/cm^−1^: 3065w (C-H str), 2925w (C-H str), 1695m (C=O str), 1640s, 1594s (C=C str), 1568s (C=C str), 1510m (C=C str), 1496m, 1481m, 1454s, 1442s, 1360m, 1270m, 1250s, 1181s, 1131s, 1110s, 1095s, 1043m, 1027m. ^1^H-NMR (500 MHz, CDCl_3_): δ 3.92 (3H, s, -OCH_3_), 4.43 (2H, t, *J* = 4.8 Hz, -OCH_2_C*H*_2_N-), 4.82 (2H, t, *J* = 4.8 Hz, -OC*H*_2_CH_2_N-), 5.30 (2H, s, -OC*H*_2_CN-), 6.72–6.74 (2H, m, ArH), 6.75 (1H, s, -C=CH), 6.79 (1H, s, -C=CH), 6.89 (2H, d, *J* = 8.8 Hz, ArH), 7.33 (1H, dd, *J* = 9.2, 3.2 Hz, ArH), 7.48–7.52 (4H, m, ArH), 7.68 (1H, d, *J* = 9.2 Hz, ArH), 7.71 (1H, d, *J* = 3.2 Hz, ArH), 7.80 (2H, d, *J* = 8.8 Hz, ArH), 7.88–7.90 (3H, m, ArH and -C*H*N-). ^13^C-NMR (500 MHz, CDCl_3_): δ 49.7, 56.0, 62.2, 66.3, 96.5, 106.4, 106.8, 111.4, 112.0, 114.8, 114.9, 120.0, 123.9, 124.2, 124.5, 125.7, 126.1, 126.2, 129.0, 131.5, 131.7, 133.1, 143.4, 146.9, 151.2, 155.4, 158.7, 163.2, 167.2, 168.2, 178.1, 182.8. LCMS (ES+) *m*/*z* = 614.2 ([M + H]^+^, *t*_R_ = 1.77 min). HRMS (ESI+) *m*/*z* = 614.1906 [M + H]^+^ found, C_36_H_28_O_7_N_3_^+^ required 614.1922.

*(Z)-3-Hydroxy-2-(4-((1-(2-(4-((3-oxobenzofuran-2(3H)-ylidene)methyl)phenoxy)ethyl)-1H-1,2,3-triazol-4-yl)methoxy)phenyl)-4H-chromen-4-one* (**122**). A mixture of alkyne flavonol **36** (391 mg, 1.34 mmol), azide aurone **88** (408 mg, 1.33 mmol), CuSO_4_·5H_2_O (435 mg, 1.74 mmol) and sodium ascorbate (682 mg, 3.44 mmol) in *t*-BuOH/H_2_O (1:1, 40 mL) was reacted according to GP-A. The crude residue was purified by flash column chromatography (SiO_2_, 1%–5% MeOH/CH_2_Cl_2_) and recrystallized from MeOH to afford triazole hybrid **122** (335 mg, 42%) as a yellow-brown powdery solid. m.p. 208–210 °C. TLC *R*_f_ = 0.32 (5% MeOH/CH_2_Cl_2_). IR ν_max_ (neat)/cm^−1^: 3300w (O-H str), 3073w (C-H str), 2951w (C-H str), 1694m (C=O str), 1646w, 1604s, 1596s (C=C str), 1567w (C=C str), 1509s (C=C str), 1458w, 1426w, 1407w, 1299m, 1256s, 1178s, 1109s, 1051s. ^1^H-NMR (500 MHz, CDCl_3_): δ 4.46 (2H, t, *J* = 4.8 Hz, -OCH_2_C*H*_2_N-), 4.84 (2H, t, *J* = 4.8 Hz, -OC*H*_2_CH_2_N-), 5.34 (2H, s, -OC*H*_2_CN-), 6.82 (1H, s, -C=CH), 6.92 (2H, d, *J* = 8.8 Hz, ArH), 6.96 (1H, br s, OH), 7.15 (2H, d, *J* = 8.8 Hz, ArH), 7.22 (1H, t, *J* = 7.6 Hz, ArH), 7.31 (1H, d, *J* = 8.4 Hz, ArH), 7.39 (1H, t, *J* = 7.6 Hz, ArH), 7.56 (1H, d, *J* = 8.8 Hz, ArH), 7.63–7.71 (2H, m, ArH), 7.80 (1H, d, *J* = 7.6 Hz, ArH), 7.86 (3H, d, *J* = 8.4 Hz, ArH and -C*H*N-), 8.21–8.25 (3H, m, ArH). ^13^C-NMR (500 MHz, CDCl_3_): δ 49.8, 62.1, 66.4, 112.6, 112.9, 114.9, 114.9, 118.1, 120.6, 121.8, 123.4, 124.1, 124.4, 124.6, 125.4, 126.1, 129.5, 133.0, 133.4, 136.5, 136.7, 137.7, 145.0, 146.1, 155.3, 157.4, 158.9, 159.5, 165.9, 173.1, 184.5. LCMS (ES+) *m*/*z* = 600.0 ([M + H]^+^, *t*_R_ = 4.68 min). HRMS (ESI+) *m*/*z* = 600.1751 [M + H]^+^ found, C_35_H_26_O_7_N_3_^+^ required 600.1765.

*(Z)-2-(4-(2-(4-((3-((4,6-Dimethoxy-3-oxobenzofuran-2(3H)-ylidene)methyl)phenoxy)methyl)-1H-1,2,3-triazol-1-yl)ethoxy)phenyl)-3-hydroxy-7-methoxy-4H-chromen-4-one* (**123**). A mixture of alkyne aurone **56** (192 mg, 0.571 mmol), azide flavonol **80** (210 mg, 0.595 mmol), CuSO_4_·5H_2_O (234 mg, 0.939 mmol) and sodium ascorbate (362 mg, 1.83 mmol) in *t*-BuOH/H_2_O (1:1, 40 mL) was reacted according to GP-A. The crude residue was purified by flash column chromatography (SiO_2_, 1%–5% MeOH/CH_2_Cl_2_) and recrystallized from MeOH to afford triazole hybrid **123** (54.3 mg, 14%) as a pale yellow-brown powdery solid. m.p. 178–180 °C. TLC *R*_f_ = 0.29 (5% MeOH/CH_2_Cl_2_). IR ν_max_ (neat)/cm^−1^: 3302w (O-H str), 2939w (C-H str), 2845w (C-H str), 1692w, 1614s (C=O str), 1599s (C=C str), 1510m (C=C str), 1503m (C=C str), 1452m, 1403w, 1361w, 1346w, 1251m, 1215s, 1187w, 1156m, 1121w, 1092s, 1036w. ^1^H-NMR (500 MHz, CDCl_3_): δ 3.91 (3H, s, -OCH_3_), 3.92 (3H, s, -OCH_3_), 3.95 (3H, s, -OCH_3_), 4.46 (2H, t, *J* = 4.5 Hz, -OCH_2_C*H*_2_N-), 4.83 (2H, t, *J* = 4.5 Hz, -OC*H*_2_CH_2_N-), 5.34 (2H, s, -OC*H*_2_CN-), 6.08 (1H, s, ArH), 6.41 (1H, s, ArH), 6.70 (1H, s, -C=CH), 6.93–6.95 (4H, m, ArH and OH), 6.99–7.03 (2H, m, ArH), 7.34 (1H, t, *J* = 8.0 Hz, ArH), 7.40 (1H, d, *J* = 7.5 Hz, ArH), 7.55 (1H, s, ArH), 7.85 (1H, br s, -C*H*N-), 8.12 (1H, d, *J* = 9.0 Hz, ArH), 8.16 (2H, d, *J* = 8.5 Hz, ArH). ^13^C-NMR (500 MHz, CDCl_3_): δ 49.8, 55.9, 56.2, 56.2, 62.2, 66.4, 89.3, 94.1, 99.9, 105.1, 110.3, 114.4, 114.6, 114.8, 116.2, 116.7, 121.9, 123.9, 124.5, 124.8, 126.7, 129.3, 129.8, 134.0, 137.4, 144.0, 148.0, 157.2, 158.3, 158.8, 159.3, 164.2, 169.1, 172.6, 180.6. LCMS (ES+) *m*/*z* = 690.2 ([M + H]^+^, *t*_R_ = 4.59 min). HRMS (ESI+) *m*/*z* = 690.2062 [M + H]^+^ found, C_38_H_32_O_10_N_3_^+^ required 690.2082.

*(Z)-7-((1-(2-(4-((6-Methoxy-3-oxobenzofuran-2(3H)-ylidene)methyl)phenoxy)ethyl)-1H-1,2,3-triazol-4-yl)-methoxy)-3-(4-methoxyphenyl)-4H-chromen-4-one* (**124**). A mixture of alkyne isoflavone **46** (276 mg, 0.901 mmol), azide aurone **89** (301 mg, 0.892 mmol), CuSO_4_·5H_2_O (348 mg, 1.39 mmol) and sodium ascorbate (496 mg, 2.50 mmol) in *t*-BuOH/H_2_O (1:1, 40 mL) was reacted according to GP-A. The crude residue was purified by flash column chromatography (SiO_2_, 1%–5% MeOH/CH_2_Cl_2_) and recrystallized from MeOH to afford triazole hybrid **124** (317 mg, 55%) as a bright yellow powdery solid. m.p. 228–230 °C. TLC *R*_f_ = 0.43 (5% MeOH/CH_2_Cl_2_). IR ν_max_ (neat)/cm^−1^: 3085w (C-H str), 2935w (C-H str), 1705m (C=O str), 1651m, 1629s, 1596s (C=C str), 1567m (C=C str), 1510s (C=C str), 1441s, 1347m, 1295m, 1249s, 1202m, 1179s, 1147s, 1098s, 1020s. ^1^H-NMR (500 MHz, CDCl_3_): δ 3.83 (3H, s, -OCH_3_), 3.93 (3H, s, -OCH_3_), 4.44 (2H, t, *J* = 4.8 Hz, -OCH_2_C*H*_2_N-), 4.83 (2H, t, *J* = 4.8 Hz, -OC*H*_2_CH_2_N-), 5.34 (2H, s, -OC*H*_2_CN-), 6.74–6.77 (2H, m, ArH), 6.76 (1H, s, -C=CH), 6.88 (2H, d, *J* = 8.8 Hz, ArH), 6.93 (2H, d, *J* = 8.8 Hz, ArH), 6.97 (1H, d, *J* = 2.4 Hz, ArH), 7.06 (1H, dd, *J* = 8.8, 2.4 Hz, ArH), 7.47 (2H, d, *J* = 8.8 Hz, ArH), 7.69 (1H, d, *J* = 8.4 Hz, ArH), 7.82 (2H, d, *J* = 8.8 Hz, ArH), 7.87 (1H, s, -C*H*N-), 7.89 (1H, s, -C=CH), 8.23 (1H, d, *J* = 8.8 Hz, ArH). ^13^C-NMR (500 MHz, CDCl_3_): δ 49.8, 55.3, 56.0, 62.4, 66.3, 96.7, 101.3, 111.3, 112.1, 113.9, 114.8, 114.9, 118.8, 124.1, 124.2, 124.9, 125.7, 126.3, 127.9, 130.0, 133.1, 143.2, 147.0, 152.1, 157.7, 158.6, 159.5, 162.3, 167.3, 168.3, 175.8, 182.8. LCMS (ES+) *m*/*z* = 644.2 ([M + H]^+^, *t*_R_ = 1.86 min). HRMS (ESI+) *m*/*z* = 644.2011 [M + H]^+^ found, C_37_H_30_O_8_N_3_^+^ required 644.2027.

*4,6-Dimethoxy-2-((Z)-3-((1-(2-(4-(((Z)-3-oxobenzofuran-2(3H)-ylidene)methyl)phenoxy)ethyl)-1H-1,2,3-triazol-4-yl)methoxy)benzylidene)benzofuran-3(2H)-one* (**125**). A mixture of alkyne aurone **56** (303 mg, 0.902 mmol), azide aurone **88** (278 mg, 0.905 mmol), CuSO_4_·5H_2_O (263 mg, 1.05 mmol) and sodium ascorbate (462 mg, 2.33 mmol) in *t*-BuOH/H_2_O (1:1, 20 mL) was reacted according to GP-A. The crude residue was purified by flash column chromatography (SiO_2_, 1%–5% MeOH/CH_2_Cl_2_) and recrystallized from MeOH to afford triazole hybrid **125** (491 mg, 85%) as a bright yellow-brown powdery solid. m.p. 148–150 °C. TLC *R*_f_ = 0.36 (5% MeOH/CH_2_Cl_2_). IR ν_max_ (neat)/cm^−1^: 2942w (C-H str), 2843w (C-H str), 1694m (C=O str), 1651m, 1587s (C=C str), 1508m (C=C str), 1458m, 1423w, 1345m, 1298m, 1251m, 1234m, 1214s, 1177s, 1154s, 1128s, 1091s, 1036m. ^1^H-NMR (500 MHz, CDCl_3_): δ 3.90 (3H, s, -OCH_3_), 3.92 (3H, s, -OCH_3_), 4.42 (2H, t, *J* = 4.8 Hz, -OCH_2_C*H*_2_N-), 4.81 (2H, t, *J* = 4.8 Hz, -OC*H*_2_CH_2_N-), 5.31 (2H, s, -OC*H*_2_CN-), 6.09 (1H, d, *J* = 1.6 Hz, ArH), 6.40 (1H, d, *J* = 1.2 Hz, ArH), 6.68 (1H, s, -C=CH), 6.81 (1H, s, -C=CH), 6.88 (2H, d, *J* = 8.4 Hz, ArH), 7.00 (1H, dd, *J* = 8.0, 2.4 Hz, ArH), 7.21 (1H, t, *J* = 7.6 Hz, ArH), 7.30–7.34 (2H, m, ArH), 7.39 (1H, d, *J* = 8.0 Hz, ArH), 7.54 (1H, s, ArH), 7.64 (1H, t, *J* = 7.6 Hz, ArH), 7.79 (1H, d, *J* = 7.6 Hz, ArH), 7.82–7.85 (3H, m, ArH and -C*H*N-). ^13^C-NMR (500 MHz, CDCl_3_): δ 49.7, 56.2, 62.2, 66.3, 89.3, 94.1, 105.1, 110.3, 112.7, 112.9, 114.3, 114.8, 116.3, 116.6, 121.8, 123.3, 123.9, 124.5, 124.5, 126.0, 129.8, 133.0, 133.4, 133.9, 136.7, 144.3, 146.1, 148.0, 158.3, 158.9, 159.3, 165.9, 169.0, 180.6, 184.5. LCMS (ES+) *m*/*z* = 644.2 ([M + H]^+^, *t*_R_ = 2.02 min). HRMS (ESI+) *m*/*z* = 644.2006 [M + H]^+^ found, C_37_H_30_O_8_N_3_^+^ required 644.2027.

*(E)-4-((1-(2-(4-(3-(2-Hydroxyphenyl)-3-oxoprop-1-en-1-yl)phenoxy)ethyl)-1H-1,2,3-triazol-4-yl)methoxy)-2H-chromen-2-one* (**126**). A mixture of alkyne coumarin **48** (207 mg, 1.03 mmol), azide chalcone **61** (302 mg, 0.975 mmol), CuSO_4_·5H_2_O (292 mg, 1.17 mmol) and sodium ascorbate (508 mg, 2.56 mmol) in *t*-BuOH/H_2_O (1:1, 40 mL) was reacted according to GP-A. The crude residue was purified by flash column chromatography (SiO_2_, 1%–5% MeOH/CH_2_Cl_2_) and recrystallized from MeOH to afford triazole hybrid **126** (387 mg, 78%) as a bright yellow powdery solid. m.p. 194–196 °C. TLC *R*_f_ = 0.43 (5% MeOH/CH_2_Cl_2_). IR ν_max_ (neat)/cm^−1^: 3083w (C-H str), 2929w (C-H str), 1725s (C=O str), 1641m, 1625m, 1607m, 1560s (C=C str), 1511s (C=C str), 1489s, 1424m, 1382m, 1273m, 1249s, 1202s, 1175s, 1156s, 1107m, 1056m, 1030m. ^1^H-NMR (500 MHz, CDCl_3_): δ 4.47 (2H, t, *J* = 5.2 Hz, -OCH_2_C*H*_2_N-), 4.87 (2H, t, *J* = 5.2 Hz, -OC*H*_2_CH_2_N-), 5.36 (2H, s, -OC*H*_2_CN-), 5.87 (1H, s, -C=CH), 6.91–6.97 (3H, m, ArH), 7.03 (1H, d, *J* = 8.4 Hz, ArH), 7.23 (1H, t, *J* = 8.0 Hz, ArH), 7.31 (1H, d, *J* = 8.4 Hz, ArH), 7.48–7.54 (2H, m, ArH), 7.54 (1H, d, *J* = 15.2 Hz, -C*H*=CHCO-), 7.61 (2H, d, *J* = 8.4 Hz, ArH), 7.78 (1H, dd, *J* = 8.0, 1.6 Hz, ArH), 7.86 (1H, d, *J* = 15.6 Hz, -CH=C*H*CO-), 7.91 (1H, dd, *J* = 8.0, 1.2 Hz, ArH), 7.94 (1H, s, -C*H*N-), 12.87 (1H, s, OH). ^13^C-NMR (500 MHz, CDCl_3_): δ 49.9, 62.5, 66.3, 91.2, 115.0, 115.4, 116.8, 118.4, 118.6, 118.8, 120.0, 123.1, 123.9, 124.7, 128.5, 129.5, 130.6, 132.6, 136.3, 141.6, 144.7, 153.3, 159.8, 162.6, 163.5, 164.9, 193.6. LCMS (ES+) *m*/*z* = 510.2 ([M + H]^+^, *t*_R_ = 1.65 min). HRMS (ESI+) *m*/*z* = 510.1651 [M + H]^+^ found, C_29_H_24_O_6_N_3_^+^ required 510.1660.

*4-((1-(2-(4-(3-Hydroxy-4-oxo-4H-chromen-2-yl)phenoxy)ethyl)-1H-1,2,3-triazol-4-yl)methoxy)-2H-chromen-2-one* (**127**). A mixture of alkyne coumarin **48** (186 mg, 0.931 mmol), azide flavonol **79** (302 mg, 0.933 mmol), CuSO_4_·5H_2_O (318 mg, 1.27 mmol) and sodium ascorbate (497 mg, 2.51 mmol) in *t*-BuOH/H_2_O (1:1, 40 mL) was reacted according to GP-A. The crude residue was purified by flash column chromatography (SiO_2_, 1%–5% MeOH/CH_2_Cl_2_) and recrystallized from MeOH to afford triazole hybrid **127** (77.9 mg, 16%) as an off-white powdery solid. m.p. 228–230 °C. TLC *R*_f_ = 0.46 (5% MeOH/CH_2_Cl_2_). IR ν_max_ (neat)/cm^−1^: 3271w (O-H str), 3015w (C-H str), 2923w (C-H str), 1714s (C=O str), 1622m, 1603s, 1565s (C=C str), 1509m (C=C str), 1480m, 1470w, 1428m, 1406w, 1376w, 1262s, 1228m, 1177m, 1140w, 1106m, 1043m. ^1^H-NMR (500 MHz, CDCl_3_): δ 4.52 (2H, t, *J* = 5.0 Hz, -OCH_2_C*H*_2_N-), 4.89 (2H, t, *J* = 5.0 Hz, -OC*H*_2_CH_2_N-), 5.37 (2H, s, -OC*H*_2_CN-), 5.89 (1H, s, -C=CH), 6.97 (1H, br s, OH), 7.03 (2H, d, *J* = 9.0 Hz, ArH), 7.24 (1H, t, *J* = 8.0 Hz, ArH), 7.31 (1H, dd, *J* = 8.5, 1.0 Hz, ArH), 7.43 (1H, t, *J* = 8.0 Hz, ArH), 7.54 (1H, t, *J* = 8.5 Hz, ArH), 7.59 (1H, d, *J* = 8.5 Hz, ArH), 7.72 (1H, t, *J* = 8.5 Hz, ArH), 7.78 (1H, dd, *J* = 8.0, 1.5 Hz, ArH), 7.96 (1H, s, -C*H*N-), 8.23–8.27 (3H, m, ArH). ^13^C-NMR (500 MHz, CDCl_3_): δ 49.9, 62.6, 66.3, 91.2, 114.5, 115.4, 116.8, 118.2, 120.7, 123.1, 123.9, 124.5, 124.7, 124.8, 125.4, 129.7, 132.5, 133.5, 137.8, 141.7, 144.6, 153.3, 155.3, 159.0, 162.6, 164.9, 173.2. LCMS (ES+) *m*/*z* = 524.2 ([M + H]^+^, *t*_R_ = 1.55 min). HRMS (ESI+) *m*/*z* = 524.1476 [M + H]^+^ found, C_29_H_22_N_3_O_7_^+^ required 524.1458.

*4-(2-(4-(((4-Oxo-2-phenyl-4H-chromen-3-yl)oxy)methyl)-1H-1,2,3-triazol-1-yl)ethoxy)-2H-chromen-2-one* (**128**). A mixture of alkyne flavone **30** (310 mg, 1.12 mmol), azide coumarin **58** (256 mg, 1.11 mmol), CuSO_4_·5H_2_O (399 mg, 1.60 mmol) and sodium ascorbate (578 mg, 2.92 mmol) in *t*-BuOH/H_2_O (1:1, 40 mL) was reacted according to GP-A. The crude residue was purified by flash column chromatography (SiO_2_, 1%–5% MeOH/CH_2_Cl_2_) to afford triazole hybrid **128** (354 mg, 63%) as a white fluffy solid. m.p. 268–270 °C. TLC *R*_f_ = 0.30 (5% MeOH/CH_2_Cl_2_). IR ν_max_ (neat)/cm^−1^: 3082w (C-H str), 2926w (C-H str), 1723s (C=O str), 1626s, 1567m (C=C str), 1494w, 1465m, 1400w, 1385m, 1274m, 1239s, 1197s, 1182s, 1148s, 1140m, 1111m, 1058w, 1030w. ^1^H-NMR (500 MHz, CDCl_3_): δ 4.47 (2H, t, *J* = 5.0 Hz, -OCH_2_C*H*_2_N-), 4.85 (2H, t, *J* = 5.0 Hz, -OC*H*_2_CH_2_N-), 5.31 (2H, s, -OC*H*_2_CN-), 5.65 (1H, s, -C=CH), 7.24 (1H, t, *J* = 7.5 Hz, ArH, overlain by CDCl_3_), 7.33 (1H, d, *J* = 8.5 Hz, ArH), 7.41–7.46 (4H, m, ArH), 7.51 (1H, d, *J* = 8.5 Hz, ArH), 7.55 (1H, t, *J* = 8.0 Hz, ArH), 7.68–7.73 (2H, m, ArH), 7.93 (1H, s, -C*H*N-), 8.02–8.04 (2H, m, ArH), 8.25 (1H, d, *J* = 7.5 Hz, ArH). ^13^C-NMR (500 MHz, CDCl_3_): δ 48.7, 65.2, 67.0, 91.1, 115.0, 116.8, 118.1, 122.9, 124.0, 124.2, 124.7, 124.9, 125.6, 128.4, 128.7, 130.7, 130.8, 132.8, 133.7, 139.7, 144.7, 153.3, 155.3, 156.3, 162.2, 164.6, 175.1. LCMS (ES+) *m*/*z* = 508.3 ([M + H]^+^, *t*_R_ = 1.81 min). HRMS (ESI+) *m*/*z* = 508.1506 [M + H]^+^ found, C_29_H_22_N_3_O_6_^+^ required 508.1509.

*(Z)-4-((1-(2-(4-((6-Methoxy-3-oxobenzofuran-2(3H)-ylidene)methyl)phenoxy)ethyl)-1H-1,2,3-triazol-4-yl)methoxy)-2H-chromen-2-one* (**129**). A mixture of alkyne coumarin **48** (181 mg, 0.902 mmol), azide aurone **89** (302 mg, 0.895 mmol), CuSO_4_·5H_2_O (250 mg, 1.00 mmol) and sodium ascorbate (459 mg, 2.32 mmol) in *t*-BuOH/H_2_O (1:1, 40 mL) was reacted according to GP-A. The crude residue was purified by flash column chromatography (SiO_2_, 1%–5% MeOH/CH_2_Cl_2_) and recrystallized from MeOH to afford triazole hybrid **129** (253 mg, 53%) as a bright yellow-orange microcrystalline solid. m.p. 138–140 °C. TLC *R*_f_ = 0.40 (5% MeOH/CH_2_Cl_2_). IR ν_max_ (neat)/cm^−1^: 3077w (C-H str), 2926w (C-H str), 1719s (C=O str), 1650m, 1623m, 1593s (C=C str), 1565m (C=C str), 1511m (C=C str), 1442m, 1399m, 1269m, 1247s, 1182s, 1130s, 1096s, 1042m, 1019m. ^1^H-NMR (500 MHz, CDCl_3_): δ 3.94 (3H, s, -OCH_3_), 4.48 (2H, t, *J* = 4.5 Hz, -OCH_2_C*H*_2_N-), 4.87 (2H, t, *J* = 4.5 Hz, -OC*H*_2_CH_2_N-), 5.36 (2H, s, -OC*H*_2_CN-), 5.88 (1H, s, -C=CH), 6.75–6.78 (2H, m, ArH), 6.76 (1H, s, -C=CH), 6.94 (2H, d, *J* = 9.0 Hz, ArH), 7.23 (1H, t, *J* = 8.0 Hz, ArH), 7.31 (1H, dd, *J* = 8.5, 1.0 Hz, ArH), 7.53 (1H, t, *J* = 8.5 Hz, ArH), 7.71 (1H, d, *J* = 9.0 Hz, ArH), 7.77 (1H, dd, *J* = 8.0, 1.5 Hz, ArH), 7.84 (2H, d, *J* = 8.5 Hz, ArH), 7.95 (1H, s, -C*H*N-). ^13^C-NMR (500 MHz, CDCl_3_): δ 49.9, 56.0, 62.5, 66.3, 91.2, 96.6, 111.3, 112.1, 114.8, 115.0, 115.4, 116.8, 123.1, 123.9, 124.7, 125.8, 126.3, 132.5, 133.1, 141.6, 147.1, 153.3, 158.6, 162.6, 164.9, 167.3, 168.3, 182.8. LCMS (ES+) *m*/*z* = 538.2 ([M + H]^+^, *t*_R_ = 1.78 min). HRMS (ESI+) *m*/*z* = 538.1617 [M + H]^+^ found, C_30_H_24_N_3_O_7_^+^ required 538.1614.

*4-(2-(4-(((3-(4-Methoxyphenyl)-4-oxo-4H-chromen-7-yl)oxy)methyl)-1H-1,2,3-triazol-1-yl)ethoxy)-2H-chromen-2-one* (**130**). A mixture of alkyne isoflavone **46** (301 mg, 0.983 mmol), azide coumarin **58** (232 mg, 1.00 mmol), CuSO_4_·5H_2_O (288 mg, 1.15 mmol) and sodium ascorbate (503 mg, 2.54 mmol) in *t*-BuOH/H_2_O (1:1, 40 mL) was reacted according to GP-A. The crude residue was purified by flash column chromatography (SiO_2_, 1%–5% MeOH/CH_2_Cl_2_) to afford triazole hybrid **130** (363 mg, 69%) as an off-white powdery solid. m.p. 208–210 °C. TLC *R*_f_ = 0.28 (5% MeOH/CH_2_Cl_2_). IR ν_max_ (neat)/cm^−1^: 3085w (C-H str), 2927w (C-H str), 1730s (C=O str), 1621s, 1566m (C=C str), 1513m (C=C str), 1495w, 1444m, 1379m, 1330w, 1279w, 1241s, 1198m, 1181s, 1144m, 1109m, 1031m. ^1^H-NMR (500 MHz, CDCl_3_): δ 3.85 (3H, s, -OCH_3_), 4.58 (2H, t, *J* = 4.4 Hz, -OCH_2_C*H*_2_N-), 4.95 (2H, t, *J* = 4.4 Hz, -OC*H*_2_CH_2_N-), 5.35 (2H, s, -OC*H*_2_CN-), 5.69 (1H, s, -C=CH), 6.97–6.99 (3H, m, ArH), 7.03 (1H, dd, *J* = 8.8, 2.0 Hz, ArH), 7.23 (1H, d, *J* = 7.6 Hz, ArH), 7.31 (1H, d, *J* = 8.0 Hz, ArH), 7.50 (2H, d, *J* = 8.4 Hz, ArH), 7.56 (1H, t, *J* = 8.0 Hz, ArH), 7.65 (1H, d, *J* = 8.0 Hz, ArH), 7.84 (1H, s, -C*H*N-), 7.90 (1H, s, -C=CH), 8.20 (1H, d, *J* = 8.8 Hz, ArH). ^13^C-NMR (500 MHz, CDCl_3_): δ 49.0, 55.3, 62.4, 67.0, 91.3, 101.2, 114.0, 114.8, 114.9, 117.0, 118.9, 120.2, 122.4, 123.5, 124.0, 124.1, 125.0, 128.0, 130.1, 132.9, 143.7, 152.1, 153.3, 157.7, 159.6, 162.2, 164.4, 175.7. LCMS (ES+) *m*/*z* = 538.0 ([M + H]^+^, *t*_R_ = 4.19 min). HRMS (ESI+) *m*/*z* = 538.1624 [M + H]^+^ found, C_30_H_24_N_3_O_7_^+^ required 538.1614.

*(E)-4-((1-(2-(4-(3-Oxo-3-(2-(prop-2-yn-1-yloxy)phenyl)prop-1-en-1-yl)phenoxy)ethyl)-1H-1,2,3-triazol-4-yl)methoxy)-2H-chromen-2-one* (**131**). A mixture of biflavonoid **126** (264 mg, 0.517 mmol), propargyl bromide (0.130 mL, 1.46 mmol) and anhydrous K_2_CO_3_ (291 mg, 2.10 mmol) in dry acetone (50 mL) was reacted according to GP-B. The crude residue was purified by flash column chromatography (SiO_2_, 1% MeOH/CH_2_Cl_2_) to afford alkyne biflavonoid **131** (229 mg, 81%) as a pale yellow-white powdery solid. m.p. 164–166 °C. TLC *R*_f_ = 0.15 (5% MeOH/CH_2_Cl_2_). IR ν_max_ (neat)/cm^−1^: 3239w (C≡C-H str), 3074w (C-H str), 2973w (C-H str), 2165w (C≡C str), 1723s (C=O str), 1651m, 1624m, 1600s (C=C str), 1566m (C=C str), 1510m (C=C str), 1482w, 1452m, 1401s, 1372m, 1328m, 1231s, 1175w, 1105m, 1028m. ^1^H-NMR (500 MHz, CDCl_3_): δ 2.54 (1H, t, *J* = 2.0 Hz, -OCH_2_C≡C*H*), 4.45 (2H, t, *J* = 4.8 Hz, -OCH_2_C*H*_2_N-), 4.79 (2H, d, *J* = 2.0 Hz, -OC*H*_2_C≡CH), 4.85 (2H, t, *J* = 4.8 Hz, -OC*H*_2_CH_2_N-), 5.35 (2H, s, -OC*H*_2_CN-), 5.87 (1H, s, -C=CH), 6.88 (2H, d, *J* = 8.8 Hz, ArH), 7.09–7.13 (2H, m, ArH), 7.23 (1H, t, *J* = 7.6 Hz, ArH), 7.29 (1H, d, *J* = 13.2 Hz, -C*H*=CHCO-, overlain by CDCl_3_), 7.32 (1H, t, *J* = 4.4 Hz, ArH), 7.49 (1H, t, *J* = 8.8 Hz, ArH), 7.53–7.58 (3H, m, ArH), 7.56 (1H, d, *J* = 15.6 Hz, -CH=C*H*CO-), 7.64 (1H, dd, *J* = 7.6, 1.2 Hz, ArH), 7.77 (1H, dd, *J* = 8.4, 0.8 Hz, ArH), 7.93 (1H, s, -C*H*N-). ^13^C-NMR (500 MHz, CDCl_3_): δ 49.9, 56.4, 62.5, 66.3, 76.1, 78.1, 91.2, 113.3, 114.8, 115.4, 116.8, 121.8, 123.1, 123.9, 124.6, 125.6, 129.0, 130.0, 130.2, 130.5, 132.6, 132.6, 141.6, 142.7, 153.3, 155.8, 159.3, 162.5, 164.9, 192.5. LCMS (ES+) *m*/*z* = 548.0 ([M + H]^+^, *t*_R_ = 4.38 min). HRMS (ESI+) *m*/*z* = 548.1803 [M + H]^+^ found, C_32_H_26_O_6_N_3_^+^ required 548.1816.

*(Z)-2-(4-((1-(2-(4-((3-Oxobenzofuran-2(3H)-ylidene)methyl)phenoxy)ethyl)-1H-1,2,3-triazol-4-yl)methoxy)phenyl)-3-(prop-2-yn-1-yloxy)-4H-chromen-4-one* (**132**). A mixture of biflavonoid **122** (212 mg, 0.354 mmol), propargyl bromide (0.063 mL, 0.707 mmol) and anhydrous K_2_CO_3_ (153 mg, 1.11 mmol) in dry acetone (20 mL) was reacted according to GP-B. The crude residue was purified by flash column chromatography (SiO_2_, 1% MeOH/CH_2_Cl_2_) to afford alkyne biflavonoid **132** (168 mg, 74%) as a bright yellow powdery solid. m.p. 150–152 °C. TLC *R*_f_ = 0.50 (5% MeOH/CH_2_Cl_2_). IR ν_max_ (neat)/cm^−1^: 3233w (C≡C-H str), 2939w (C-H str), 2115w (C≡C str), 1697s (C=O str), 1635m, 1606s, 1598s (C=C str), 1566m (C=C str), 1508s (C=C str), 1472m, 1395m, 1343w, 1301m, 1250s, 1198m, 1184s, 1148m, 1133w, 1047m, 1017w. ^1^H-NMR (500 MHz, CDCl_3_): δ 2.34 (1H, t, *J* = 2.4 Hz, -OCH_2_C≡C*H*), 4.46 (2H, t, *J* = 4.8 Hz, -OCH_2_C*H*_2_N-), 4.84 (2H, t, *J* = 4.8 Hz, -OC*H*_2_CH_2_N-), 5.00 (2H, d, *J* = 2.4 Hz, -OC*H*_2_C≡CH), 5.33 (2H, s, -OC*H*_2_CN-), 6.83 (1H, s, -C=CH), 6.93 (2H, d, *J* = 8.8 Hz, ArH), 7.12 (2H, d, *J* = 9.2 Hz, ArH), 7.22 (1H, t, *J* = 7.6 Hz, ArH), 7.31 (1H, d, *J* = 8.4 Hz, ArH), 7.38 (1H, t, *J* = 7.6 Hz, ArH), 7.51 (1H, d, *J* = 8.4 Hz, ArH), 7.63–7.69 (2H, m, ArH), 7.80 (1H, d, *J* = 7.6 Hz, ArH), 7.86 (2H, d, *J* = 6.8 Hz, ArH), 7.87 (1H, s, -C*H*N-), 8.16 (2H, d, *J* = 8.8 Hz, ArH), 8.22 (1H, dd, *J* = 8.0, 1.6 Hz, ArH). ^13^C-NMR (500 MHz, CDCl_3_): δ 49.8, 59.0, 62.0, 66.3, 76.0, 78.7, 112.6, 112.9, 114.6, 114.9, 117.9, 121.8, 123.4, 123.8, 123.9, 124.1, 124.6, 124.7, 125.7, 126.1, 130.7, 133.4, 136.7, 138.0, 146.1, 155.1, 156.4, 158.9, 160.1, 165.8, 174.7, 184.5. LCMS (ES+) *m*/*z* = 638.1 ([M + H]^+^, *t*_r_ = 4.82 min). HRMS (ESI+) *m*/*z* = 638.1902 [M + H]^+^ found, C_38_H_28_O_7_N_3_^+^ required 638.1922.

*(E)-1-(4-Methoxy-2-(prop-2-yn-1-yloxy)phenyl)-3-(4-methoxy-3-((1-(2-(2-methoxy-4-((E)-3-(2,4,6-trimethoxyphenyl)acryloyl)phenoxy)ethyl)-1H-1,2,3-triazol-4-yl)methoxy)phenyl)prop-2-en-1-one* (**133**). A mixture of biflavonoid **92** (111 mg, 0.148 mmol), propargyl bromide (0.050 mL, 0.561 mmol) and anhydrous K_2_CO_3_ (113 mg, 0.815 mmol) in dry acetone (50 mL) was reacted according to GP-B. The crude residue was purified by flash column chromatography (SiO_2_, 1% MeOH/CH_2_Cl_2_) to afford alkyne biflavonoid **133** (107 mg, 92%) as a bright yellow powdery solid. m.p. 170–172 °C. TLC *R*_f_ = 0.29 (5% MeOH/CH_2_Cl_2_). IR ν_max_ (neat)/cm^−1^: 3286w (C≡C-H str), 3002w (C-H str), 2936w (C-H str), 2841w (C-H str), 2160w (C≡C str), 1650m (C=O str), 1599s (C=C str), 1511m (C=C str), 1458m, 1441w, 1432w, 1418w, 1399w, 1377w, 1337m, 1321m, 1301m, 1259s, 1205w, 1159m, 1124m, 1027m. ^1^H-NMR (500 MHz, CDCl_3_): δ 2.64 (1H, t, *J* = 2.4 Hz, -OCH_2_C≡C*H*), 3.87 (6H, s, 2 × -OCH_3_), 3.89 (3H, s, -OCH_3_), 3.91 (9H, s, 3 × -OCH_3_), 4.45 (2H, t, *J* = 4.8 Hz, -OCH_2_C*H*_2_N-), 4.83 (2H, t, *J* = 4.8 Hz, -OC*H*_2_CH_2_N-), 4.84 (2H, d, *J* = 2.4 Hz, -OC*H*_2_C≡CH), 5.33 (2H, s, -OC*H*_2_CN-), 6.15 (2H, s, ArH), 6.61–6.63 (2H, m, ArH), 6.83 (1H, d, *J* = 8.0 Hz, ArH), 6.87 (1H, d, *J* = 8.0 Hz, ArH), 7.18 (1H, dd, *J* = 8.4, 2.0 Hz, ArH), 7.39 (1H, d, *J* = 1.6 Hz, ArH), 7.46 (1H, d, *J* = 15.6 Hz, -C*H*=CHCO-), 7.56–7.64 (2H, m, ArH), 7.61 (1H, d, *J* = 16.0 Hz, -CH=C*H*CO-), 7.79 (1H, d, *J* = 9.2 Hz, ArH), 7.85 (1H, d, *J* = 15.6 Hz, -C*H*=CHCO-), 8.03 (1H, s, -C*H*N-), 8.23 (1H, d, *J* = 16.0 Hz, -CH=C*H*CO-). ^13^C-NMR (500 MHz, CDCl_3_): δ 49.7, 55.6, 55.8, 55.9, 55.9, 56.5, 63.1, 67.5, 76.3, 78.1, 90.5, 100.1, 106.4, 106.6, 111.5, 111.7, 112.4, 112.8, 121.6, 122.3, 122.8, 124.0, 124.6, 125.3, 128.5, 132.9, 133.7, 135.6, 142.1, 144.1, 147.7, 149.5, 150.6, 151.5, 158.2, 158.3, 161.6, 163.0, 163.8, 190.0, 190.3. LCMS (ES+) *m*/*z* = 790.3 ([M + H]^+^, *t*_R_ = 4.71 min). HRMS (ESI+) *m*/*z* = 790.2949 [M + H]^+^ found, C_44_H_44_O_11_N_3_^+^ required 790.2970.

*2-(4-(2-(4-(((4-Oxo-2-phenyl-4H-chromen-3-yl)oxy)methyl)-1H-1,2,3-triazol-1-yl)ethoxy)phenyl)-3-(prop-2-yn-1-yloxy)-4H-chromen-4-one* (**134**). A mixture of biflavonoid **110** (161 mg, 0.268 mmol), propargyl bromide (0.045 mL, 0.500 mmol) and anhydrous K_2_CO_3_ (111 mg, 0.803 mmol) in dry acetone (20 mL) was reacted according to GP-B. The crude residue was purified by flash column chromatography (SiO_2_, 1% MeOH/CH_2_Cl_2_) to afford alkyne biflavonoid **134** (161 mg, 94%) as a pale yellow-white powdery solid. m.p. 118–120 °C. TLC *R*_f_ = 0.34 (5% MeOH/CH_2_Cl_2_). IR ν_max_ (neat)/cm^−1^: 3233w (C≡C-H str), 2939w (C-H str), 2116w (C≡C str), 1631s (C=O str), 1613s, 1600s, 1559m (C=C str), 1508m (C=C str), 1467s, 1392s, 1287w, 1252m, 1239m, 1184s, 1146m, 1123w, 1109w, 1042w. ^1^H-NMR (500 MHz, CDCl_3_): δ 2.33 (1H, t, *J* = 2.4 Hz, -OCH_2_C≡C*H*), 4.39 (2H, t, *J* = 5.2 Hz, -OCH_2_C*H*_2_N-), 4.73 (2H, t, *J* = 5.2 Hz, -OC*H*_2_CH_2_N-), 4.99 (2H, d, *J* = 2.4 Hz, -OC*H*_2_C≡CH), 5.33 (2H, s, -OC*H*_2_CN-) 6.97 (2H, d, *J* = 9.2 Hz, ArH), 7.39–7.45 (5H, m, ArH), 7.53 (1H, d, *J* = 8.0 Hz, ArH), 7.54 (1H, d, *J* = 8.0 Hz, ArH), 7.67–7.72 (2H, m, ArH), 7.85 (1H, s, -C*H*N-), 8.02–8.04 (2H, m, ArH), 8.15 (2H, d, *J* = 9.2 Hz, ArH), 8.25 (1H, dd, *J* = 8.0, 1.2 Hz, ArH), 8.28 (1H, dd, *J* = 8.0, 1.2 Hz, ArH). ^13^C-NMR (500 MHz, CDCl_3_): δ 49.5, 59.0, 65.1, 66.2, 76.1, 78.6, 114.3, 117.9, 118.1, 124.0, 124.1, 124.3, 124.7, 124.8, 124.9, 125.7, 128.3, 128.7, 130.7, 130.8, 133.4, 133.6, 138.1, 139.5, 144.1, 155.1, 155.3, 156.2, 156.4, 159.6, 174.7, 175.1. LCMS (ES+) *m*/*z* = 638.1 ([M + H]^+^, *t*_R_ = 4.70 min). HRMS (ESI+) *m*/*z* = 638.1905 [M + H]^+^ found, C_38_H_28_O_7_N_3_^+^ required 638.1922.

*(E)-2-(4-(2-(4-((4-(3-(Ferrocenyl)acryloyl)-2-methoxyphenoxy)methyl)-1H-1,2,3-triazol-1-yl)ethoxy)phenyl)-3-(prop-2-yn-1-yloxy)-4H-chromen-4-one* (**135**). A mixture of biflavonoid **106** (190 mg, 0.263 mmol), propargyl bromide (0.047 mL, 0.526 mmol) and anhydrous K_2_CO_3_ (127 mg, 0.920 mmol) in dry acetone (20 mL) was reacted according to GP-B. The crude residue was purified by flash column chromatography (SiO_2_, 1% MeOH/CH_2_Cl_2_) to afford alkyne biflavonoid **135** (95.5 mg, 48%) as a dark red powdery solid. m.p. 108–110 °C. TLC *R*_f_ = 0.37 (5% MeOH/CH_2_Cl_2_). IR ν_max_ (neat)/cm^−1^: 3292w (C≡C-H str), 2937w (C-H str), 2245w (C≡C str), 1636m (C=O str), 1598s (C=C str), 1576s (C=C str), 1509s (C=C str), 1467m, 1419m, 1395m, 1349w, 1258s, 1198m, 1184m, 1150m, 1029w, 1000w. ^1^H-NMR (500 MHz, CDCl_3_): δ 2.33 (1H, t, *J* = 2.5 Hz, -OCH_2_C≡C*H*), 3.96 (3H, s, -OCH_3_), 4.17 (5H, s, -C_5_H_5_), 4.45–4.47 (4H, m, -C_5_H_4_ and -OCH_2_C*H*_2_N-), 4.57 (2H, t, *J* = 2.0 Hz, -C_5_H_4_), 4.82 (2H, t, *J* = 5.0 Hz, -OC*H*_2_CH_2_N-), 4.98 (2H, d, *J* = 2.5 Hz, -OC*H*_2_C≡CH), 5.42 (2H, s, -OC*H*_2_CN-), 6.96 (2H, d, *J* = 9.0 Hz, ArH), 7.13 (1H, d, *J* = 15.5 Hz, -C*H*=CHCO-), 7.15 (1H, d, *J* = 8.5 Hz, ArH), 7.41 (1H, t, *J* = 8.5 Hz, ArH), 7.54 (1H, dd, *J* = 8.5, 0.5 Hz, ArH), 7.58 (1H, dd, *J* = 8.5, 2.0 Hz, ArH), 7.63 (1H, d, *J* = 2.0 Hz, ArH), 7.69 (1H, t, *J* = 8.5 Hz, ArH), 7.73 (1H, d, *J* = 15.0 Hz, -CH=C*H*CO-), 7.90 (1H, s, -C*H*N-), 8.14 (2H, d, *J* = 9.0 Hz, ArH), 8.25 (1H, dd, *J* = 8.0, 1.5 Hz, ArH). ^13^C-NMR (500 MHz, CDCl_3_): δ 49.7, 56.1, 59.0, 62.8, 66.2, 68.9, 69.7, 71.2, 76.1, 78.6, 79.3, 111.2, 112.3, 114.2, 117.9, 118.5, 122.4, 124.0, 124.3, 124.4, 124.7, 125.7, 130.8, 132.3, 133.4, 138.1, 143.8, 146.0, 149.5, 151.3, 155.1, 156.1, 159.5, 174.7, 187.9. LCMS (ES+) *m*/*z* = 762.1 ([M + H]^+^, *t*_R_ = 4.88 min). HRMS (ESI+) *m*/*z* = 762.1878 [M + H]^+^ found, C_43_H_36_O_7_N_3_Fe^+^ required 762.1897.

*(E)-4-((1-(2-(4-(3-Oxo-3-(2-((1-(2-((4-oxo-2-phenyl-4H-chromen-7-yl)oxy)ethyl)-1H-1,2,3-triazol-4-yl)methoxy)phenyl)prop-1-en-1-yl)phenoxy)ethyl)-1H-1,2,3-triazol-4-yl)methoxy)-2H-chromen-2-one* (**136**). A mixture of alkyne biflavonoid **131** (205 mg, 0.375 mmol), azide flavone **86** (123 mg, 0.399 mmol), CuSO_4_·5H_2_O (115 mg, 0.459 mmol) and sodium ascorbate (192 mg, 0.969 mmol) in *t*-BuOH/H_2_O (1:1, 40 mL) was reacted according to GP-A. The crude residue was purified by flash column chromatography (SiO_2_, 1%–5% MeOH/CH_2_Cl_2_) to afford triazole hybrid **136** (214 mg, 67%) as a pale yellow-white powdery solid. m.p. 78–80 °C. TLC *R*_f_ = 0.37 (7% MeOH/CH_2_Cl_2_). IR ν_max_ (neat)/cm^−1^: 3080w (C-H str), 2923w (C-H str), 1708s (C=O str), 1622s, 1597s (C=C str), 1567s (C=C str), 1509w (C=C str), 1493w, 1449m, 1356m, 1274w, 1237m, 1173m, 1139w, 1105w, 1043w, 1026w. ^1^H-NMR (500 MHz, CDCl_3_): δ 4.38 (2H, t, *J* = 5.2 Hz, -OCH_2_C*H*_2_N-), 4.41 (2H, t, *J* = 5.2 Hz, -OCH_2_C*H*_2_N-), 4.70 (2H, t, *J* = 5.2 Hz, -OC*H*_2_CH_2_N-), 4.82 (2H, t, *J* = 5.2 Hz, -OC*H*_2_CH_2_N-), 5.34 (2H, s, -OC*H*_2_CN-), 5.34 (2H, s, -OC*H*_2_CN-), 5.85 (1H, s, -C=CH), 6.75 (1H, s, -C=CH), 6.77–6.80 (3H, m, ArH), 6.85 (1H, d, *J* = 2.4 Hz, ArH), 7.08 (1H, t, *J* = 7.6 Hz, ArH), 7.14 (1H, d, *J* = 8.4 Hz, ArH), 7.20 (1H, t, *J* = 8.0 Hz, ArH), 7.26 (1H, d, *J* = 16.0 Hz, -C*H*=CHCO-, overlain by CDCl_3_), 7.28 (1H, d, *J* = 4.0 Hz, ArH), 7.38 (2H, d, *J* = 8.4 Hz, ArH), 7.46–7.54 (5H, m, ArH), 7.50 (1H, d, *J* = 16.0 Hz, -CH=C*H*CO-), 7.62 (1H, dd, *J* = 8.8, 1.6 Hz, ArH), 7.74 (1H, dd, *J* = 8.0, 1.2 Hz, ArH), 7.77 (1H, s, -C*H*N-), 7.89 (2H, dd, *J* = 7.6, 1.6 Hz, ArH), 7.96 (1H, s, -C*H*N-), 8.03 (1H, d, *J* = 8.8 Hz, ArH). ^13^C-NMR (500 MHz, CDCl_3_): δ 49.4, 49.8, 62.5, 62.8, 66.2, 66.6, 91.2, 101.4, 107.5, 113.1, 114.1, 114.8, 115.4, 116.7, 118.5, 121.5, 123.1, 123.9, 124.0, 124.8, 125.7, 126.2, 127.3, 128.7, 129.1, 129.8, 130.1, 130.4, 131.6, 131.6, 132.5, 132.9, 141.6, 142.4, 144.1, 153.3, 156.5, 157.6, 159.3, 161.9, 162.5, 163.2, 164.9, 177.6, 192.6. LCMS (ES+) *m*/*z* = 855.3 ([M + H]^+^, *t*_R_ = 4.57 min). HRMS (ESI+) *m*/*z* = 855.2743 [M + H]^+^ found, C_49_H_39_O_9_N_6_^+^ required 855.2773.

*(Z)-7-(2-(4-(((4-Oxo-2-(4-((1-(2-(4-((3-oxobenzofuran-2(3H)-ylidene)methyl)phenoxy)ethyl)-1H-1,2,3-triazol-4-yl)methoxy)phenyl)-4H-chromen-3-yl)oxy)methyl)-1H-1,2,3-triazol-1-yl)ethoxy)-2-phenyl-4H-chromen-4-one* (**137**). A mixture of alkyne biflavonoid **132** (145 mg, 0.227 mmol), azide flavone **86** (73.5 mg, 0.239 mmol), CuSO_4_·5H_2_O (70.1 mg, 0.281 mmol) and sodium ascorbate (129 mg, 0.651 mmol) in *t*-BuOH/H_2_O (1:1, 40 mL) was reacted according to GP-A. The crude residue was purified by flash column chromatography (SiO_2_, 1%–5% MeOH/CH_2_Cl_2_) to afford triazole hybrid **137** (106 mg, 49%) as a bright yellow powdery solid. m.p. 198–200 °C. TLC *R*_f_ = 0.33 (7% MeOH/CH_2_Cl_2_). IR ν_max_ (neat)/cm^−1^: 2931w (C-H str), 2874w (C-H str), 1698w, 1628s (C=O str), 1602s, 1594s (C=C str), 1508s (C=C str), 1466m, 1449m, 1423w, 1396w, 1374m, 1299w, 1288w, 1249s, 1178s, 1148w, 1128w, 1110w, 1096w, 1041m. ^1^H-NMR (500 MHz, CDCl_3_): δ 4.44–4.46 (4H, m, 2 × -OCH_2_C*H*_2_N-), 4.78 (2H, t, *J* = 4.8 Hz, -OCH_2_C*H*_2_N-), 4.83 (2H, t, *J* = 4.8 Hz, -OC*H*_2_CH_2_N-), 5.28 (2H, s, -OC*H*_2_CN-), 5.33 (2H, s, -OC*H*_2_CN-), 6.75 (1H, s, -C=CH), 6.81 (1H, s, -C=CH), 6.91–6.94 (4H, m, ArH), 7.04 (2H, d, *J* = 9.2 Hz, ArH), 7.21 (1H, d, *J* = 7.6 Hz, ArH), 7.30 (1H, d, *J* = 8.4 Hz, ArH), 7.37 (1H, d, *J* = 7.6 Hz, ArH), 7.47–7.53 (4H, m, ArH), 7.62–7.67 (2H, m, ArH), 7.79 (1H, d, *J* = 7.6 Hz, ArH), 7.84 (2H, d, *J* = 8.8 Hz, ArH), 7.87–7.90 (3H, m, -C*H*N- and ArH), 7.94 (1H, s, -C*H*N-), 8.04 (2H, d, *J* = 8.8 Hz, ArH), 8.11 (1H, d, *J* = 8.8 Hz, ArH), 8.23 (1H, dd, *J* = 8.0, 1.2 Hz, ArH). ^13^C-NMR (500 MHz, CDCl_3_): δ 49.3, 49.7, 61.9, 65.0, 66.3, 66.7, 101.5, 107.6, 112.7, 112.9, 114.2, 114.7, 114.9, 117.9, 118.5, 121.8, 123.4, 123.6, 124.0, 124.1, 124.6, 124.7, 125.0, 125.6, 126.1, 126.2, 127.4, 129.0, 130.5, 131.5, 131.7, 133.4, 133.4, 136.7, 139.0, 143.7, 144.3, 146.1, 155.1, 156.1, 157.7, 159.0, 160.0, 162.1, 163.2, 165.9, 174.9, 177.7, 184.5. LCMS (ES+) *m*/*z* = 945.4 ([M + H]^+^, *t*_R_ = 4.89 min). HRMS (ESI+) *m*/*z* = 945.2851 [M + H]^+^ found, C_55_H_41_O_10_N_6_^+^ required 945.2879.

*7-Methoxy-2-(4-(2-(4-((5-methoxy-2-((E)-3-(4-methoxy-3-((1-(2-(2-methoxy-4-((E)-3-(2,4,6-trimethoxyphenyl)acryloyl)phenoxy)ethyl)-1H-1,2,3-triazol-4-yl)methoxy)phenyl)acryloyl)phenoxy)methyl)-1H-1,2,3-triazol-1-yl)ethoxy)phenyl)-4H-chromen-4-one* (**138**). A mixture of alkyne biflavonoid **133** (107 mg, 0.135 mmol), azide flavone **87** (49.3 mg, 0.146 mmol), CuSO_4_·5H_2_O (66.0 mg, 0.264 mmol) and sodium ascorbate (72.8 mg, 0.367 mmol) in *t*-BuOH/H_2_O (1:1, 40 mL) was reacted according to GP-A. The crude residue was purified by flash column chromatography (SiO_2_, 1%–5% MeOH/CH_2_Cl_2_) to afford triazole hybrid **138** (8.00 mg, 5%) as a pale yellow-white powdery solid. m.p. 128–130 °C. TLC *R*_f_ = 0.19 (5% MeOH/CH_2_Cl_2_). IR ν_max_ (neat)/cm^−1^: 2927w (C-H str), 2846w (C-H str), 1736w, 1627m (C=O str), 1600s (C=C str), 1510s (C=C str), 1454m, 1439m, 1423m, 1375w, 1355w, 1338w, 1258s, 1204m, 1162m, 1123s, 1023s. ^1^H-NMR (500 MHz, DMSO-*d*_6_): δ 3.76 (6H, s, 2 × -OCH_3_), 3.85 (6H, s, 2 × -OCH_3_), 3.90 (9H, s, 3 × -OCH_3_), 4.38 (2H, t, *J* = 4.4 Hz, -OCH_2_C*H*_2_N-), 4.50 (2H, t, *J* = 4.8 Hz, -OCH_2_C*H*_2_N-), 4.71 (2H, t, *J* = 4.8 Hz, -OC*H*_2_CH_2_N-), 4.82 (2H, t, *J* = 4.8 Hz, -OC*H*_2_CH_2_N-), 5.24 (2H, s, -OC*H*_2_CN-), 5.38 (2H, s, -OC*H*_2_CN-), 6.29 (2H, s, ArH), 6.57–6.66 (1H, m, ArH), 6.81 (1H, s, -C=CH), 6.82–6.85 (1H, m, ArH), 6.92–6.98 (3H, m, ArH), 7.02–7.11 (3H, m, ArH), 7.20 (1H, d, *J* = 8.8 Hz, ArH), 7.27 (1H, dd, *J* = 5.6, 2.4 Hz, ArH), 7.47 (1H, d, *J* = 15.6 Hz, -C*H*=CHCO-), 7.48 (1H, d, *J* = 8.0 Hz, ArH), 7.54–7.64 (2H, m, ArH), 7.56 (1H, d, *J* = 15.2 Hz, -CH=C*H*CO-), 7.82 (1H, d, *J* = 16.0 Hz, -C*H*=CHCO-), 7.87–8.06 (3H, m, ArH and -C*H*N-), 8.02 (1H, d, *J* = 16.0 Hz, -CH=C*H*CO-), 8.28 (1H, d, *J* = 6.4 Hz, ArH), 8.31 (1H, s, -C*H*N-). ^13^C-NMR (500 MHz, DMSO-*d*_6_): δ 48.9, 49.0, 55.5, 55.7, 56.0, 61.5, 61.7, 66.2, 67.1, 91.0, 99.8, 100.9, 105.1, 105.4, 106.6, 111.0, 111.3, 111.8, 112.0, 112.7, 114.5, 114.9, 114.9, 115.0, 115.0, 117.1, 120.2, 122.1, 123.8, 125.3, 126.1, 127.6, 127.9, 128.0, 132.0, 132.1, 134.4, 137.4, 141.4, 142.3, 142.7, 147.7, 148.9, 149.8, 151.0, 151.1, 157.4, 157.9, 158.6, 160.4, 161.3, 162.0, 163.2, 163.7, 176.3, 188.3, 189.1. LCMS (ES+) *m*/*z* = 1128.0 ([M + H]^+^, *t*_R_ = 1.87 min). HRMS (ESI+) *m*/*z* = 1127.4014 [M + H]^+^ found, C_62_H_59_O_15_N_6_^+^ required 1127.4033.

*(Z)-3-((1-(2-(4-((6-Methoxy-3-oxobenzofuran-2(3H)-ylidene)methyl)phenoxy)ethyl)-1H-1,2,3-triazol-4-yl)methoxy)-2-(4-(2-(4-(((4-oxo-2-phenyl-4H-chromen-3-yl)oxy)methyl)-1H-1,2,3-triazol-1-yl)ethoxy)phenyl)-4H-chromen-4-one* (**139**). A mixture of alkyne biflavonoid **134** (134 mg, 0.210 mmol), azide aurone **89** (83.5 mg, 0.248 mmol), CuSO_4_·5H_2_O (70.8 mg, 0.284 mmol) and sodium ascorbate (131 mg, 0.659 mmol) in *t*-BuOH/H_2_O (1:1, 40 mL) was reacted according to GP-A. The crude residue was purified by flash column chromatography (SiO_2_, 1%–5% MeOH/CH_2_Cl_2_) to afford triazole hybrid **139** (73.9 mg, 36%) as a bright yellow powdery solid. m.p. 98–100 °C. TLC *R*_f_ = 0.39 (7% MeOH/CH_2_Cl_2_). IR ν_max_ (neat)/cm^−1^: 3001w (C-H str), 2929w (C-H str), 2874w (C-H str), 1693m (C=O str), 1632m, 1600s (C=C str), 1509m (C=C str), 1466m, 1444w, 1395m, 1343w, 1248s, 1181s, 1146m, 1129m, 1110m, 1096w, 1044m. ^1^H-NMR (500 MHz, CDCl_3_): δ 3.93 (3H, s, -OCH_3_), 4.34–4.37 (4H, m, 2 × -OCH_2_C*H*_2_N-), 4.69–4.72 (4H, m, 2 × -OC*H*_2_CH_2_N-), 5.32 (4H, s, 2 × -OC*H*_2_CN-), 6.77 (1H, d, *J* = 5.6 Hz, ArH), 6.77 (1H, s, -C=CH), 6.88 (2H, d, *J* = 8.8 Hz, ArH), 6.90 (2H, d, *J* = 8.8 Hz, ArH), 7.40–7.45 (6H, m, ArH), 7.51 (1H, d, *J* = 3.6 Hz, ArH), 7.53 (1H, d, *J* = 3.6 Hz, ArH), 7.67–7.72 (3H, m, ArH), 7.81 (2H, d, *J* = 9.2 Hz, ArH), 7.84 (1H, s, -C*H*N-), 7.88 (1H, s, -C*H*N-), 8.01–8.05 (4H, m, ArH), 8.27 (2H, dd, *J* = 8.0, 1.6 Hz, ArH). ^13^C-NMR (500 MHz, CDCl_3_): δ 49.4, 49.5, 56.0, 64.9, 65.2, 66.2, 66.3, 96.6, 111.5, 112.1, 114.3, 114.9, 115.0, 118.0, 118.1, 124.0, 124.1, 124.1, 124.8, 124.8, 124.9, 125.0, 125.7, 125.7, 125.7, 126.1, 128.4, 128.7, 130.6, 130.7, 133.1, 133.4, 133.6, 139.0, 139.6, 144.0, 144.1, 147.0, 155.2, 155.3, 156.0, 156.4, 158.8, 159.5, 167.3, 168.3, 175.0, 175.1, 182.8. LCMS (ES+) *m*/*z* = 975.4 ([M + H]^+^, *t*_R_ = 4.94 min). HRMS (ESI+) *m*/*z* = 975.2956 [M + H]^+^ found, C_56_H_43_O_11_N_6_^+^ required 975.2984.

*2-(4-(2-(4-((4-((E)-3-(Ferrocenyl)acryloyl)-2-methoxyphenoxy)methyl)-1H-1,2,3-triazol-1-yl)ethoxy)phenyl)-3-((1-(2-(4-(((Z)-6-methoxy-3-oxobenzofuran-2(3H)-ylidene)methyl)phenoxy)ethyl)-1H-1,2,3-triazol-4-yl)methoxy)-4H-chromen-4-one* (**140**). A mixture of alkyne biflavonoid **135** (71.6 mg, 0.0940 mmol), azide aurone **89** (38.1 mg, 0.113 mmol), CuSO_4_·5H_2_O (46.1 mg, 0.185 mmol) and sodium ascorbate (58.0 mg, 0.293 mmol) in *t*-BuOH/H_2_O (1:1, 40 mL) was reacted according to GP-A. The crude residue was purified by flash column chromatography (SiO_2_, 1%–5% MeOH/CH_2_Cl_2_) and further purified by preparative HPLC to afford triazole hybrid **140** (2.80 mg, 3%) as a bright red powdery solid. m.p. 158–160 °C. TLC *R*_f_ = 0.35 (7% MeOH/CH_2_Cl_2_). IR ν_max_ (neat)/cm^−1^: 2917w (C-H str), 2852w (C-H str), 1737w, 1605s (C=O str), 1509m (C=C str), 1465m, 1443w, 1378w, 1353w, 1260s, 1198m, 1151m, 1133w, 1113w, 1042w, 1015w. ^1^H-NMR (500 MHz, CDCl_3_): δ 3.94 (3H, s, -OCH_3_), 3.96 (3H, s, -OCH_3_), 4.17 (5H, br s, -C_5_H_5_), 4.38 (2H, t, *J* = 5.0 Hz, -OCH_2_C*H*_2_N-), 4.42 (2H, t, *J* = 5.0 Hz, -OCH_2_C*H*_2_N-), 4.47 (2H, br s, -C_5_H_4_), 4.57 (2H, br s, -C_5_H_4_), 4.73 (2H, t, *J* = 5.0 Hz, -OC*H*_2_CH_2_N-), 4.80 (2H, t, *J* = 4.5 Hz, -OC*H*_2_CH_2_N-), 5.30 (2H, s, -OC*H*_2_CN-), 5.41 (2H, s, -OC*H*_2_CN-), 6.76–6.79 (2H, m, ArH and -C=CH), 6.77 (1H, d, *J* = 14.5 Hz, -C*H*=CHCO-), 6.89 (2H, d, *J* = 9.0 Hz, ArH), 6.90 (2H, d, *J* = 8.5 Hz, ArH), 7.14 (1H, d, *J* = 8.0 Hz, ArH), 7.43 (1H, t, *J* = 8.0 Hz, ArH), 7.53 (1H, d, *J* = 8.5 Hz, ArH), 7.58 (1H, d, *J* = 8.2 Hz, ArH), 7.62 (1H, s, ArH), 7.70 (1H, t, *J* = 8.5 Hz, ArH), 7.70 (1H, d, *J* = 16.0 Hz, -CH=C*H*CO-), 7.71 (2H, d, *J* = 8.5 Hz, ArH), 7.84 (2H, d, *J* = 9.0 Hz, ArH), 7.89 (1H, s, -C*H*N-), 7.94 (1H, s, -C*H*N-), 8.05 (2H, d, *J* = 9.0 Hz, ArH), 8.27 (1H, dd, *J* = 8.0, 1.5 Hz, ArH). ^13^C-NMR (500 MHz, CDCl_3_): δ 49.6, 49.7, 56.1, 61.2, 62.8, 66.2, 66.3, 68.9, 69.8, 71.3, 79.3, 96.6, 111.2, 111.6, 111.8, 112.1, 112.3, 114.3, 114.3, 114.9, 118.0, 118.6, 121.3, 122.5, 122.5, 124.1, 124.1, 124.3, 124.4, 124.8, 125.1, 125.7, 125.7, 125.8, 130.6, 133.0, 133.2, 133.5, 138.3, 144.0, 146.0, 149.6, 151.4, 155.1, 157.4, 158.8, 159.5, 167.5, 167.7, 179.2, 182.9, 187.9. LCMS (ES+) *m*/*z* = 1098.8 ([M + H]^+^, *t*_R_ = 2.02 min). HRMS (ESI+) *m*/*z* = 1099.2960 [M + H]^+^ found, C_61_H_51_FeN_6_O_11_^+^ required 1099.2921.

### 3.4. Biological Screening

#### 3.4.1. Aβ Preparation

Aβ_**42**_ (1 mg) was purchased from Eurogentec Ltd. (Hampshire, UK) as a lyophilised powder. The peptide was dissolved in trifluroacetic acid (TFA, 1 mL), sonicated in an ice-water bath for 60 s, then the TFA removed in a vacuum desiccator. Ice cold 1,1,1,3,3,3-hexafluro-2-propanol (HFIP, 1 mL) was added to re-suspend the lyophilised peptide. The sample was sonicated for 60 s at 0 °C, then aliquoted into 20 μL portions. The HFIP was removed in the vacuum desiccator overnight and the lyophilised samples were stored at −80 °C until use. The required concentration of Aβ_42_ was prepared by dissolving the sample in dimethyl sulfoxide (DMSO) (5% of total solvent volume), then adding sodium phosphate buffer (50 mM, pH 7.4). The solution was sonicated at 0 °C for 3 min, then centrifuged at 13,400 rpm for 30 min at 0 °C to separate any aggregated species. 

#### 3.4.2. Thioflavin T (THT) Assay

ThT was purchased from AbCam (Cambridge, UK). Final concentrations of 10 μM Aβ_42_, 20 μM ThT and 50 μM compound in sodium phosphate buffer (50 mM, pH 7.4) were used for all samples. The assay samples (100 μL) were mixed in a black non-binding 96-well plate (Greiner Bio-One, Stonehouse, UK) which was sealed (Nunc™ polyolefin acrylate film Nunc, ThermoFisher) and loaded into the fluorescence plate reader (Tecan, Männedorf, Switzerland) at 37 °C. Fluorescence kinetics were measured at 5 min reading intervals, with 15 s shaking before each read. The excitation and emission wavelengths were 440 and 480 nm respectively.

## 4. Conclusions

Herein, we have described a highly modular branching-type strategy for the synthesis of biologically interesting and rare triazole-linked flavonoid dimers and trimers by the varied combination of readily-accessible flavonoid building blocks. Application of this strategy enabled concise and highly step-efficient access to a structurally diverse library of 46 final compounds, with six different biologically-relevant flavonoid structural subclasses (chalcone, flavonol, aurone, flavone, coumarin and isoflavone) successfully incorporated into the library. Each library member features structural motifs that are associated with biological activity (at least two flavonoid units and a 1,2,3-triazole linkage) and many also incorporate additional potential biomolecular-interacting elements (for example, hydrogen-bonding motifs). Many library compounds also feature groups that could provide synthetic handles for further elaboration or diversification. The synthetic strategy could conceivably be applied on a larger scale using a greater range of building blocks. However, this current strategy is limited to the installation of one linker type between the flavonoid units. It may be possible to adapt the strategy to allow for greater variation in the linker motif (for example, the use of an alternate type of building block may allow the alkyl chain length to be varied and 1,5-triazole linkages could conceivably be accessed though ruthenium-mediated ‘click’ cycloaddition conditions). Such variety may be of value in the context of biological screening; for example, previous studies of flavonoid dimers have suggested that linker length variation had a significant effect upon biological activity [14,16]. Preliminary biological screening of a representative sub-set of compounds has revealed that a selection of the triazole-linked dimers exhibit moderate inhibitory activity against the aggregation of Aβ_**42**_, a process closely linked with the development of Alzheimer’s disease. Such findings prompt for continued screening of the entire library and further study of the active scaffolds identified. Milligram (typically multimilligram) quantities of most final library compounds were isolated, which should provide ample material for screening in a wider range of biological assays; the systematic modification of any compounds with interesting properties should be facilitated by the conciseness and inherent modularity of the synthetic strategy [55]. More detailed biological assessment of the compound library is currently ongoing and notable results will be reported in due course.

## Supplementary Materials

Supplementary materials can be accessed at: http://www.mdpi.com/1420-3049/21/9/1230/s1.
