# Supplementary material for: Combinatorial Synthesis of Structurally Diverse Triazole-Bridged Flavonoid Dimers and Trimers
Source: Molecules. 2016 Sep 16;21(9):1230. doi: 10.3390/molecules21091230 (PMC6273872; doi:10.3390/molecules21091230)
Supplement: Supplementary file 1 [file molecules-21-01230-s001.pdf]

Supplementary Materials: Combinatorial Synthesis of Structurally Diverse Triazole-bridged Flavonoid Dimers and Trimers

Tze Han Sum, Tze Jing Sum, Warren R. J. D. Galloway, Súil Collins, David G. Twigg, Florian Hollfelder and David R. Spring

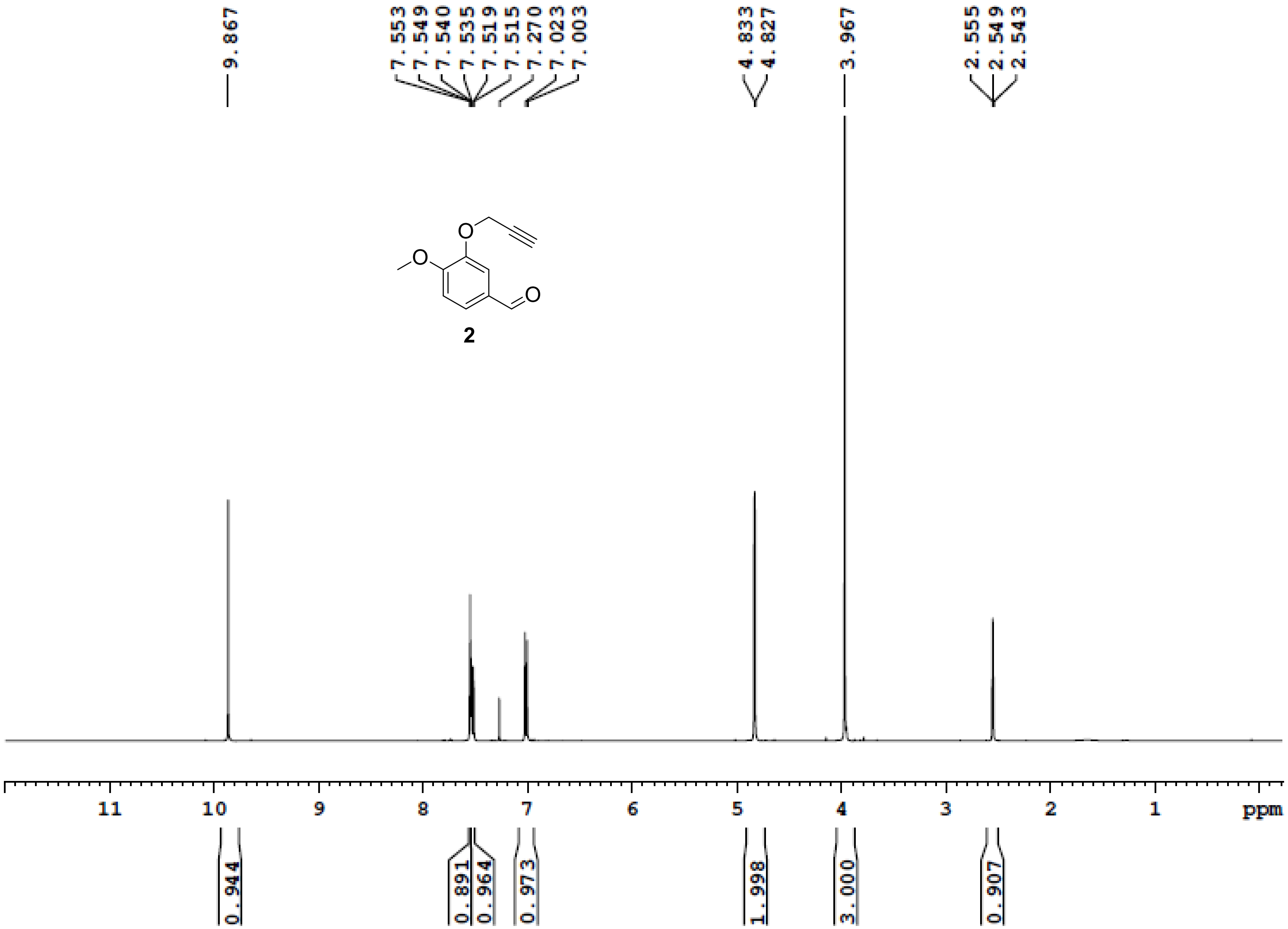
9.867
7.553
7.549
7.540
7.535
7.519
7.515
7.270
7.023
7.003
4.833
4.827
3.967
2.555
2.549
2.543
O
O
O
2
11
10
9
8
7
6
5
4
3
2
1
ppm
0.944
0.891
0.964
0.973
1.998
3.000
0.907

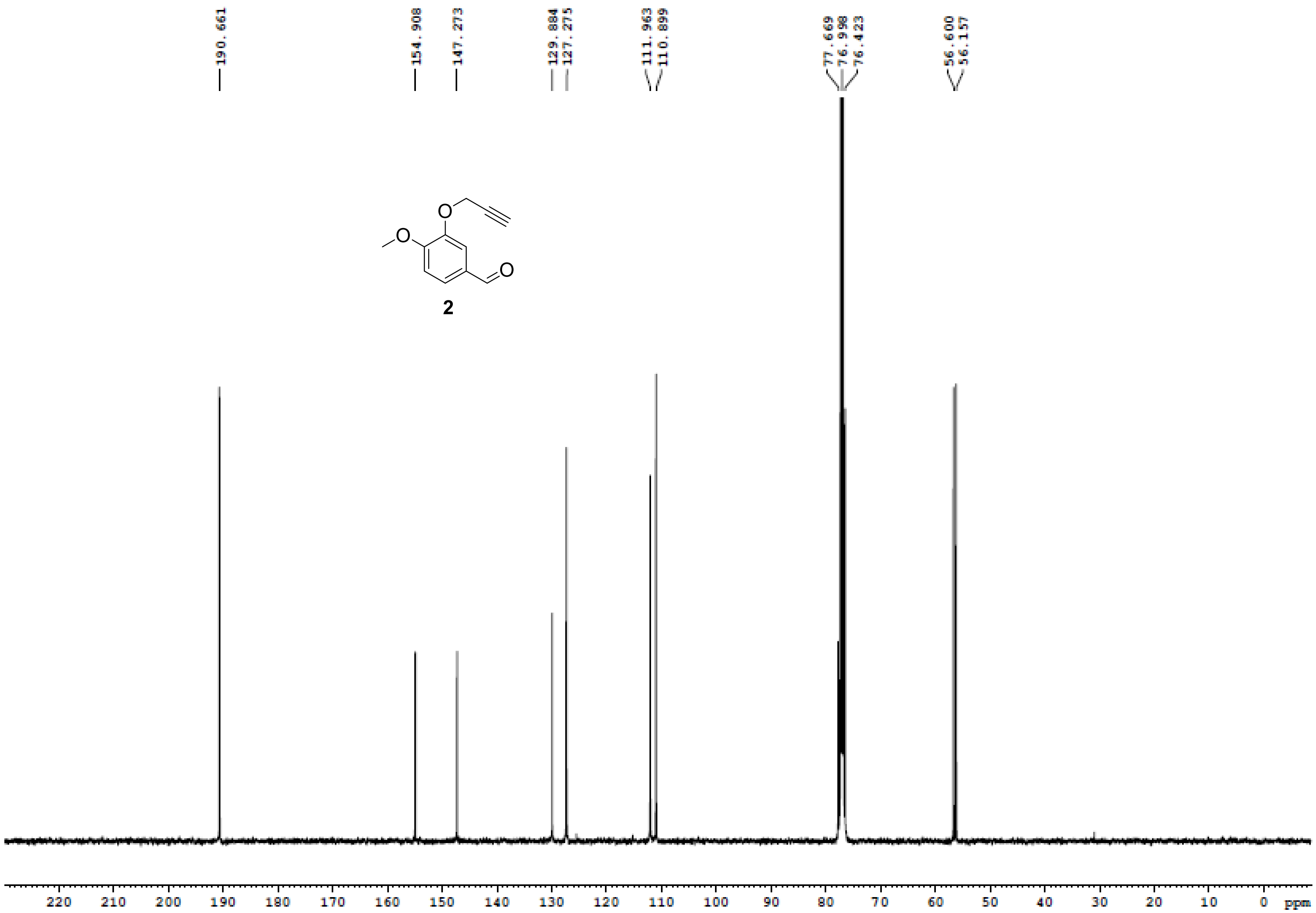
190.661
154.908
147.273
129.884
127.275
111.963
110.899
77.669
76.998
76.423
56.600
56.157
O
O
O
2
220 210 200 190 180 170 160 150 140 130 120 110 100 90 80 70 60 50 40 30 20 10 0 ppm

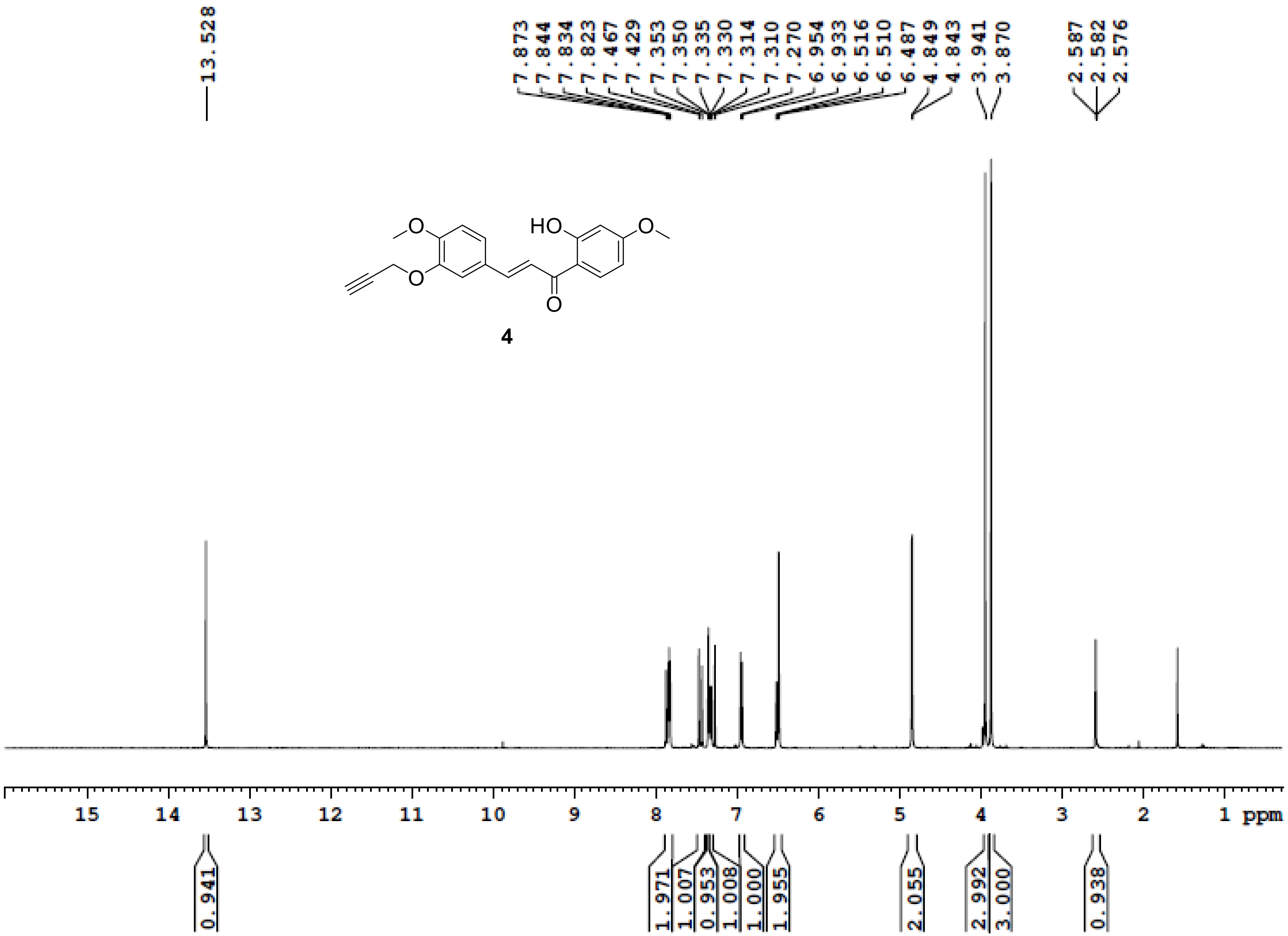
13.528
7.873 7.844 7.834 7.823 7.467 7.429 7.353 7.350 7.335 7.330 7.314 7.310 7.270 6.954 6.933 6.516 6.510 6.487 4.849 4.843 3.941 3.870
2.587 2.582 2.576
HO
O
4
15 14 13 12 11 10 9 8 7 6 5 4 3 2 1 ppm
0.941
1.971 1.007 0.953 1.008 1.000 1.955
2.055
2.992 3.000
0.938

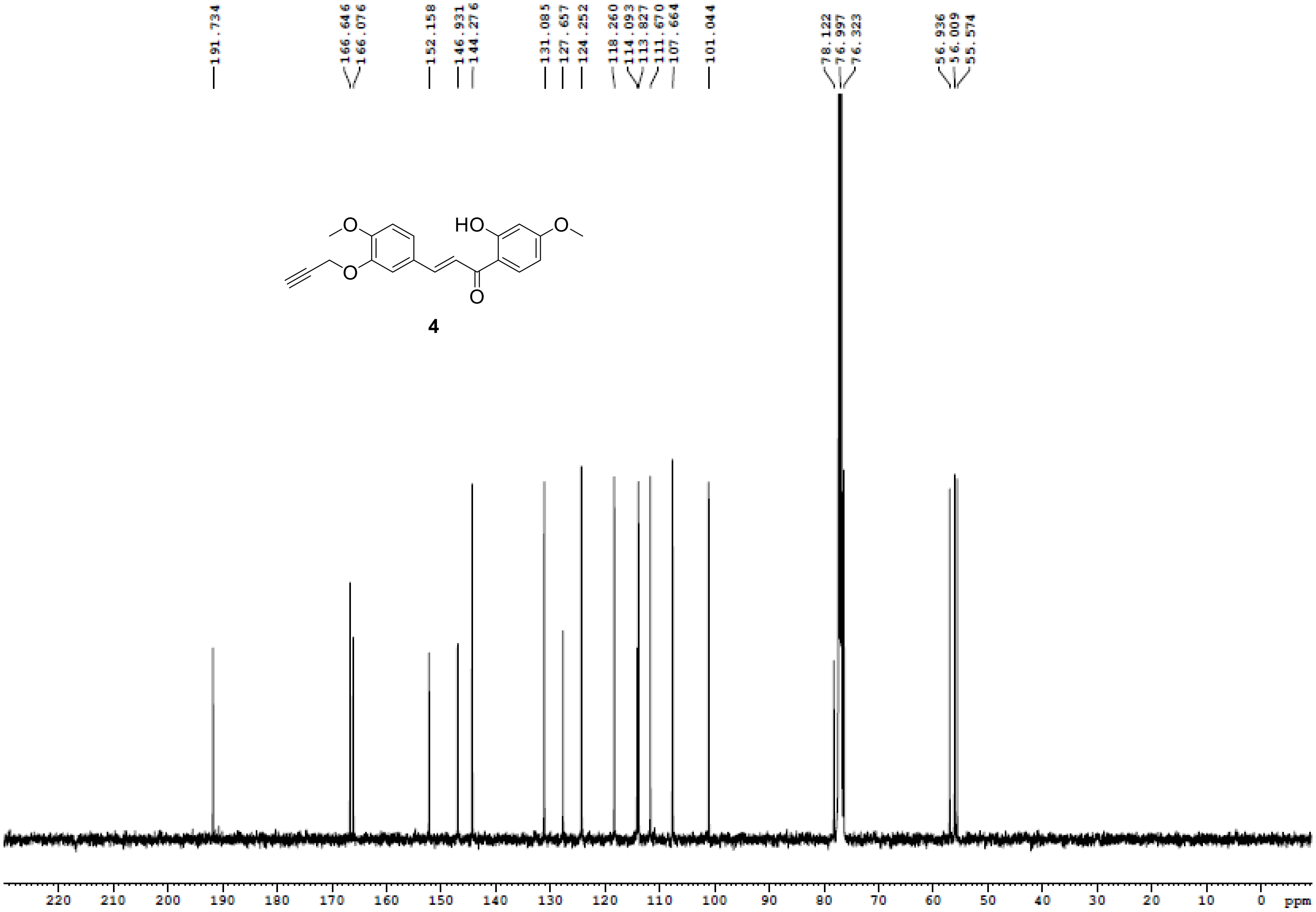
191.734
166.646
166.076
152.158
146.931
144.276
131.085
127.657
124.252
118.260
114.093
113.827
111.670
107.664
101.044
78.122
76.997
76.323
56.936
56.009
55.574
HO
O
O
O
O
4
220 210 200 190 180 170 160 150 140 130 120 110 100 90 80 70 60 50 40 30 20 10 0 ppm

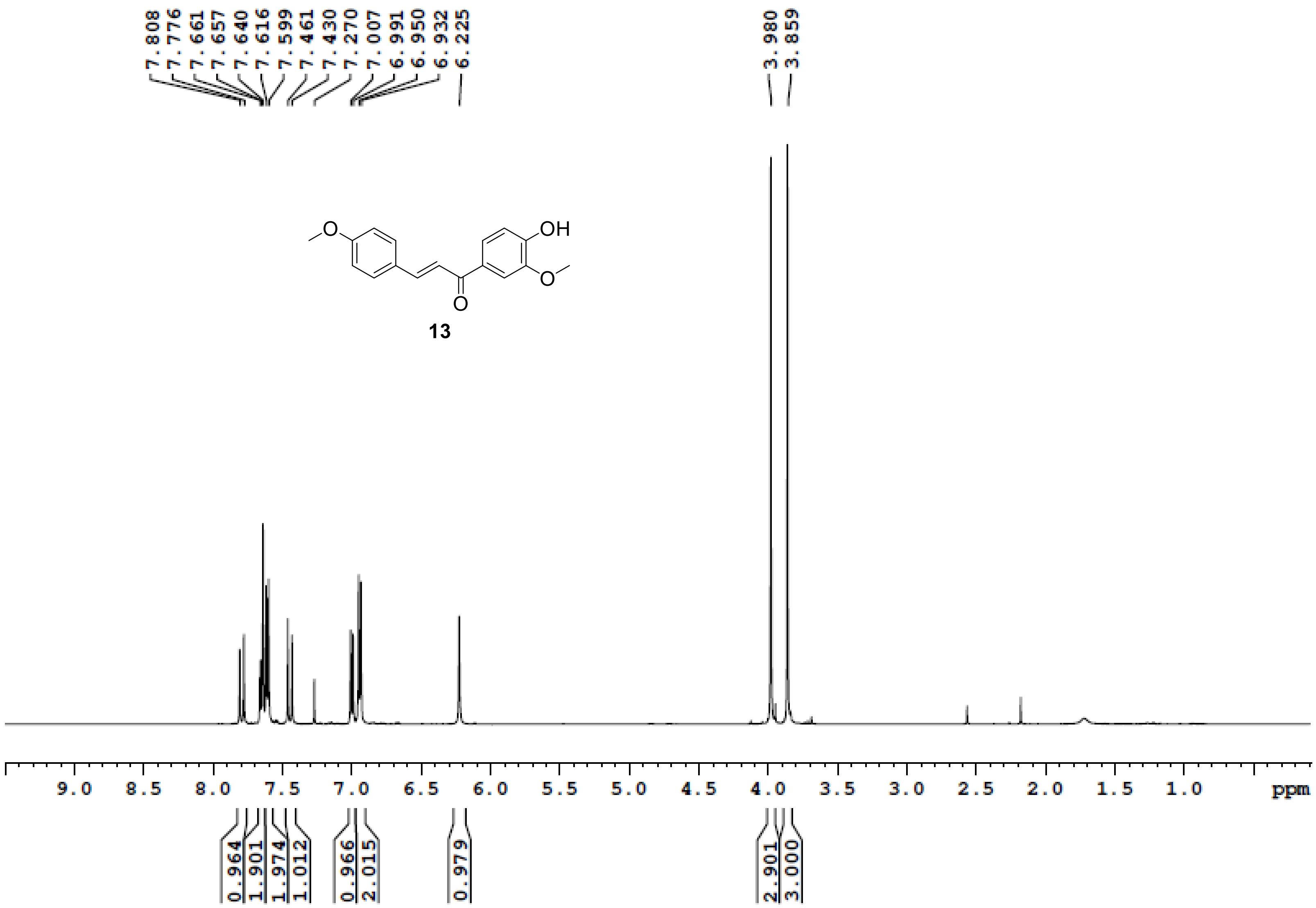
7.808 7.776 7.661 7.657 7.640 7.616 7.599 7.461 7.430 7.270 7.007 6.991 6.950 6.932 6.225
3.980 3.859
OH
O
O
O
13
9.0 8.5 8.0 7.5 7.0 6.5 6.0 5.5 5.0 4.5 4.0 3.5 3.0 2.5 2.0 1.5 1.0 ppm
0.964 1.901 1.974 1.012 0.966 2.015 0.979 2.901 3.000

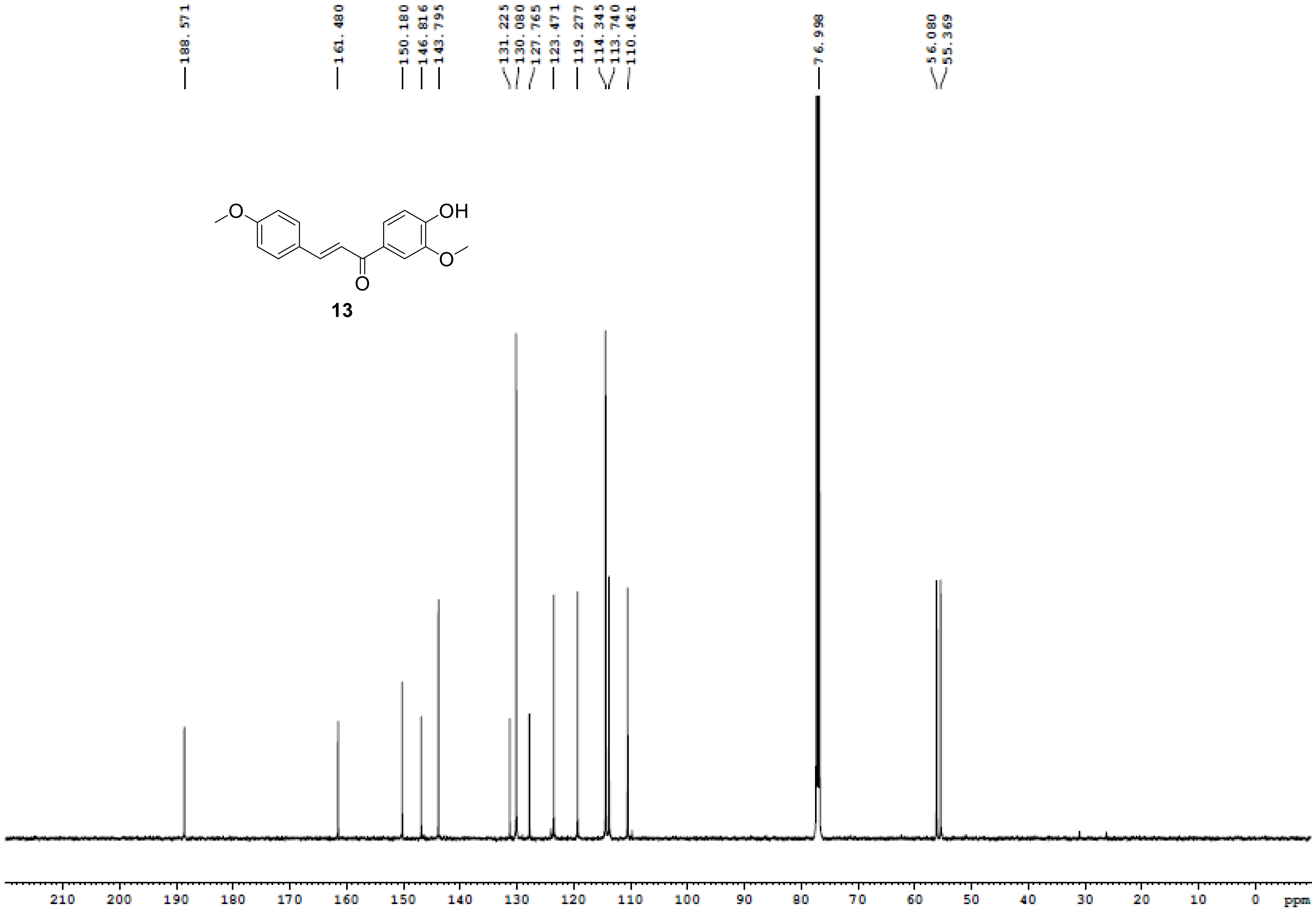
188.571
161.480
150.180
146.816
143.795
131.225
130.080
127.765
123.471
119.277
114.345
113.740
110.461
76.998
56.080
55.369
OH
O
O
O
13
210 200 190 180 170 160 150 140 130 120 110 100 90 80 70 60 50 40 30 20 10 0 ppm

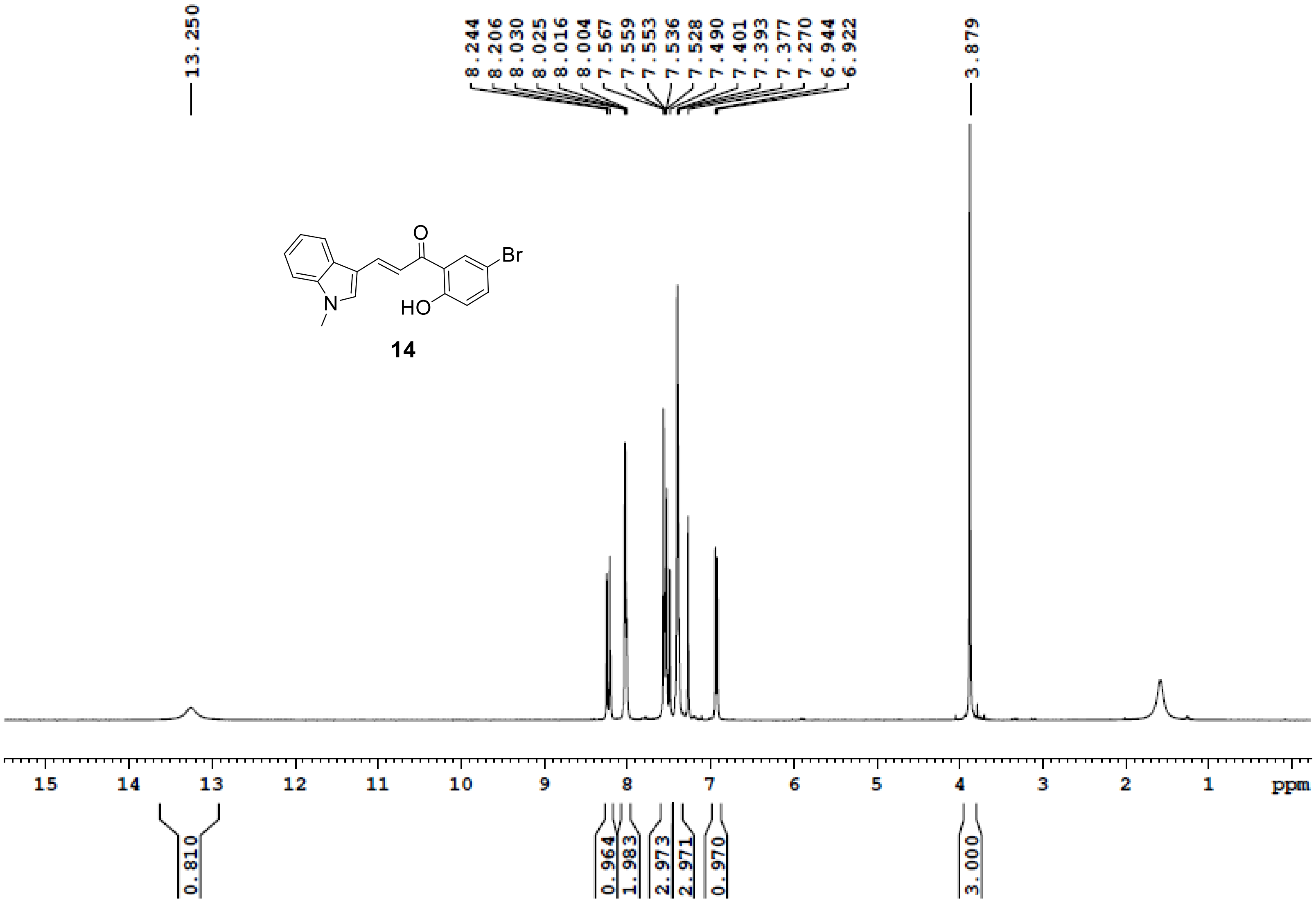
13.250
8.244
8.206
8.030
8.025
8.016
8.004
7.567
7.559
7.553
7.536
7.528
7.490
7.401
7.393
7.377
7.270
6.944
6.922
3.879
O
Br
N
HO
14
15
14
13
12
11
10
9
8
7
6
5
4
3
2
1
ppm
0.810
0.964
1.983
2.973
2.971
0.970
3.000

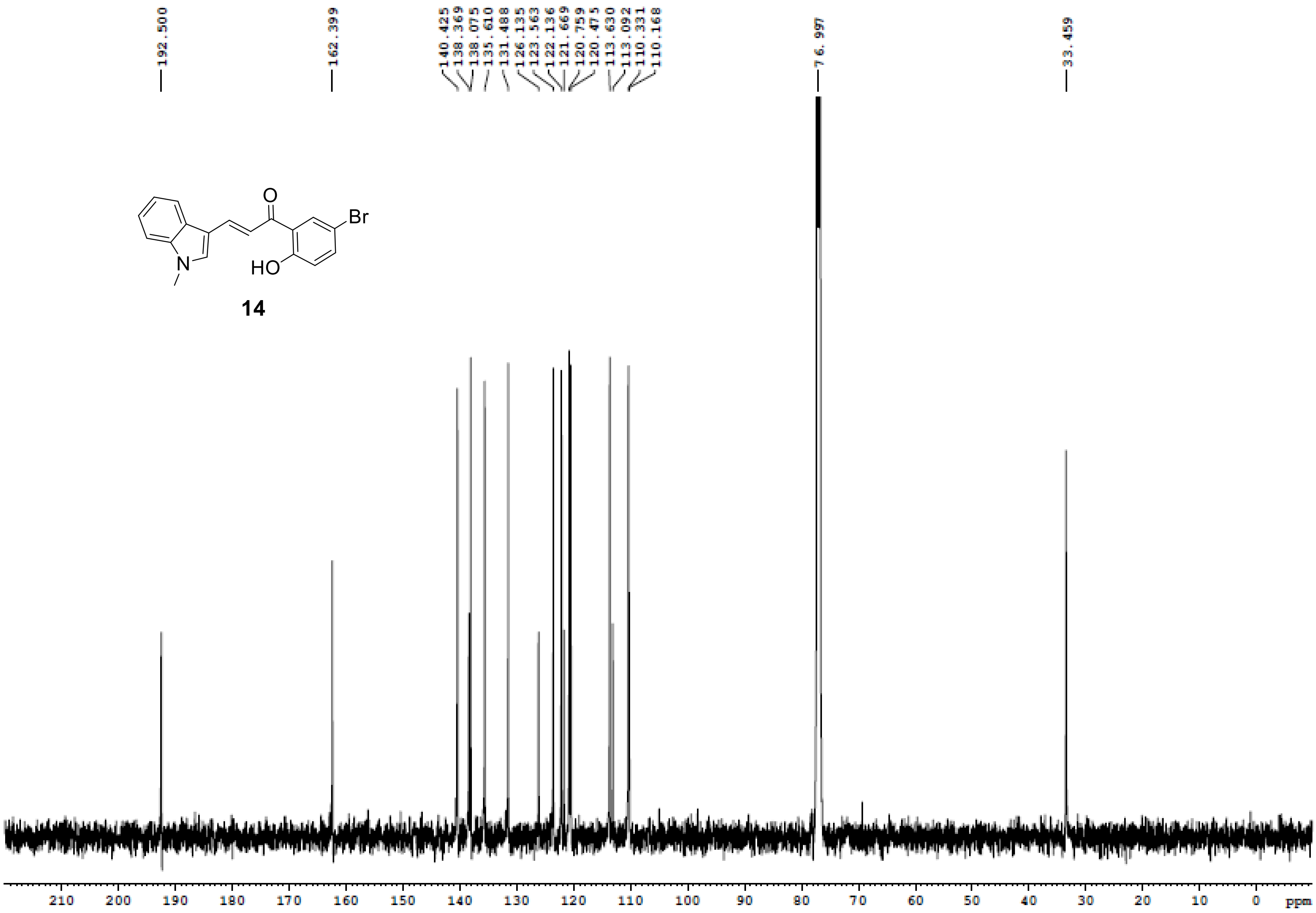
192.500
162.399
140.425
138.369
138.075
135.610
131.488
126.135
123.563
122.136
121.669
120.759
120.475
113.630
113.092
110.331
110.168
76.997
33.459
O
Br
N
HO
14
210 200 190 180 170 160 150 140 130 120 110 100 90 80 70 60 50 40 30 20 10 0 ppm

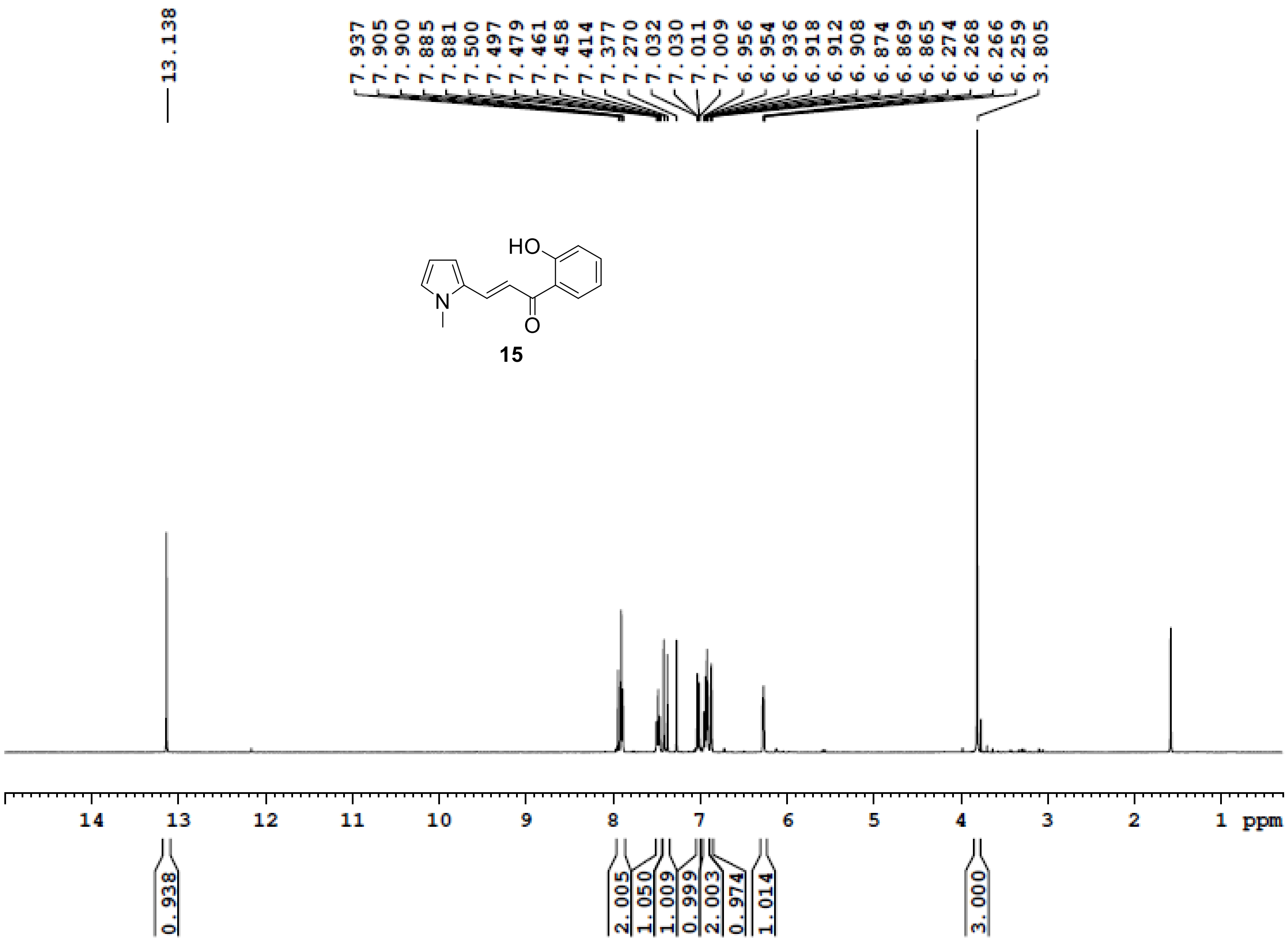
13.138
7.937
7.905
7.900
7.885
7.881
7.500
7.497
7.479
7.461
7.458
7.414
7.377
7.270
7.032
7.030
7.011
7.009
6.956
6.954
6.936
6.918
6.912
6.908
6.874
6.869
6.865
6.274
6.268
6.266
6.259
3.805
HO
N
O
15
14
13
12
11
10
9
8
7
6
5
4
3
2
1 ppm
0.938
2.005
1.050
1.009
0.999
2.003
0.974
1.014
3.000

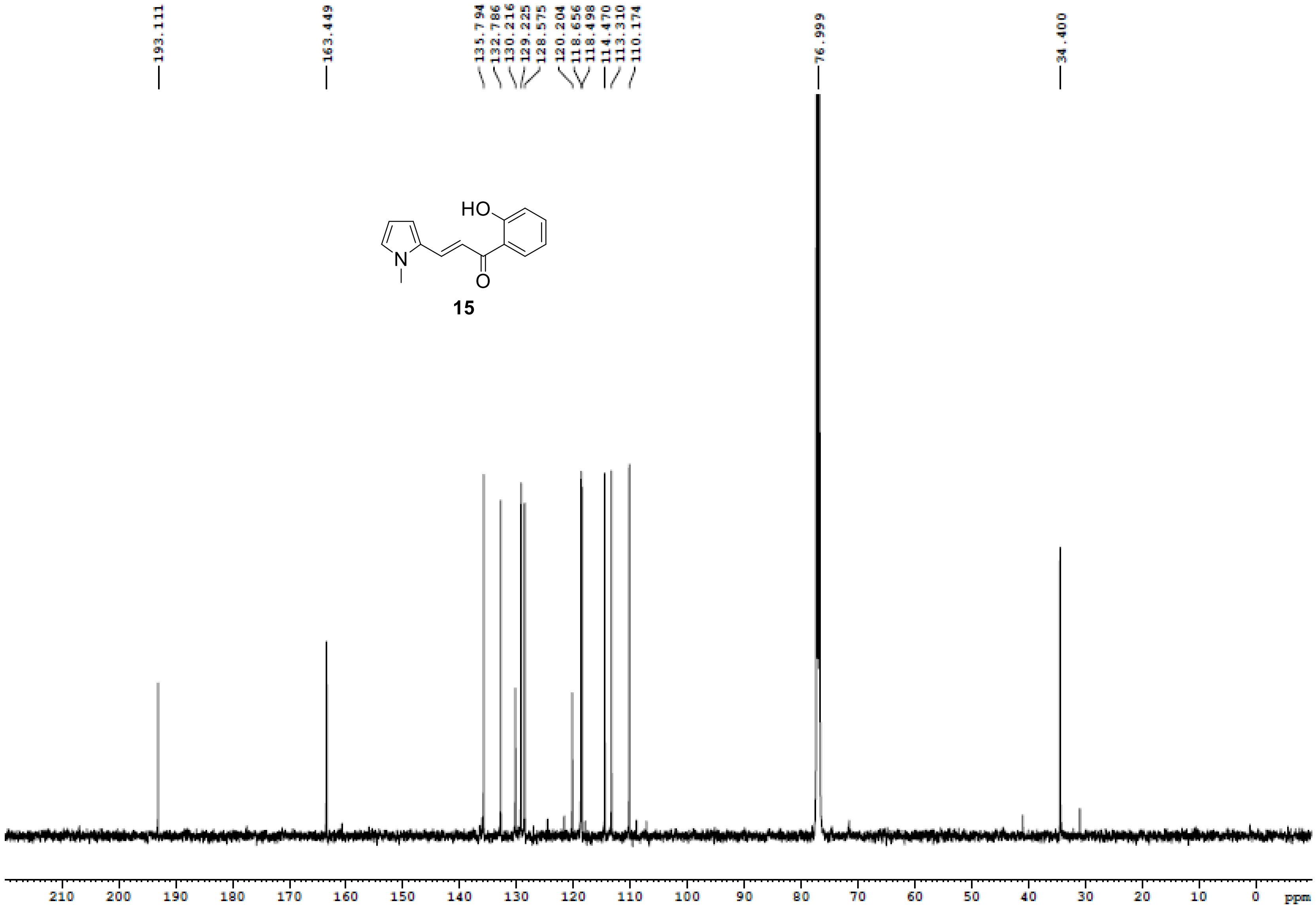
193.111
163.449
135.794
132.786
130.216
129.225
128.575
120.204
118.656
118.498
114.470
113.310
110.174
76.999
34.400
HO
N
O
15
210 200 190 180 170 160 150 140 130 120 110 100 90 80 70 60 50 40 30 20 10 0 ppm

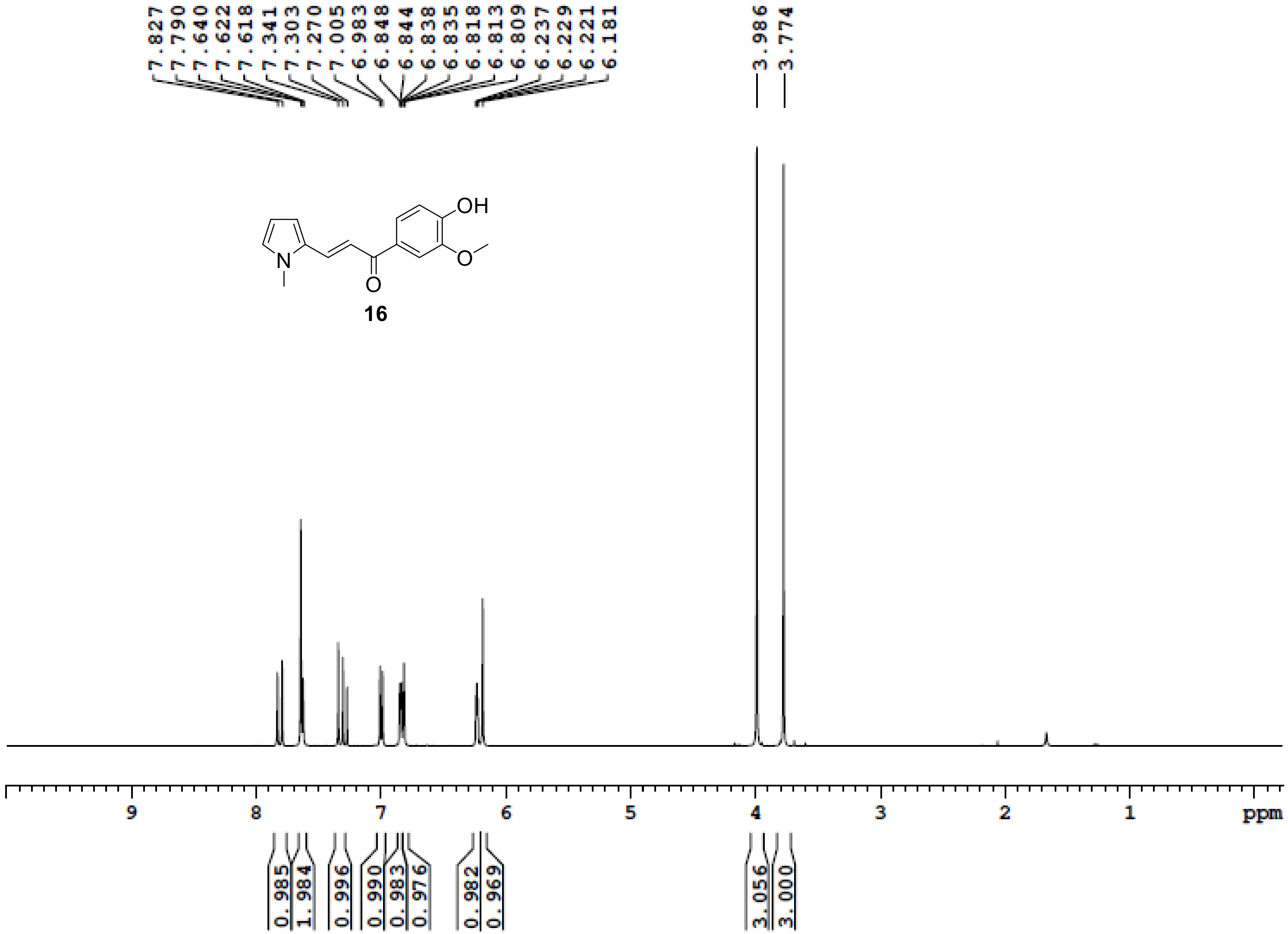
7.827
7.790
7.640
7.622
7.618
7.341
7.303
7.270
7.005
6.983
6.848
6.844
6.838
6.835
6.818
6.813
6.809
6.237
6.229
6.221
6.181
3.986
3.774
OH
N
O
O
16
9
8
7
6
5
4
3
2
1
ppm
0.985
1.984
0.996
0.990
0.983
0.976
0.982
0.969
3.056
3.000

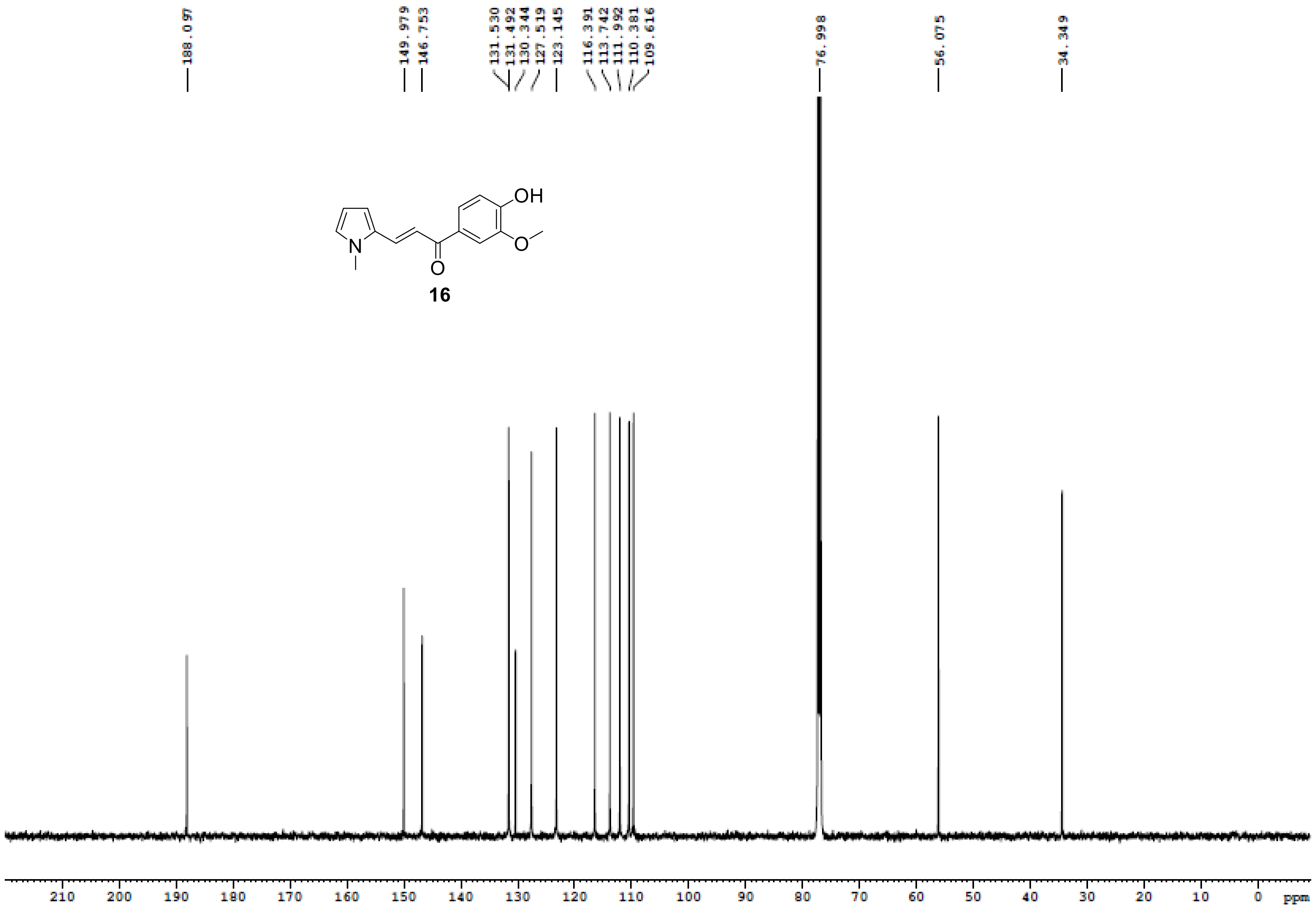
188.097
149.979
146.753
131.530
131.492
130.344
127.519
123.145
116.391
113.742
111.992
110.381
109.616
76.998
56.075
34.349
OH
N
O
O
16
210 200 190 180 170 160 150 140 130 120 110 100 90 80 70 60 50 40 30 20 10 0 ppm

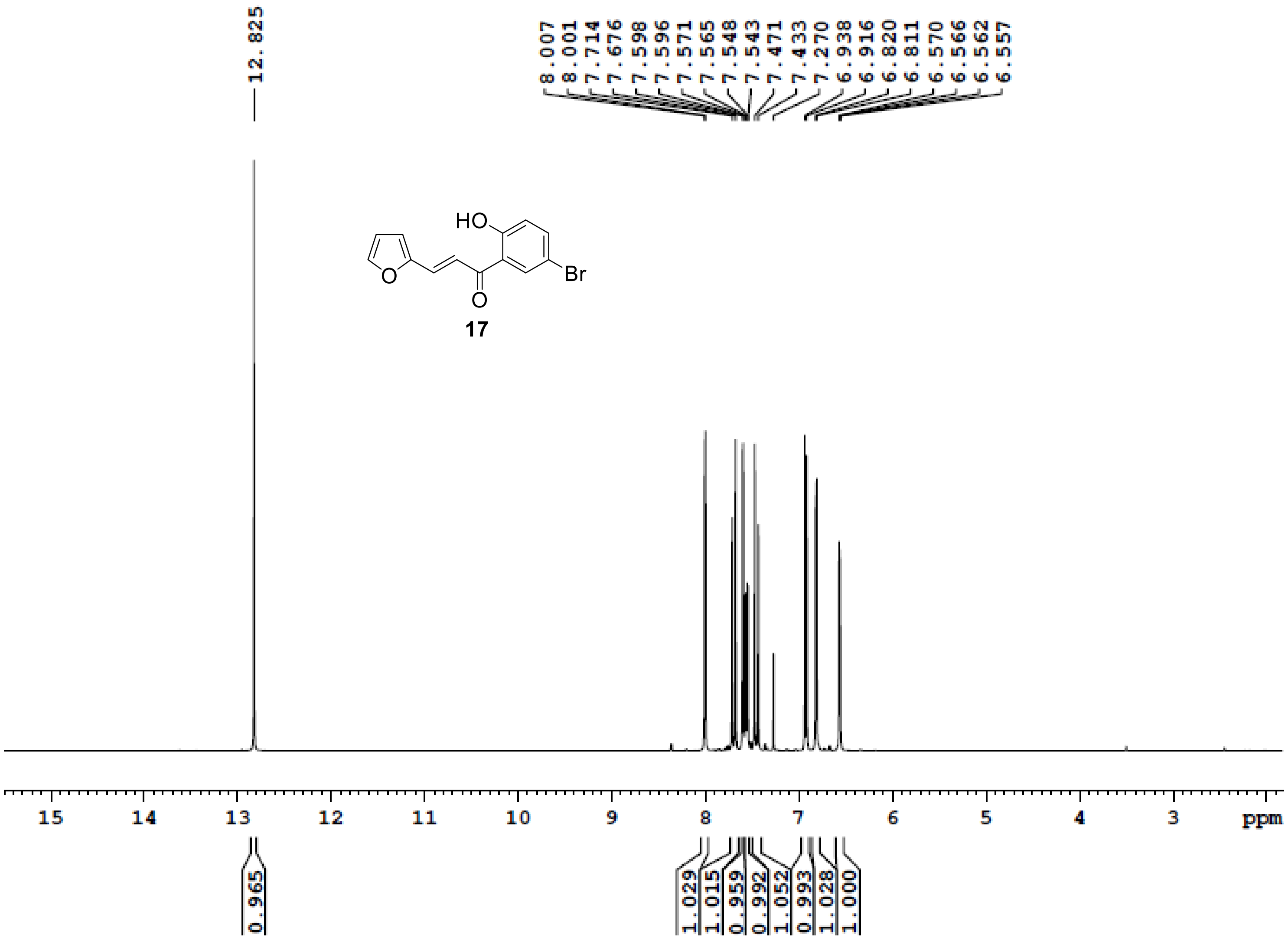
12.825
8.007
8.001
7.714
7.676
7.598
7.596
7.571
7.565
7.548
7.543
7.471
7.433
7.270
6.938
6.916
6.820
6.811
6.570
6.566
6.562
6.557
HO
O
Br
O
17
15
14
13
12
11
10
9
8
7
6
5
4
3
ppm
0.965
1.029
1.015
0.959
0.992
1.052
0.993
1.028
1.000

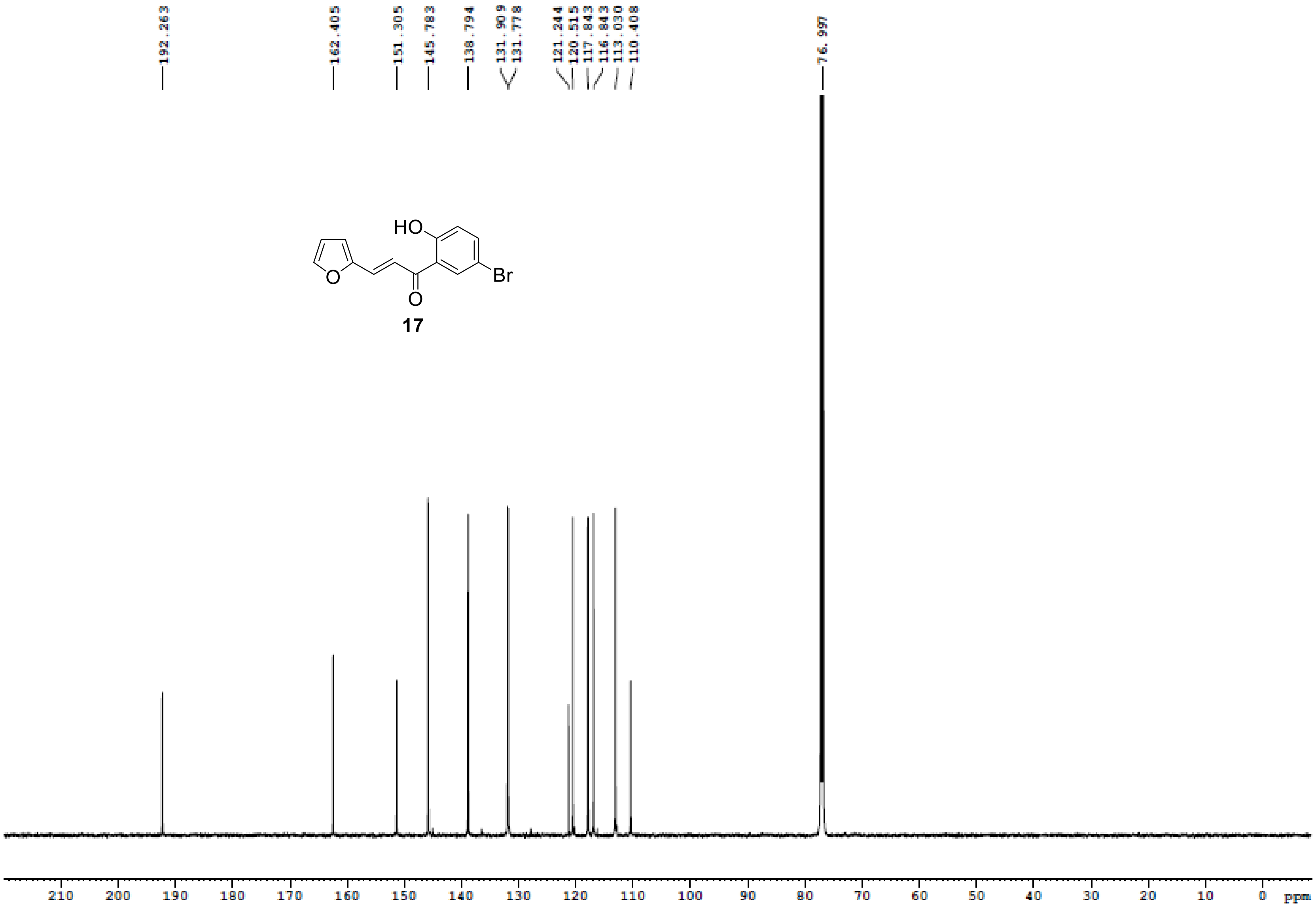
192.263
162.405
151.305
145.783
138.794
131.909
131.778
121.244
120.515
117.843
116.843
113.030
110.408
76.997
HO
O
Br
O
17
210 200 190 180 170 160 150 140 130 120 110 100 90 80 70 60 50 40 30 20 10 0 ppm

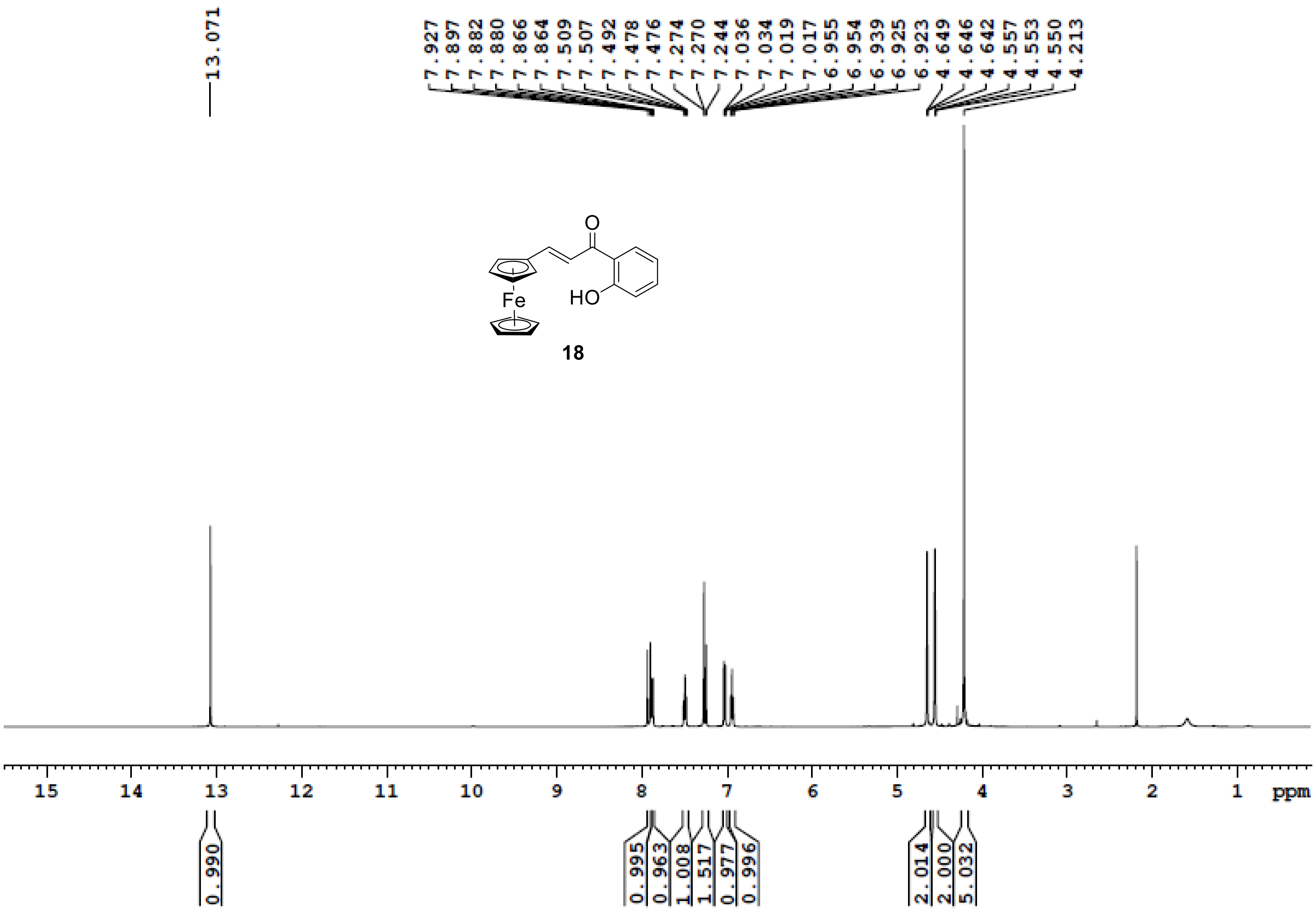
13.071
7.927
7.897
7.882
7.880
7.866
7.864
7.509
7.507
7.492
7.478
7.476
7.274
7.270
7.244
7.036
7.034
7.019
7.017
6.955
6.954
6.939
6.925
6.923
4.649
4.646
4.642
4.557
4.553
4.550
4.213
O
Fe
HO
18
15
14
13
12
11
10
9
8
7
6
5
4
3
2
1
ppm
0.990
0.995
0.963
1.008
1.517
0.977
0.996
2.014
2.000
5.032

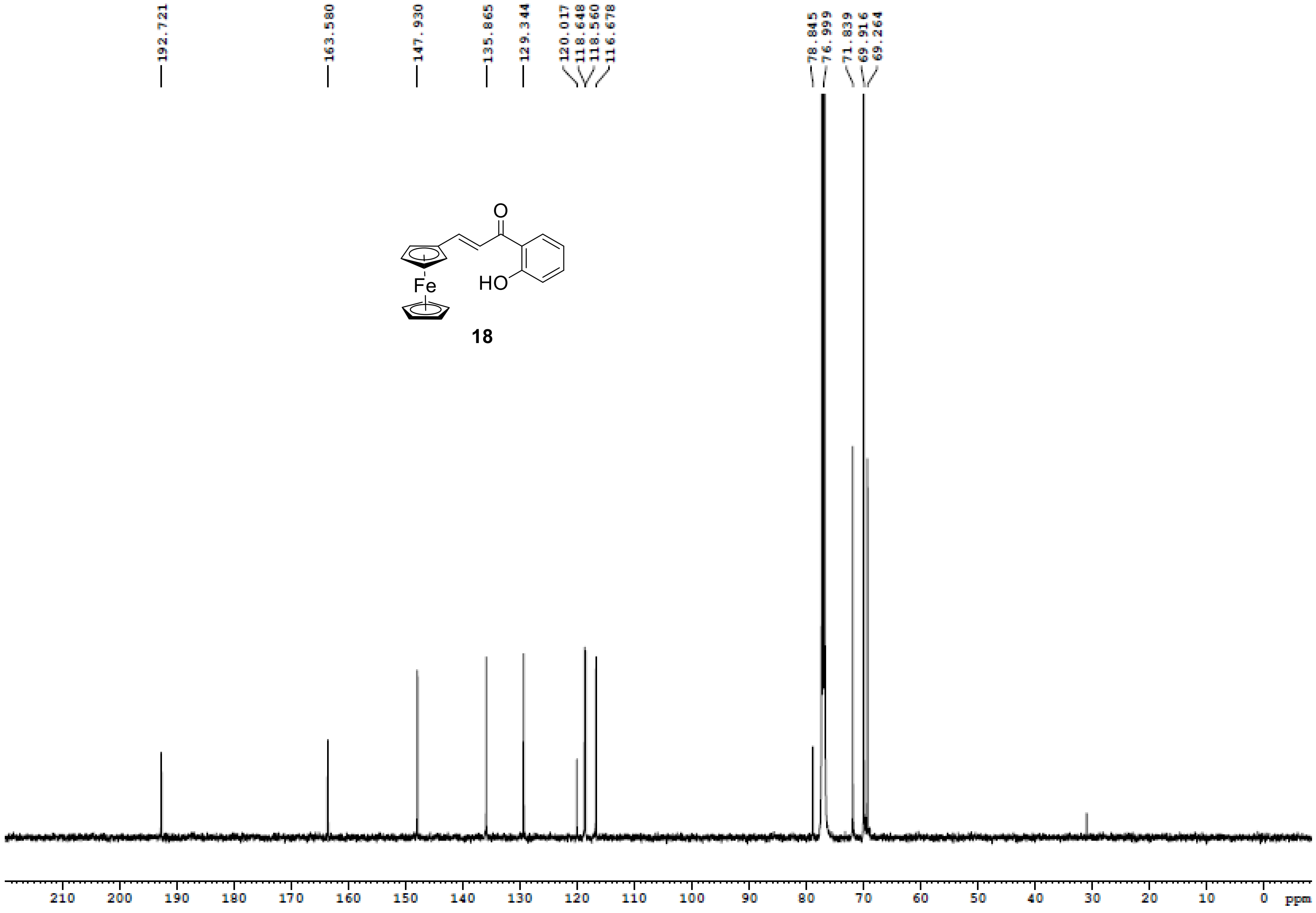
192.721
163.580
147.930
135.865
129.344
120.017
118.648
118.560
116.678
78.845
76.999
71.839
69.916
69.264
O
Fe
HO
18
210 200 190 180 170 160 150 140 130 120 110 100 90 80 70 60 50 40 30 20 10 0 ppm

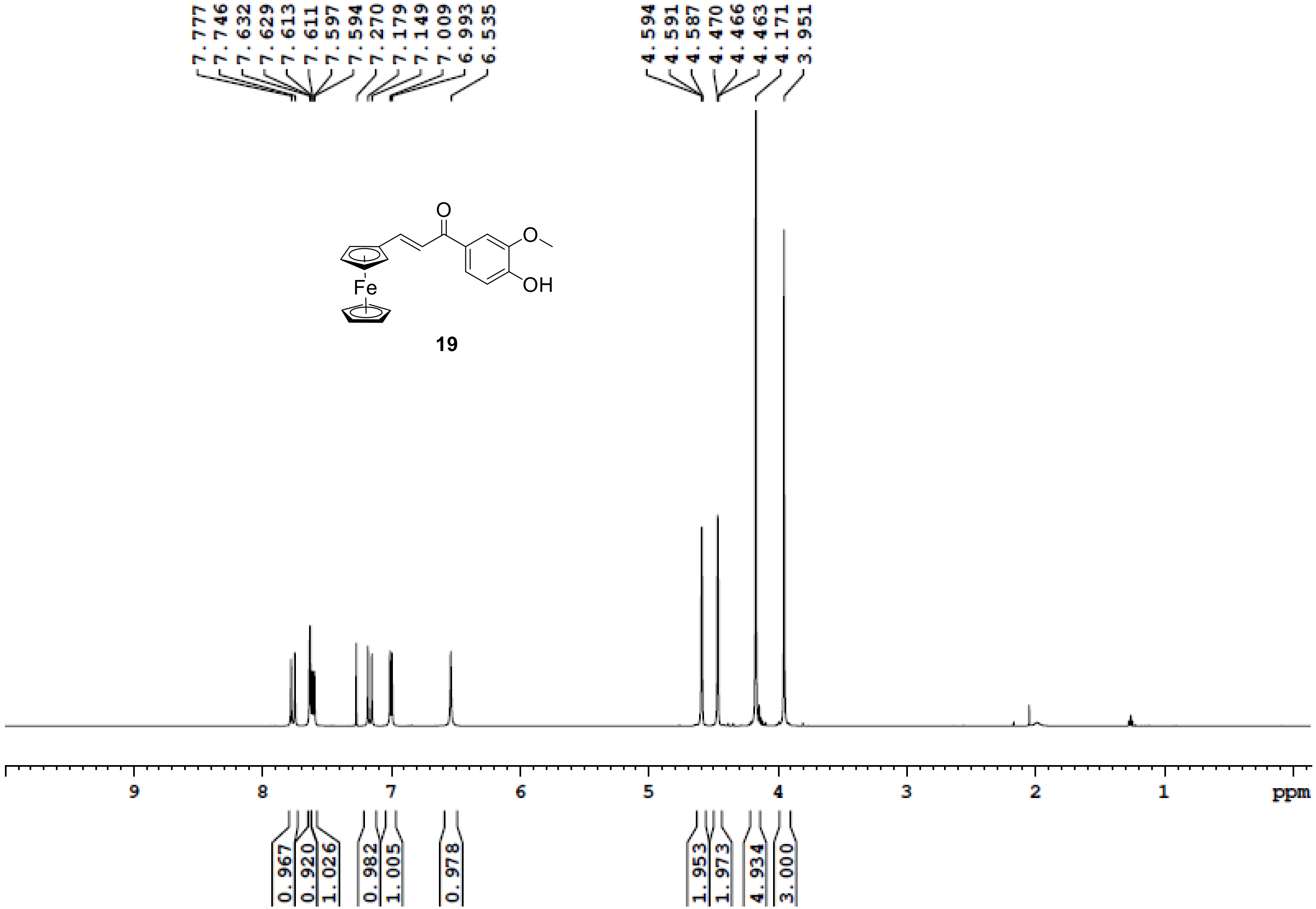
7.777
7.746
7.632
7.629
7.613
7.611
7.597
7.594
7.270
7.179
7.149
7.009
6.993
6.535
4.594
4.591
4.587
4.470
4.466
4.463
4.171
3.951
O
OH
Fe
19
9
8
7
6
5
4
3
2
1
ppm
0.967
0.920
1.026
0.982
1.005
0.978
1.953
1.973
4.934
3.000

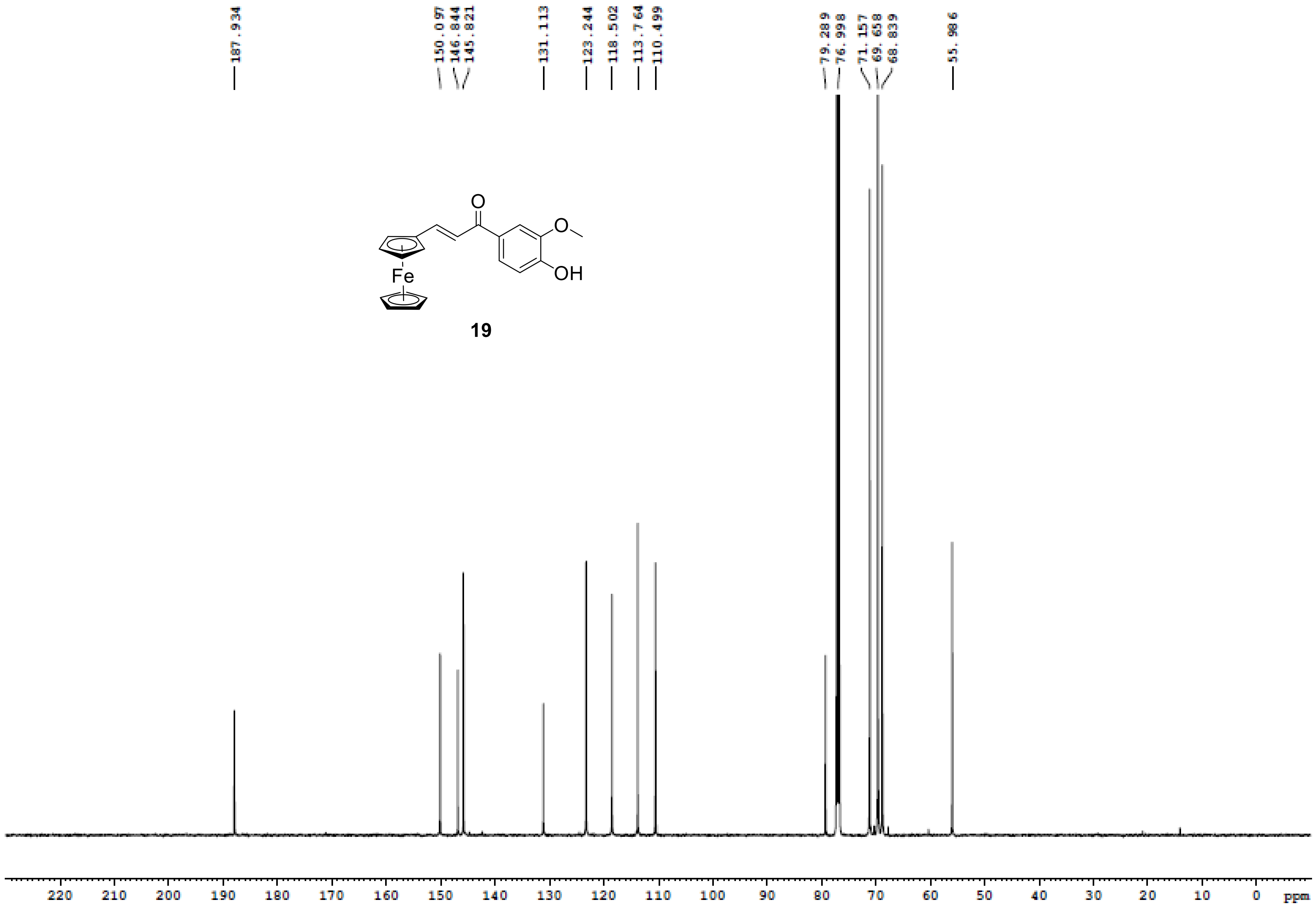
187.934
150.097
146.844
145.821
131.113
123.244
118.502
113.764
110.499
79.289
76.998
71.157
69.658
68.839
55.986
O
O
Fe
OH
19
220
210
200
190
180
170
160
150
140
130
120
110
100
90
80
70
60
50
40
30
20
10
0
ppm

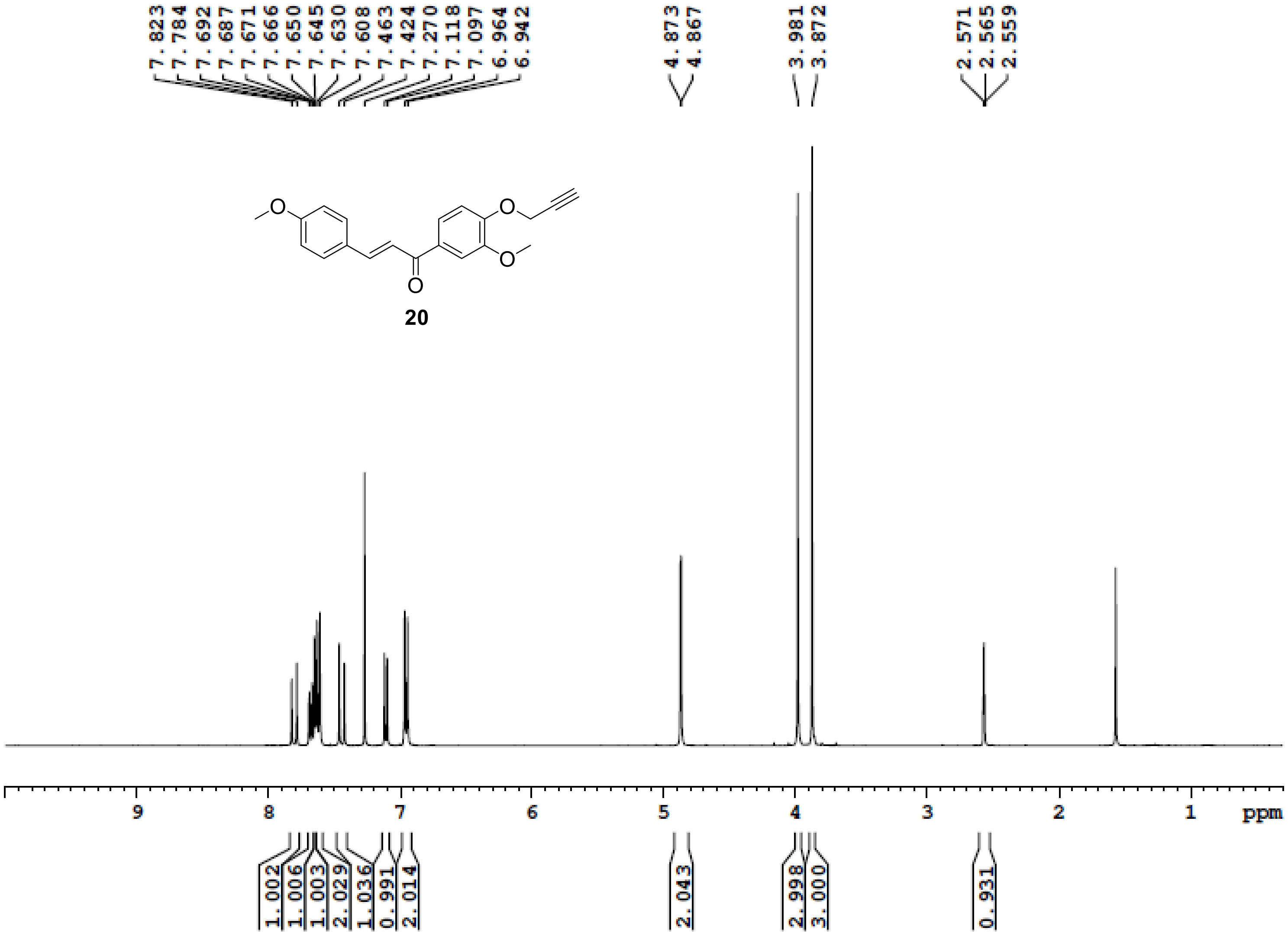
7.823
7.784
7.692
7.687
7.671
7.666
7.650
7.645
7.630
7.608
7.463
7.424
7.270
7.118
7.097
6.964
6.942
4.873
4.867
3.981
3.872
2.571
2.565
2.559
20
9
8
7
6
5
4
3
2
1
ppm
1.002
1.006
1.003
2.029
1.036
0.991
2.014
2.043
2.998
3.000
0.931

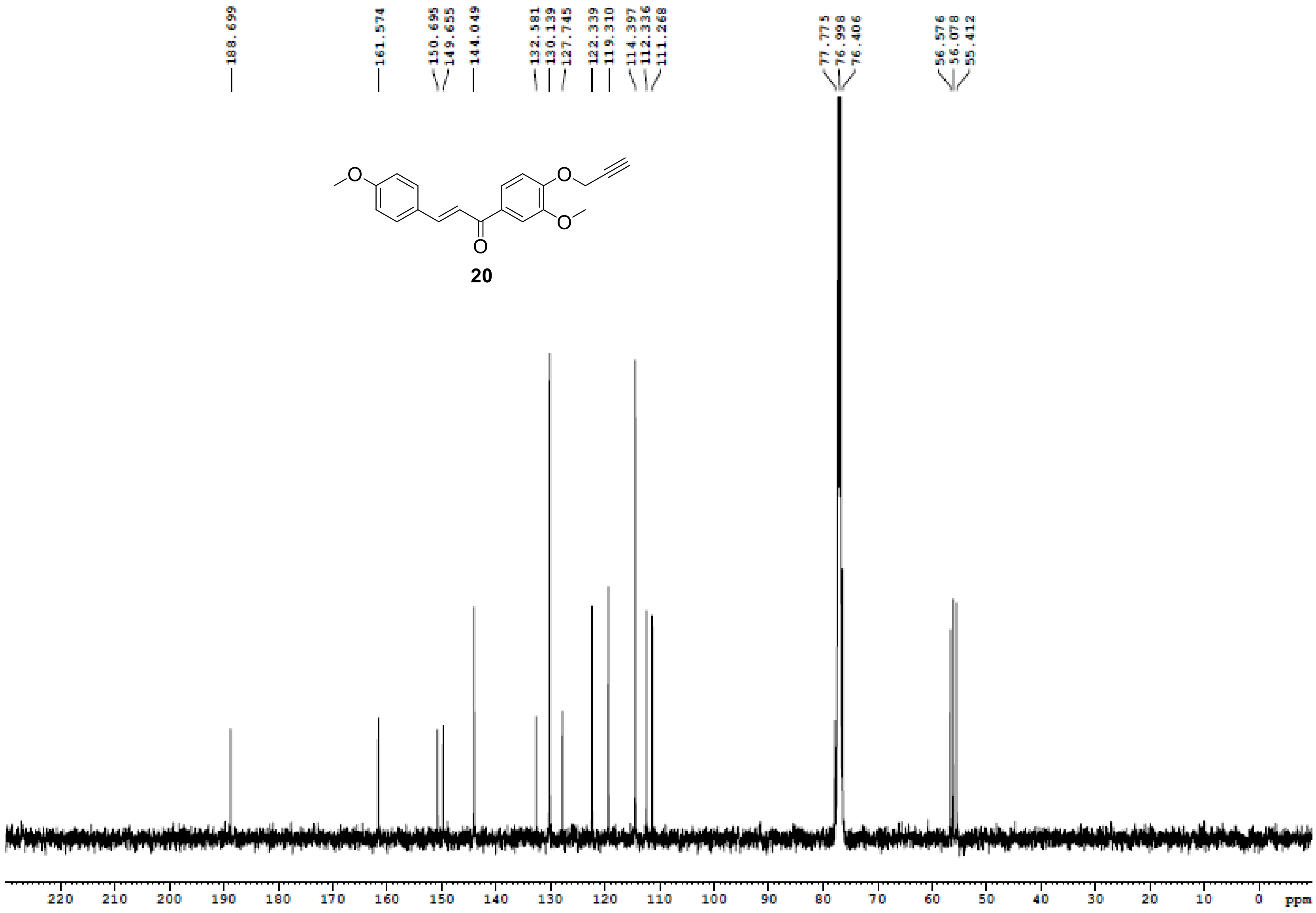
188.699
161.574
150.695
149.655
144.049
132.581
130.139
127.745
122.339
119.310
114.397
112.336
111.268
77.775
76.998
76.406
56.576
56.078
55.412
20
220 210 200 190 180 170 160 150 140 130 120 110 100 90 80 70 60 50 40 30 20 10 0 ppm

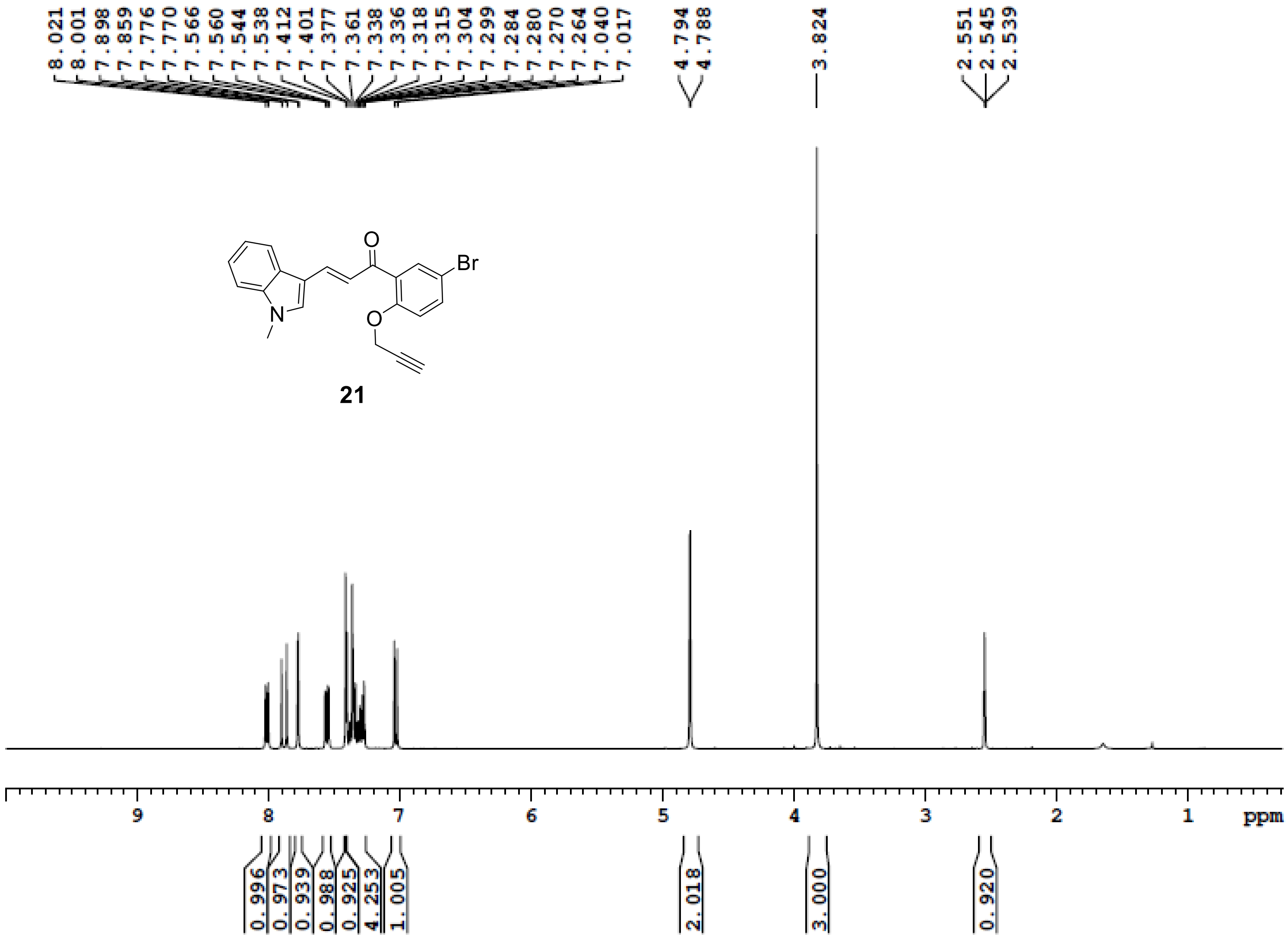
8.021
8.001
7.898
7.859
7.776
7.770
7.566
7.560
7.544
7.538
7.412
7.401
7.377
7.361
7.338
7.336
7.318
7.315
7.304
7.299
7.284
7.280
7.270
7.264
7.040
7.017
4.794
4.788
3.824
2.551
2.545
2.539
O
Br
N
O
9
8
7
6
5
4
3
2
1
ppm
0.996
0.973
0.939
0.988
0.925
4.253
1.005
2.018
3.000
0.920

21

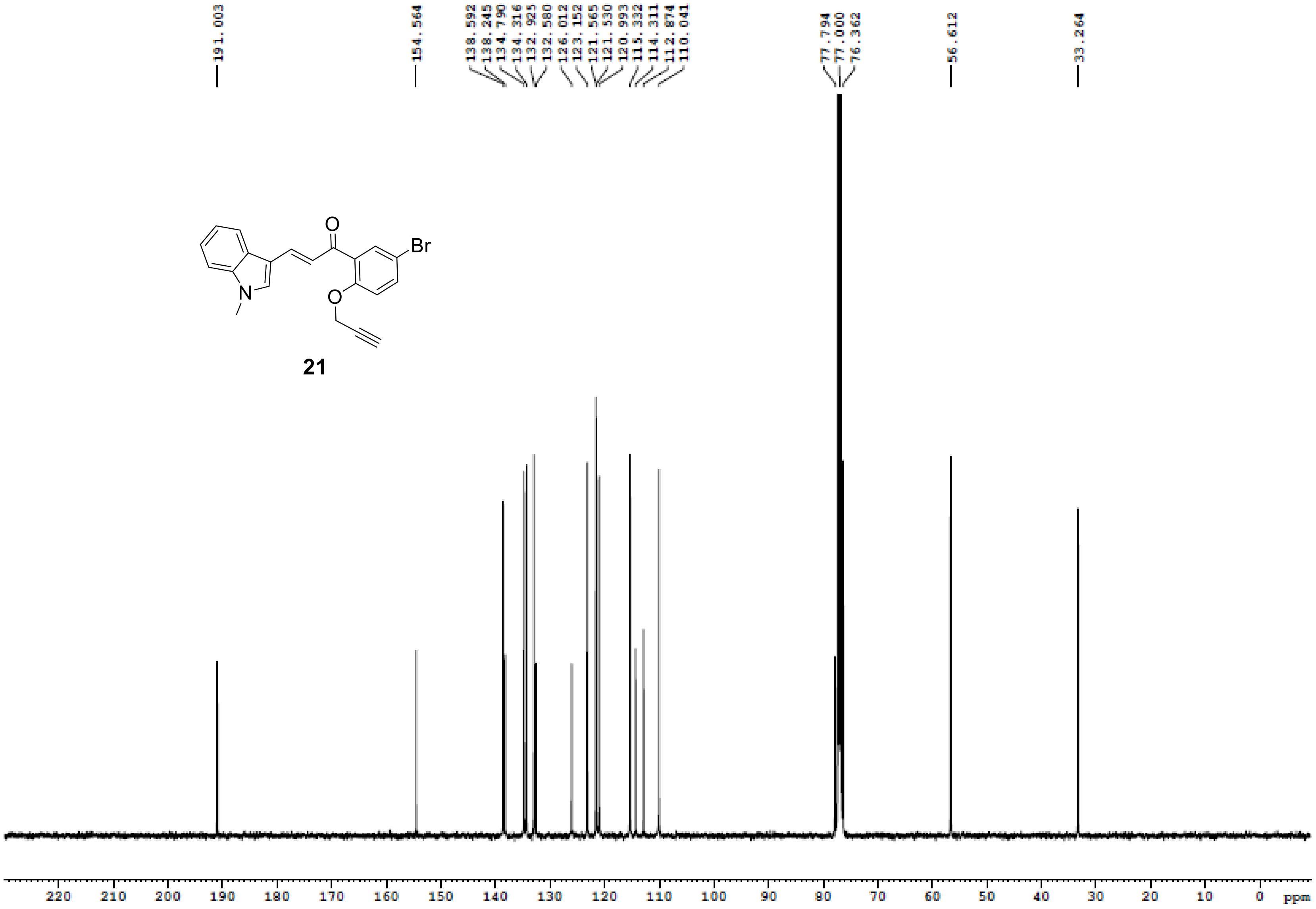
191.003
154.564
138.592
138.245
134.790
134.316
132.925
132.580
126.012
123.152
121.565
121.530
120.993
115.332
114.311
112.874
110.041
77.794
77.000
76.362
56.612
33.264
O
Br
N
O
21
220 210 200 190 180 170 160 150 140 130 120 110 100 90 80 70 60 50 40 30 20 10 0 ppm

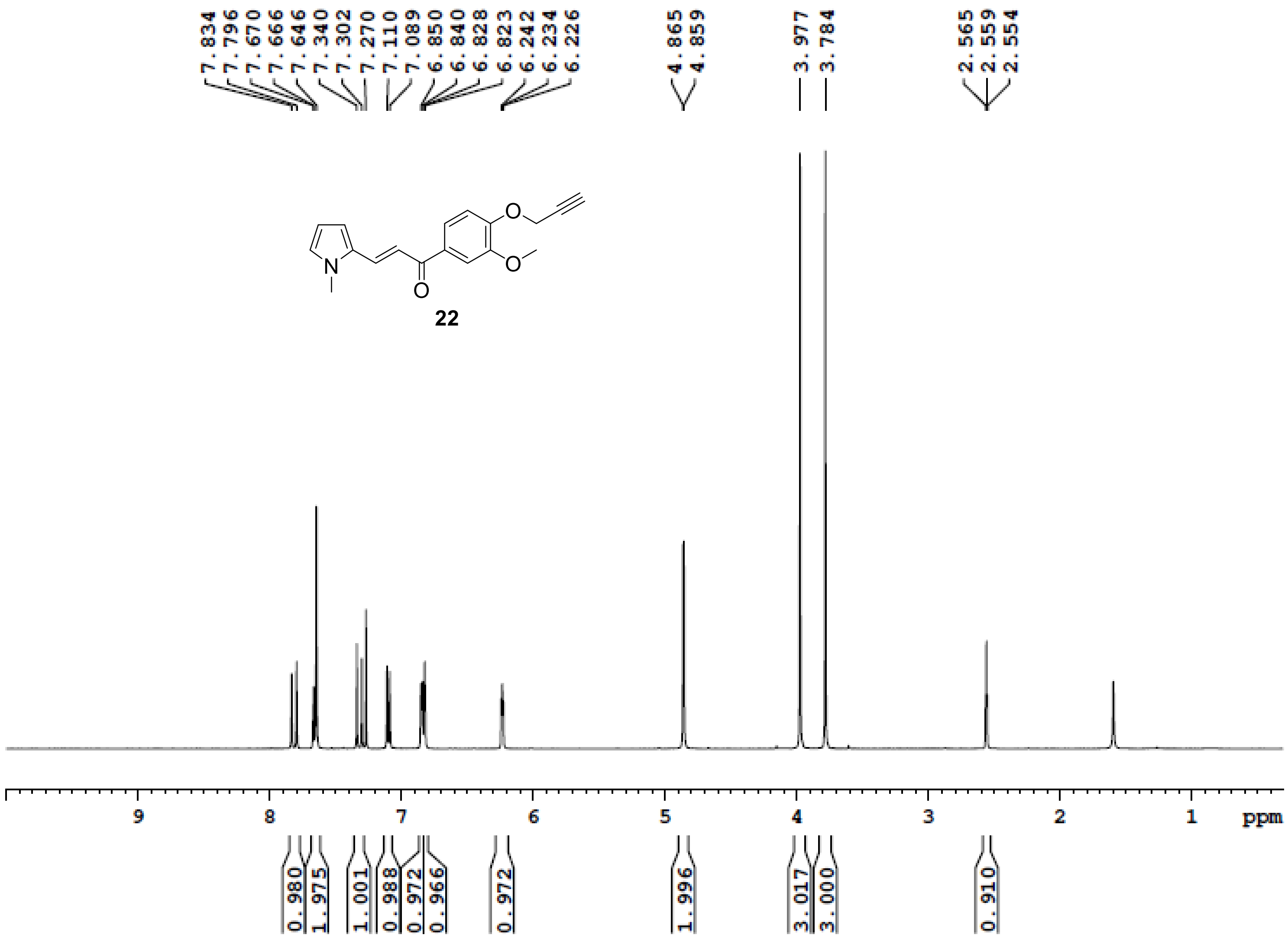
7.834
7.796
7.670
7.666
7.646
7.340
7.302
7.270
7.110
7.089
6.850
6.840
6.828
6.823
6.242
6.234
6.226
4.865
4.859
3.977
3.784
2.565
2.559
2.554
N
O
O
O
22
9
8
7
6
5
4
3
2
1
ppm
0.980
1.975
1.001
0.988
0.972
0.966
0.972
1.996
3.017
3.000
0.910

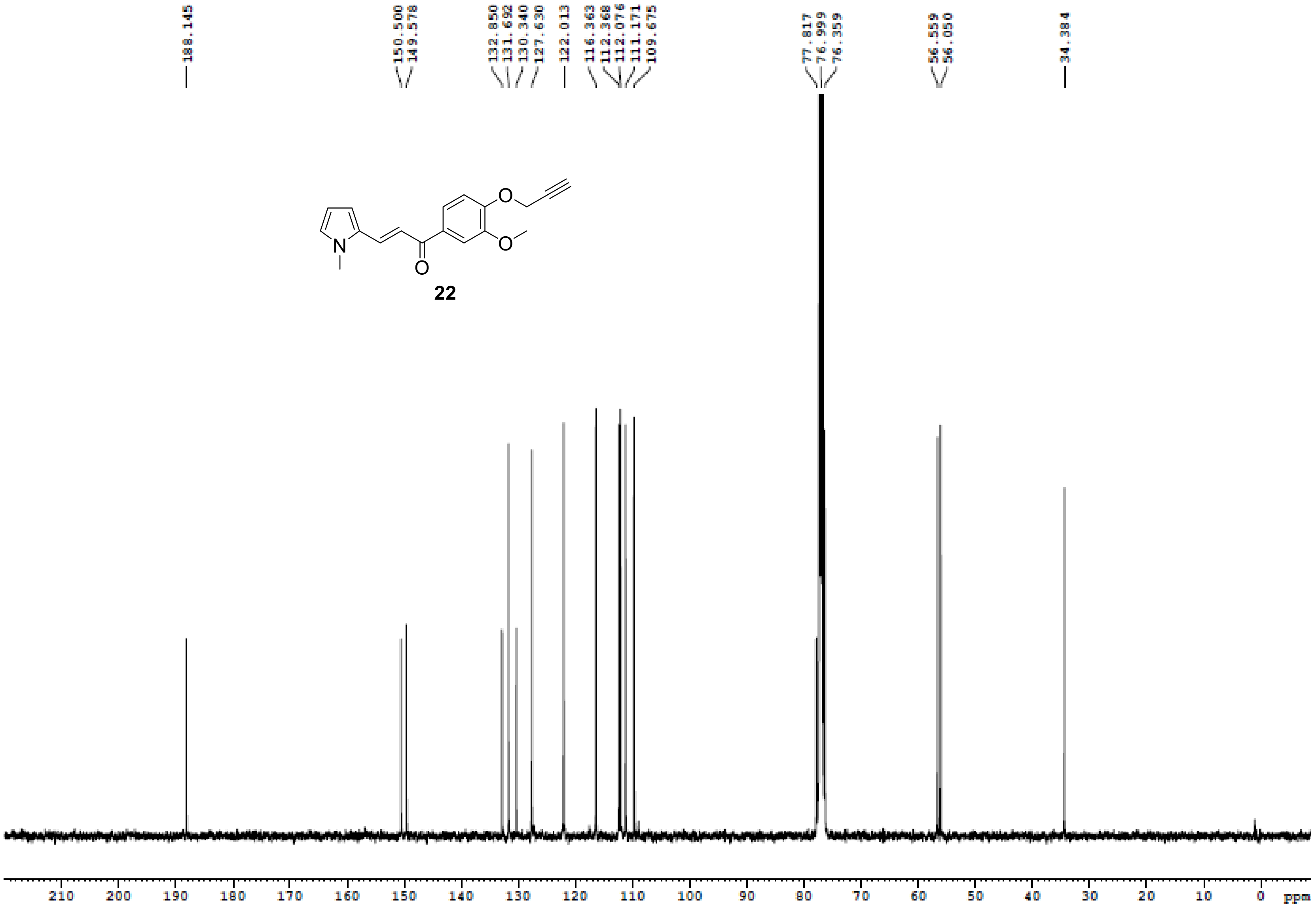
188.145
150.500
149.578
132.850
131.692
130.340
127.630
122.013
116.363
112.368
112.076
111.171
109.675
77.817
76.999
76.359
56.559
56.050
34.384
N
O
O
O
22
210 200 190 180 170 160 150 140 130 120 110 100 90 80 70 60 50 40 30 20 10 0 ppm

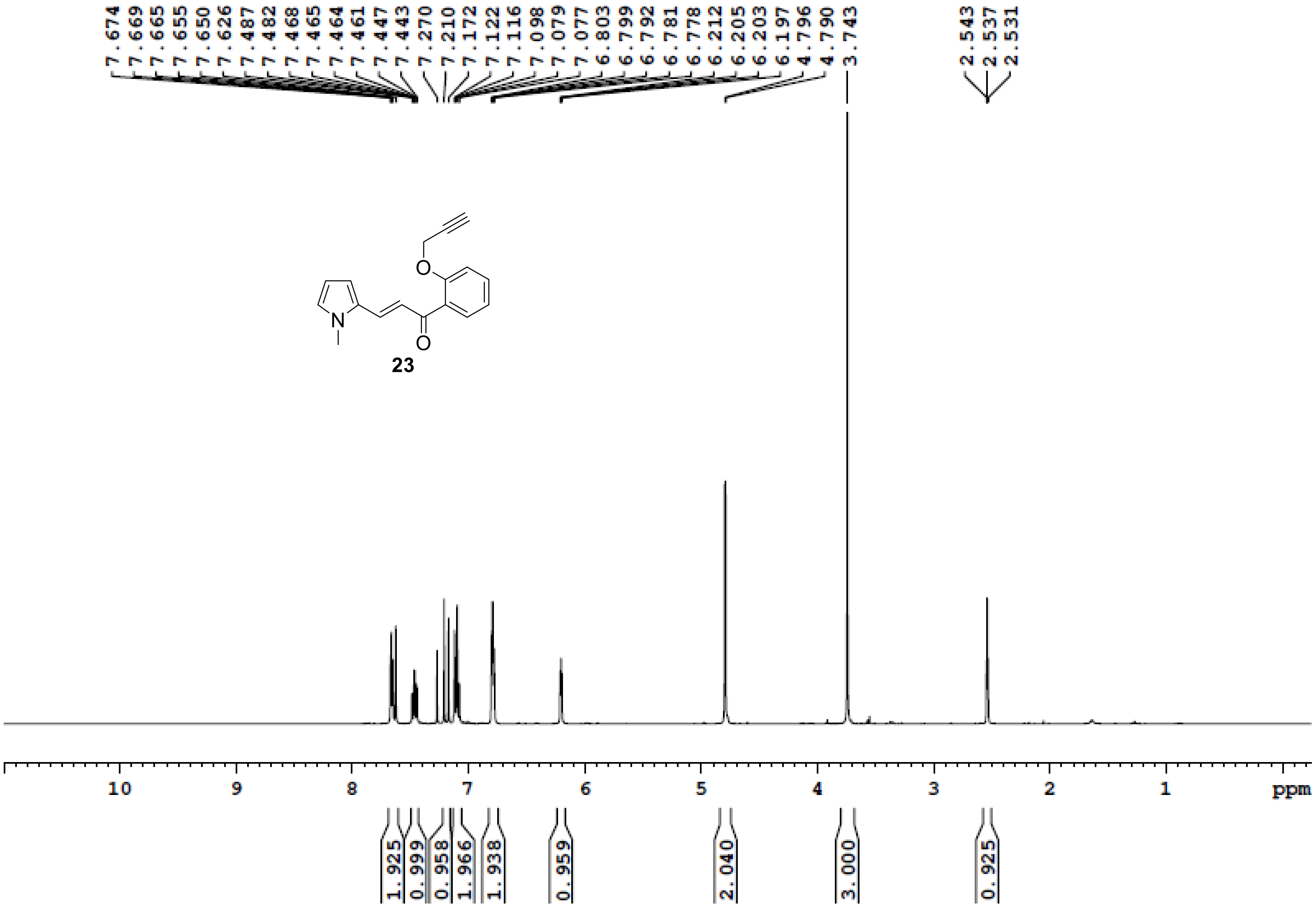
7.674
7.669
7.665
7.655
7.650
7.626
7.487
7.482
7.468
7.465
7.464
7.461
7.447
7.443
7.270
7.210
7.172
7.122
7.116
7.098
7.079
7.077
6.803
6.799
6.792
6.781
6.778
6.212
6.205
6.203
6.197
4.796
4.790
3.743
2.543
2.537
2.531
23
10
9
8
7
6
5
4
3
2
1
ppm
1.925
0.999
0.958
1.966
1.938
0.959
2.040
3.000
0.925

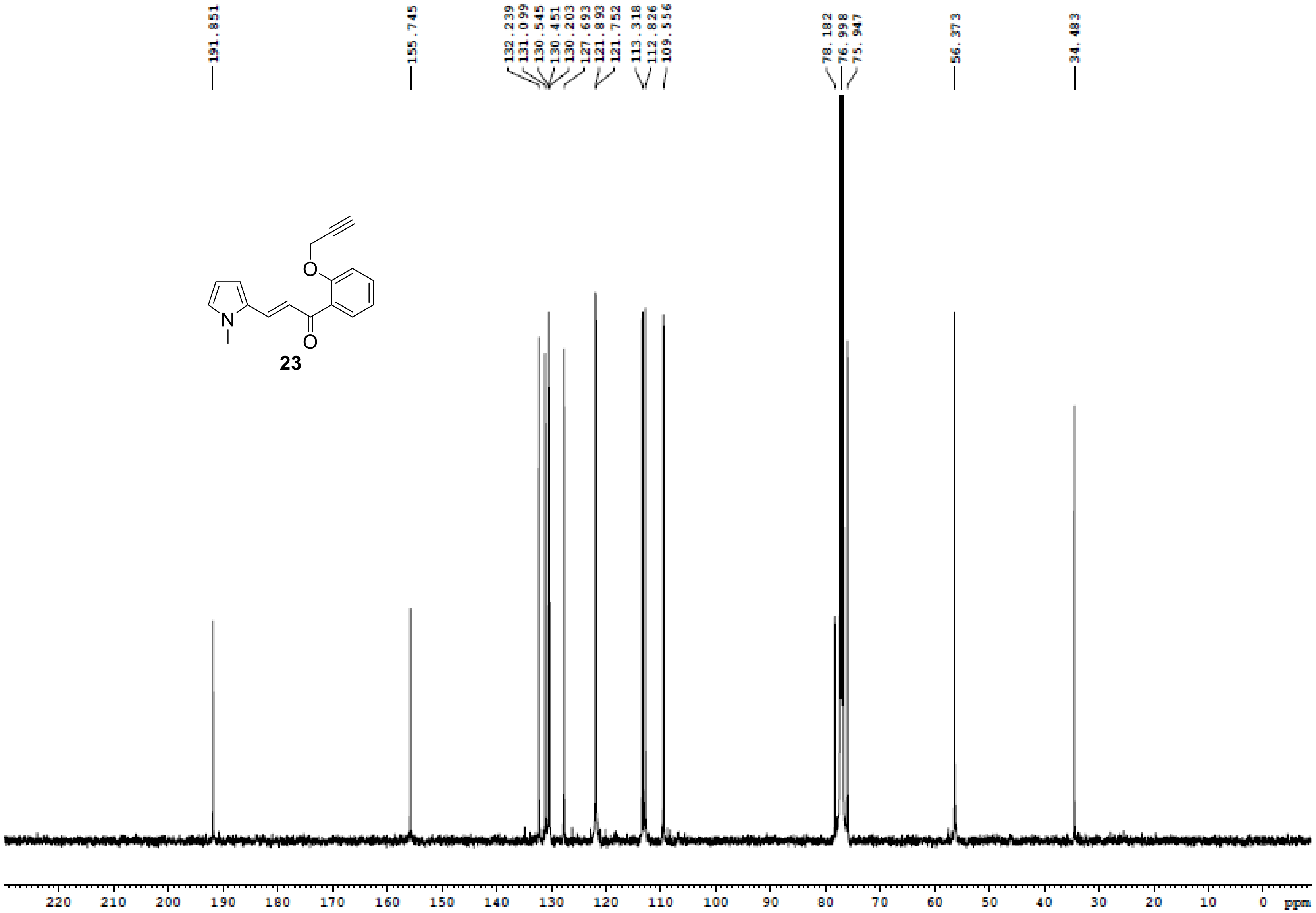
191.851
155.745
132.239
131.099
130.545
130.451
130.203
127.693
121.893
121.752
113.318
112.826
109.556
78.182
76.998
75.947
56.373
34.483
O
N
O
23
220 210 200 190 180 170 160 150 140 130 120 110 100 90 80 70 60 50 40 30 20 10 0 ppm

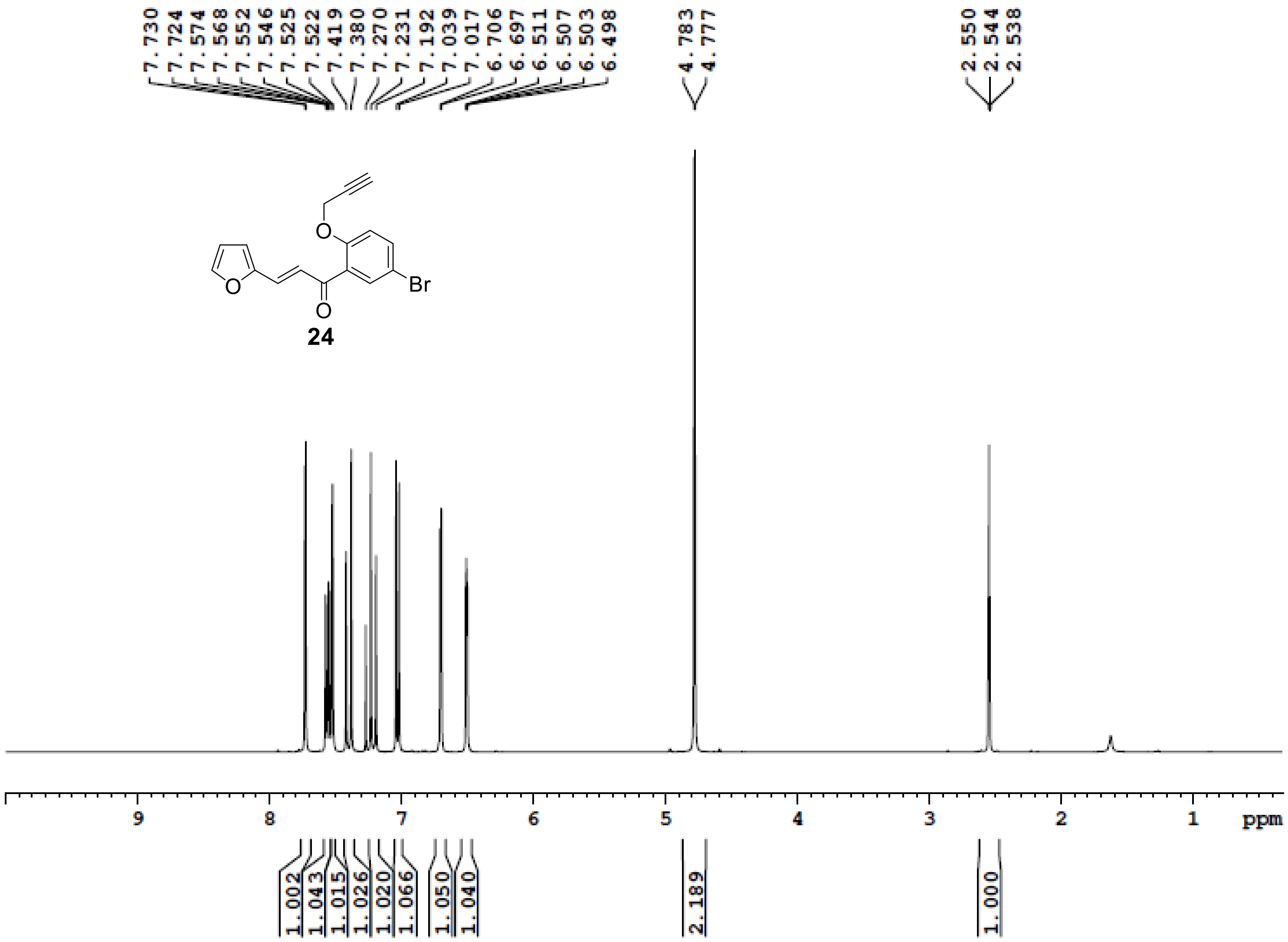
7.730
7.724
7.574
7.568
7.552
7.546
7.525
7.522
7.419
7.380
7.270
7.231
7.192
7.039
7.017
6.706
6.697
6.511
6.507
6.503
6.498
4.783
4.777
2.550
2.544
2.538
O
O
O
Br
24
9
8
7
6
5
4
3
2
1
ppm
1.002
1.043
1.015
1.026
1.020
1.066
1.050
1.040
2.189
1.000

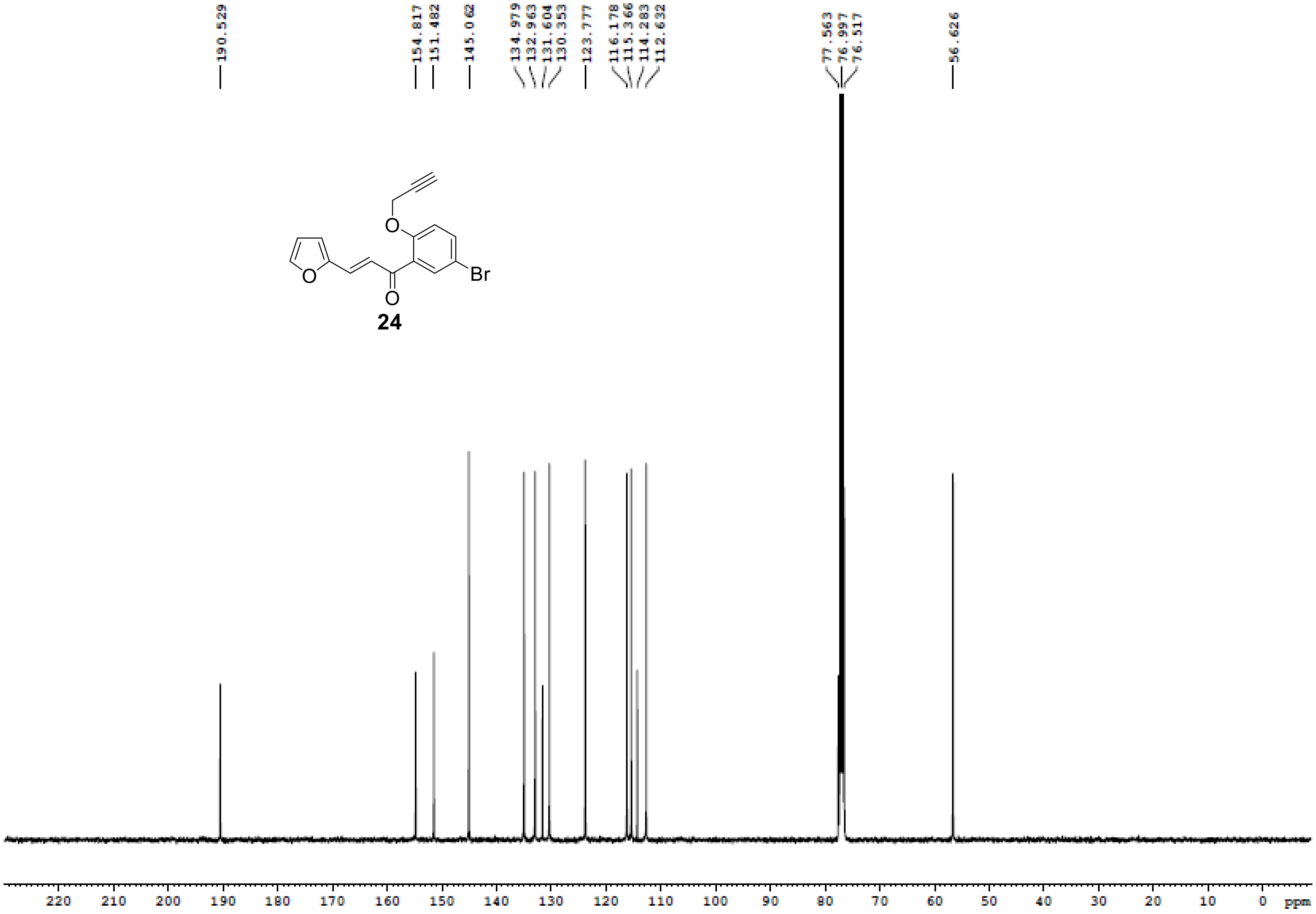
190.529
154.817
151.482
145.062
134.979
132.963
131.604
130.353
123.777
116.178
115.366
114.283
112.632
77.563
76.997
76.517
56.626
O
O
O
Br
24
220 210 200 190 180 170 160 150 140 130 120 110 100 90 80 70 60 50 40 30 20 10 0 ppm

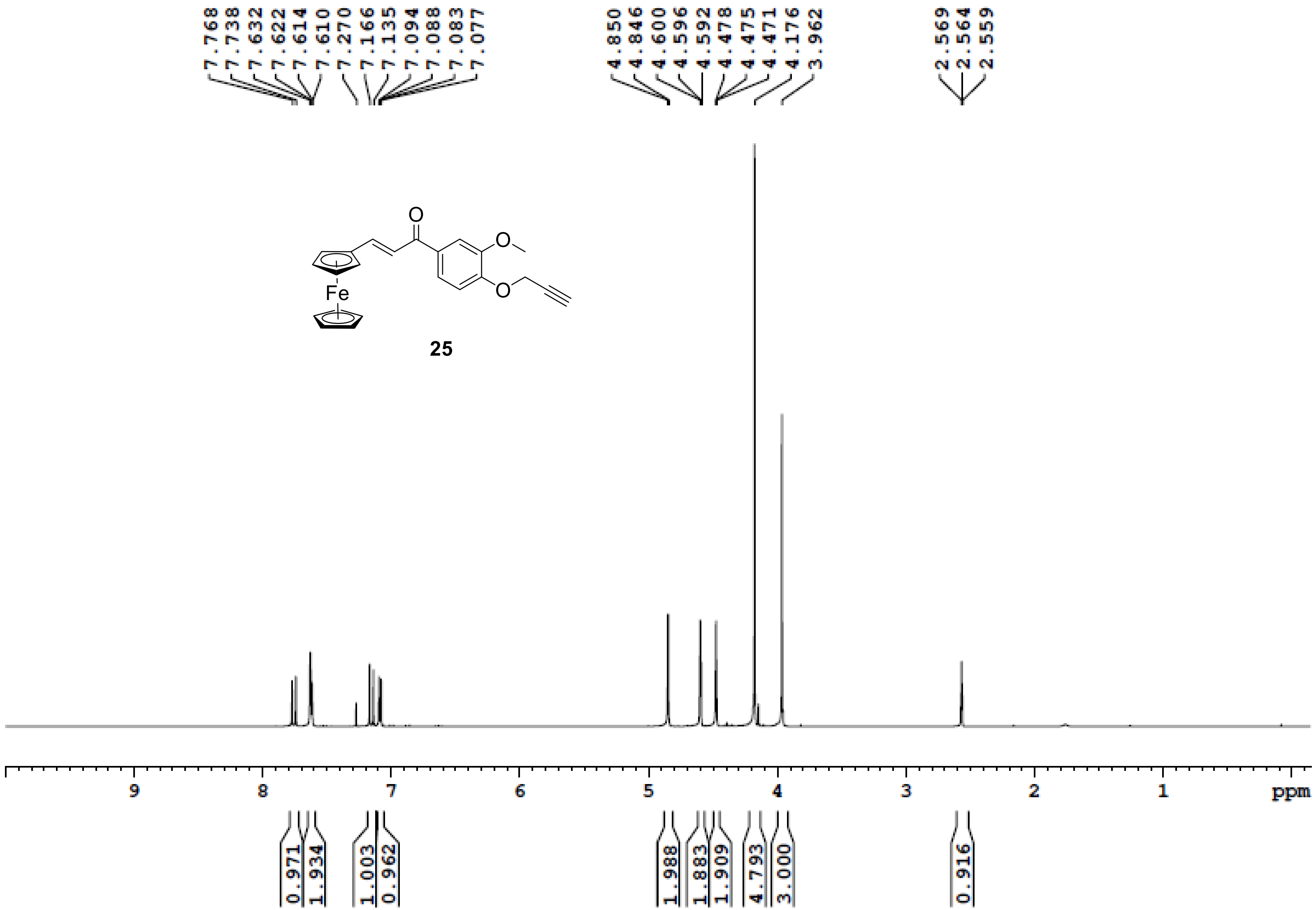
7.768
7.738
7.632
7.622
7.614
7.610
7.270
7.166
7.135
7.094
7.088
7.083
7.077
4.850
4.846
4.600
4.596
4.592
4.478
4.475
4.471
4.176
3.962
2.569
2.564
2.559
O
O
O
Fe
25
9
8
7
6
5
4
3
2
1
ppm
0.971
1.934
1.003
0.962
1.988
1.883
1.909
4.793
3.000
0.916

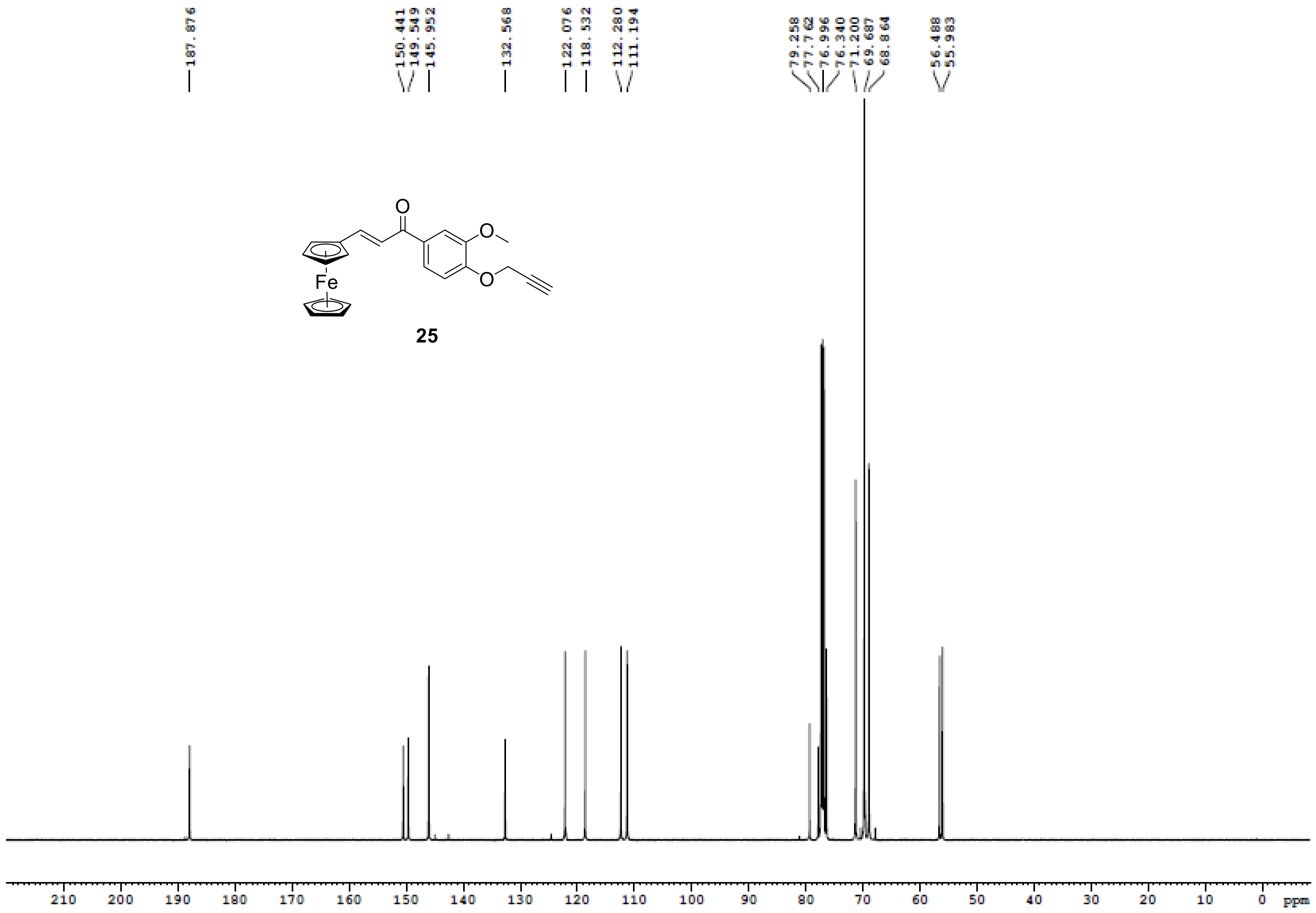
187.876
150.441
149.549
145.952
132.568
122.076
118.532
112.280
111.194
79.258
77.762
76.996
76.340
71.200
69.687
68.864
56.488
55.983
O
O
Fe
O
25
210 200 190 180 170 160 150 140 130 120 110 100 90 80 70 60 50 40 30 20 10 0 ppm

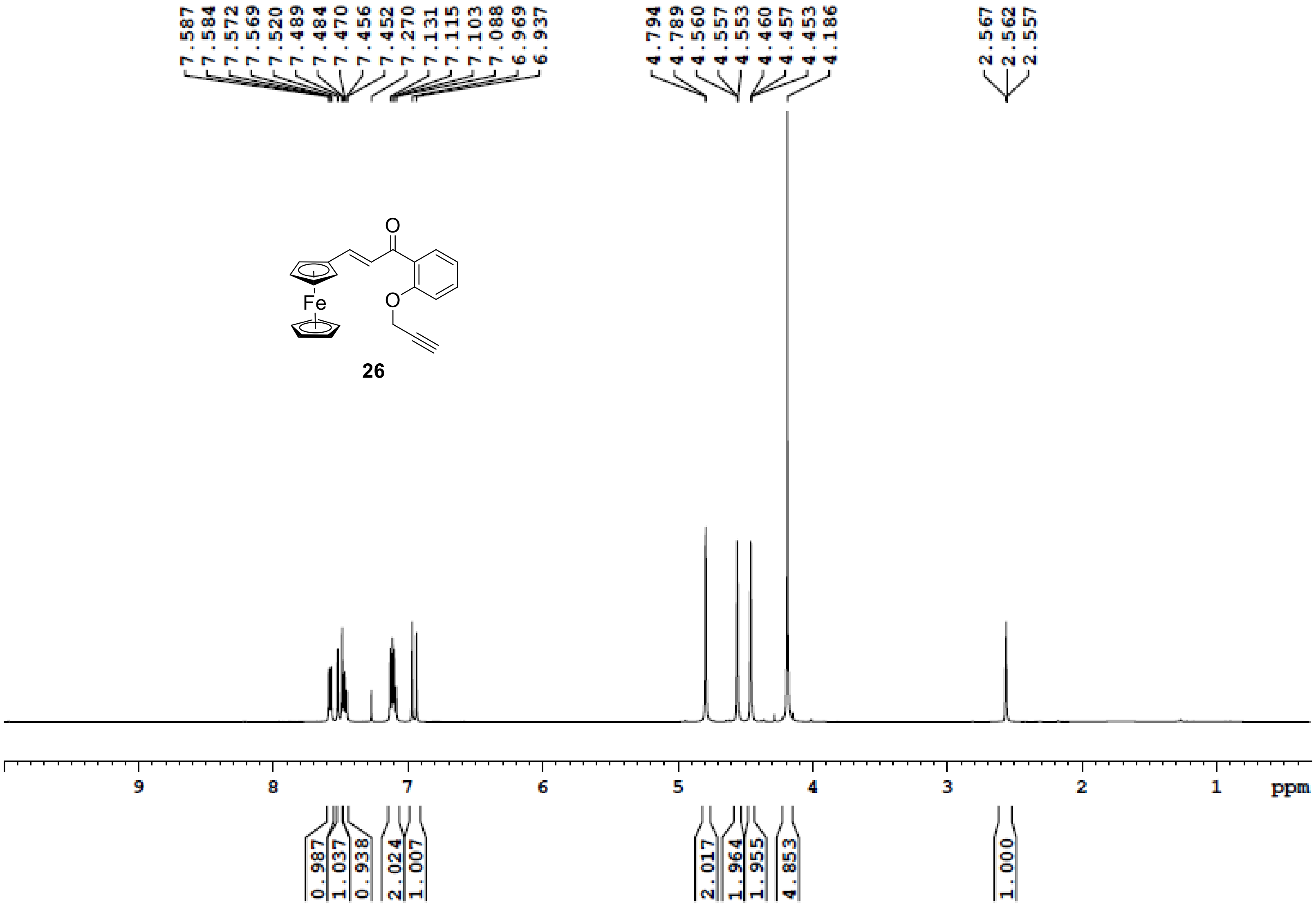
7.587
7.584
7.572
7.569
7.520
7.489
7.484
7.470
7.456
7.452
7.270
7.131
7.115
7.103
7.088
6.969
6.937
4.794
4.789
4.560
4.557
4.553
4.460
4.457
4.453
4.186
2.567
2.562
2.557
O
Fe
O
26
9
8
7
6
5
4
3
2
1
ppm
0.987
1.037
0.938
2.024
1.007
2.017
1.964
1.955
4.853
1.000

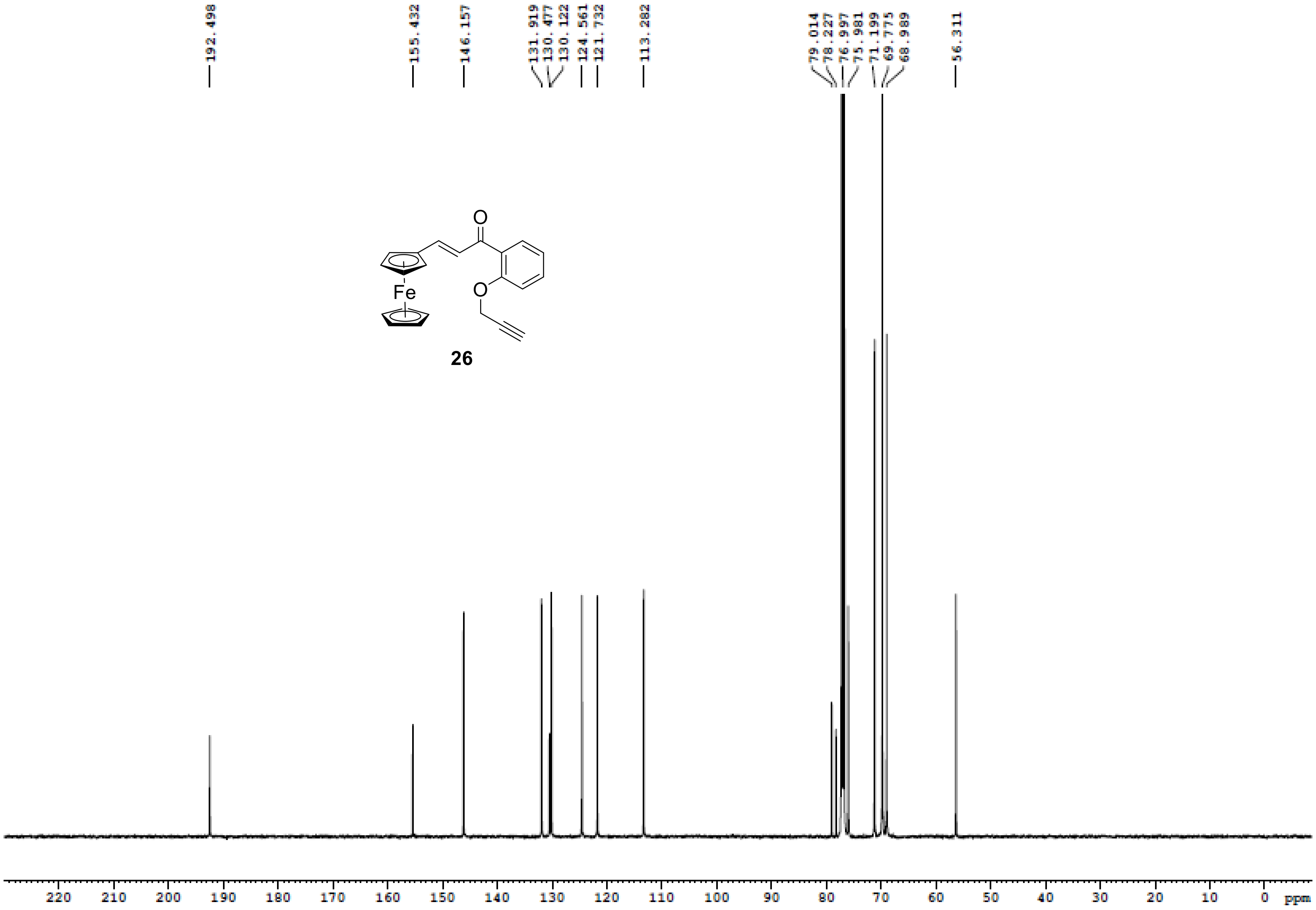
192.498
155.432
146.157
131.919
130.477
130.122
124.561
121.732
113.282
79.014
78.227
76.997
75.981
71.199
69.775
68.989
56.311
O
Fe
O
26
220 210 200 190 180 170 160 150 140 130 120 110 100 90 80 70 60 50 40 30 20 10 0 ppm

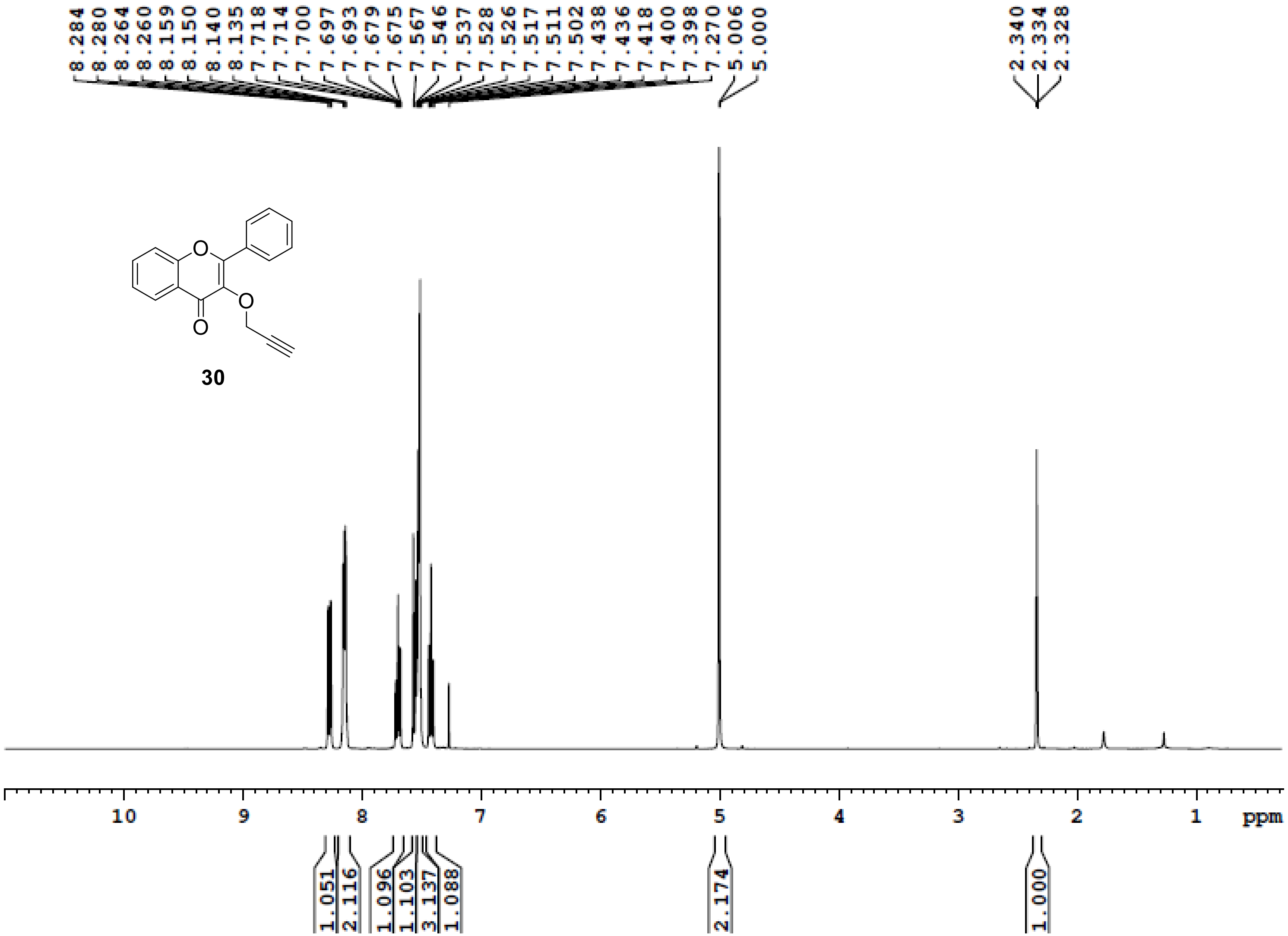
8.284
8.280
8.264
8.260
8.159
8.150
8.140
8.135
7.718
7.714
7.700
7.697
7.693
7.679
7.675
7.567
7.546
7.537
7.528
7.526
7.517
7.511
7.502
7.438
7.436
7.418
7.400
7.398
7.270
5.006
5.000
2.340
2.334
2.328
O
O
O
30
10
9
8
7
6
5
4
3
2
1
ppm
1.051
2.116
1.096
1.103
3.137
1.088
2.174
1.000

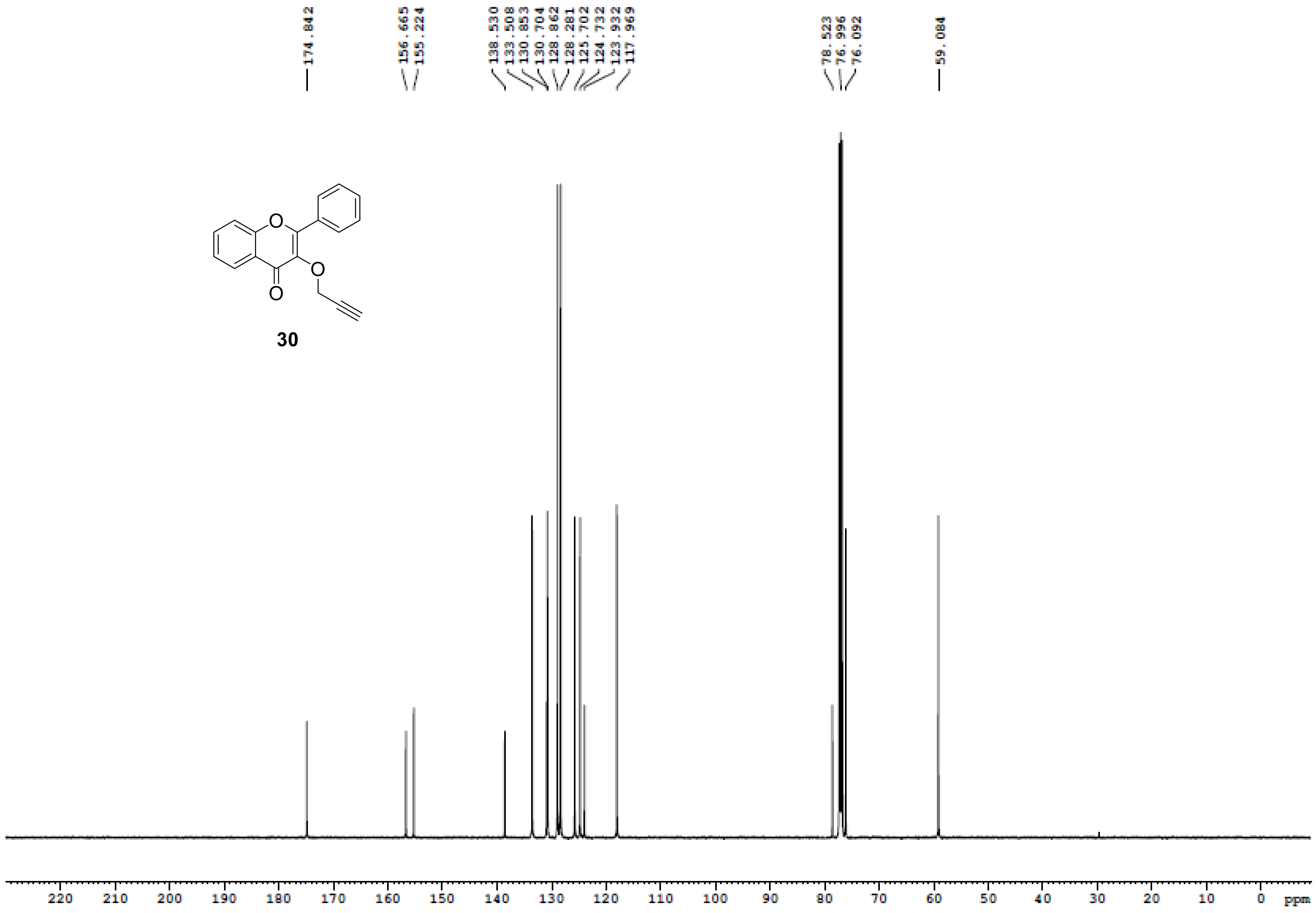
174.842
156.665
155.224
138.530
133.508
130.853
130.704
128.862
128.281
125.702
124.732
123.932
117.969
78.523
76.996
76.092
59.084
30
220 210 200 190 180 170 160 150 140 130 120 110 100 90 80 70 60 50 40 30 20 10 0 ppm

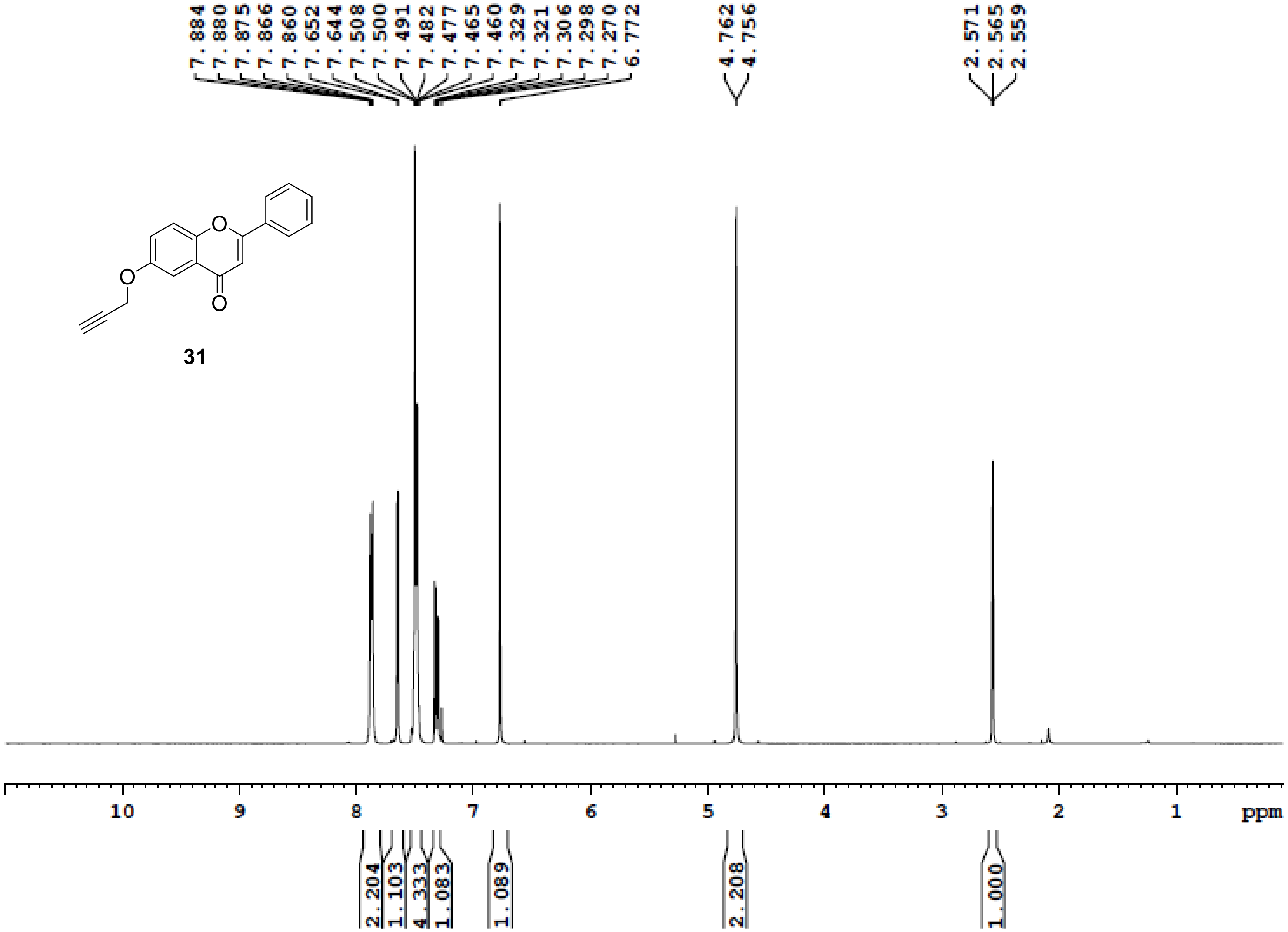
7.884
7.880
7.875
7.866
7.860
7.652
7.644
7.508
7.500
7.491
7.482
7.477
7.465
7.460
7.329
7.321
7.306
7.298
7.270
6.772
4.762
4.756
2.571
2.565
2.559
31
10
9
8
7
6
5
4
3
2
1
ppm
2.204
1.103
4.333
1.083
1.089
2.208
1.000

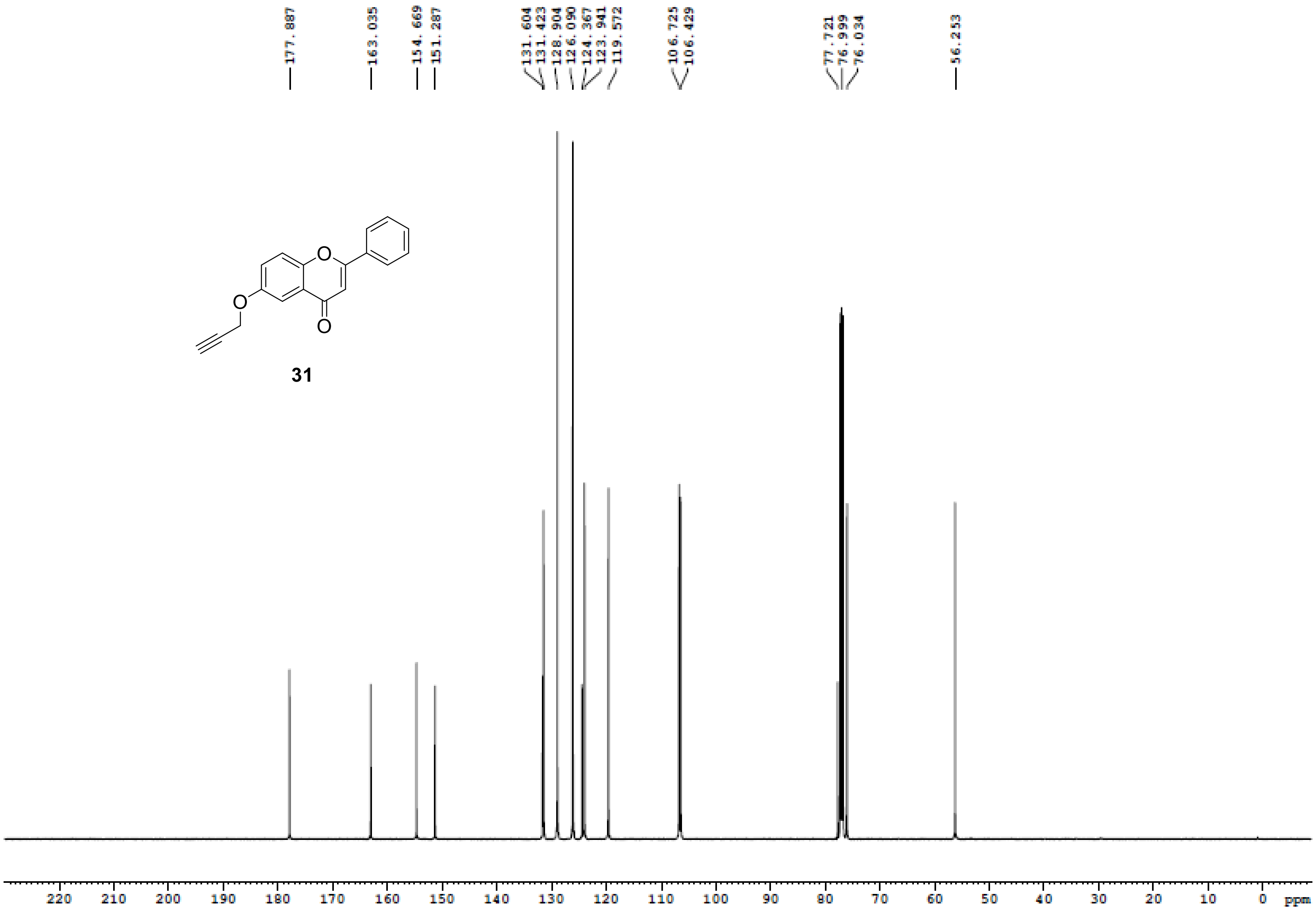
177.887
163.035
154.669
151.287
131.604
131.423
128.904
126.090
124.367
123.941
119.572
106.725
106.429
77.721
76.999
76.034
56.253
O
O
O
31
220 210 200 190 180 170 160 150 140 130 120 110 100 90 80 70 60 50 40 30 20 10 0 ppm

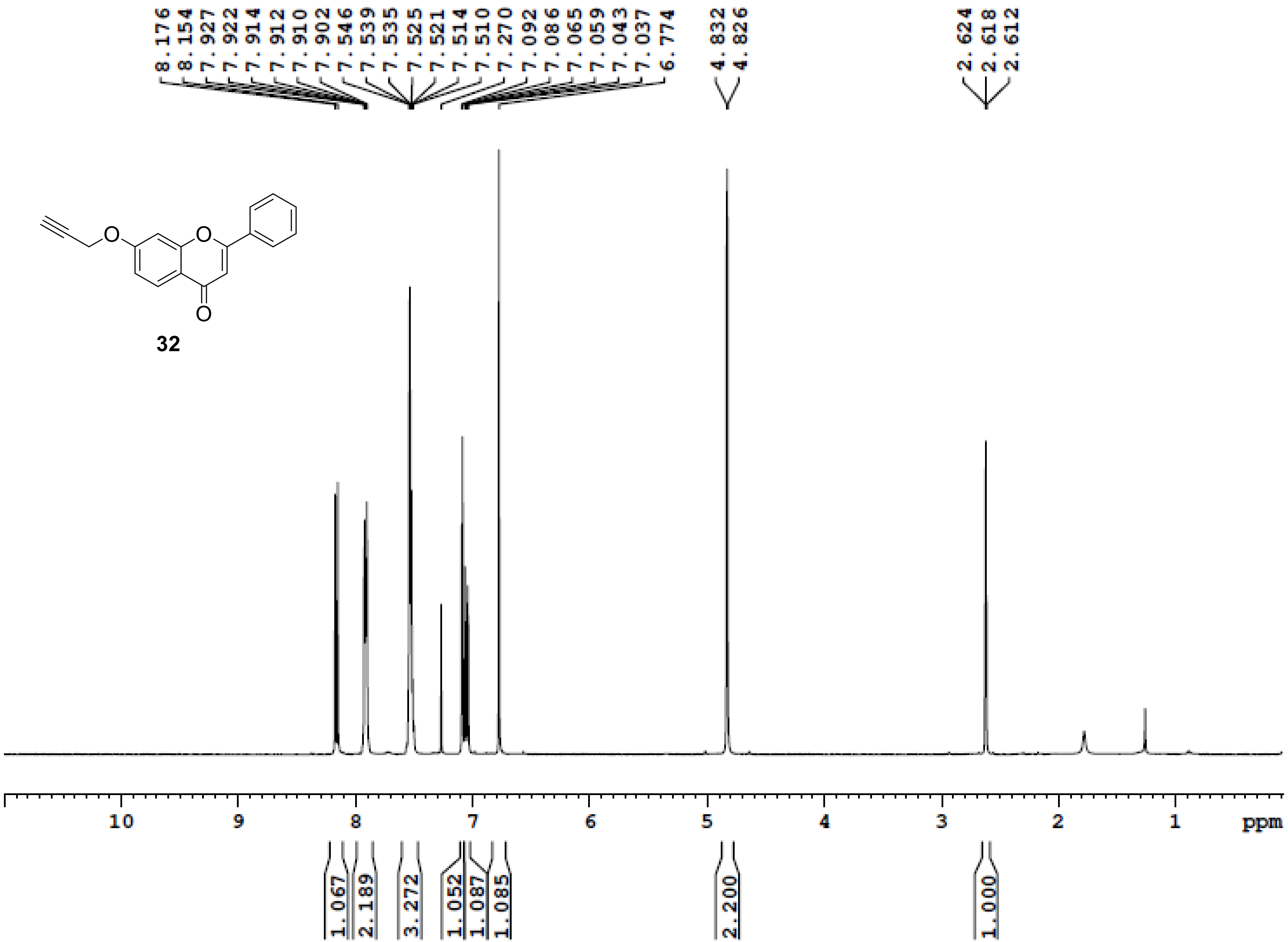
8.176
8.154
7.927
7.922
7.914
7.912
7.910
7.902
7.546
7.539
7.535
7.525
7.521
7.514
7.510
7.270
7.092
7.086
7.065
7.059
7.043
7.037
6.774
4.832
4.826
2.624
2.618
2.612
32
10 9 8 7 6 5 4 3 2 1 ppm
1.067
2.189
3.272
1.052
1.087
1.085
2.200
1.000

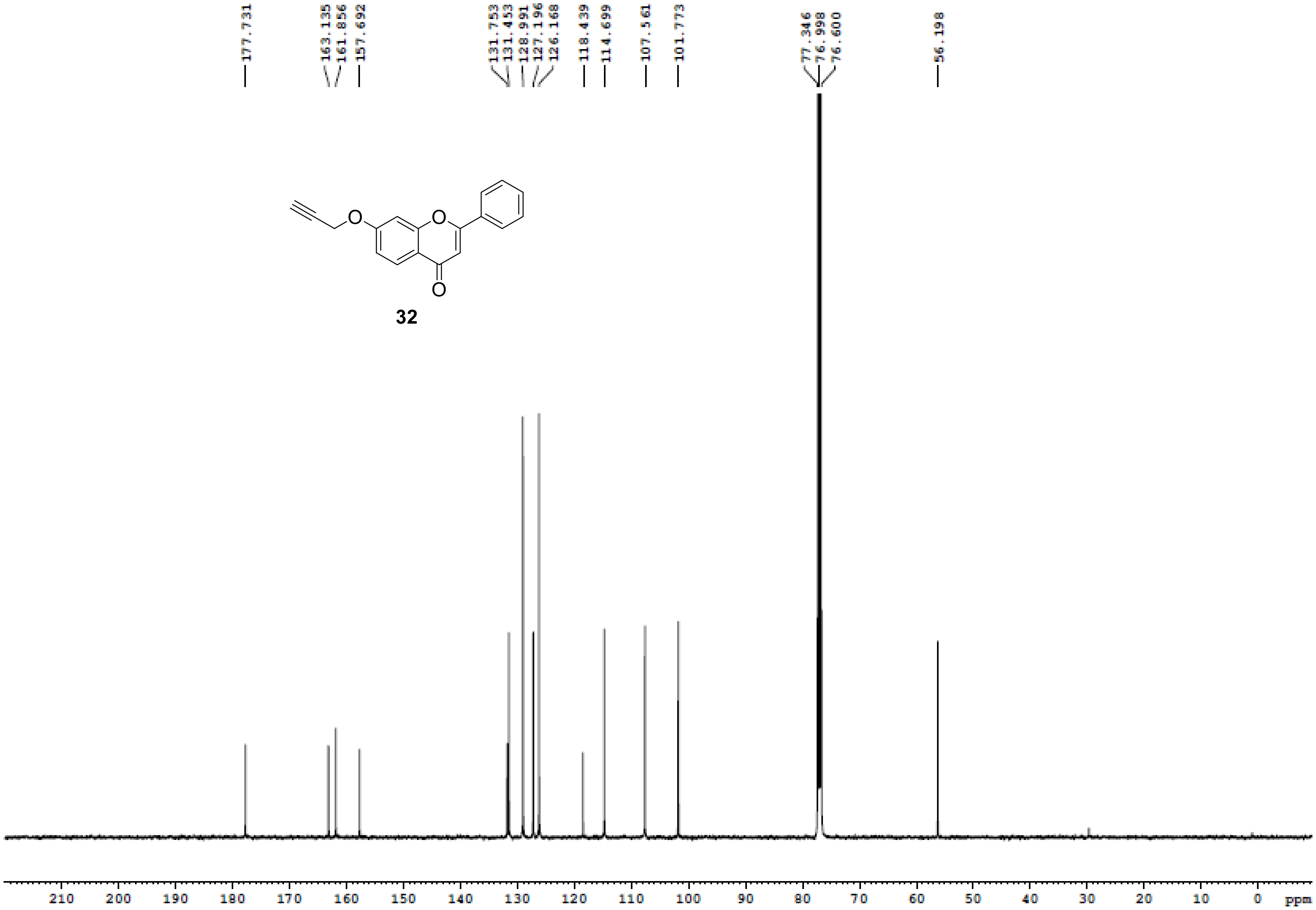
177.731
163.135
161.856
157.692
131.753
131.453
128.991
127.196
126.168
118.439
114.699
107.561
101.773
77.346
76.998
76.600
56.198
32
210 200 190 180 170 160 150 140 130 120 110 100 90 80 70 60 50 40 30 20 10 0 ppm

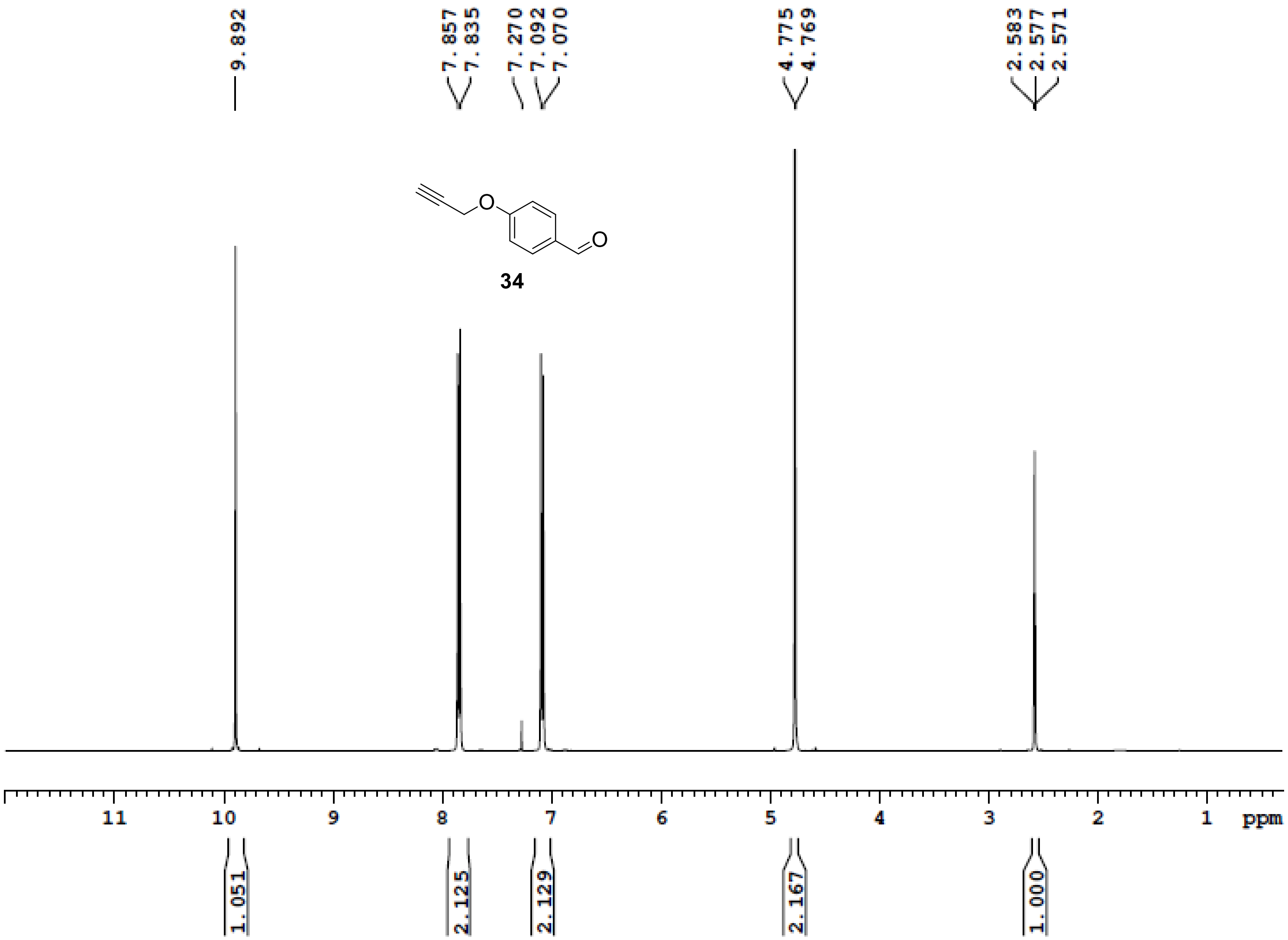
9.892
7.857
7.835
7.270
7.092
7.070
4.775
4.769
2.583
2.577
2.571
O
O
34
11
10
9
8
7
6
5
4
3
2
1
ppm
1.051
2.125
2.129
2.167
1.000

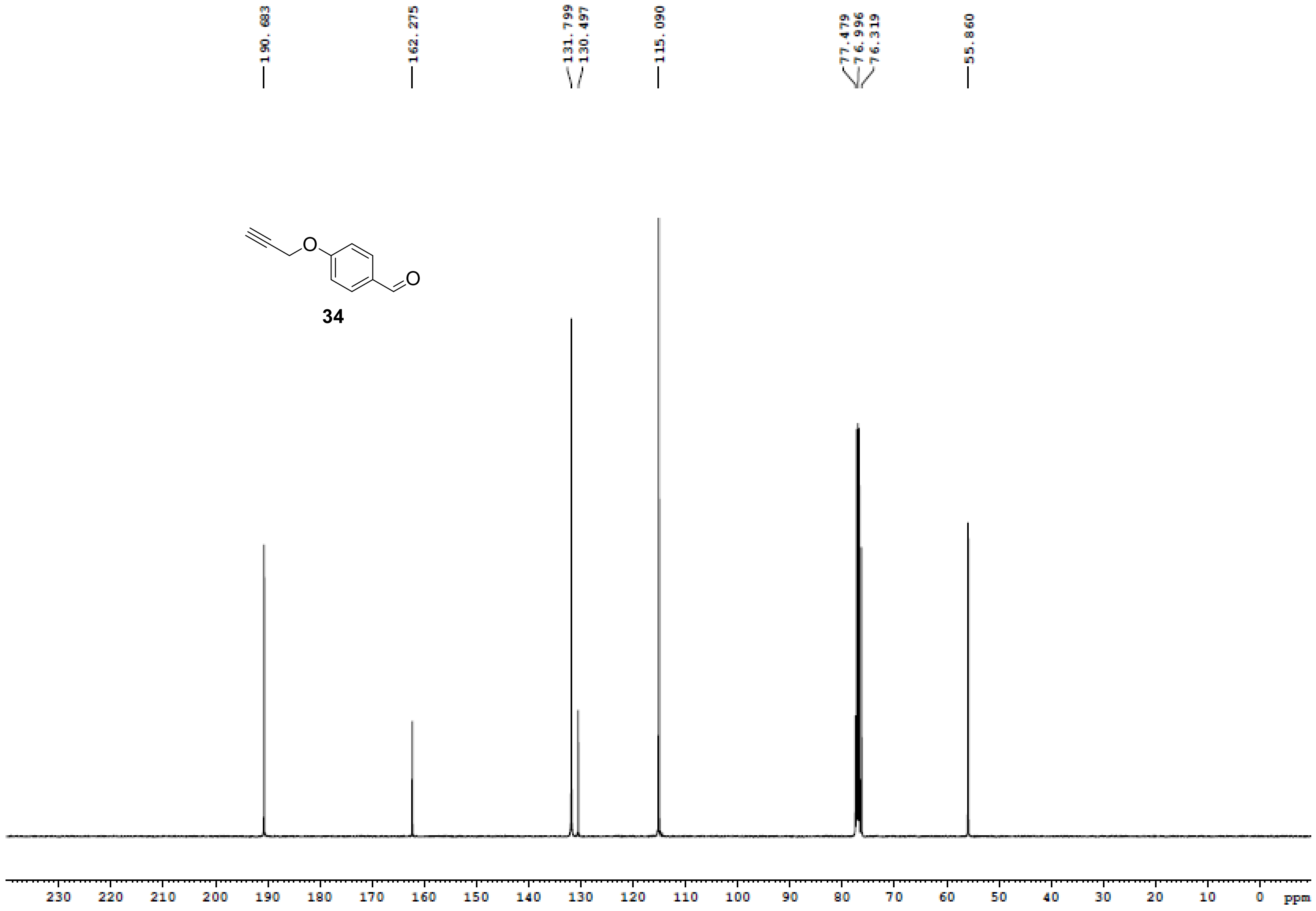
190.683
162.275
131.799
130.497
115.090
77.479
76.996
76.319
55.860
O
O
34
230 220 210 200 190 180 170 160 150 140 130 120 110 100 90 80 70 60 50 40 30 20 10 0 ppm

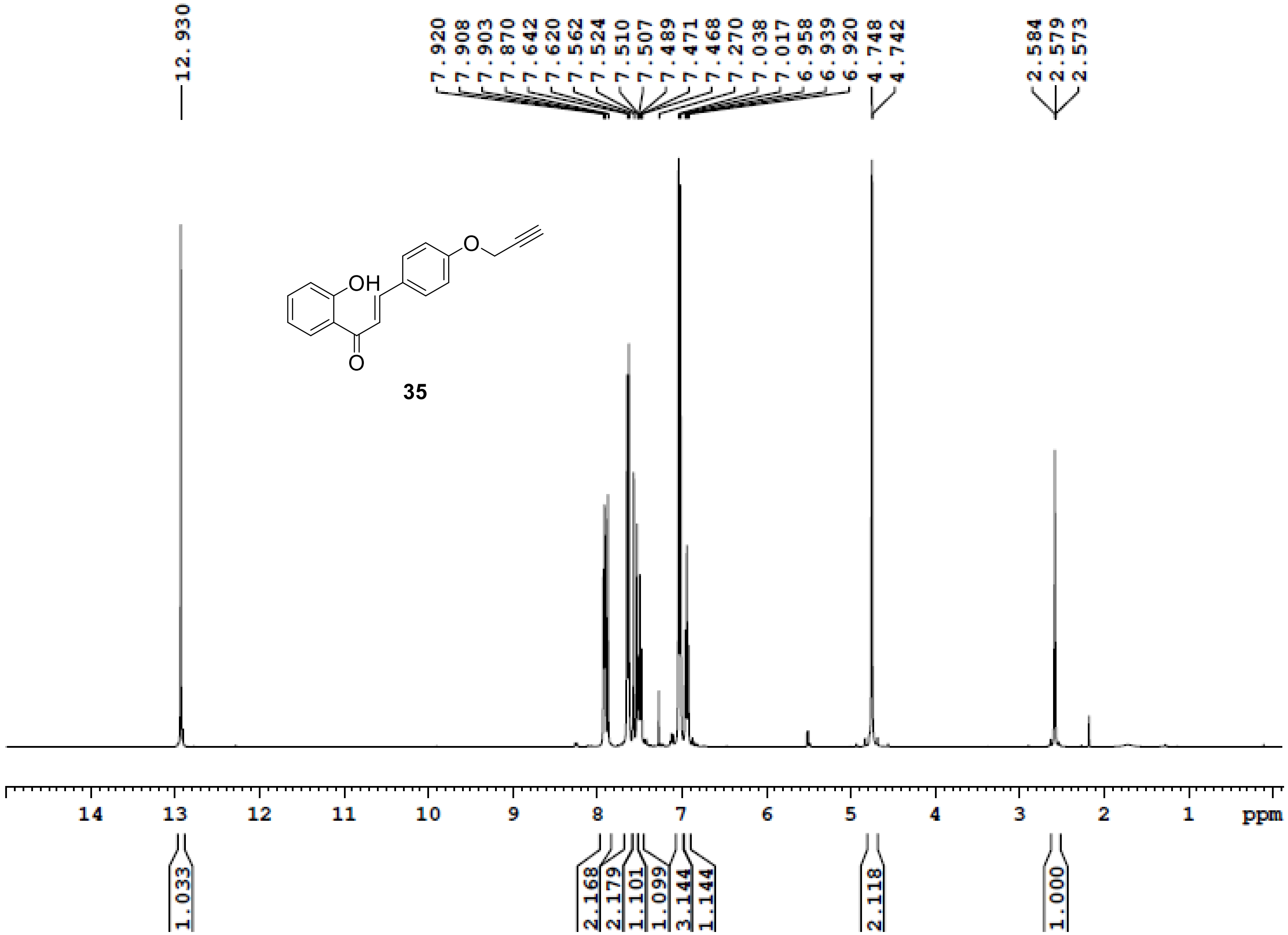

12.930
7.920
7.908
7.903
7.870
7.642
7.620
7.562
7.524
7.510
7.507
7.489
7.471
7.468
7.270
7.038
7.017
6.958
6.939
6.920
4.748
4.742
2.584
2.579
2.573
OH
O
O
35
14
13
12
11
10
9
8
7
6
5
4
3
2
1
ppm
1.033
2.168
2.179
1.101
1.099
3.144
1.144
2.118
1.000

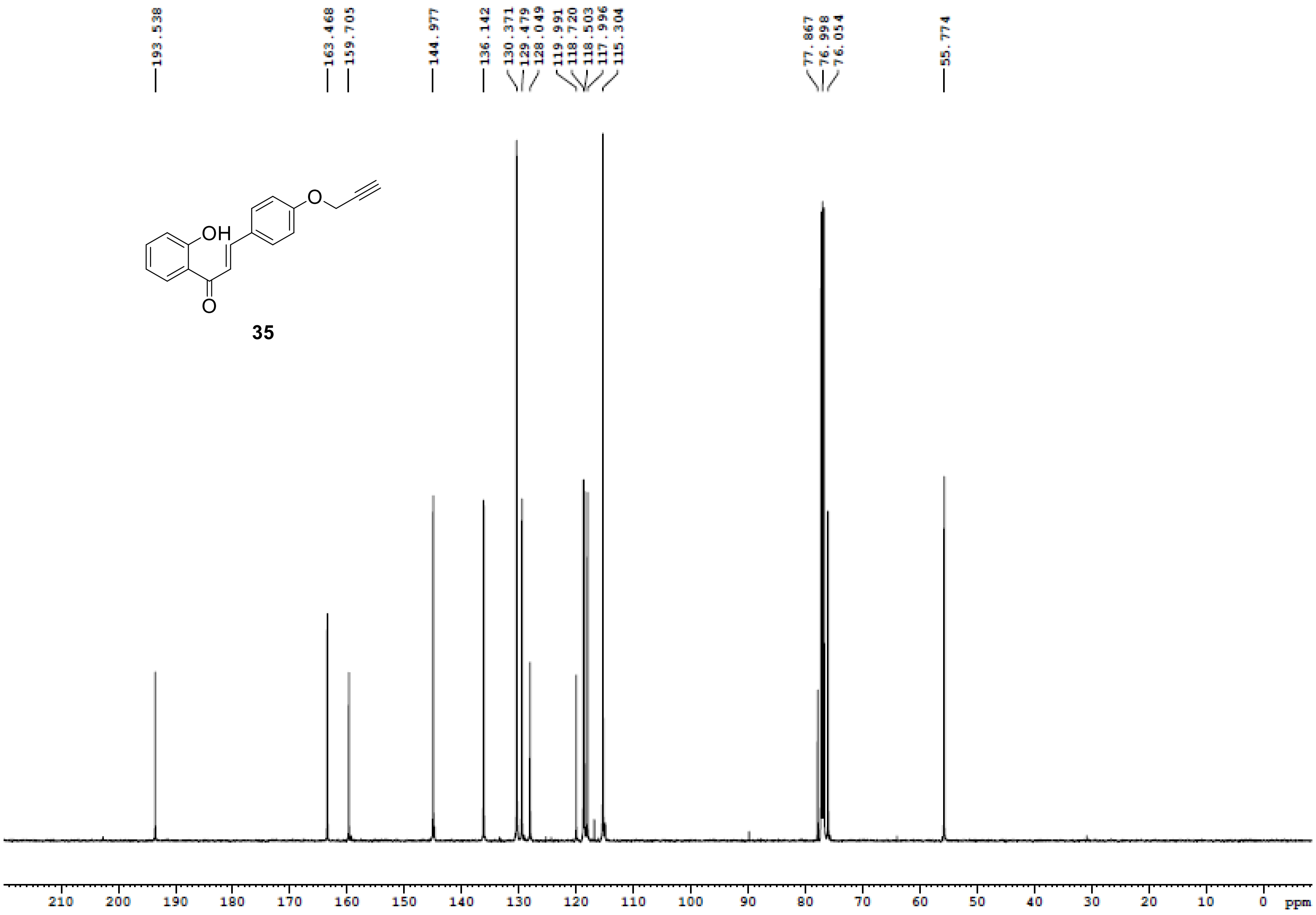
193.538
163.468
159.705
144.977
136.142
130.371
129.479
128.049
119.991
118.720
118.503
117.996
115.304
77.867
76.998
76.054
55.774
OH
O
35
210 200 190 180 170 160 150 140 130 120 110 100 90 80 70 60 50 40 30 20 10 0 ppm

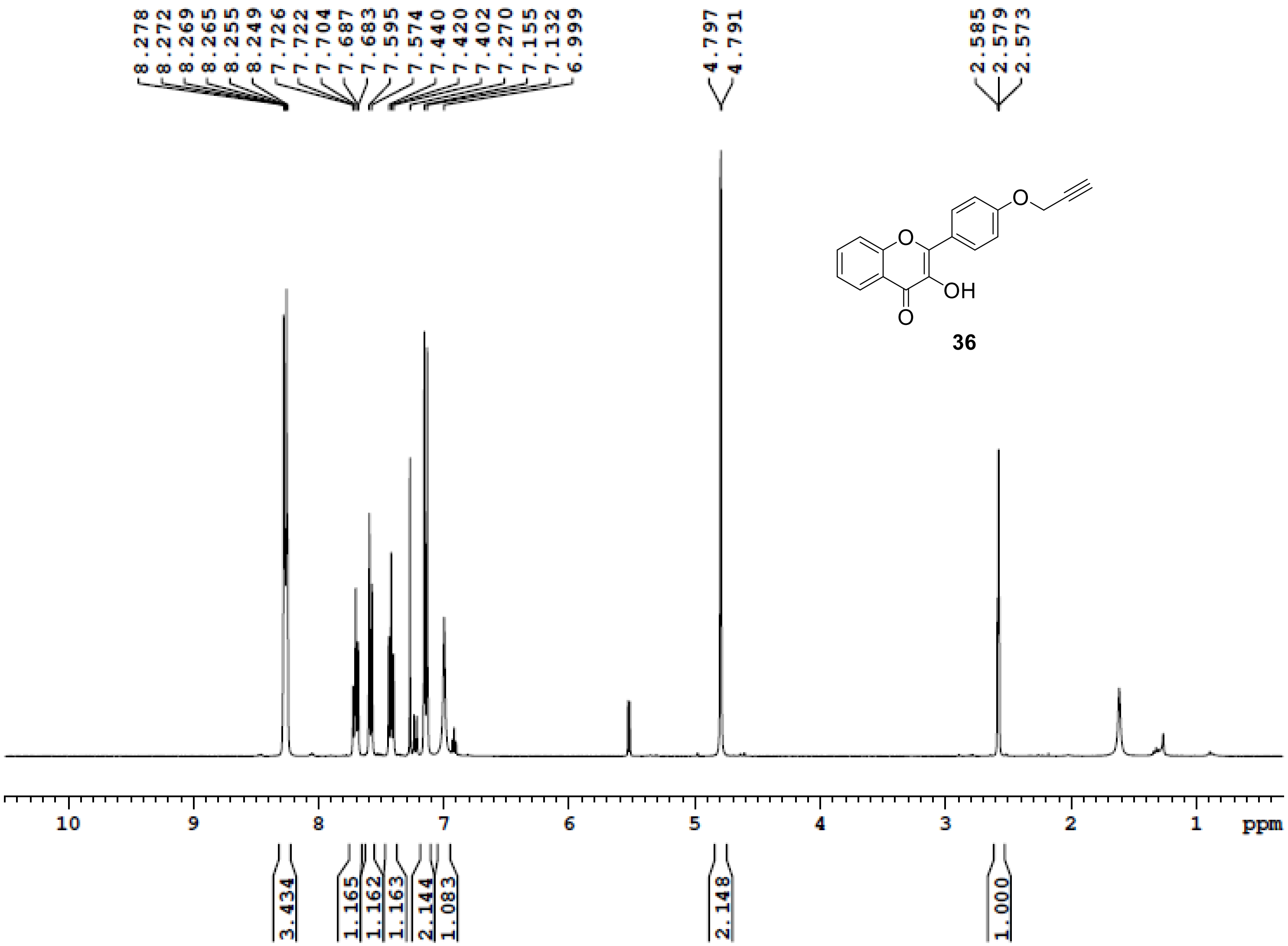
8.278
8.272
8.269
8.265
8.255
8.249
7.726
7.722
7.704
7.687
7.683
7.595
7.574
7.440
7.420
7.402
7.270
7.155
7.132
6.999
4.797
4.791
2.585
2.579
2.573
O
OH
O
O
36
10
9
8
7
6
5
4
3
2
1
ppm
3.434
1.165
1.162
1.163
2.144
1.083
2.148
1.000

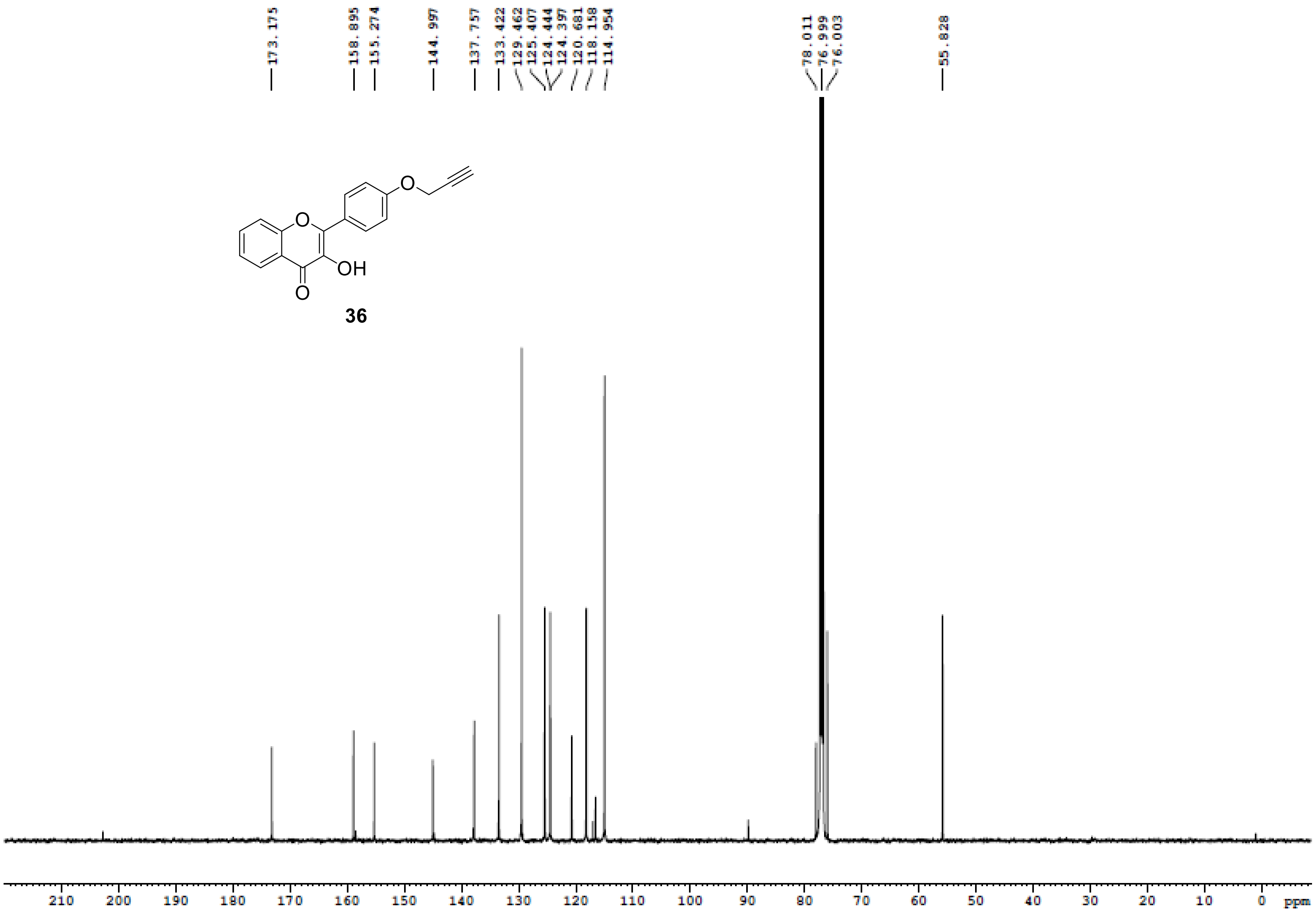
173.175
158.895
155.274
144.997
137.757
133.422
129.462
125.407
124.444
124.397
120.681
118.158
114.954
78.011
76.999
76.003
55.828
O
O
OH
O
36
210 200 190 180 170 160 150 140 130 120 110 100 90 80 70 60 50 40 30 20 10 0 ppm

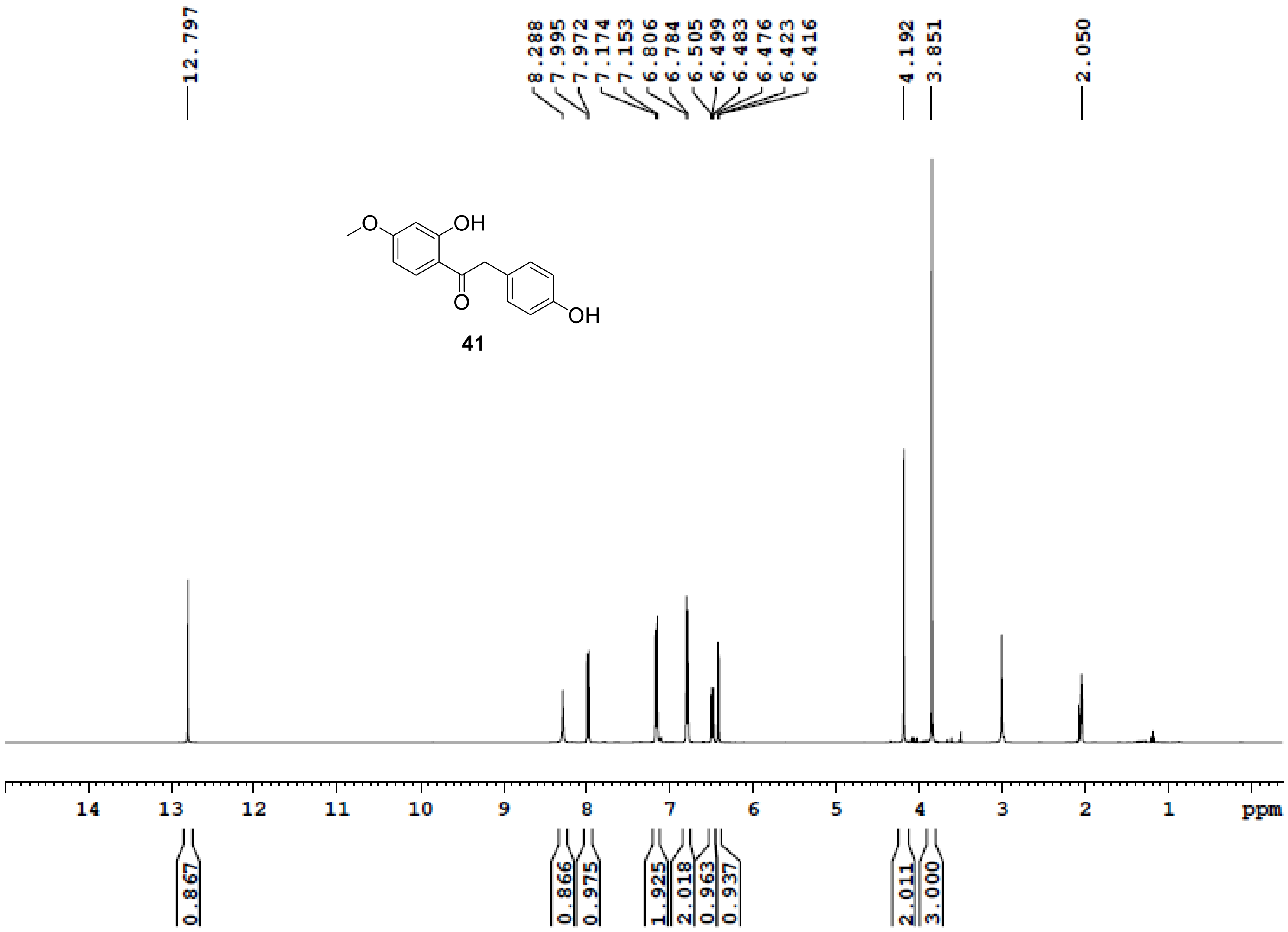
12.797
8.288
7.995
7.972
7.174
7.153
6.806
6.784
6.505
6.499
6.483
6.476
6.423
6.416
4.192
3.851
2.050
OH
O
OH
41
14 13 12 11 10 9 8 7 6 5 4 3 2 1 ppm
0.867
0.866
0.975
1.925
2.018
0.963
0.937
2.011
3.000

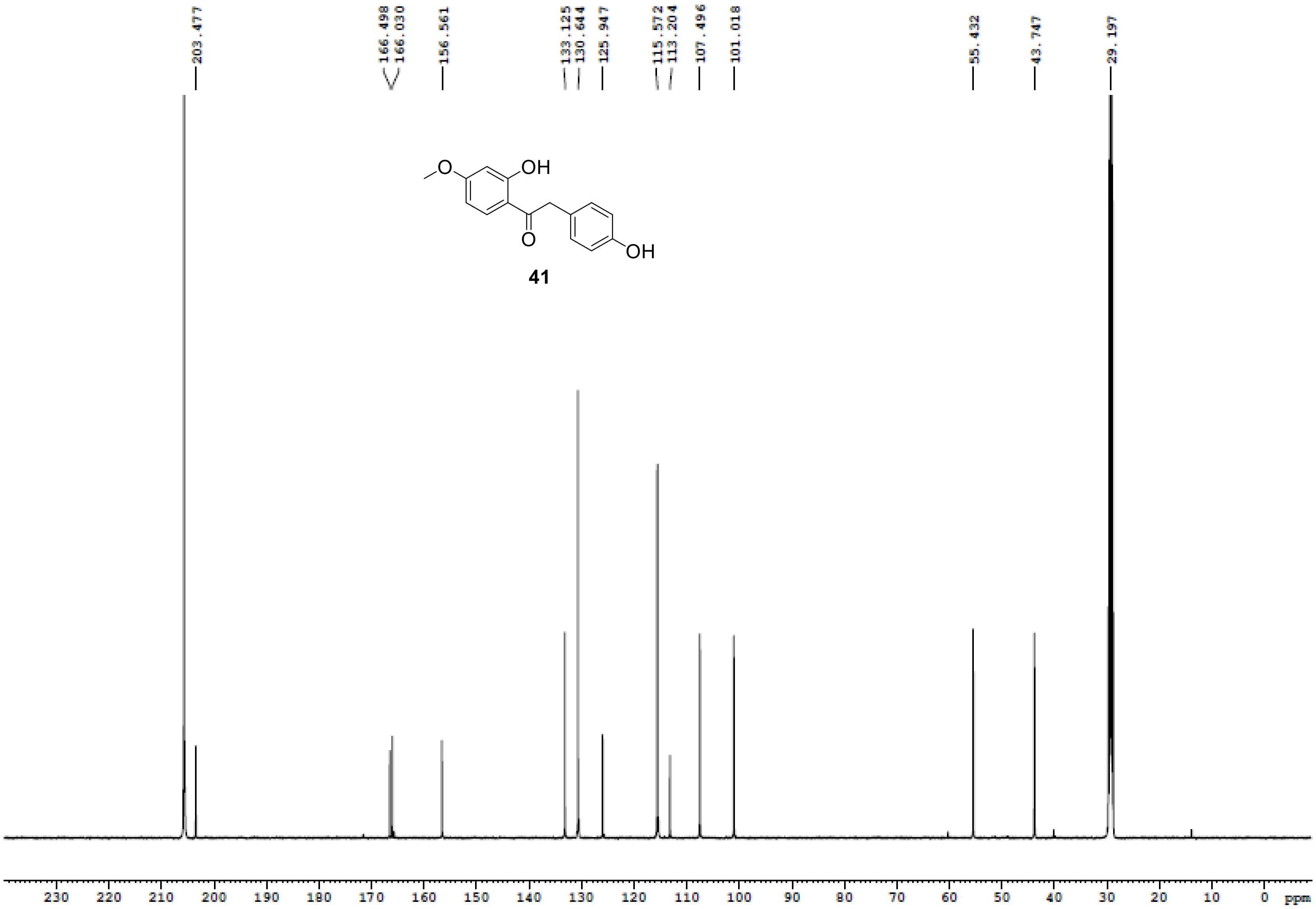

203.477
166.498
166.030
156.561
133.125
130.644
125.947
115.572
113.204
107.496
101.018
55.432
43.747
29.197
OH
O
O
OH
41
230
220
210
200
190
180
170
160
150
140
130
120
110
100
90
80
70
60
50
40
30
20
10
0
ppm

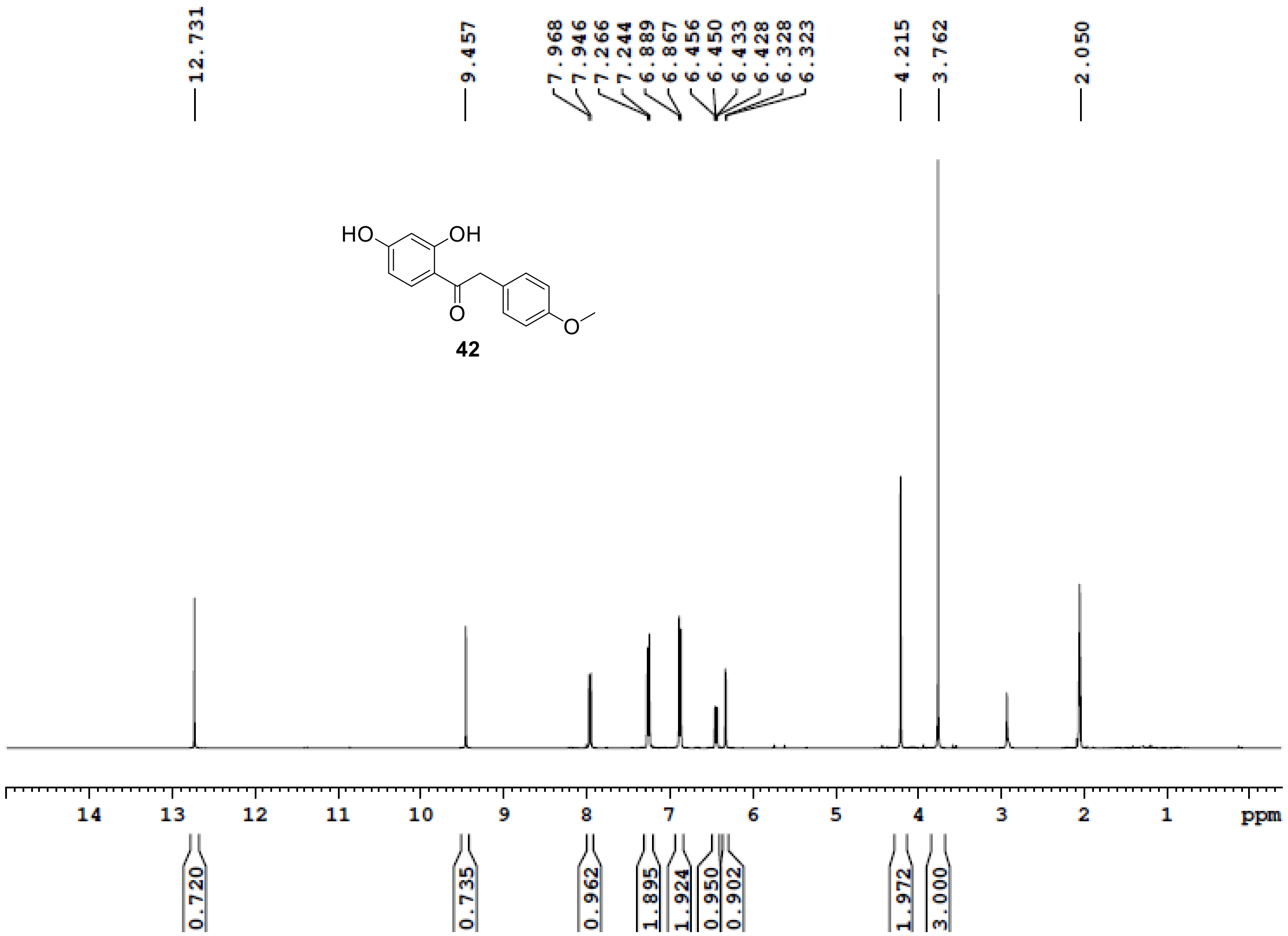
12.731
9.457
7.968
7.946
7.266
7.244
6.889
6.867
6.456
6.450
6.433
6.428
6.328
6.323
4.215
3.762
2.050
HO
OH
O
O
42
14
13
12
11
10
9
8
7
6
5
4
3
2
1
ppm
0.720
0.735
0.962
1.895
1.924
0.950
0.902
1.972
3.000

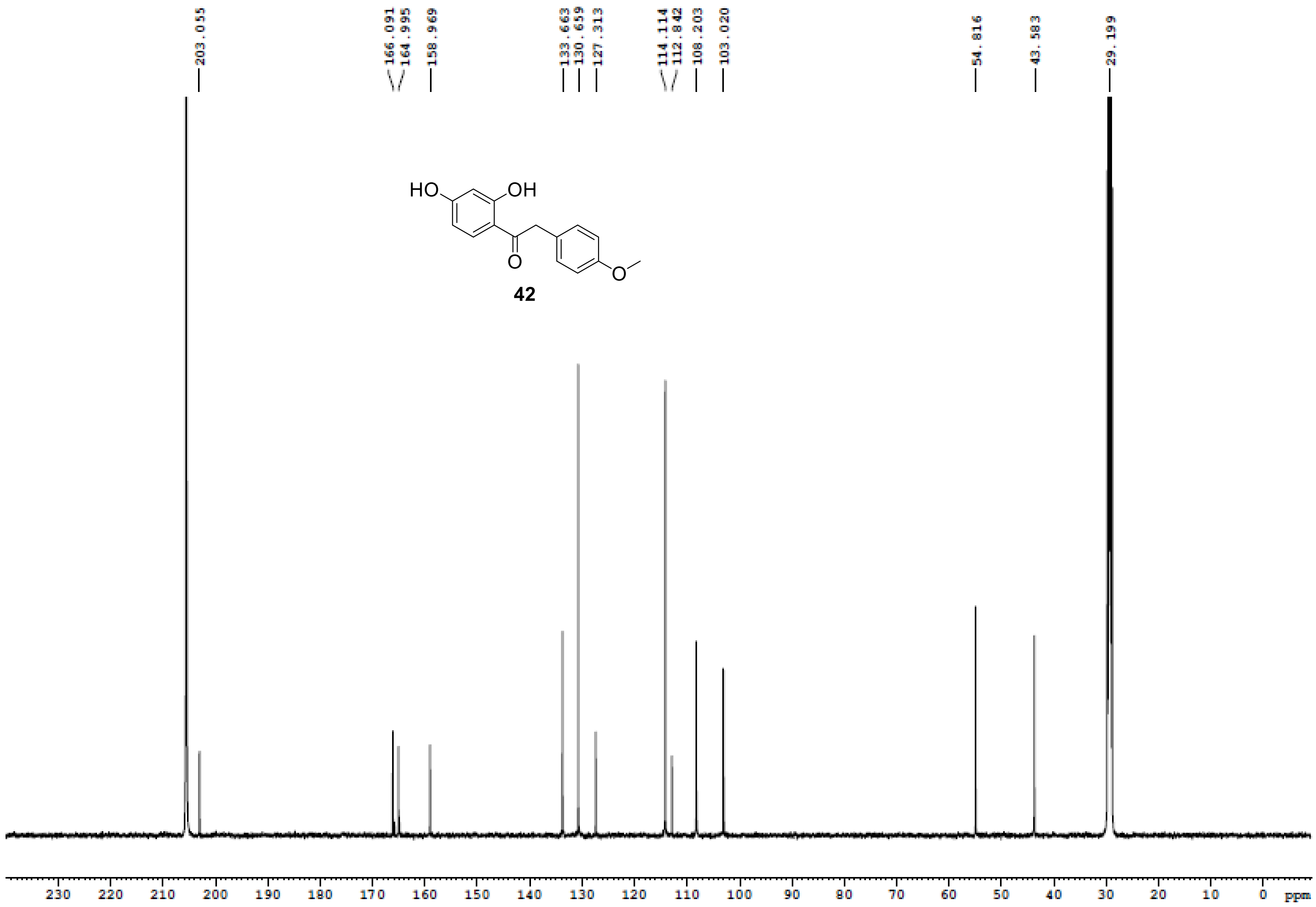
203.055
166.091
164.995
158.969
133.663
130.659
127.313
114.114
112.842
108.203
103.020
54.816
43.583
29.199
HO
OH
O
O
42
230 220 210 200 190 180 170 160 150 140 130 120 110 100 90 80 70 60 50 40 30 20 10 0 ppm

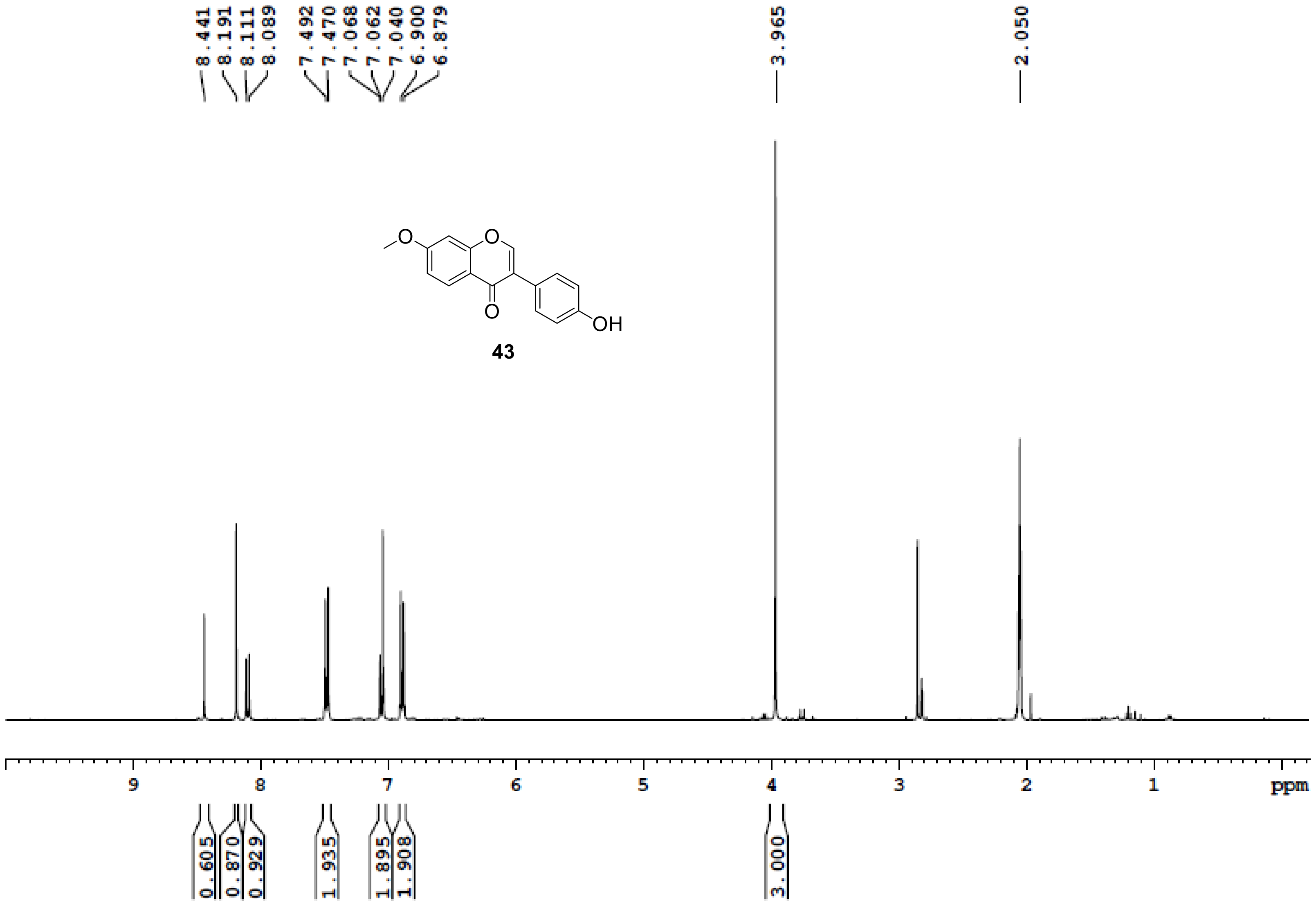
8.441
8.191
8.111
8.089
7.492
7.470
7.068
7.062
7.040
6.900
6.879
3.965
2.050
OH
43
9
8
7
6
5
4
3
2
1
ppm
0.605
0.870
0.929
1.935
1.895
1.908
3.000

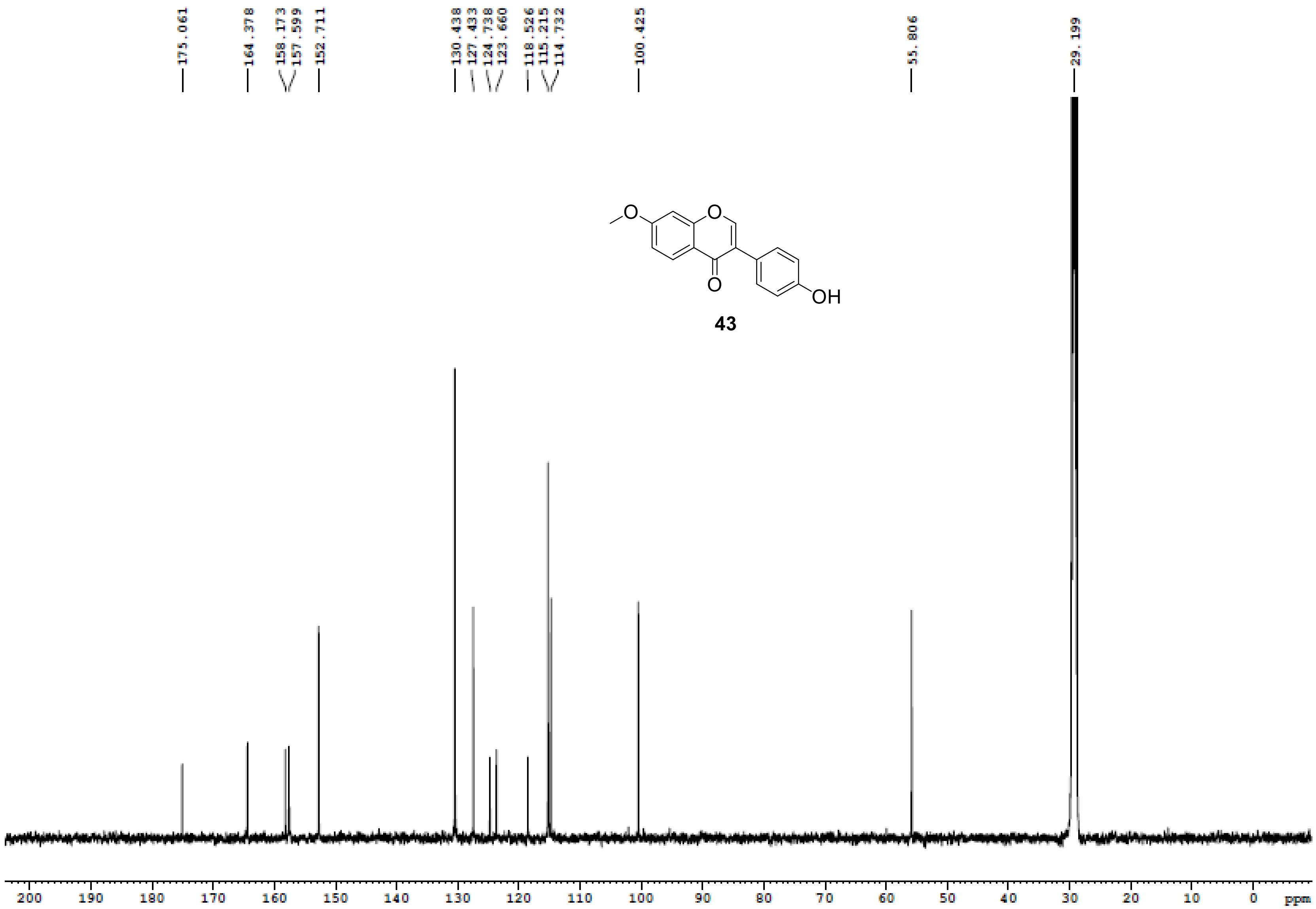

175.061
164.378
158.173
157.599
152.711
130.438
127.433
124.738
123.660
118.526
115.215
114.732
100.425
55.806
29.199
O
O
O
OH
43
200 190 180 170 160 150 140 130 120 110 100 90 80 70 60 50 40 30 20 10 0 ppm

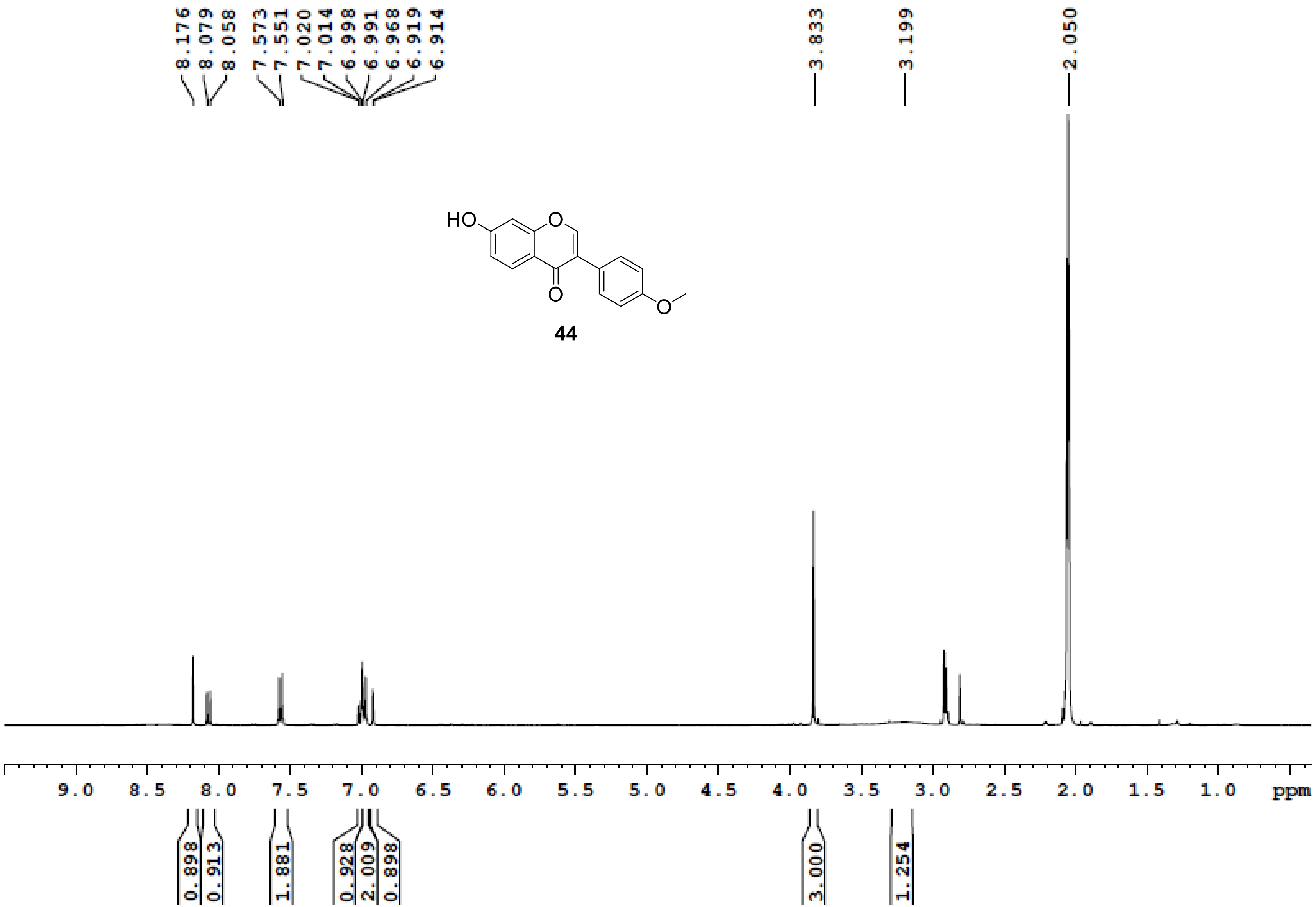
8.176
8.079
8.058
7.573
7.551
7.020
7.014
6.998
6.991
6.968
6.919
6.914
3.833
3.199
2.050
HO
O
O
O
44
9.0 8.5 8.0 7.5 7.0 6.5 6.0 5.5 5.0 4.5 4.0 3.5 3.0 2.5 2.0 1.5 1.0 ppm
0.898
0.913
1.881
0.928
2.009
0.898
3.000
1.254

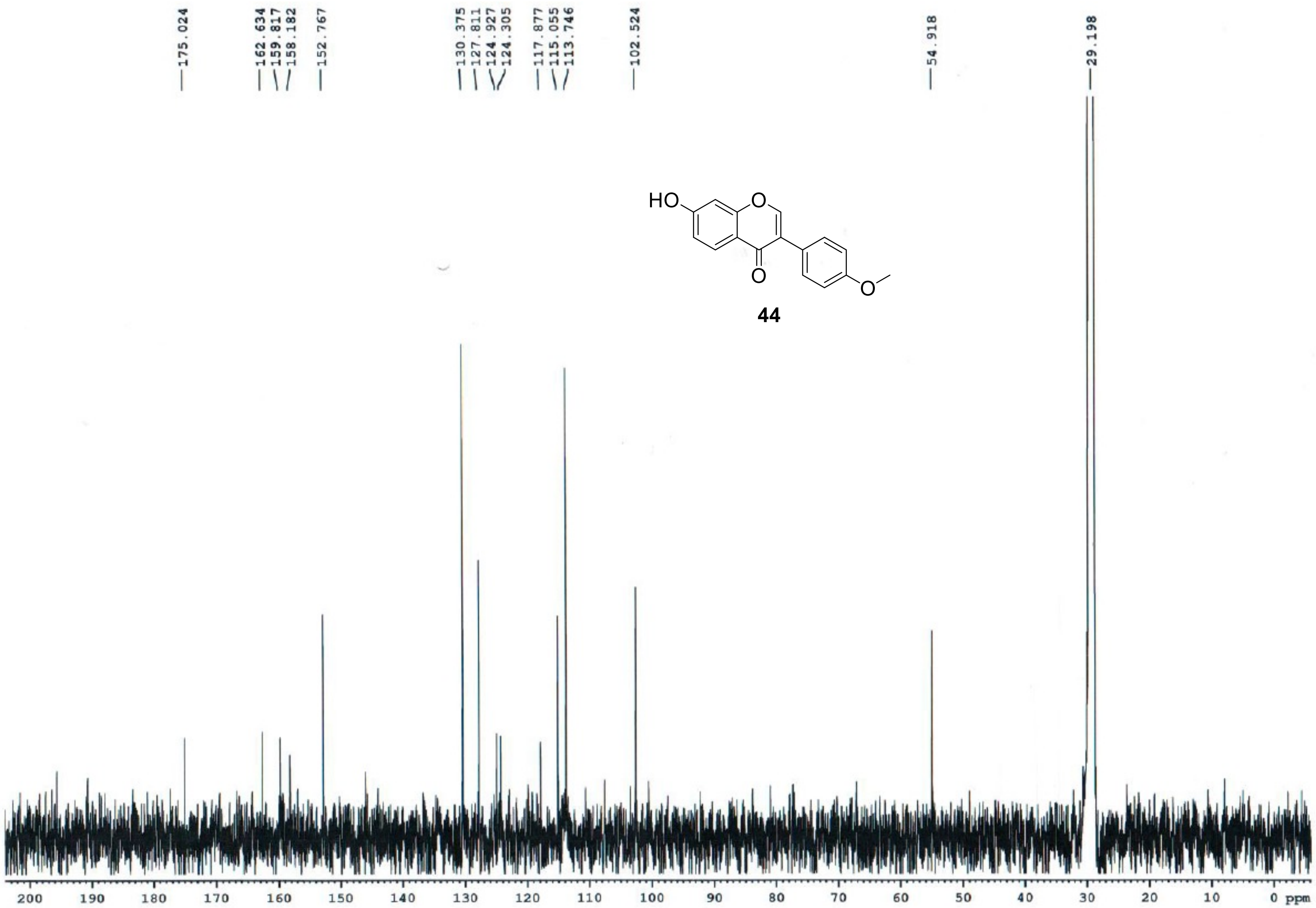
175.024
162.634
159.817
158.182
152.767
130.375
127.811
124.927
124.305
117.877
115.055
113.746
102.524
54.918
29.198
HO
O
O
O
44
200 190 180 170 160 150 140 130 120 110 100 90 80 70 60 50 40 30 20 10 0 ppm

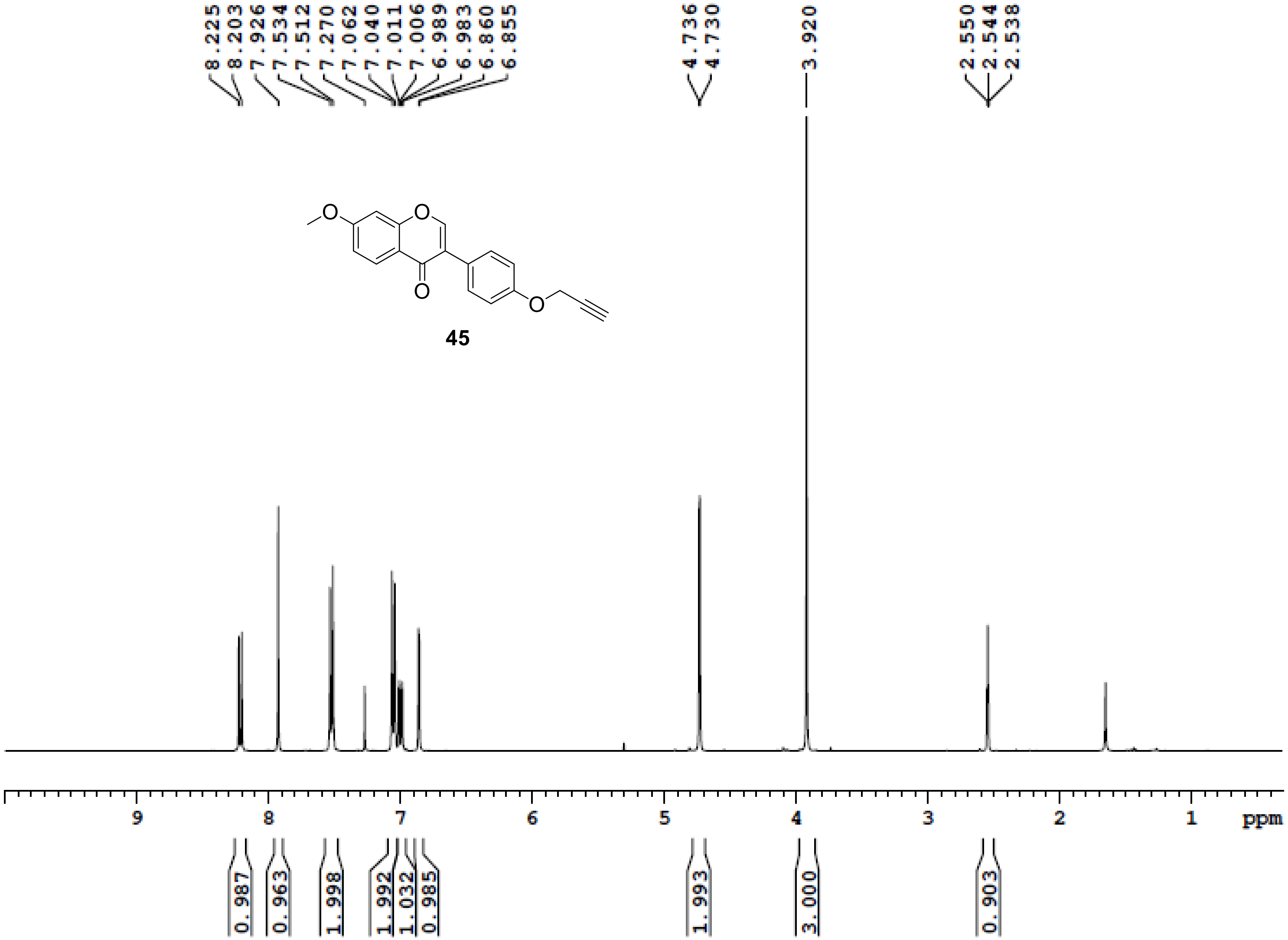
8.225
8.203
7.926
7.534
7.512
7.270
7.062
7.040
7.011
7.006
6.989
6.983
6.860
6.855
4.736
4.730
3.920
2.550
2.544
2.538
45
9
8
7
6
5
4
3
2
1
ppm
0.987
0.963
1.998
1.992
1.032
0.985
1.993
3.000
0.903

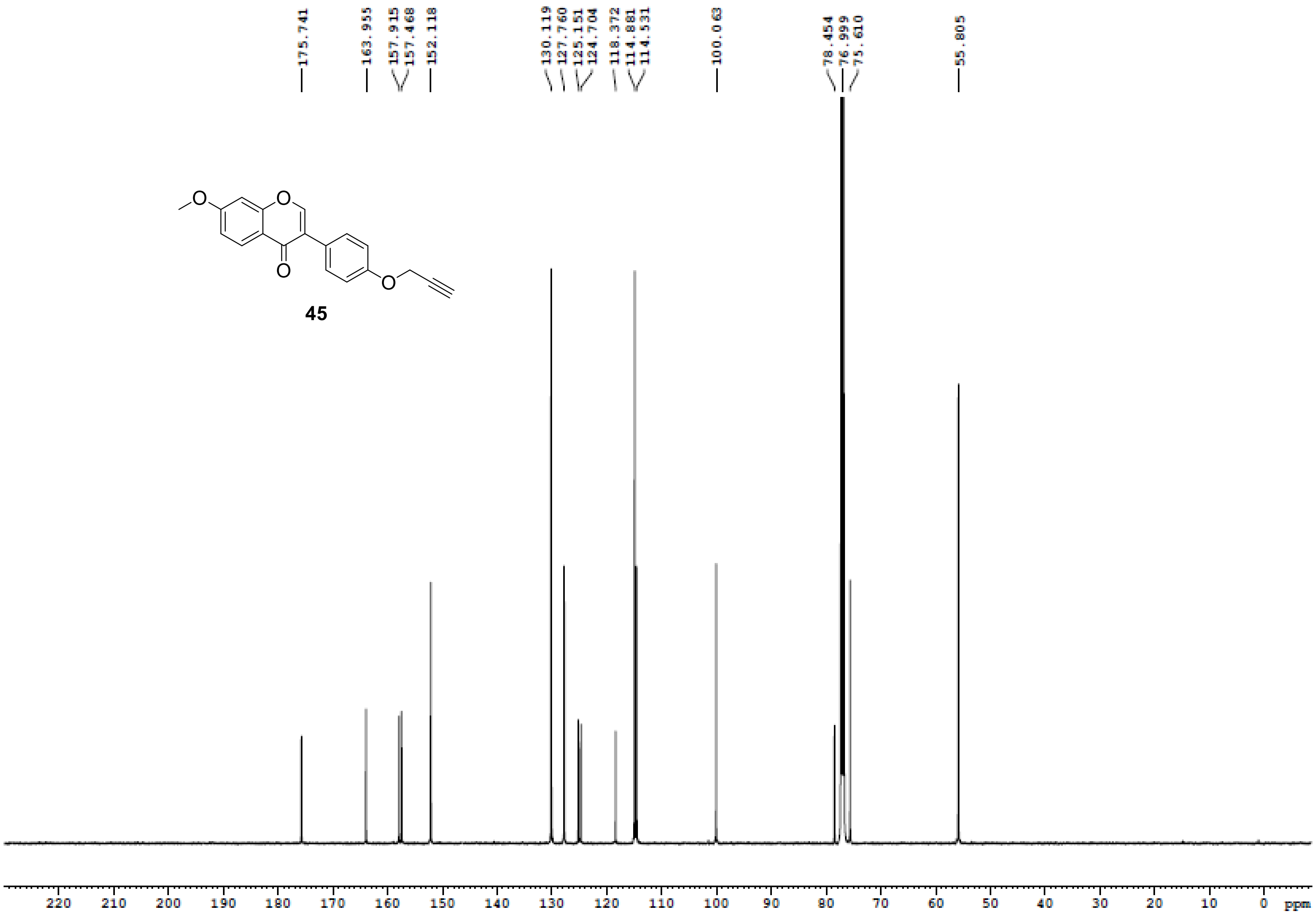
175.741
163.955
157.915
157.468
152.118
130.119
127.760
125.151
124.704
118.372
114.881
114.531
100.063
78.454
76.999
75.610
55.805
O
O
O
O
45
220 210 200 190 180 170 160 150 140 130 120 110 100 90 80 70 60 50 40 30 20 10 0 ppm

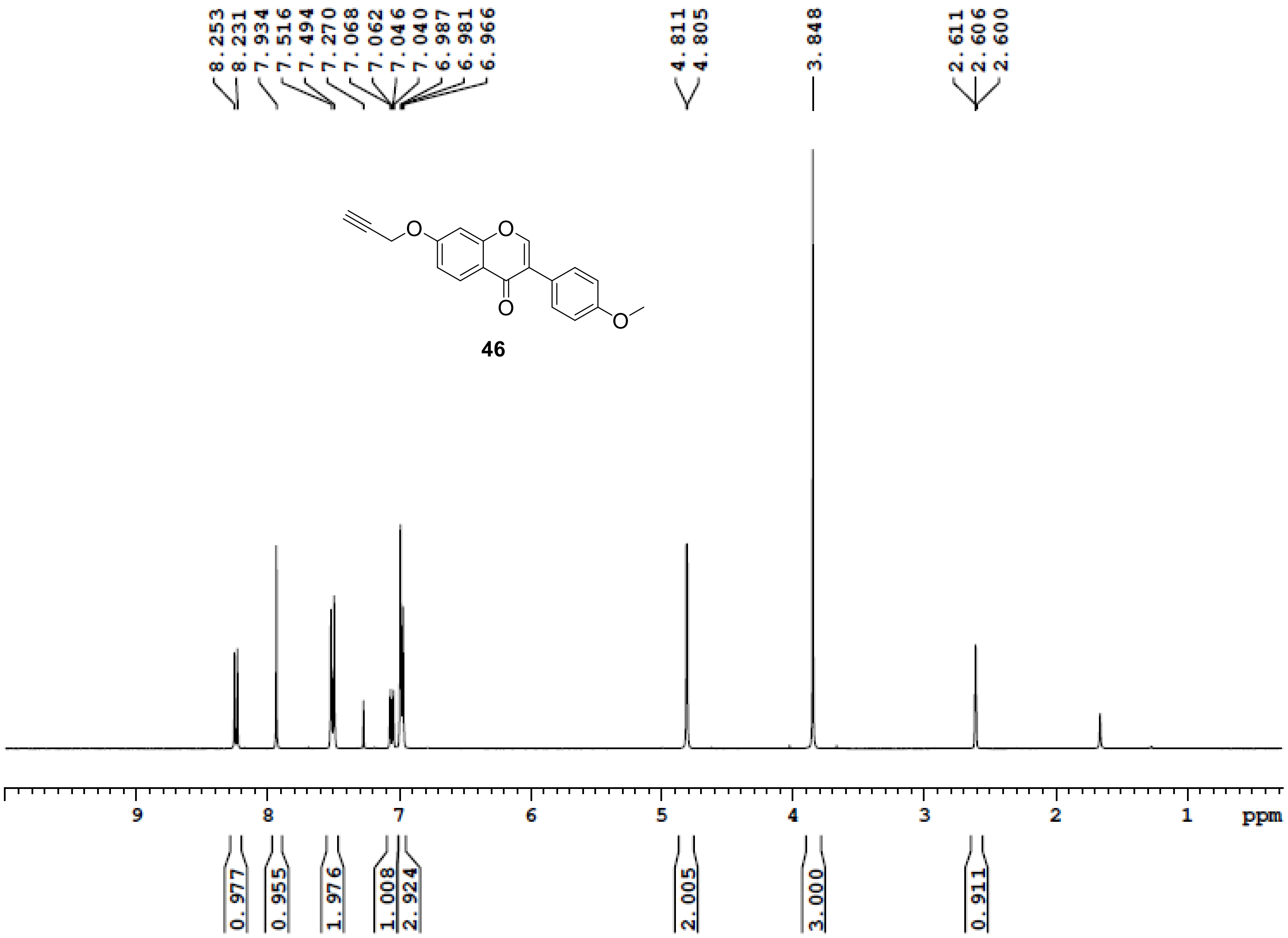
8.253
8.231
7.934
7.516
7.494
7.270
7.068
7.062
7.046
7.040
6.987
6.981
6.966
4.811
4.805
3.848
2.611
2.606
2.600
46
9
8
7
6
5
4
3
2
1
ppm
0.977
0.955
1.976
1.008
2.924
2.005
3.000
0.911

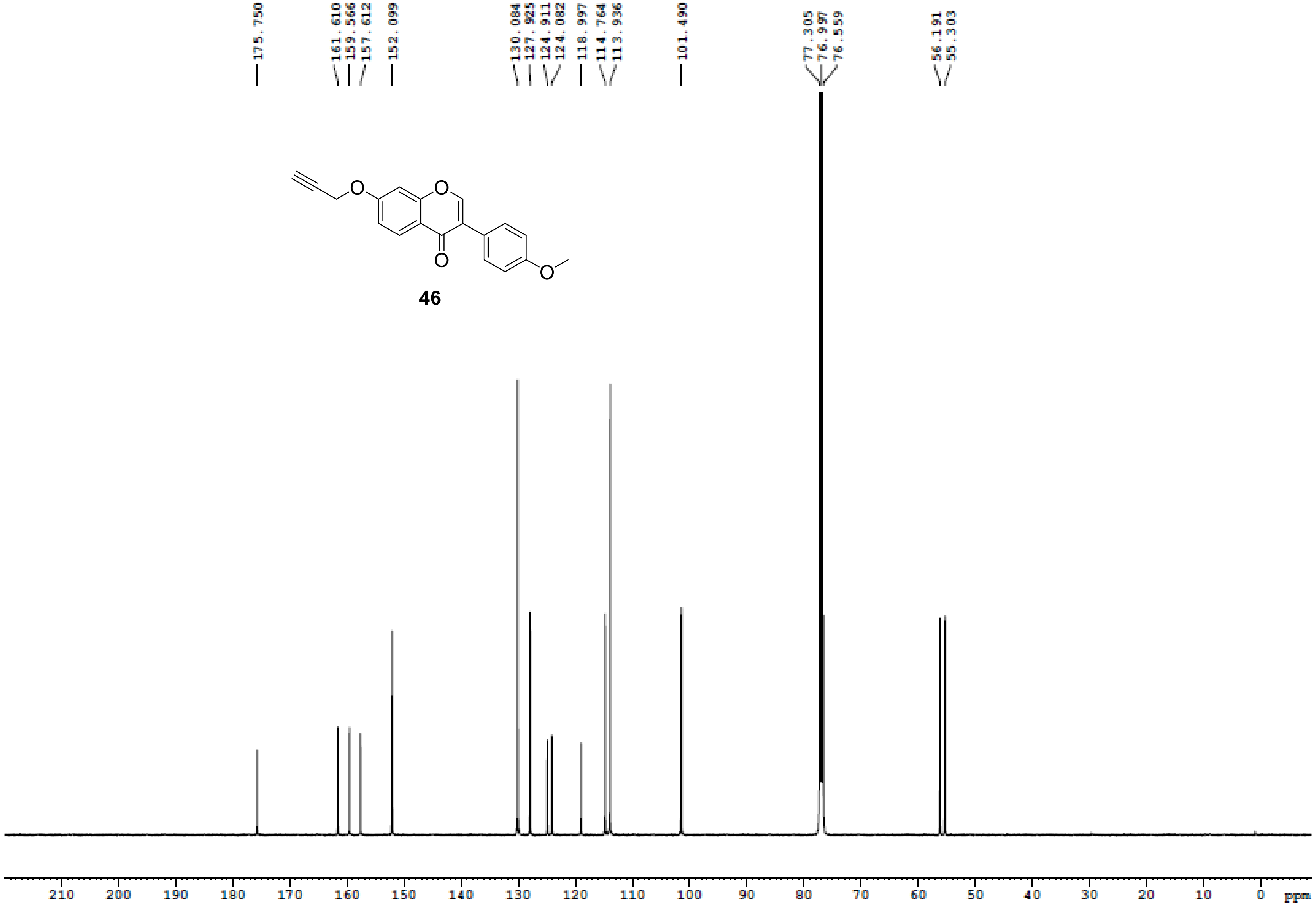
175.750
161.610
159.566
157.612
152.099
130.084
127.925
124.911
124.082
118.997
114.764
113.936
101.490
77.305
76.997
76.559
56.191
55.303
210 200 190 180 170 160 150 140 130 120 110 100 90 80 70 60 50 40 30 20 10 0 ppm

46

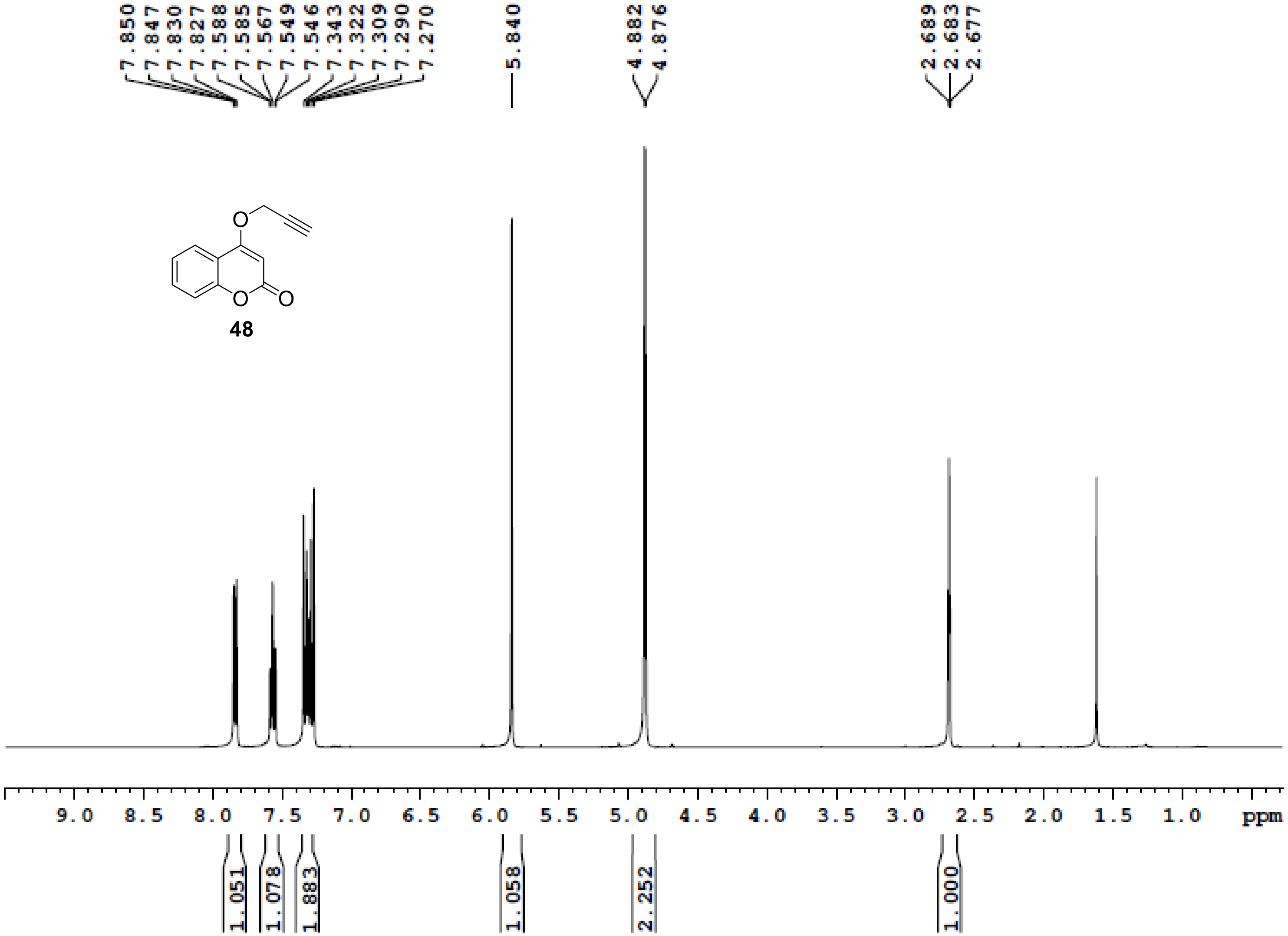
7.850
7.847
7.830
7.827
7.588
7.585
7.567
7.549
7.546
7.343
7.322
7.309
7.290
7.270
5.840
4.882
4.876
2.689
2.683
2.677
O
O
O
48
9.0 8.5 8.0 7.5 7.0 6.5 6.0 5.5 5.0 4.5 4.0 3.5 3.0 2.5 2.0 1.5 1.0 ppm
1.051
1.078
1.883
1.058
2.252
1.000

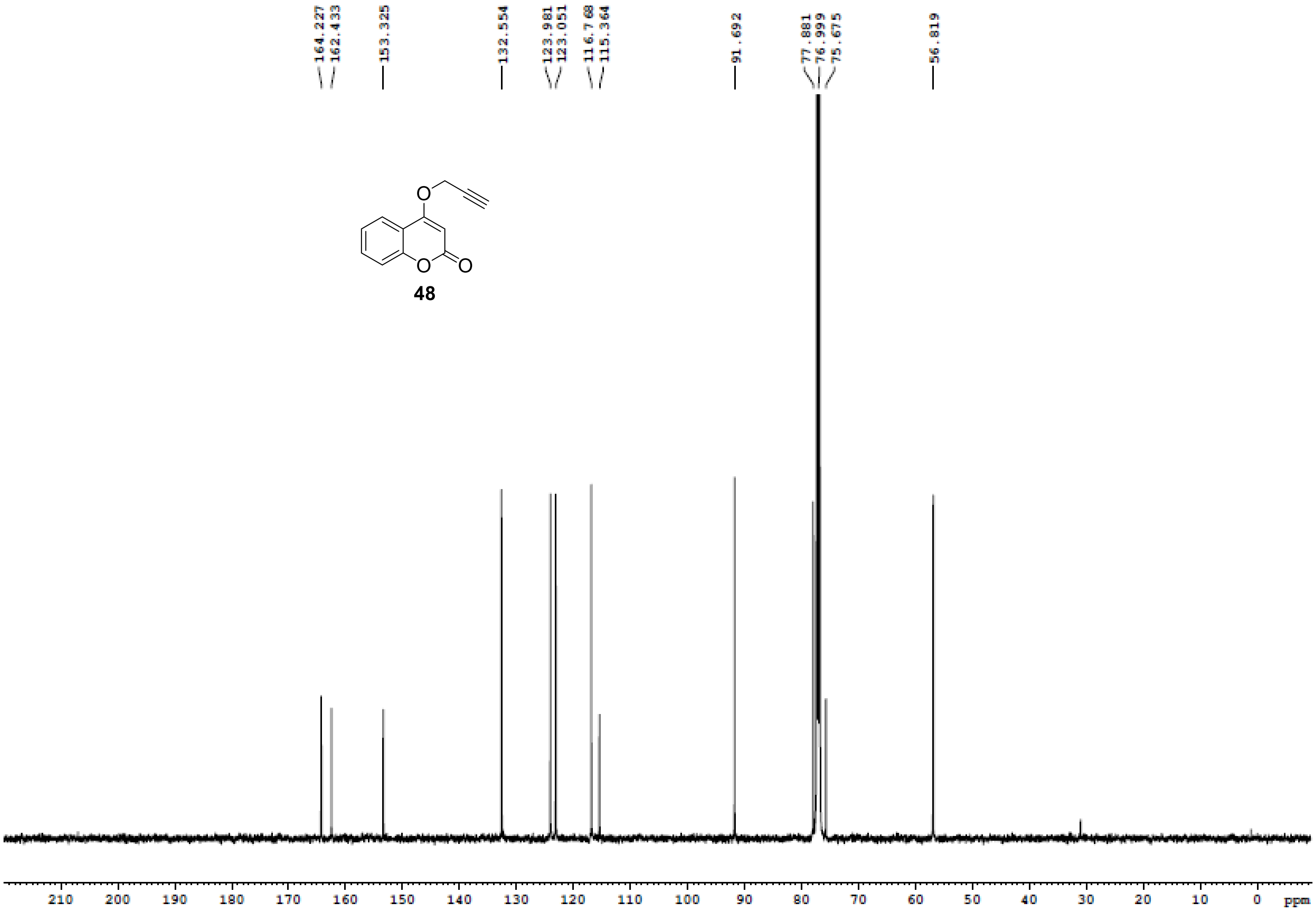
164.227
162.433
153.325
132.554
123.981
123.051
116.768
115.364
91.692
77.881
76.999
75.675
56.819
48
210 200 190 180 170 160 150 140 130 120 110 100 90 80 70 60 50 40 30 20 10 0 ppm

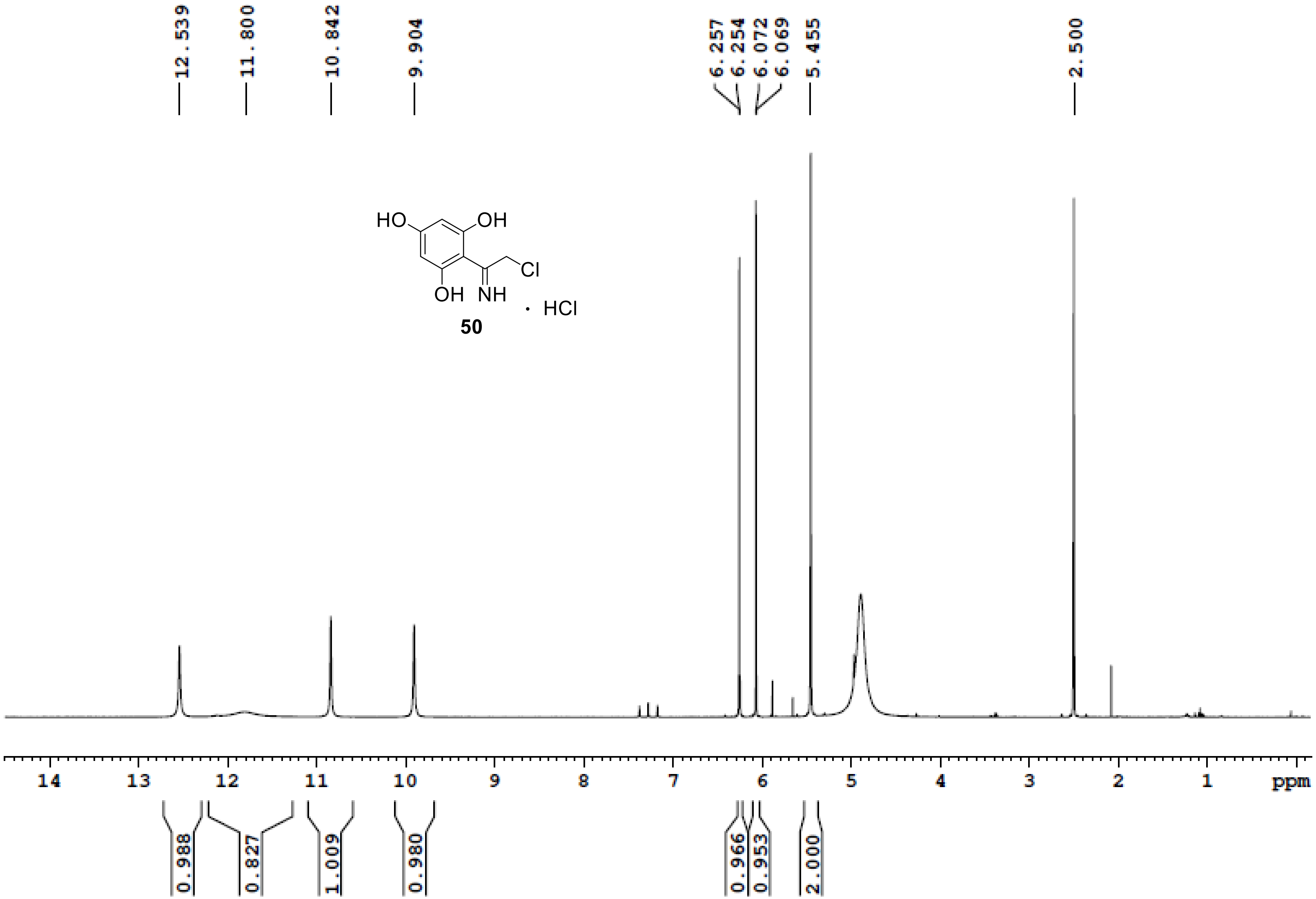
12.539
11.800
10.842
9.904
6.257
6.254
6.072
6.069
5.455
2.500
HO
OH
Cl
OH
NH
· HCl
50
14
13
12
11
10
9
8
7
6
5
4
3
2
1
ppm
0.988
0.827
1.009
0.980
0.966
0.953
2.000

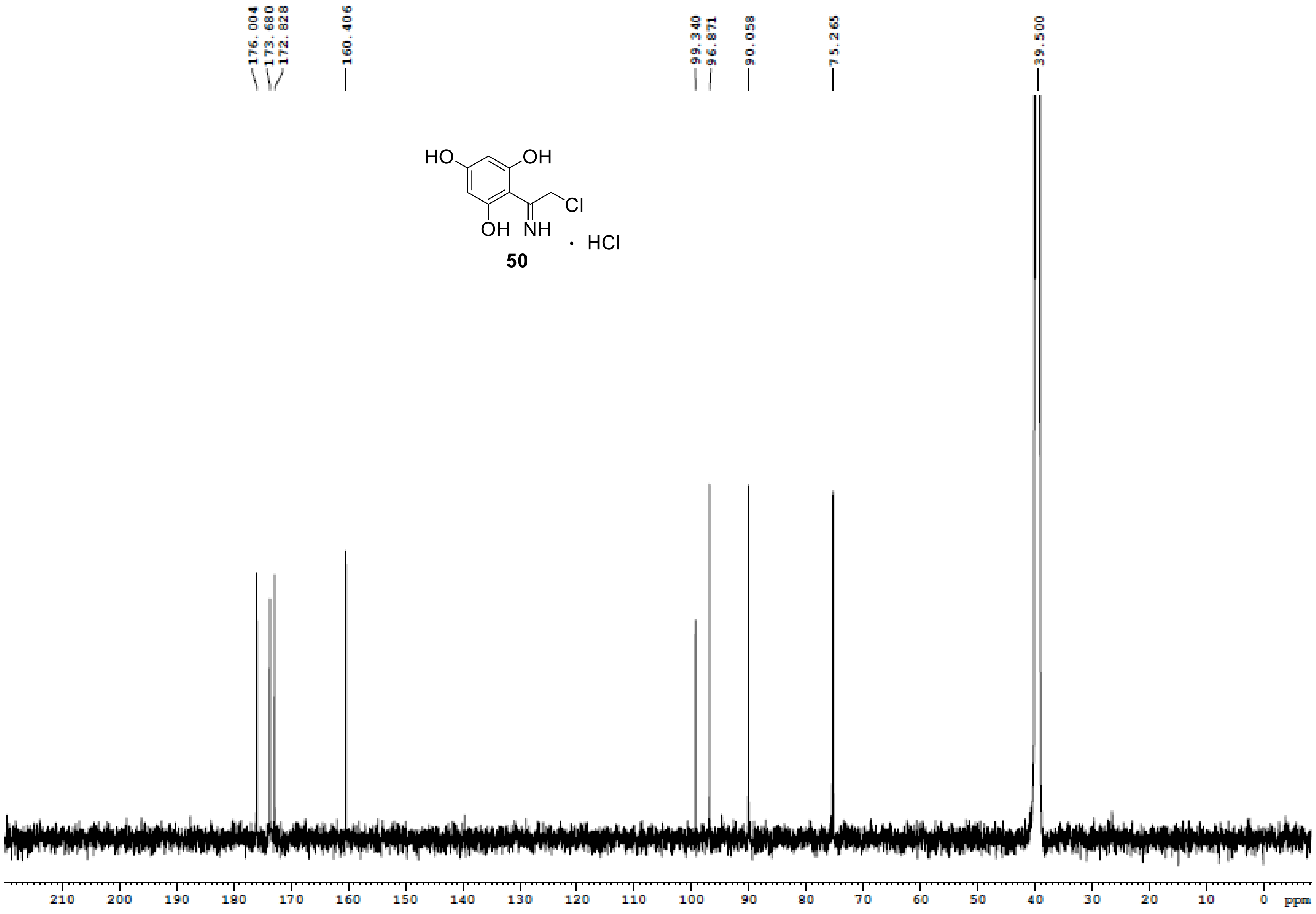
176.004
173.680
172.828
160.406
99.340
96.871
90.058
75.265
39.500
HO
OH
Cl
OH
NH
· HCl
50
210 200 190 180 170 160 150 140 130 120 110 100 90 80 70 60 50 40 30 20 10 0 ppm

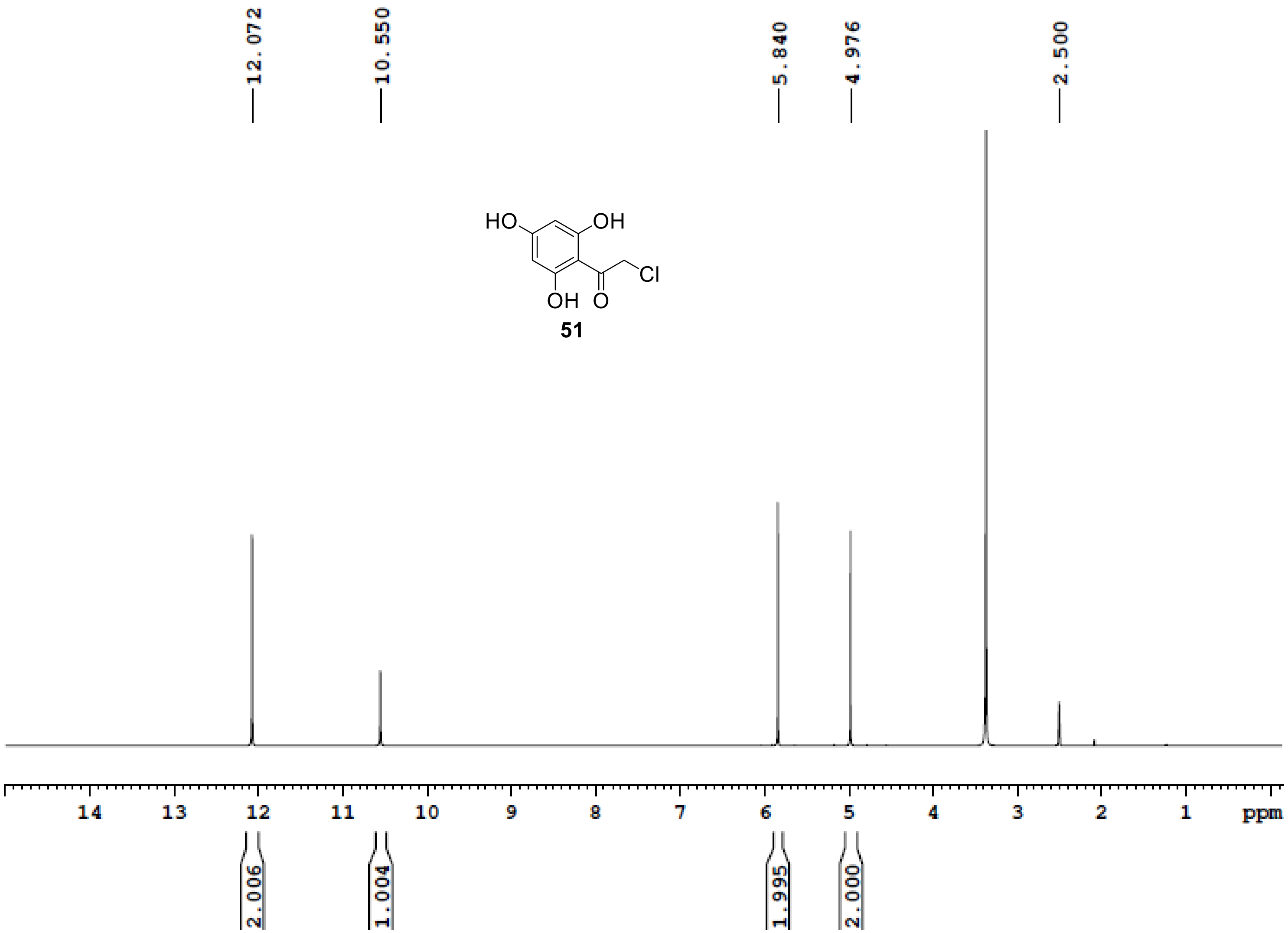
12.072
10.550
5.840
4.976
2.500
HO
OH
Cl
OH
O
51
14
13
12
11
10
9
8
7
6
5
4
3
2
1
ppm
2.006
1.004
1.995
2.000

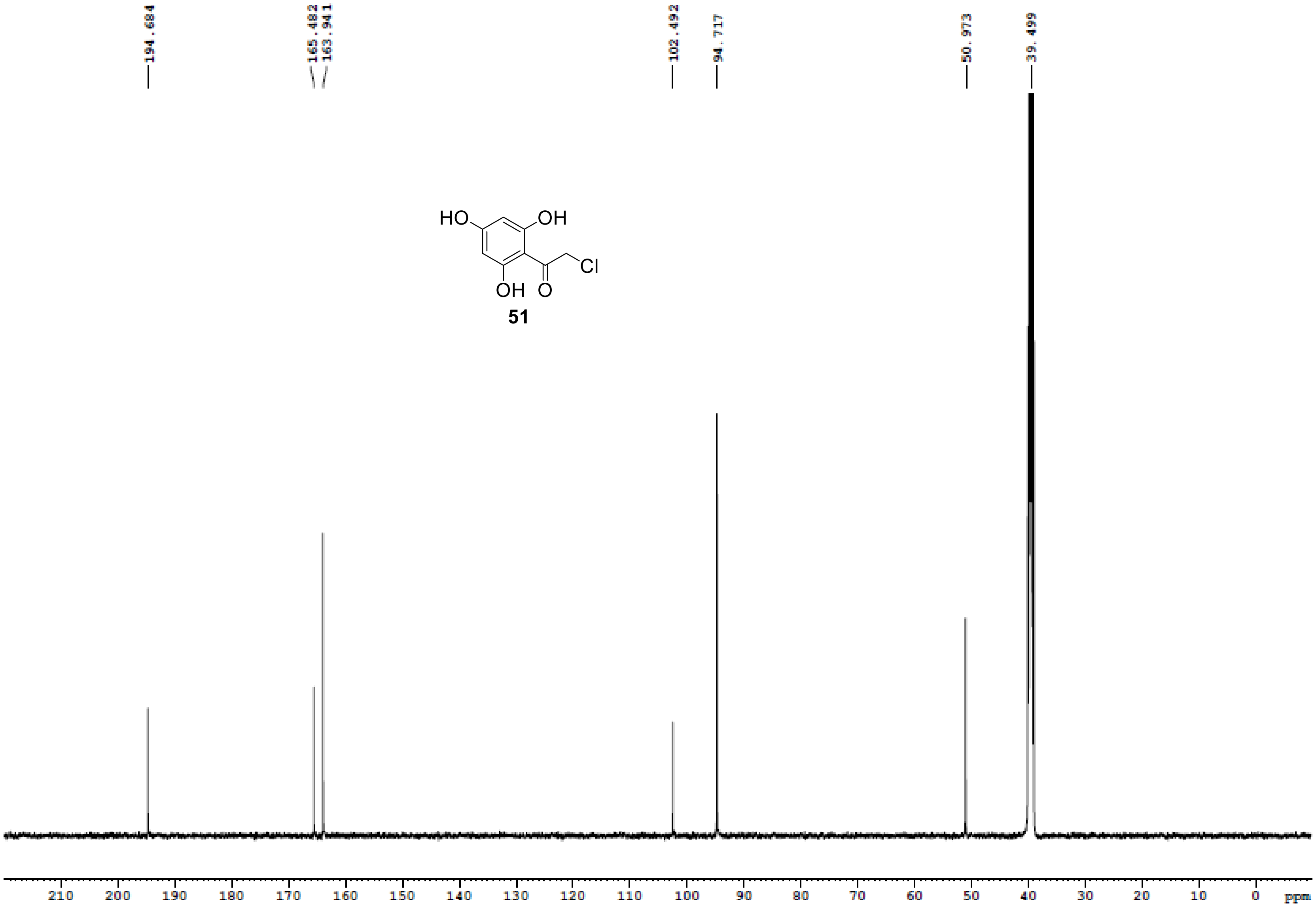
194.684
165.482
163.941
102.492
94.717
50.973
39.499
HO
OH
Cl
OH
O
51
210 200 190 180 170 160 150 140 130 120 110 100 90 80 70 60 50 40 30 20 10 0 ppm

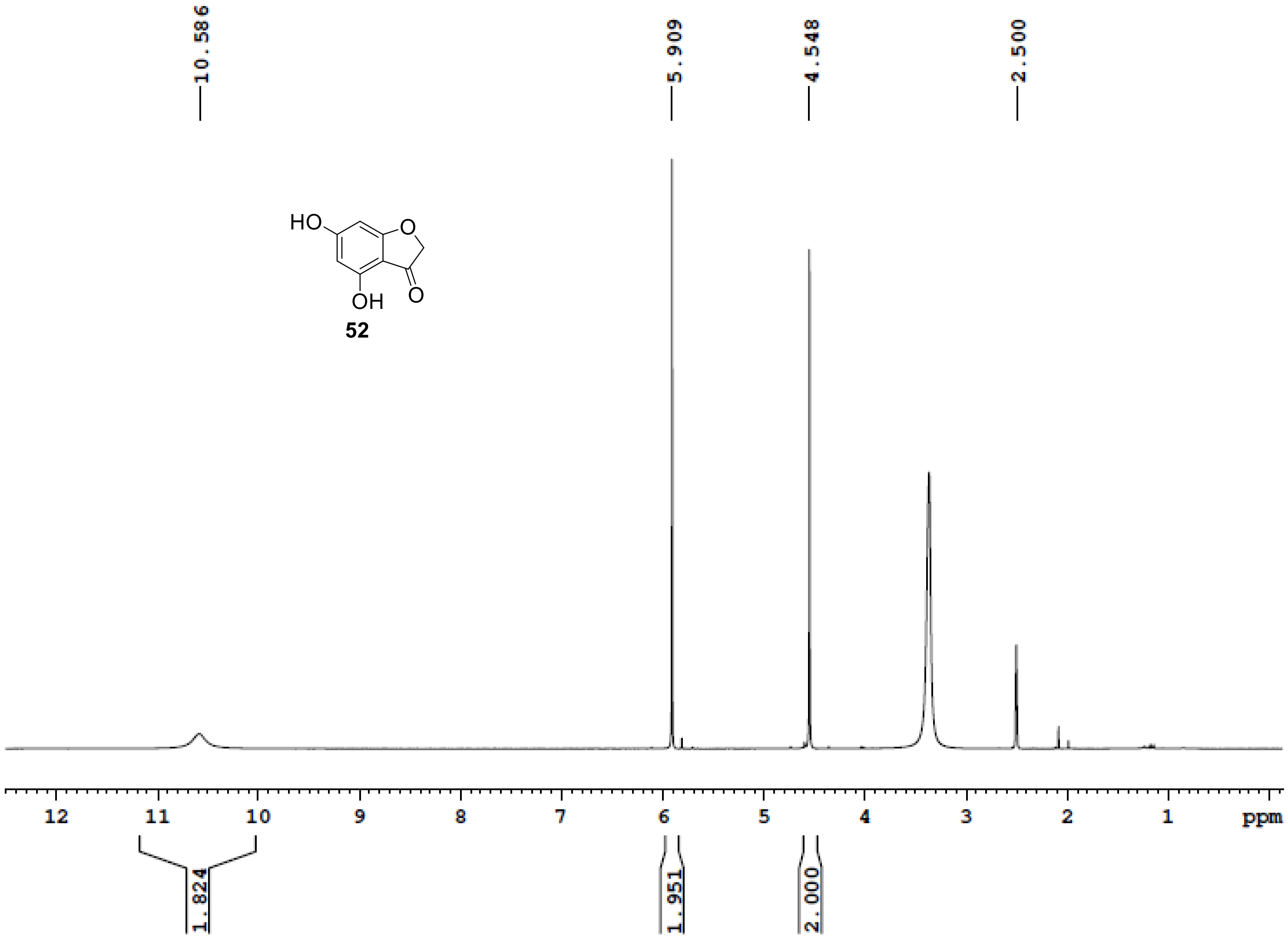
10.586
5.909
4.548
2.500
HO
O
OH
O
52
12
11
10
9
8
7
6
5
4
3
2
1
ppm
1.824
1.951
2.000

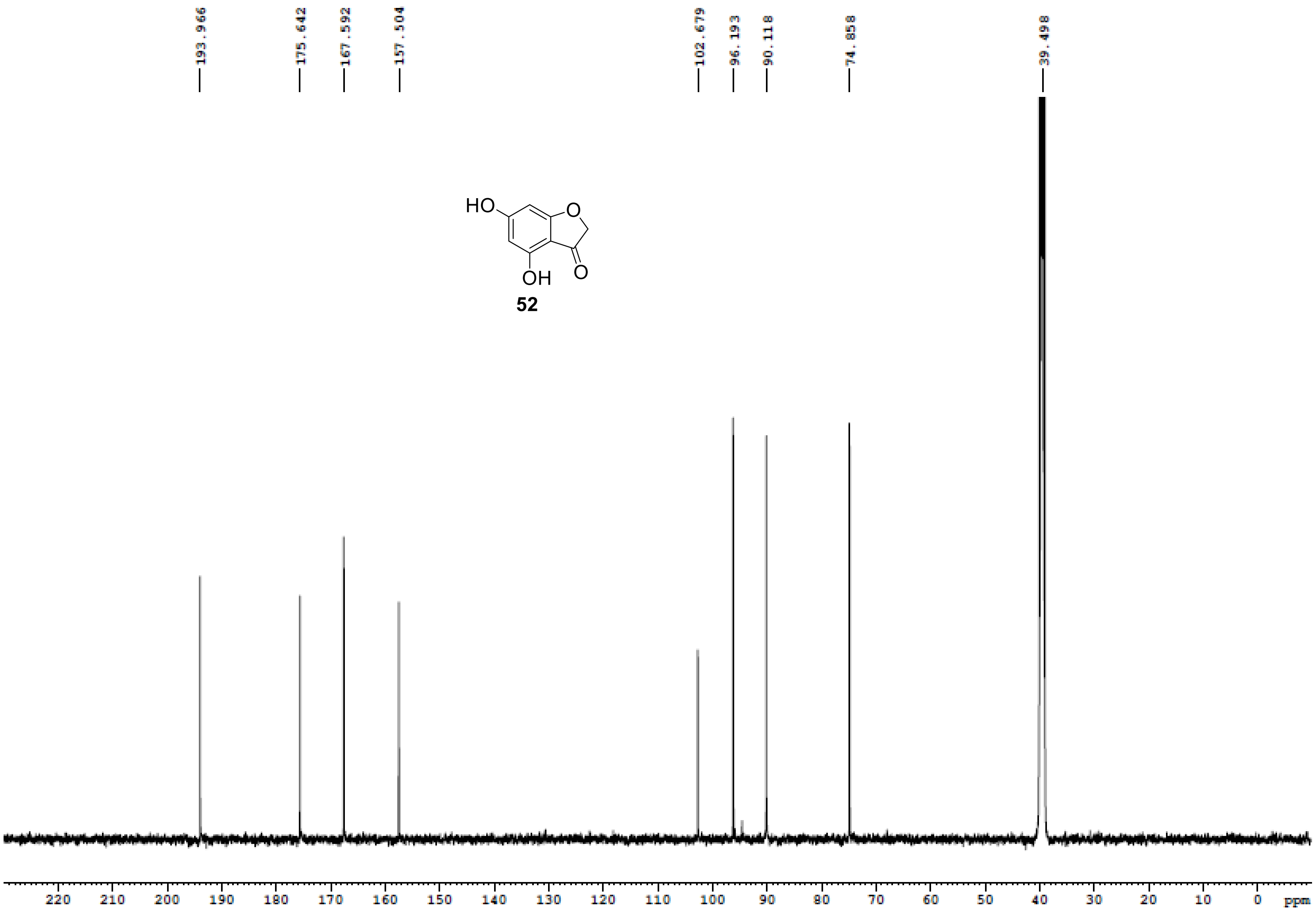
193.966
175.642
167.592
157.504
102.679
96.193
90.118
74.858
39.498
HO
O
OH
O
52
220 210 200 190 180 170 160 150 140 130 120 110 100 90 80 70 60 50 40 30 20 10 0 ppm

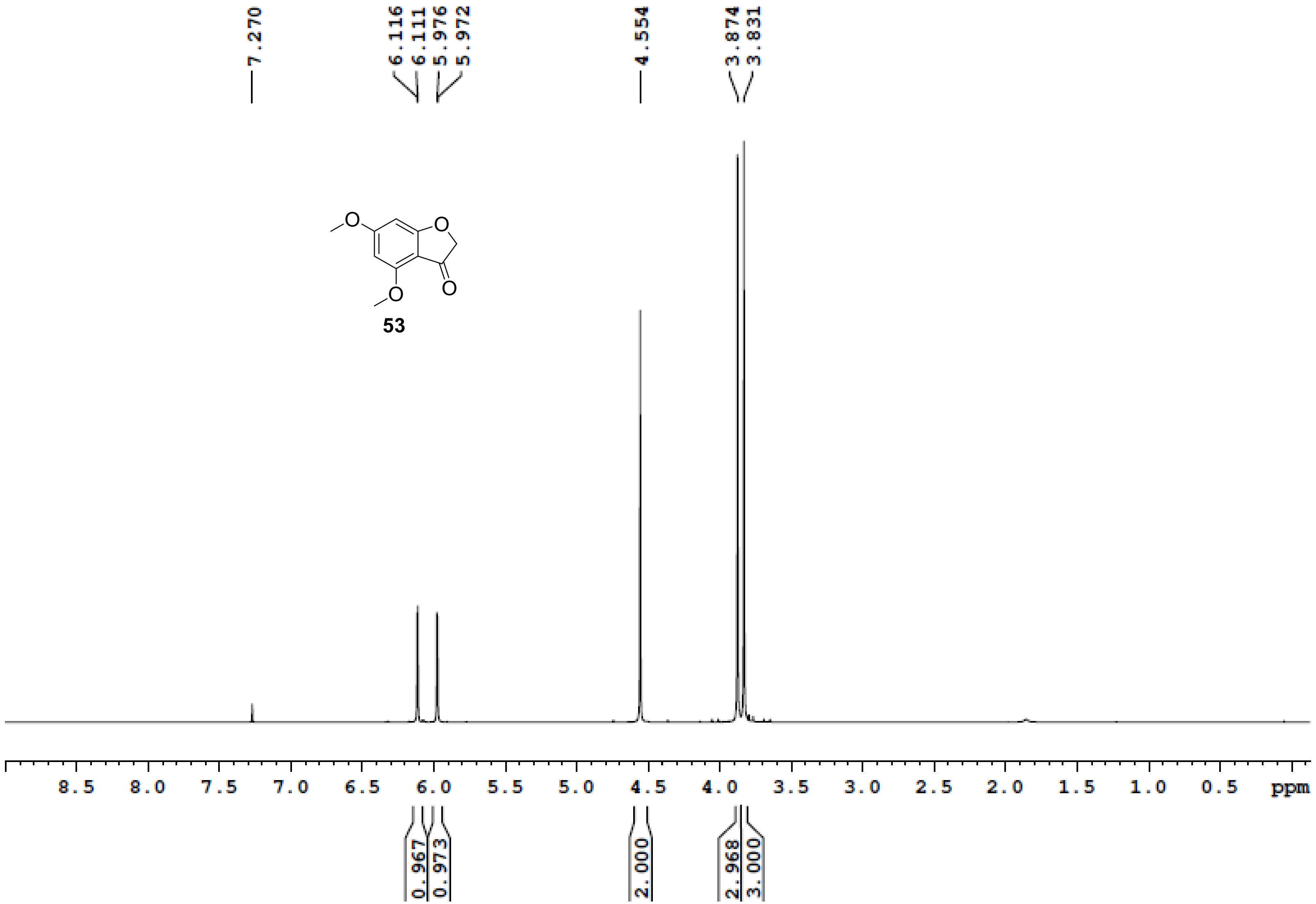
7.270
6.116
6.111
5.976
5.972
4.554
3.874
3.831
53
8.5 8.0 7.5 7.0 6.5 6.0 5.5 5.0 4.5 4.0 3.5 3.0 2.5 2.0 1.5 1.0 0.5 ppm
0.967
0.973
2.000
2.968
3.000

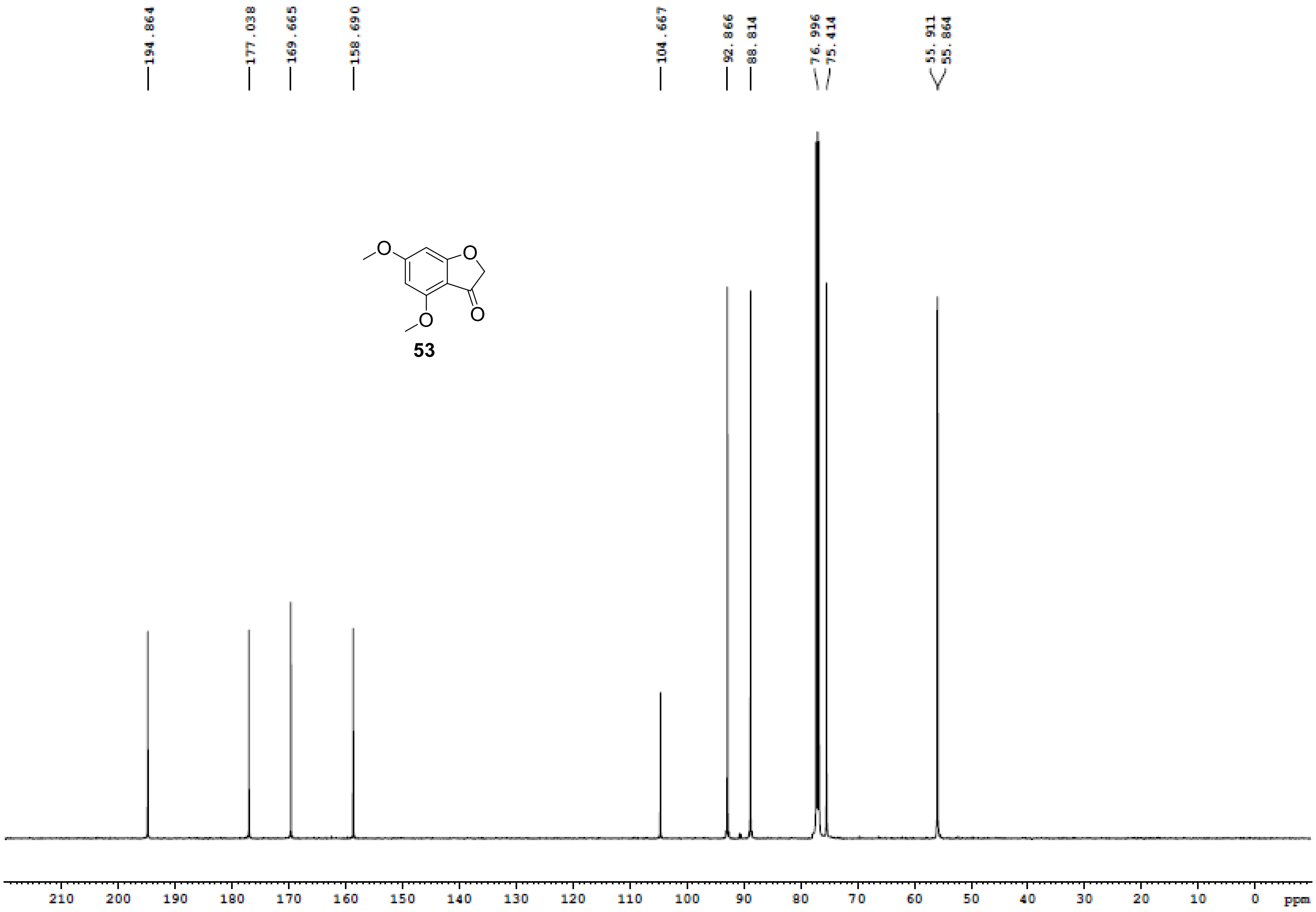
194.864
177.038
169.665
158.690
104.667
92.866
88.814
76.996
75.414
55.911
55.864
O
O
O
O
53
210 200 190 180 170 160 150 140 130 120 110 100 90 80 70 60 50 40 30 20 10 0 ppm

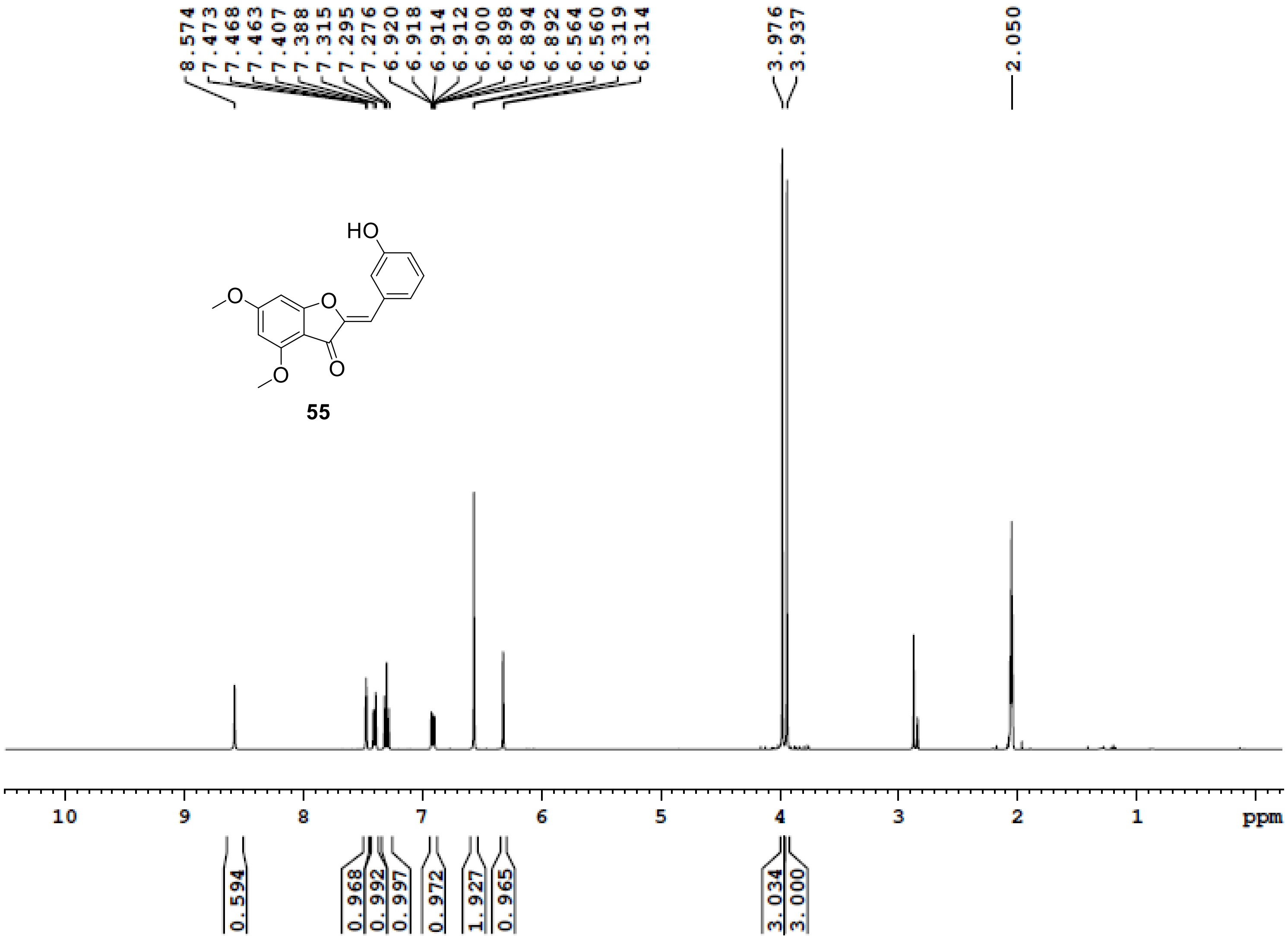
8.574
7.473
7.468
7.463
7.407
7.388
7.315
7.295
7.276
6.920
6.918
6.914
6.912
6.900
6.898
6.894
6.892
6.564
6.560
6.319
6.314
3.976
3.937
2.050
HO
O
O
O
O
55
10
9
8
7
6
5
4
3
2
1
ppm
0.594
0.968
0.992
0.997
0.972
1.927
0.965
3.034
3.000

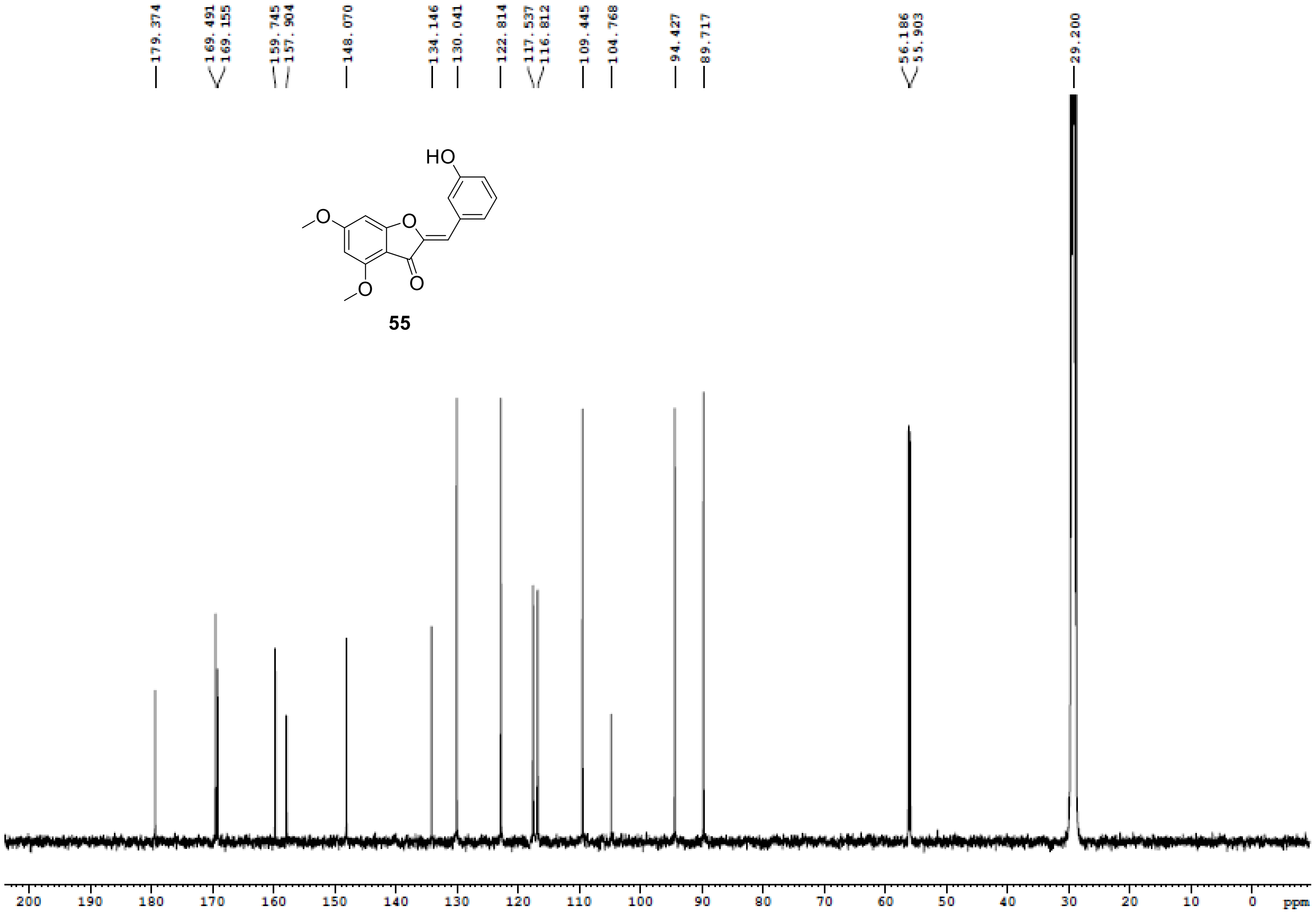
179.374
169.491
169.155
159.745
157.904
148.070
134.146
130.041
122.814
117.537
116.812
109.445
104.768
94.427
89.717
56.186
55.903
29.200
HO
O
O
O
O
55
200 190 180 170 160 150 140 130 120 110 100 90 80 70 60 50 40 30 20 10 0 ppm

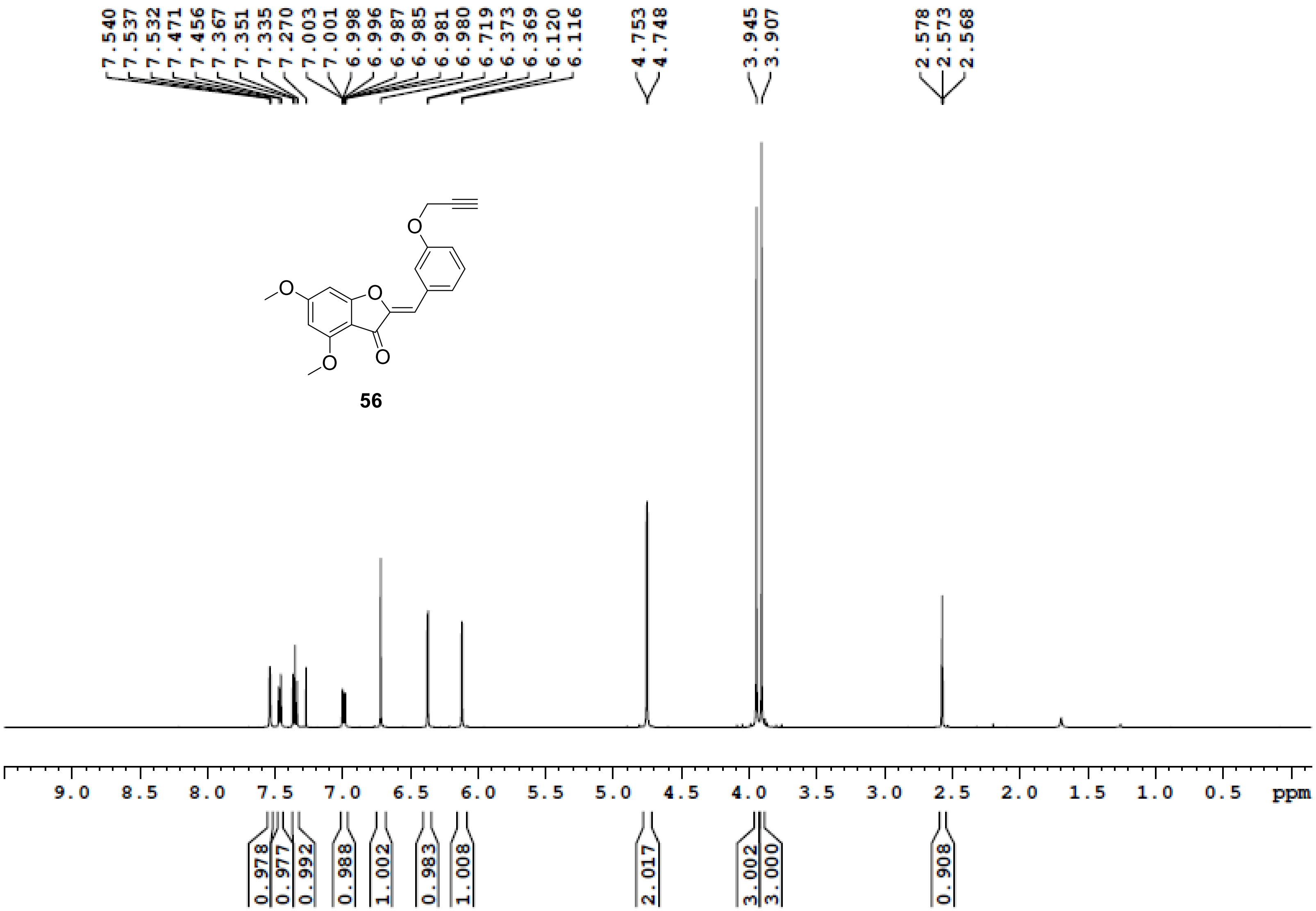
7.540
7.537
7.532
7.471
7.456
7.367
7.351
7.335
7.270
7.003
7.001
6.998
6.996
6.987
6.985
6.981
6.980
6.719
6.373
6.369
6.120
6.116
4.753
4.748
3.945
3.907
2.578
2.573
2.568
O
O
O
O
O
56
9.0
8.5
8.0
7.5
7.0
6.5
6.0
5.5
5.0
4.5
4.0
3.5
3.0
2.5
2.0
1.5
1.0
0.5
ppm
0.978
0.977
0.992
0.988
1.002
0.983
1.008
2.017
3.002
3.000
0.908

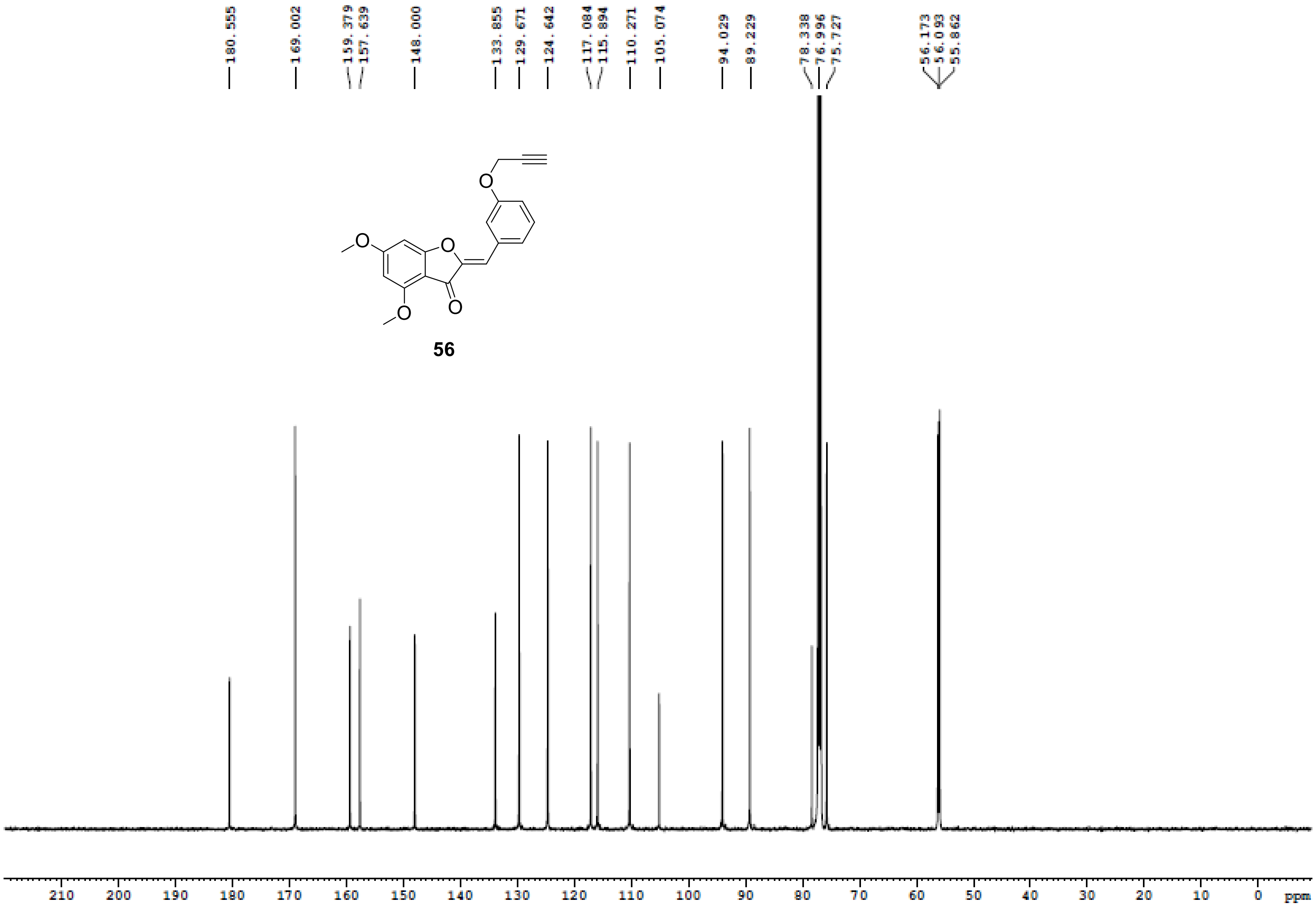
180.555
169.002
159.379
157.639
148.000
133.855
129.671
124.642
117.084
115.894
110.271
105.074
94.029
89.229
78.338
76.996
75.727
56.173
56.093
55.862
56
210 200 190 180 170 160 150 140 130 120 110 100 90 80 70 60 50 40 30 20 10 0 ppm

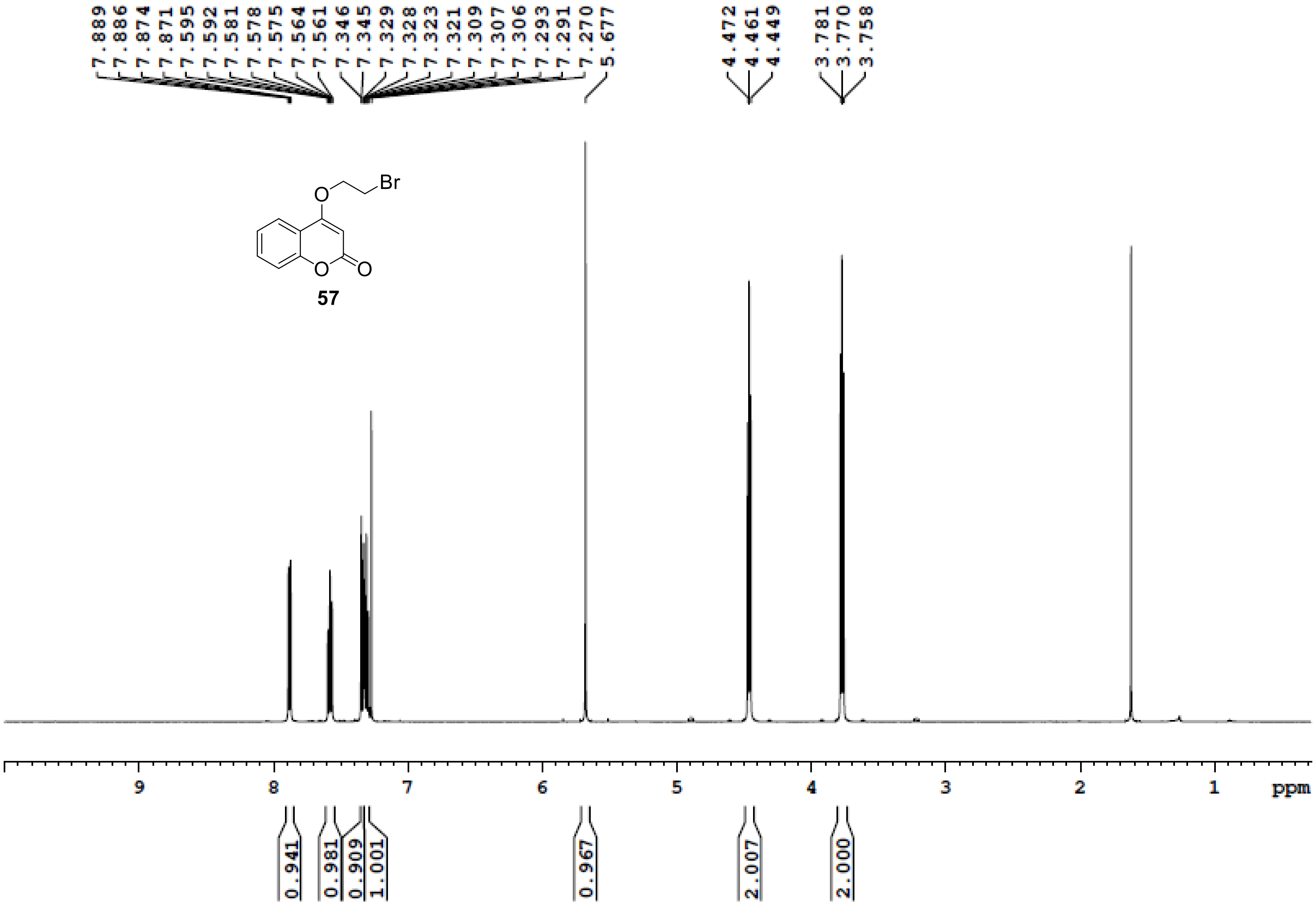
7.889
7.886
7.874
7.871
7.595
7.592
7.581
7.578
7.575
7.564
7.561
7.346
7.345
7.329
7.328
7.323
7.321
7.309
7.307
7.306
7.293
7.291
7.270
5.677
4.472
4.461
4.449
3.781
3.770
3.758
O
Br
O
O
57
9
8
7
6
5
4
3
2
1
ppm
0.941
0.981
0.909
1.001
0.967
2.007
2.000

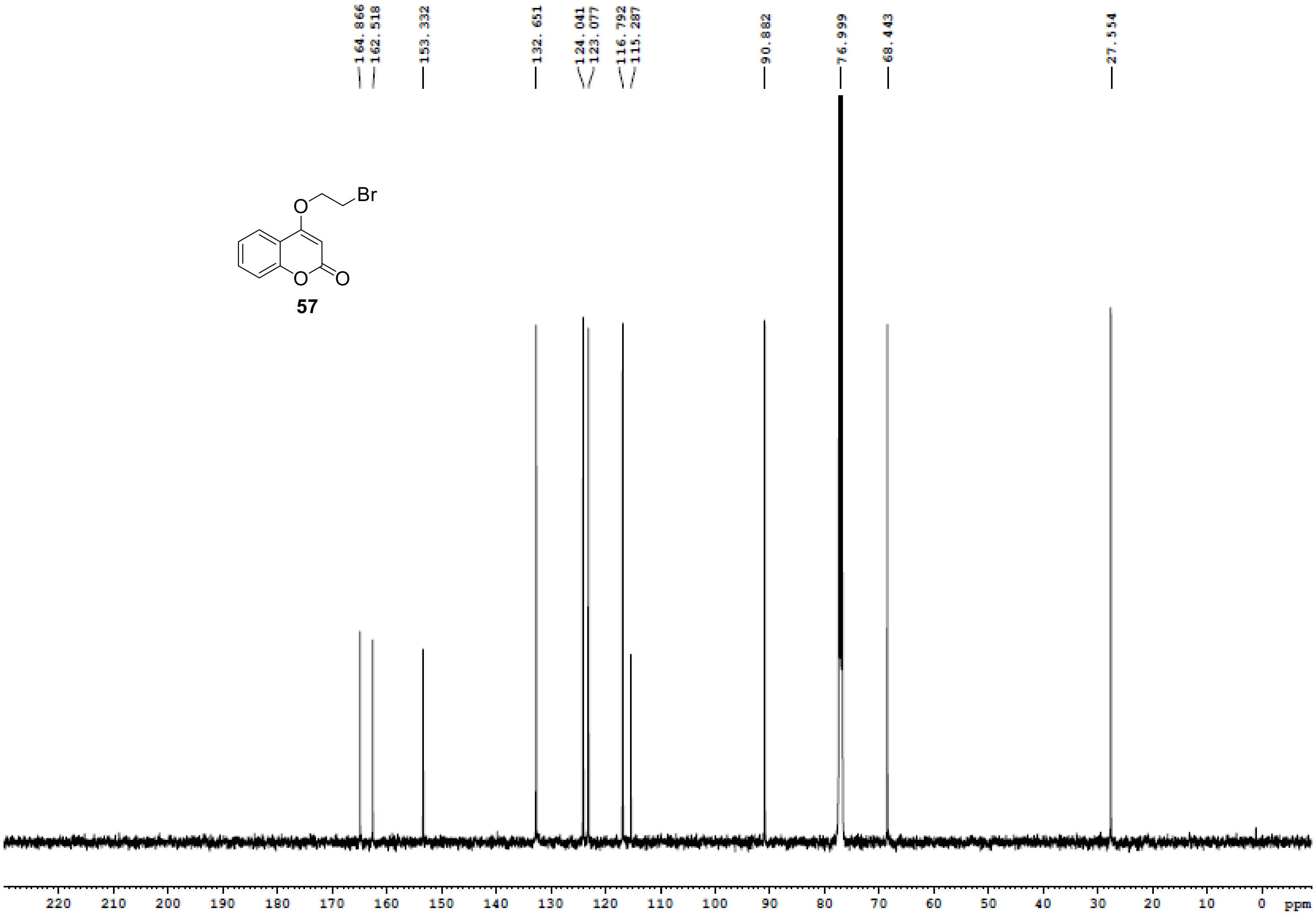
164.866
162.518
153.332
132.651
124.041
123.077
116.792
115.287
90.882
76.999
68.443
27.554
O
Br
O
O
57
220 210 200 190 180 170 160 150 140 130 120 110 100 90 80 70 60 50 40 30 20 10 0 ppm

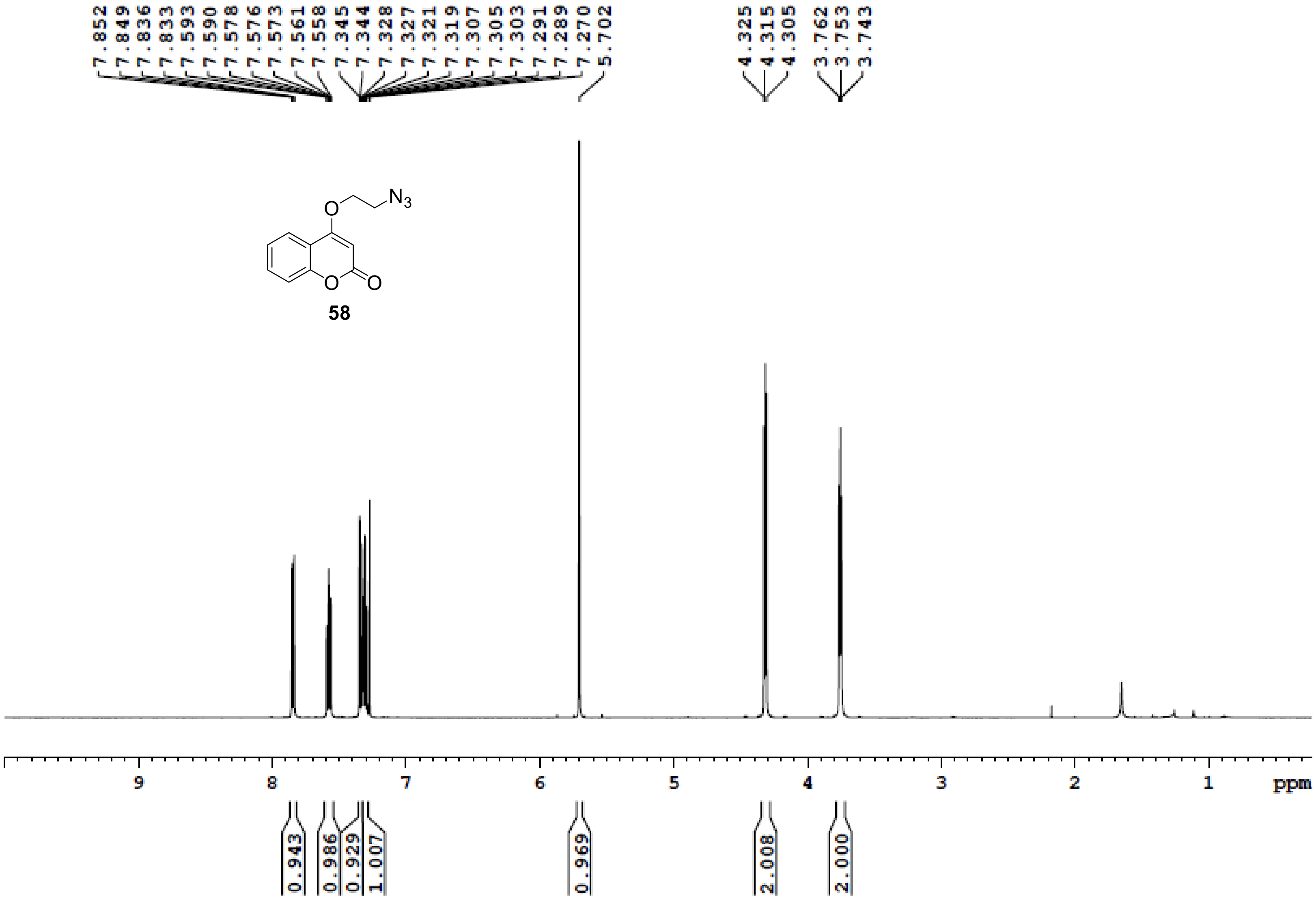
7.852
7.849
7.836
7.833
7.593
7.590
7.578
7.576
7.573
7.561
7.558
7.345
7.344
7.328
7.327
7.321
7.319
7.307
7.305
7.303
7.291
7.289
7.270
5.702
4.325
4.315
4.305
3.762
3.753
3.743
N3
O
O
O
58
9
8
7
6
5
4
3
2
1
ppm
0.943
0.986
0.929
1.007
0.969
2.008
2.000

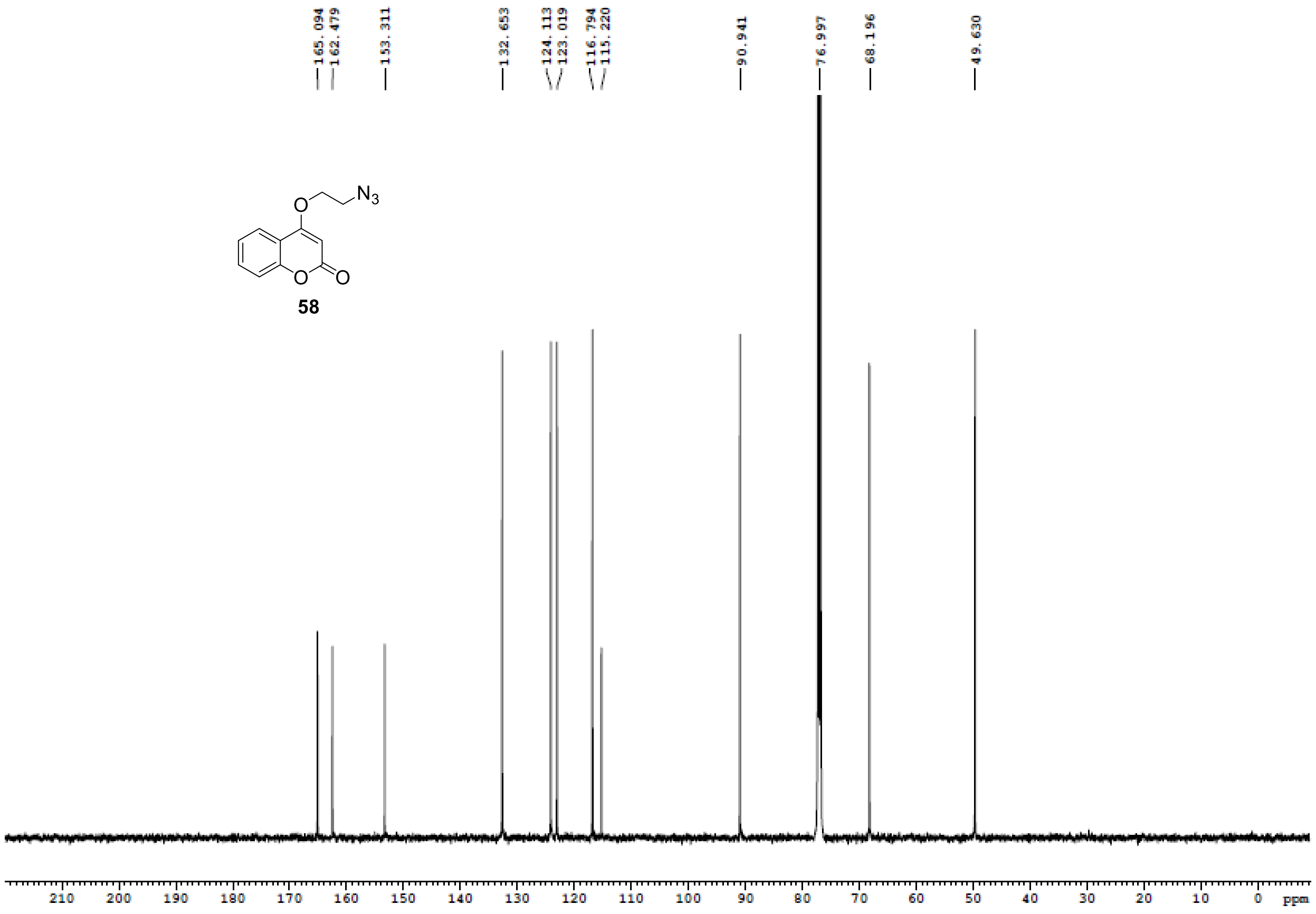

165.094
162.479
153.311
132.653
124.113
123.019
116.794
115.220
90.941
76.997
68.196
49.630
N3
58
210 200 190 180 170 160 150 140 130 120 110 100 90 80 70 60 50 40 30 20 10 0 ppm

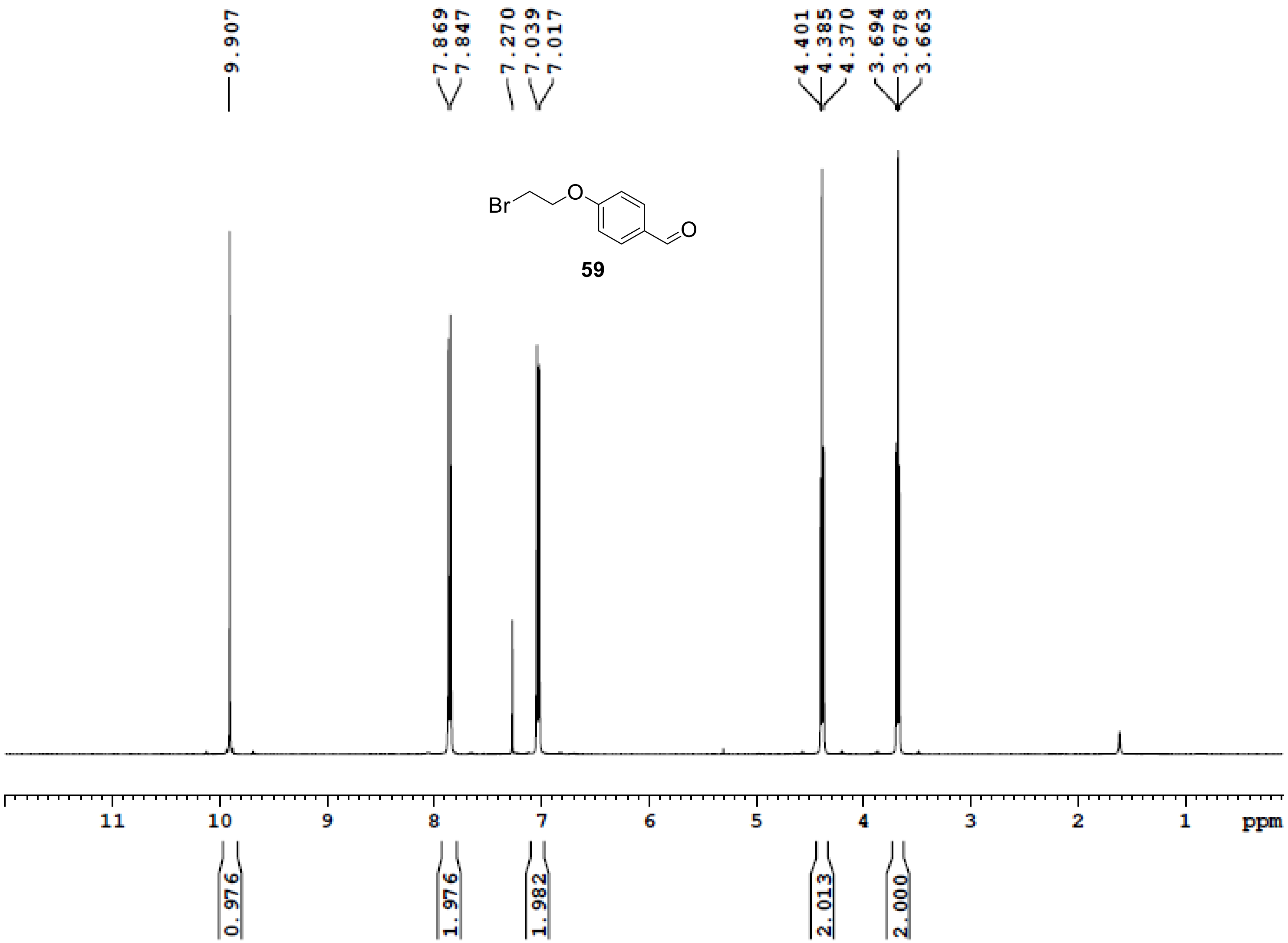
9.907
7.869
7.847
7.270
7.039
7.017
4.401
4.385
4.370
3.694
3.678
3.663
Br
O
O
59
11
10
9
8
7
6
5
4
3
2
1
ppm
0.976
1.976
1.982
2.013
2.000

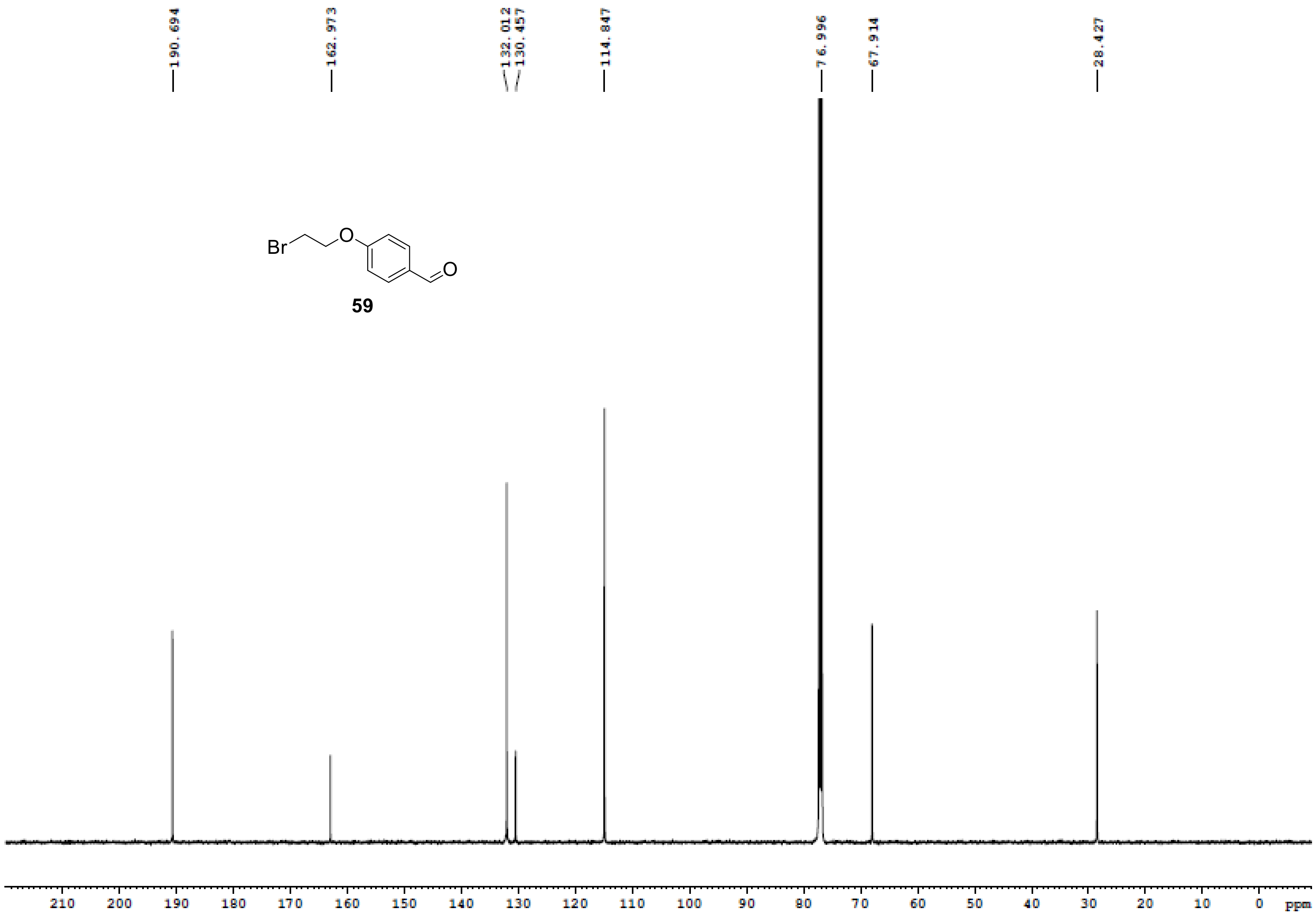

190.694
162.973
132.012
130.457
114.847
76.996
67.914
28.427
Br
O
O
59
210 200 190 180 170 160 150 140 130 120 110 100 90 80 70 60 50 40 30 20 10 0 ppm

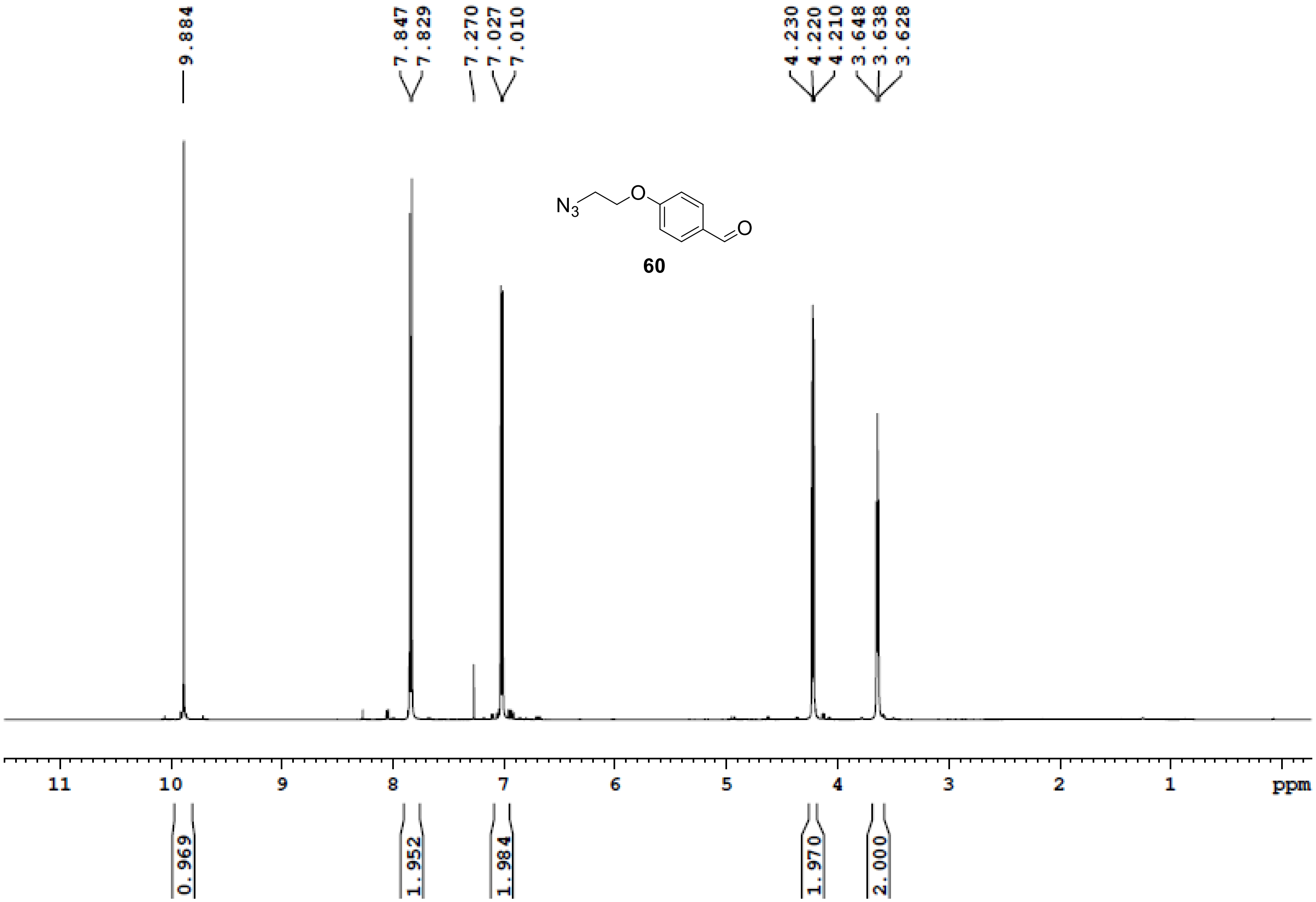
9.884
7.847
7.829
7.270
7.027
7.010
4.230
4.220
4.210
3.648
3.638
3.628
N3
O
O
60
11
10
9
8
7
6
5
4
3
2
1
ppm
0.969
1.952
1.984
1.970
2.000

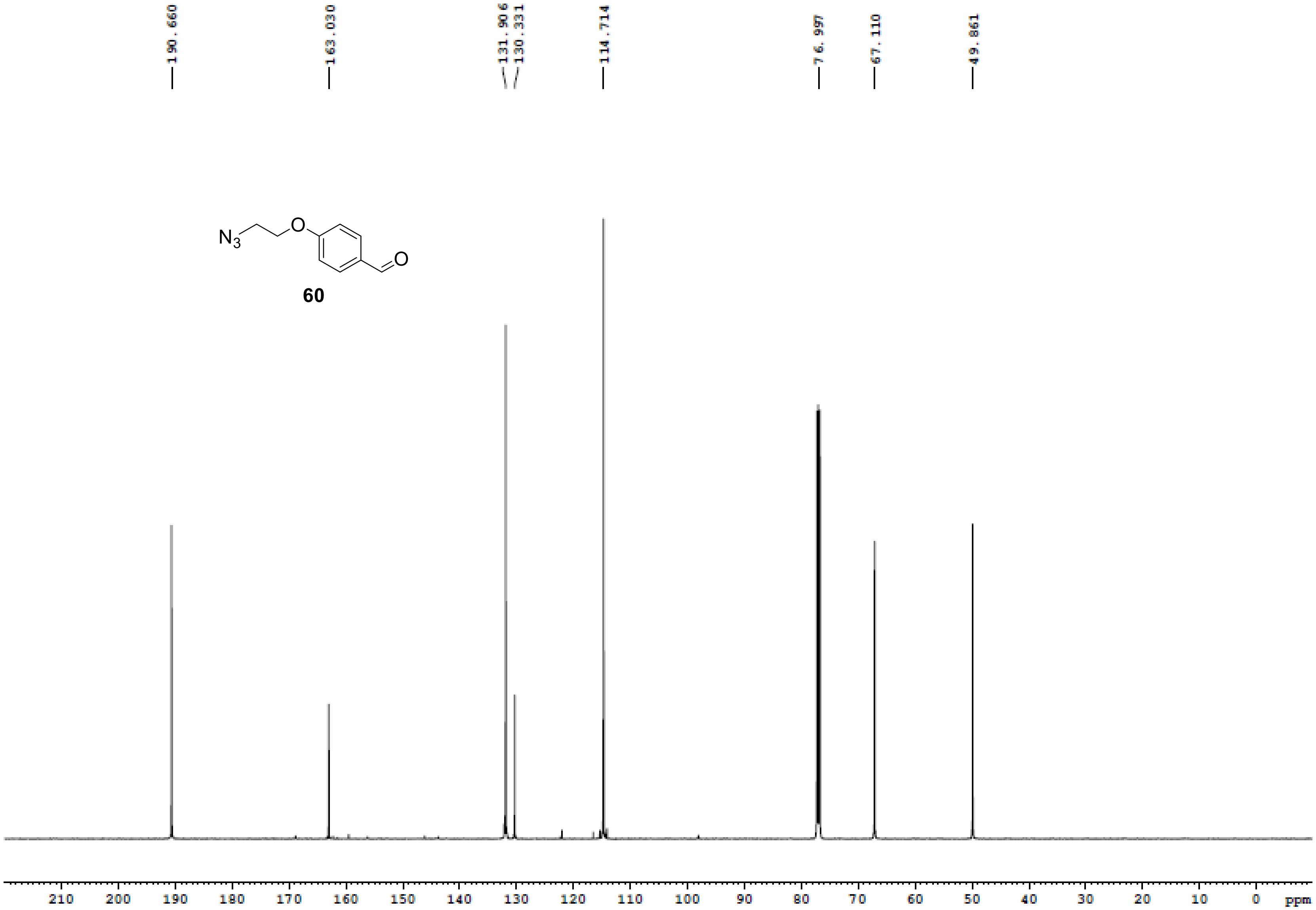

190.660
163.030
131.906
130.331
114.714
76.997
67.110
49.861
N3
O
O
210
200
190
180
170
160
150
140
130
120
110
100
90
80
70
60
50
40
30
20
10
0
ppm

60

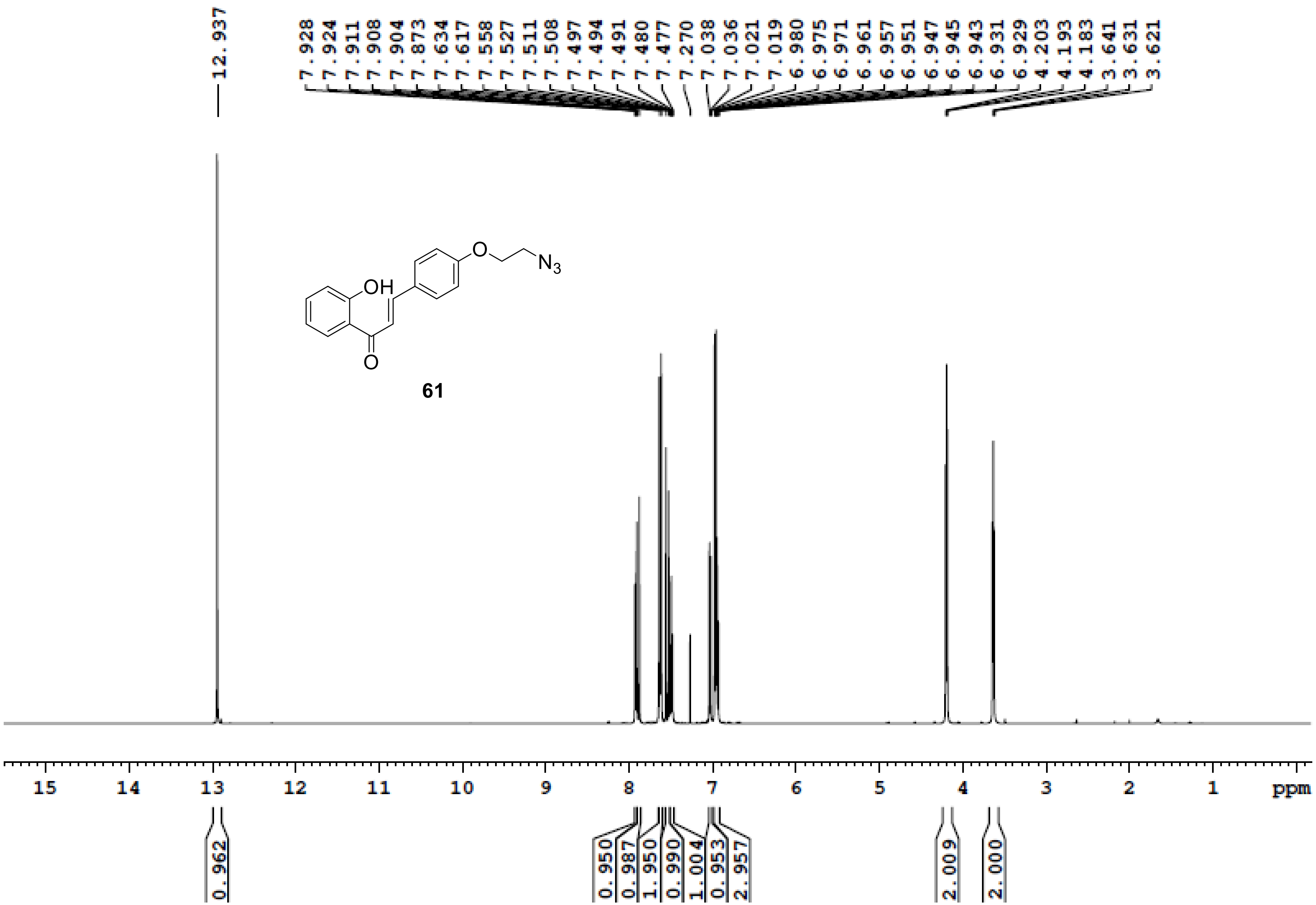
12.937
7.928 7.924 7.911 7.908 7.904 7.873 7.634 7.617 7.558 7.527 7.511 7.508 7.497 7.494 7.491 7.480 7.477 7.270 7.038 7.036 7.021 7.019 6.980 6.975 6.971 6.961 6.957 6.951 6.947 6.945 6.943 6.931 6.929 4.203 4.193 4.183 3.641 3.631 3.621
OH
O
N3
O
61
15 14 13 12 11 10 9 8 7 6 5 4 3 2 1 ppm
0.962
0.950 0.987 1.950 0.990 1.004 0.953 2.957
2.009
2.000

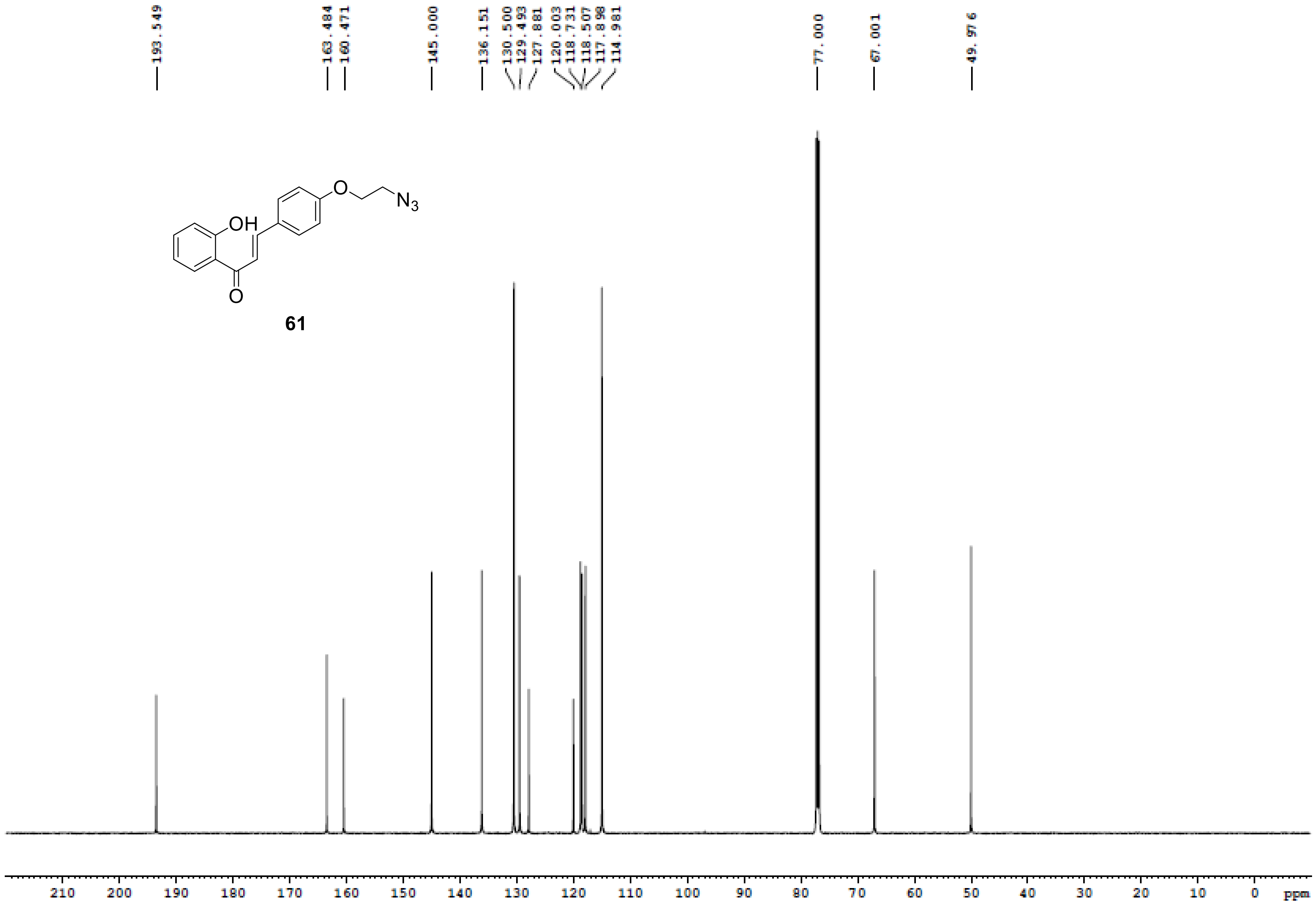
193.549
163.484
160.471
145.000
136.151
130.500
129.493
127.881
120.003
118.731
118.507
117.898
114.981
77.000
67.001
49.976
OH
O
O
N3
61
210 200 190 180 170 160 150 140 130 120 110 100 90 80 70 60 50 40 30 20 10 0 ppm

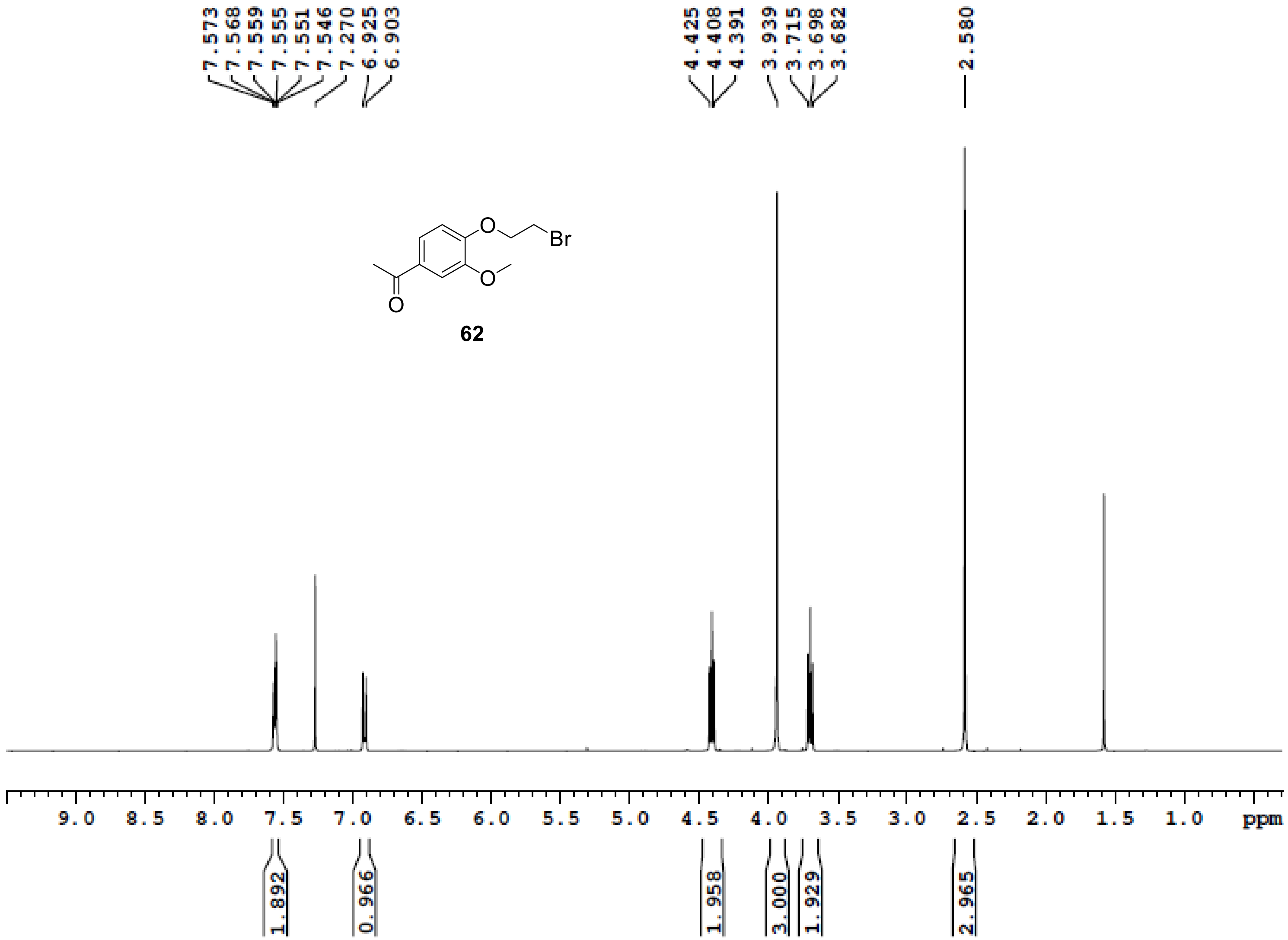
7.573
7.568
7.559
7.555
7.551
7.546
7.270
6.925
6.903
4.425
4.408
4.391
3.939
3.715
3.698
3.682
2.580
Br
O
O
O
62
9.0
8.5
8.0
7.5
7.0
6.5
6.0
5.5
5.0
4.5
4.0
3.5
3.0
2.5
2.0
1.5
1.0
ppm
1.892
0.966
1.958
3.000
1.929
2.965

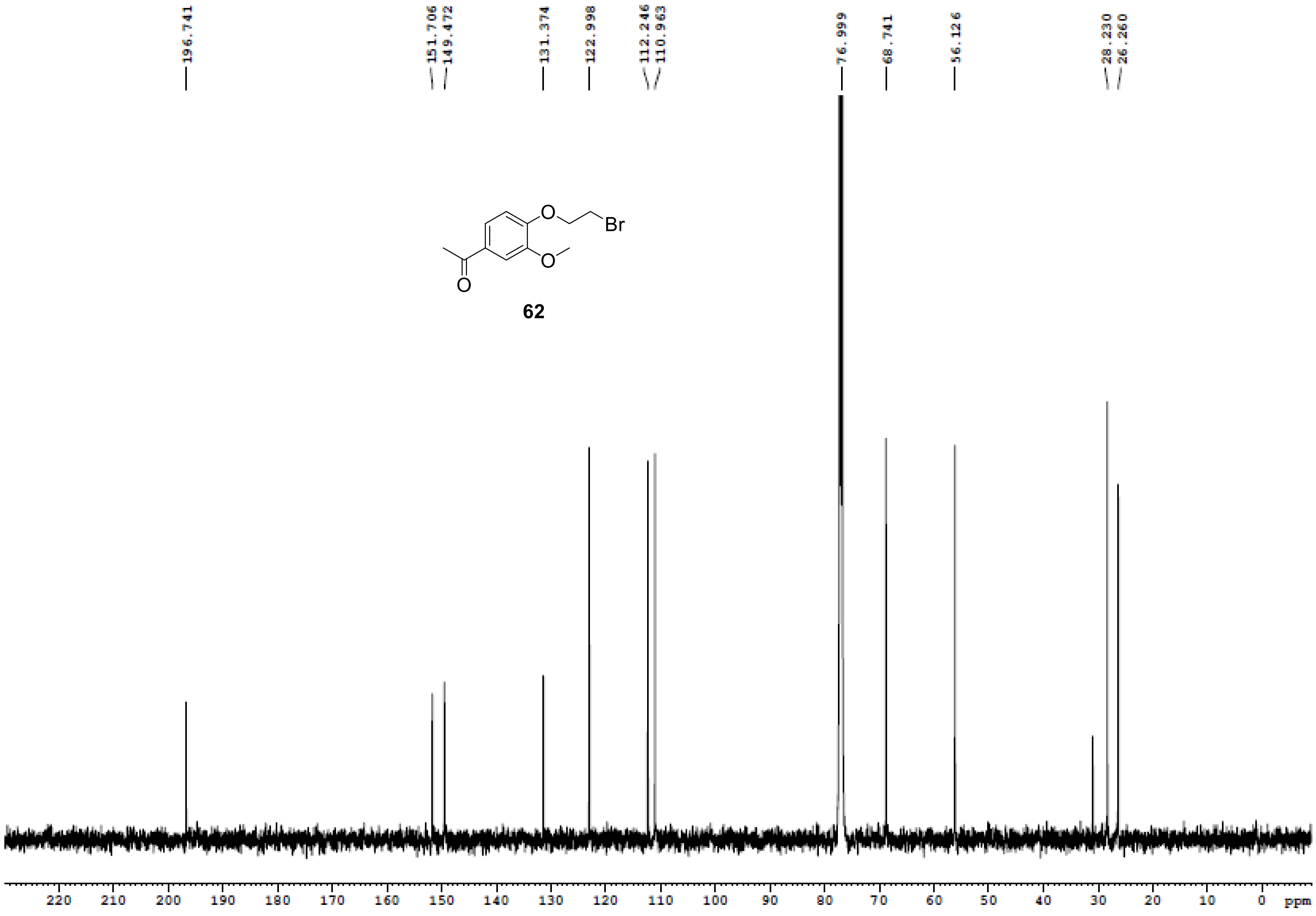
196.741
151.706
149.472
131.374
122.998
112.246
110.963
76.999
68.741
56.126
28.230
26.260
Br
O
O
O
62
220
210
200
190
180
170
160
150
140
130
120
110
100
90
80
70
60
50
40
30
20
10
0
ppm

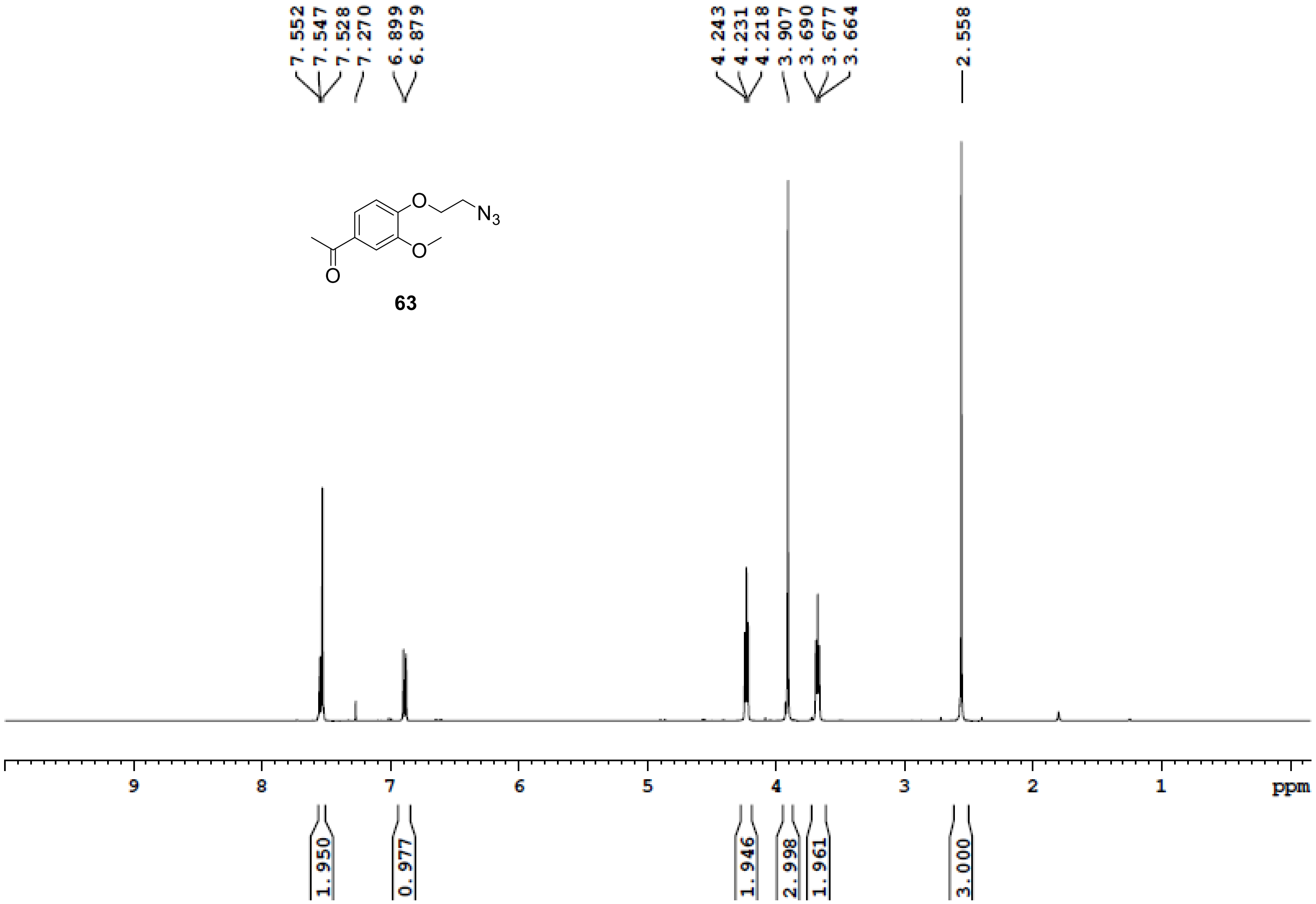
7.552
7.547
7.528
7.270
6.899
6.879
4.243
4.231
4.218
3.907
3.690
3.677
3.664
2.558
O
N3
O
O
63
9
8
7
6
5
4
3
2
1
ppm
1.950
0.977
1.946
2.998
1.961
3.000

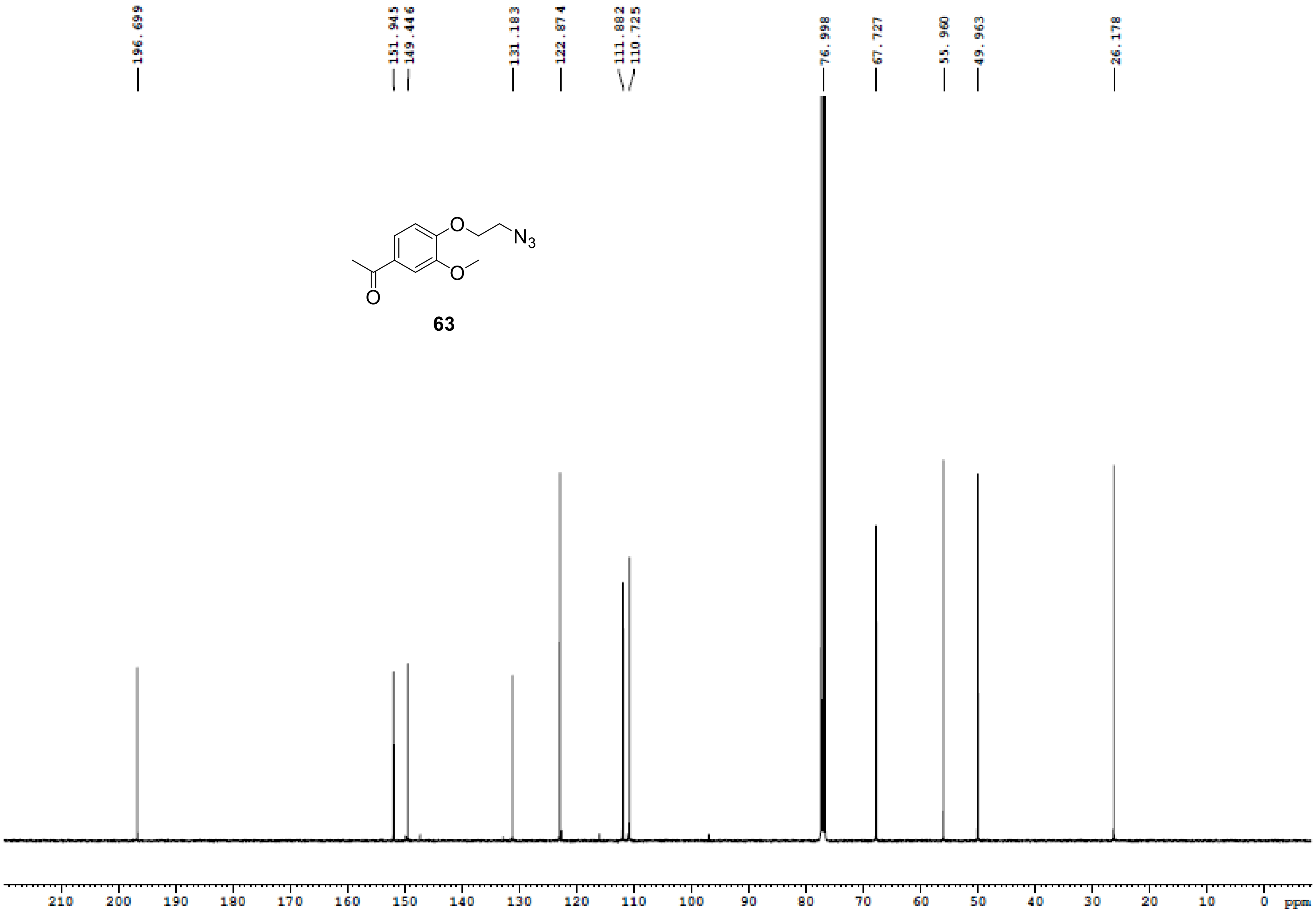
196.699
151.945
149.446
131.183
122.874
111.882
110.725
76.998
67.727
55.960
49.963
26.178
O
N3
O
O
63
210 200 190 180 170 160 150 140 130 120 110 100 90 80 70 60 50 40 30 20 10 0 ppm

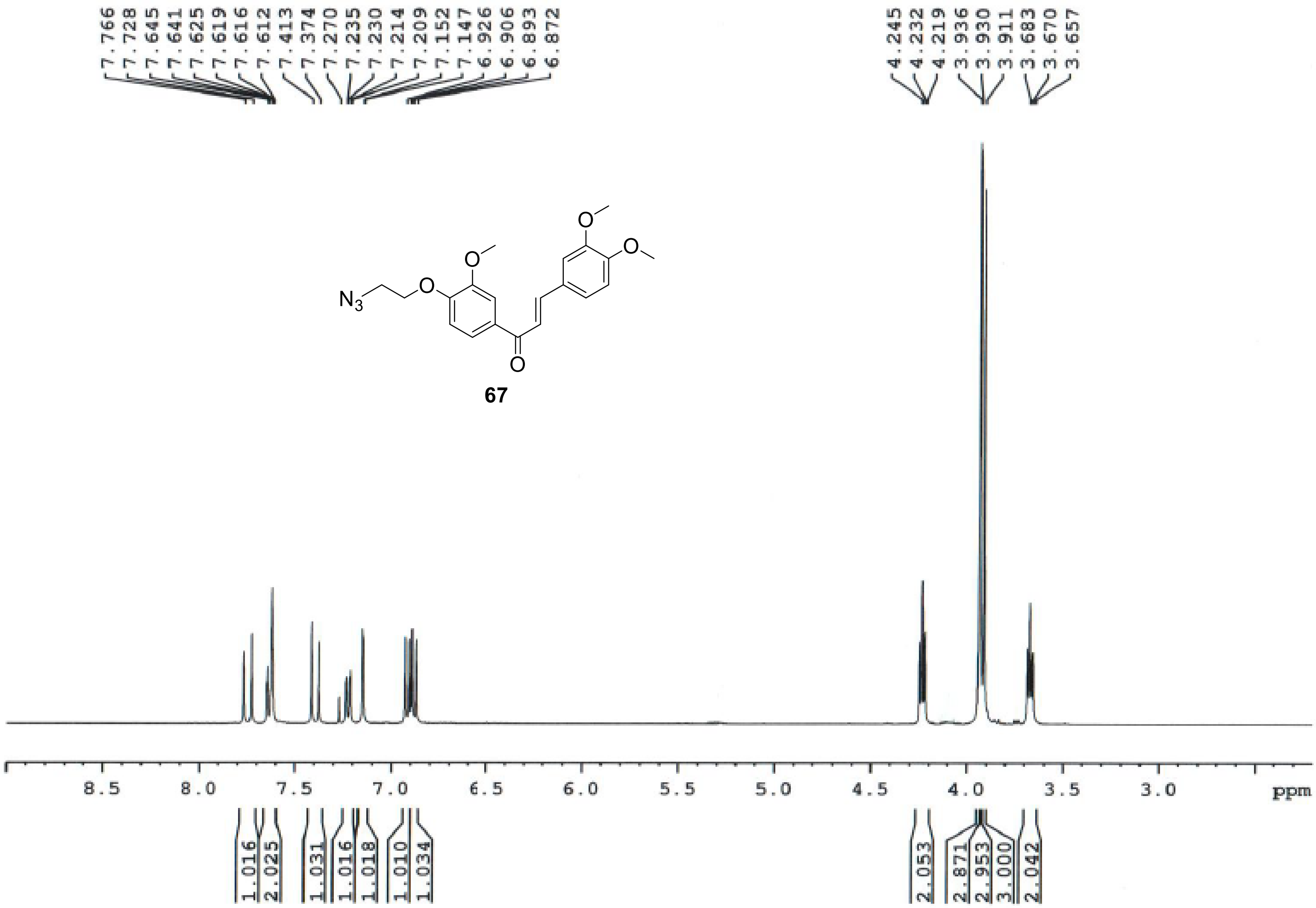
7.766
7.728
7.645
7.641
7.625
7.619
7.616
7.612
7.413
7.374
7.270
7.235
7.230
7.214
7.209
7.152
7.147
6.926
6.906
6.893
6.872
4.245
4.232
4.219
3.936
3.930
3.911
3.683
3.670
3.657
N3
67
8.5
8.0
7.5
7.0
6.5
6.0
5.5
5.0
4.5
4.0
3.5
3.0
ppm
1.016
2.025
1.031
1.016
1.018
1.010
1.034
2.053
2.871
2.953
3.000
2.042

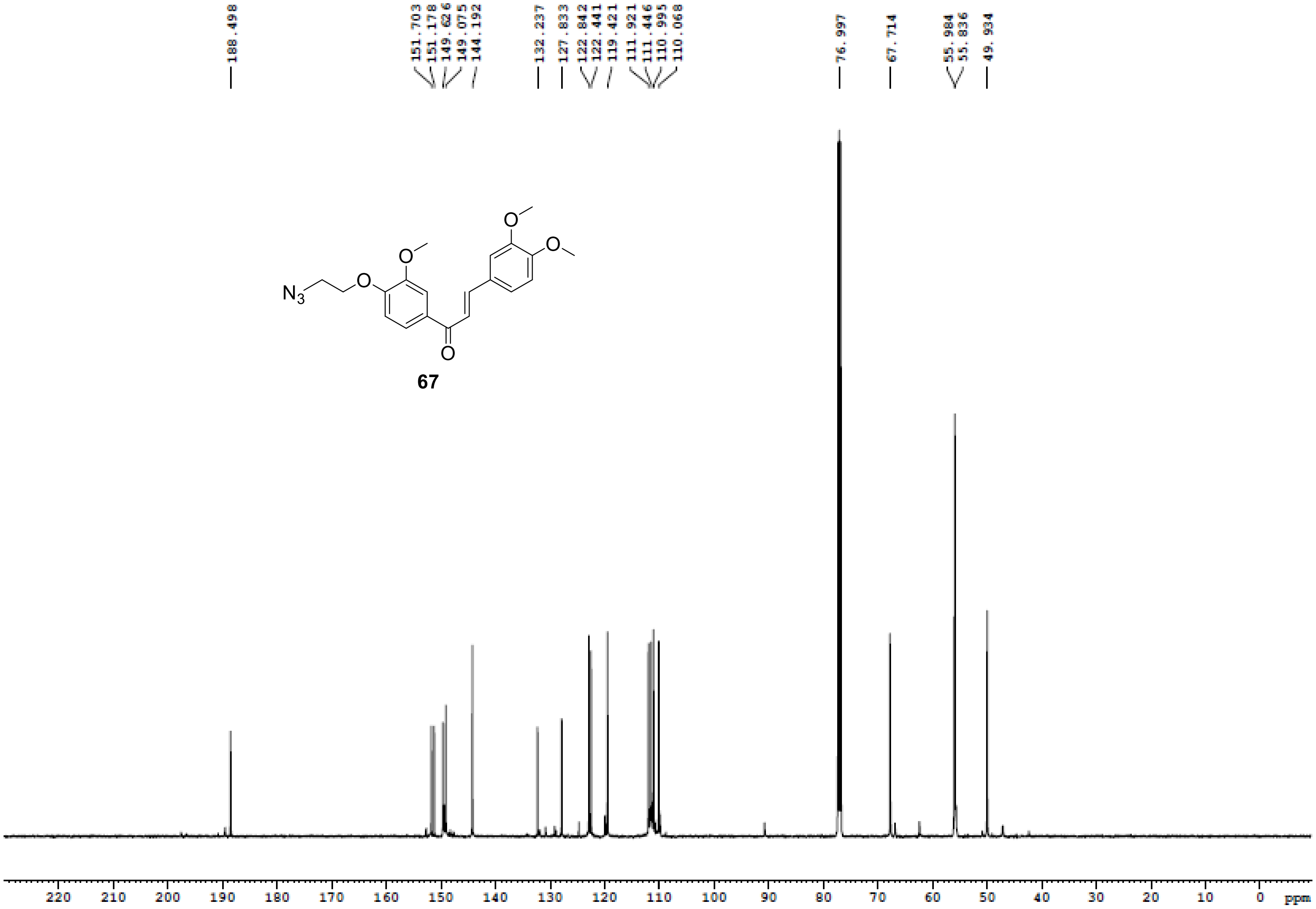
188.498
151.703
151.178
149.626
149.075
144.192
132.237
127.833
122.842
122.441
119.421
111.921
111.446
110.995
110.068
76.997
67.714
55.984
55.836
49.934
N3
O
O
O
O
O
67
220 210 200 190 180 170 160 150 140 130 120 110 100 90 80 70 60 50 40 30 20 10 0 ppm

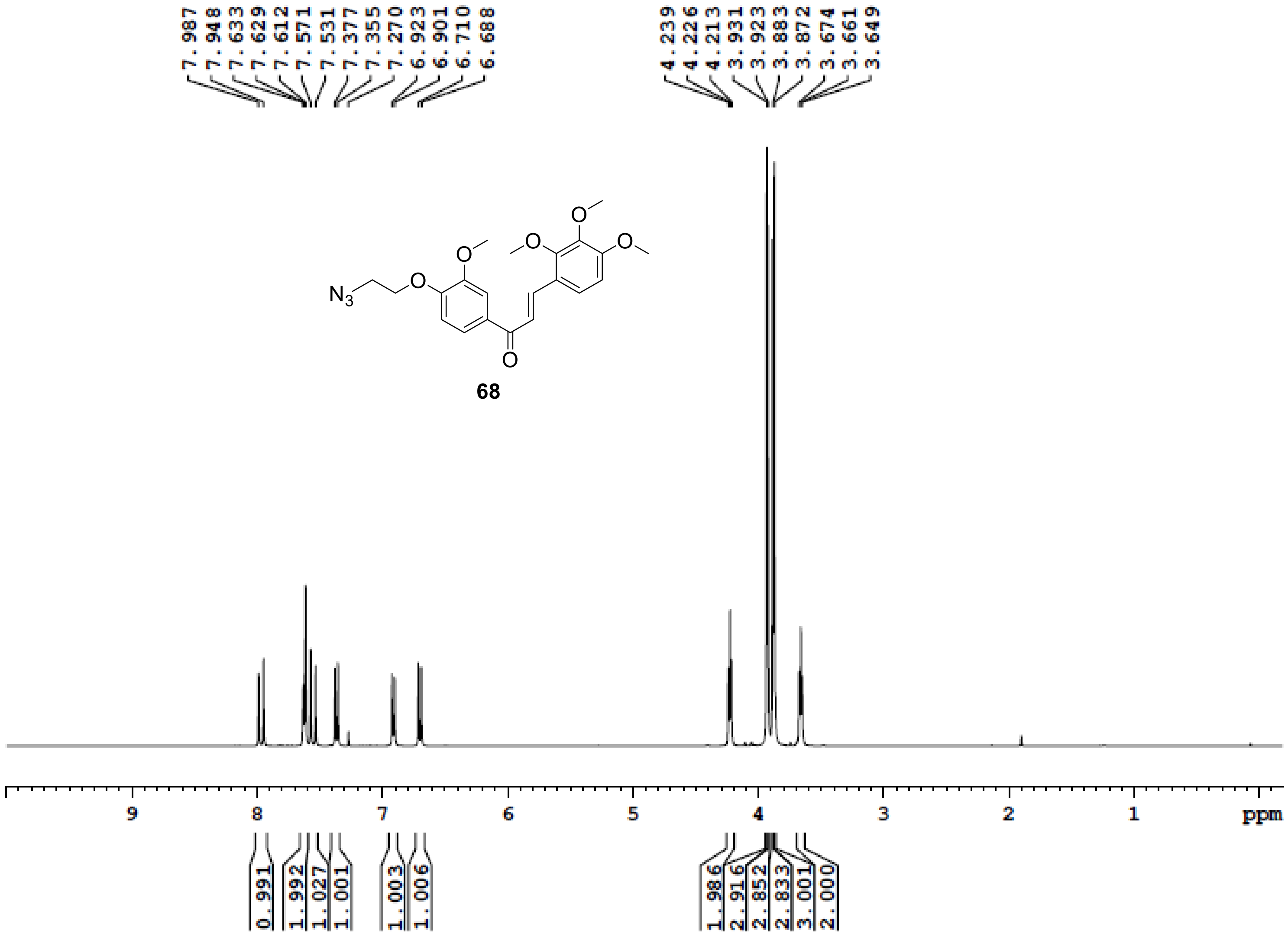
7.987
7.948
7.633
7.629
7.612
7.571
7.531
7.377
7.355
7.270
6.923
6.901
6.710
6.688
4.239
4.226
4.213
3.931
3.923
3.883
3.872
3.674
3.661
3.649
N_3
O
68
9
8
7
6
5
4
3
2
1
ppm
0.991
1.992
1.027
1.001
1.003
1.006
1.986
2.916
2.852
2.833
3.001
2.000

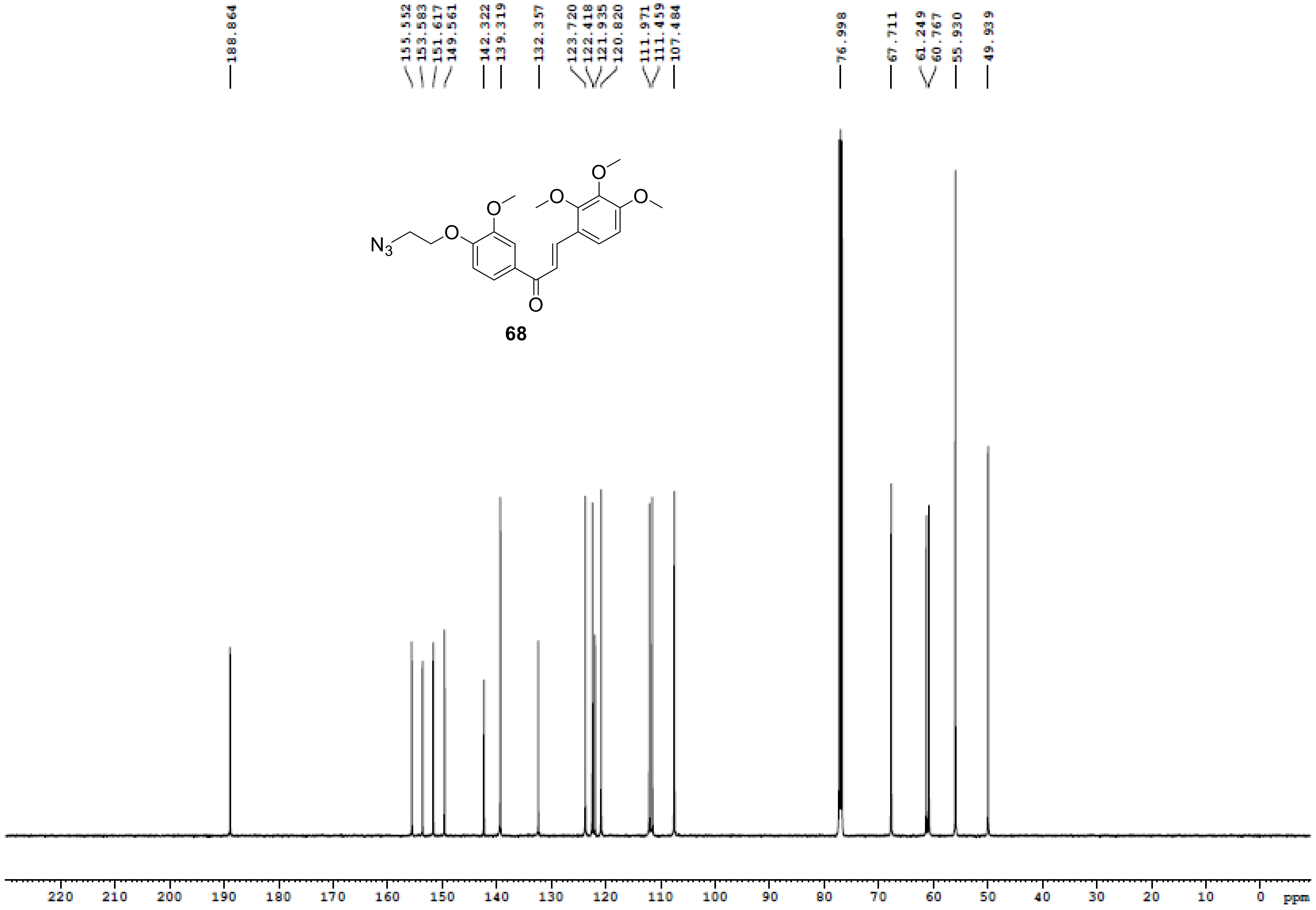
188.864
155.552
153.583
151.617
149.561
142.322
139.319
132.357
123.720
122.418
121.935
120.820
111.971
111.459
107.484
76.998
67.711
61.249
60.767
55.930
49.939
68
220 210 200 190 180 170 160 150 140 130 120 110 100 90 80 70 60 50 40 30 20 10 0 ppm

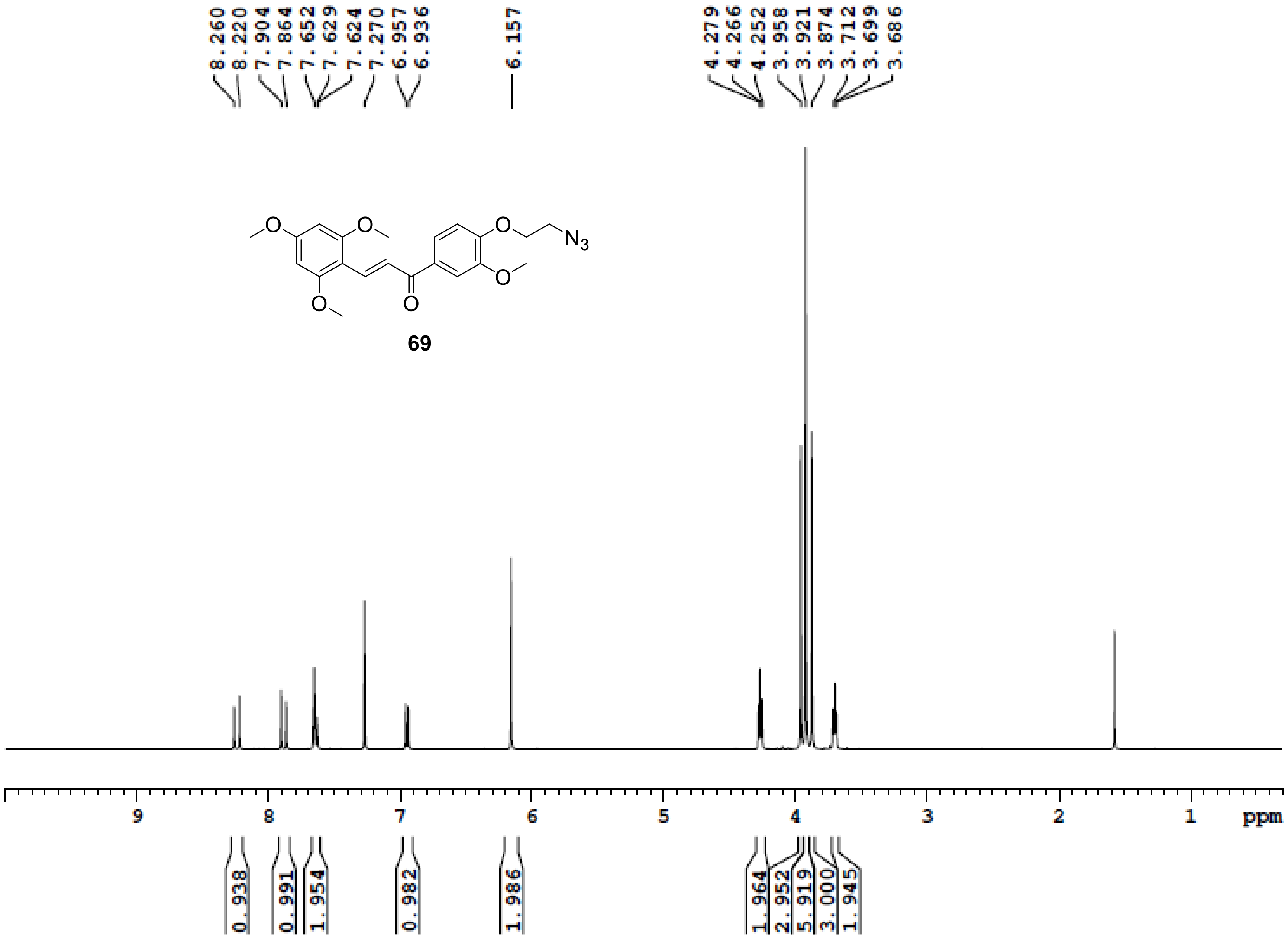
8.260
8.220
7.904
7.864
7.652
7.629
7.624
7.270
6.957
6.936
6.157
4.279
4.266
4.252
3.958
3.921
3.874
3.712
3.699
3.686
O
O
O
O
O
O
N3
69
9
8
7
6
5
4
3
2
1
ppm
0.938
0.991
1.954
0.982
1.986
1.964
2.952
5.919
3.000
1.945

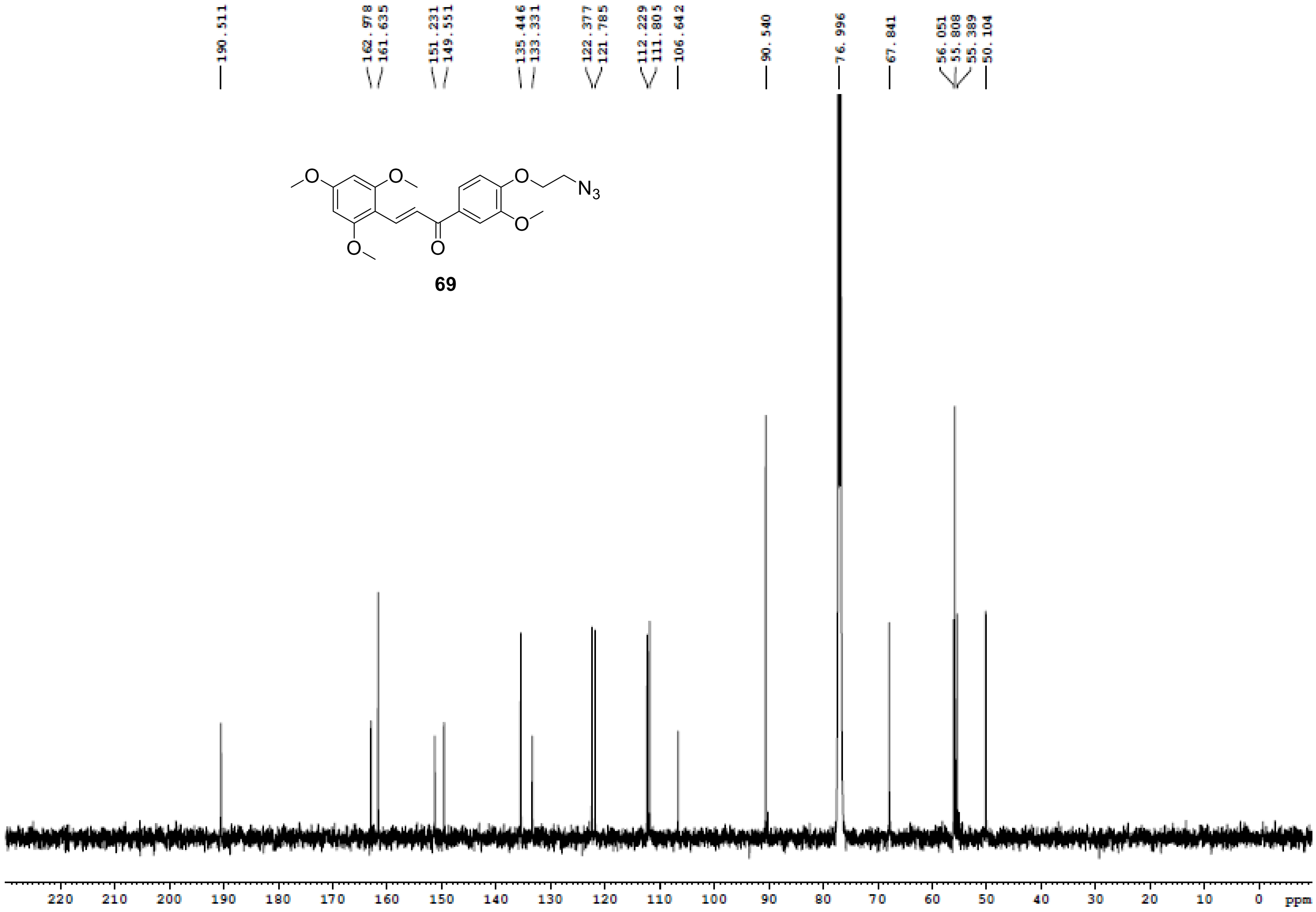
190.511
162.978
161.635
151.231
149.551
135.446
133.331
122.377
121.785
112.229
111.805
106.642
90.540
76.996
67.841
56.051
55.808
55.389
50.104
O
O
O
O
O
O
N3
69
220 210 200 190 180 170 160 150 140 130 120 110 100 90 80 70 60 50 40 30 20 10 0 ppm

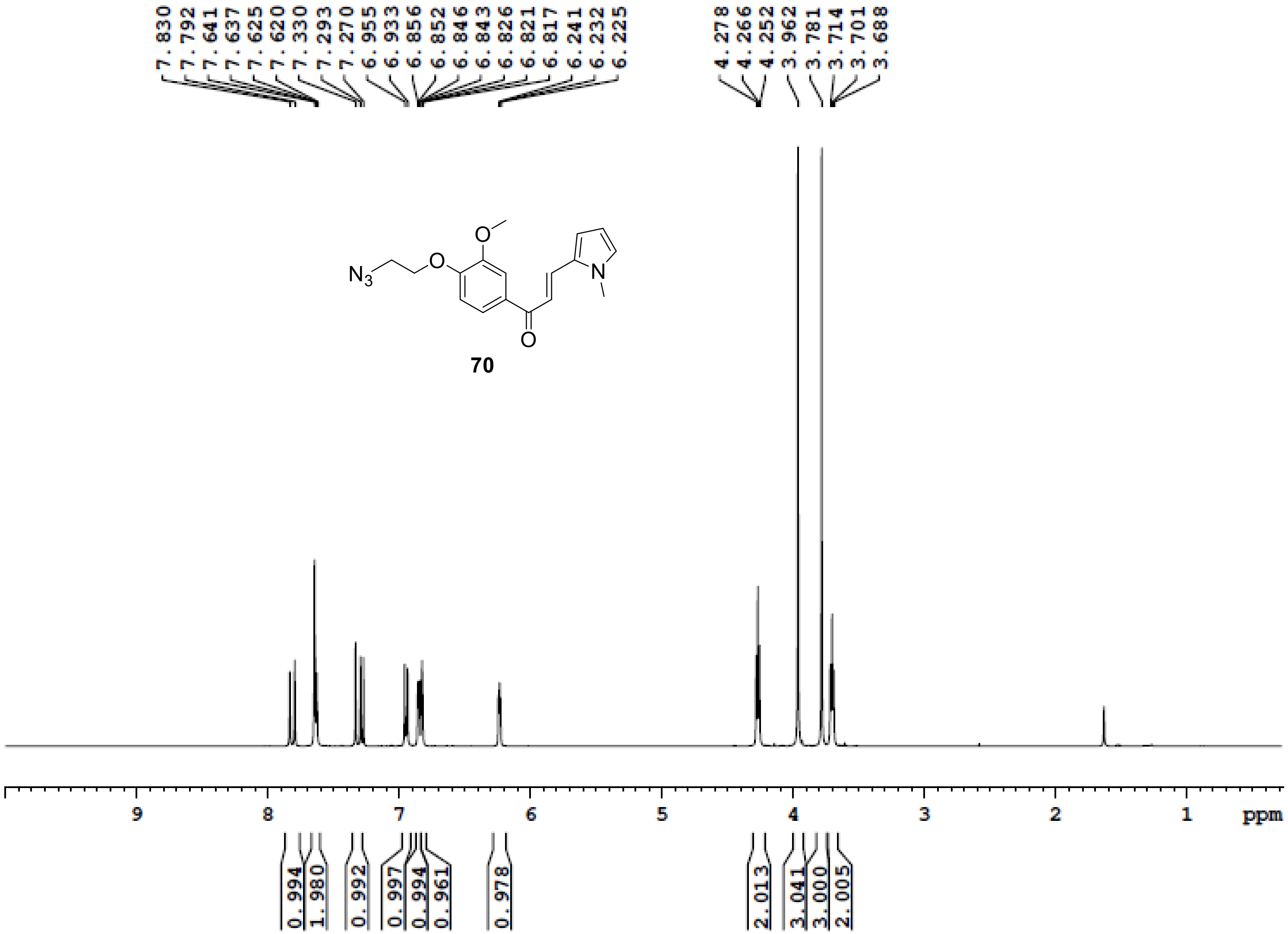
7.830
7.792
7.641
7.637
7.625
7.620
7.330
7.293
7.270
6.955
6.933
6.856
6.852
6.846
6.843
6.826
6.821
6.817
6.241
6.232
6.225
4.278
4.266
4.252
3.962
3.781
3.714
3.701
3.688
O
N3
O
N
O
70
9
8
7
6
5
4
3
2
1
ppm
0.994
1.980
0.992
0.997
0.994
0.961
0.978
2.013
3.041
3.000
2.005

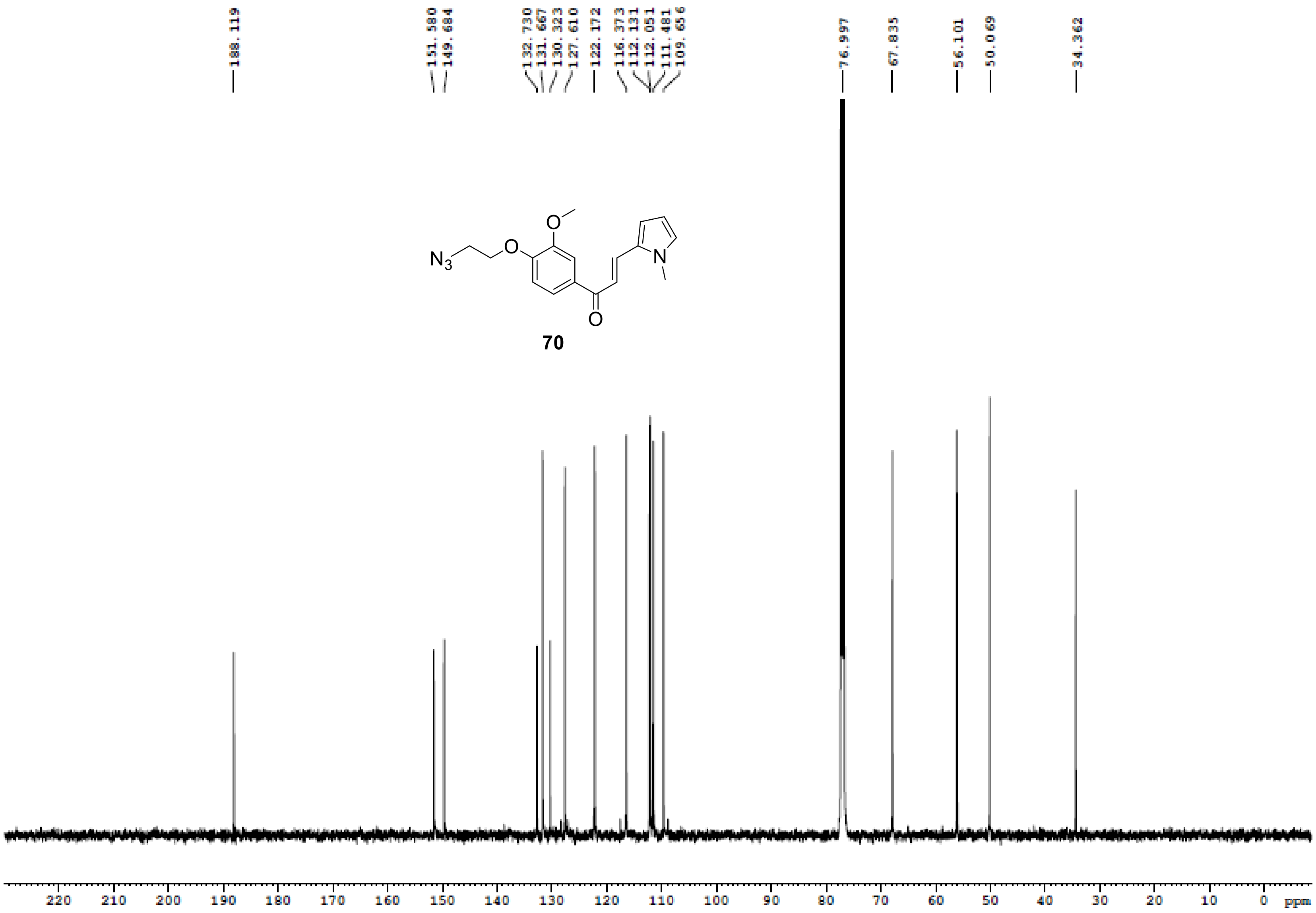
188.119
151.580
149.684
132.730
131.667
130.323
127.610
122.172
116.373
112.131
112.051
111.481
109.656
76.997
67.835
56.101
50.069
34.362
N_3
O
O
N
O
70
220 210 200 190 180 170 160 150 140 130 120 110 100 90 80 70 60 50 40 30 20 10 0 ppm

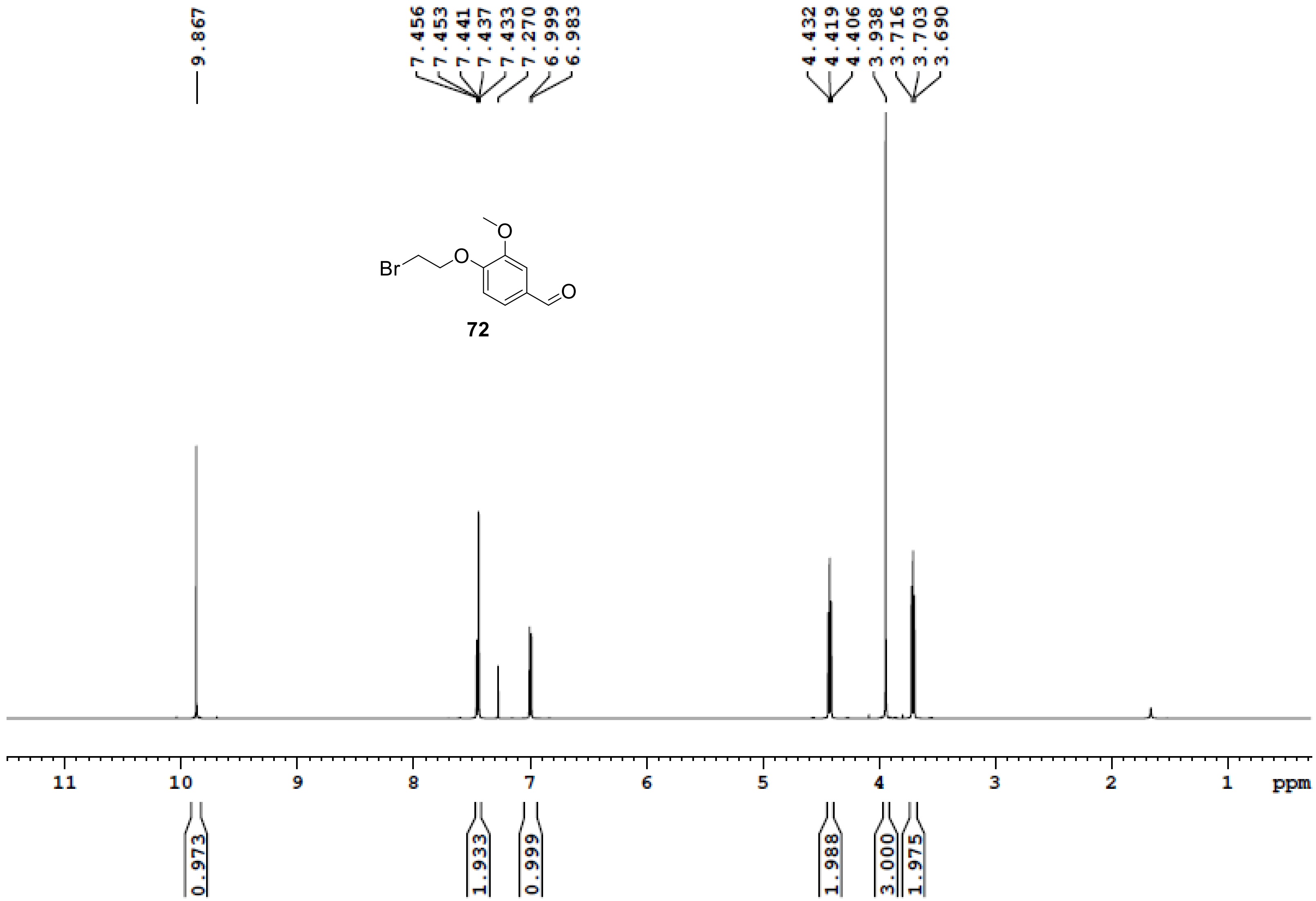
9.867
7.456 7.453 7.441 7.437 7.433 7.270 6.999 6.983
4.432 4.419 4.406 3.938 3.716 3.703 3.690
Br
O
O
O
72
11 10 9 8 7 6 5 4 3 2 1 ppm
0.973 1.933 0.999 1.988 3.000 1.975

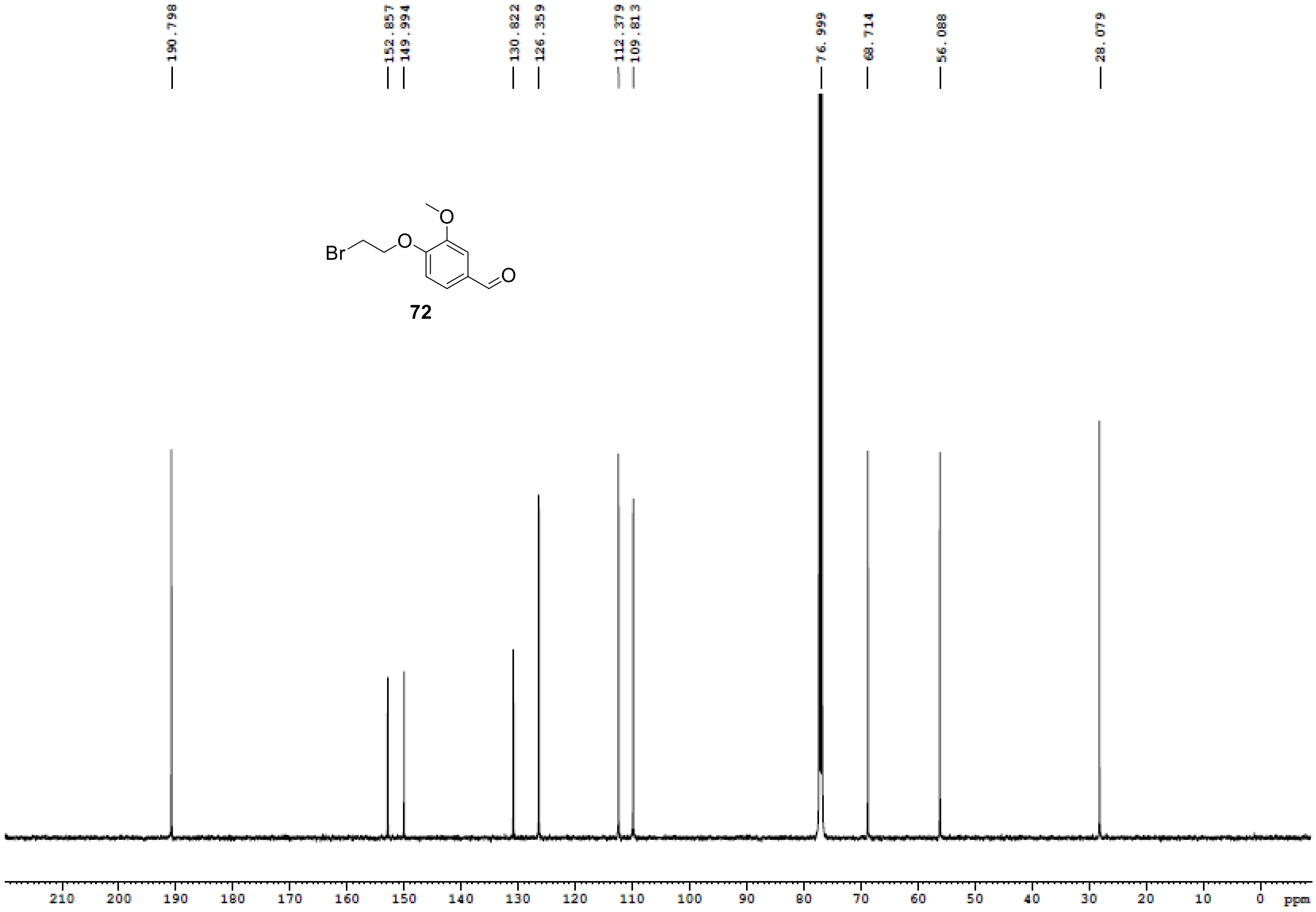
190.798
152.857
149.994
130.822
126.359
112.379
109.813
76.999
68.714
56.088
28.079
Br
O
O
O
72
210 200 190 180 170 160 150 140 130 120 110 100 90 80 70 60 50 40 30 20 10 0 ppm

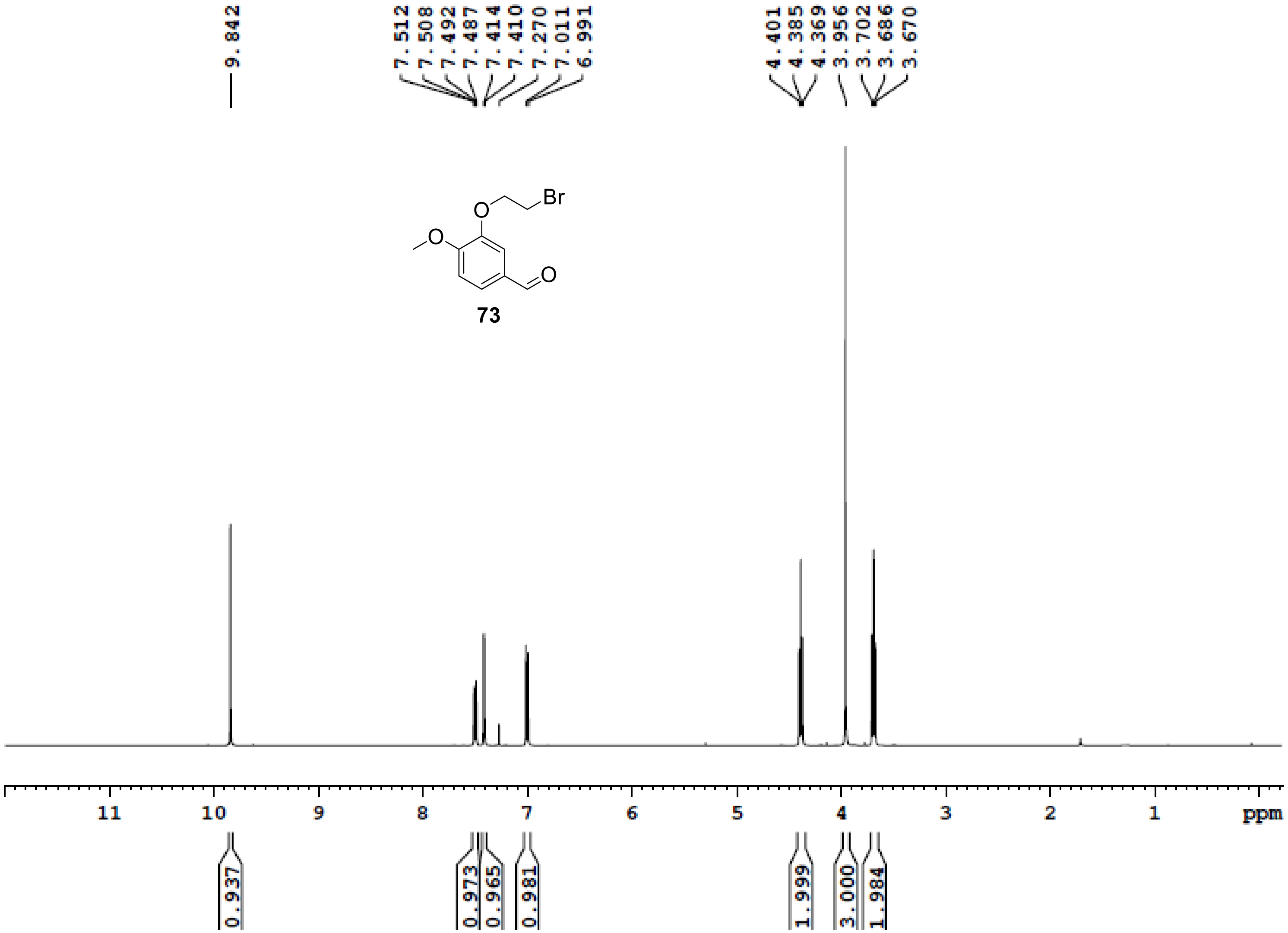
9.842
7.512
7.508
7.492
7.487
7.414
7.410
7.270
7.011
6.991
4.401
4.385
4.369
3.956
3.702
3.686
3.670
Br
O
O
O
73
11
10
9
8
7
6
5
4
3
2
1
ppm
0.937
0.973
0.965
0.981
1.999
3.000
1.984

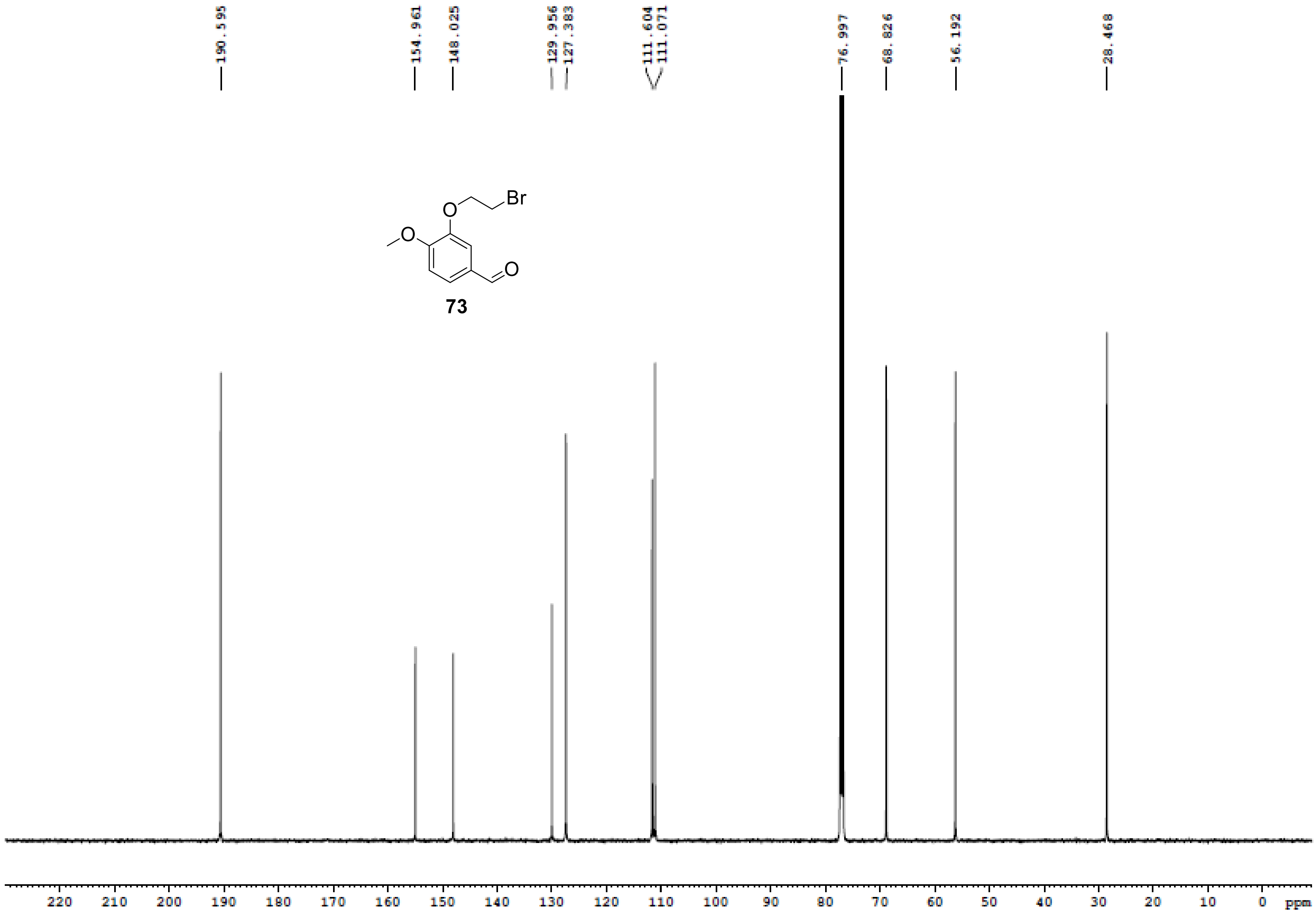
190.595
154.961
148.025
129.956
127.383
111.604
111.071
76.997
68.826
56.192
28.468
Br
O
O
O
73
220 210 200 190 180 170 160 150 140 130 120 110 100 90 80 70 60 50 40 30 20 10 0 ppm

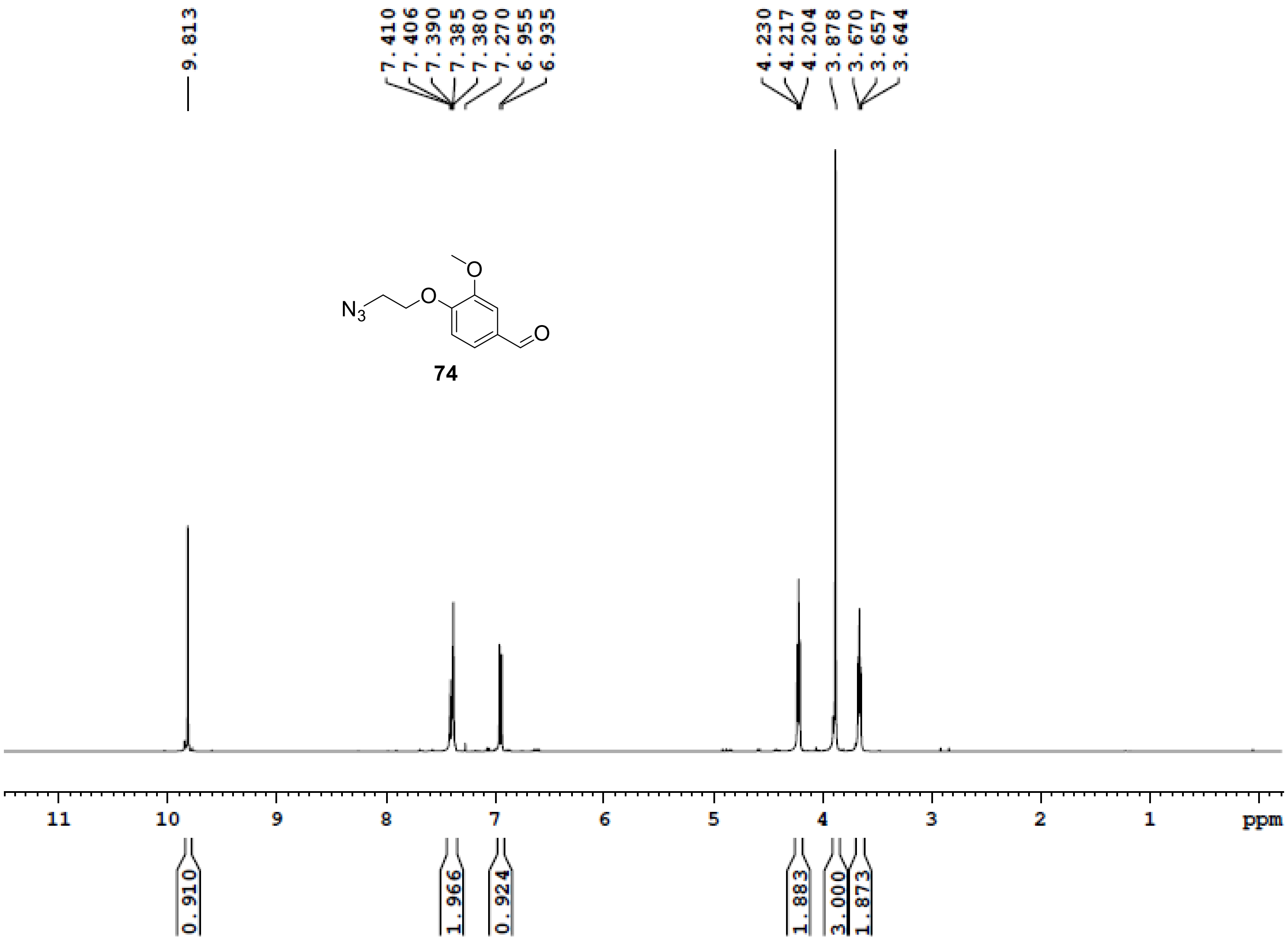
9.813
7.410
7.406
7.390
7.385
7.380
7.270
6.955
6.935
4.230
4.217
4.204
3.878
3.670
3.657
3.644
O
N3
O
O
74
11
10
9
8
7
6
5
4
3
2
1
ppm
0.910
1.966
0.924
1.883
3.000
1.873

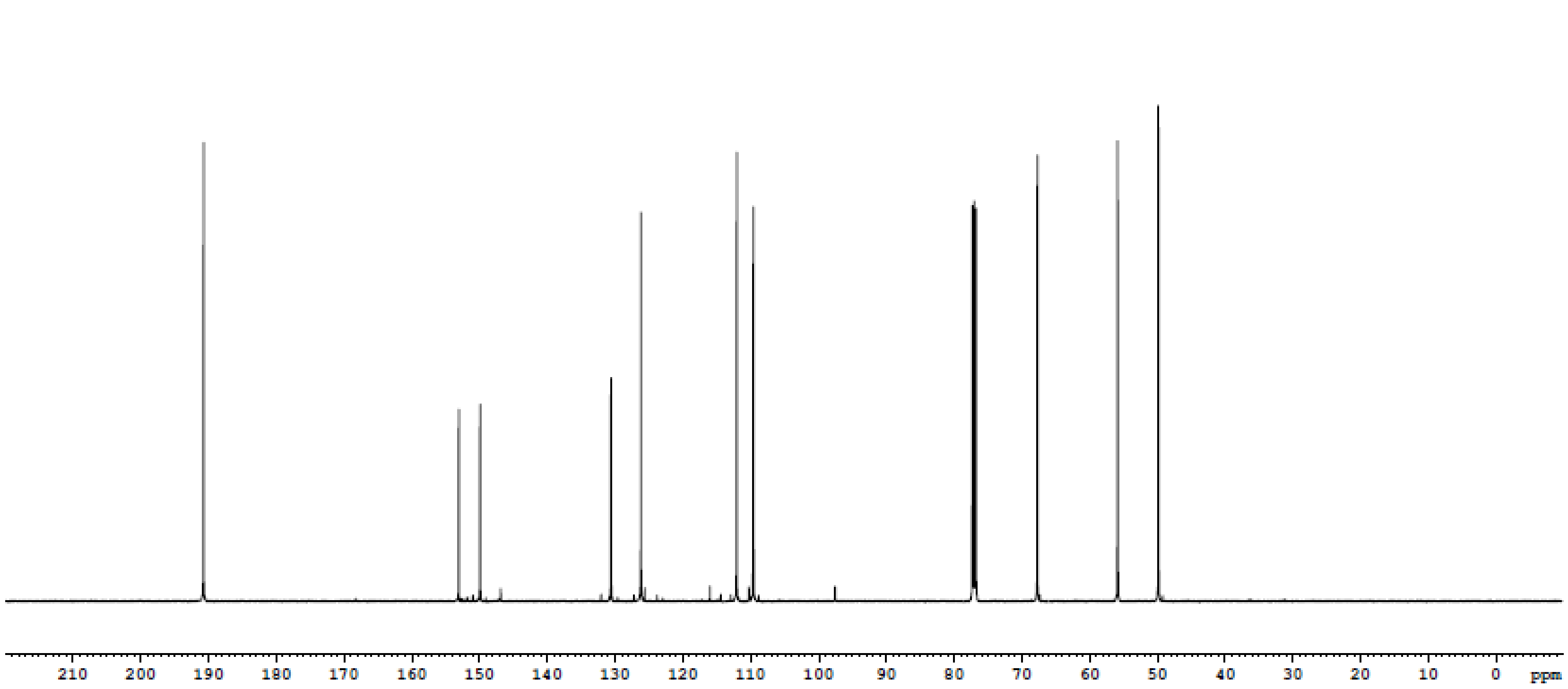

190.711
153.039
149.888
130.561
126.136
111.992
109.547
76.996
67.673
55.819
49.801
210 200 190 180 170 160 150 140 130 120 110 100 90 80 70 60 50 40 30 20 10 0 ppm

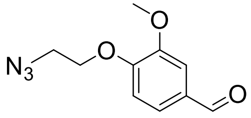

O
N3
O
O

74

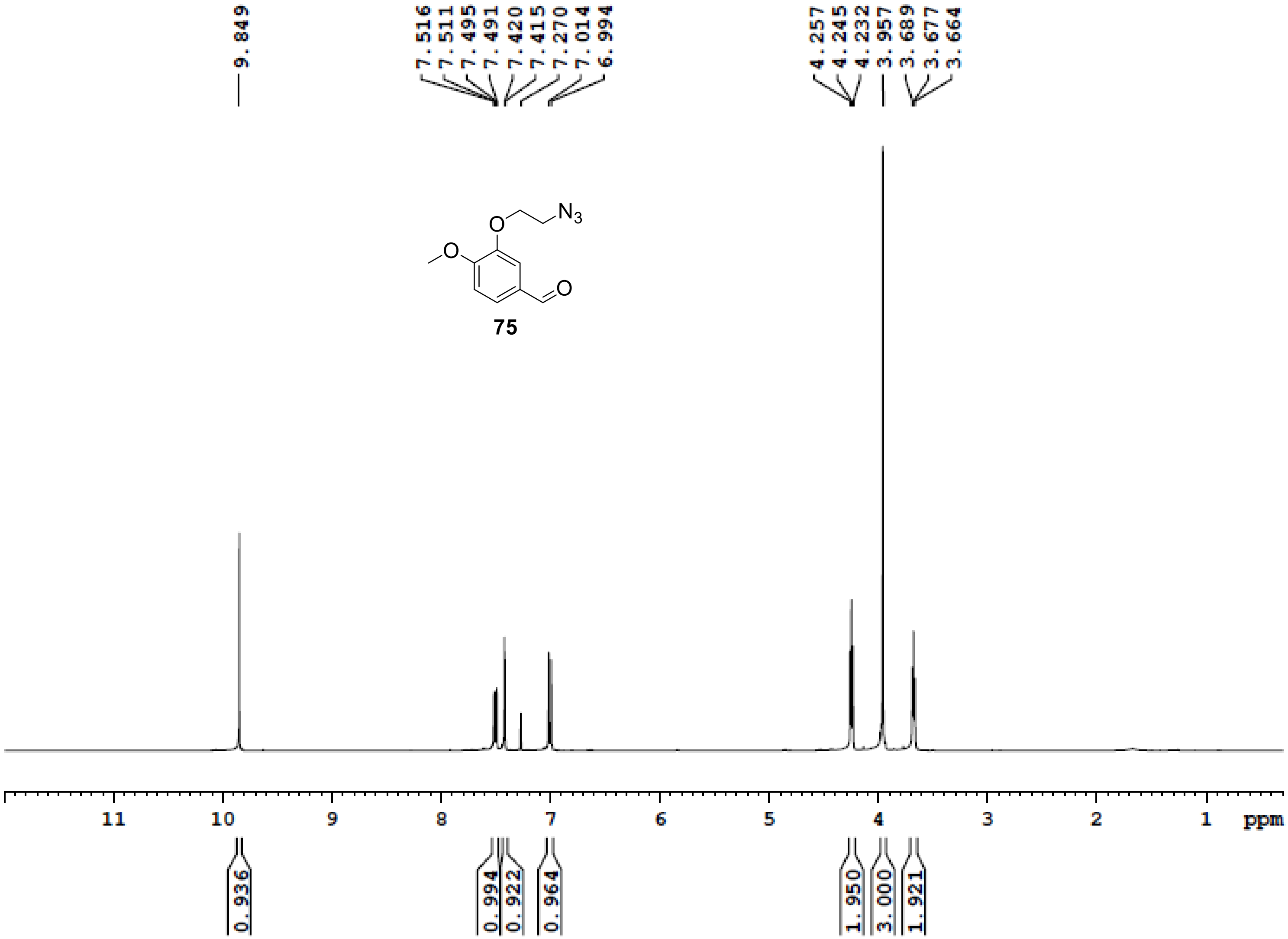
9.849
7.516
7.511
7.495
7.491
7.420
7.415
7.270
7.014
6.994
4.257
4.245
4.232
3.957
3.689
3.677
3.664
N3
O
O
O
75
11
10
9
8
7
6
5
4
3
2
1
ppm
0.936
0.994
0.922
0.964
1.950
3.000
1.921

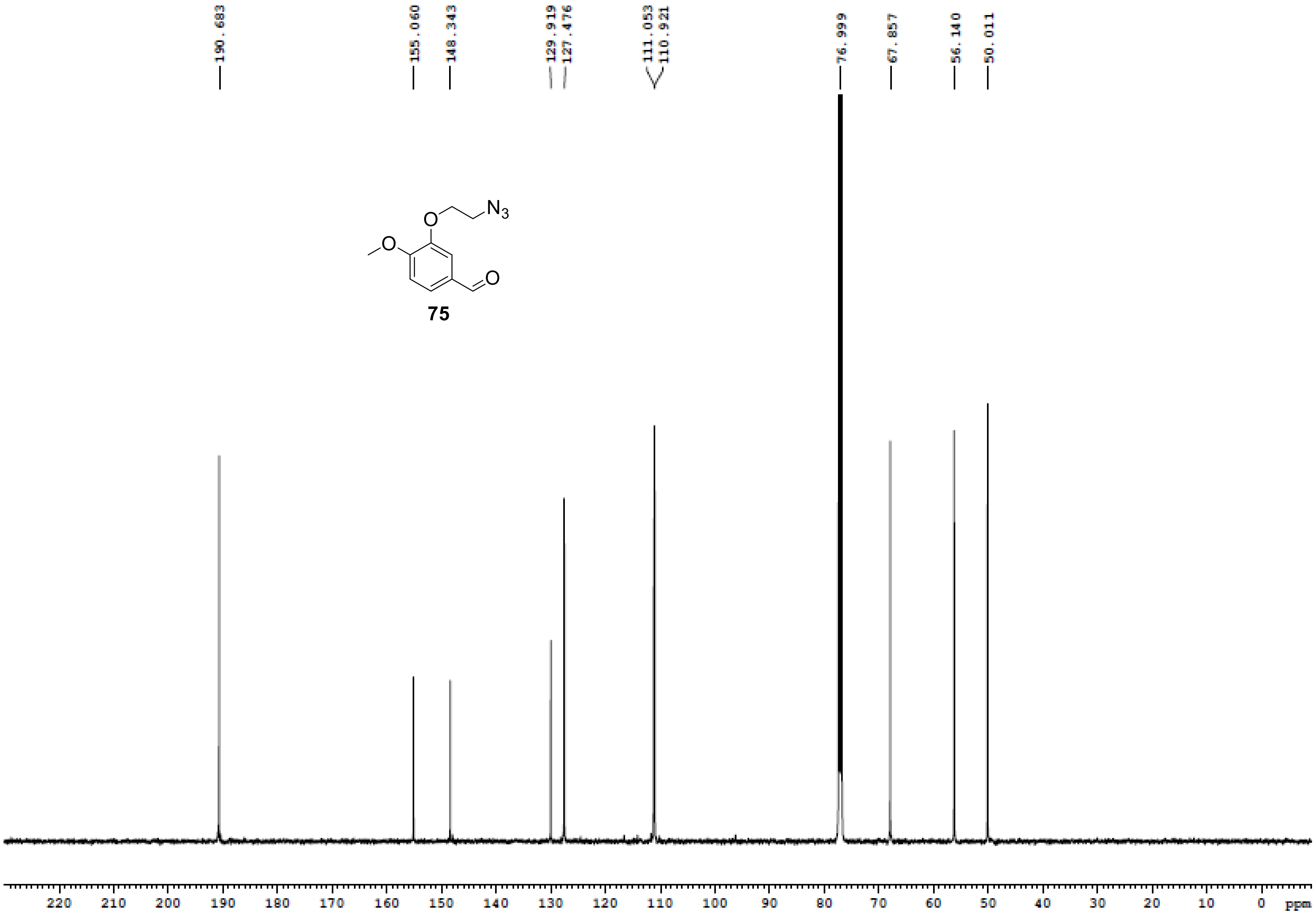
190.683
155.060
148.343
129.919
127.476
111.053
110.921
76.999
67.857
56.140
50.011
O
N3
O
O
75
220
210
200
190
180
170
160
150
140
130
120
110
100
90
80
70
60
50
40
30
20
10
0
ppm

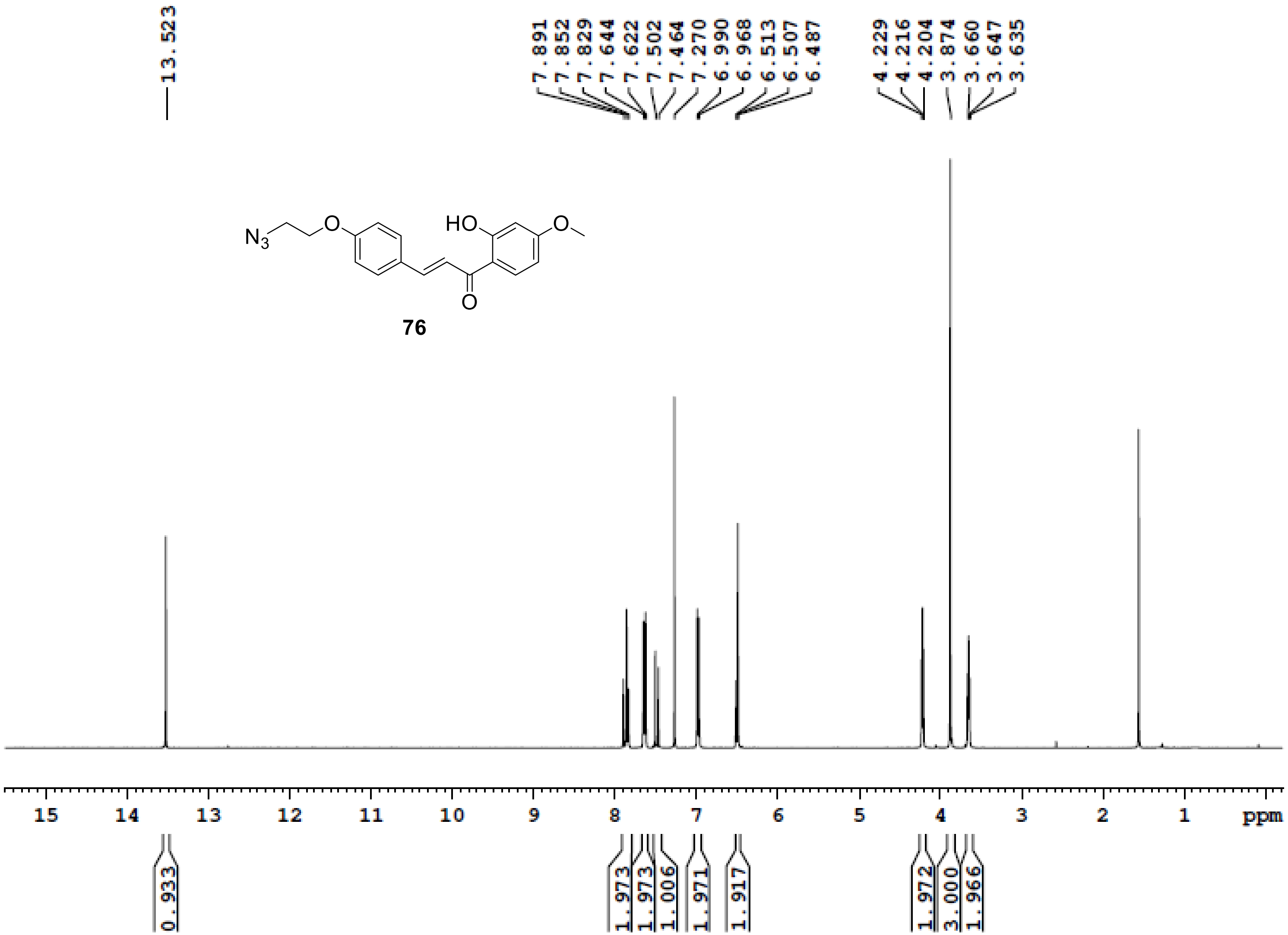
13.523
7.891
7.852
7.829
7.644
7.622
7.502
7.464
7.270
6.990
6.968
6.513
6.507
6.487
4.229
4.216
4.204
3.874
3.660
3.647
3.635
N_3
O
HO
O
O
76
15 14 13 12 11 10 9 8 7 6 5 4 3 2 1 ppm
0.933
1.973
1.973
1.006
1.971
1.917
1.972
3.000
1.966

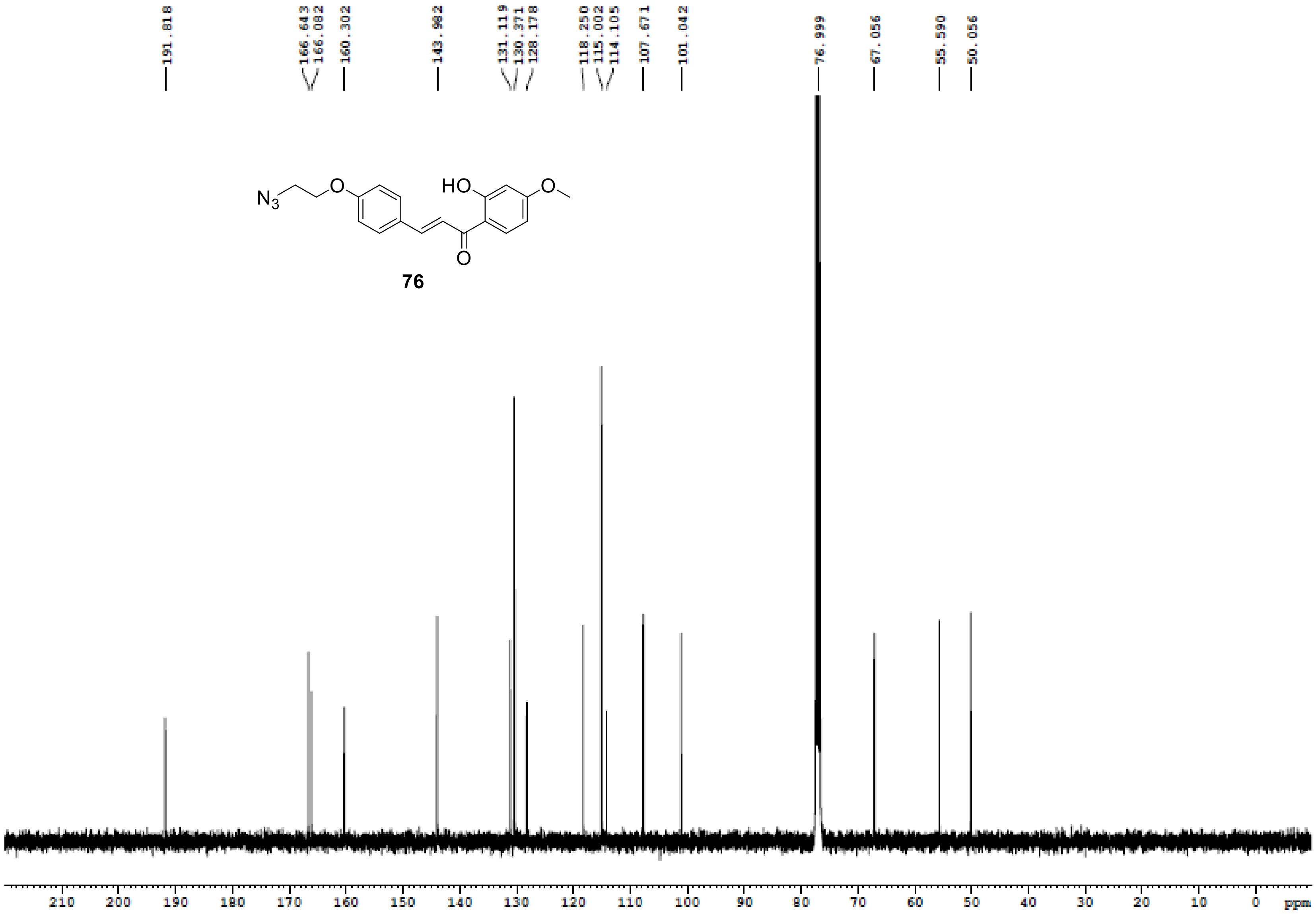

191.818
166.643
166.082
160.302
143.982
131.119
130.371
128.178
118.250
115.002
114.105
107.671
101.042
76.999
67.056
55.590
50.056
HO
O
N3
O
O
210
200
190
180
170
160
150
140
130
120
110
100
90
80
70
60
50
40
30
20
10
0
ppm

76

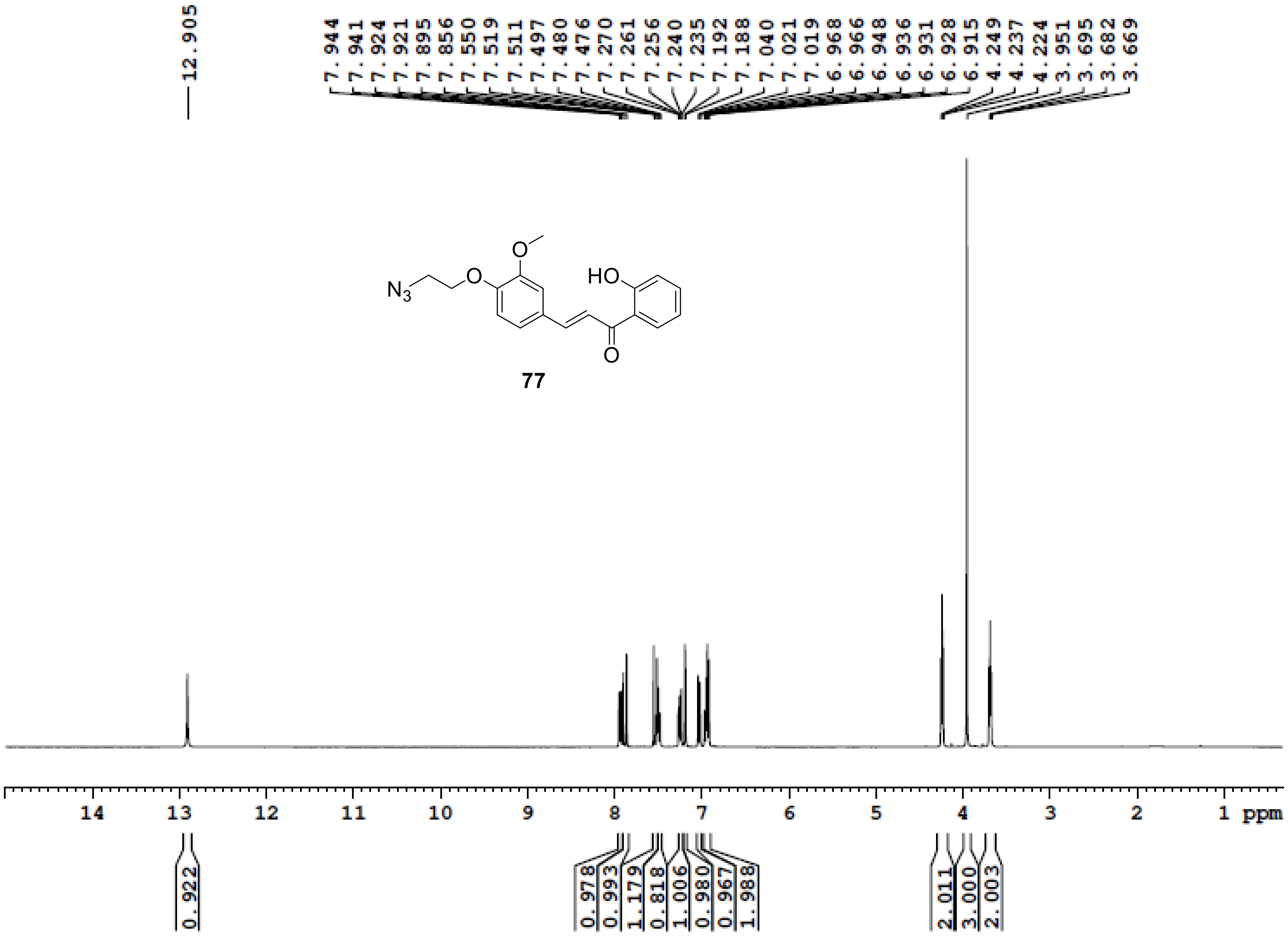
12.905
7.944
7.941
7.924
7.921
7.895
7.856
7.550
7.519
7.511
7.497
7.480
7.476
7.270
7.261
7.256
7.240
7.235
7.192
7.188
7.040
7.021
7.019
6.968
6.966
6.948
6.936
6.931
6.928
6.915
4.249
4.237
4.224
3.951
3.695
3.682
3.669
N3
O
O
HO
O
77
14
13
12
11
10
9
8
7
6
5
4
3
2
1 ppm
0.922
0.978
0.993
1.179
0.818
1.006
0.980
0.967
1.988
2.011
3.000
2.003

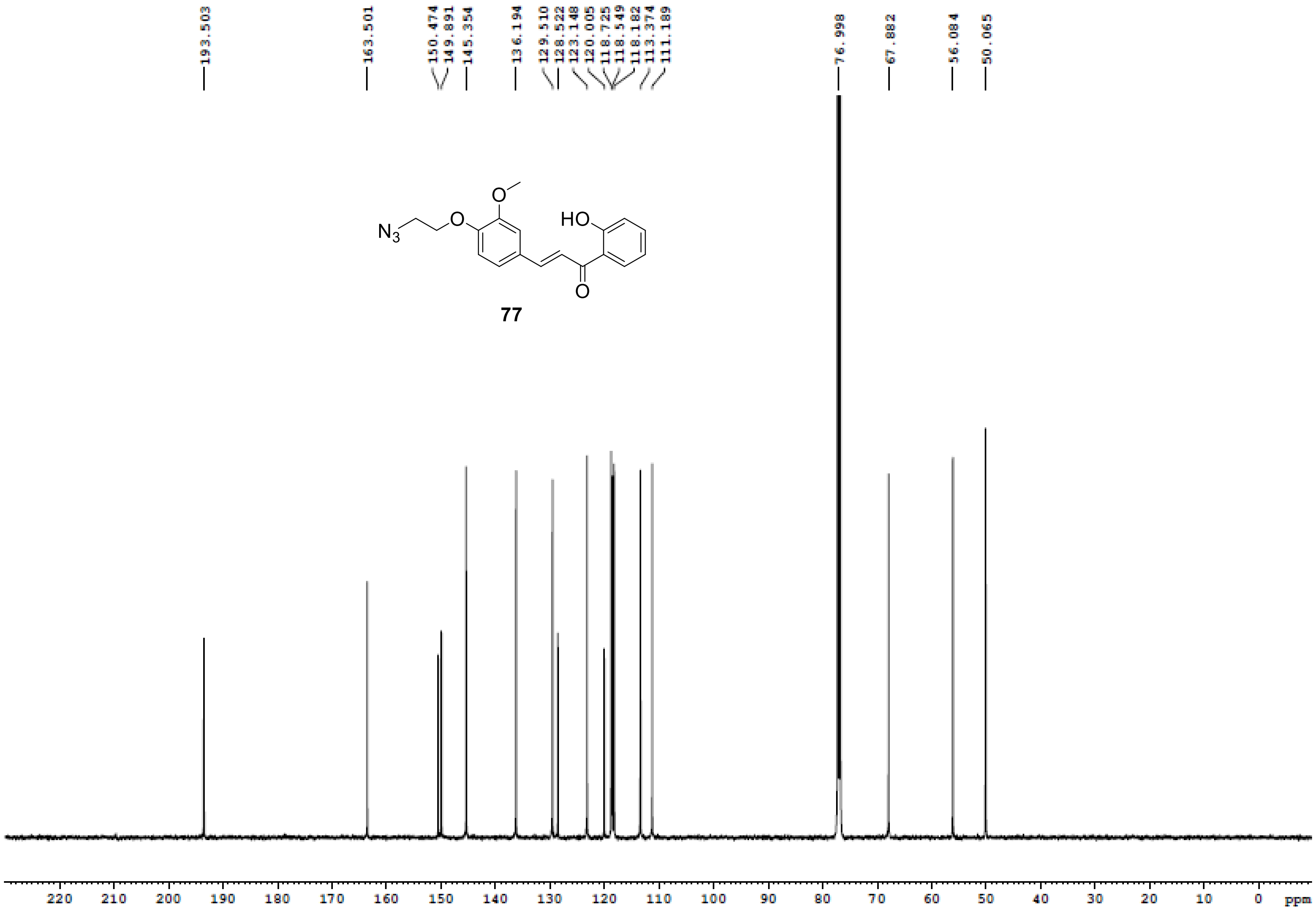
193.503
163.501
150.474
149.891
145.354
136.194
129.510
128.522
123.148
120.005
118.725
118.549
118.182
113.374
111.189
76.998
67.882
56.084
50.065
N3
O
O
HO
O
77
220 210 200 190 180 170 160 150 140 130 120 110 100 90 80 70 60 50 40 30 20 10 0 ppm

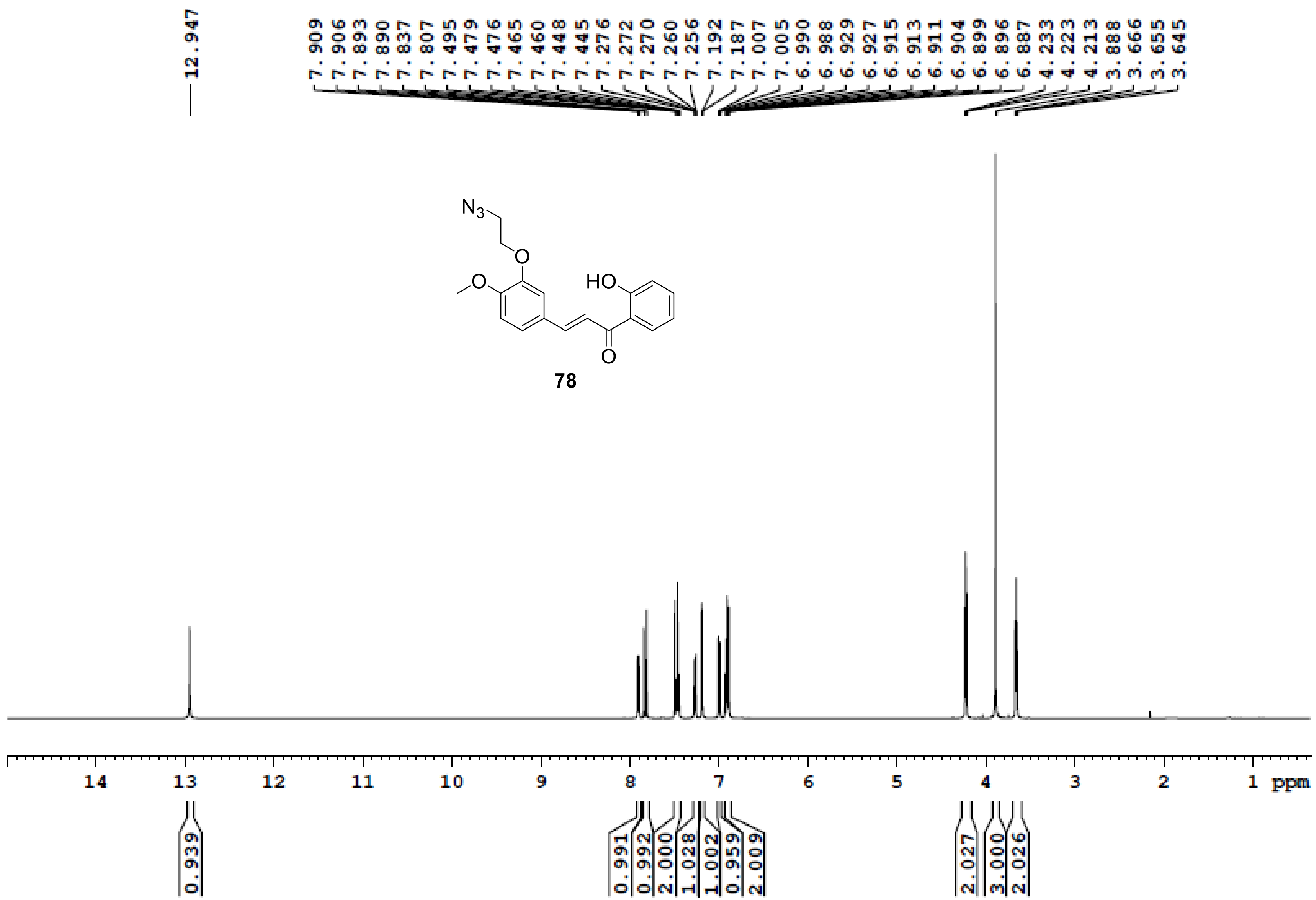
12.947
7.909 7.906 7.893 7.890 7.837 7.807 7.495 7.479 7.476 7.465 7.460 7.448 7.445 7.276 7.272 7.270 7.260 7.256 7.192 7.187 7.007 7.005 6.990 6.988 6.929 6.927 6.915 6.913 6.911 6.904 6.899 6.896 6.887 4.233 4.223 4.213 3.888 3.666 3.655 3.645
N3
O
O
HO
O
78
14 13 12 11 10 9 8 7 6 5 4 3 2 1 ppm
0.939
0.991 0.992 2.000 1.028 1.002 0.959 2.009
2.027 3.000 2.026

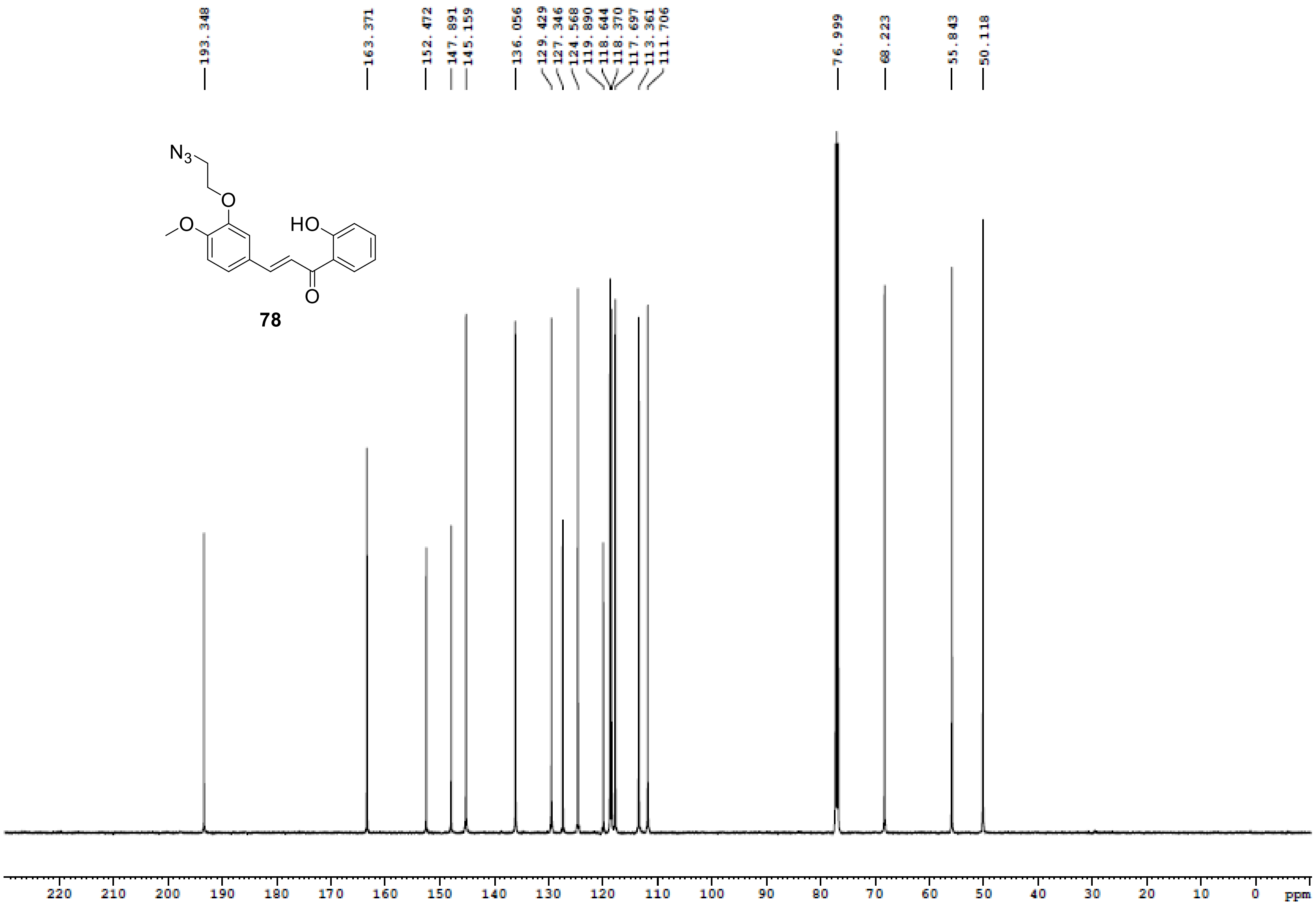
193.348
163.371
152.472
147.891
145.159
136.056
129.429
127.346
124.568
119.890
118.644
118.370
117.697
113.361
111.706
76.999
68.223
55.843
50.118
N3
O
O
HO
O
78
220 210 200 190 180 170 160 150 140 130 120 110 100 90 80 70 60 50 40 30 20 10 0 ppm

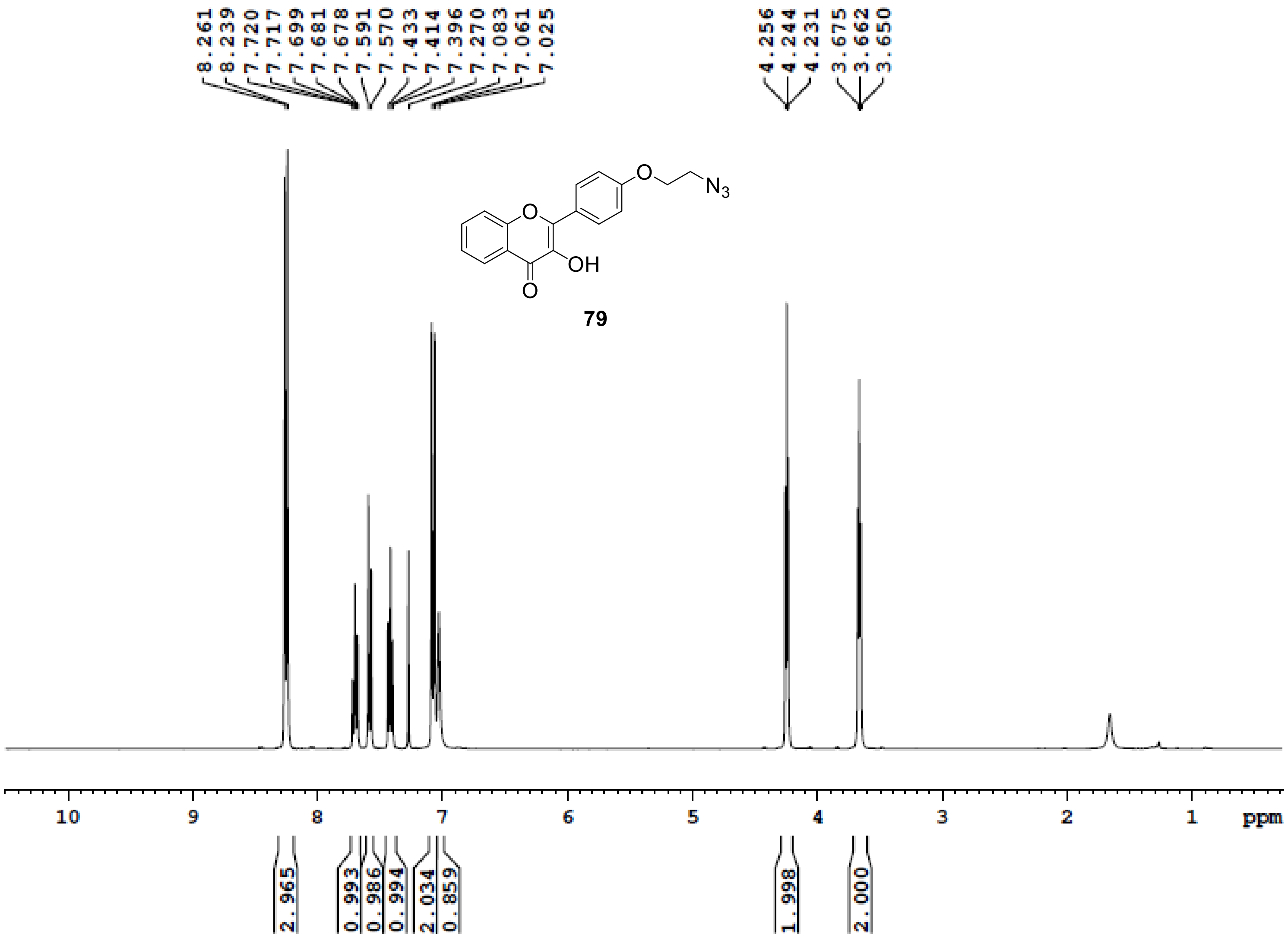
8.261
8.239
7.720
7.717
7.699
7.681
7.678
7.591
7.570
7.433
7.414
7.396
7.270
7.083
7.061
7.025
4.256
4.244
4.231
3.675
3.662
3.650
O
N3
O
OH
O
79
10
9
8
7
6
5
4
3
2
1
ppm
2.965
0.993
0.986
0.994
2.034
0.859
1.998
2.000

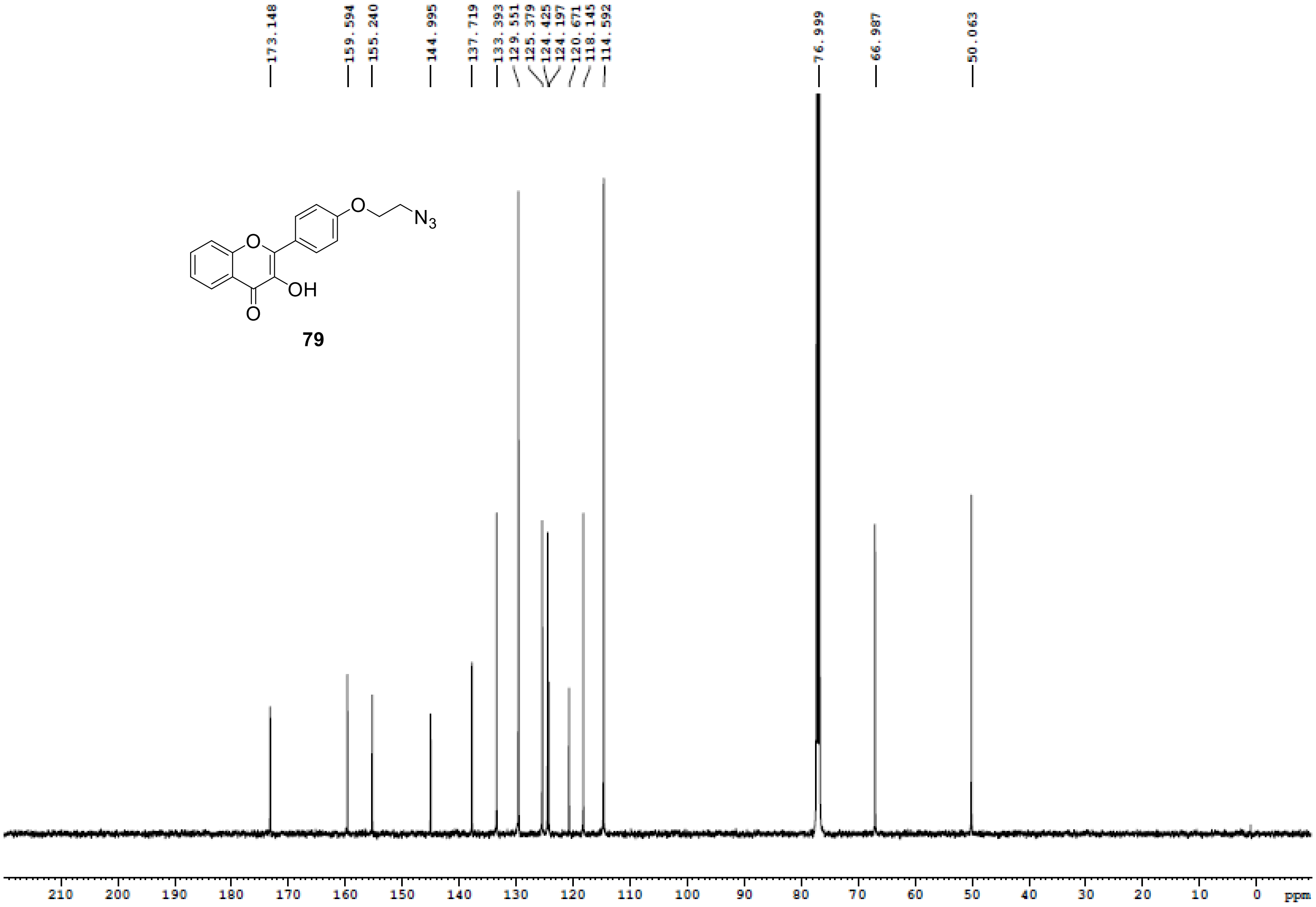
173.148
159.594
155.240
144.995
137.719
133.393
129.551
125.379
124.425
124.197
120.671
118.145
114.592
76.999
66.987
50.063
O
N3
O
OH
O
79
210
200
190
180
170
160
150
140
130
120
110
100
90
80
70
60
50
40
30
20
10
0
ppm

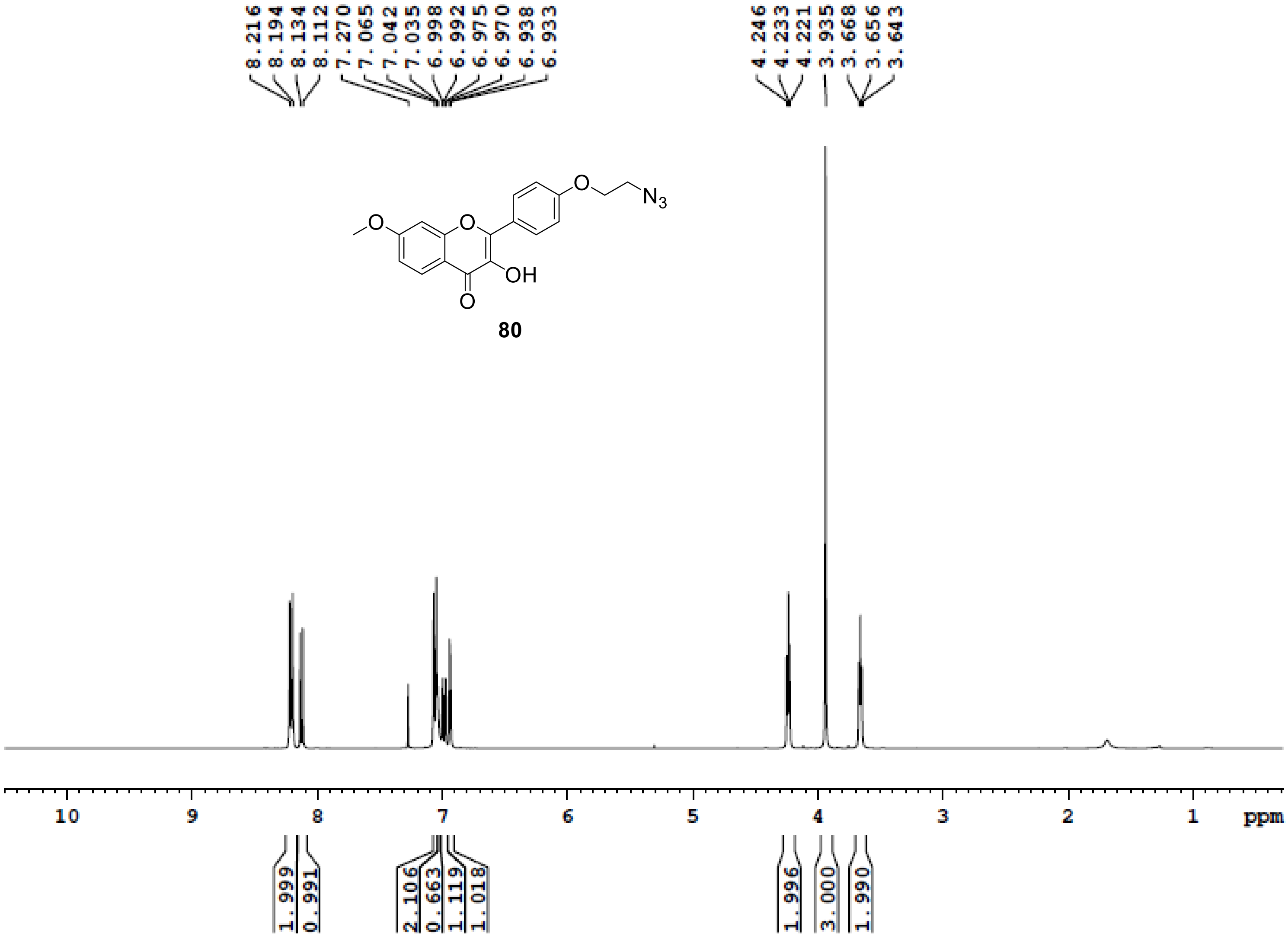
8.216
8.194
8.134
8.112
7.270
7.065
7.042
7.035
6.998
6.992
6.975
6.970
6.938
6.933
4.246
4.233
4.221
3.935
3.668
3.656
3.643
O
O
N3
OH
O
80
10
9
8
7
6
5
4
3
2
1
ppm
1.999
0.991
2.106
0.663
1.119
1.018
1.996
3.000
1.990

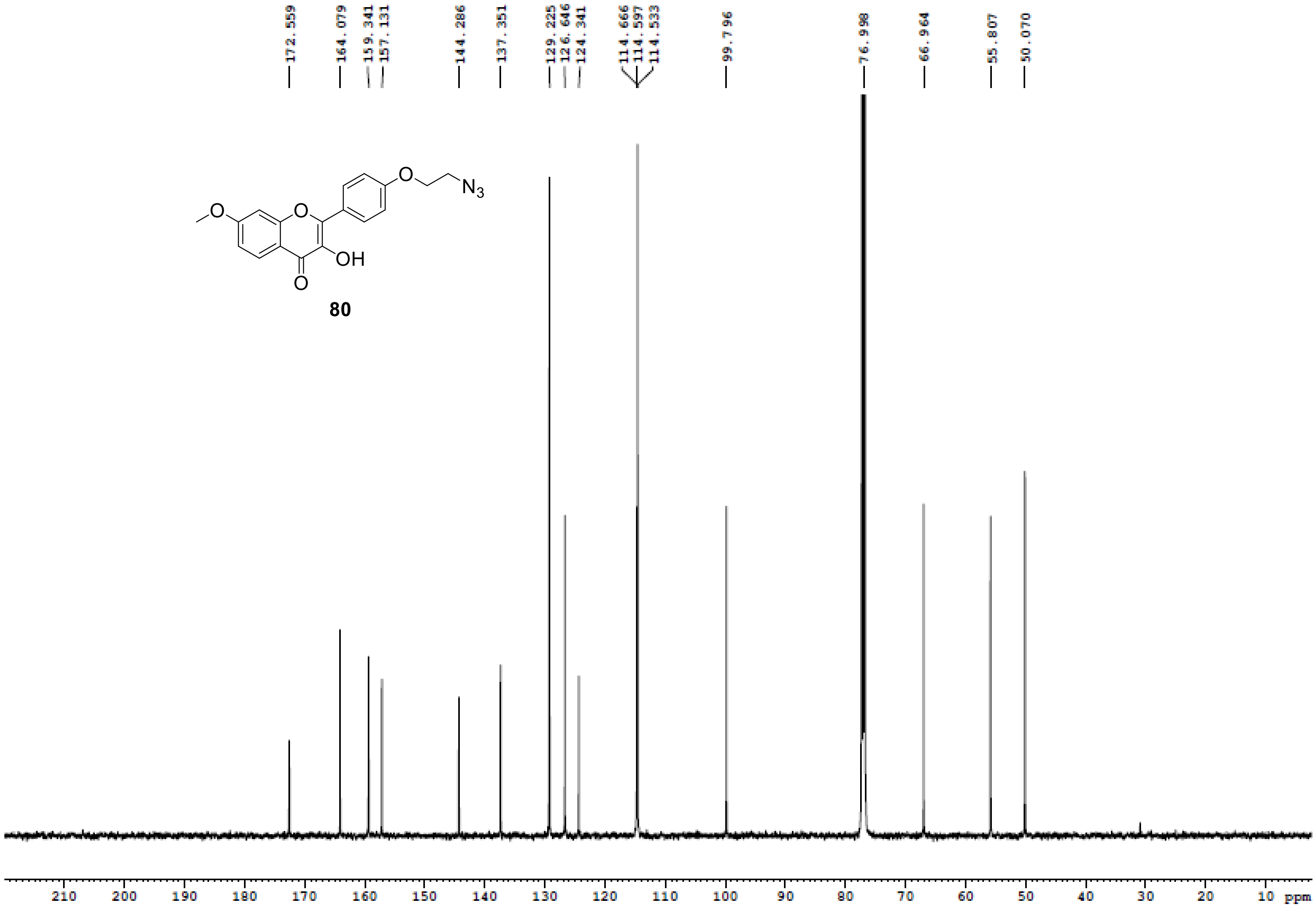
172.559
164.079
159.341
157.131
144.286
137.351
129.225
126.646
124.341
114.666
114.597
114.533
99.796
76.998
66.964
55.807
50.070
O
O
OH
O
O
N3
210 200 190 180 170 160 150 140 130 120 110 100 90 80 70 60 50 40 30 20 10 ppm

80

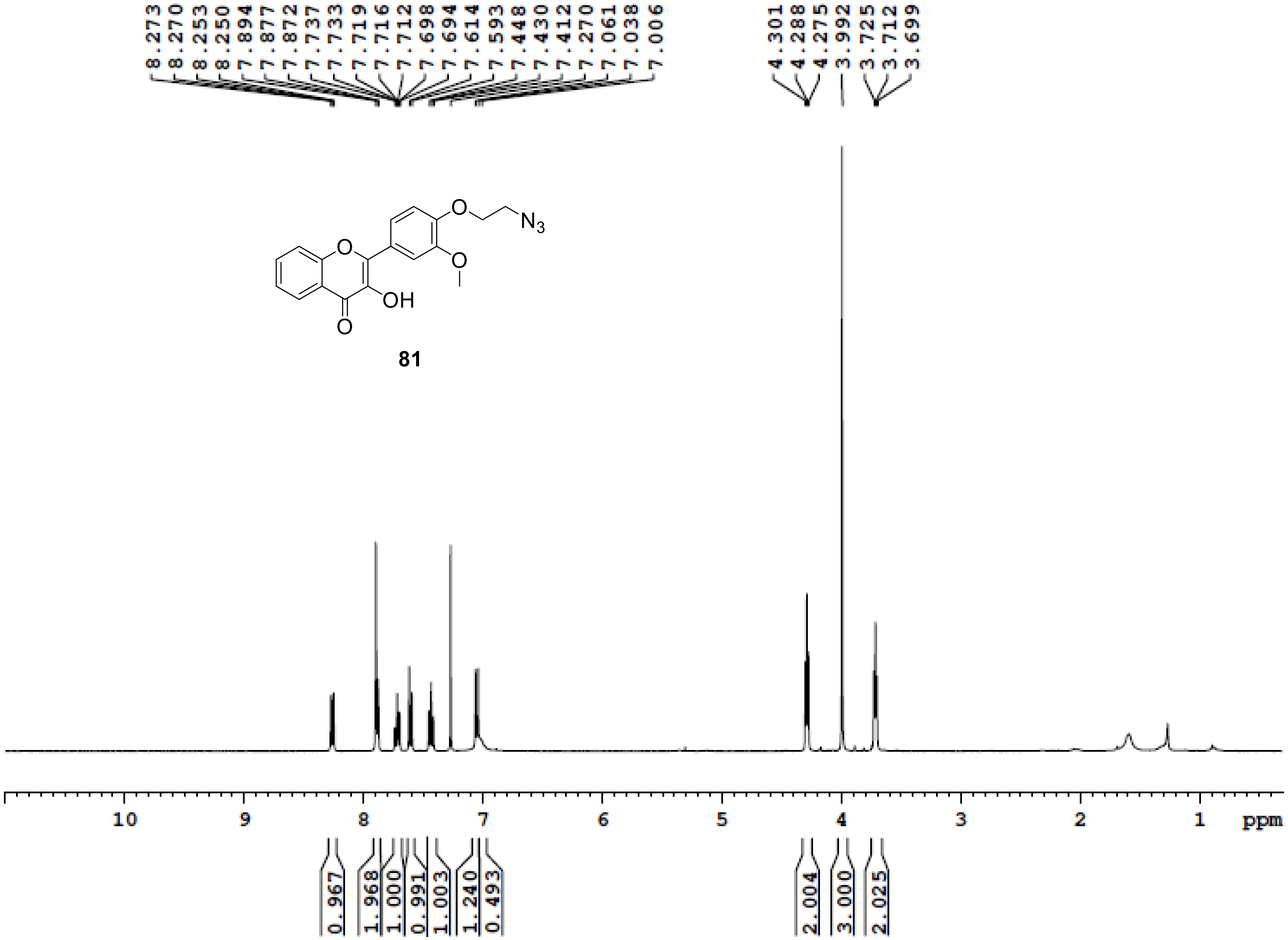
8.273
8.270
8.253
8.250
7.894
7.877
7.872
7.737
7.733
7.719
7.716
7.712
7.698
7.694
7.614
7.593
7.448
7.430
7.412
7.270
7.061
7.038
7.006
4.301
4.288
4.275
3.992
3.725
3.712
3.699
O
N3
O
O
OH
O
81
10
9
8
7
6
5
4
3
2
1
ppm
0.967
1.968
1.000
0.991
1.003
1.240
0.493
2.004
3.000
2.025

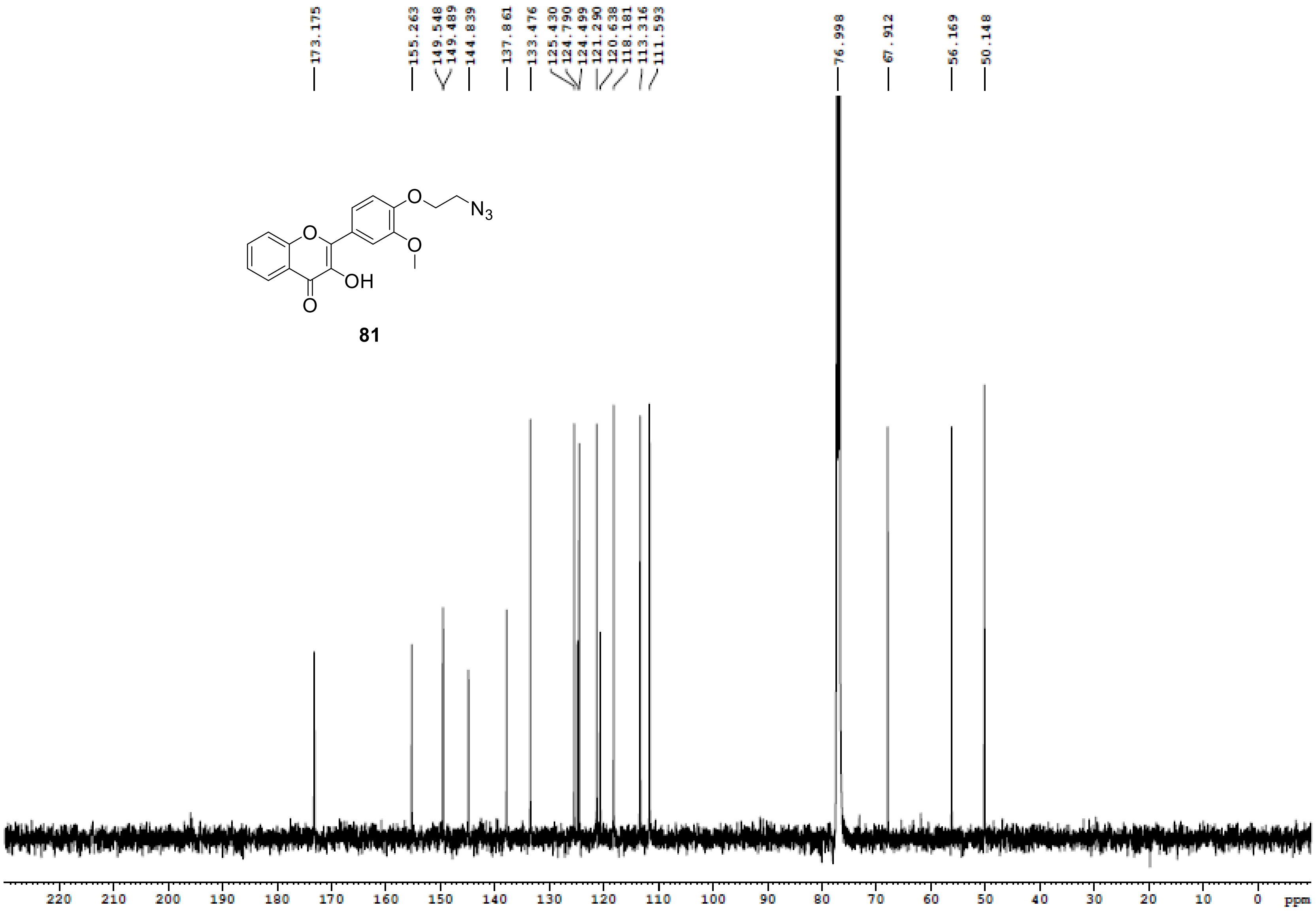
173.175
155.263
149.548
149.489
144.839
137.861
133.476
125.430
124.790
124.499
121.290
120.638
118.181
113.316
111.593
76.998
67.912
56.169
50.148
O
OH
O
O
O
N3
81
220 210 200 190 180 170 160 150 140 130 120 110 100 90 80 70 60 50 40 30 20 10 0 ppm

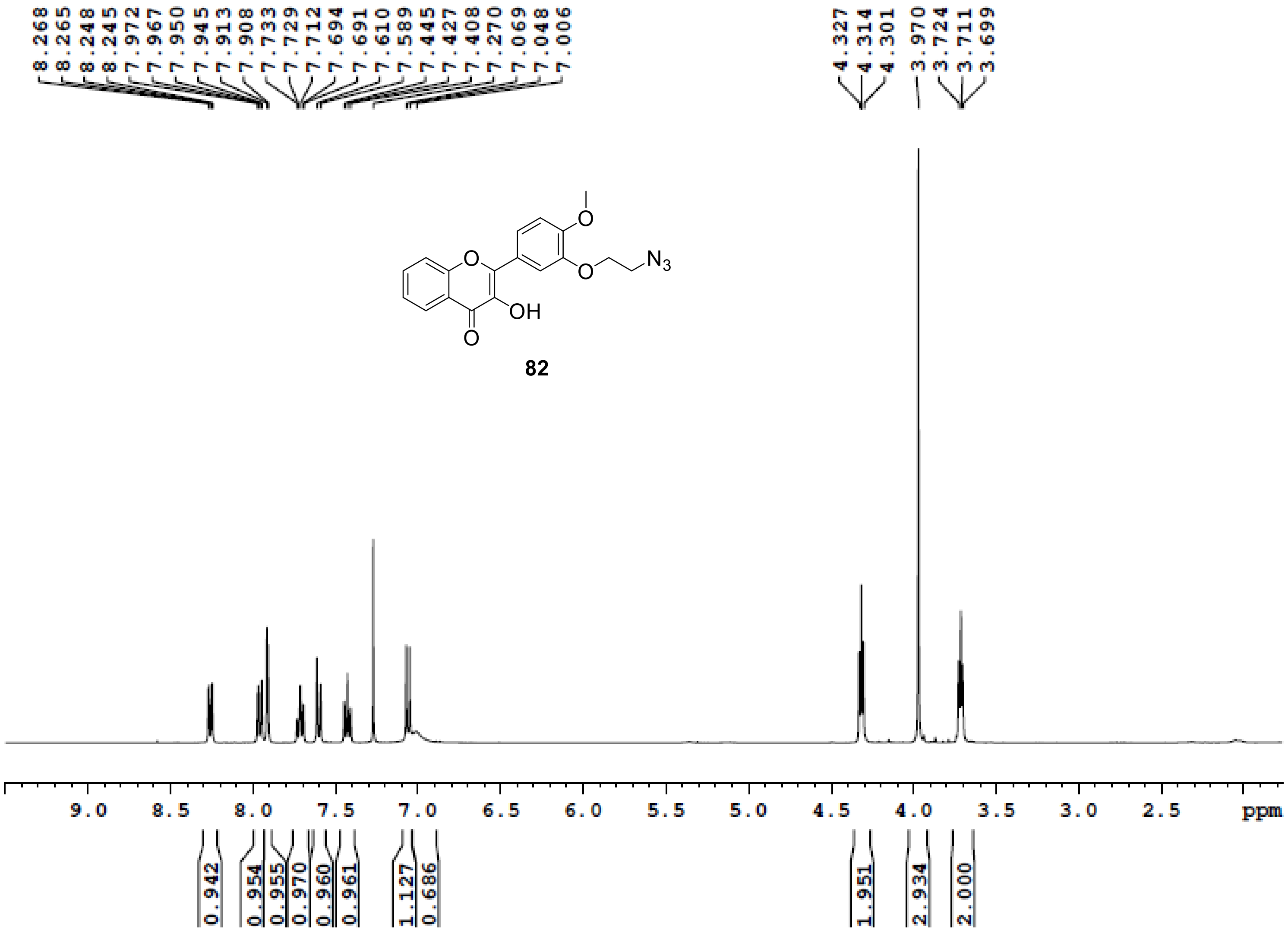
8.268 8.265 8.248 8.245 7.972 7.967 7.950 7.945 7.913 7.908 7.733 7.729 7.712 7.694 7.691 7.610 7.589 7.445 7.427 7.408 7.270 7.069 7.048 7.006
4.327 4.314 4.301 3.970 3.724 3.711 3.699
O
N3
OH
9.0 8.5 8.0 7.5 7.0 6.5 6.0 5.5 5.0 4.5 4.0 3.5 3.0 2.5 ppm
0.942 0.954 0.955 0.970 0.960 0.961 1.127 0.686 1.951 2.934 2.000

82

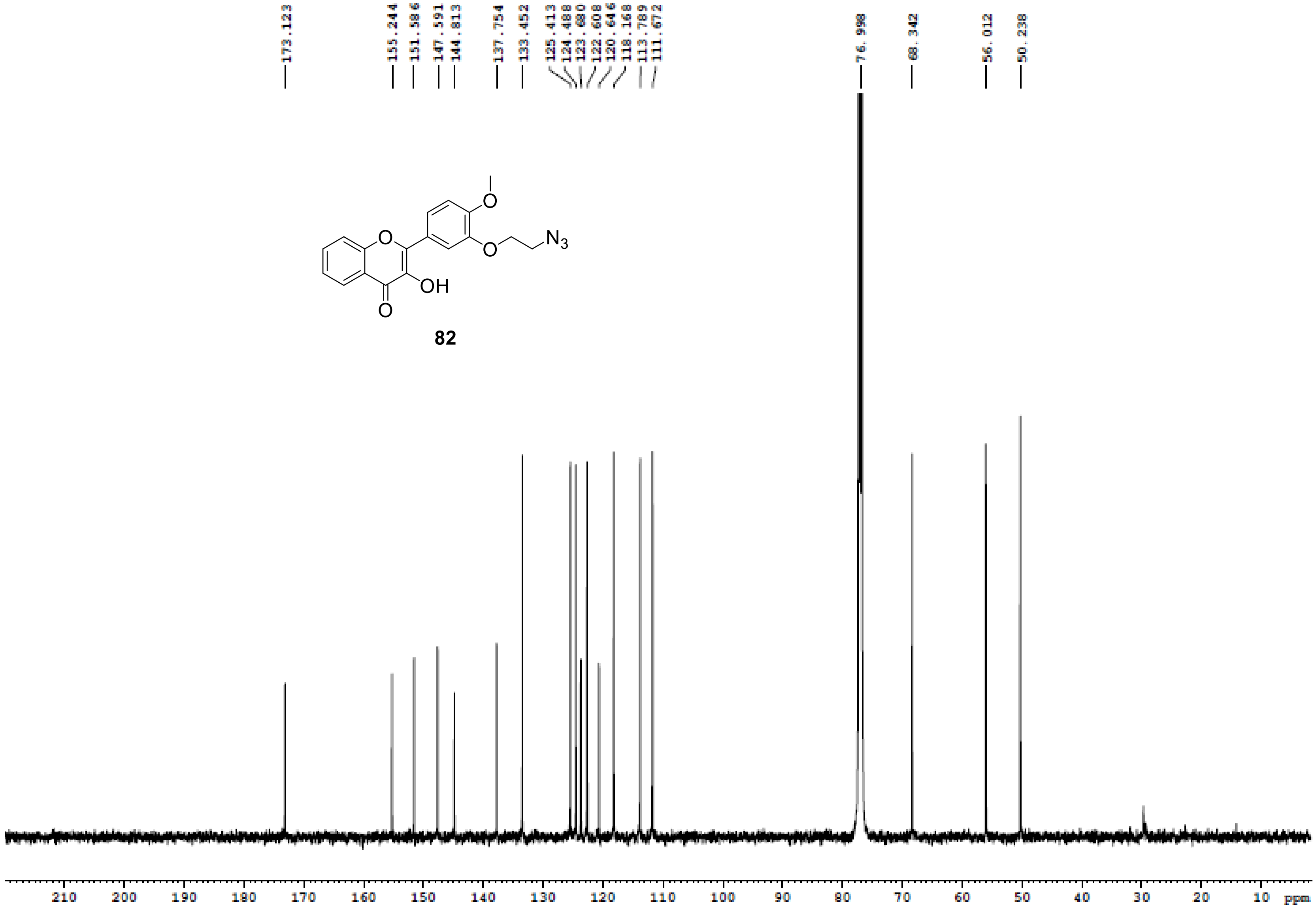
173.123
155.244
151.586
147.591
144.813
137.754
133.452
125.413
124.488
123.680
122.608
120.646
118.168
113.789
111.672
76.998
68.342
56.012
50.238
O
O
N3
O
OH
O
82
210
200
190
180
170
160
150
140
130
120
110
100
90
80
70
60
50
40
30
20
10
ppm

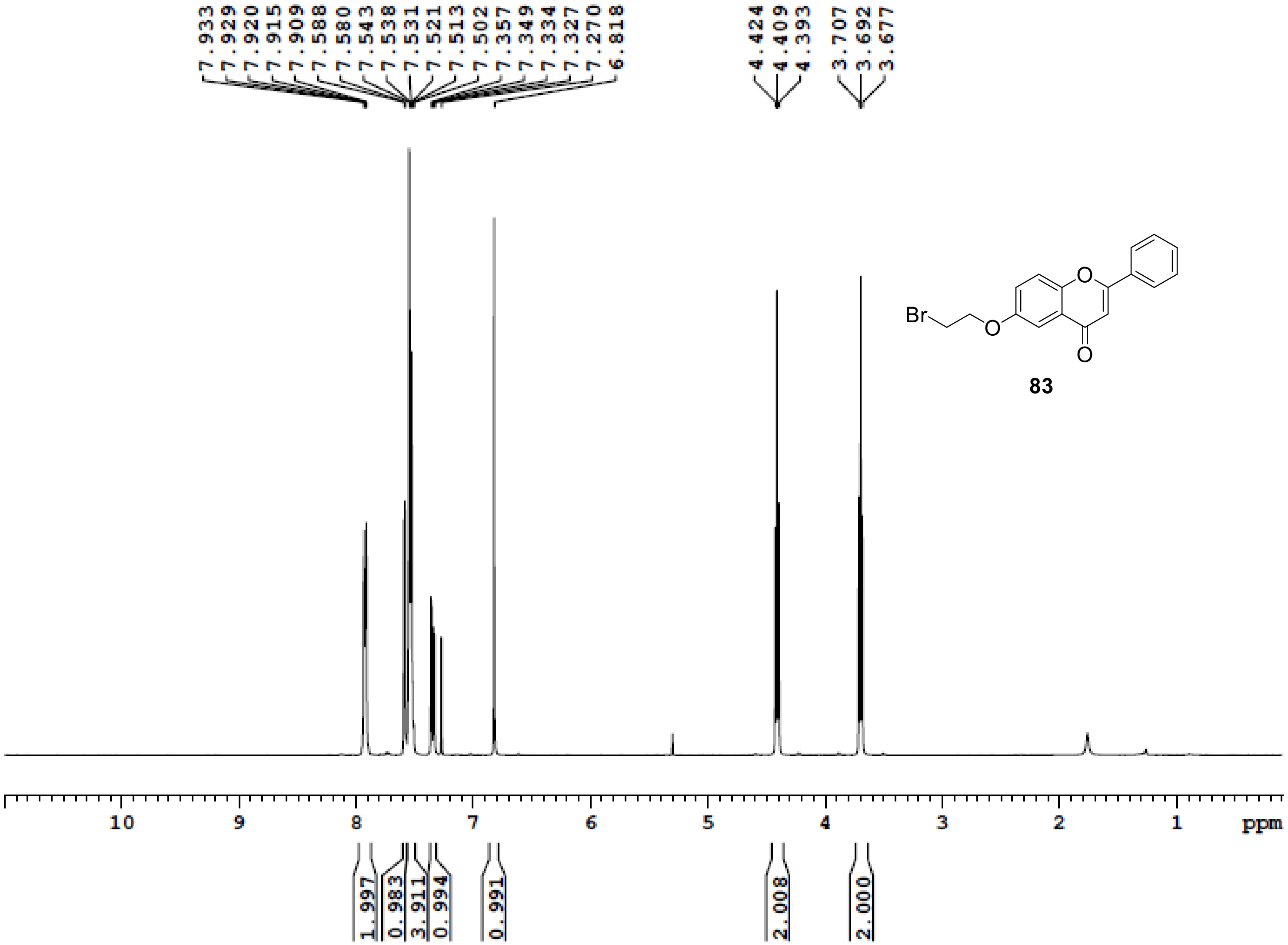
7.933
7.929
7.920
7.915
7.909
7.588
7.580
7.543
7.538
7.531
7.521
7.513
7.502
7.357
7.349
7.334
7.327
7.270
6.818
4.424
4.409
4.393
3.707
3.692
3.677
Br
O
O
O
83
10
9
8
7
6
5
4
3
2
1
ppm
1.997
0.983
3.911
0.994
0.991
2.008
2.000

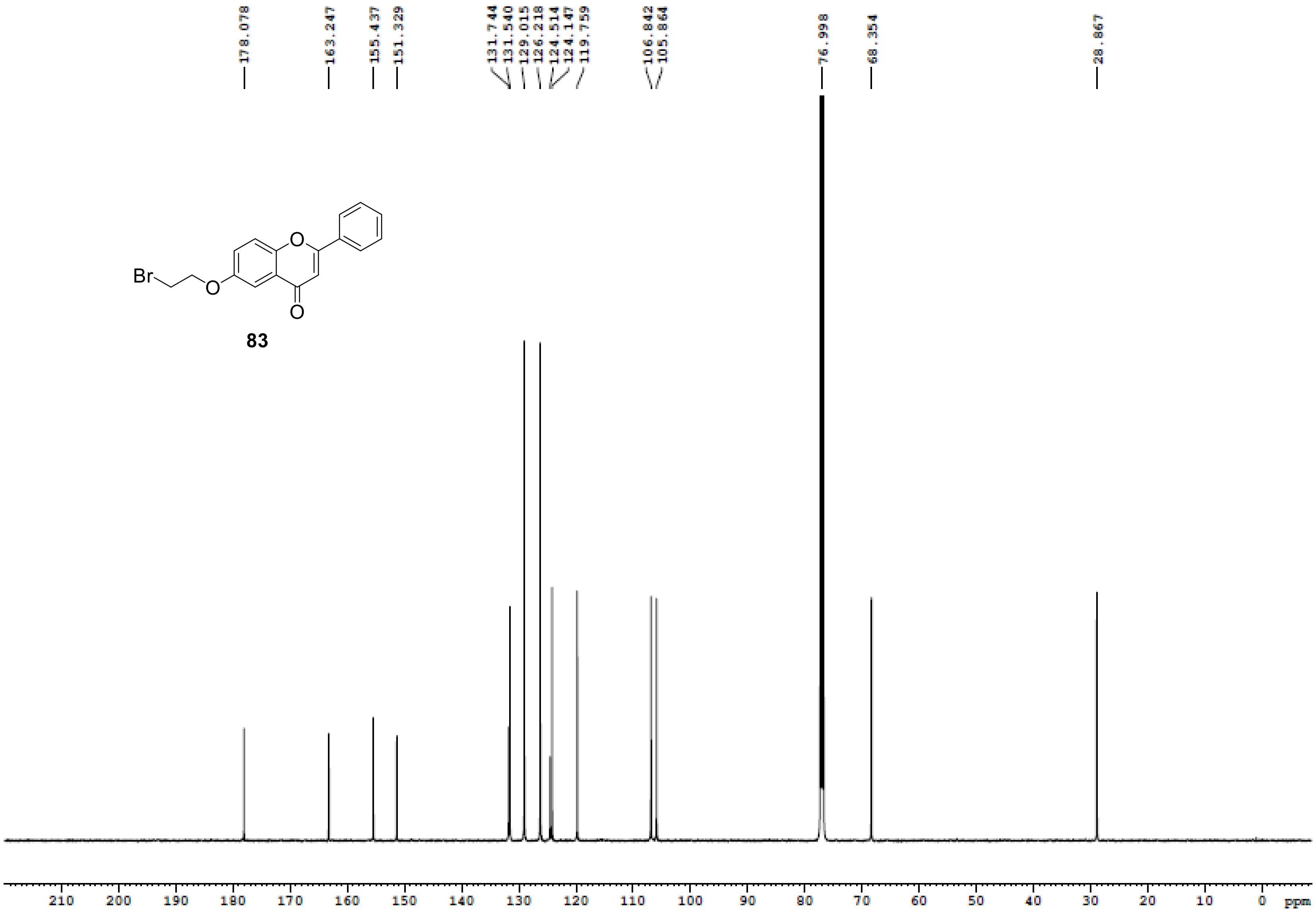
178.078
163.247
155.437
151.329
131.744
131.540
129.015
126.218
124.514
124.147
119.759
106.842
105.864
76.998
68.354
28.867
Br
O
O
O
83
210 200 190 180 170 160 150 140 130 120 110 100 90 80 70 60 50 40 30 20 10 0 ppm

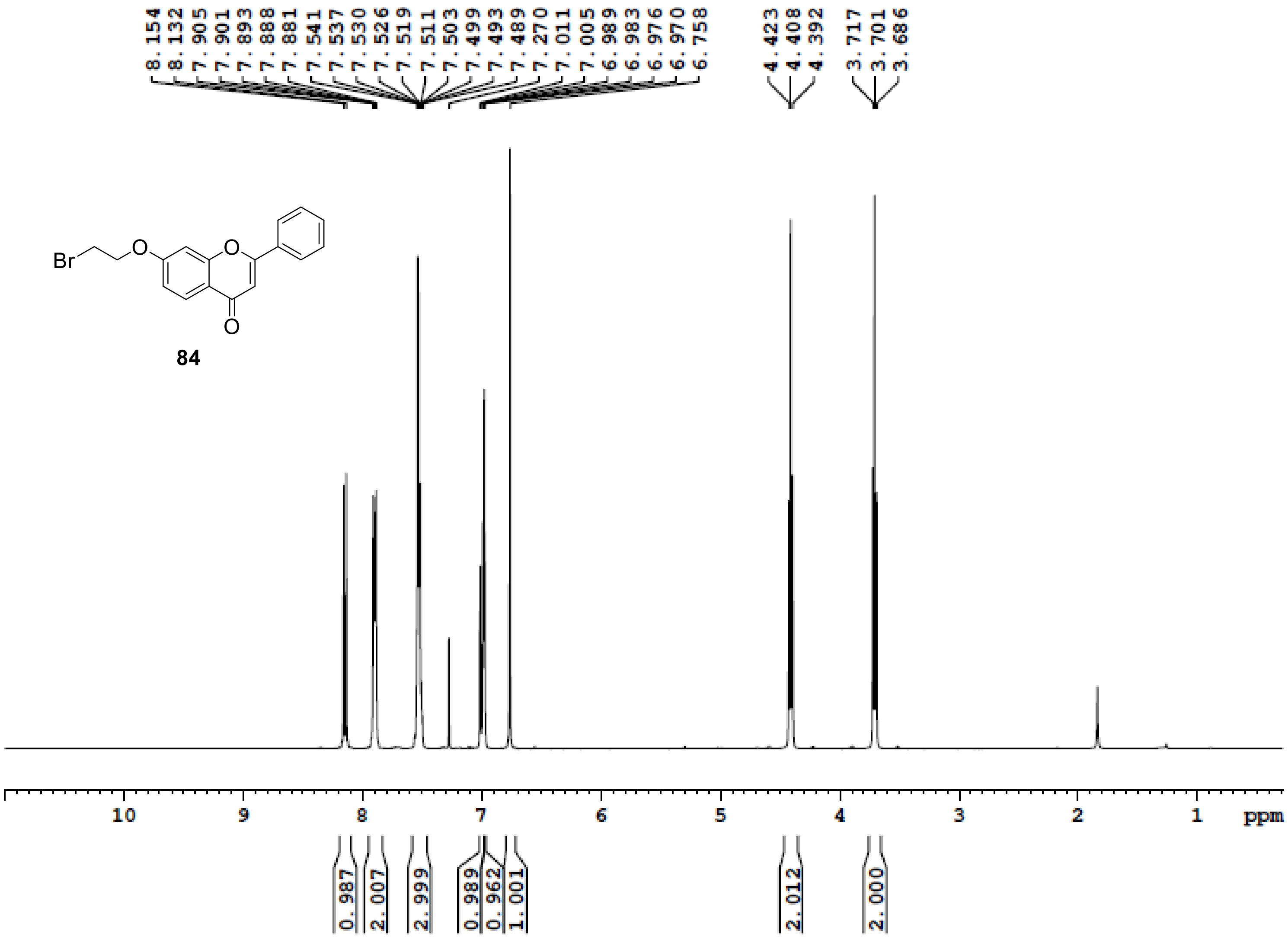
8.154
8.132
7.905
7.901
7.893
7.888
7.881
7.541
7.537
7.530
7.526
7.519
7.511
7.503
7.499
7.493
7.489
7.270
7.011
7.005
6.989
6.983
6.976
6.970
6.758
4.423
4.408
4.392
3.717
3.701
3.686
Br
O
O
O
84
10
9
8
7
6
5
4
3
2
1
ppm
0.987
2.007
2.999
0.989
0.962
1.001
2.012
2.000

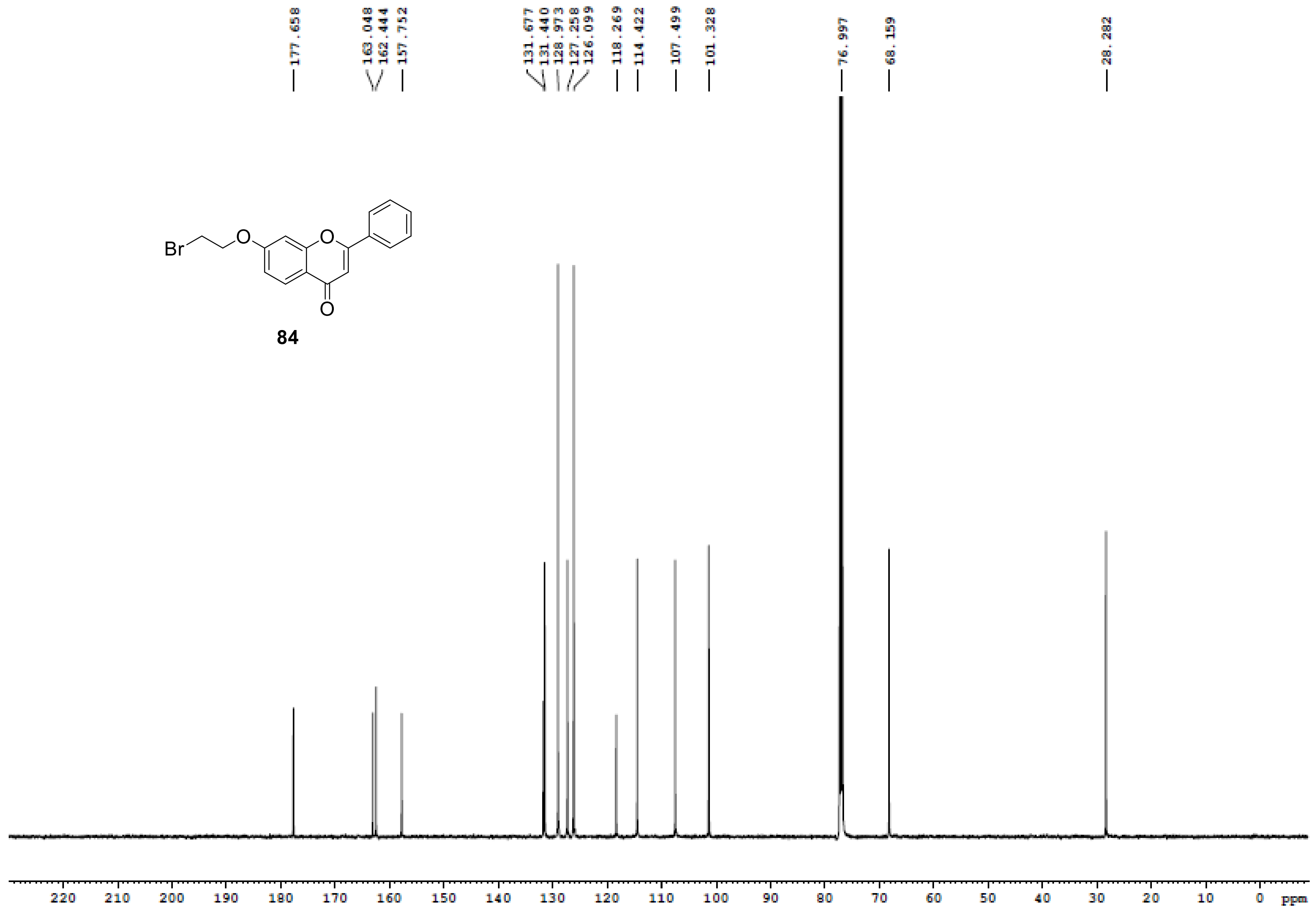
177.658
163.048
162.444
157.752
131.677
131.440
128.973
127.258
126.099
118.269
114.422
107.499
101.328
76.997
68.159
28.282
Br
O
O
O
84
220 210 200 190 180 170 160 150 140 130 120 110 100 90 80 70 60 50 40 30 20 10 0 ppm

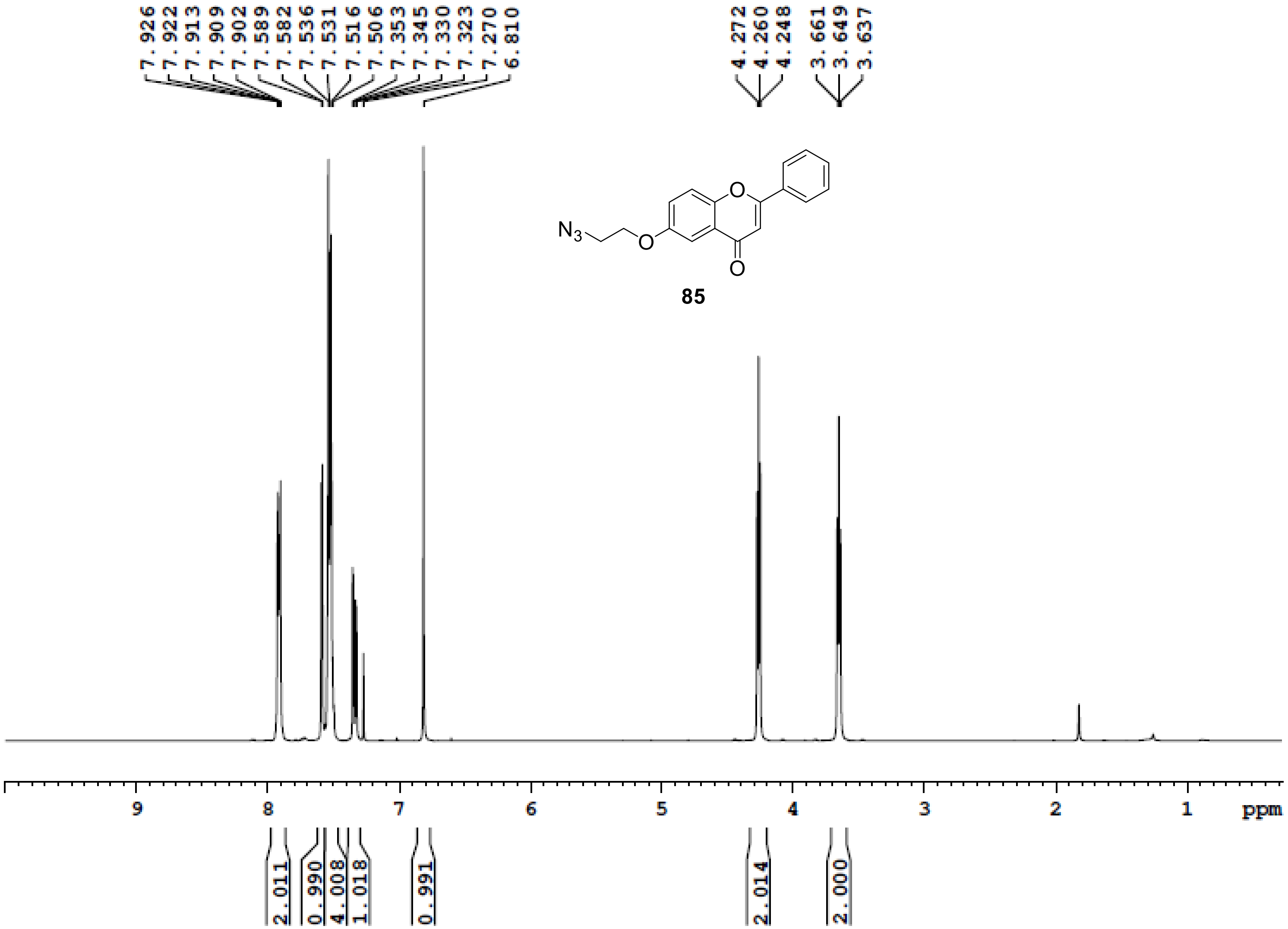
7.926
7.922
7.913
7.909
7.902
7.589
7.582
7.536
7.531
7.516
7.506
7.353
7.345
7.330
7.323
7.270
6.810
4.272
4.260
4.248
3.661
3.649
3.637
N3
O
O
O
85
9
8
7
6
5
4
3
2
1
ppm
2.011
0.990
4.008
1.018
0.991
2.014
2.000

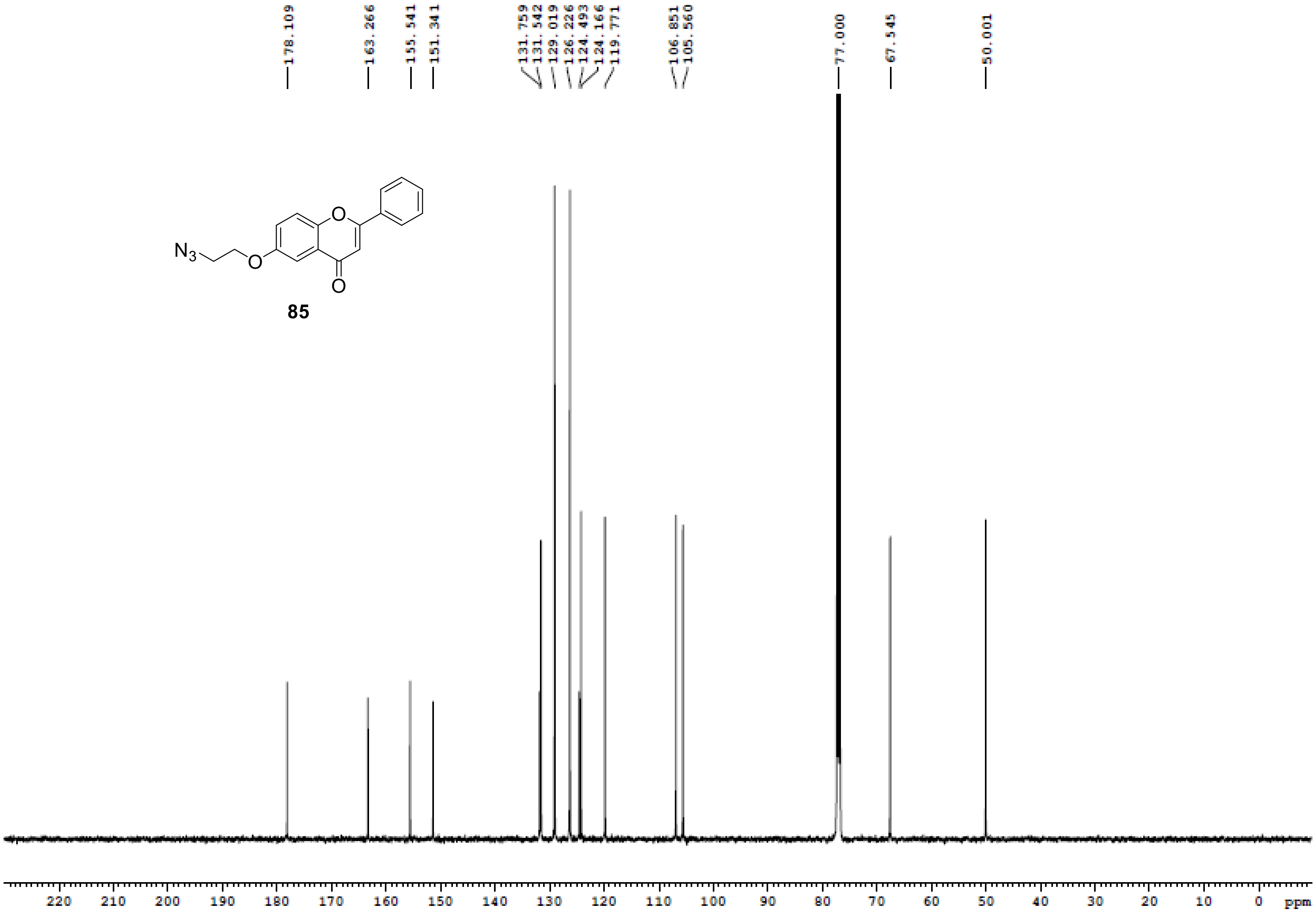
178.109
163.266
155.541
151.341
131.759
131.542
129.019
126.226
124.493
124.166
119.771
106.851
105.560
77.000
67.545
50.001
N3
O
O
O
85
220
210
200
190
180
170
160
150
140
130
120
110
100
90
80
70
60
50
40
30
20
10
0
ppm

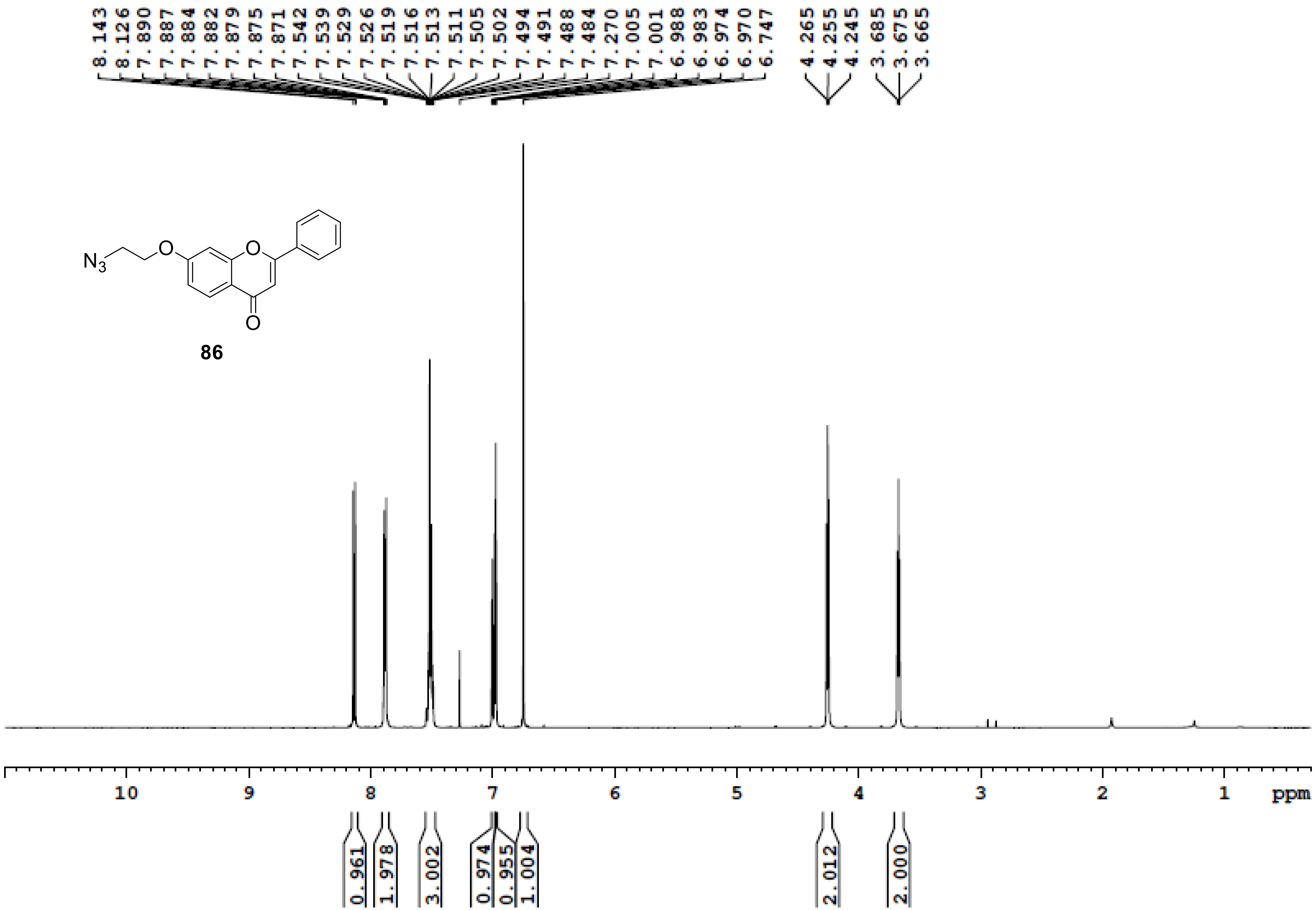
8.143 8.126 7.890 7.887 7.884 7.882 7.879 7.875 7.871 7.542 7.539 7.529 7.526 7.519 7.516 7.513 7.511 7.505 7.502 7.494 7.491 7.488 7.484 7.270 7.005 7.001 6.988 6.983 6.974 6.970 6.747
4.265 4.255 4.245 3.685 3.675 3.665
N3
O
O
O
86
10 9 8 7 6 5 4 3 2 1 ppm
0.961 1.978 3.002 0.974 0.955 1.004 2.012 2.000

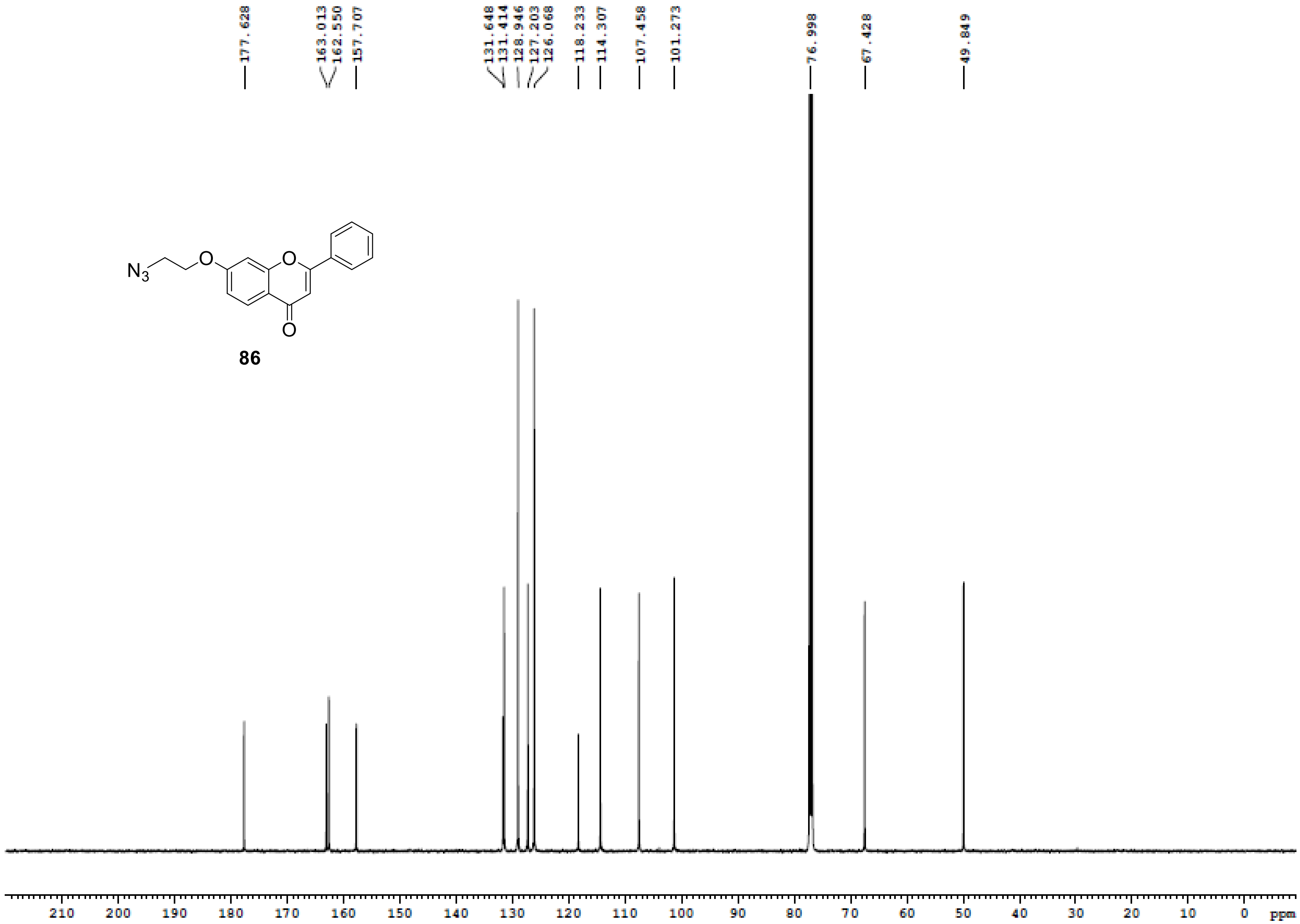
177.628
163.013
162.550
157.707
131.648
131.414
128.946
127.203
126.068
118.233
114.307
107.458
101.273
76.998
67.428
49.849
N3
O
O
O
86
210 200 190 180 170 160 150 140 130 120 110 100 90 80 70 60 50 40 30 20 10 0 ppm

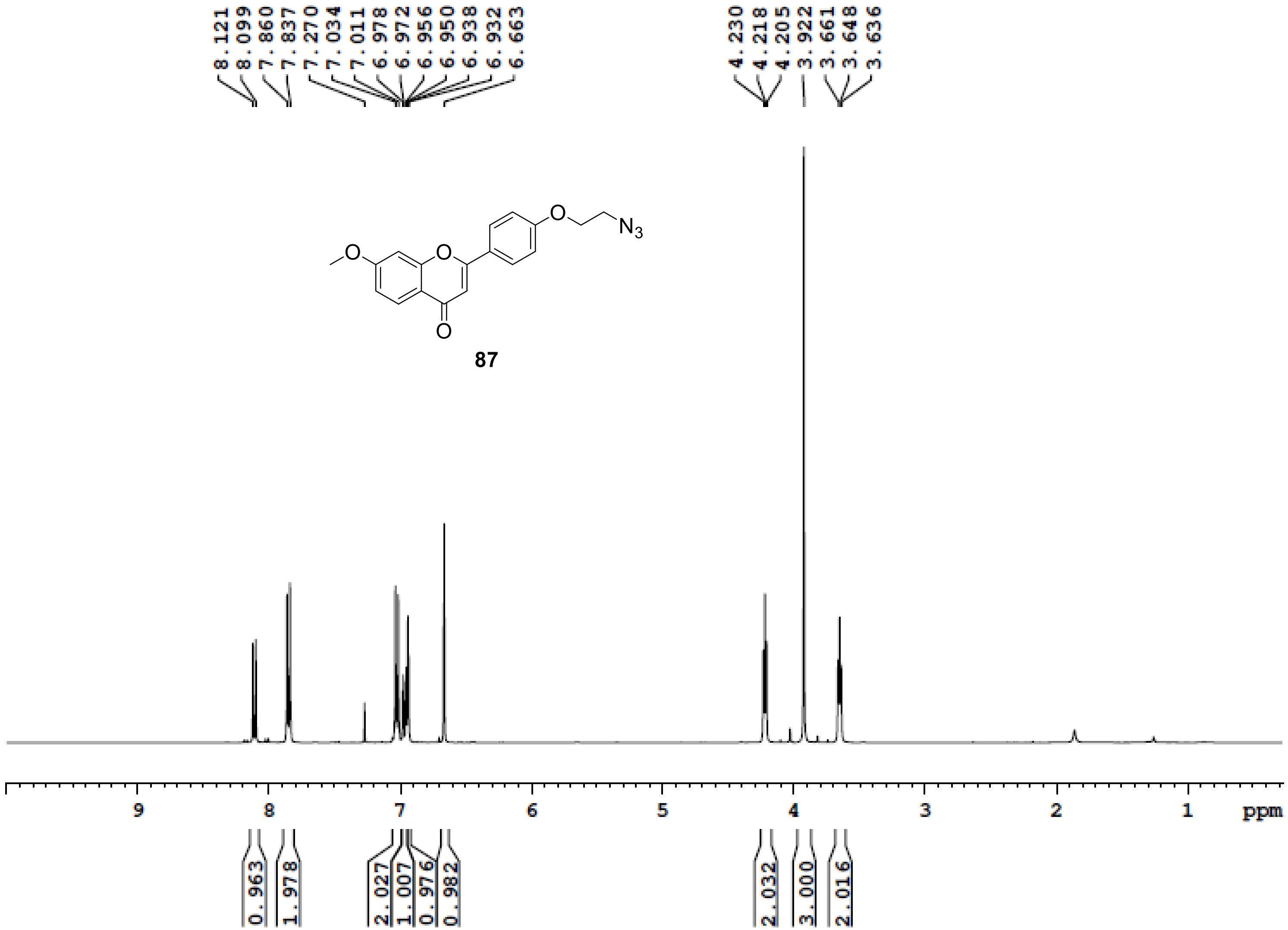
8.121 8.099 7.860 7.837 7.270 7.034 7.011 6.978 6.972 6.956 6.950 6.938 6.932 6.663
4.230 4.218 4.205 3.922 3.661 3.648 3.636
O
O
O
N3
O
87
9 8 7 6 5 4 3 2 1 ppm
0.963 1.978 2.027 1.007 0.976 0.982 2.032 3.000 2.016

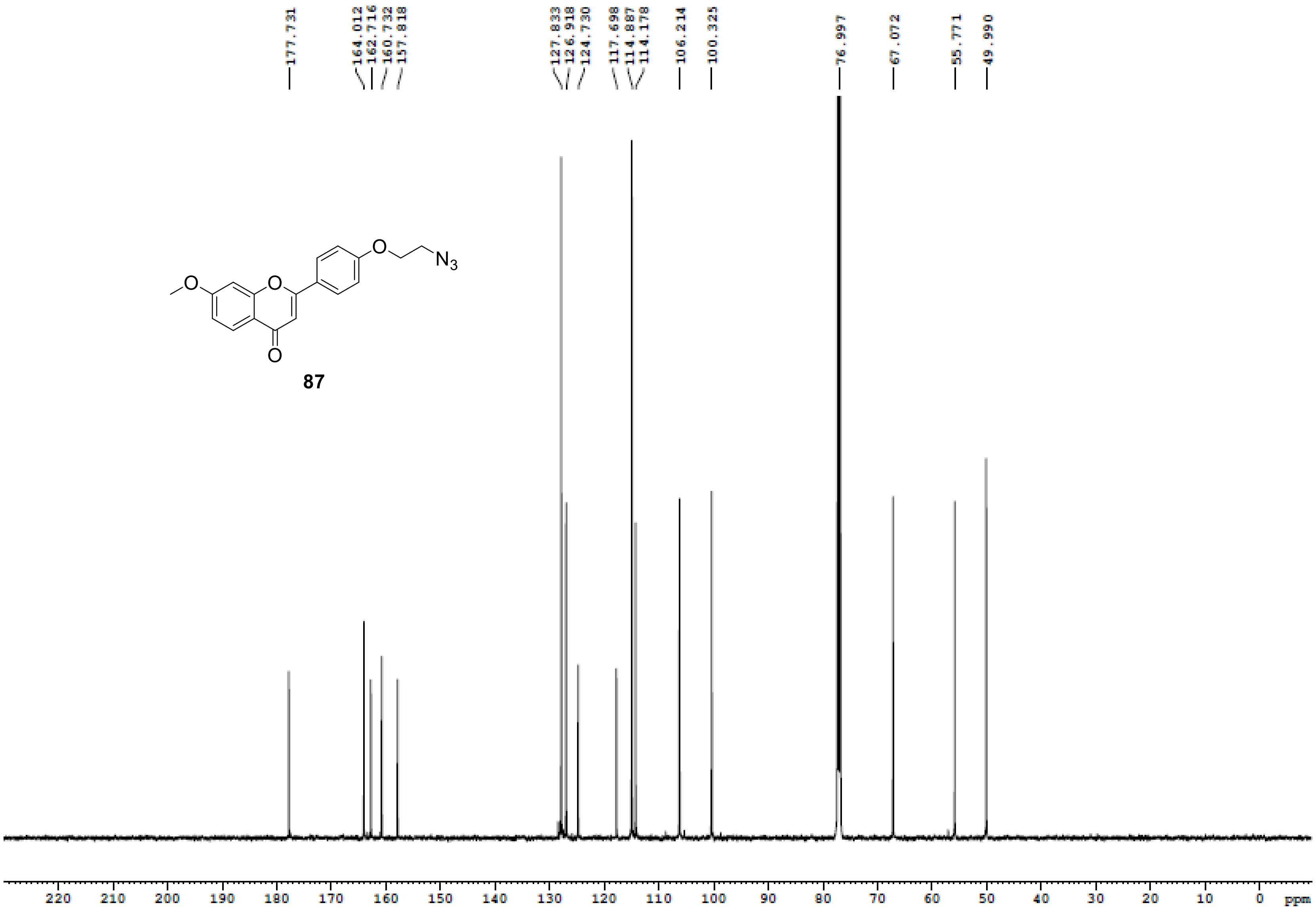
177.731
164.012
162.716
160.732
157.818
127.833
126.918
124.730
117.698
114.887
114.178
106.214
100.325
76.997
67.072
55.771
49.990
87
220 210 200 190 180 170 160 150 140 130 120 110 100 90 80 70 60 50 40 30 20 10 0 ppm

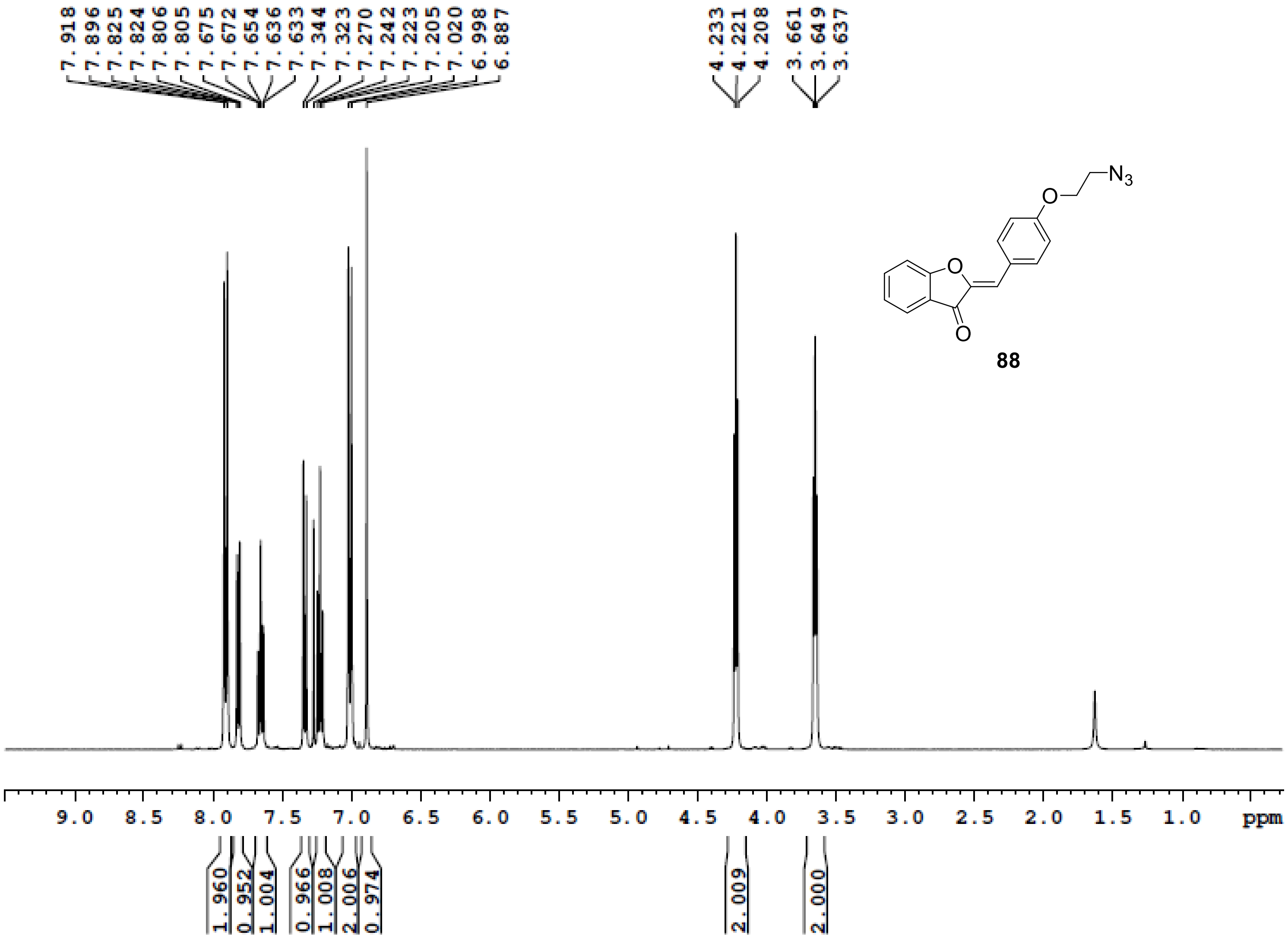
7.918
7.896
7.825
7.824
7.806
7.805
7.675
7.672
7.654
7.636
7.633
7.344
7.323
7.270
7.242
7.223
7.205
7.020
6.998
6.887
4.233
4.221
4.208
3.661
3.649
3.637
O
N3
O
O
88
9.0 8.5 8.0 7.5 7.0 6.5 6.0 5.5 5.0 4.5 4.0 3.5 3.0 2.5 2.0 1.5 1.0 ppm
1.960
0.952
1.004
0.966
1.008
2.006
0.974
2.009
2.000

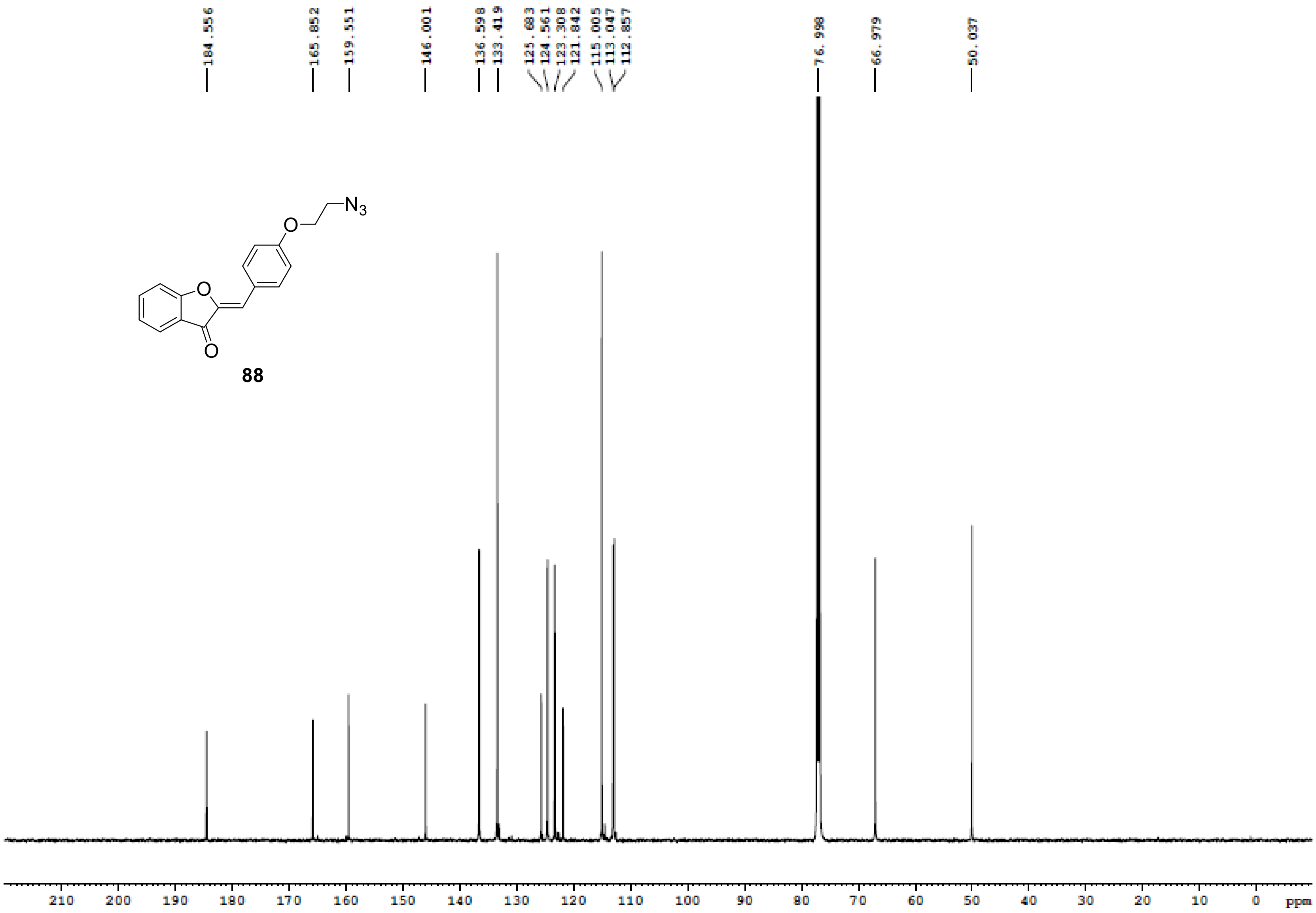
184.556
165.852
159.551
146.001
136.598
133.419
125.683
124.561
123.308
121.842
115.005
113.047
112.857
76.998
66.979
50.037
O
N3
O
O
88
210 200 190 180 170 160 150 140 130 120 110 100 90 80 70 60 50 40 30 20 10 0 ppm

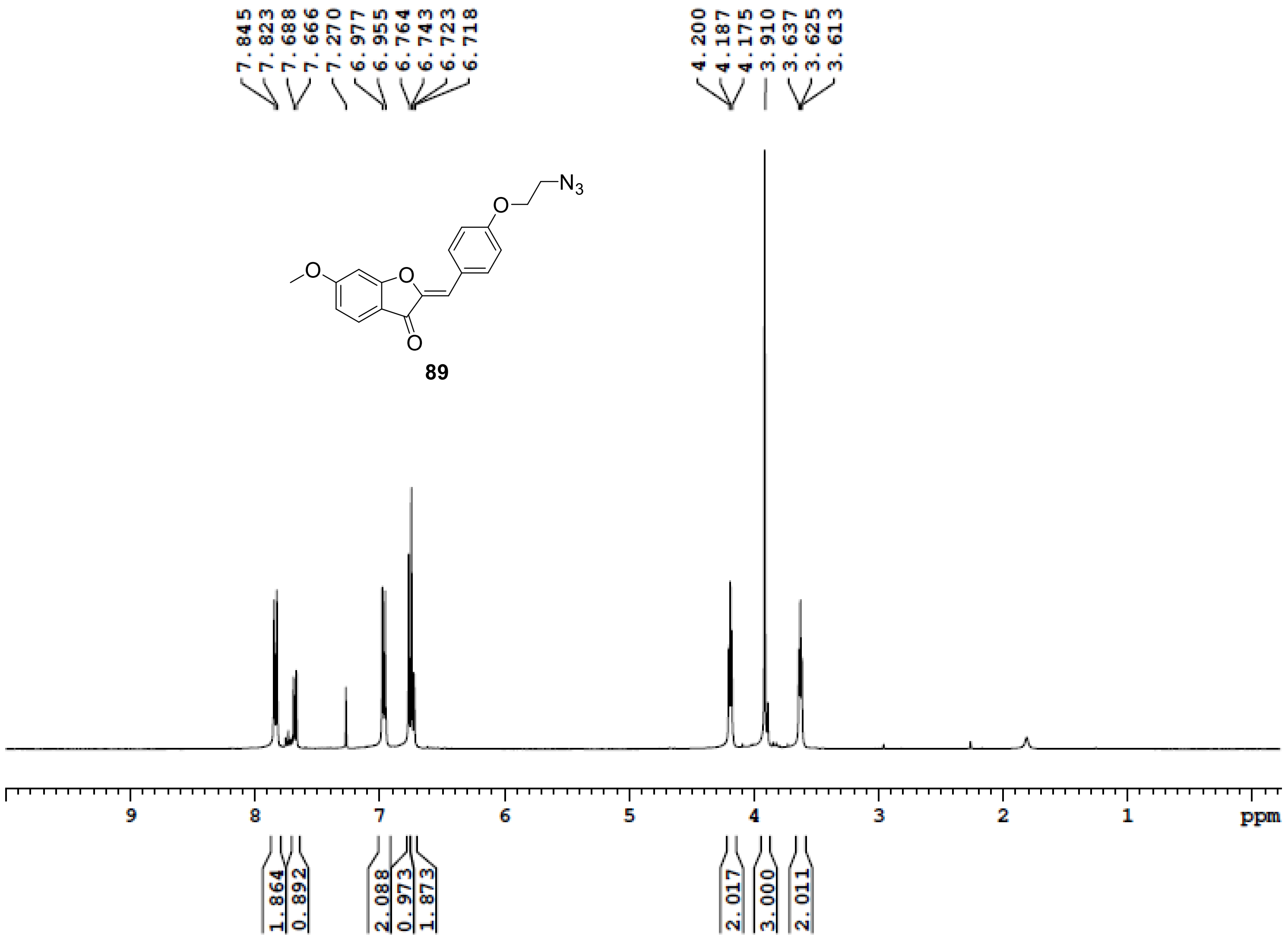
7.845
7.823
7.688
7.666
7.270
6.977
6.955
6.764
6.743
6.723
6.718
4.200
4.187
4.175
3.910
3.637
3.625
3.613
N3
O
O
O
O
89
9
8
7
6
5
4
3
2
1
ppm
1.864
0.892
2.088
0.973
1.873
2.017
3.000
2.011

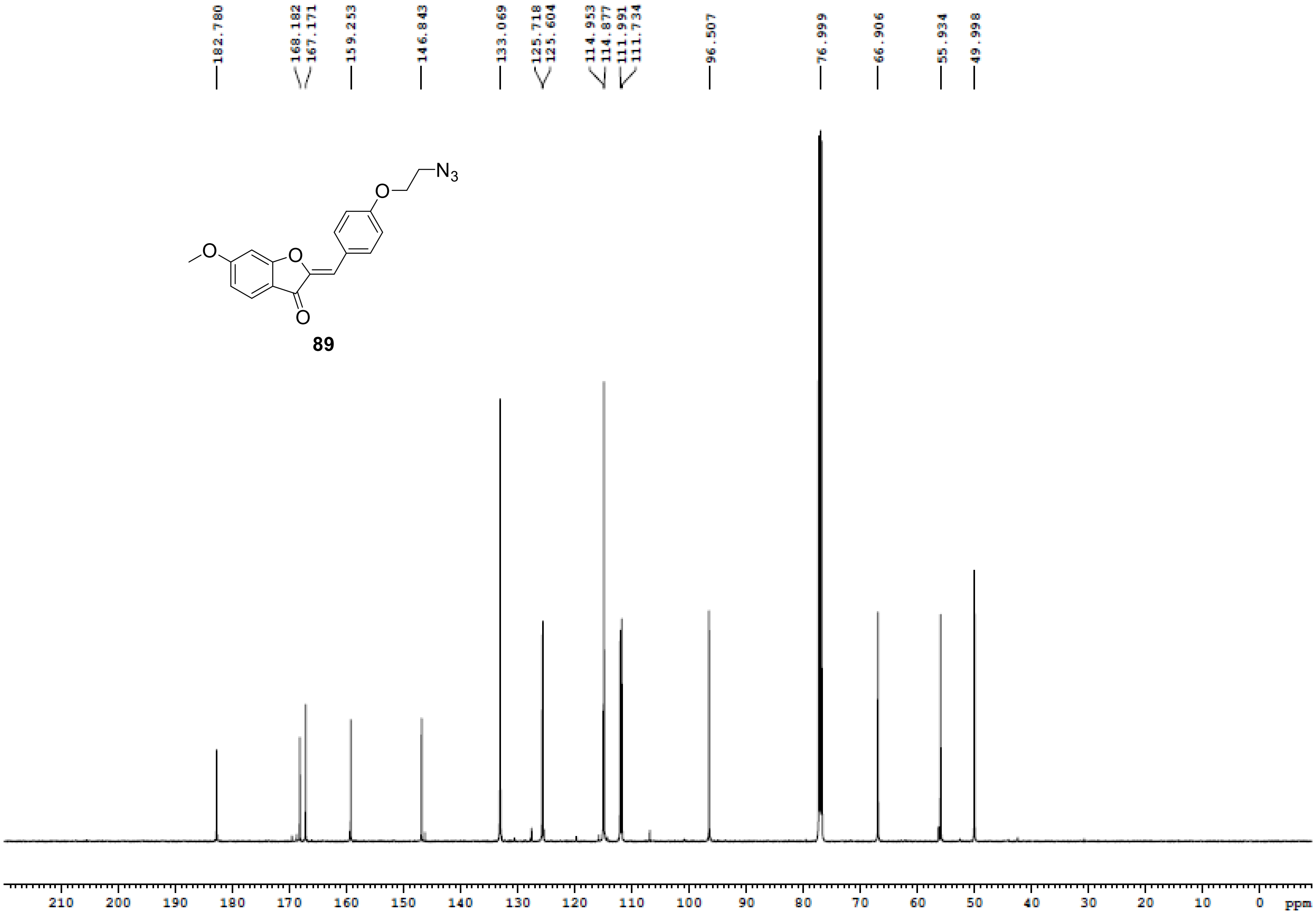
182.780
168.182
167.171
159.253
146.843
133.069
125.718
125.604
114.953
114.877
111.991
111.734
96.507
76.999
66.906
55.934
49.998
N3
O
89
210
200
190
180
170
160
150
140
130
120
110
100
90
80
70
60
50
40
30
20
10
0
ppm

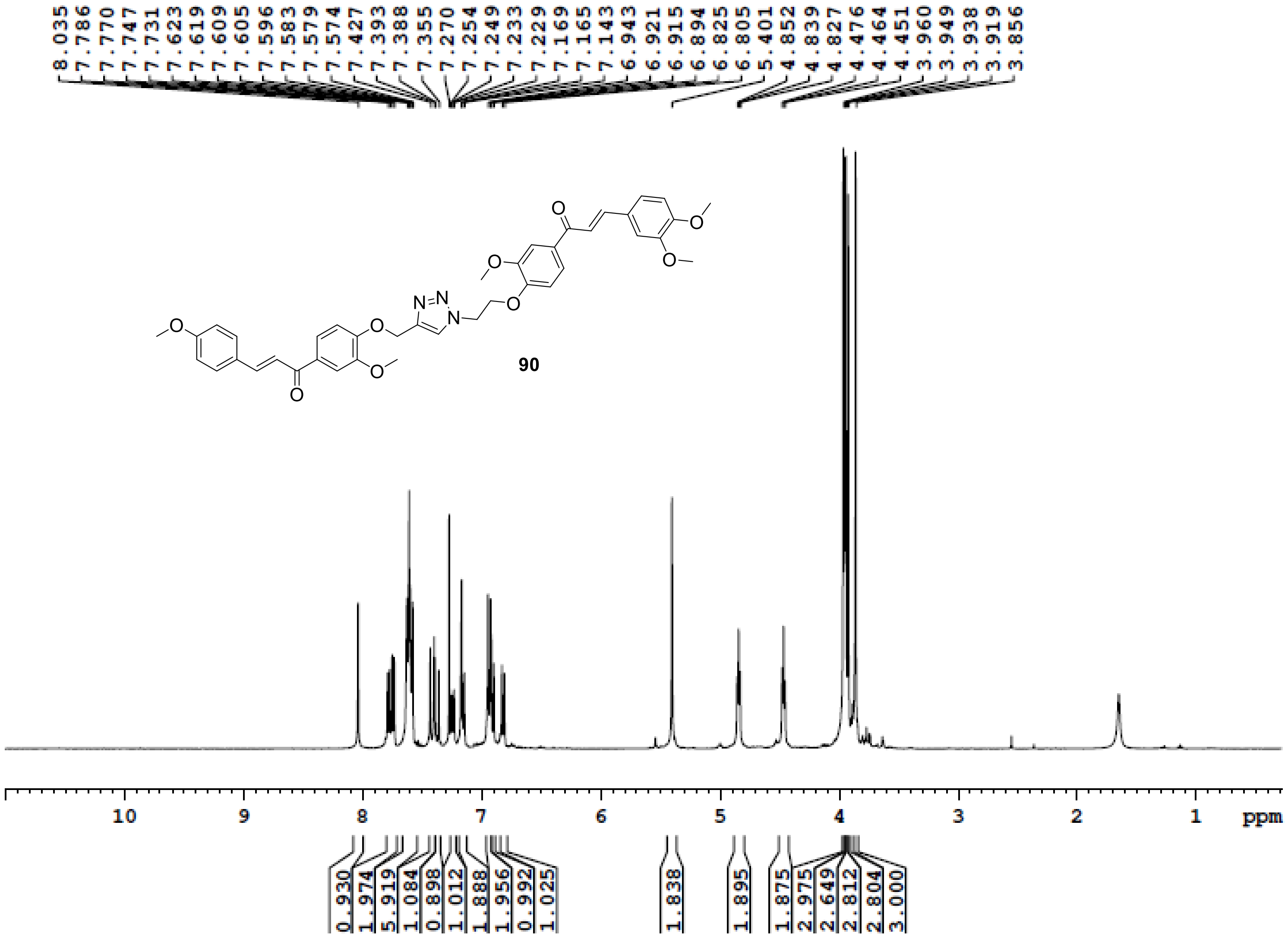
8.035
7.786
7.770
7.747
7.731
7.623
7.619
7.609
7.605
7.596
7.583
7.579
7.574
7.427
7.393
7.388
7.355
7.270
7.254
7.249
7.233
7.229
7.169
7.165
7.143
6.943
6.921
6.915
6.894
6.825
6.805
5.401
4.852
4.839
4.827
4.476
4.464
4.451
3.960
3.949
3.938
3.919
3.856
90
10
9
8
7
6
5
4
3
2
1
ppm
0.930
1.974
5.919
1.084
0.898
1.012
1.888
1.956
0.992
1.025
1.838
1.895
1.875
2.975
2.649
2.812
2.804
3.000

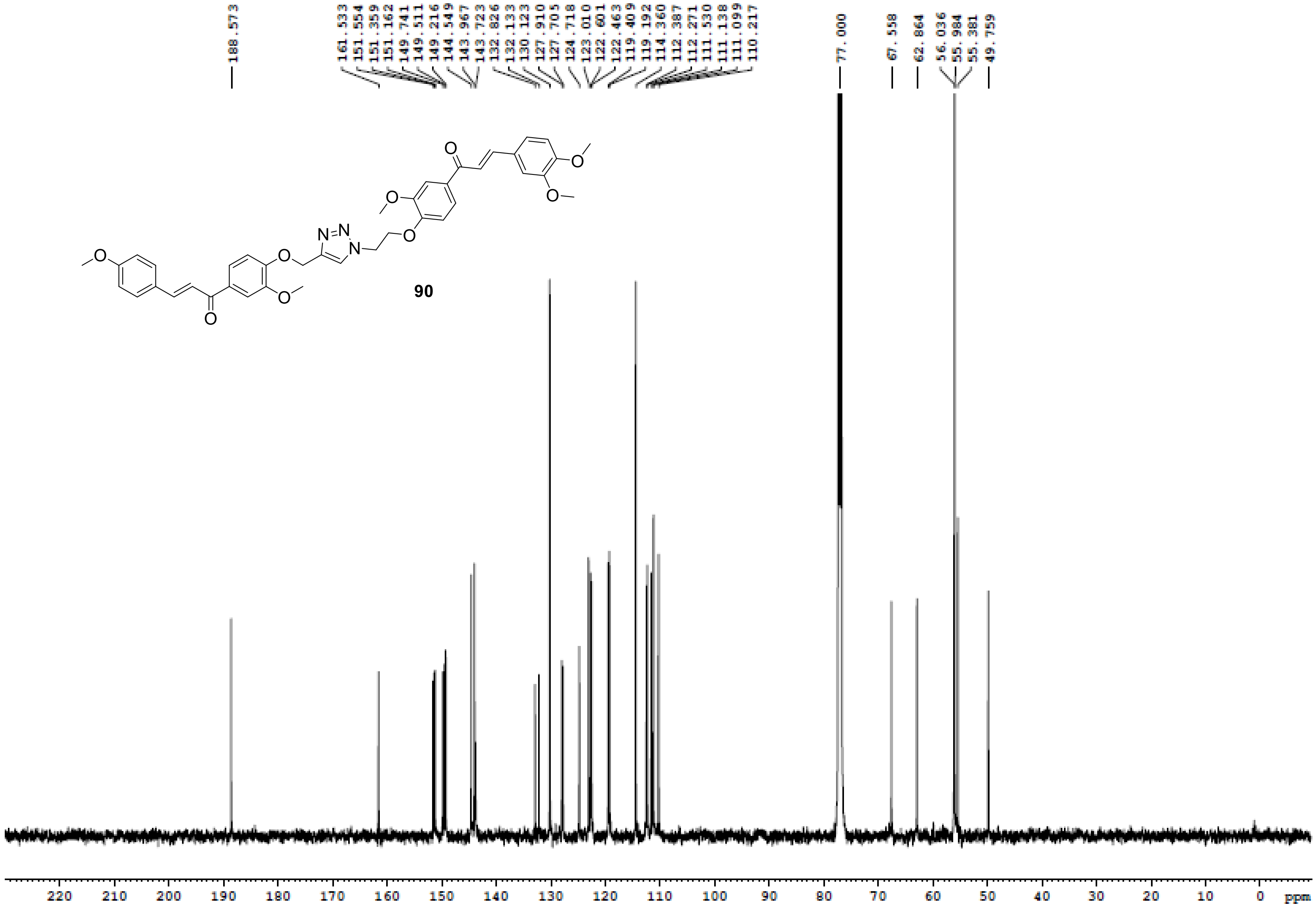
188.573
161.533
151.554
151.359
151.162
149.741
149.511
149.216
144.549
143.967
143.723
132.826
132.133
130.123
127.910
127.705
124.718
123.010
122.601
122.463
119.409
119.192
114.360
112.387
112.271
111.530
111.138
111.099
110.217
77.000
67.558
62.864
56.036
55.984
55.381
49.759
90
220 210 200 190 180 170 160 150 140 130 120 110 100 90 80 70 60 50 40 30 20 10 0 ppm

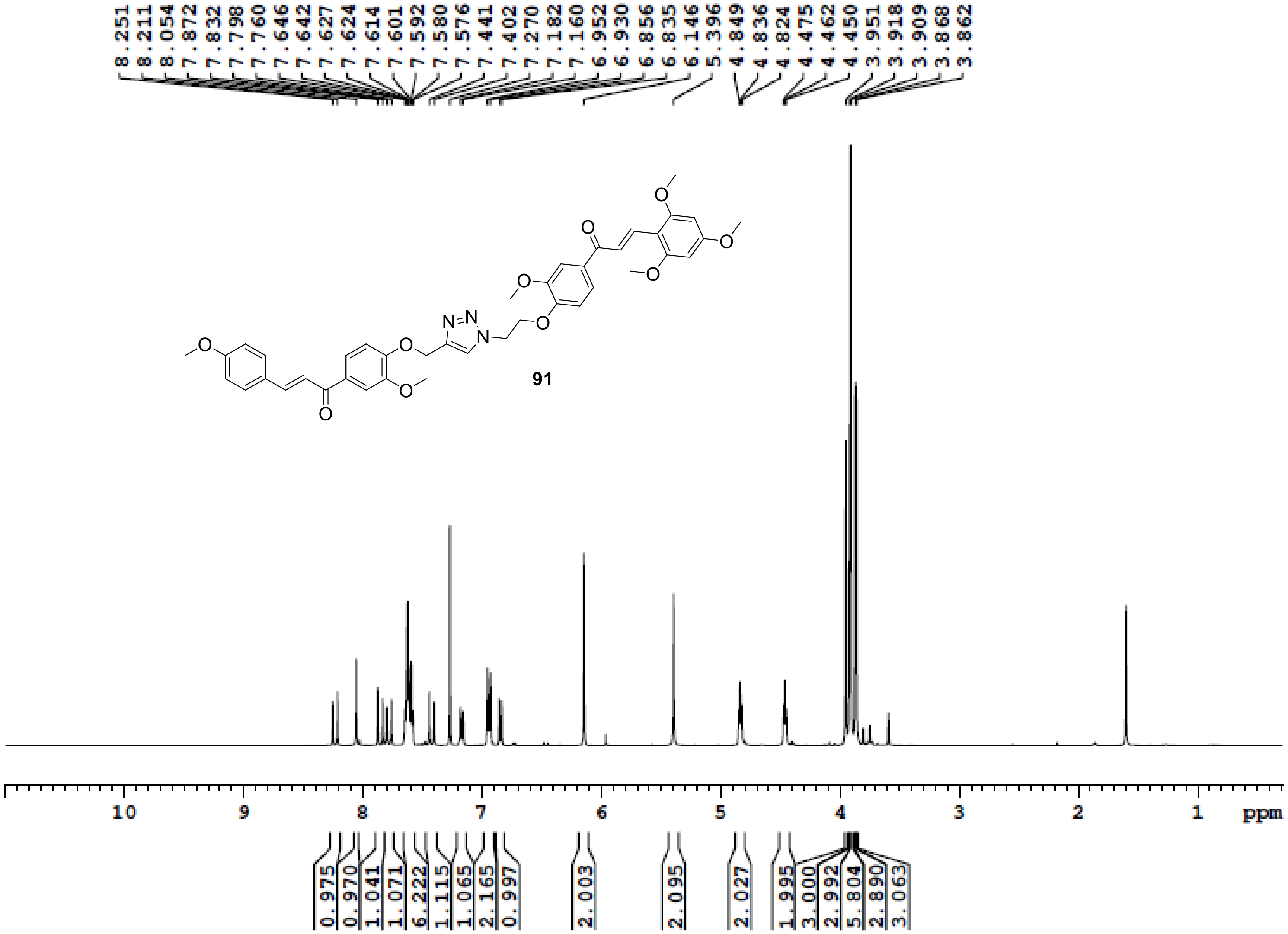
8.251
8.211
8.054
7.872
7.832
7.798
7.760
7.646
7.642
7.627
7.624
7.614
7.601
7.592
7.580
7.576
7.441
7.402
7.270
7.182
7.160
6.952
6.930
6.856
6.835
6.146
5.396
4.849
4.836
4.824
4.475
4.462
4.450
3.951
3.918
3.909
3.868
3.862
91
10
9
8
7
6
5
4
3
2
1
ppm
0.975
0.970
1.041
1.071
6.222
1.115
1.065
2.165
0.997
2.003
2.095
2.027
1.995
3.000
2.992
5.804
2.890
3.063

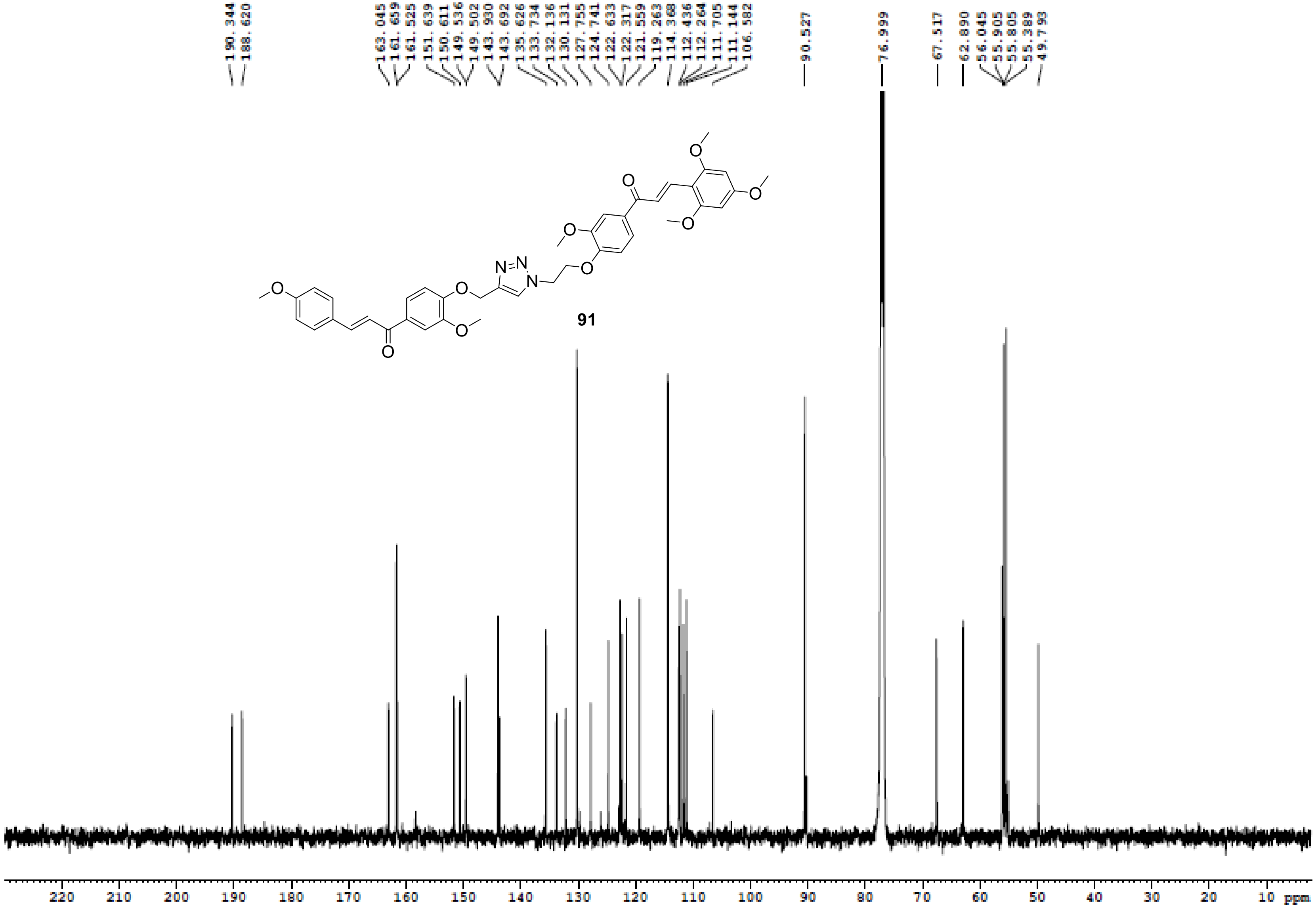
190.344
188.620
163.045
161.659
161.525
151.639
150.611
149.536
149.502
143.930
143.692
135.626
133.734
132.136
130.131
127.755
124.741
122.633
122.317
121.559
119.263
114.368
112.436
112.264
111.705
111.144
106.582
90.527
76.999
67.517
62.890
56.045
55.905
55.805
55.389
49.793
91
220 210 200 190 180 170 160 150 140 130 120 110 100 90 80 70 60 50 40 30 20 10 ppm

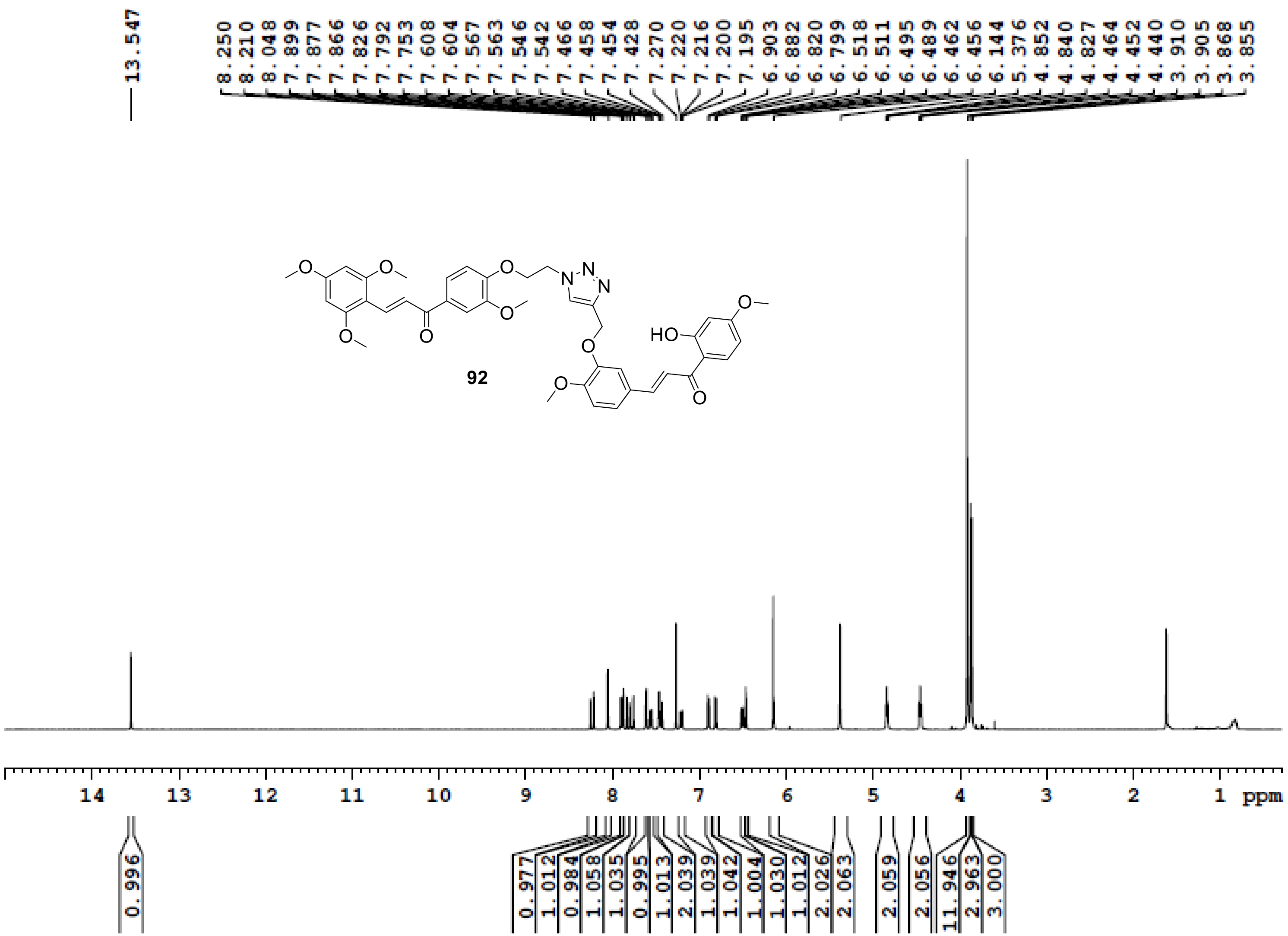
13.547
8.250 8.210 8.048 7.899 7.877 7.866 7.826 7.792 7.753 7.608 7.604 7.567 7.563 7.546 7.542 7.466 7.458 7.454 7.428 7.270 7.220 7.216 7.200 7.195 6.903 6.882 6.820 6.799 6.518 6.511 6.495 6.489 6.462 6.456 6.144 5.376 4.852 4.840 4.827 4.464 4.452 4.440 3.910 3.905 3.868 3.855
HO
92
14 13 12 11 10 9 8 7 6 5 4 3 2 1 ppm
0.996
0.977 1.012 0.984 1.058 1.035 0.995 1.013 2.039 1.039 1.042 1.004 1.030 1.012 2.026 2.063 2.059 2.056 11.946 2.963 3.000

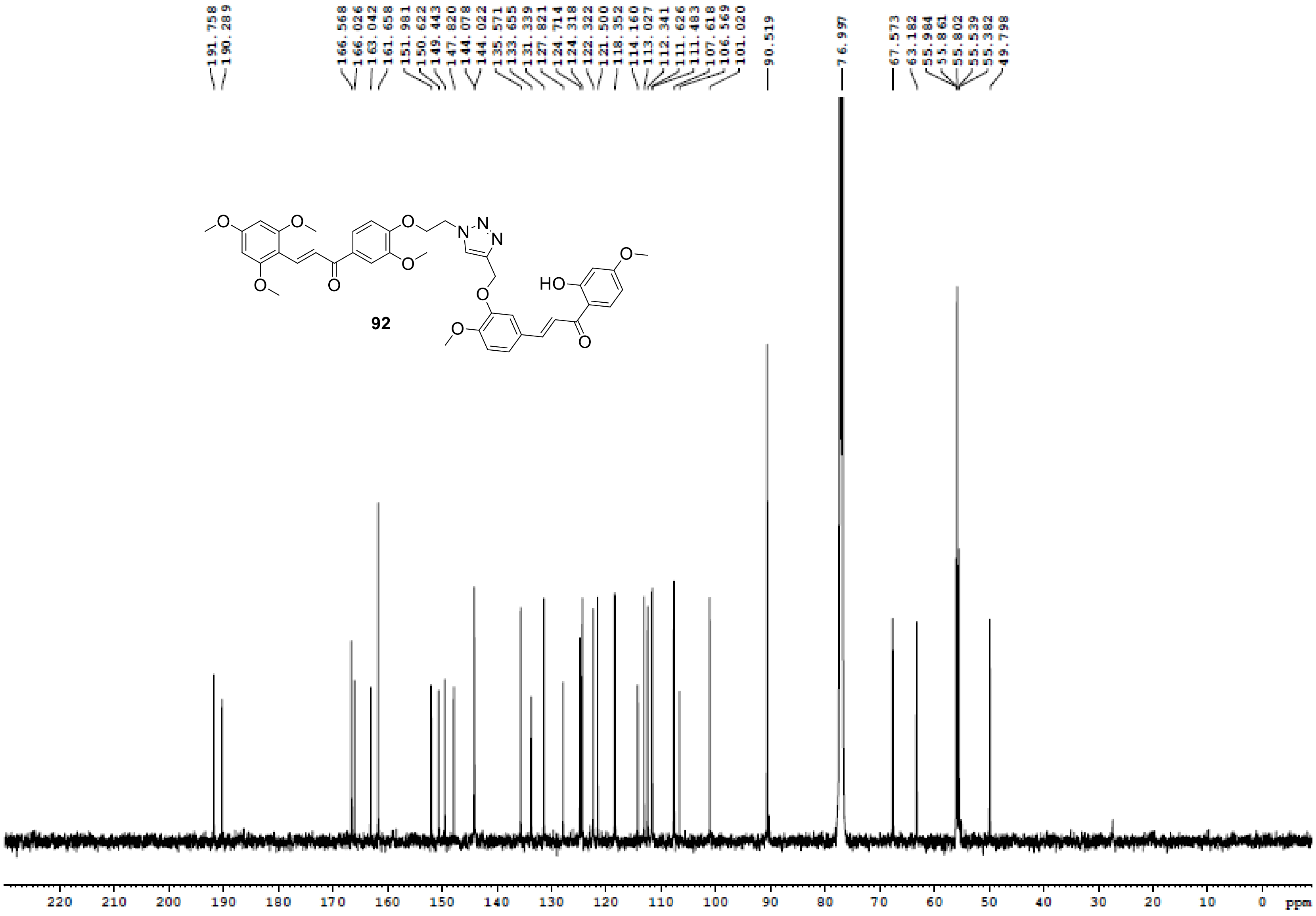
191.758
190.289
166.568
166.026
163.042
161.658
151.981
150.622
149.443
147.820
144.078
144.022
135.571
133.655
131.339
127.821
124.714
124.318
122.322
121.500
118.352
114.160
113.027
112.341
111.626
111.483
107.618
106.569
101.020
90.519
76.997
67.573
63.182
55.984
55.861
55.802
55.539
55.382
49.798
HO
92
220 210 200 190 180 170 160 150 140 130 120 110 100 90 80 70 60 50 40 30 20 10 0 ppm

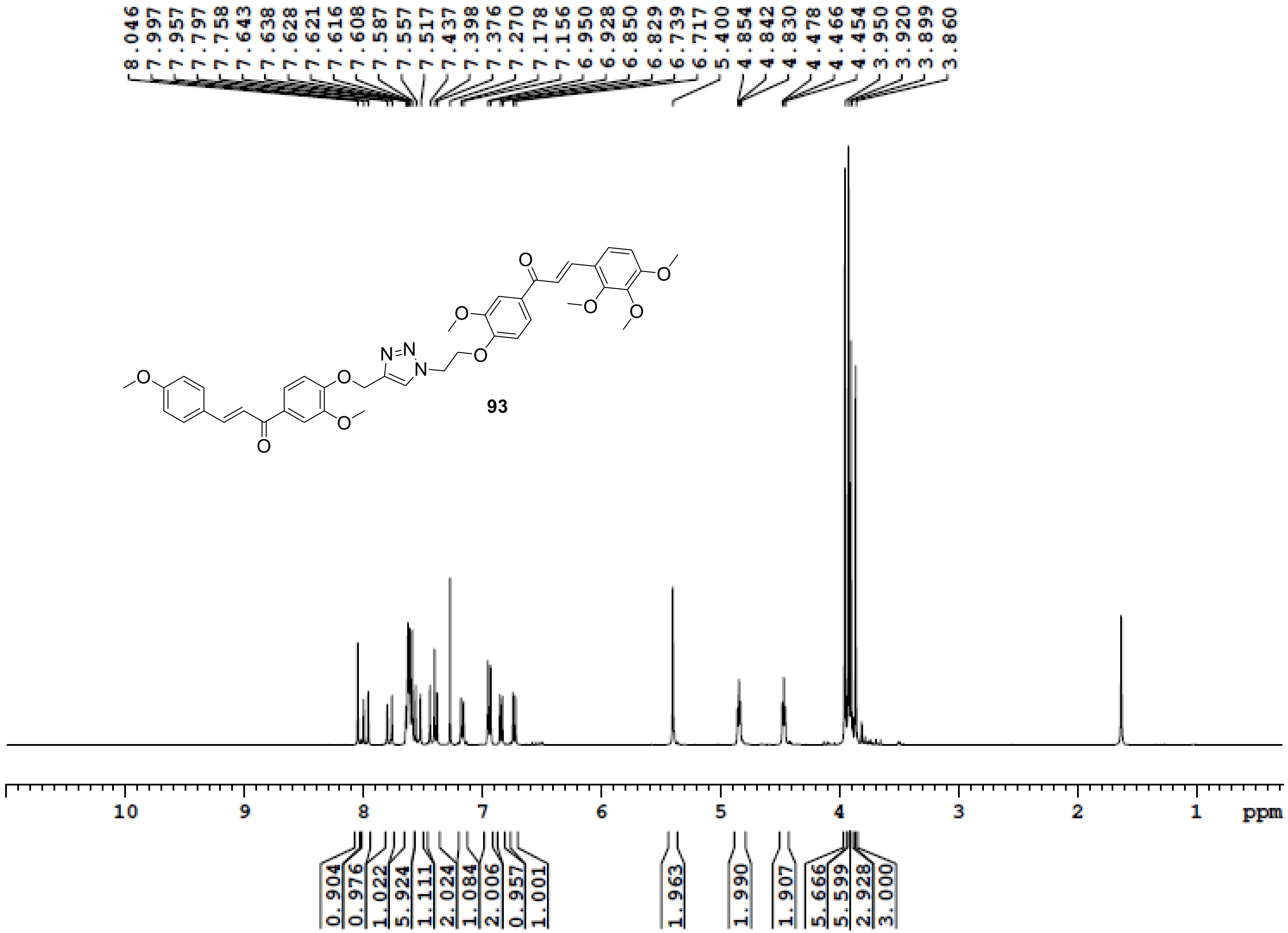
8.046
7.997
7.957
7.797
7.758
7.643
7.638
7.628
7.621
7.616
7.608
7.587
7.557
7.517
7.437
7.398
7.376
7.270
7.178
7.156
6.950
6.928
6.850
6.829
6.739
6.717
5.400
4.854
4.842
4.830
4.478
4.466
4.454
3.950
3.920
3.899
3.860
93
10
9
8
7
6
5
4
3
2
1
ppm
0.904
0.976
1.022
5.924
1.111
2.024
1.084
2.006
0.957
1.001
1.963
1.990
1.907
5.666
5.599
2.928
3.000

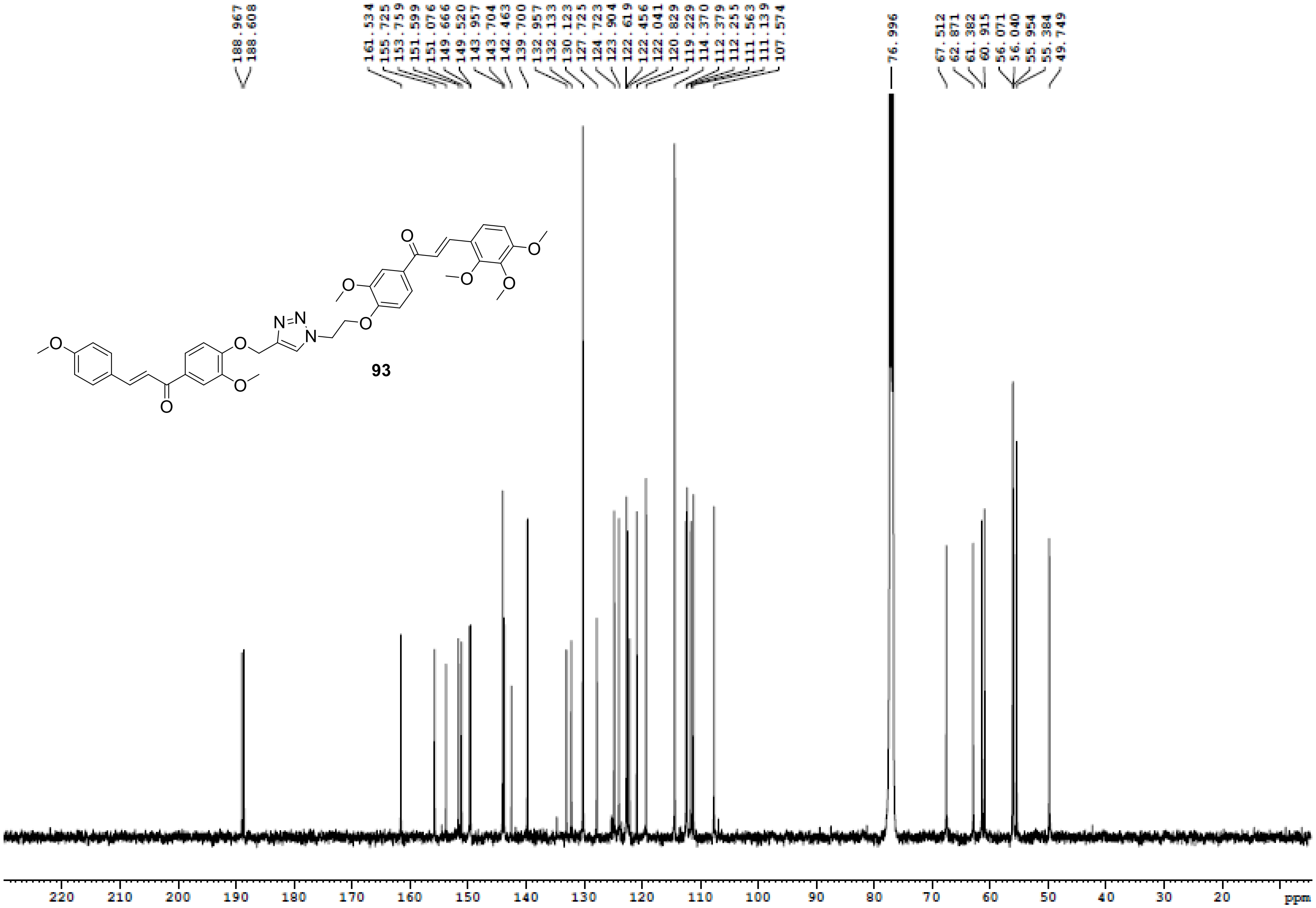
188.967
188.608
161.534
155.725
153.759
151.599
151.076
149.666
149.520
143.957
143.704
142.463
139.700
132.957
132.133
130.123
127.725
124.723
123.904
122.619
122.456
122.041
120.829
119.229
114.370
112.379
112.255
111.563
111.139
107.574
76.996
67.512
62.871
61.382
60.915
56.071
56.040
55.954
55.384
49.749
93
220 210 200 190 180 170 160 150 140 130 120 110 100 90 80 70 60 50 40 30 20 ppm

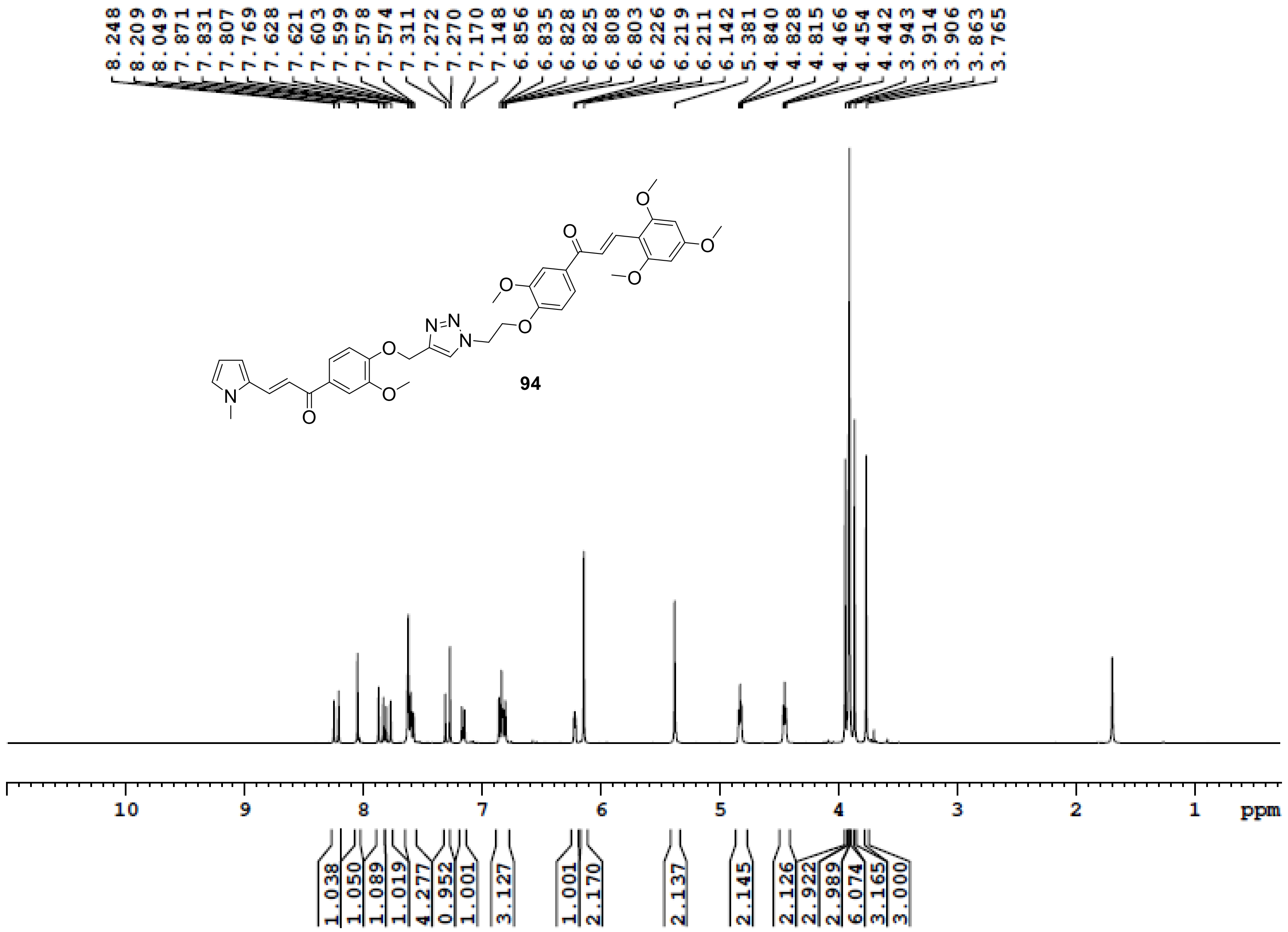
8.248 8.209 8.049 7.871 7.831 7.807 7.769 7.628 7.621 7.603 7.599 7.578 7.574 7.311 7.272 7.270 7.170 7.148 6.856 6.835 6.828 6.825 6.808 6.803 6.226 6.219 6.211 6.142 5.381 4.840 4.828 4.815 4.466 4.454 4.442 3.943 3.914 3.906 3.863 3.765
94
10 9 8 7 6 5 4 3 2 1 ppm
1.038 1.050 1.089 1.019 4.277 0.952 1.001 3.127 1.001 2.170 2.137 2.145 2.126 2.922 2.989 6.074 3.165 3.000

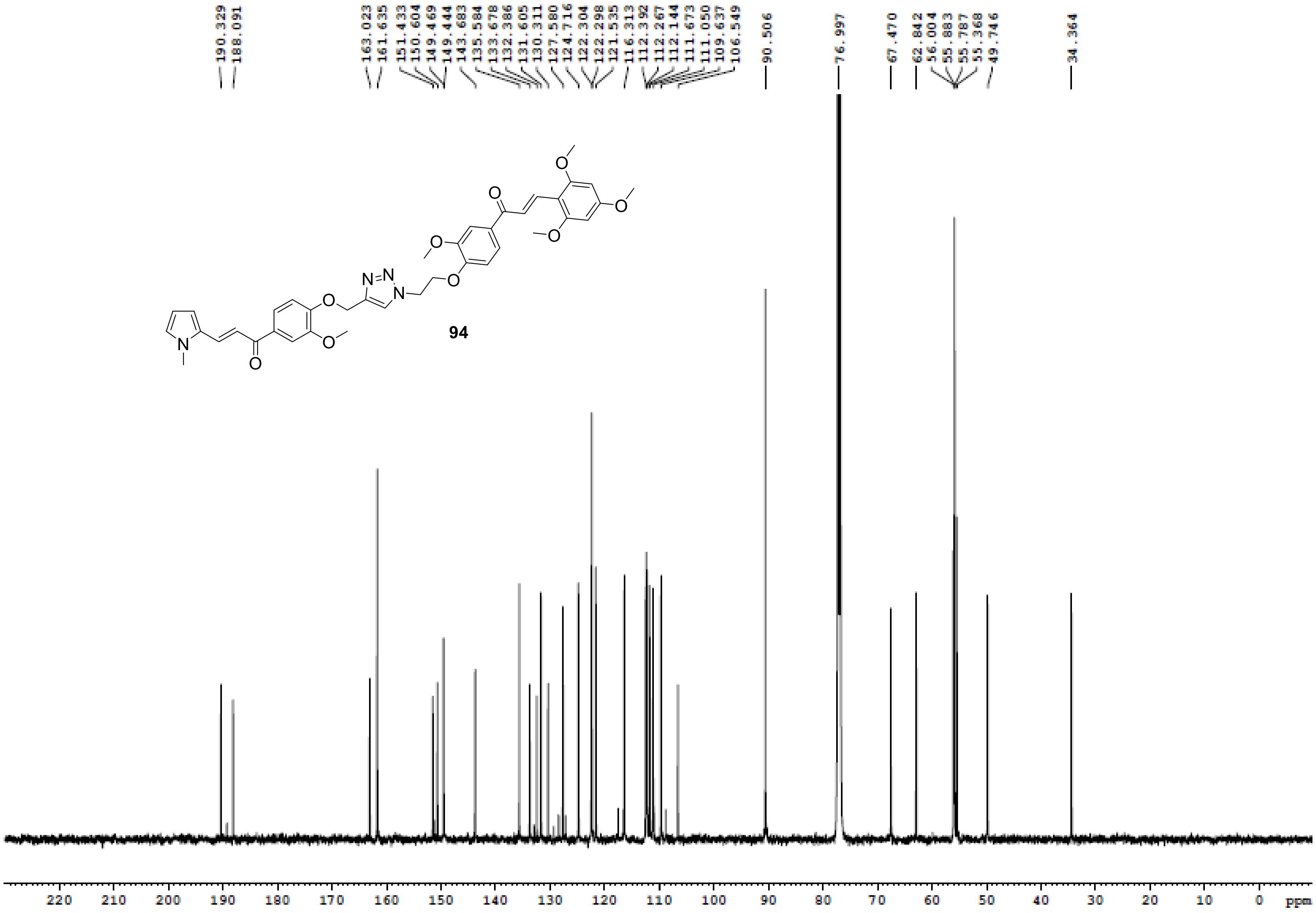
190.329
188.091
163.023
161.635
151.433
150.604
149.469
149.444
143.683
135.584
133.678
132.386
131.605
130.311
127.580
124.716
122.304
122.298
121.535
116.313
112.392
112.267
112.144
111.673
111.050
109.637
106.549
90.506
76.997
67.470
62.842
56.004
55.883
55.787
55.368
49.746
34.364
94
220 210 200 190 180 170 160 150 140 130 120 110 100 90 80 70 60 50 40 30 20 10 0 ppm

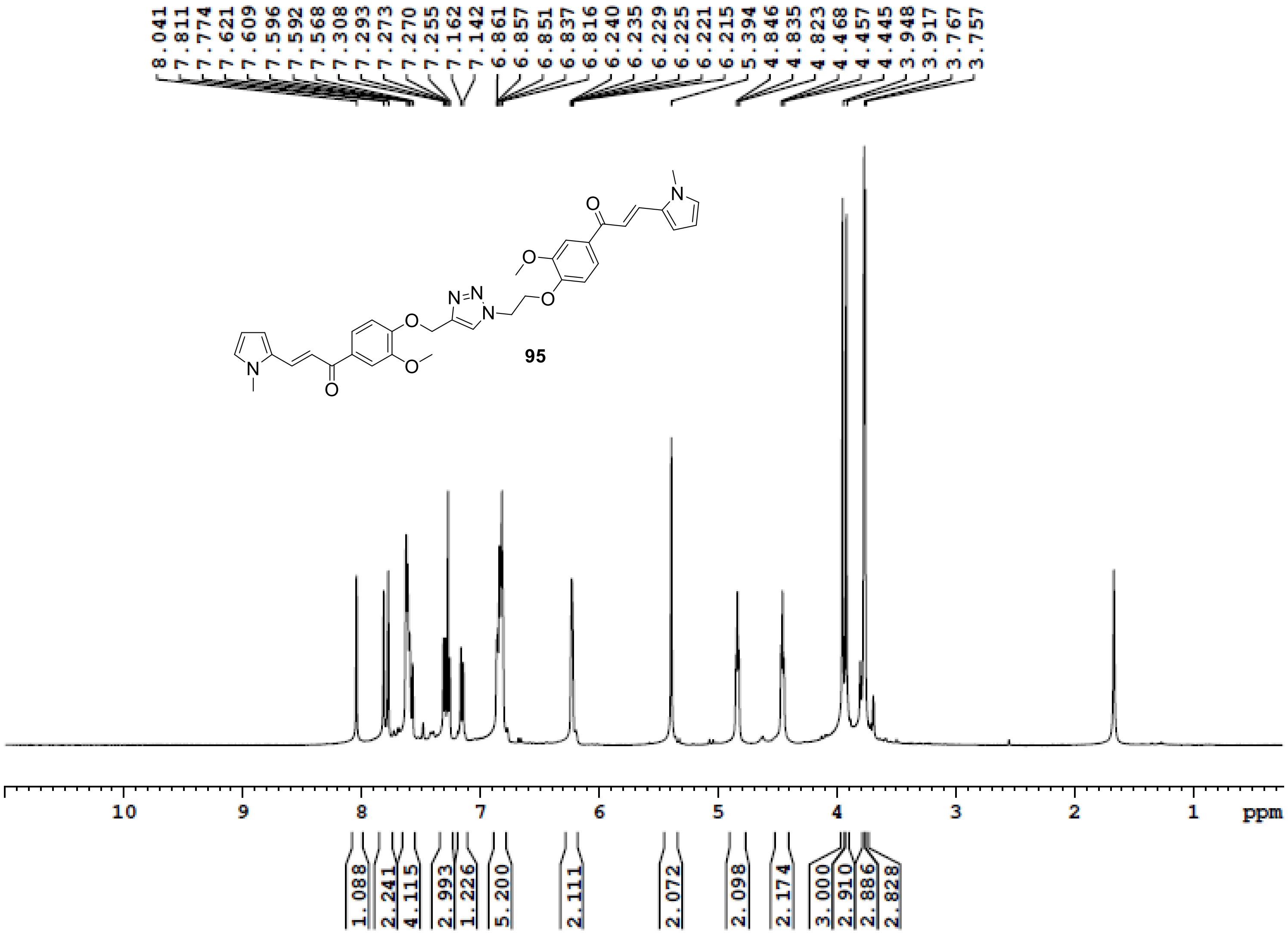
8.041
7.811
7.774
7.621
7.609
7.596
7.592
7.568
7.308
7.293
7.273
7.270
7.255
7.162
7.142
6.861
6.857
6.851
6.837
6.816
6.240
6.235
6.229
6.225
6.221
6.215
5.394
4.846
4.835
4.823
4.468
4.457
4.445
3.948
3.917
3.767
3.757
95
10
9
8
7
6
5
4
3
2
1
ppm
1.088
2.241
4.115
2.993
1.226
5.200
2.111
2.072
2.098
2.174
3.000
2.910
2.886
2.828

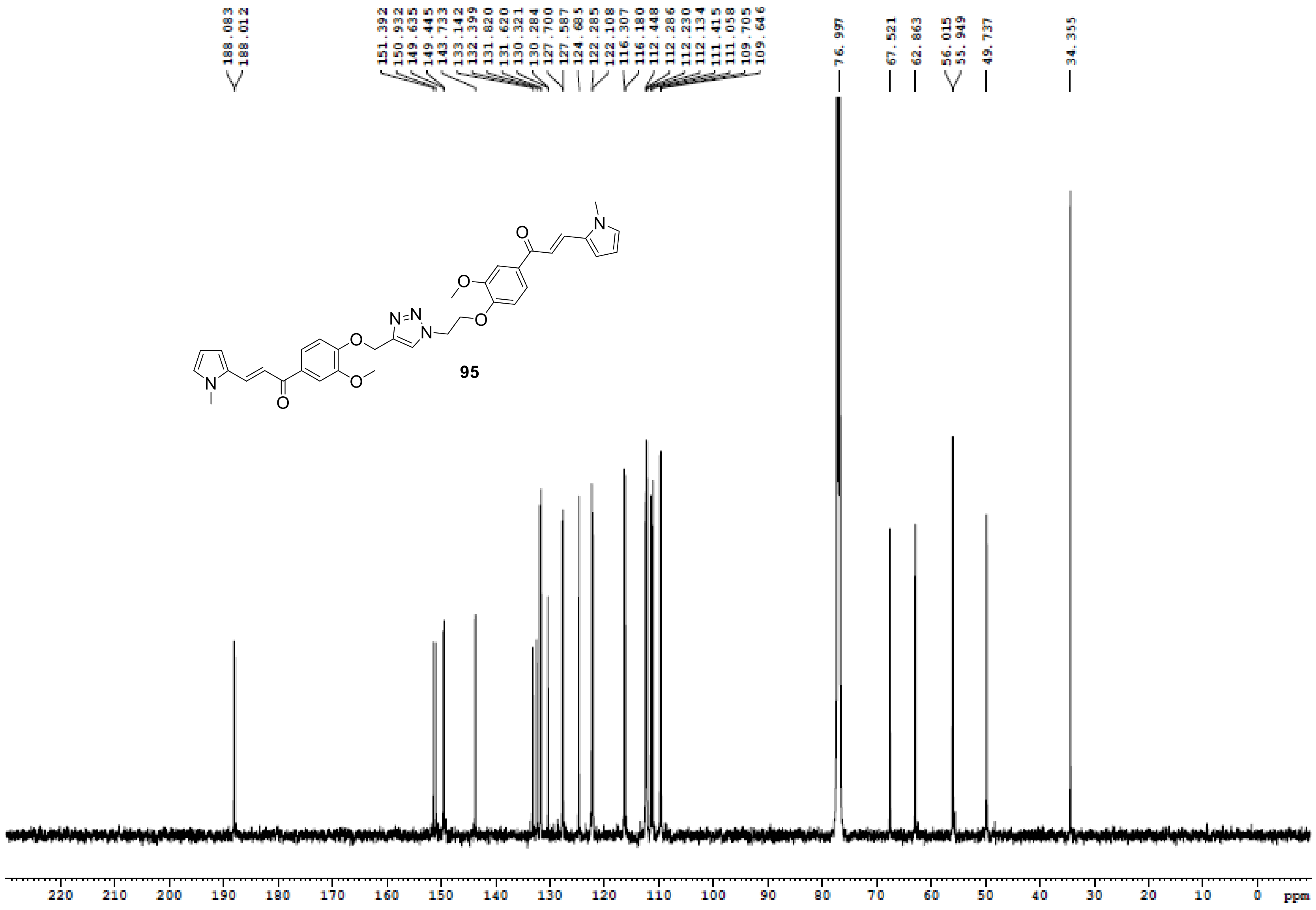
188.083
188.012
151.392
150.932
149.635
149.445
143.733
133.142
132.399
131.820
131.620
130.321
130.284
127.700
127.587
124.685
122.285
122.108
116.307
116.180
112.448
112.286
112.230
112.134
111.415
111.058
109.705
109.646
76.997
67.521
62.863
56.015
55.949
49.737
34.355
95
220
210
200
190
180
170
160
150
140
130
120
110
100
90
80
70
60
50
40
30
20
10
0
ppm

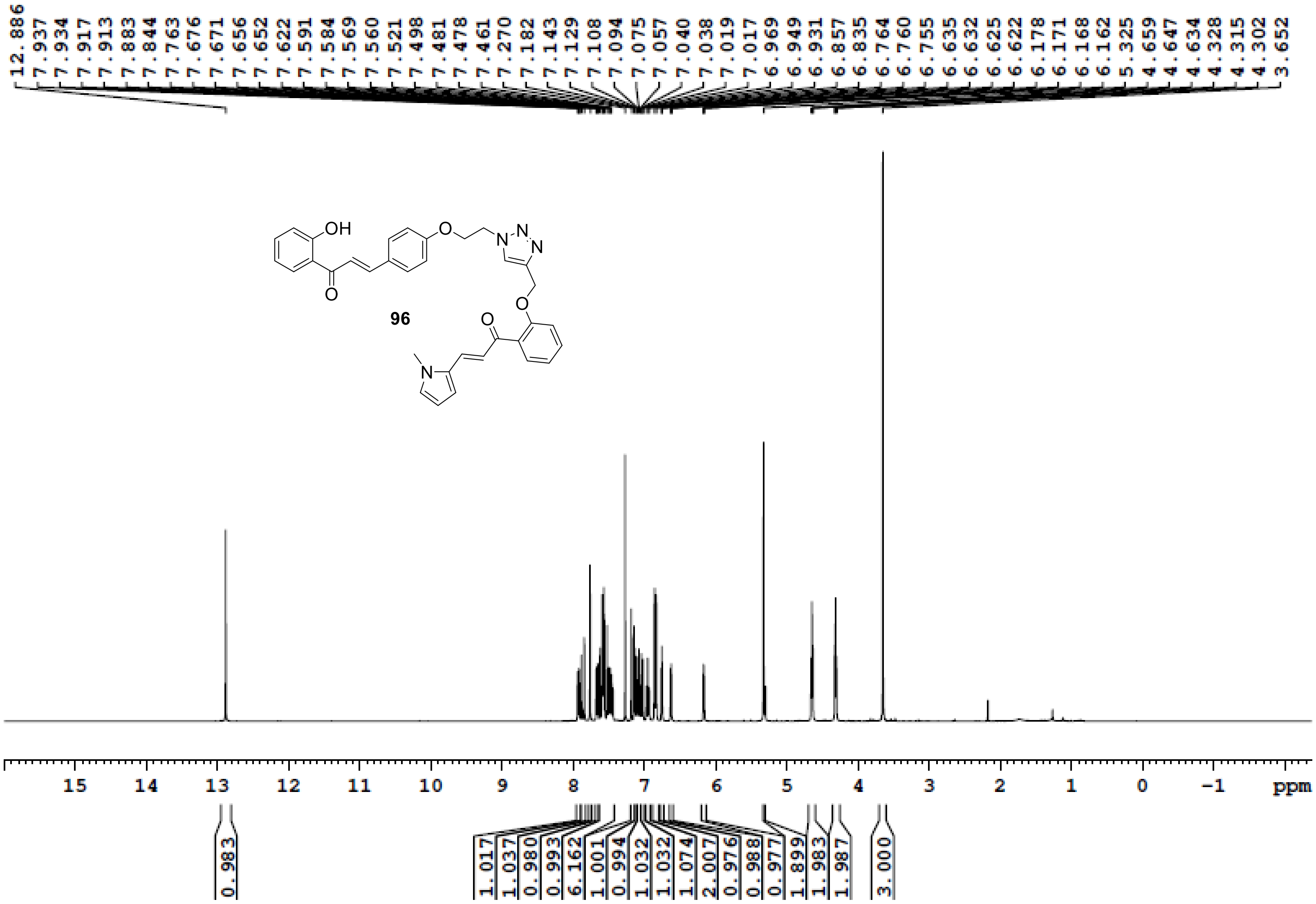
12.886
7.937
7.934
7.917
7.913
7.883
7.844
7.763
7.676
7.671
7.656
7.652
7.622
7.591
7.584
7.569
7.560
7.521
7.498
7.481
7.478
7.461
7.270
7.182
7.143
7.129
7.108
7.094
7.075
7.057
7.040
7.038
7.019
7.017
6.969
6.949
6.931
6.857
6.835
6.764
6.760
6.755
6.635
6.632
6.625
6.622
6.178
6.171
6.168
6.162
5.325
4.659
4.647
4.634
4.328
4.315
4.302
3.652
OH
O
N
N
N
O
O
O
N
96
15
14
13
12
11
10
9
8
7
6
5
4
3
2
1
0
-1
ppm
0.983
1.017
1.037
0.980
0.993
6.162
1.001
0.994
1.032
1.032
1.074
2.007
0.976
0.988
0.977
1.899
1.983
1.987
3.000

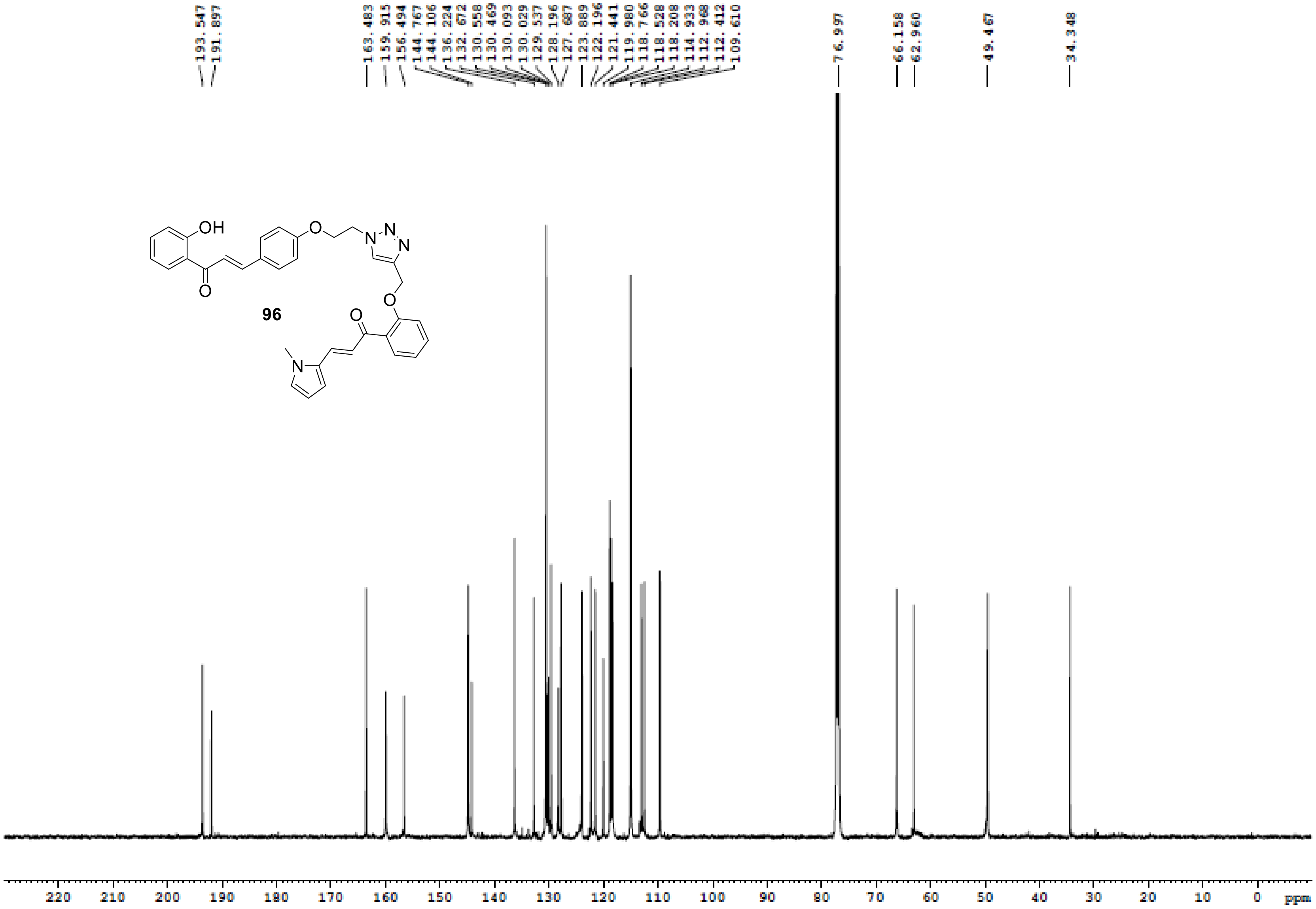
193.547
191.897
163.483
159.915
156.494
144.767
144.106
136.224
132.672
130.558
130.469
130.093
130.029
129.537
128.196
127.687
123.889
122.196
121.441
119.980
118.766
118.528
118.208
114.933
112.968
112.412
109.610
76.997
66.158
62.960
49.467
34.348
OH
O
N
N
N
O
O
O
N
96
220 210 200 190 180 170 160 150 140 130 120 110 100 90 80 70 60 50 40 30 20 10 0 ppm

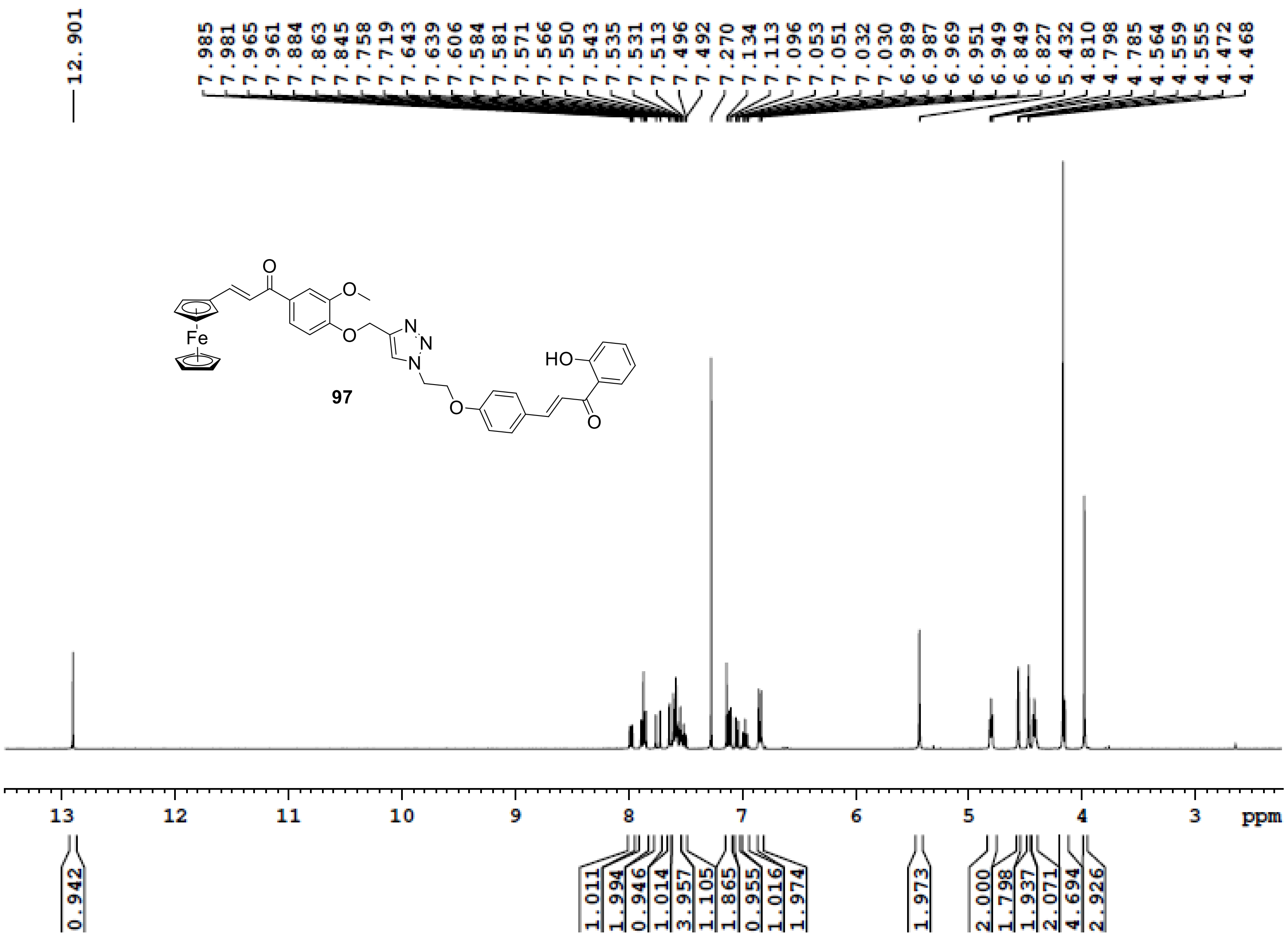
12.901
7.985 7.981 7.965 7.961 7.884 7.863 7.845 7.758 7.719 7.643 7.639 7.606 7.584 7.581 7.571 7.566 7.550 7.543 7.535 7.531 7.513 7.496 7.492 7.270 7.134 7.113 7.096 7.053 7.051 7.032 7.030 6.989 6.987 6.969 6.951 6.949 6.849 6.827 5.432 4.810 4.798 4.785 4.564 4.559 4.555 4.472 4.468
O
O
Fe
N
N
N
HO
O
O
97
13 12 11 10 9 8 7 6 5 4 3 ppm
0.942
1.011 1.994 0.946 1.014 3.957 1.105 1.865 0.955 1.016 1.974
1.973
2.000 1.798 1.937 2.071 4.694 2.926

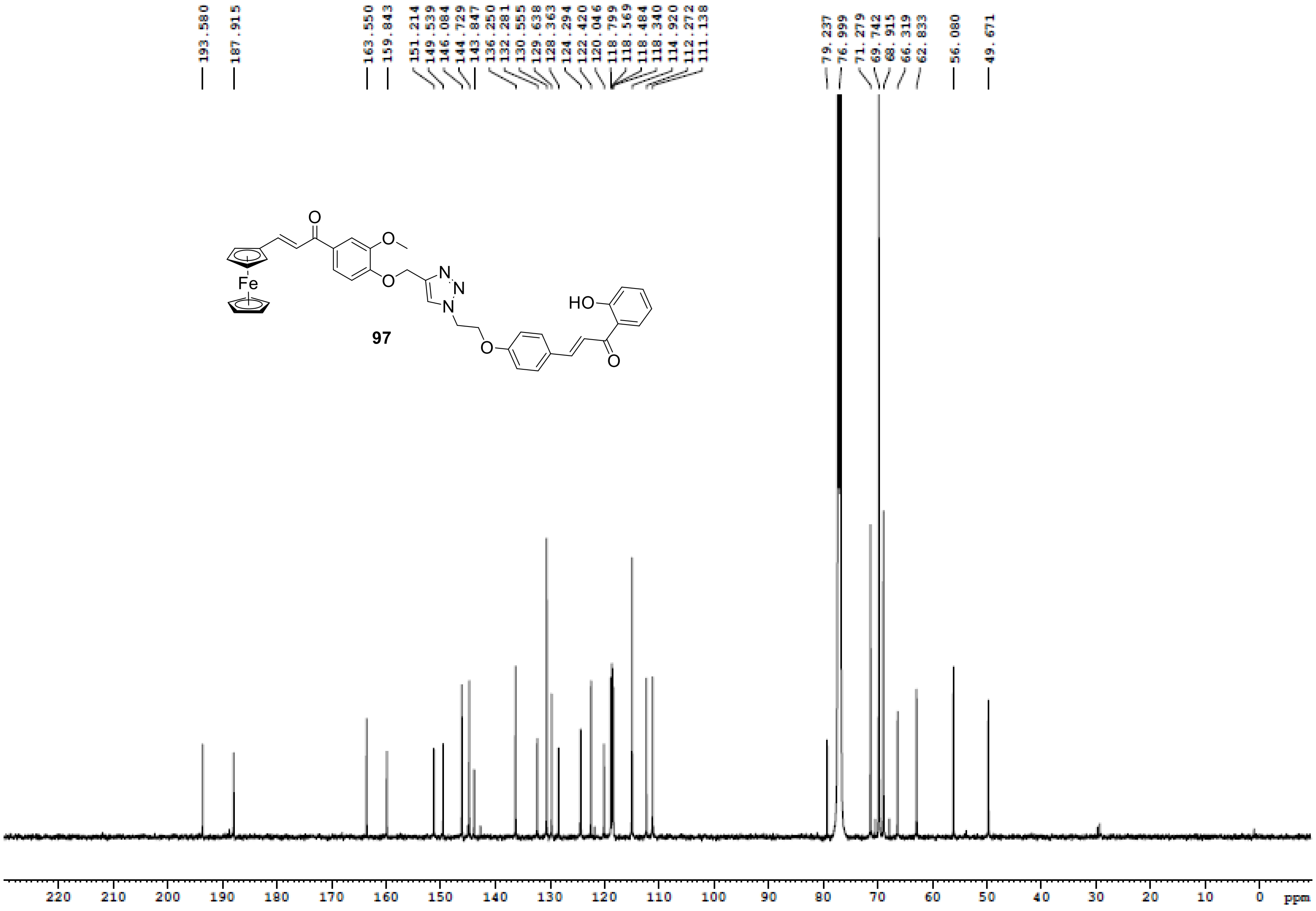
193.580
187.915
163.550
159.843
151.214
149.539
146.084
144.729
143.847
136.250
132.281
130.555
129.638
128.363
124.294
122.420
120.046
118.799
118.569
118.484
118.340
114.920
112.272
111.138
79.237
76.999
71.279
69.742
68.915
66.319
62.833
56.080
49.671
Fe
HO
O
N
97
ppm

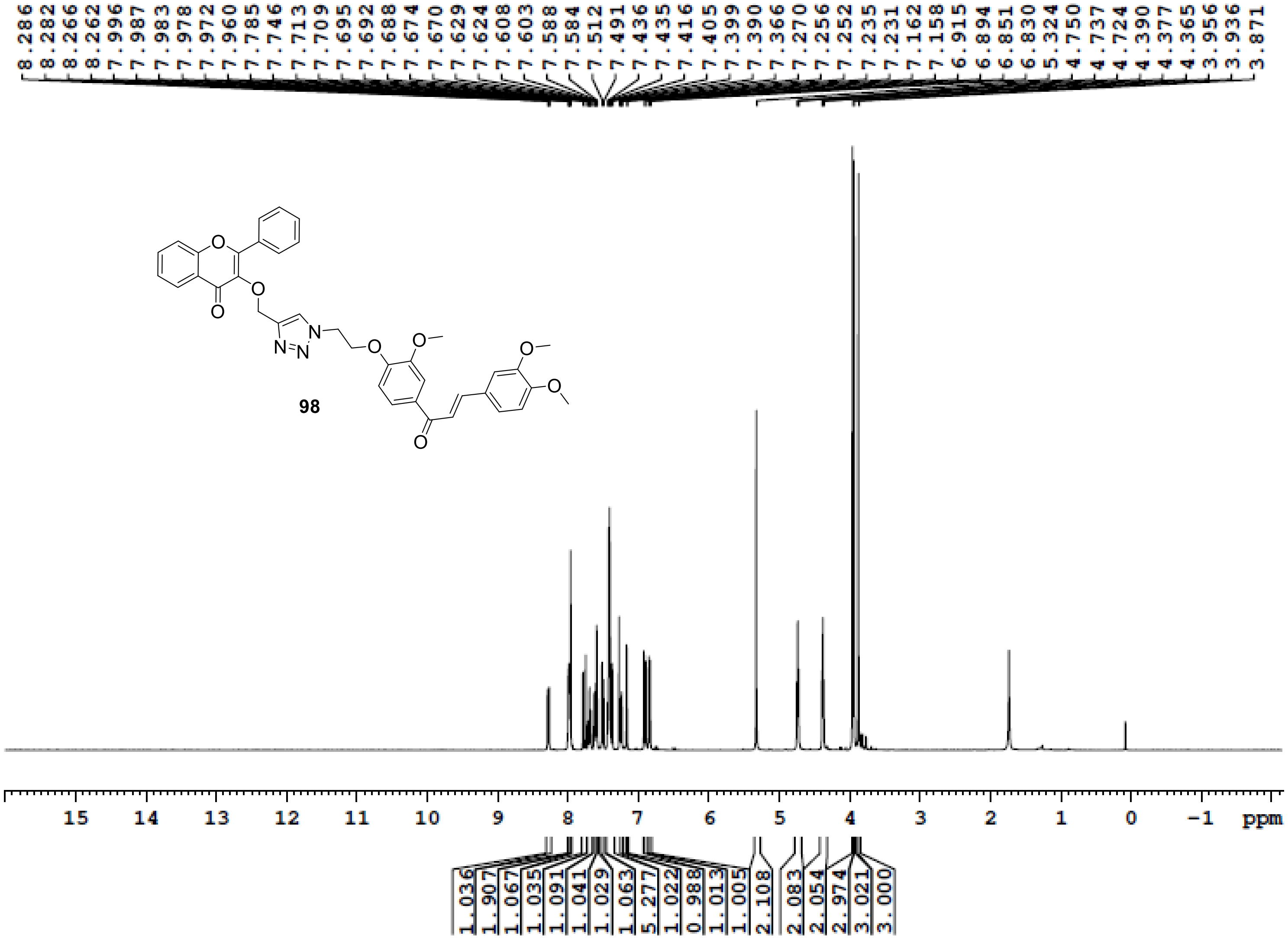
8.286
8.282
8.266
8.262
7.996
7.987
7.983
7.978
7.972
7.960
7.785
7.746
7.713
7.709
7.695
7.692
7.688
7.674
7.670
7.629
7.624
7.608
7.603
7.588
7.584
7.512
7.491
7.436
7.435
7.416
7.405
7.399
7.390
7.366
7.270
7.256
7.252
7.235
7.231
7.162
7.158
6.915
6.894
6.851
6.830
5.324
4.750
4.737
4.724
4.390
4.377
4.365
3.956
3.936
3.871
15 14 13 12 11 10 9 8 7 6 5 4 3 2 1 0 -1 ppm
1.036
1.907
1.067
1.035
1.091
1.041
1.029
1.063
5.277
1.022
0.988
1.013
1.005
2.108
2.083
2.054
2.974
3.021
3.000

98

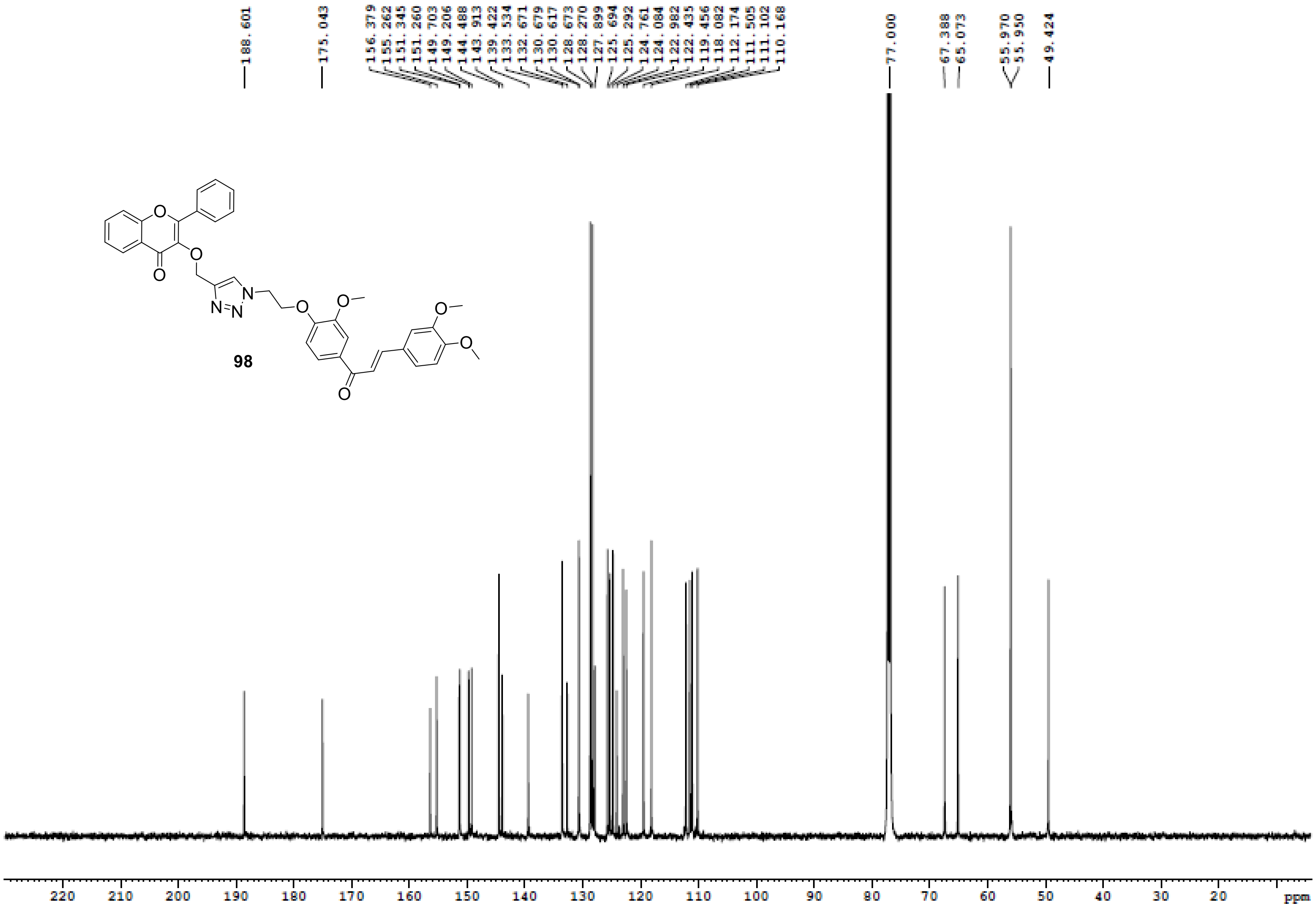
188.601
175.043
156.379
155.262
151.345
151.260
149.703
149.206
144.488
143.913
139.422
133.534
132.671
130.679
130.617
128.673
128.270
127.899
125.694
125.292
124.761
124.084
122.982
122.435
119.456
118.082
112.174
111.505
111.102
110.168
77.000
67.388
65.073
55.970
55.950
49.424
98
220 210 200 190 180 170 160 150 140 130 120 110 100 90 80 70 60 50 40 30 20 ppm

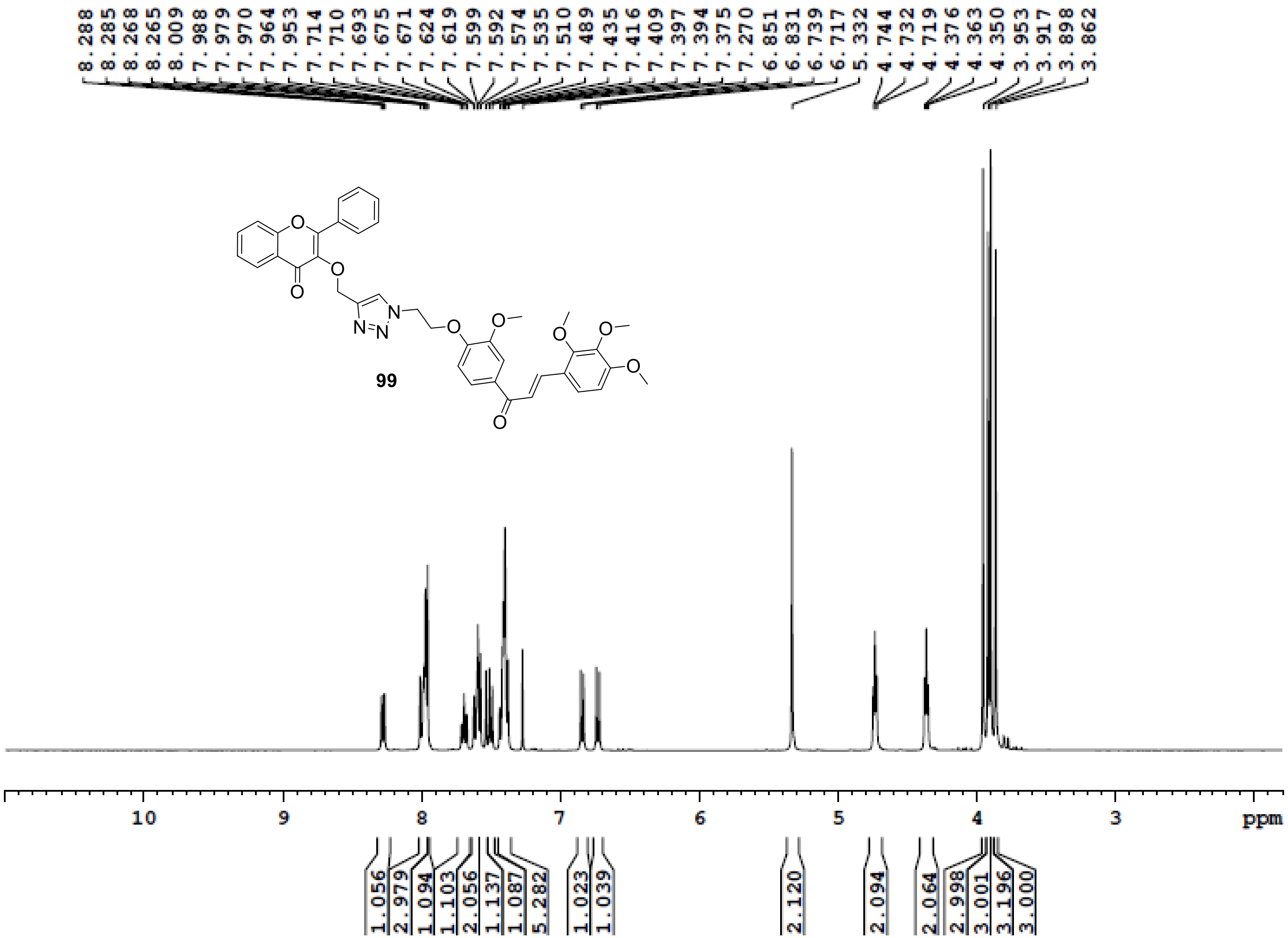
8.288 8.285 8.268 8.265 8.009 7.988 7.979 7.970 7.964 7.953 7.714 7.710 7.693 7.675 7.671 7.624 7.619 7.599 7.592 7.574 7.535 7.510 7.489 7.435 7.416 7.409 7.397 7.394 7.375 7.270 6.851 6.831 6.739 6.717 5.332 4.744 4.732 4.719 4.376 4.363 4.350 3.953 3.917 3.898 3.862
99
10 9 8 7 6 5 4 3 ppm
1.056 2.979 1.094 1.103 2.056 1.137 1.087 5.282 1.023 1.039 2.120 2.094 2.064 2.998 3.001 3.196 3.000

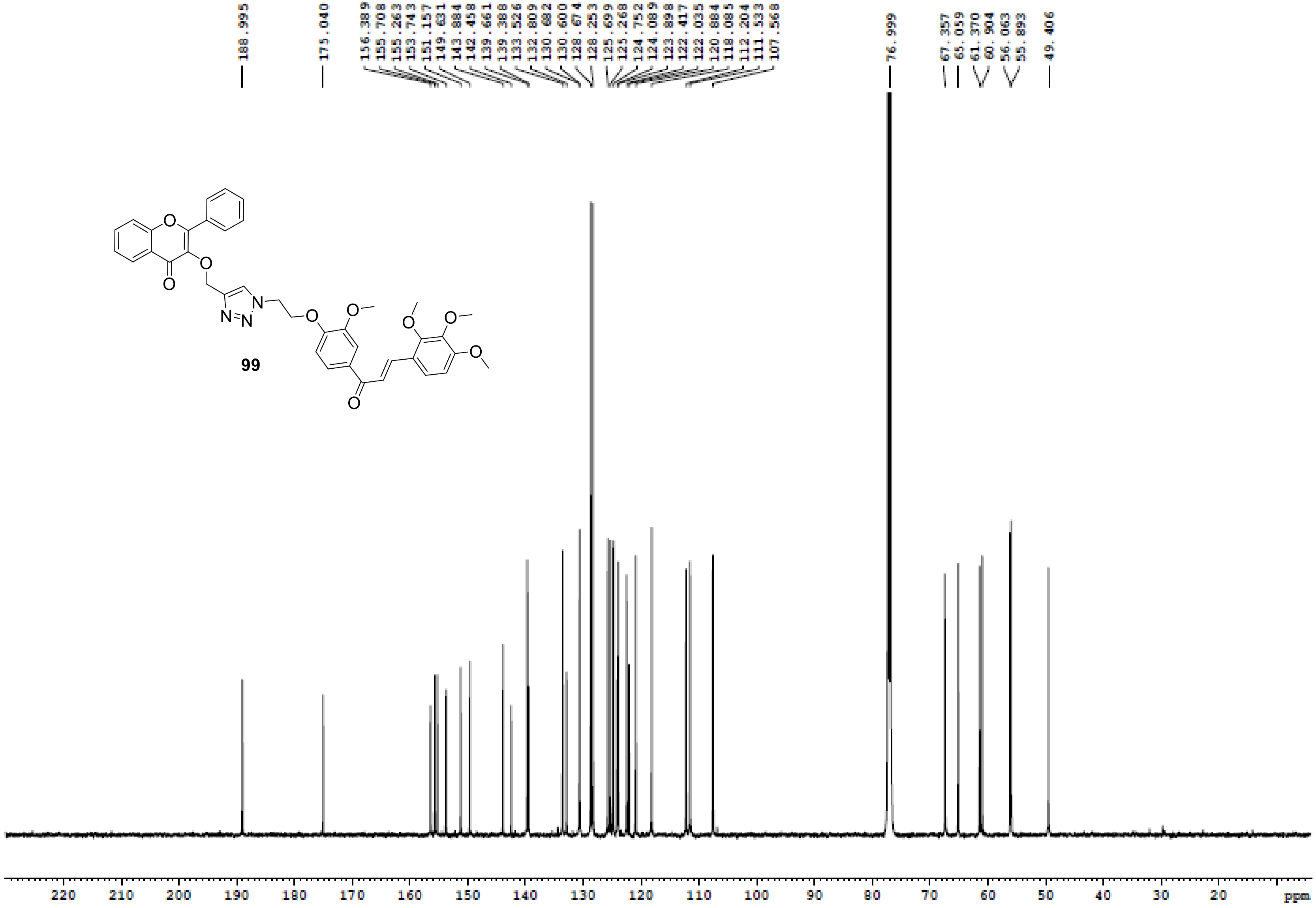

188.995
175.040
156.389
155.708
155.263
153.743
151.157
149.631
143.884
142.458
139.661
139.388
133.526
132.809
130.682
130.600
128.674
128.253
125.699
125.268
124.752
124.089
123.898
122.417
122.035
120.884
118.085
112.204
111.533
107.568
76.999
67.357
65.059
61.370
60.904
56.063
55.893
49.406
99
220 210 200 190 180 170 160 150 140 130 120 110 100 90 80 70 60 50 40 30 20 ppm

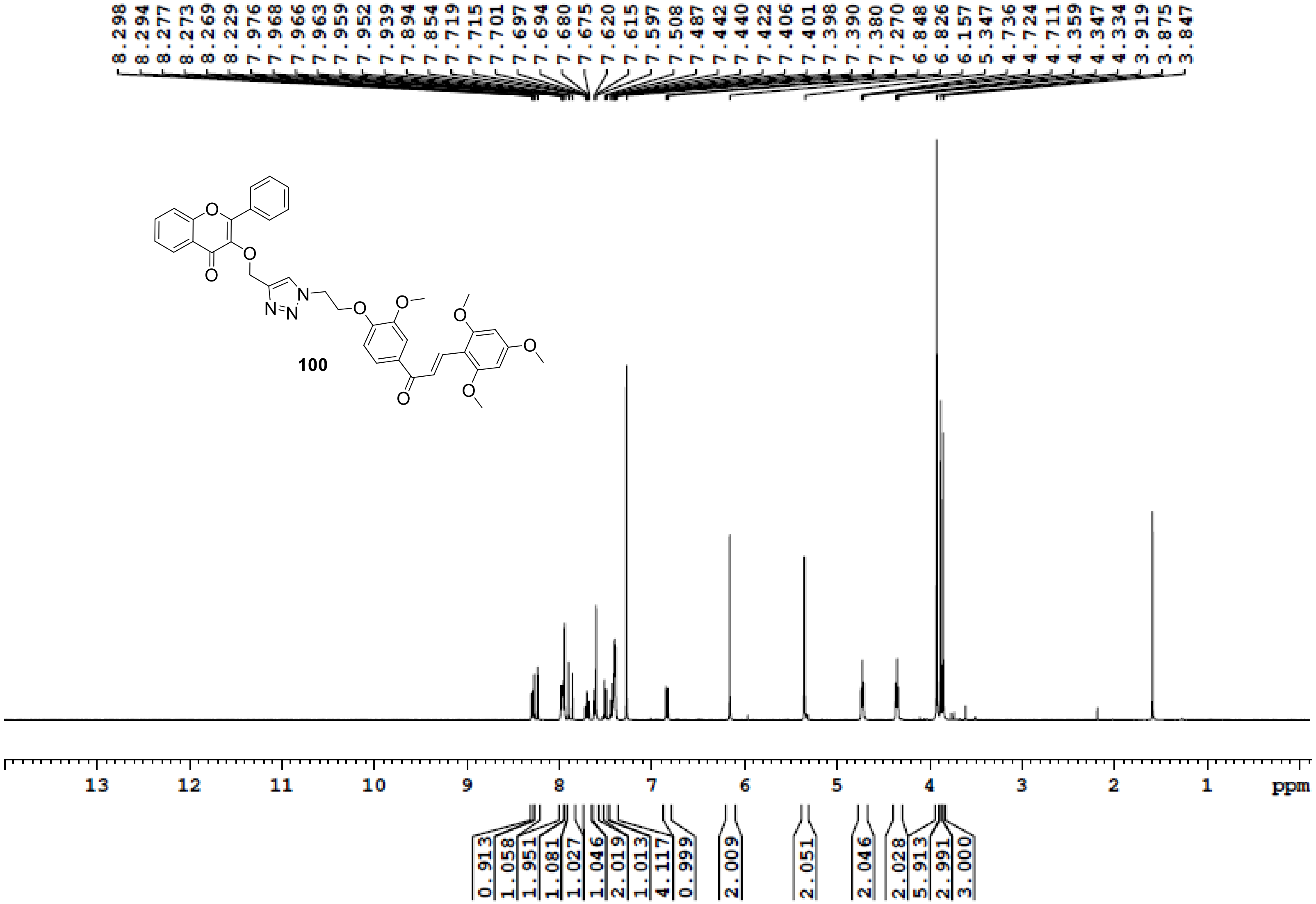
8.298
8.294
8.277
8.273
8.269
8.229
7.976
7.968
7.966
7.963
7.959
7.952
7.939
7.894
7.854
7.719
7.715
7.701
7.697
7.694
7.680
7.675
7.620
7.615
7.597
7.508
7.487
7.442
7.440
7.422
7.406
7.401
7.398
7.390
7.380
7.270
6.848
6.826
6.157
5.347
4.736
4.724
4.711
4.359
4.347
4.334
3.919
3.875
3.847
100
13 12 11 10 9 8 7 6 5 4 3 2 1 ppm
0.913
1.058
1.951
1.081
1.027
1.046
2.019
1.013
4.117
0.999
2.009
2.051
2.046
2.028
5.913
2.991
3.000

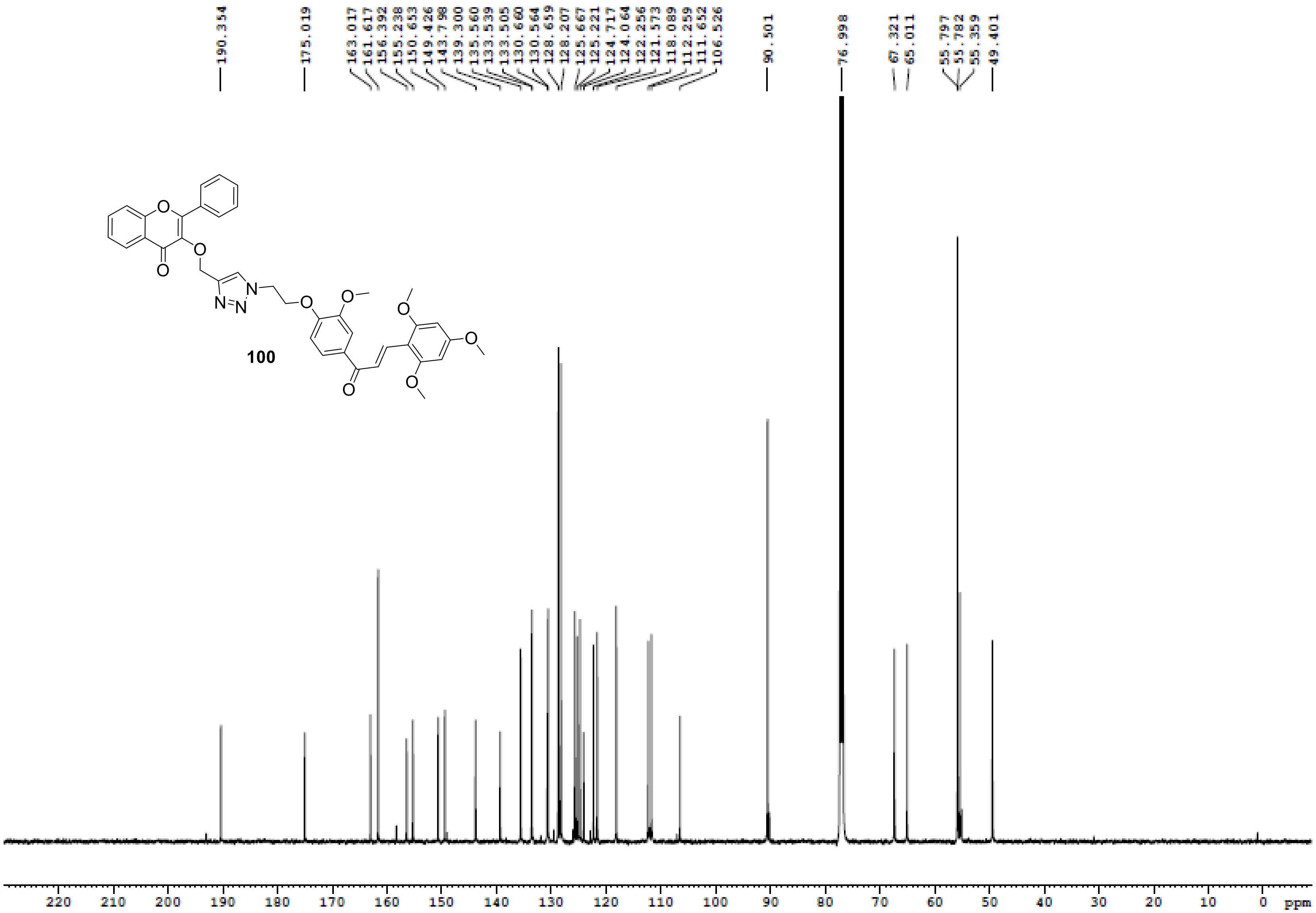
190.354
175.019
163.017
161.617
156.392
155.238
150.653
149.426
143.798
139.300
135.560
133.539
133.505
130.660
130.564
128.659
128.207
125.667
125.221
124.717
124.064
122.256
121.573
118.089
112.259
111.652
106.526
90.501
76.998
67.321
65.011
55.797
55.782
55.359
49.401
100
220 210 200 190 180 170 160 150 140 130 120 110 100 90 80 70 60 50 40 30 20 10 0 ppm

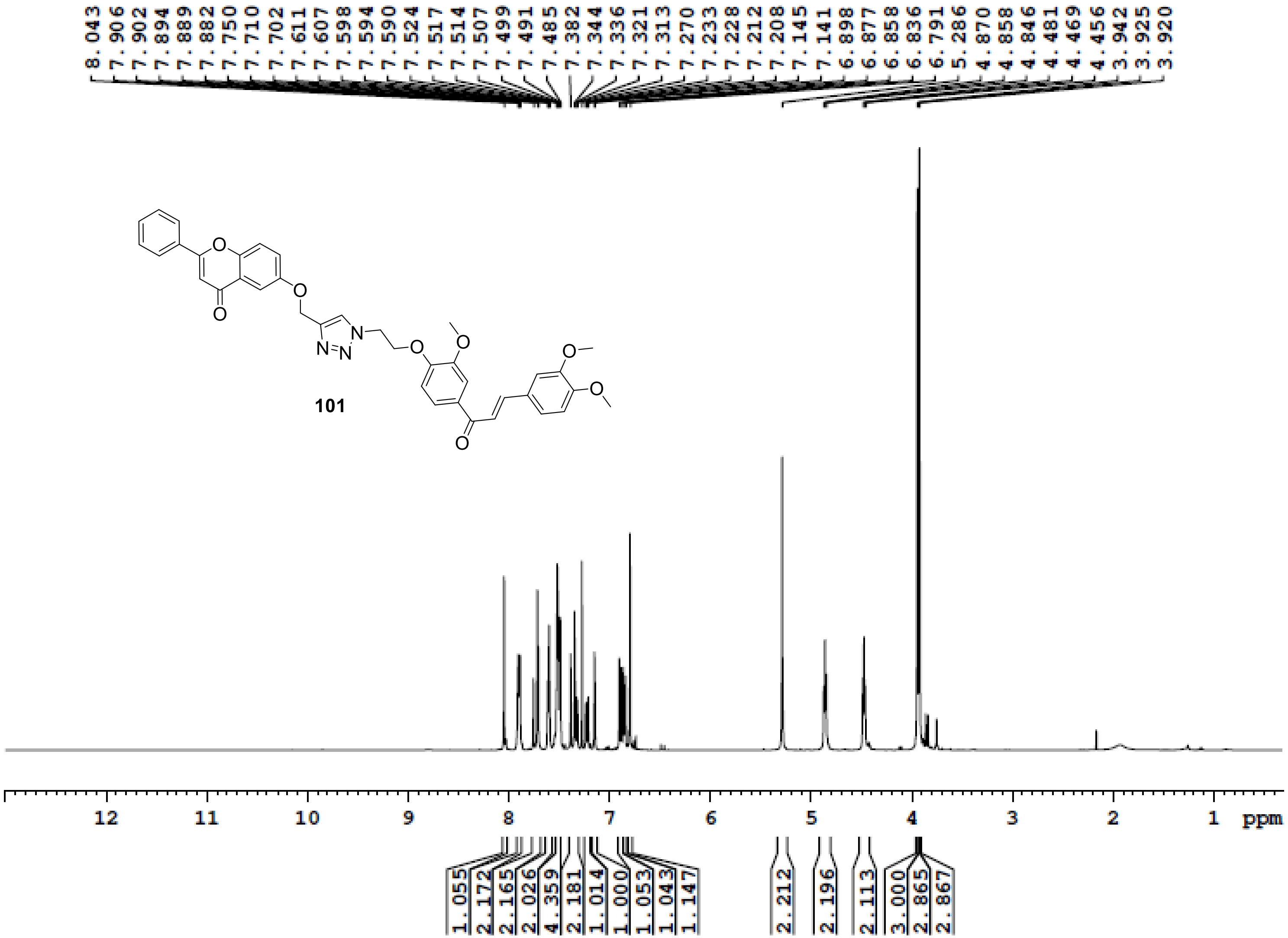
8.043
7.906
7.902
7.894
7.889
7.882
7.750
7.710
7.702
7.611
7.607
7.598
7.594
7.590
7.524
7.517
7.514
7.507
7.499
7.491
7.485
7.382
7.344
7.336
7.321
7.313
7.270
7.233
7.228
7.212
7.208
7.145
7.141
6.898
6.877
6.858
6.836
6.791
5.286
4.870
4.858
4.846
4.481
4.469
4.456
3.942
3.925
3.920
101
12
11
10
9
8
7
6
5
4
3
2
1
ppm
1.055
2.172
2.165
2.026
4.359
2.181
1.014
1.000
1.053
1.043
1.147
2.212
2.196
2.113
3.000
2.865
2.867

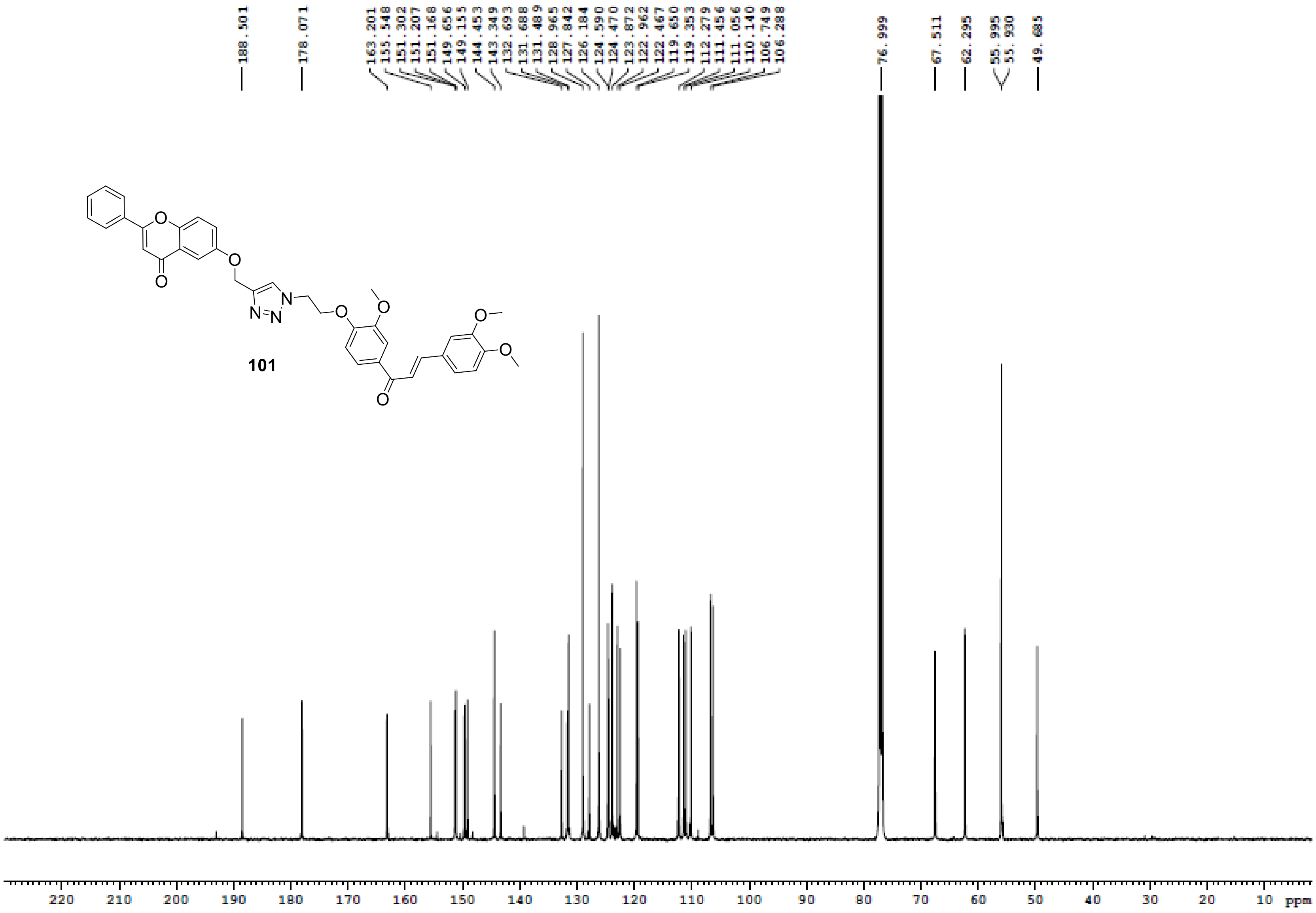
188.501
178.071
163.201
155.548
151.302
151.207
151.168
149.656
149.155
144.453
143.349
132.693
131.688
131.489
128.965
127.842
126.184
124.590
124.470
123.872
122.962
122.467
119.650
119.353
112.279
111.456
111.056
110.140
106.749
106.288
76.999
67.511
62.295
55.995
55.930
49.685
101
220 210 200 190 180 170 160 150 140 130 120 110 100 90 80 70 60 50 40 30 20 10 ppm

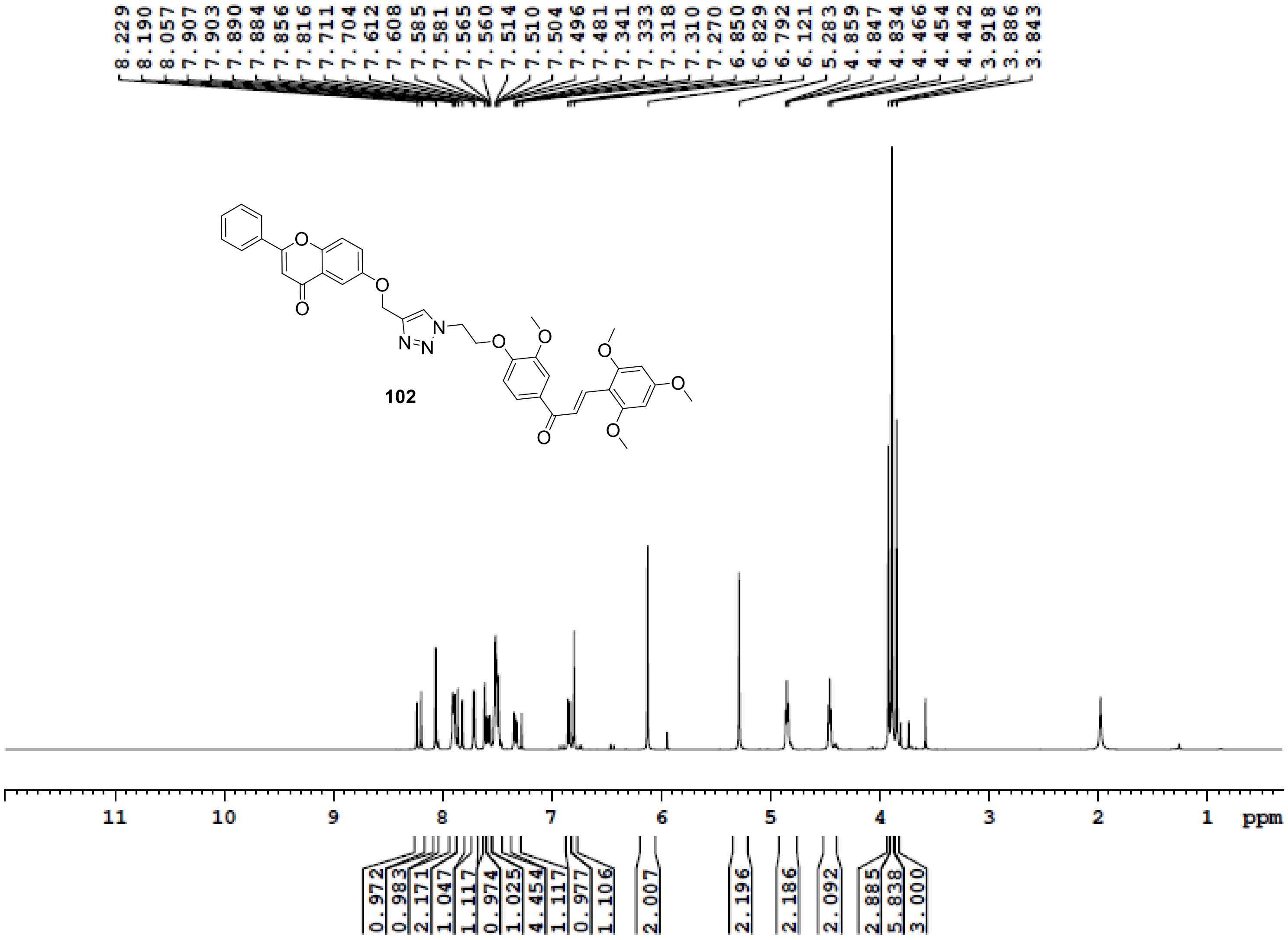
8.229
8.190
8.057
7.907
7.903
7.890
7.884
7.856
7.816
7.711
7.704
7.612
7.608
7.585
7.581
7.565
7.560
7.514
7.510
7.504
7.496
7.481
7.341
7.333
7.318
7.310
7.270
6.850
6.829
6.792
6.121
5.283
4.859
4.847
4.834
4.466
4.454
4.442
3.918
3.886
3.843
102
11
10
9
8
7
6
5
4
3
2
1
ppm
0.972
0.983
2.171
1.047
1.117
0.974
1.025
4.454
1.117
0.977
1.106
2.007
2.196
2.186
2.092
2.885
5.838
3.000

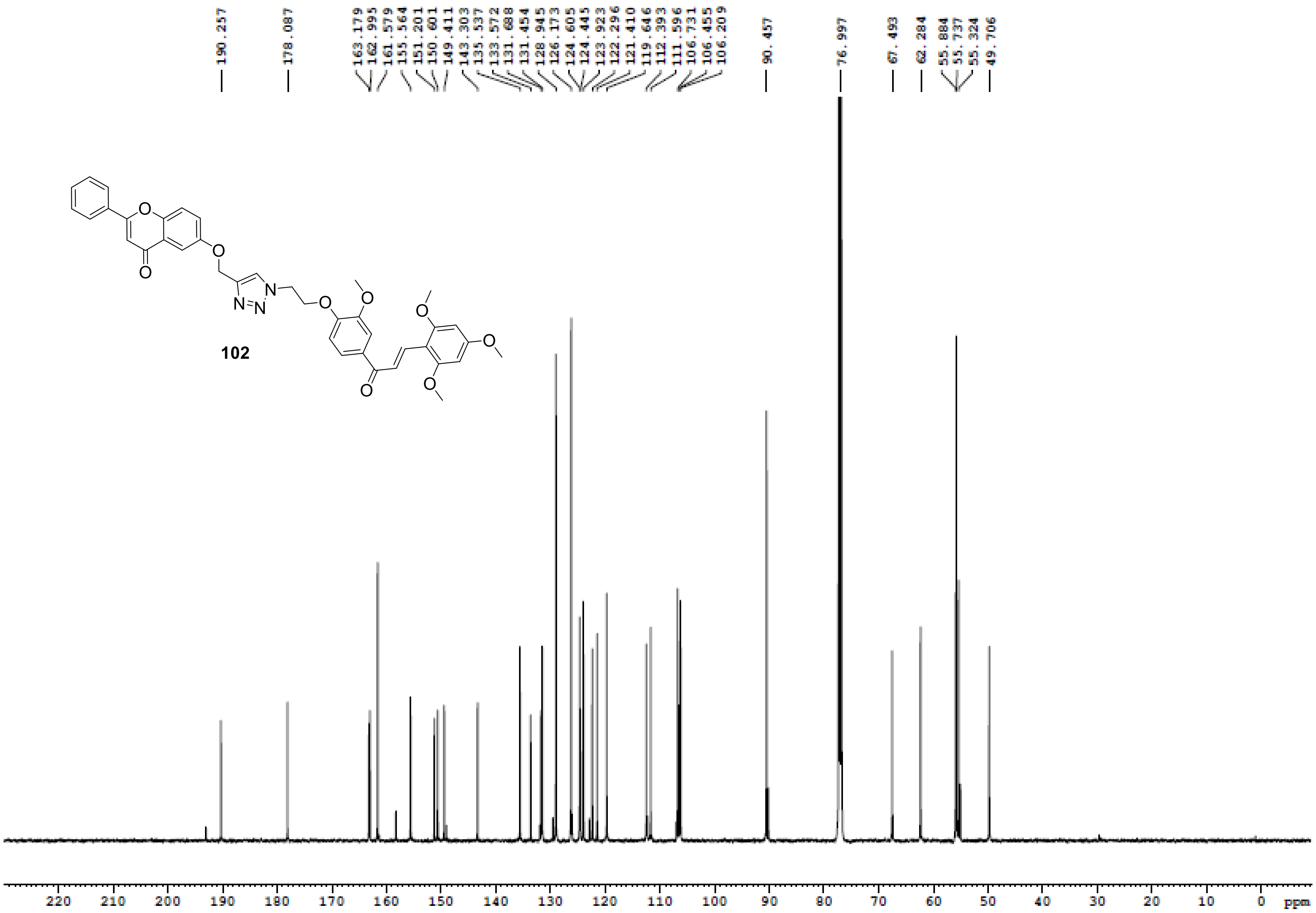
190.257
178.087
163.179
162.995
161.579
155.564
151.201
150.601
149.411
143.303
135.537
133.572
131.688
131.454
128.945
126.173
124.605
124.445
123.923
122.296
121.410
119.646
112.393
111.596
106.731
106.455
106.209
90.457
76.997
67.493
62.284
55.884
55.737
55.324
49.706
102
220 210 200 190 180 170 160 150 140 130 120 110 100 90 80 70 60 50 40 30 20 10 0 ppm

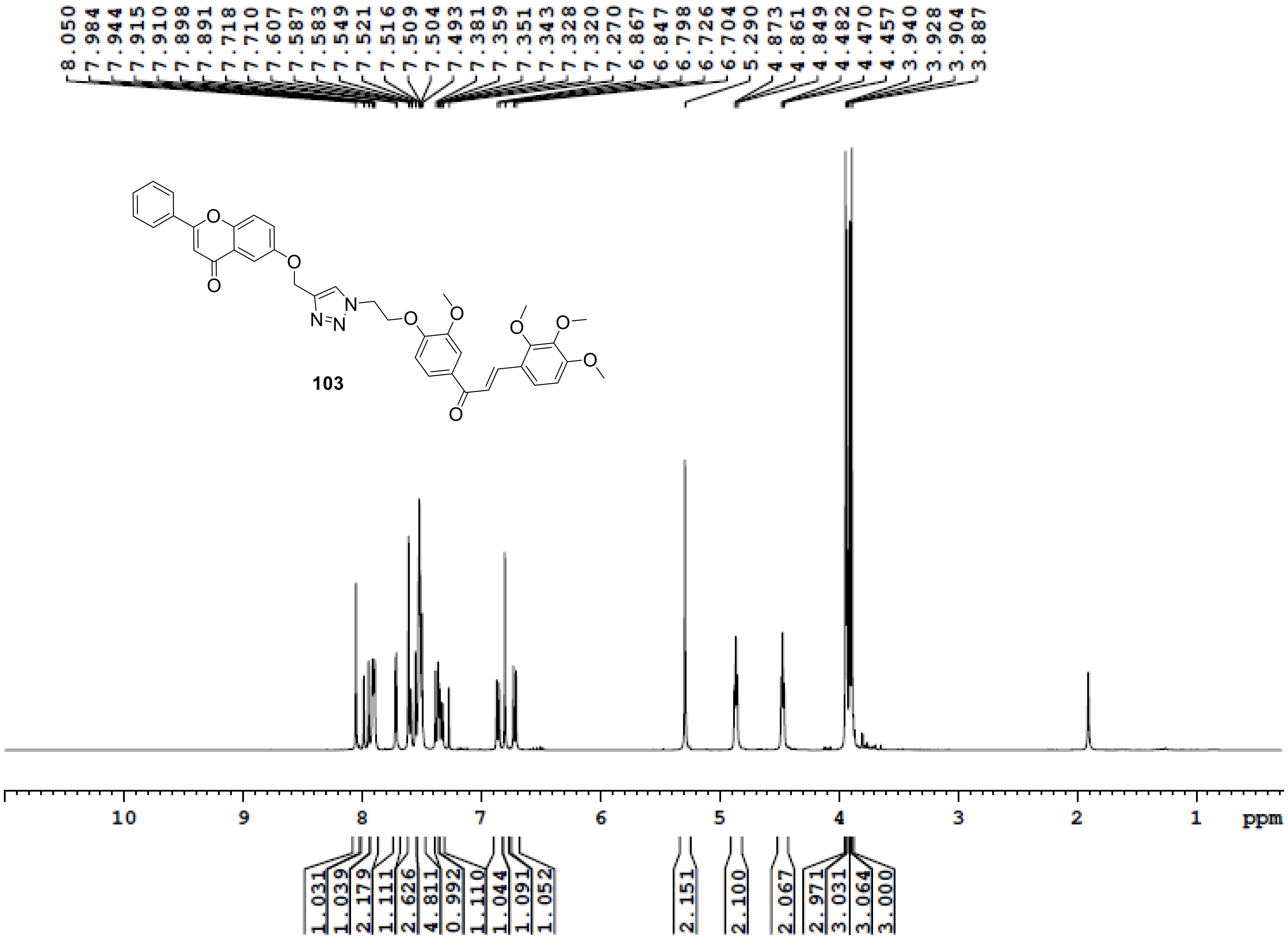
8.050
7.984
7.944
7.915
7.910
7.898
7.891
7.718
7.710
7.607
7.587
7.583
7.549
7.521
7.516
7.509
7.504
7.493
7.381
7.359
7.351
7.343
7.328
7.320
7.270
6.867
6.847
6.798
6.726
6.704
5.290
4.873
4.861
4.849
4.482
4.470
4.457
3.940
3.928
3.904
3.887
103
10
9
8
7
6
5
4
3
2
1
ppm
1.031
1.039
2.179
1.111
2.626
4.811
0.992
1.110
1.044
1.091
1.052
2.151
2.100
2.067
2.971
3.031
3.064
3.000

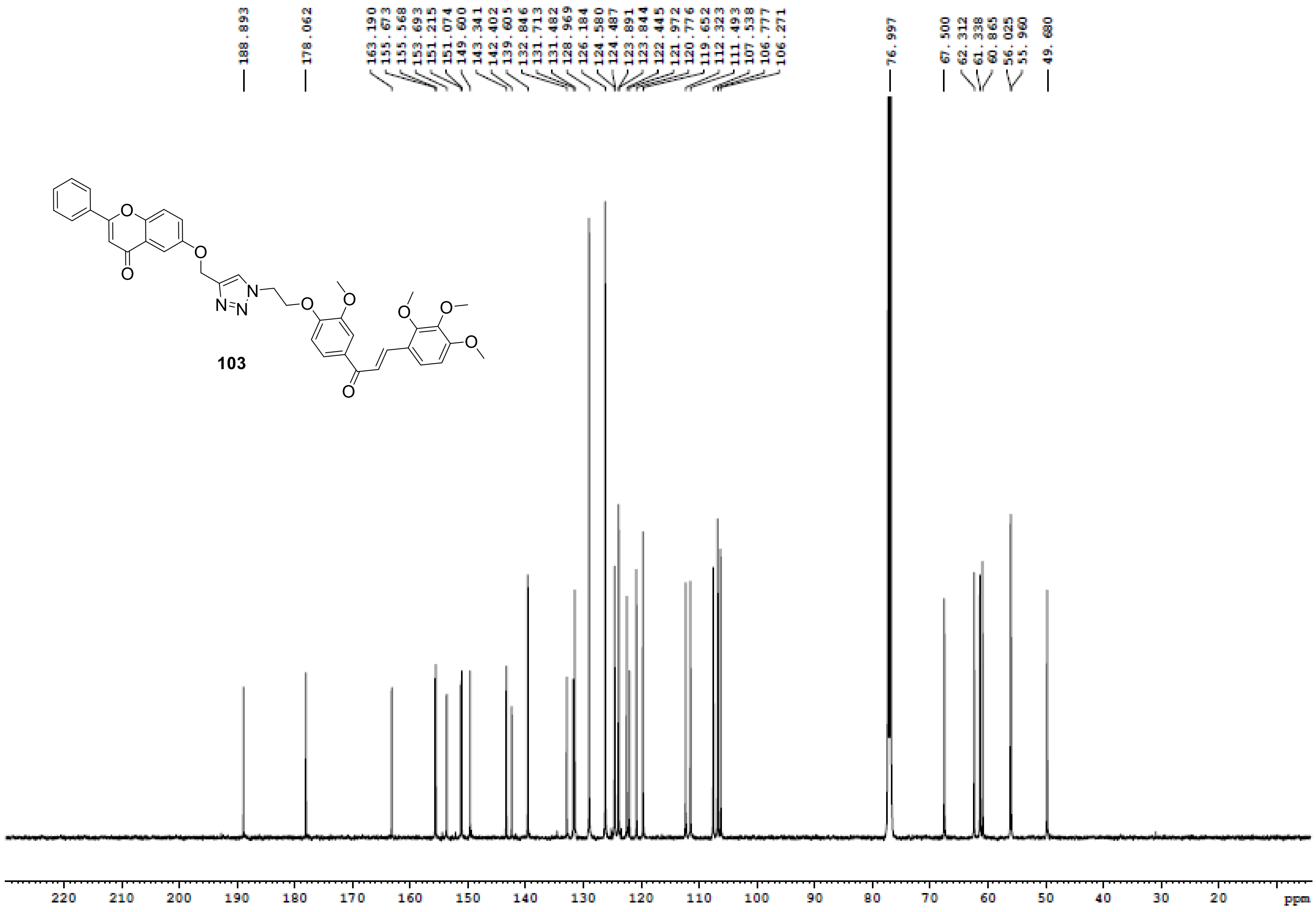
188.893
178.062
163.190
155.673
155.568
153.693
151.215
151.074
149.600
143.341
142.402
139.605
132.846
131.713
131.482
128.969
126.184
124.580
124.487
123.891
123.844
122.445
121.972
120.776
119.652
112.323
111.493
107.538
106.777
106.271
76.997
67.500
62.312
61.338
60.865
56.025
55.960
49.680
103
220 210 200 190 180 170 160 150 140 130 120 110 100 90 80 70 60 50 40 30 20 ppm

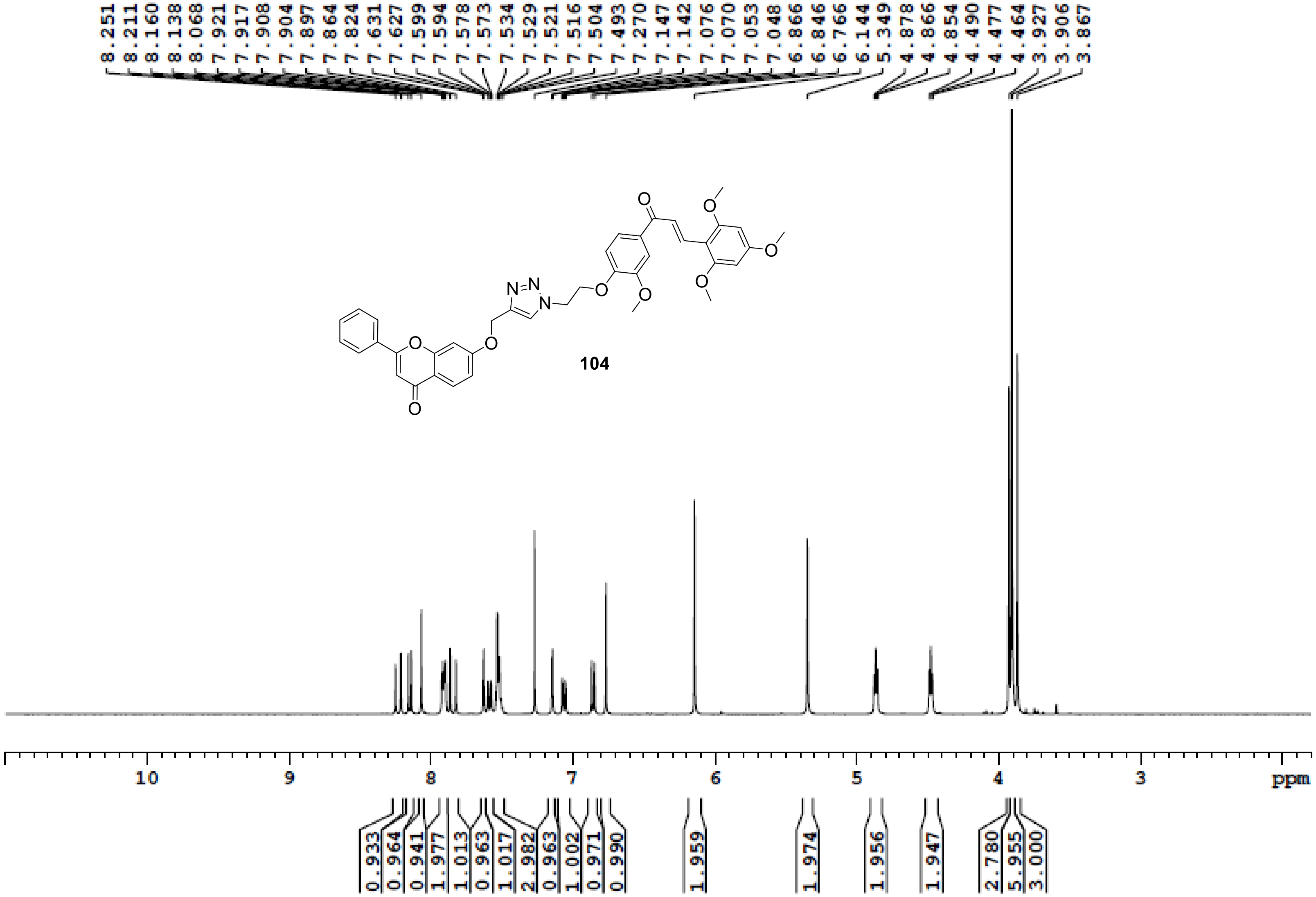
8.251 8.211 8.160 8.138 8.068 7.921 7.917 7.908 7.904 7.897 7.864 7.824 7.631 7.627 7.599 7.594 7.578 7.573 7.534 7.529 7.521 7.516 7.504 7.493 7.270 7.147 7.142 7.076 7.070 7.053 7.048 6.866 6.846 6.766 6.144 5.349 4.878 4.866 4.854 4.490 4.477 4.464 3.927 3.906 3.867
104
10 9 8 7 6 5 4 3 ppm
0.933 0.964 0.941 1.977 1.013 0.963 1.017 2.982 0.963 1.002 0.971 0.990 1.959 1.974 1.956 1.947 2.780 5.955 3.000

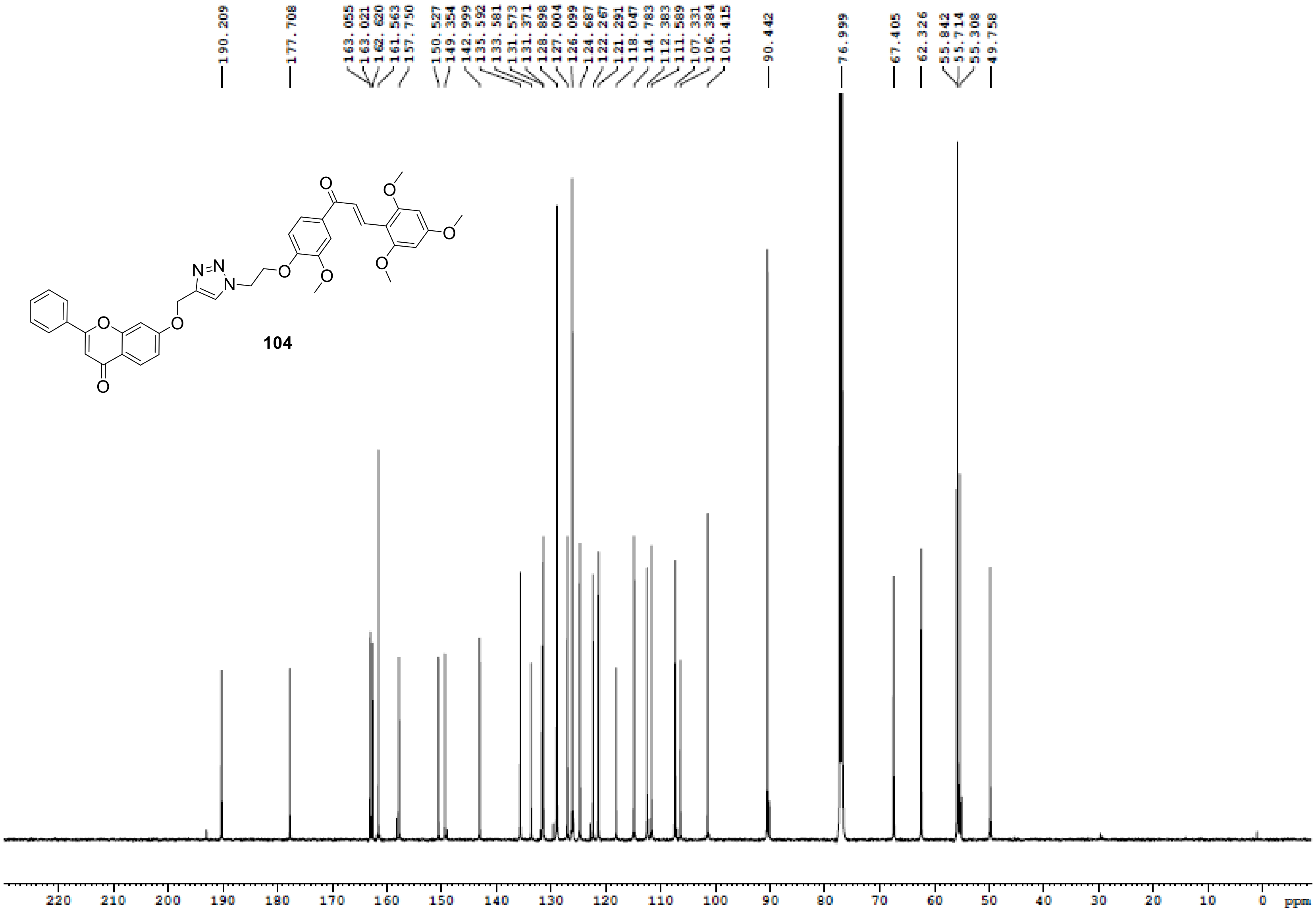
190.209
177.708
163.055
163.021
162.620
161.563
157.750
150.527
149.354
142.999
135.592
133.581
131.573
131.371
128.898
127.004
126.099
124.687
122.267
121.291
118.047
114.783
112.383
111.589
107.331
106.384
101.415
90.442
76.999
67.405
62.326
55.842
55.714
55.308
49.758
104
220 210 200 190 180 170 160 150 140 130 120 110 100 90 80 70 60 50 40 30 20 10 0 ppm

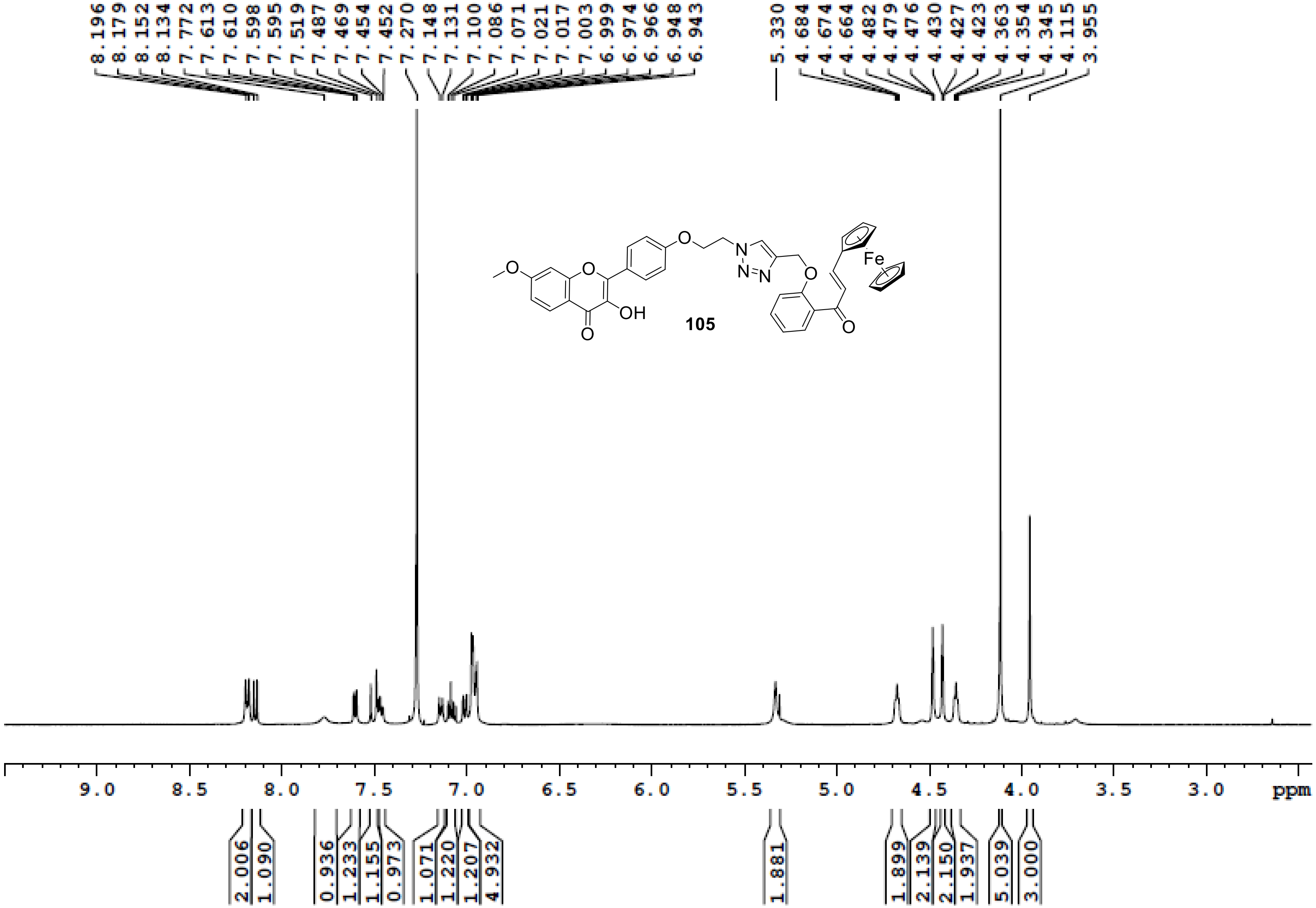
8.196 8.179 8.152 8.134 7.772 7.613 7.610 7.598 7.595 7.519 7.487 7.469 7.454 7.452 7.270 7.148 7.131 7.100 7.086 7.071 7.021 7.017 7.003 6.999 6.974 6.966 6.948 6.943
5.330 4.684 4.674 4.664 4.482 4.479 4.476 4.430 4.427 4.423 4.363 4.354 4.345 4.115 3.955
Fe
OH
105
9.0 8.5 8.0 7.5 7.0 6.5 6.0 5.5 5.0 4.5 4.0 3.5 3.0 ppm
2.006 1.090 0.936 1.233 1.155 0.973 1.071 1.220 1.207 4.932 1.881 1.899 2.139 2.150 1.937 5.039 3.000

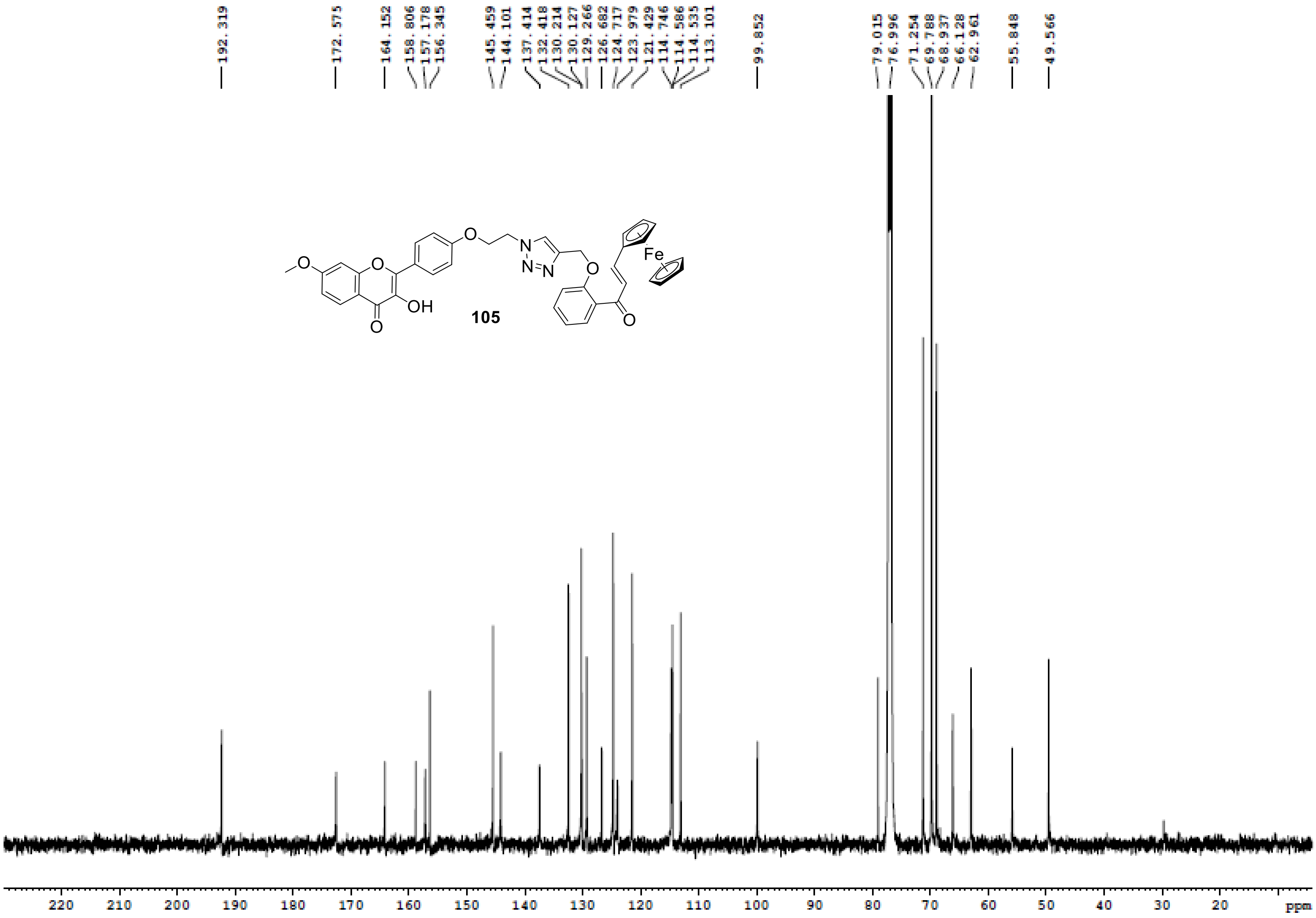
192.319
172.575
164.152
158.806
157.178
156.345
145.459
144.101
137.414
132.418
130.214
130.127
129.266
126.682
124.717
123.979
121.429
114.746
114.586
114.535
113.101
99.852
79.015
76.996
71.254
69.788
68.937
66.128
62.961
55.848
49.566
105
Fe
OH
220 210 200 190 180 170 160 150 140 130 120 110 100 90 80 70 60 50 40 30 20 ppm

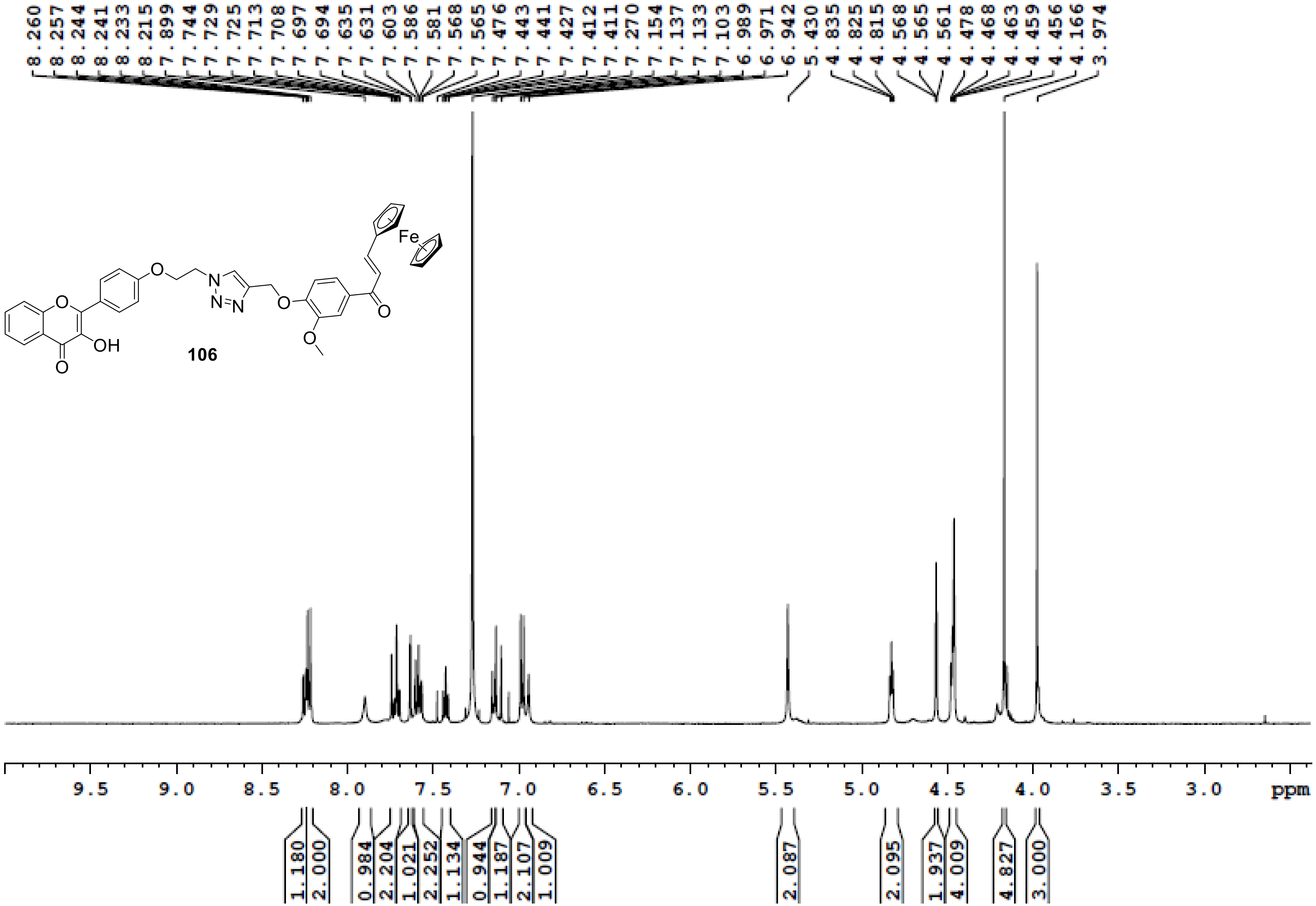
8.260
8.257
8.244
8.241
8.233
8.215
7.899
7.744
7.729
7.725
7.713
7.708
7.697
7.694
7.635
7.631
7.603
7.586
7.581
7.568
7.565
7.476
7.443
7.441
7.427
7.412
7.411
7.270
7.154
7.137
7.133
7.103
6.989
6.971
6.942
5.430
4.835
4.825
4.815
4.568
4.565
4.561
4.478
4.468
4.463
4.459
4.456
4.166
3.974
Fe
O
OH
N
O
106
9.5
9.0
8.5
8.0
7.5
7.0
6.5
6.0
5.5
5.0
4.5
4.0
3.5
3.0
ppm
1.180
2.000
0.984
2.204
1.021
2.252
1.134
0.944
1.187
2.107
1.009
2.087
2.095
1.937
4.009
4.827
3.000

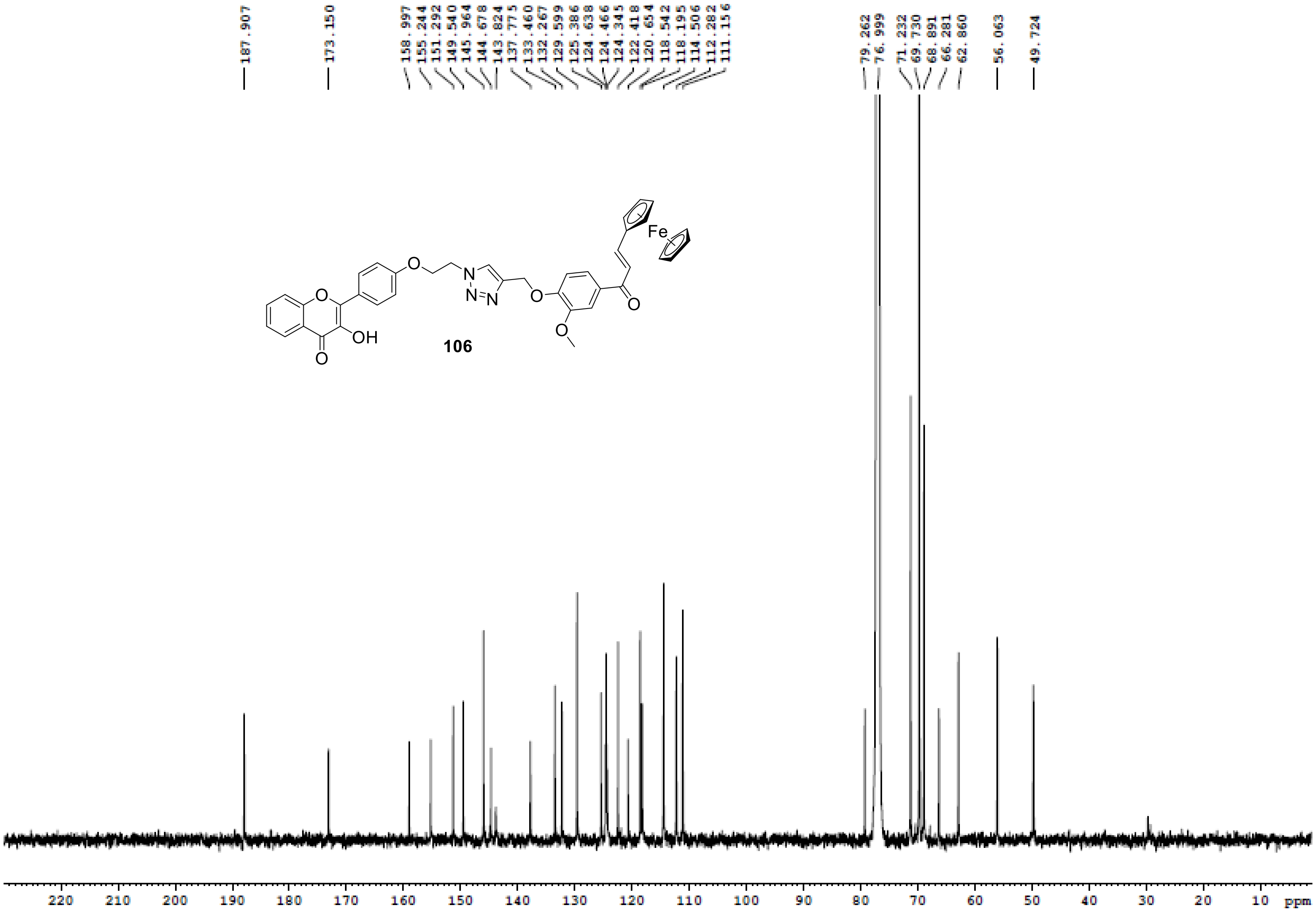
187.907
173.150
158.997
155.244
151.292
149.540
145.964
144.678
143.824
137.775
133.460
132.267
129.599
125.386
124.638
124.466
124.345
122.418
120.654
118.542
118.195
114.506
112.282
111.156
79.262
76.999
71.232
69.730
68.891
66.281
62.860
56.063
49.724
106
Fe
OH
220 210 200 190 180 170 160 150 140 130 120 110 100 90 80 70 60 50 40 30 20 10 ppm

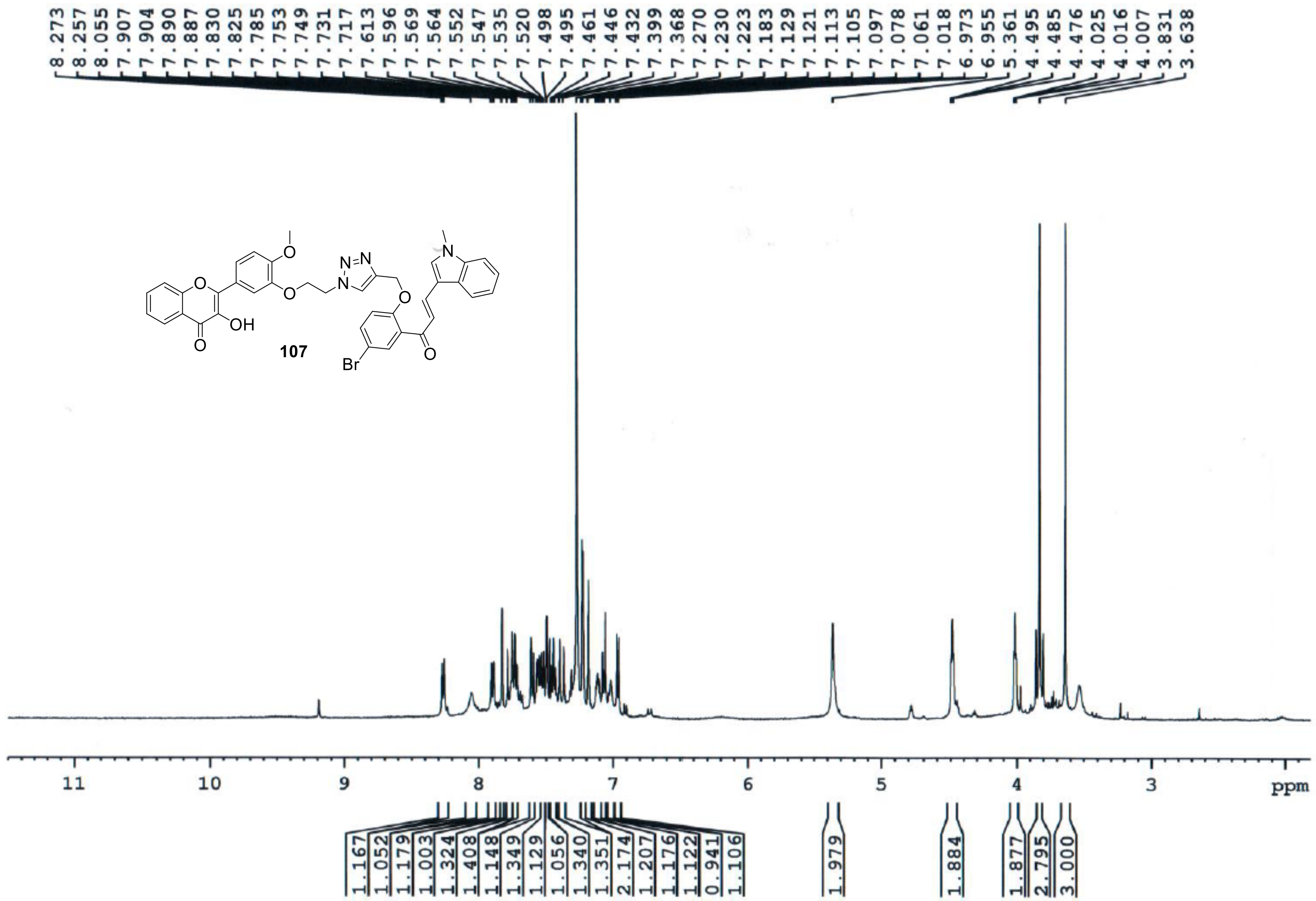
8.273 8.257 8.055 7.907 7.904 7.890 7.887 7.830 7.825 7.785 7.753 7.749 7.731 7.717 7.613 7.596 7.569 7.564 7.552 7.547 7.535 7.520 7.498 7.495 7.461 7.446 7.432 7.399 7.368 7.270 7.230 7.223 7.183 7.129 7.121 7.113 7.105 7.097 7.078 7.061 7.018 6.973 6.955 5.361 4.495 4.485 4.476 4.025 4.016 4.007 3.831 3.638
107
OH
Br
11 10 9 8 7 6 5 4 3 ppm
1.167 1.052 1.179 1.003 1.324 1.408 1.148 1.349 1.129 1.056 1.340 1.351 2.174 1.207 1.176 1.122 0.941 1.106 1.979 1.884 1.877 2.795 3.000

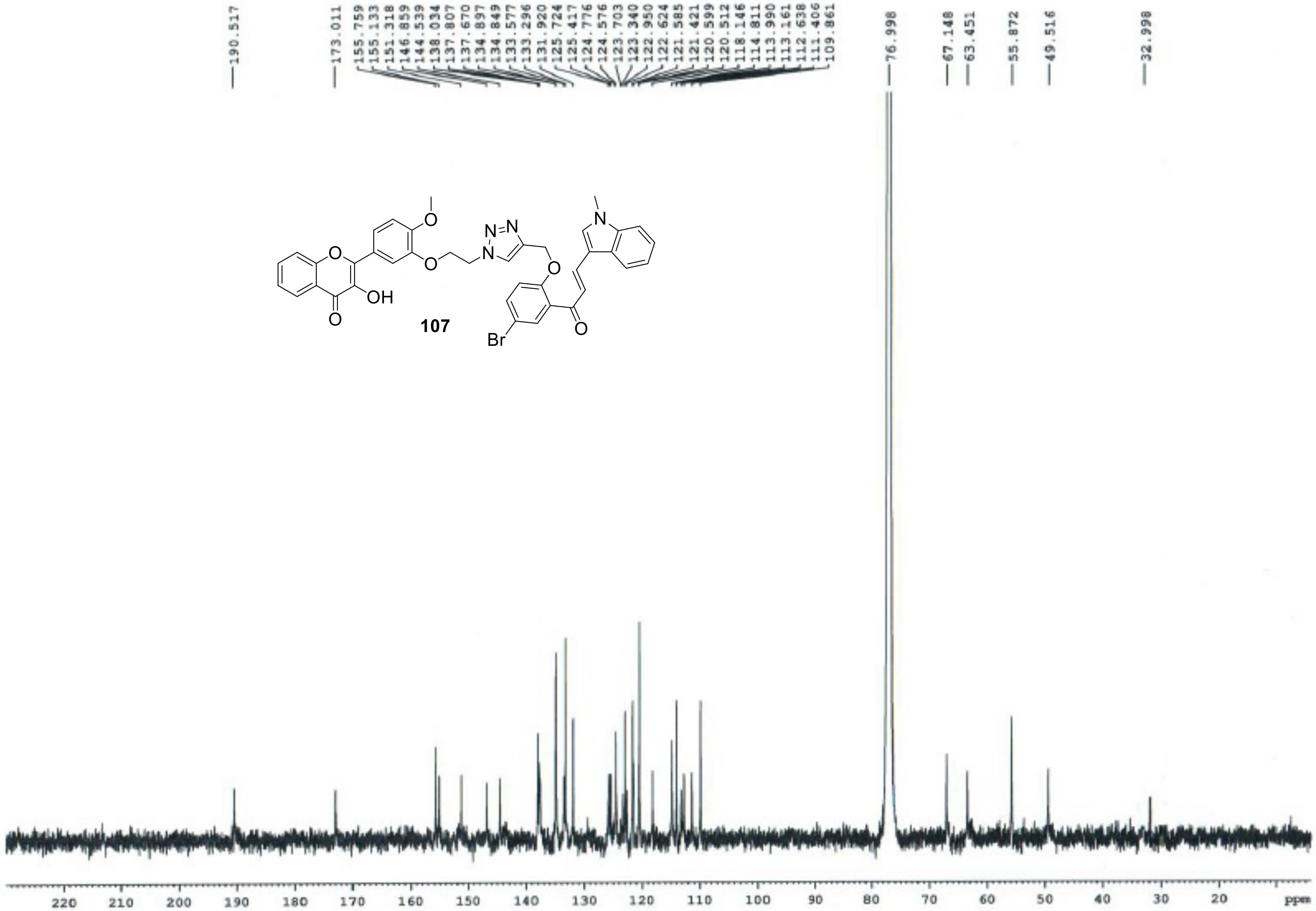
190.517
173.011
155.759
155.133
151.318
146.859
144.539
138.034
137.807
137.670
134.897
134.849
133.577
133.296
131.920
125.724
125.417
124.776
124.576
123.703
123.340
122.950
122.624
121.585
121.421
120.599
120.512
118.146
114.811
113.990
113.161
112.638
111.406
109.861
76.998
67.148
63.451
55.872
49.516
32.998
107
OH
Br
220 210 200 190 180 170 160 150 140 130 120 110 100 90 80 70 60 50 40 30 20 ppm

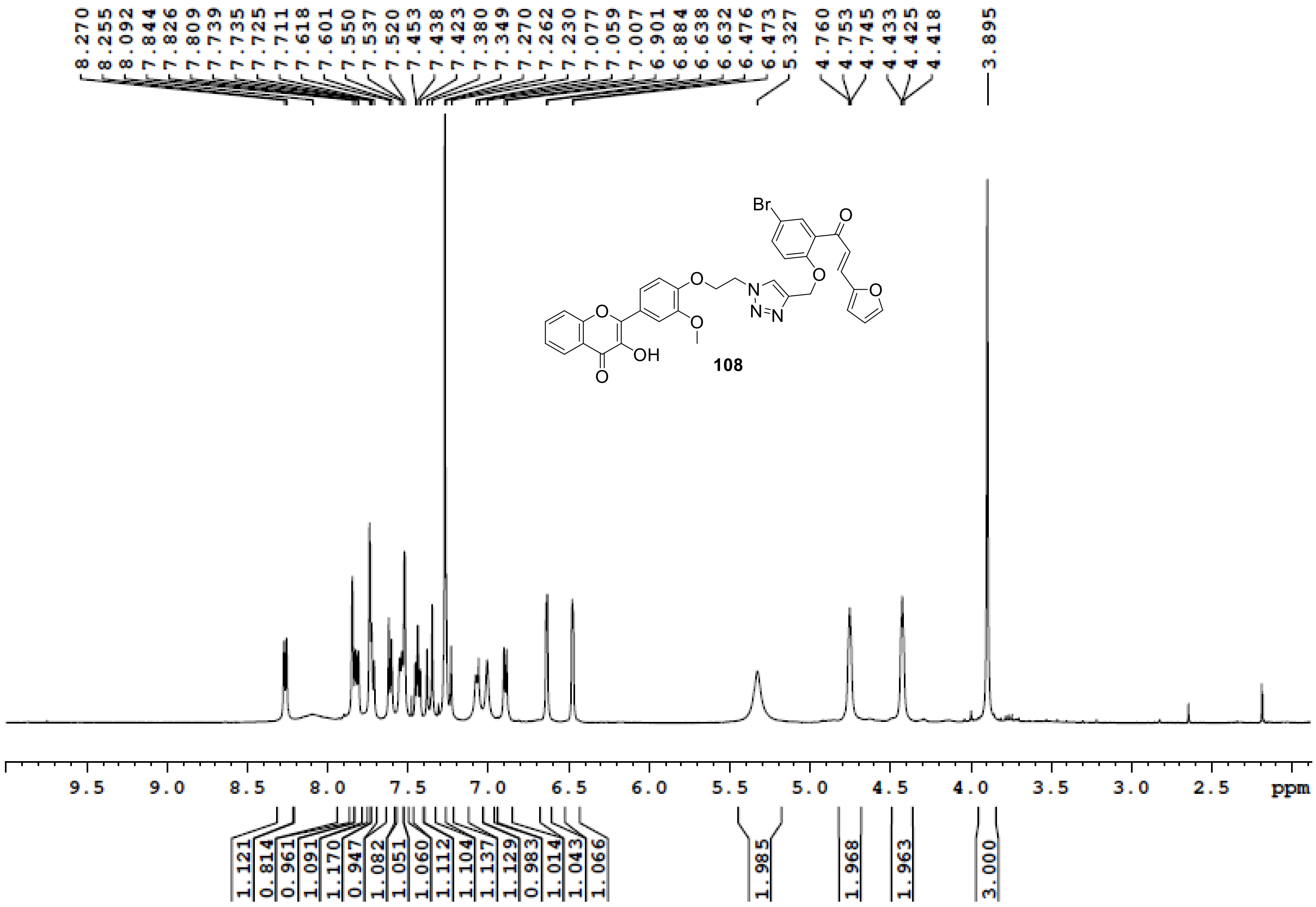
8.270
8.255
8.092
7.844
7.826
7.809
7.739
7.735
7.725
7.711
7.618
7.601
7.550
7.537
7.520
7.453
7.438
7.423
7.380
7.349
7.270
7.262
7.230
7.077
7.059
7.007
6.901
6.884
6.638
6.632
6.476
6.473
5.327
4.760
4.753
4.745
4.433
4.425
4.418
3.895
Br
O
O
O
N
N
N
O
O
O
OH
O
O
108
9.5
9.0
8.5
8.0
7.5
7.0
6.5
6.0
5.5
5.0
4.5
4.0
3.5
3.0
2.5
ppm
1.121
0.814
0.961
1.091
1.170
0.947
1.082
1.051
1.060
1.112
1.104
1.137
1.129
0.983
1.014
1.043
1.066
1.985
1.968
1.963
3.000

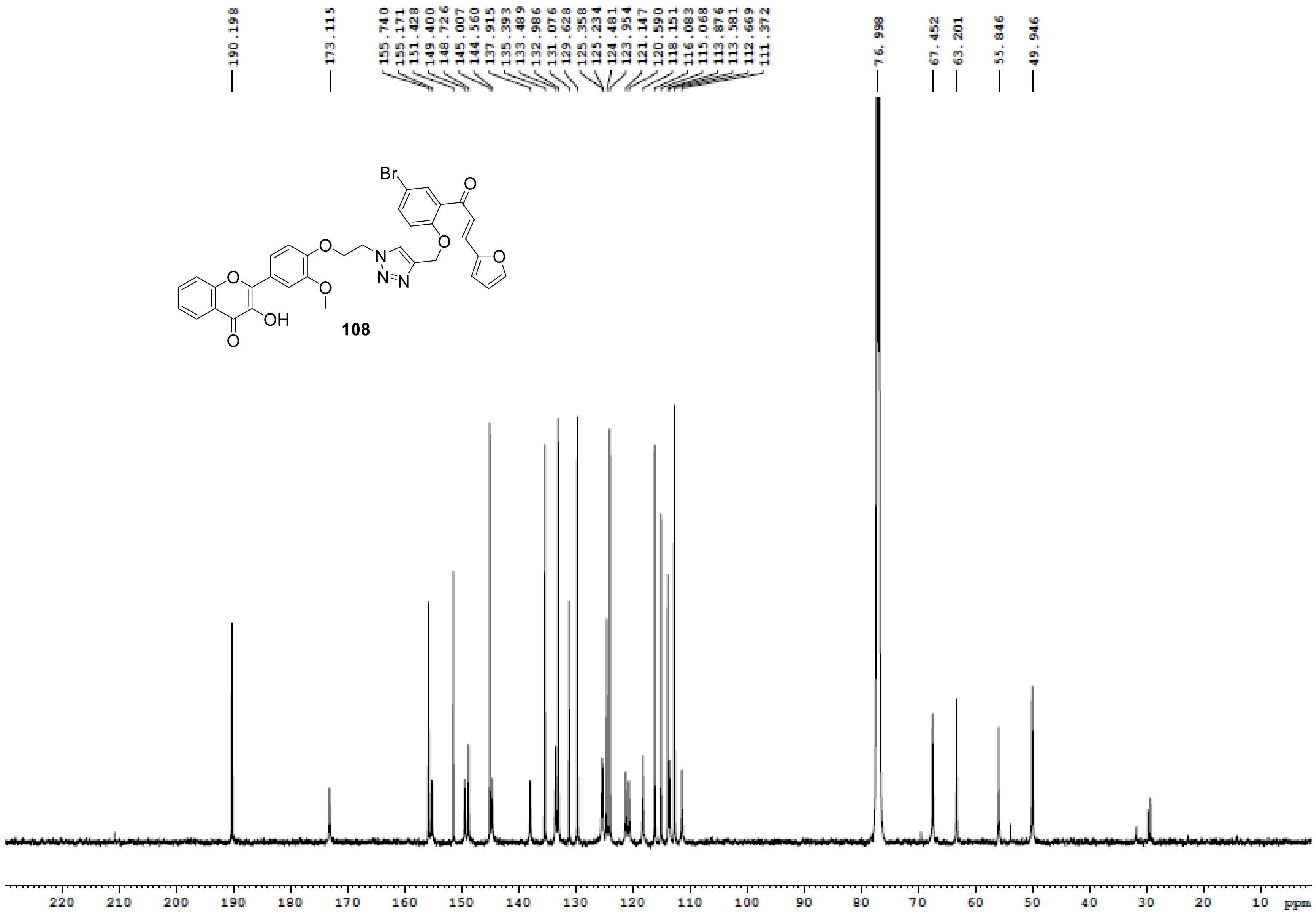
190.198
173.115
155.740
155.171
151.428
149.400
148.726
145.007
144.560
137.915
135.393
133.489
132.986
131.076
129.628
125.358
125.234
124.481
123.954
121.147
120.590
118.151
116.083
115.068
113.876
113.581
112.669
111.372
76.998
67.452
63.201
55.846
49.946
Br
O
OH
N
108
220 210 200 190 180 170 160 150 140 130 120 110 100 90 80 70 60 50 40 30 20 10 ppm

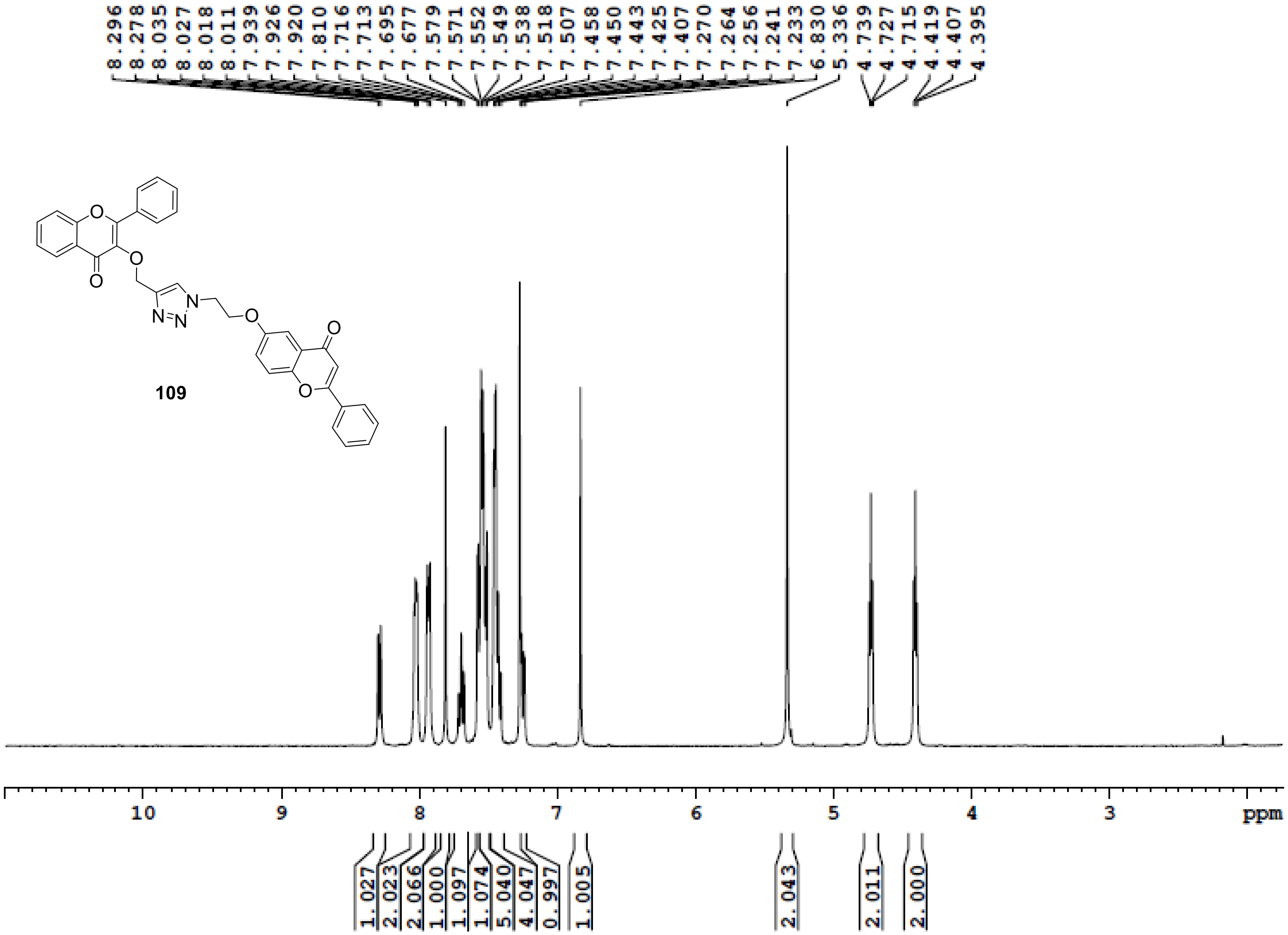
8.296 8.278 8.035 8.027 8.018 8.011 7.939 7.926 7.920 7.810 7.716 7.713 7.695 7.677 7.579 7.571 7.552 7.549 7.538 7.518 7.507 7.458 7.450 7.443 7.425 7.407 7.270 7.264 7.256 7.241 7.233 6.830 5.336 4.739 4.727 4.715 4.419 4.407 4.395
109
10 9 8 7 6 5 4 3 ppm
1.027 2.023 2.066 1.000 1.097 1.074 5.040 4.047 0.997 1.005 2.043 2.011 2.000

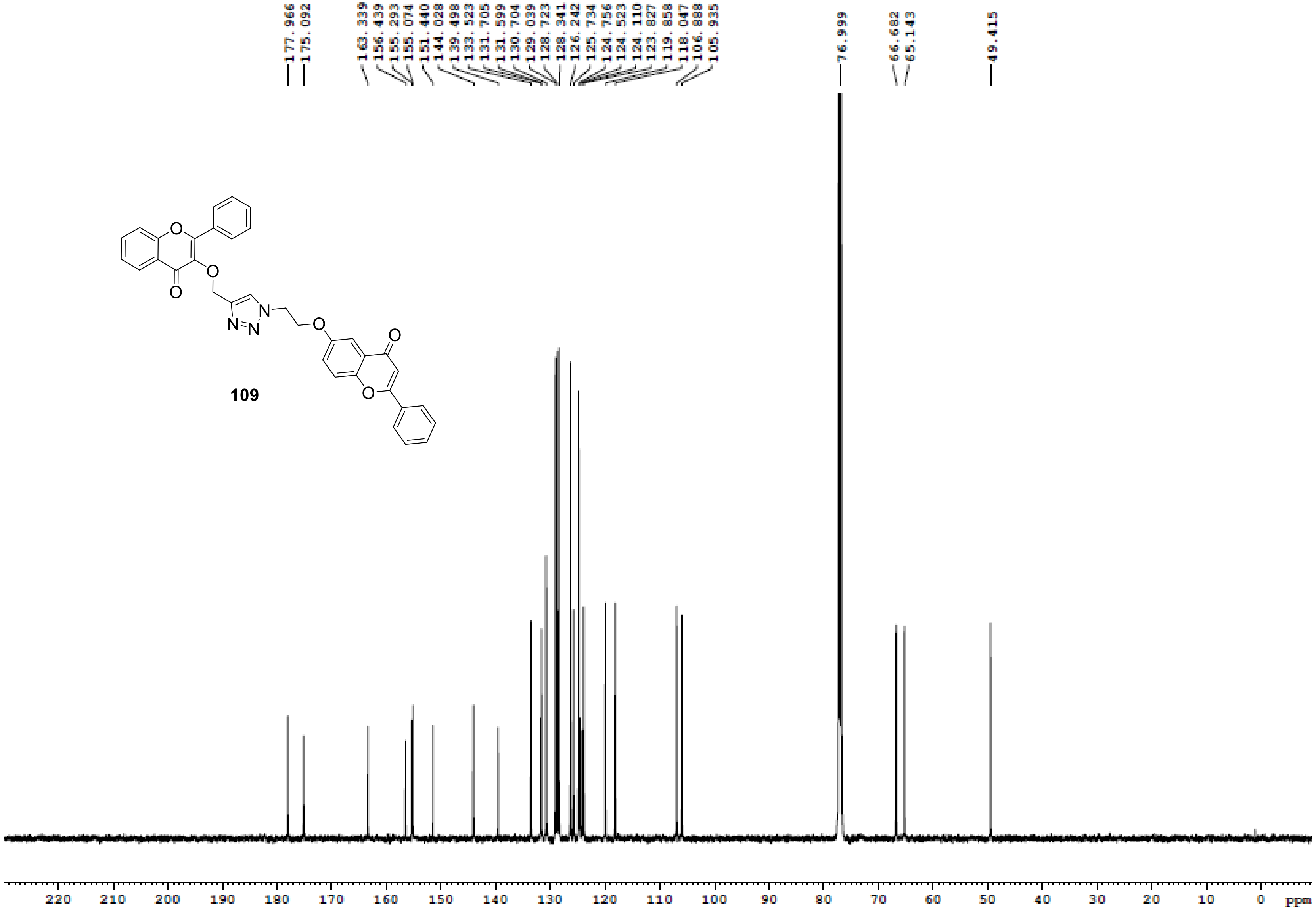
177.966
175.092
163.339
156.439
155.293
155.074
151.440
144.028
139.498
133.523
131.705
131.599
130.704
129.039
128.723
128.341
126.242
125.734
124.756
124.523
124.110
123.827
119.858
118.047
106.888
105.935
76.999
66.682
65.143
49.415
109
220 210 200 190 180 170 160 150 140 130 120 110 100 90 80 70 60 50 40 30 20 10 0 ppm

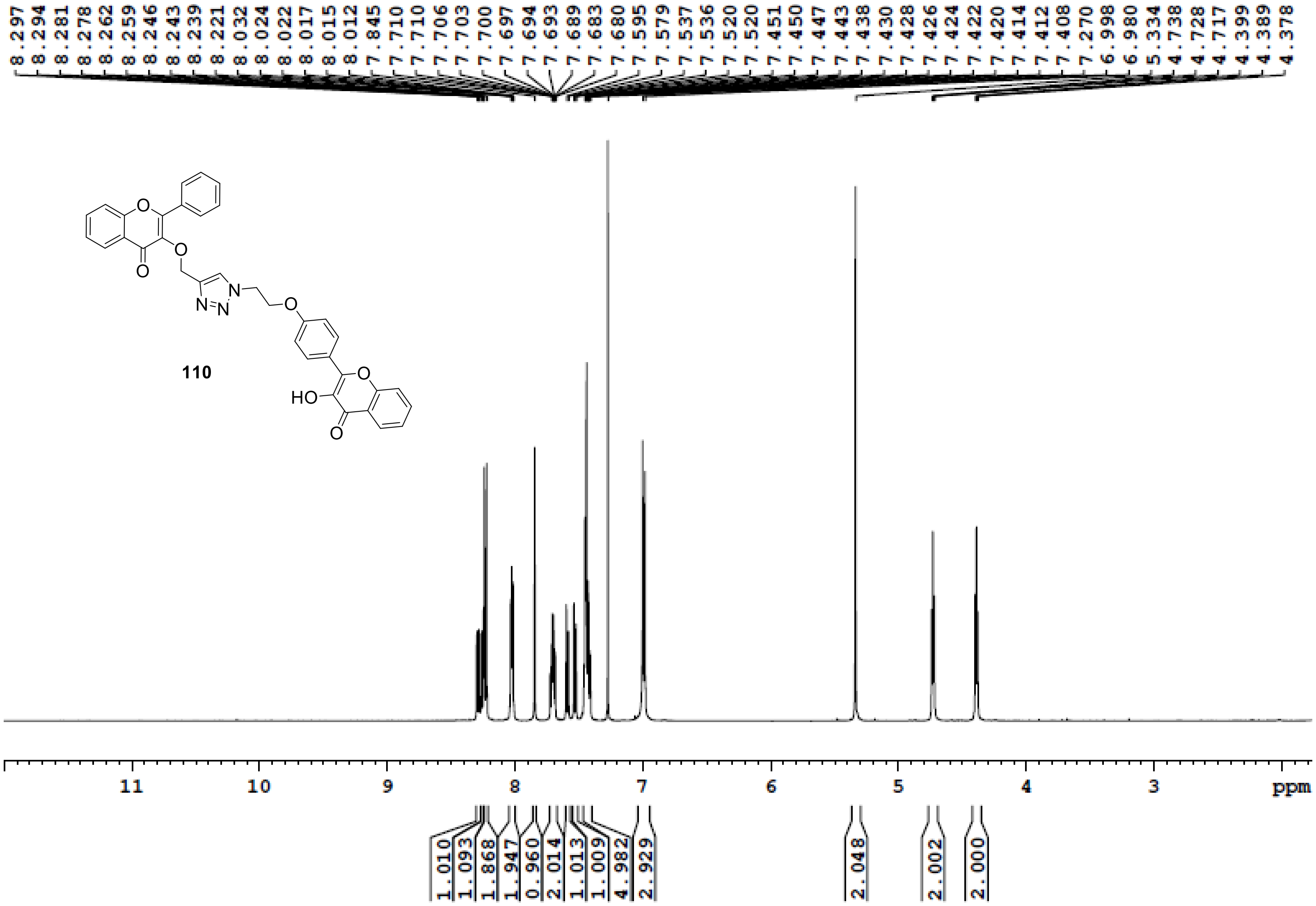
8.297 8.294 8.281 8.278 8.262 8.259 8.246 8.243 8.239 8.221 8.032 8.024 8.022 8.017 8.015 8.012 7.845 7.710 7.710 7.706 7.703 7.700 7.697 7.694 7.693 7.689 7.683 7.680 7.595 7.579 7.537 7.536 7.520 7.520 7.451 7.450 7.447 7.443 7.438 7.430 7.428 7.426 7.424 7.422 7.420 7.414 7.412 7.408 7.270 6.998 6.980 5.334 4.738 4.728 4.717 4.399 4.389 4.378
110
HO
11
10
9
8
7
6
5
4
3
ppm
1.010 1.093 1.868 1.947 0.960 2.014 1.013 1.009 4.982 2.929
2.048 2.002 2.000

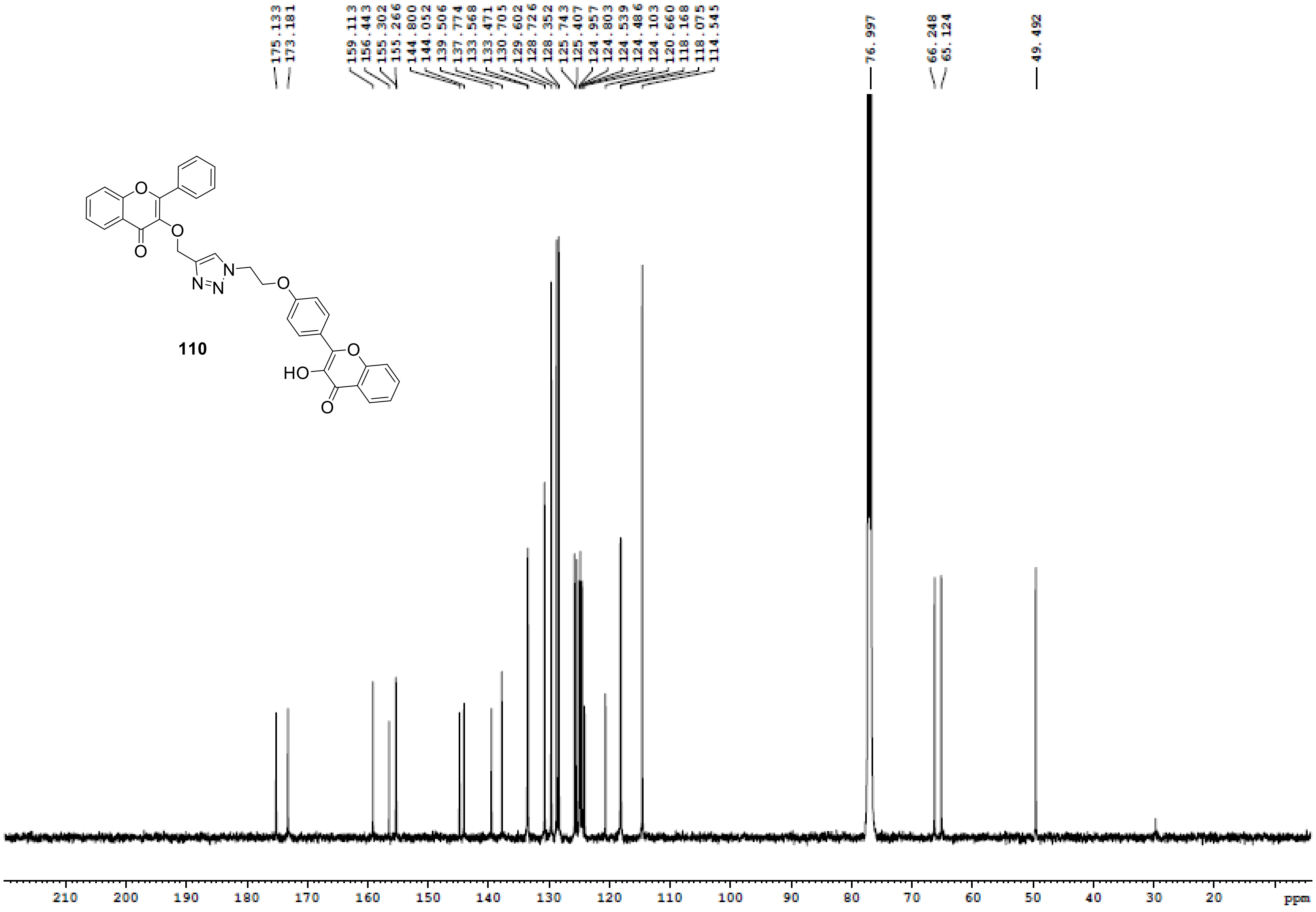
175.133
173.181
159.113
156.443
155.302
155.266
144.800
144.052
139.506
137.774
133.568
133.471
130.705
129.602
128.726
128.352
125.743
125.407
124.957
124.803
124.539
124.486
124.103
120.660
118.168
118.075
114.545
76.997
66.248
65.124
49.492
110
HO
210 200 190 180 170 160 150 140 130 120 110 100 90 80 70 60 50 40 30 20 ppm

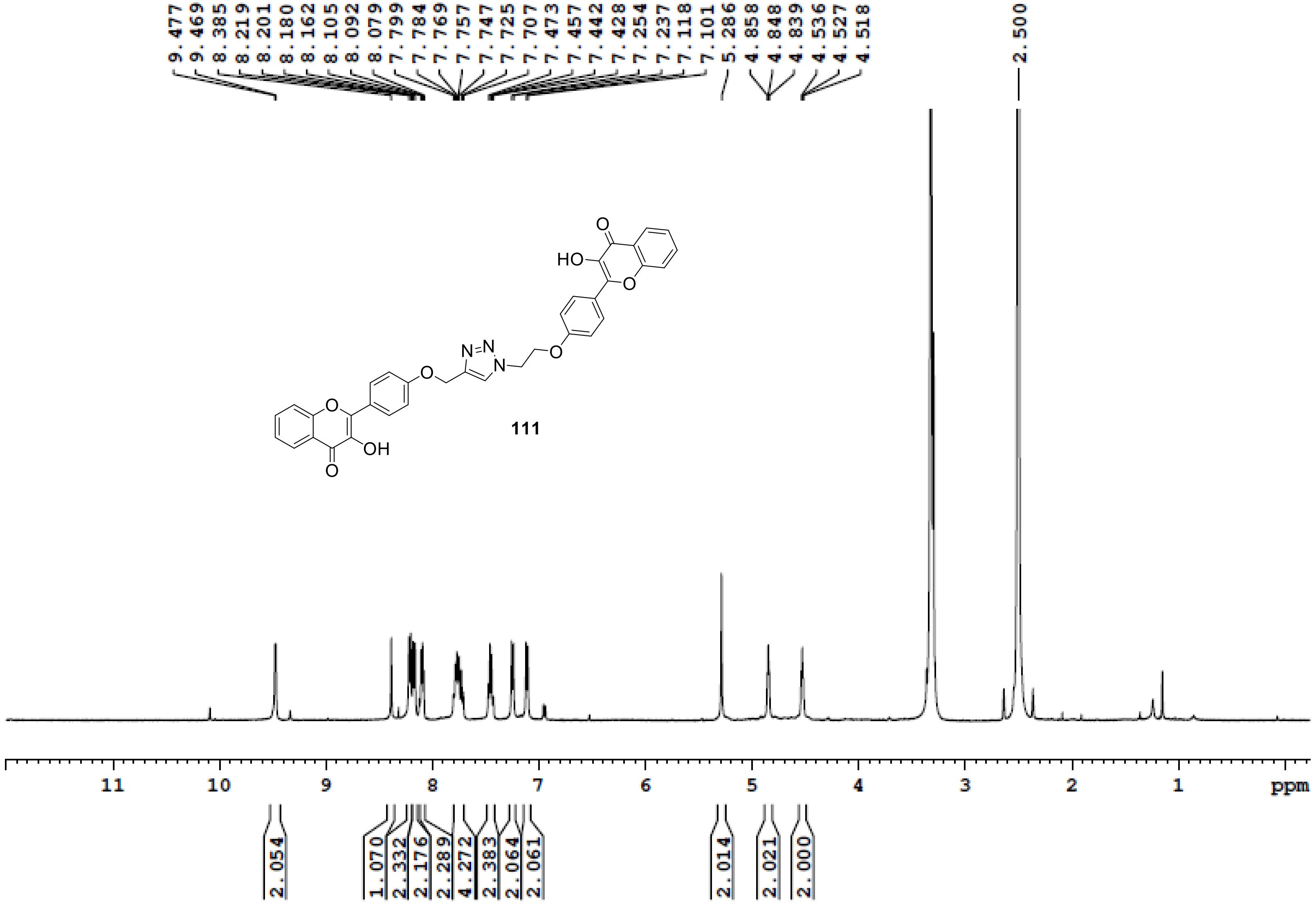
9.477
9.469
8.385
8.219
8.201
8.180
8.162
8.105
8.092
8.079
7.799
7.784
7.769
7.757
7.747
7.725
7.707
7.473
7.457
7.442
7.428
7.254
7.237
7.118
7.101
5.286
4.858
4.848
4.839
4.536
4.527
4.518
2.500
111
ppm
2.054
1.070
2.332
2.176
2.289
4.272
2.383
2.064
2.061
2.014
2.021
2.000

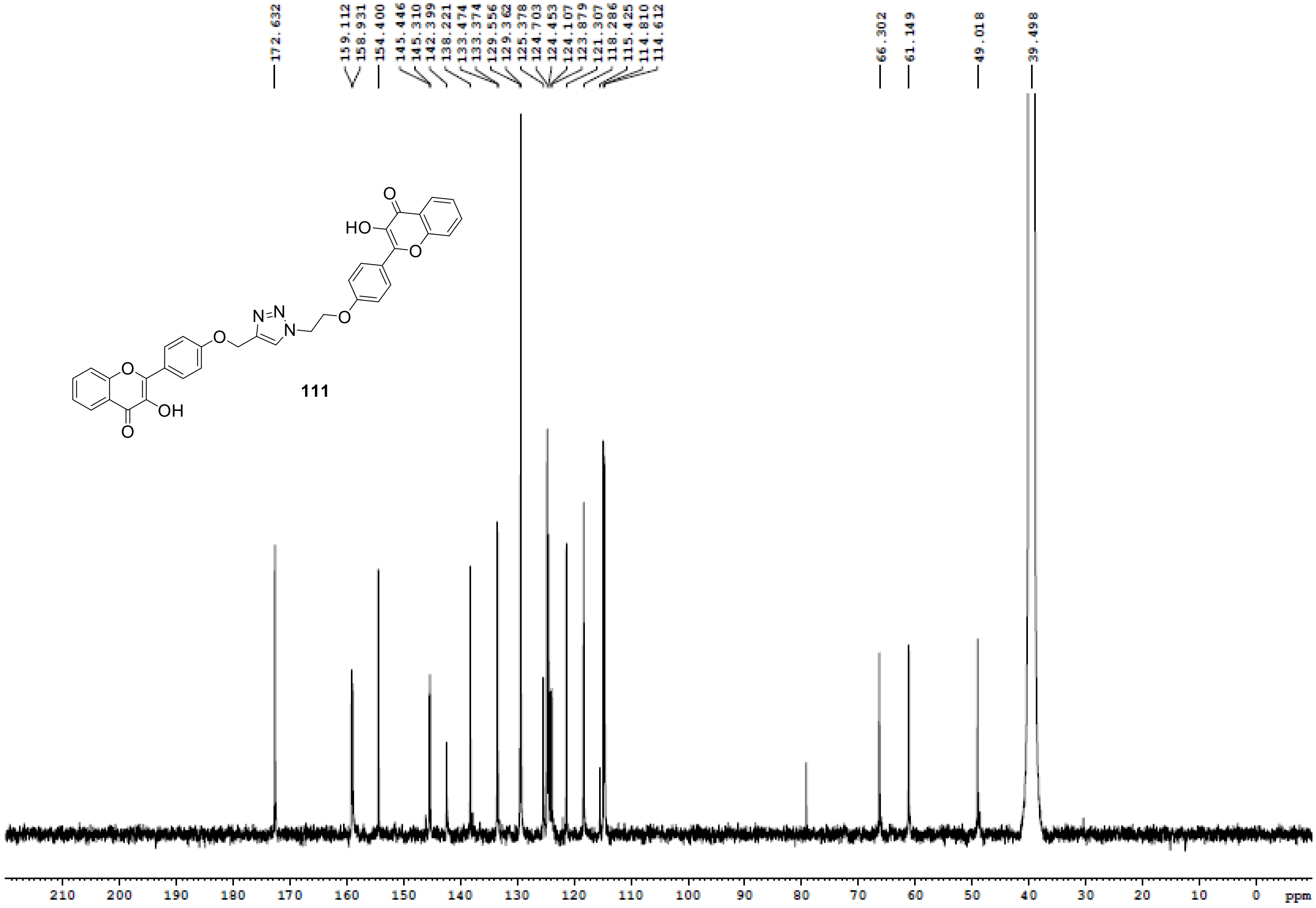
172.632
159.112
158.931
154.400
145.446
145.310
142.399
138.221
133.474
133.374
129.556
129.362
125.378
124.703
124.453
124.107
123.879
121.307
118.286
115.425
114.810
114.612
66.302
61.149
49.018
39.498
HO
OH
O
N
111
210 200 190 180 170 160 150 140 130 120 110 100 90 80 70 60 50 40 30 20 10 0 ppm

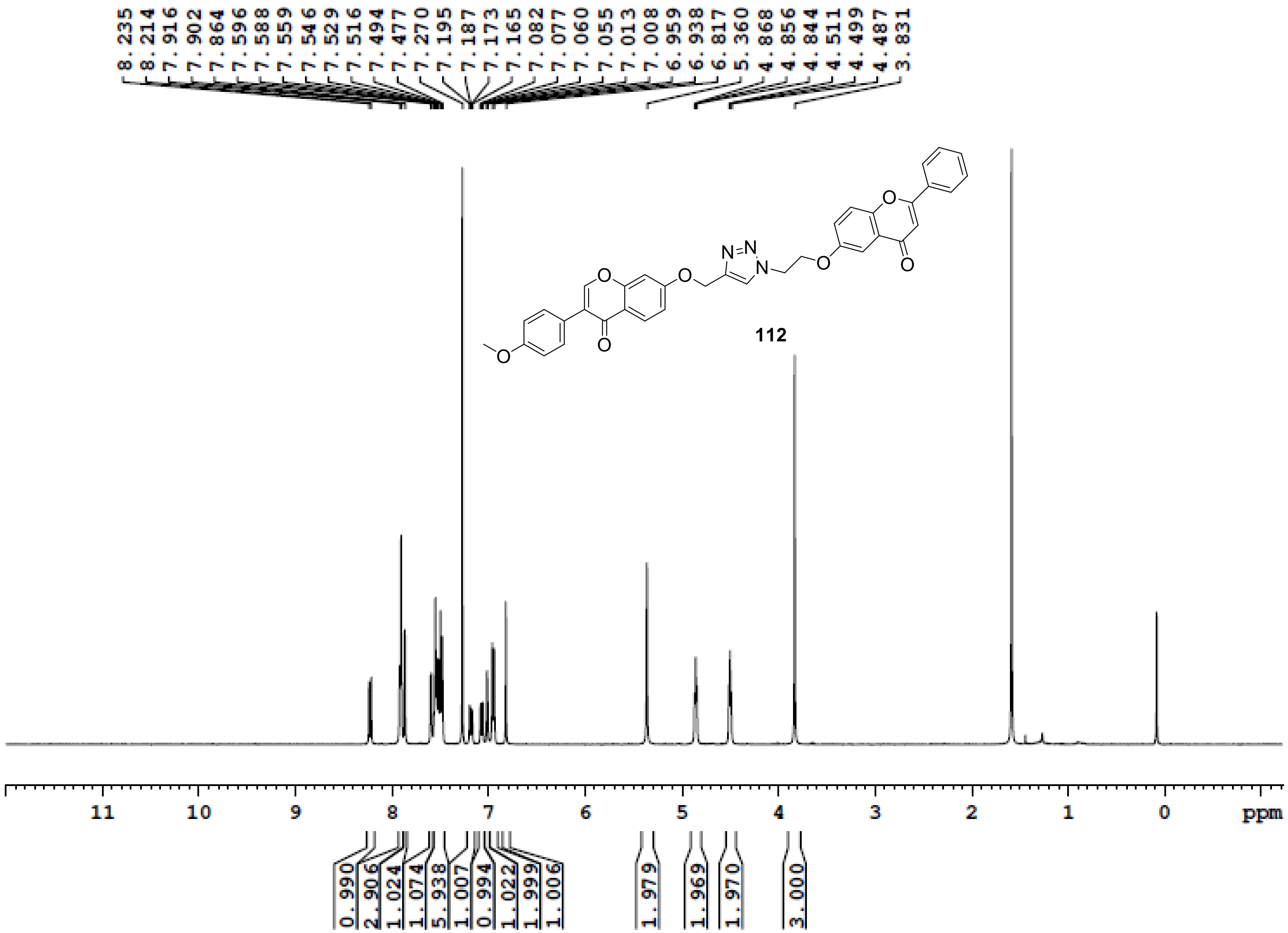
8.235
8.214
7.916
7.902
7.864
7.596
7.588
7.559
7.546
7.529
7.516
7.494
7.477
7.270
7.195
7.187
7.173
7.165
7.082
7.077
7.060
7.055
7.013
7.008
6.959
6.938
6.817
5.360
4.868
4.856
4.844
4.511
4.499
4.487
3.831
112
11 10 9 8 7 6 5 4 3 2 1 0 ppm
0.990
2.906
1.024
1.074
5.938
1.007
0.994
1.022
1.999
1.006
1.979
1.969
1.970
3.000

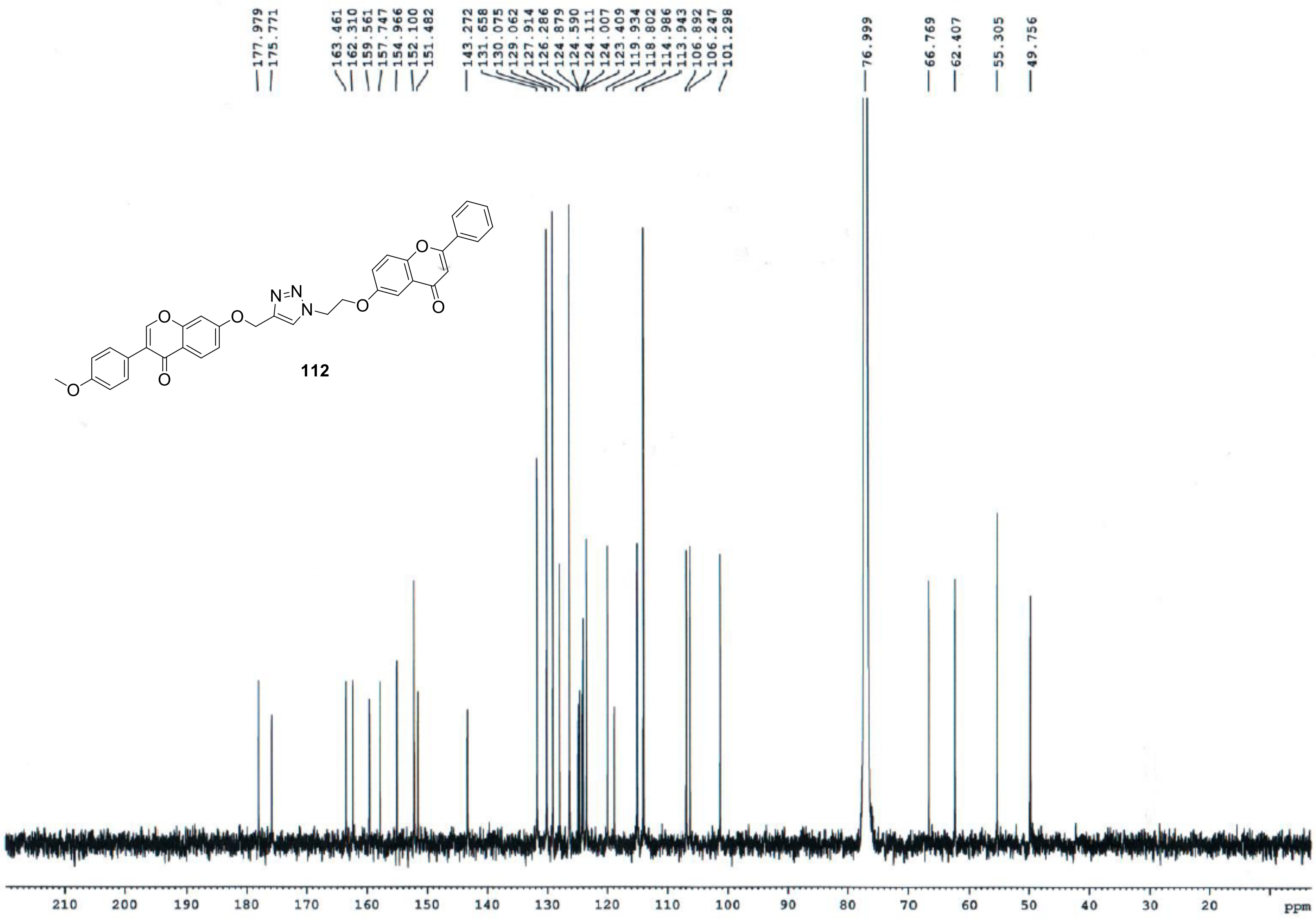
177.979
175.771
163.461
162.310
159.561
157.747
154.966
152.100
151.482
143.272
131.658
130.075
129.062
127.914
126.286
124.879
124.590
124.111
124.007
123.409
119.934
118.802
114.986
113.943
106.892
106.247
101.298
76.999
66.769
62.407
55.305
49.756
112
210 200 190 180 170 160 150 140 130 120 110 100 90 80 70 60 50 40 30 20 ppm

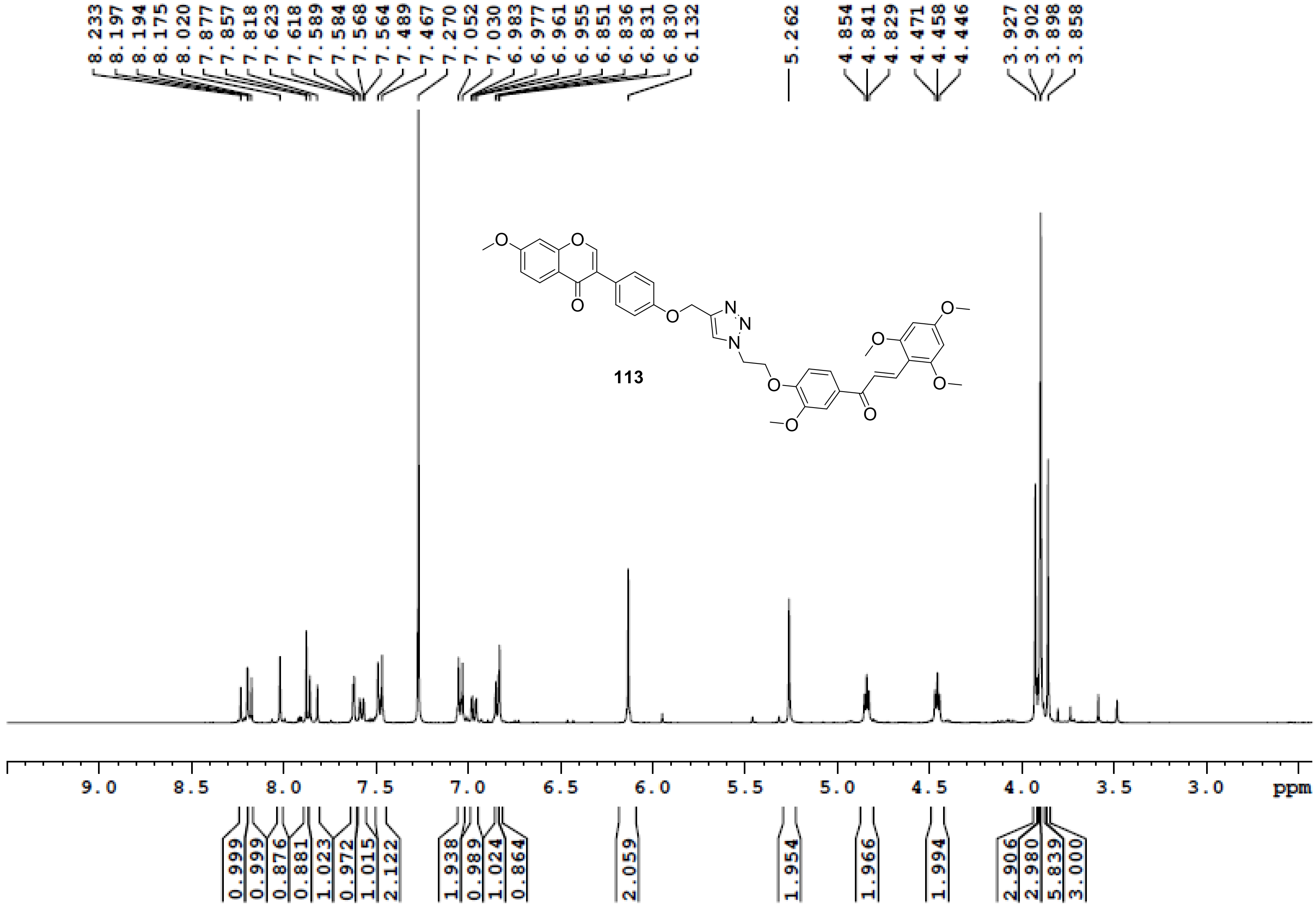
8.233 8.197 8.194 8.175 8.020 7.877 7.857 7.818 7.623 7.618 7.589 7.584 7.568 7.564 7.489 7.467 7.270 7.052 7.030 6.983 6.977 6.961 6.955 6.851 6.836 6.831 6.830 6.132
5.262
4.854 4.841 4.829 4.471 4.458 4.446
3.927 3.902 3.898 3.858
113
9.0 8.5 8.0 7.5 7.0 6.5 6.0 5.5 5.0 4.5 4.0 3.5 3.0 ppm
0.999 0.999 0.876 0.881 1.023 0.972 1.015 2.122 1.938 0.989 1.024 0.864 2.059 1.954 1.966 1.994 2.906 2.980 5.839 3.000

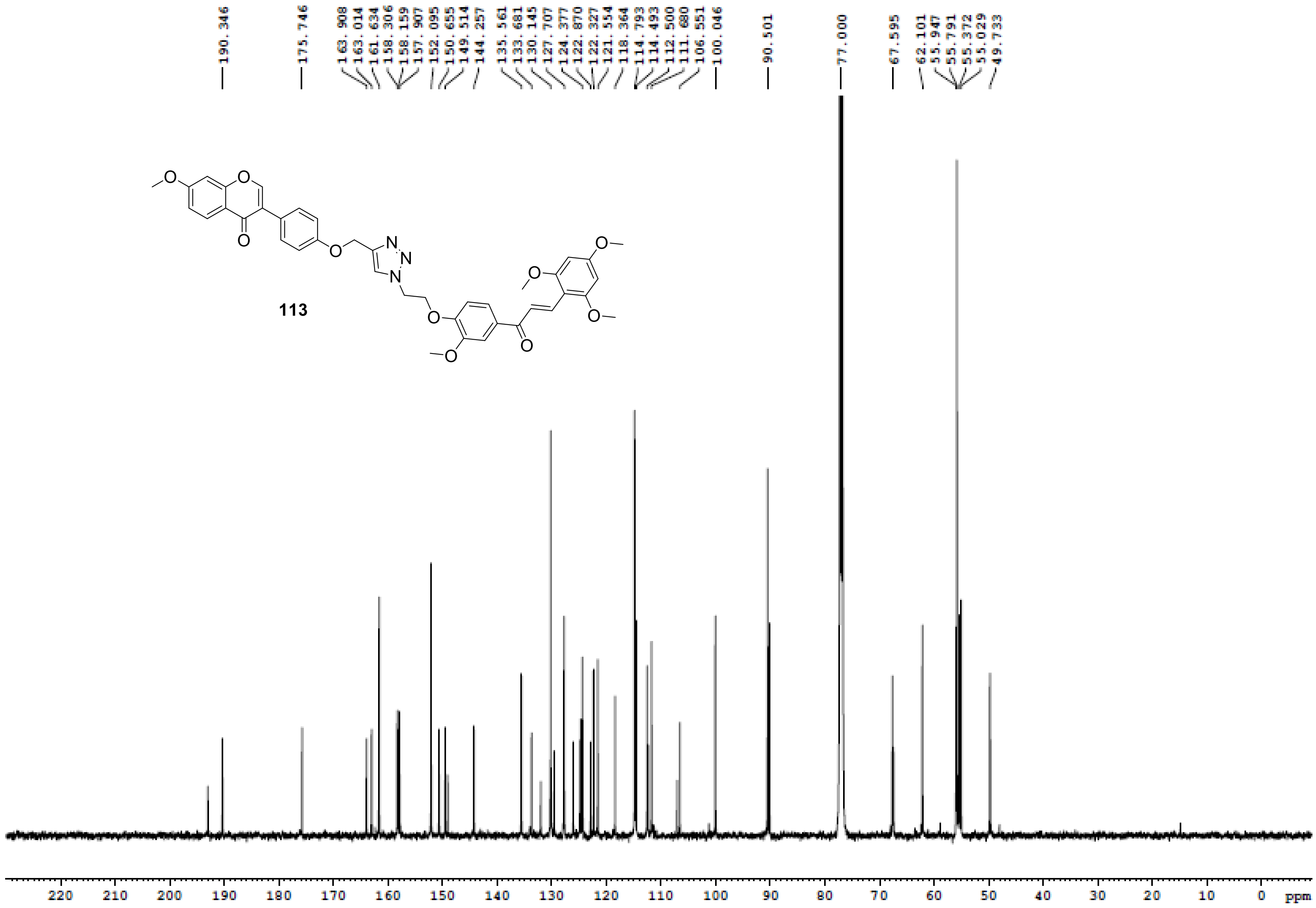
190.346
175.746
163.908
163.014
161.634
158.306
158.159
157.907
152.095
150.655
149.514
144.257
135.561
133.681
130.145
127.707
124.377
122.870
122.327
121.554
118.364
114.793
114.493
112.500
111.680
106.551
100.046
90.501
77.000
67.595
62.101
55.947
55.791
55.372
55.029
49.733
113
220 210 200 190 180 170 160 150 140 130 120 110 100 90 80 70 60 50 40 30 20 10 0 ppm

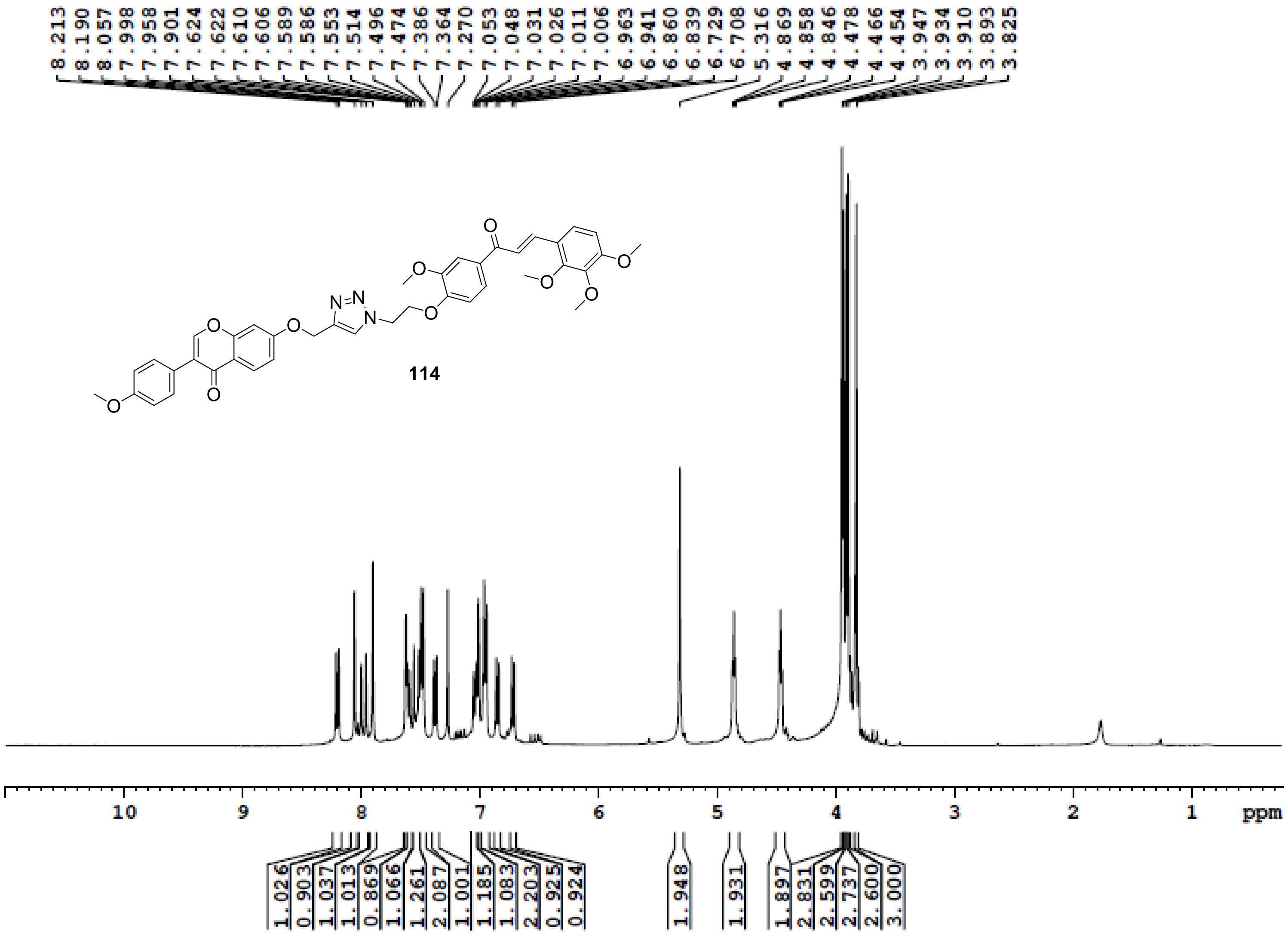
8.213 8.190 8.057 7.998 7.958 7.901 7.624 7.622 7.610 7.606 7.589 7.586 7.553 7.514 7.496 7.474 7.386 7.364 7.270 7.053 7.048 7.031 7.026 7.011 7.006 6.963 6.941 6.860 6.839 6.729 6.708 5.316 4.869 4.858 4.846 4.478 4.466 4.454 3.947 3.934 3.910 3.893 3.825
114
10 9 8 7 6 5 4 3 2 1 ppm
1.026 0.903 1.037 1.013 0.869 1.066 1.261 2.087 1.001 1.185 1.083 2.203 0.925 0.924 1.948 1.931 1.897 2.831 2.599 2.737 2.600 3.000

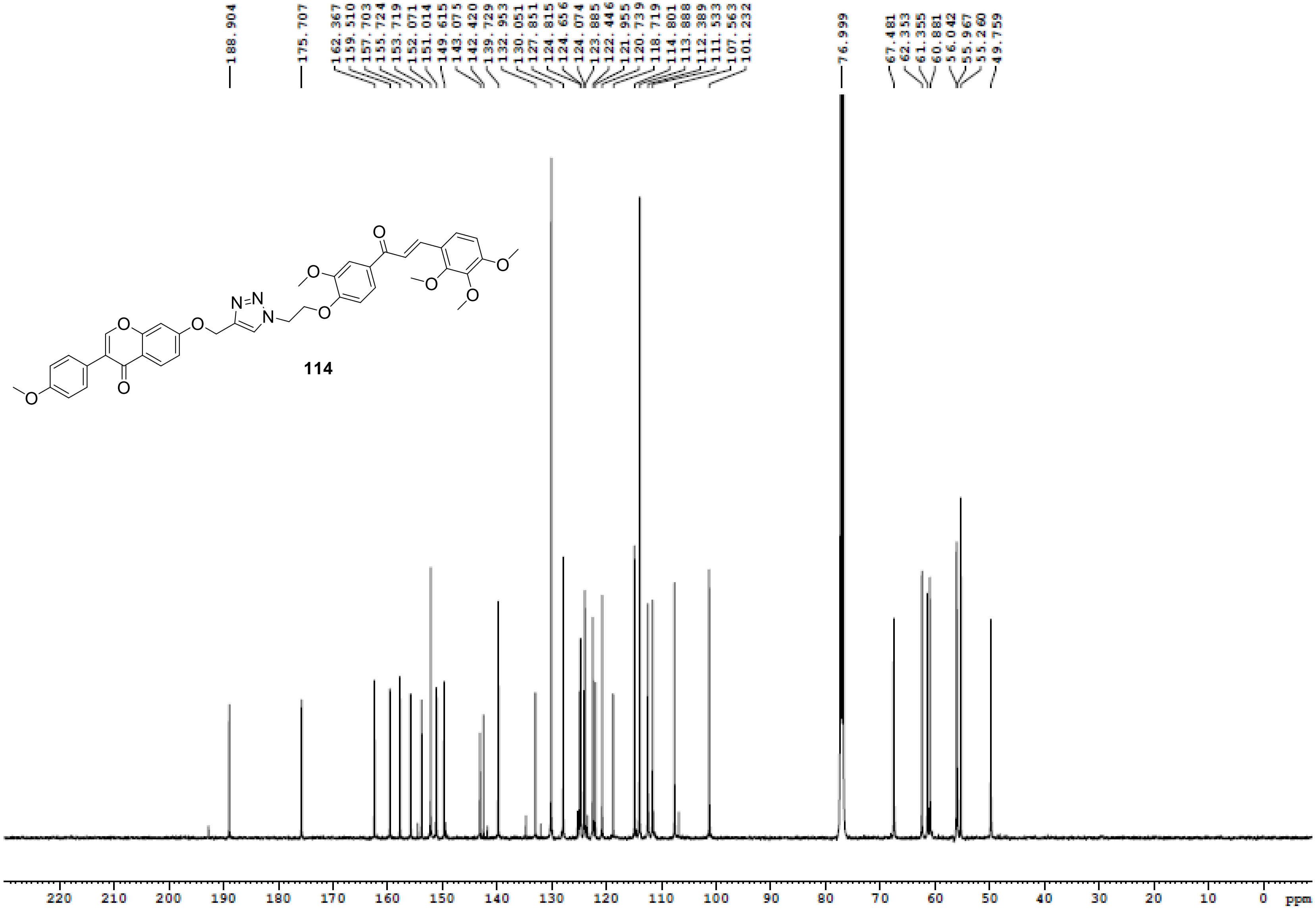
188.904
175.707
162.367
159.510
157.703
155.724
153.719
152.071
151.014
149.615
143.075
142.420
139.729
132.953
130.051
127.851
124.815
124.656
124.074
123.885
122.446
121.955
120.739
118.719
114.801
113.888
112.389
111.533
107.563
101.232
76.999
67.481
62.353
61.355
60.881
56.042
55.967
55.260
49.759
114
220 210 200 190 180 170 160 150 140 130 120 110 100 90 80 70 60 50 40 30 20 10 0 ppm

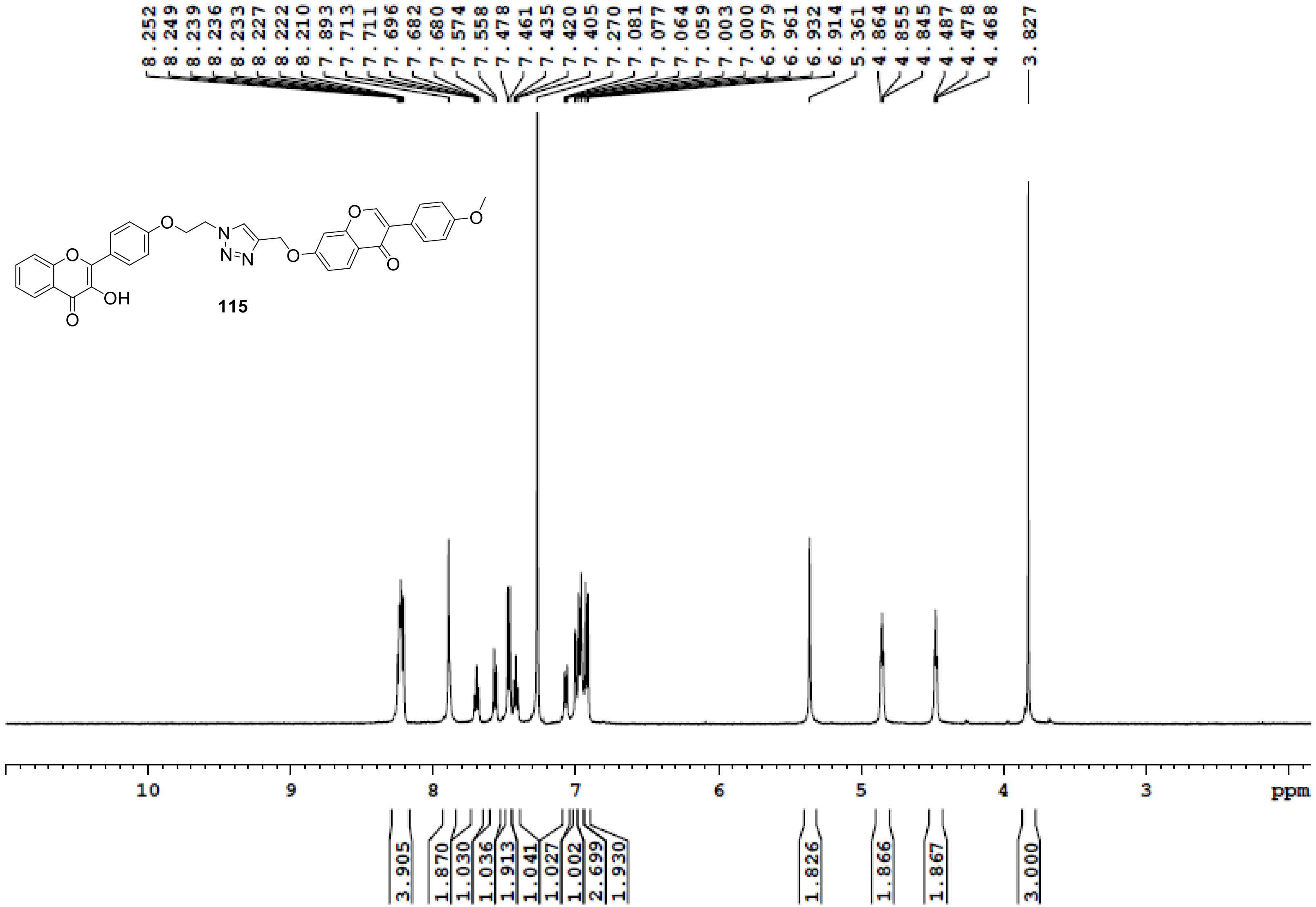
8.252
8.249
8.239
8.236
8.233
8.227
8.222
8.210
7.893
7.713
7.711
7.696
7.682
7.680
7.574
7.558
7.478
7.461
7.435
7.420
7.405
7.270
7.081
7.077
7.064
7.059
7.003
7.000
6.979
6.961
6.932
6.914
5.361
4.864
4.855
4.845
4.487
4.478
4.468
3.827
OH
O
N
115
10
9
8
7
6
5
4
3
ppm
3.905
1.870
1.030
1.036
1.913
1.041
1.027
1.002
2.699
1.930
1.826
1.866
1.867
3.000

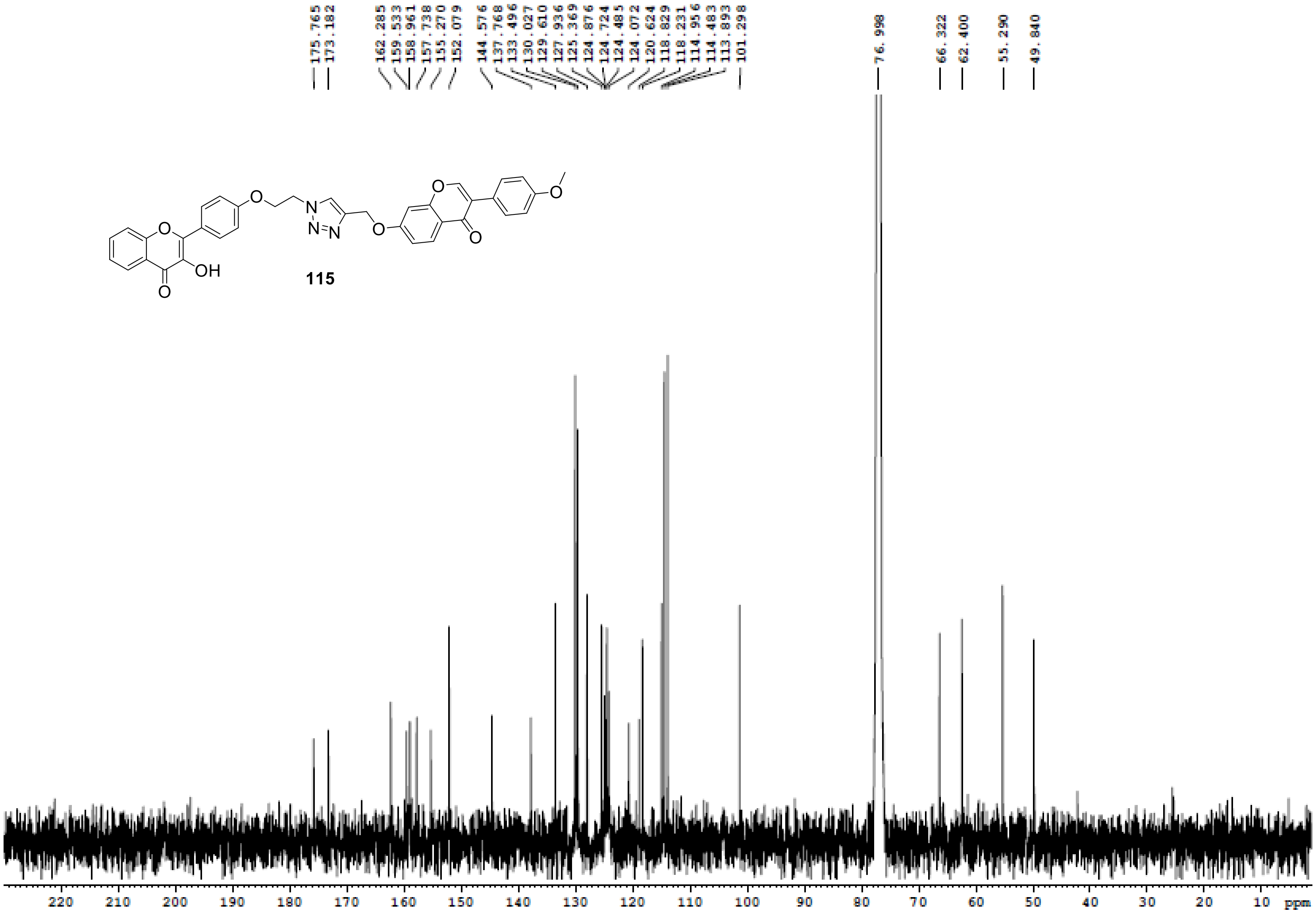
175.765
173.182
162.285
159.533
158.961
157.738
155.270
152.079
144.576
137.768
133.496
130.027
129.610
127.936
125.369
124.876
124.724
124.485
124.072
120.624
118.829
118.231
114.956
114.483
113.893
101.298
76.998
66.322
62.400
55.290
49.840
O
OH
N
115
220
210
200
190
180
170
160
150
140
130
120
110
100
90
80
70
60
50
40
30
20
10
ppm

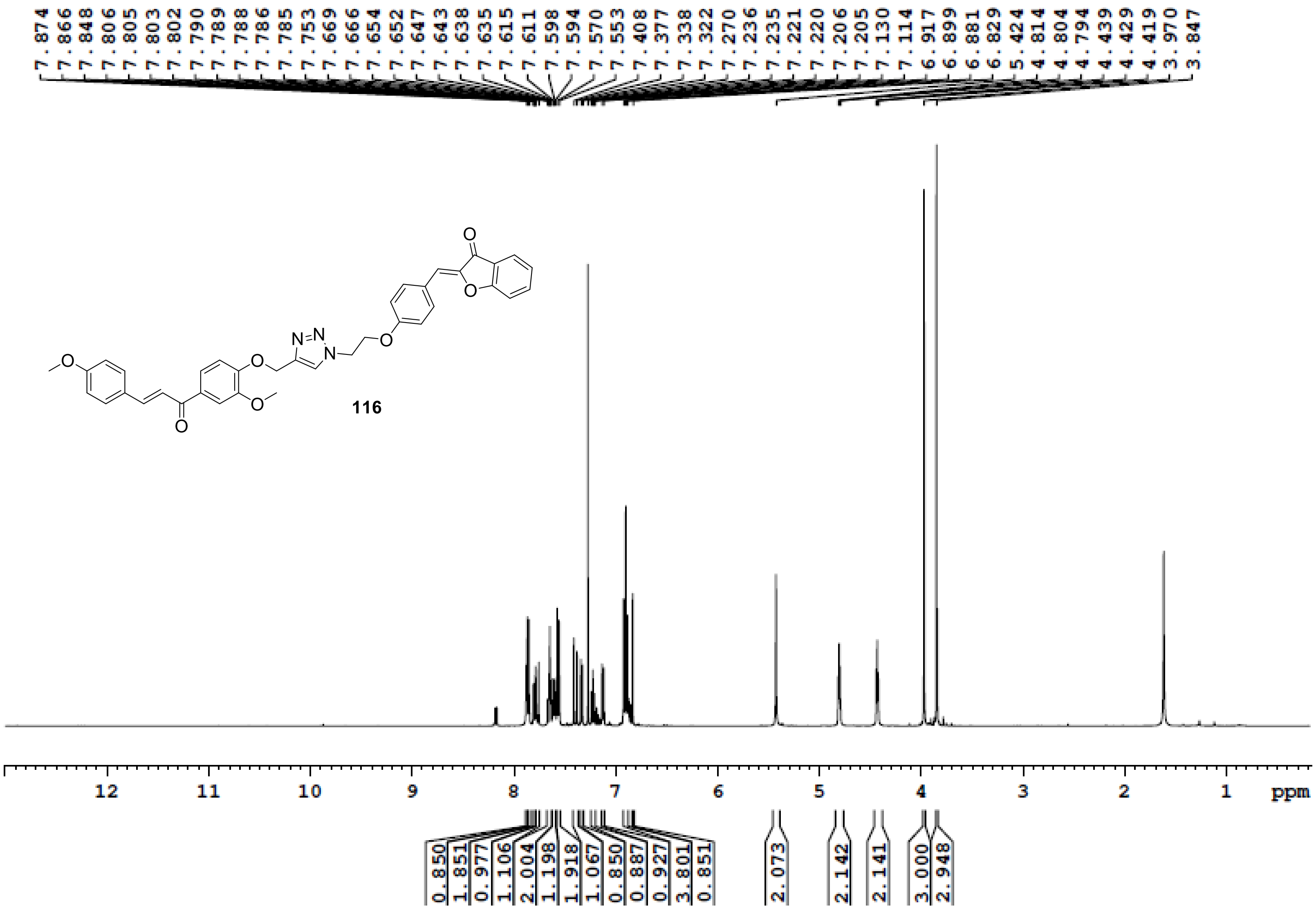
7.874 7.866 7.848 7.806 7.805 7.803 7.802 7.790 7.789 7.788 7.786 7.785 7.753 7.669 7.666 7.654 7.652 7.647 7.643 7.638 7.635 7.615 7.611 7.598 7.594 7.570 7.553 7.408 7.377 7.338 7.322 7.270 7.236 7.235 7.221 7.220 7.206 7.205 7.130 7.114 6.917 6.899 6.881 6.829 5.424 4.814 4.804 4.794 4.439 4.429 4.419 3.970 3.847
116
12 11 10 9 8 7 6 5 4 3 2 1 ppm
0.850 1.851 0.977 1.106 2.004 1.198 1.918 1.067 0.850 0.887 0.927 3.801 0.851 2.073 2.142 2.141 3.000 2.948

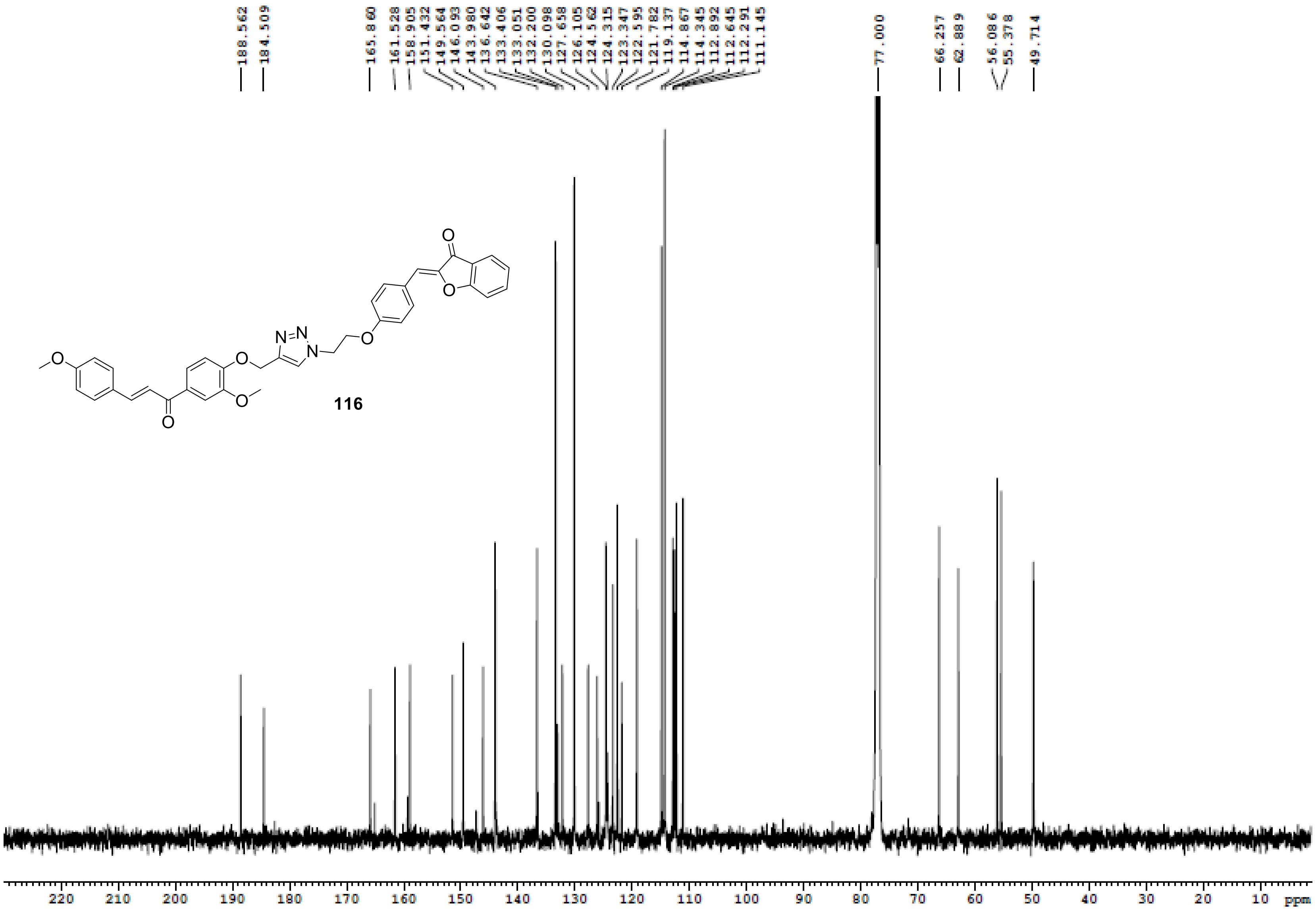
188.562
184.509
165.860
161.528
158.905
151.432
149.564
146.093
143.980
136.642
133.406
133.051
132.200
130.098
127.658
126.105
124.562
124.315
123.347
122.595
121.782
119.137
114.867
114.345
112.892
112.645
112.291
111.145
77.000
66.257
62.889
56.086
55.378
49.714
116
220
210
200
190
180
170
160
150
140
130
120
110
100
90
80
70
60
50
40
30
20
10
ppm

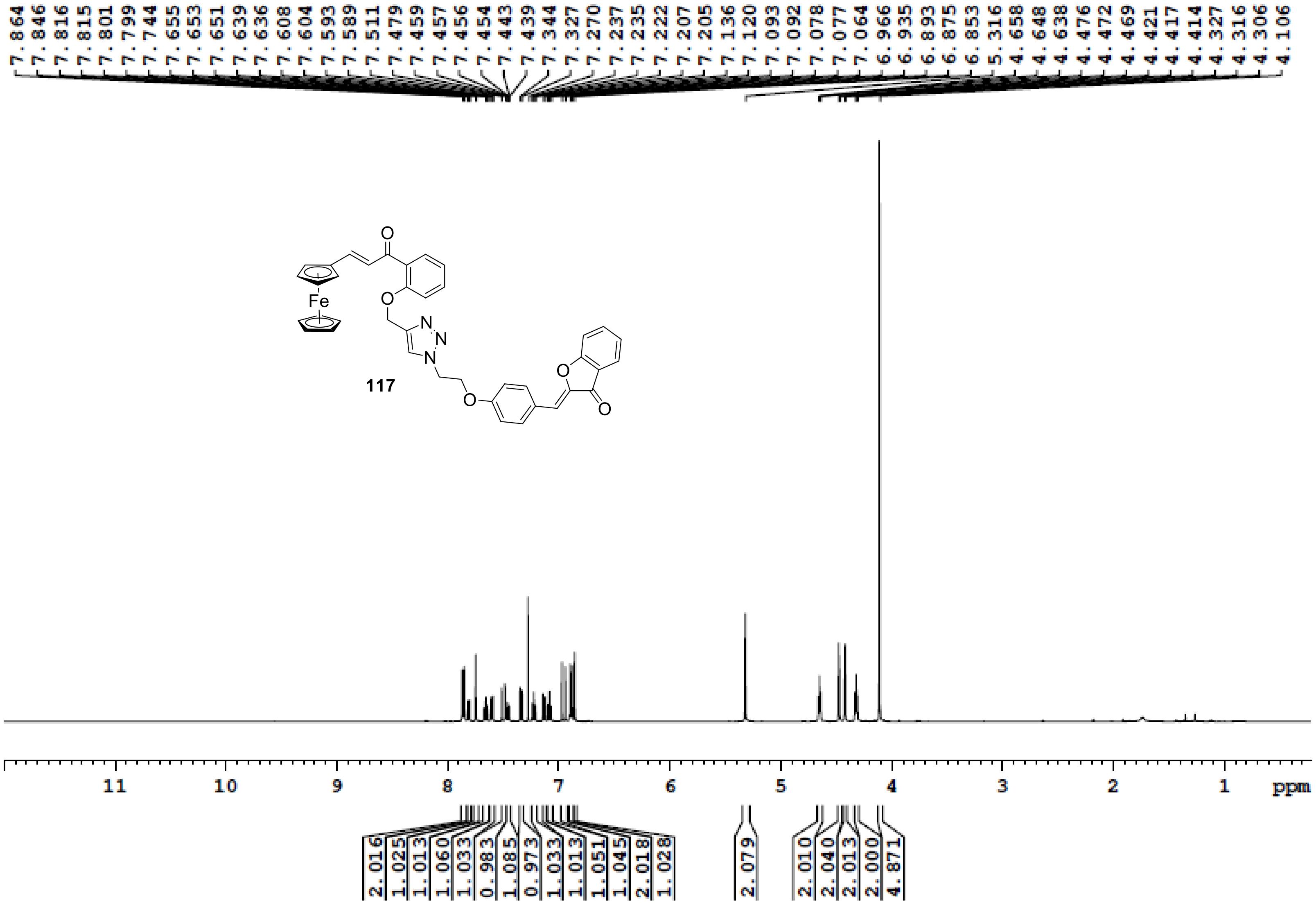
7.864 7.846 7.816 7.815 7.801 7.799 7.744 7.655 7.653 7.651 7.639 7.636 7.608 7.604 7.593 7.589 7.511 7.479 7.459 7.457 7.456 7.454 7.443 7.439 7.344 7.327 7.270 7.237 7.235 7.222 7.207 7.205 7.136 7.120 7.093 7.092 7.078 7.077 7.064 6.966 6.935 6.893 6.875 6.853 5.316 4.658 4.648 4.638 4.476 4.472 4.469 4.421 4.417 4.414 4.327 4.316 4.306 4.106
Fe
117
11 10 9 8 7 6 5 4 3 2 1 ppm
2.016 1.025 1.013 1.060 1.033 0.983 1.085 0.973 1.033 1.013 1.051 1.045 2.018 1.028 2.079 2.010 2.040 2.013 2.000 4.871

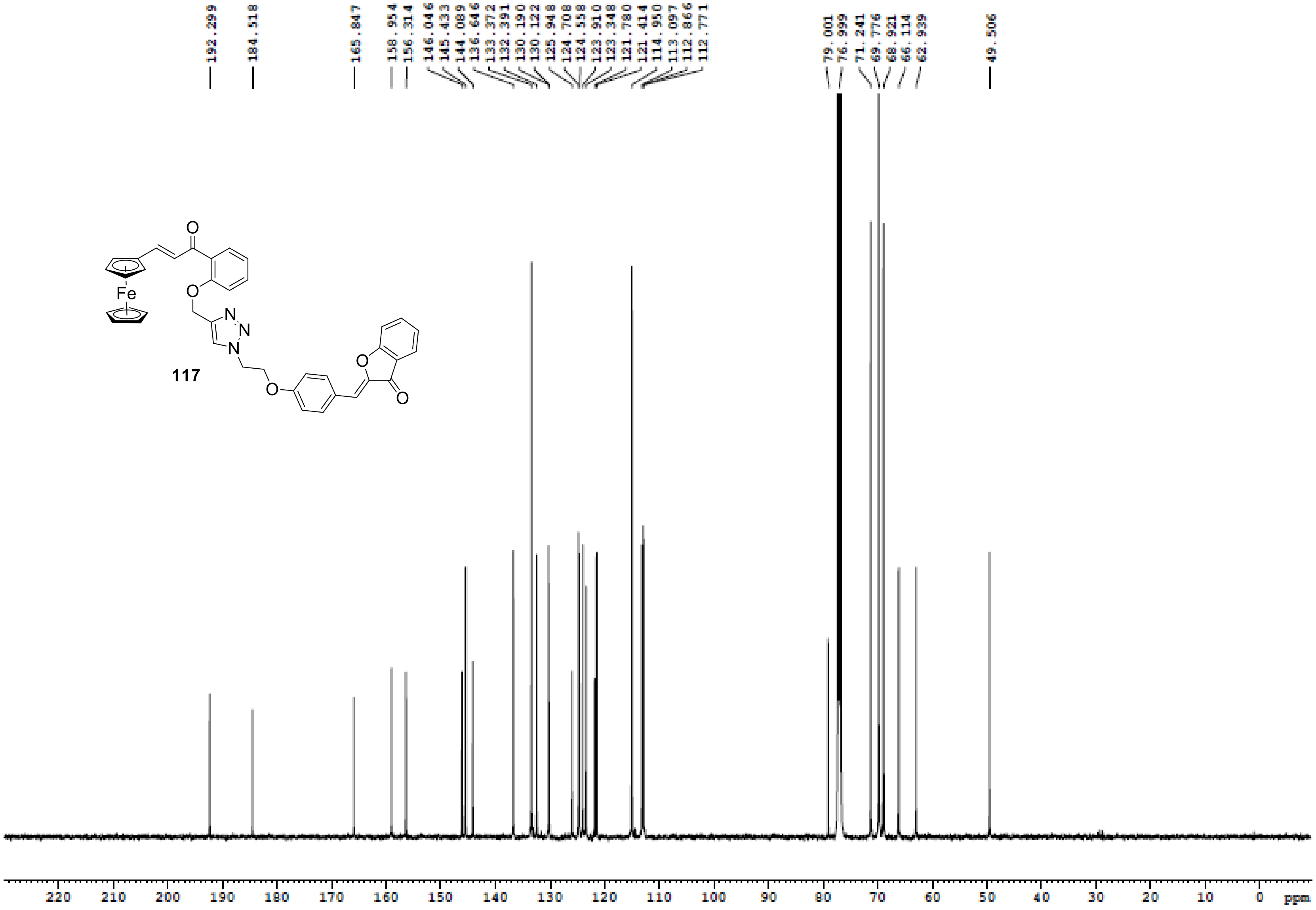
192.299
184.518
165.847
158.954
156.314
146.046
145.433
144.089
136.646
133.372
132.391
130.190
130.122
125.948
124.708
124.558
123.910
123.348
121.780
121.414
114.950
113.097
112.866
112.771
79.001
76.999
71.241
69.776
68.921
66.114
62.939
49.506
117
220 210 200 190 180 170 160 150 140 130 120 110 100 90 80 70 60 50 40 30 20 10 0 ppm

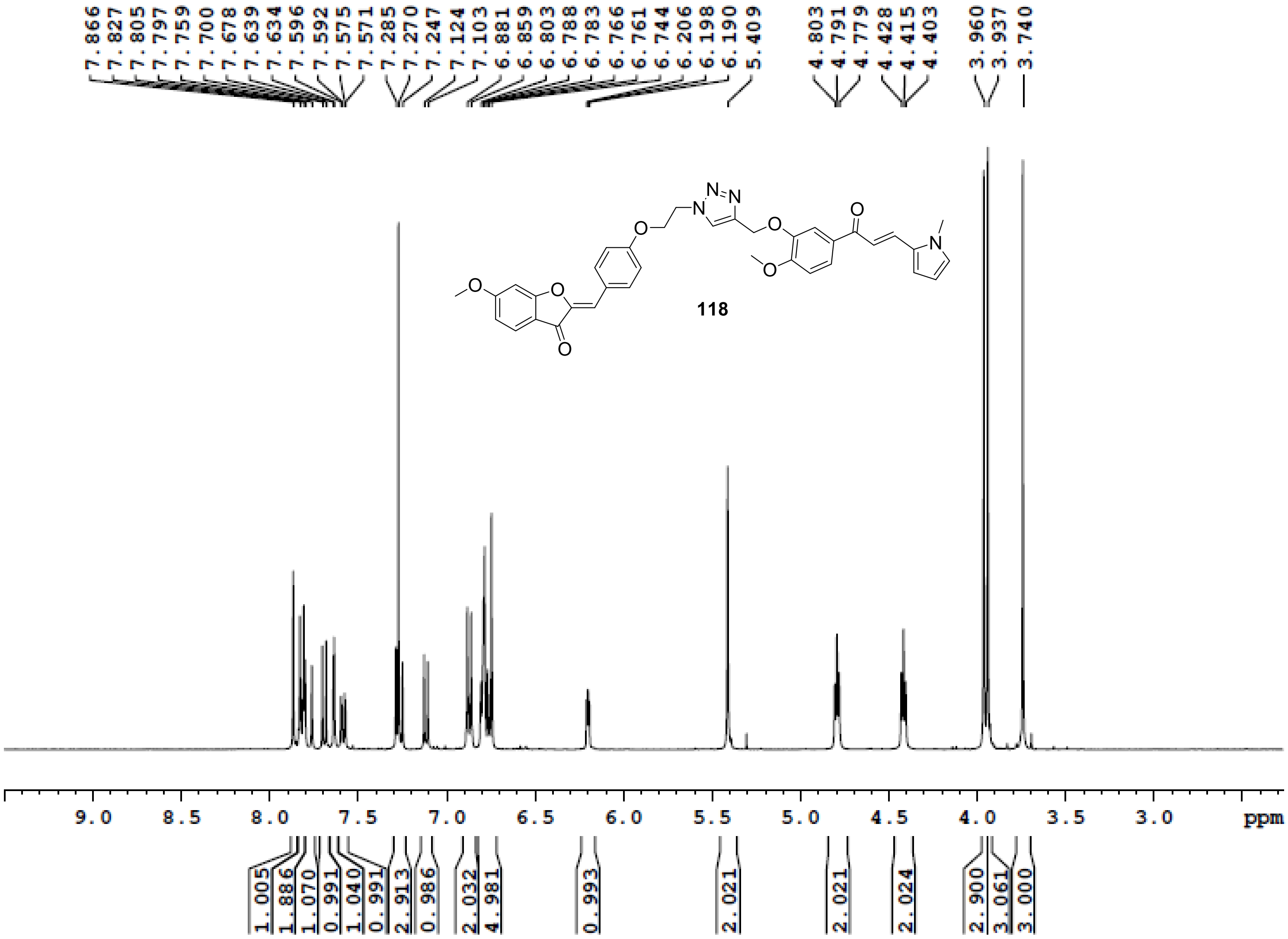
7.866 7.827 7.805 7.797 7.759 7.700 7.678 7.639 7.634 7.596 7.592 7.575 7.571 7.285 7.270 7.247 7.124 7.103 6.881 6.859 6.803 6.788 6.783 6.766 6.761 6.744 6.206 6.198 6.190 5.409
4.803 4.791 4.779 4.428 4.415 4.403
3.960 3.937 3.740
118
9.0 8.5 8.0 7.5 7.0 6.5 6.0 5.5 5.0 4.5 4.0 3.5 3.0 ppm
1.005 1.886 1.070 0.991 1.040 0.991 2.913 0.986 2.032 4.981 0.993 2.021 2.021 2.024 2.900 3.061 3.000

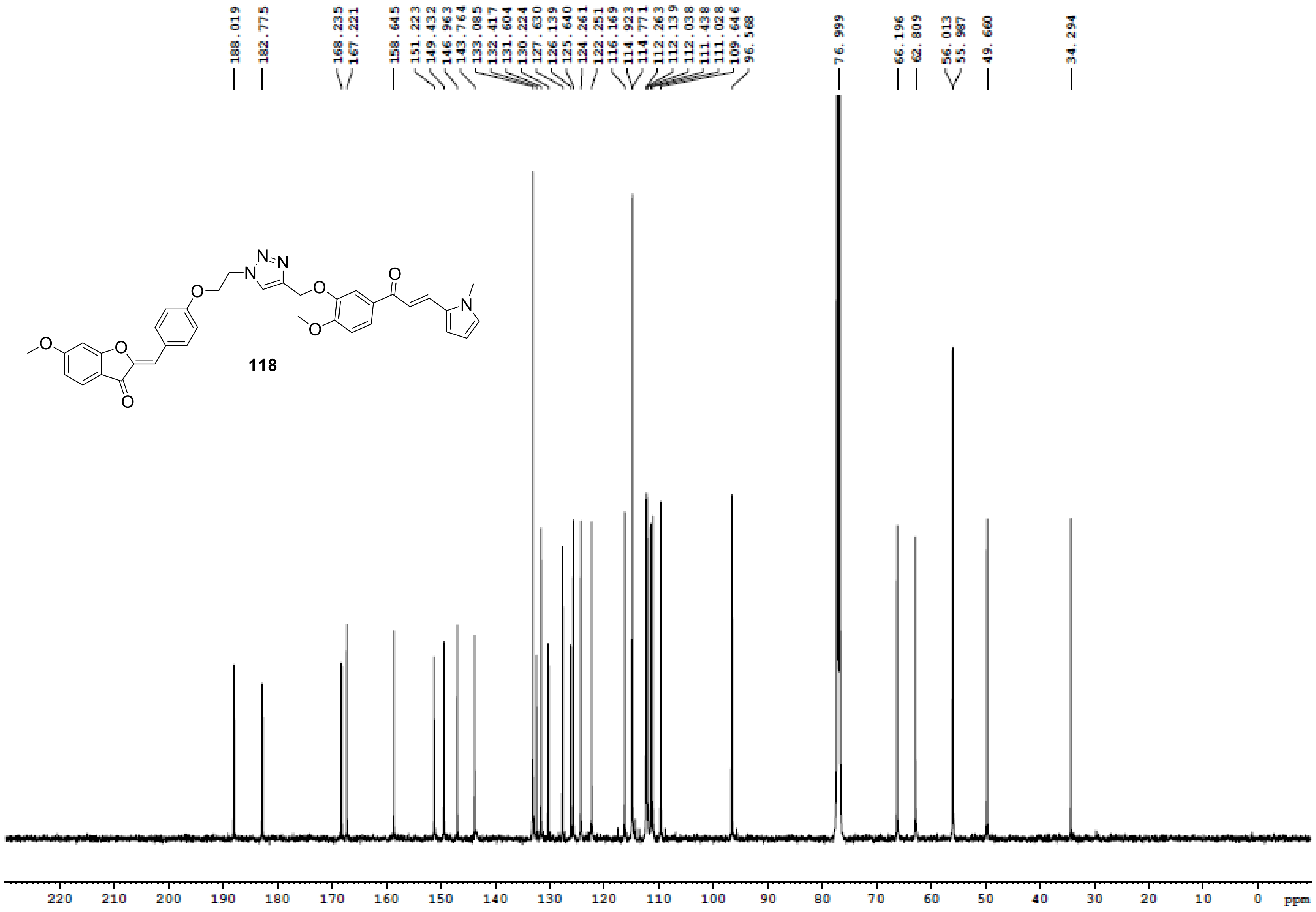
188.019
182.775
168.235
167.221
158.645
151.223
149.432
146.963
143.764
133.085
132.417
131.604
130.224
127.630
126.139
125.640
124.261
122.251
116.169
114.923
114.771
112.263
112.139
112.038
111.438
111.028
109.646
96.568
76.999
66.196
62.809
56.013
55.987
49.660
34.294
118
ppm

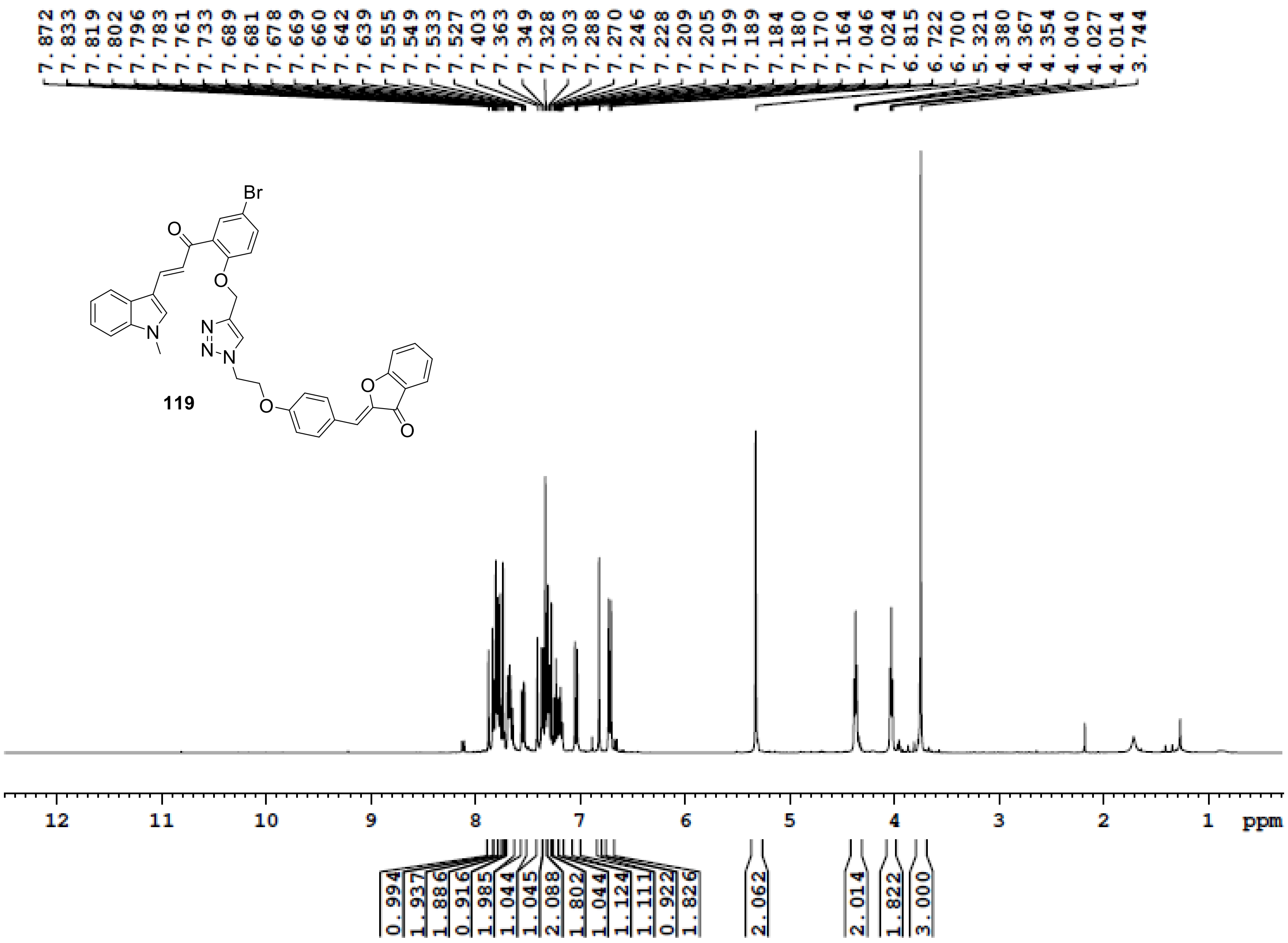
7.872
7.833
7.819
7.802
7.796
7.783
7.761
7.733
7.689
7.681
7.678
7.669
7.660
7.642
7.639
7.555
7.549
7.533
7.527
7.403
7.363
7.349
7.328
7.303
7.288
7.270
7.246
7.228
7.209
7.205
7.199
7.189
7.184
7.180
7.170
7.164
7.046
7.024
6.815
6.722
6.700
5.321
4.380
4.367
4.354
4.040
4.027
4.014
3.744
Br
O
N
N
N
N
O
O
O
O
119
12
11
10
9
8
7
6
5
4
3
2
1
ppm
0.994
1.937
1.886
0.916
1.985
1.044
1.045
2.088
1.802
1.044
1.124
1.111
0.922
1.826
2.062
2.014
1.822
3.000

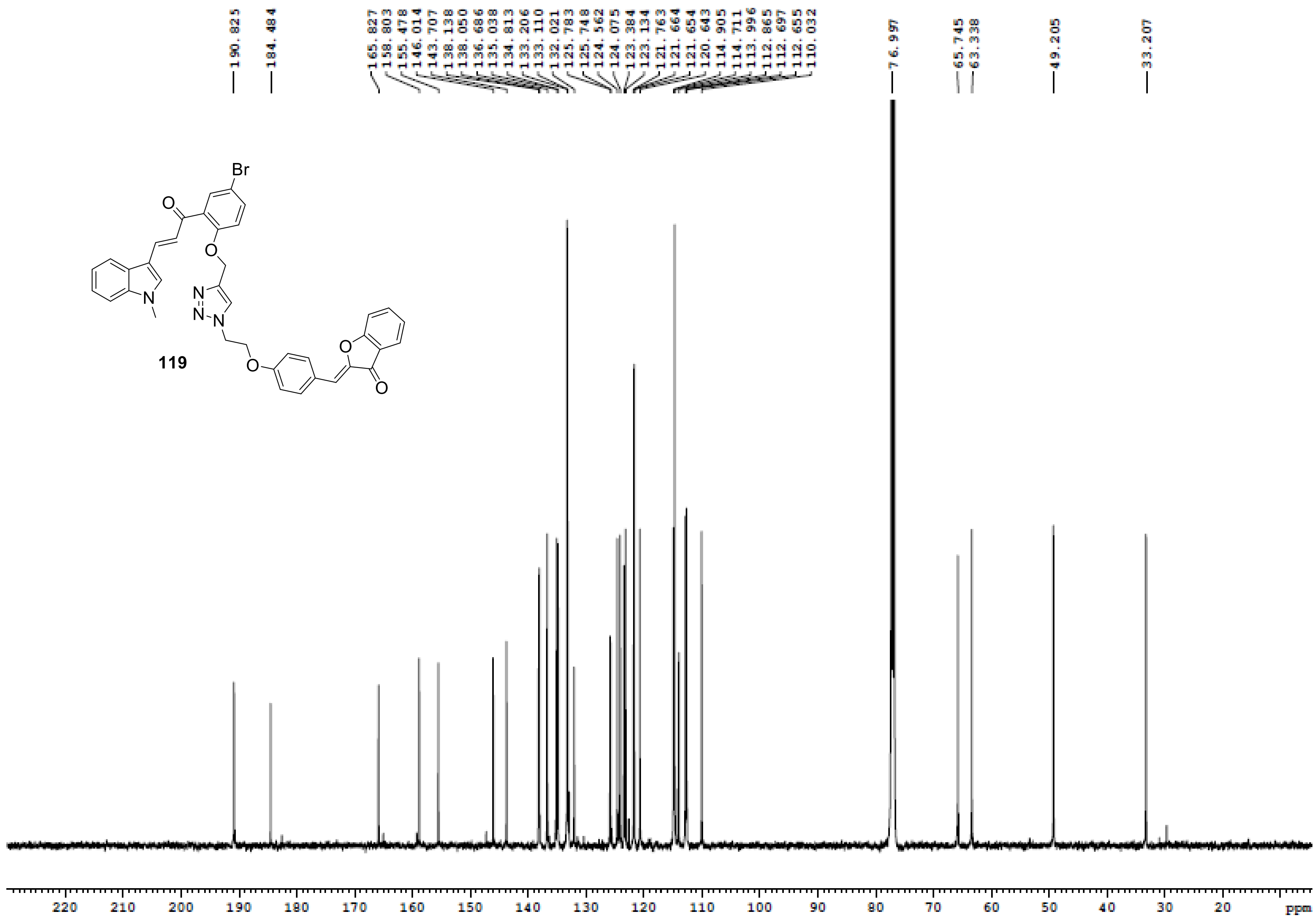
190.825
184.484
165.827
158.803
155.478
146.014
143.707
138.138
138.050
136.686
135.038
134.813
133.206
133.110
132.021
125.783
125.748
124.562
124.075
123.384
123.134
121.763
121.664
121.654
120.643
114.905
114.711
113.996
112.865
112.697
112.655
110.032
76.997
65.745
63.338
49.205
33.207
119
220 210 200 190 180 170 160 150 140 130 120 110 100 90 80 70 60 50 40 30 20 ppm

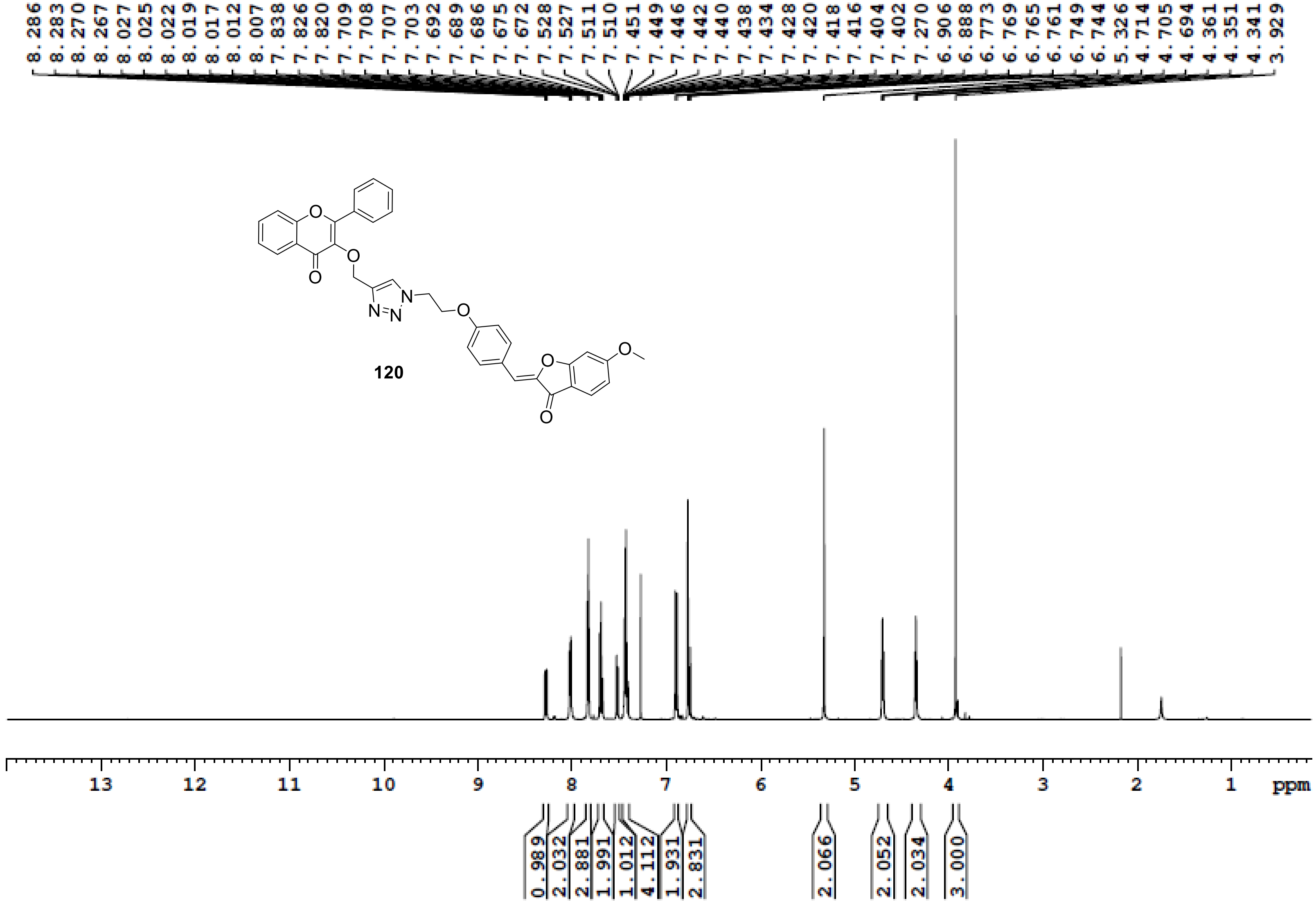
8.286 8.283 8.270 8.267 8.027 8.025 8.022 8.019 8.017 8.012 8.007 7.838 7.826 7.820 7.709 7.708 7.707 7.703 7.692 7.689 7.686 7.675 7.672 7.528 7.527 7.511 7.510 7.451 7.449 7.446 7.442 7.440 7.438 7.434 7.428 7.420 7.418 7.416 7.404 7.402 7.270 6.906 6.888 6.773 6.769 6.765 6.761 6.749 6.744 5.326 4.714 4.705 4.694 4.361 4.351 4.341 3.929
120
13 12 11 10 9 8 7 6 5 4 3 2 1 ppm
0.989 2.032 2.881 1.991 1.012 4.112 1.931 2.831 2.066 2.052 2.034 3.000

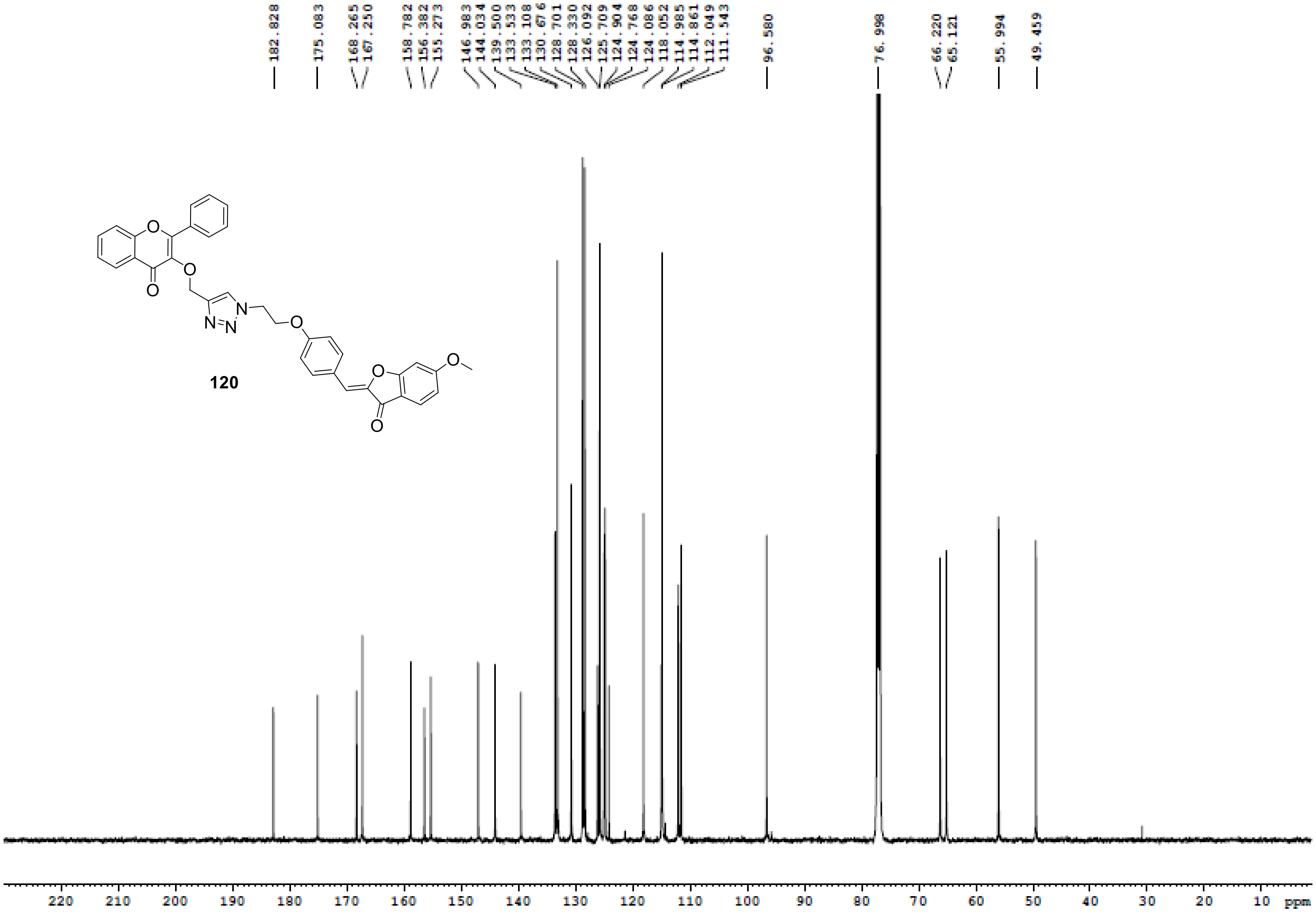
182.828
175.083
168.265
167.250
158.782
156.382
155.273
146.983
144.034
139.500
133.533
133.108
130.676
128.701
128.330
126.092
125.709
124.904
124.768
124.086
118.052
114.985
114.861
112.049
111.543
96.580
76.998
66.220
65.121
55.994
49.459
120
220 210 200 190 180 170 160 150 140 130 120 110 100 90 80 70 60 50 40 30 20 10 ppm
O
O
O
N
N
N
O
O
O
O

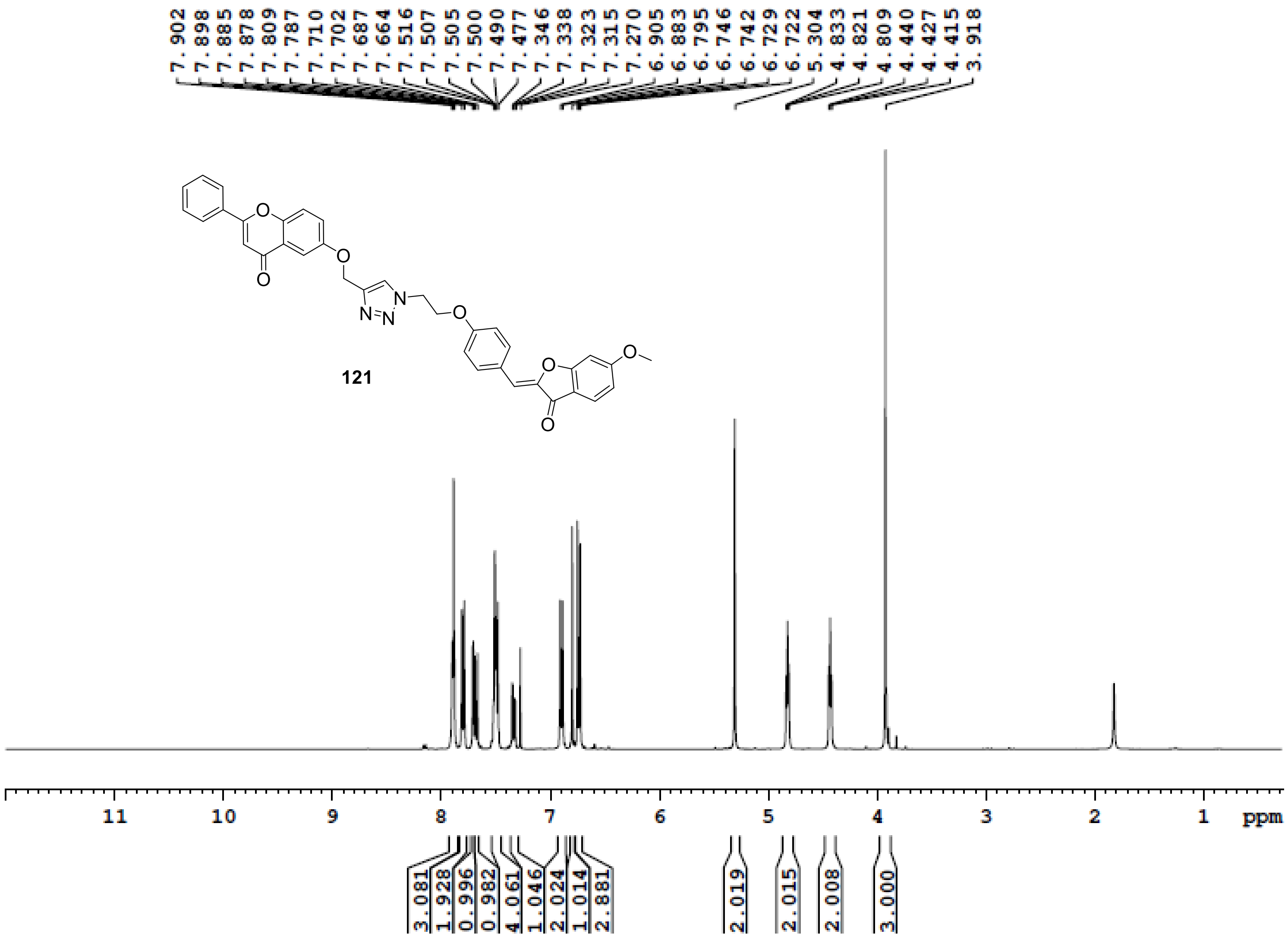
7.902
7.898
7.885
7.878
7.809
7.787
7.710
7.702
7.687
7.664
7.516
7.507
7.505
7.500
7.490
7.477
7.346
7.338
7.323
7.315
7.270
6.905
6.883
6.795
6.746
6.742
6.729
6.722
5.304
4.833
4.821
4.809
4.440
4.427
4.415
3.918
121
11
10
9
8
7
6
5
4
3
2
1
ppm
3.081
1.928
0.996
0.982
4.061
1.046
2.024
1.014
2.881
2.019
2.015
2.008
3.000

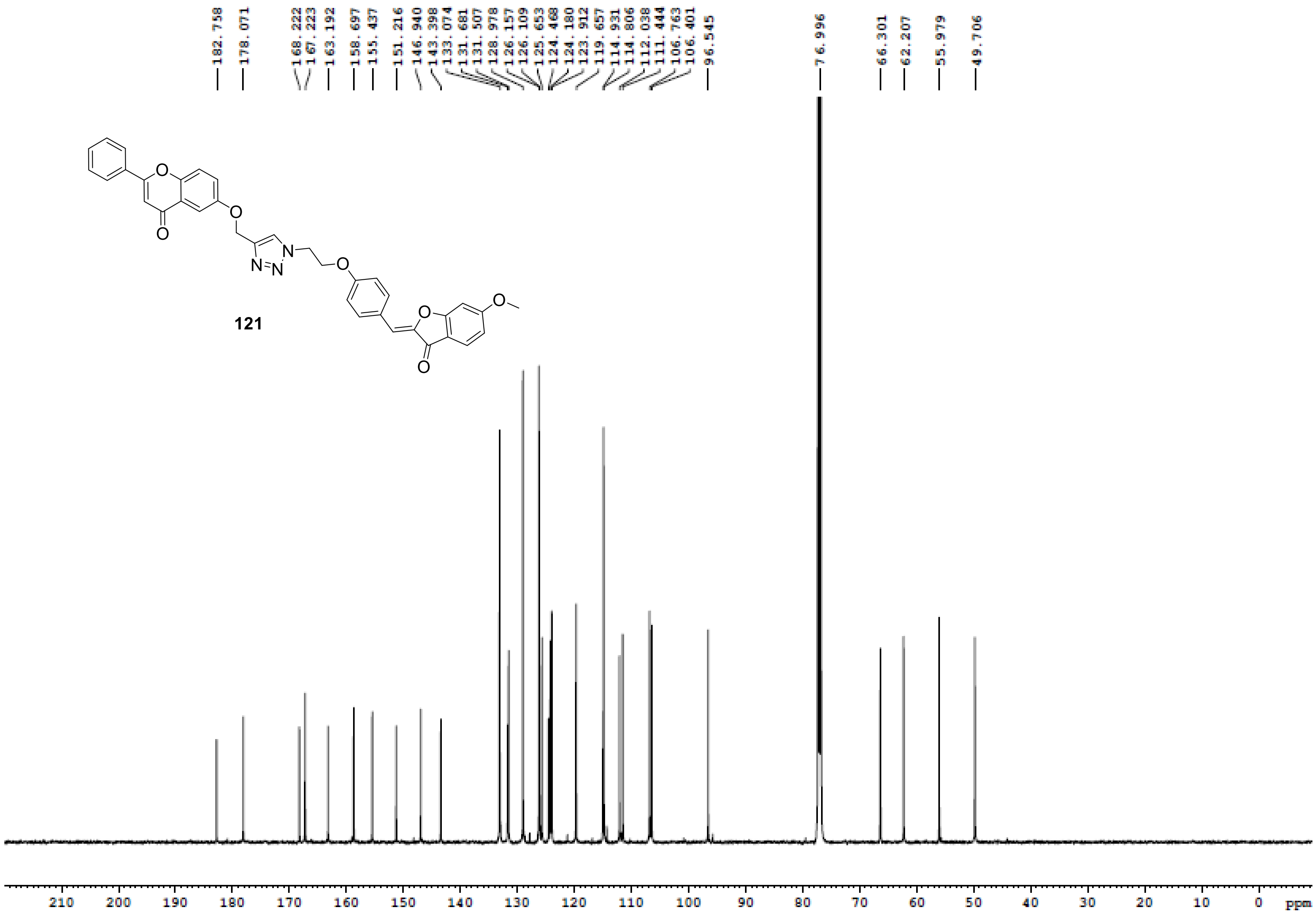
182.758
178.071
168.222
167.223
163.192
158.697
155.437
151.216
146.940
143.398
133.074
131.681
131.507
128.978
126.157
126.109
125.653
124.468
124.180
123.912
119.657
114.931
114.806
112.038
111.444
106.763
106.401
96.545
76.996
66.301
62.207
55.979
49.706
121
210 200 190 180 170 160 150 140 130 120 110 100 90 80 70 60 50 40 30 20 10 0 ppm

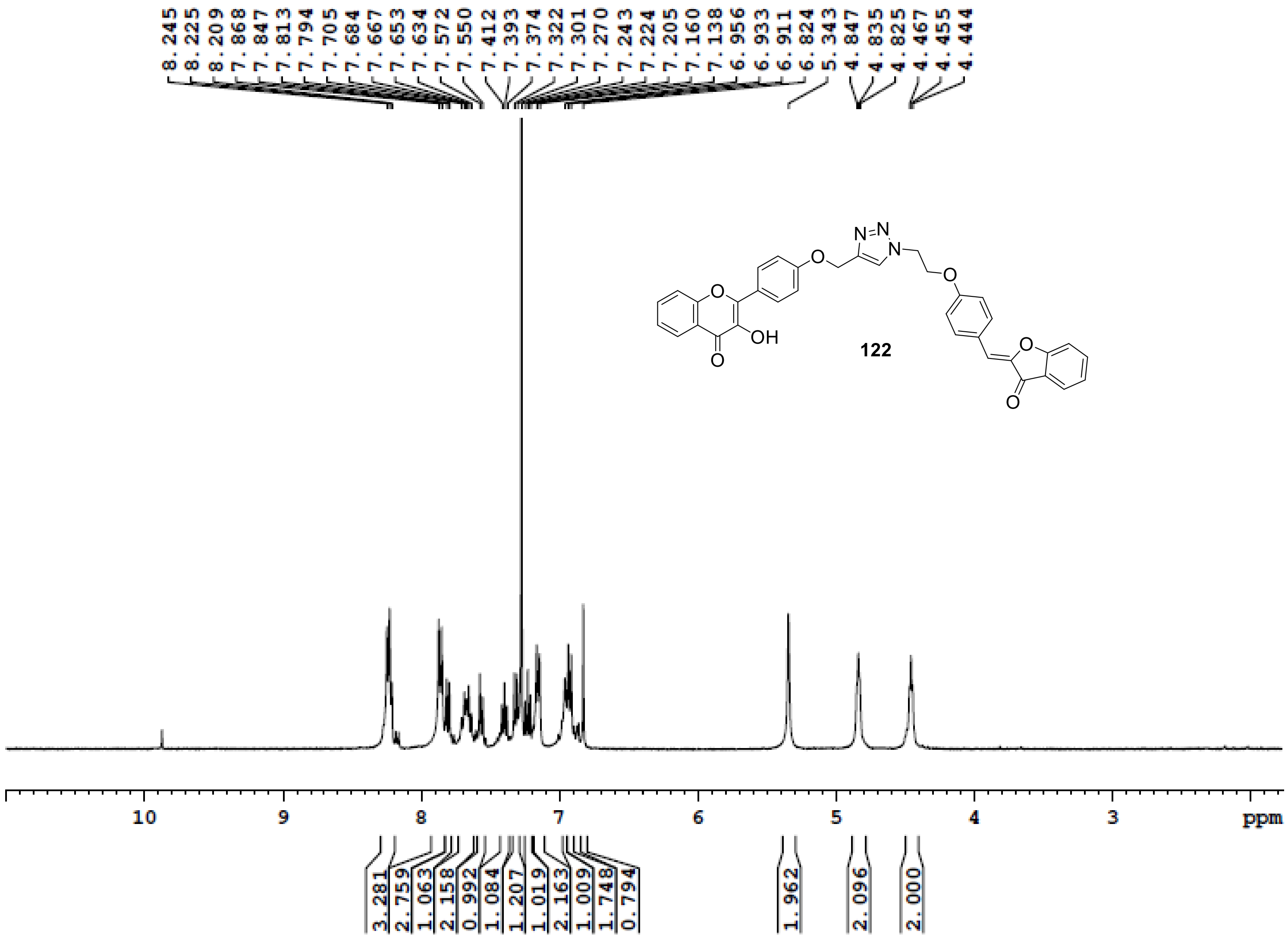
8.245
8.225
8.209
7.868
7.847
7.813
7.794
7.705
7.684
7.667
7.653
7.634
7.572
7.550
7.412
7.393
7.374
7.322
7.301
7.270
7.243
7.224
7.205
7.160
7.138
6.956
6.933
6.911
6.824
5.343
4.847
4.835
4.825
4.467
4.455
4.444
N
N
N
O
O
O
OH
O
122
O
O
10
9
8
7
6
5
4
3
ppm
3.281
2.759
1.063
2.158
0.992
1.084
1.207
1.019
2.163
1.009
1.748
0.794
1.962
2.096
2.000

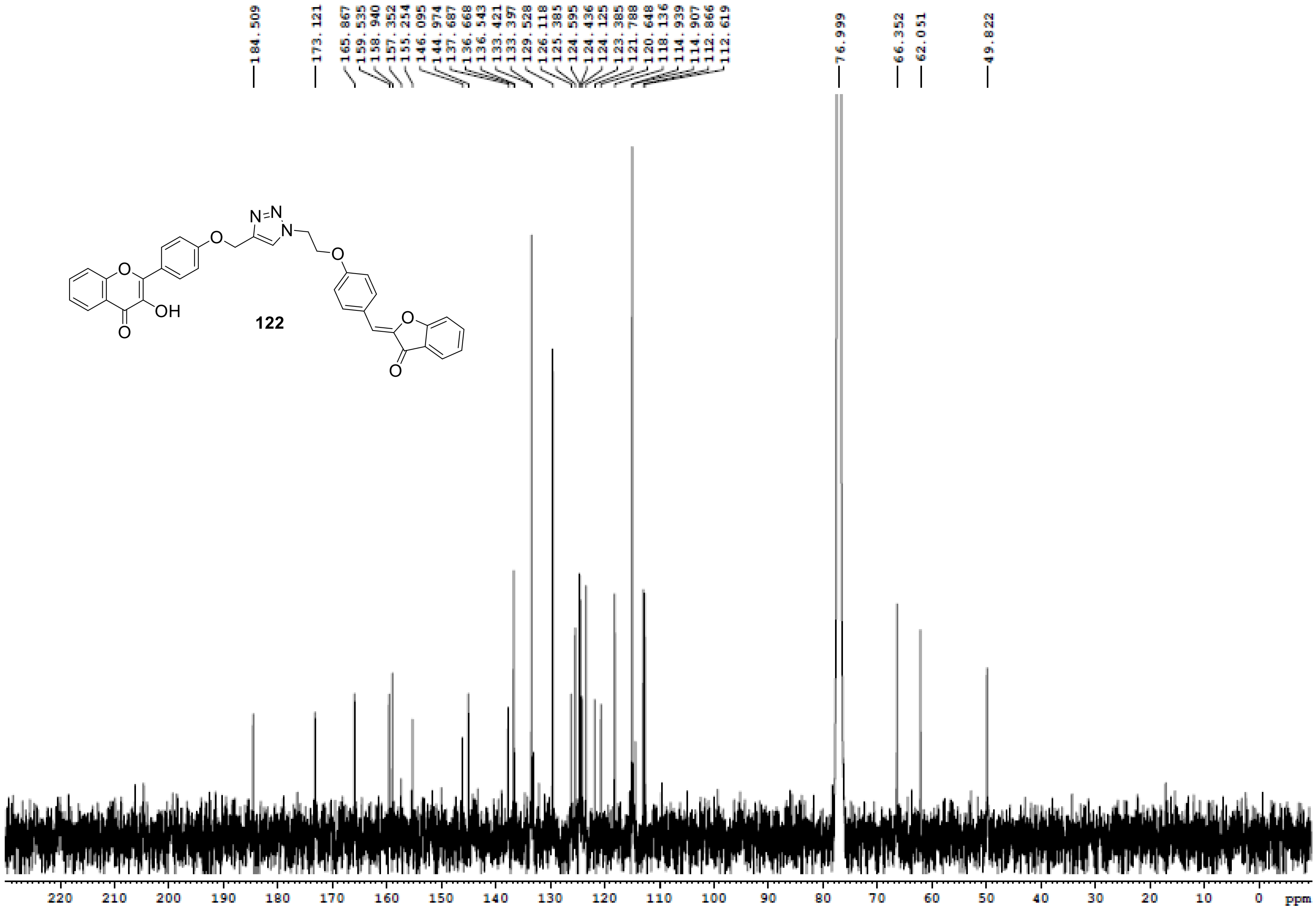
184.509
173.121
165.867
159.535
158.940
157.352
155.254
146.095
144.974
137.687
136.668
136.543
133.421
133.397
129.528
126.118
125.385
124.595
124.436
124.125
123.385
121.788
120.648
118.136
114.939
114.907
112.866
112.619
76.999
66.352
62.051
49.822
N=N
N
O
O
O
OH
O
O
O
122
220 210 200 190 180 170 160 150 140 130 120 110 100 90 80 70 60 50 40 30 20 10 0 ppm

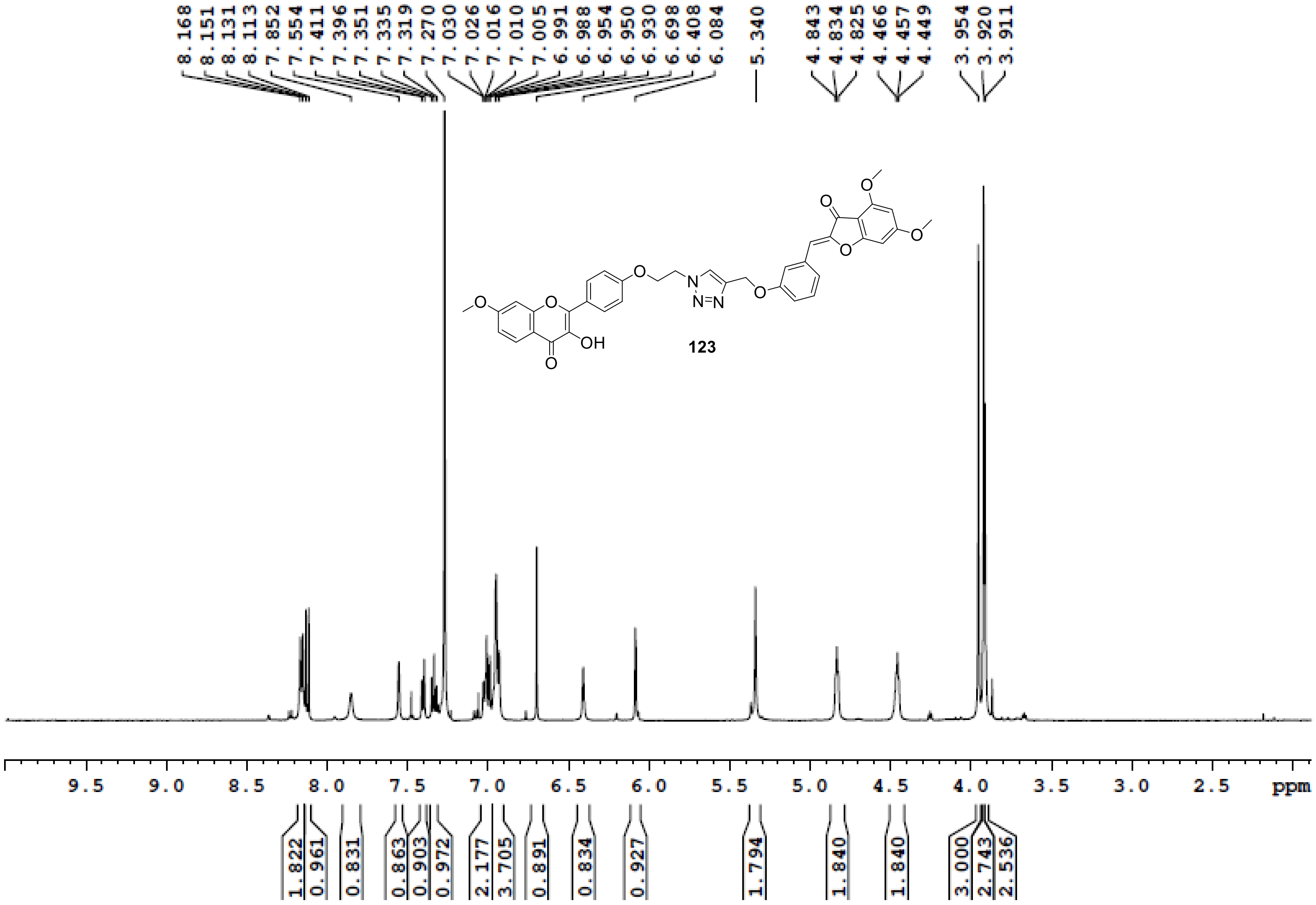
8.168 8.151 8.131 8.113 7.852 7.554 7.411 7.396 7.351 7.335 7.319 7.270 7.030 7.026 7.016 7.010 7.005 6.991 6.988 6.954 6.950 6.930 6.698 6.408 6.084
5.340
4.843 4.834 4.825 4.466 4.457 4.449
3.954 3.920 3.911
OH
123
9.5 9.0 8.5 8.0 7.5 7.0 6.5 6.0 5.5 5.0 4.5 4.0 3.5 3.0 2.5 ppm
1.822 0.961 0.831 0.863 0.903 0.972 2.177 3.705 0.891 0.834 0.927 1.794 1.840 1.840 3.000 2.743 2.536

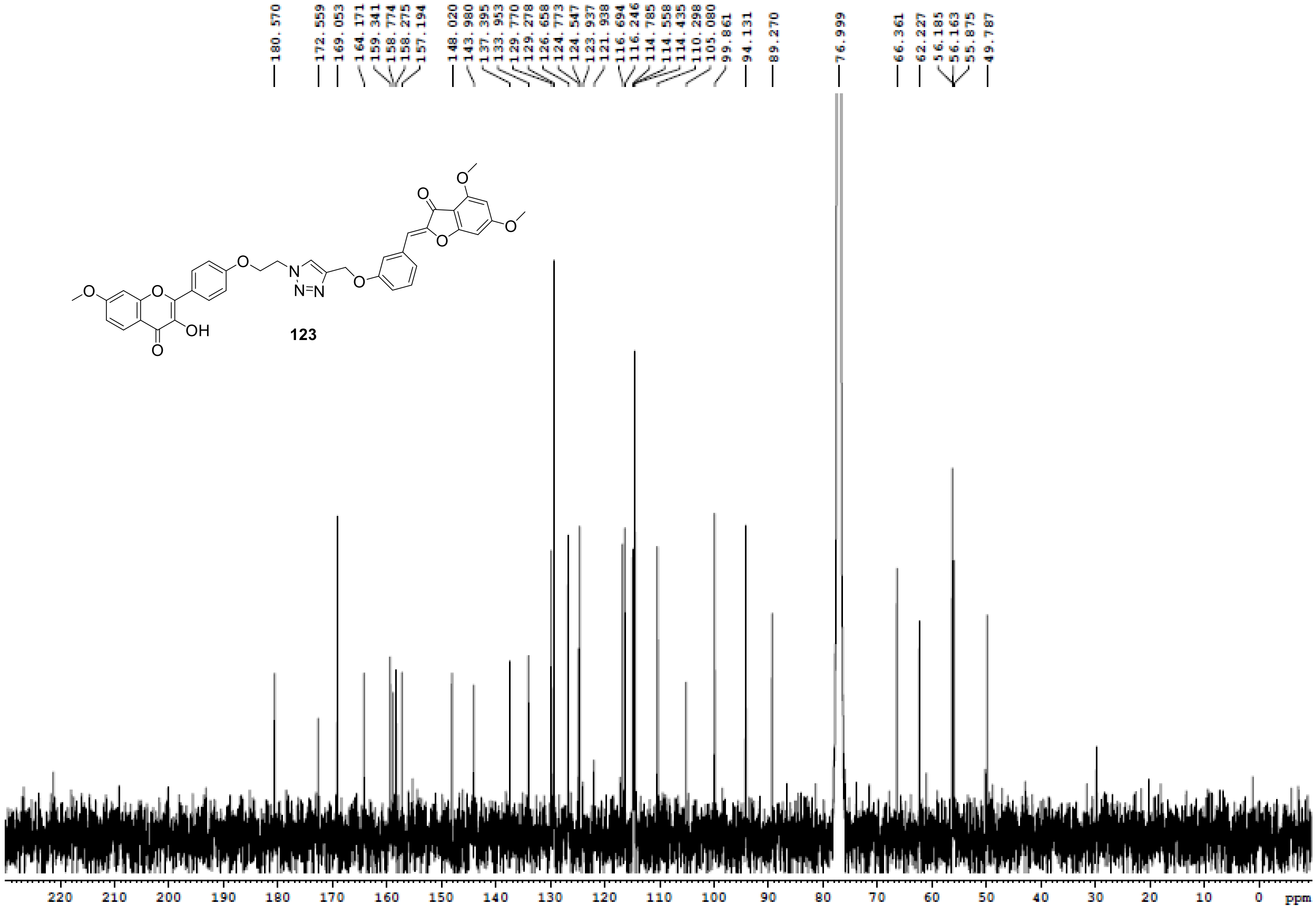
180.570
172.559
169.053
164.171
159.341
158.774
158.275
157.194
148.020
143.980
137.395
133.953
129.770
129.278
126.658
124.773
124.547
123.937
121.938
116.694
116.246
114.785
114.558
114.435
110.298
105.080
99.861
94.131
89.270
76.999
66.361
62.227
56.185
56.163
55.875
49.787
O
OH
O
O
N
N=N
O
O
O
O
123
220 210 200 190 180 170 160 150 140 130 120 110 100 90 80 70 60 50 40 30 20 10 0 ppm

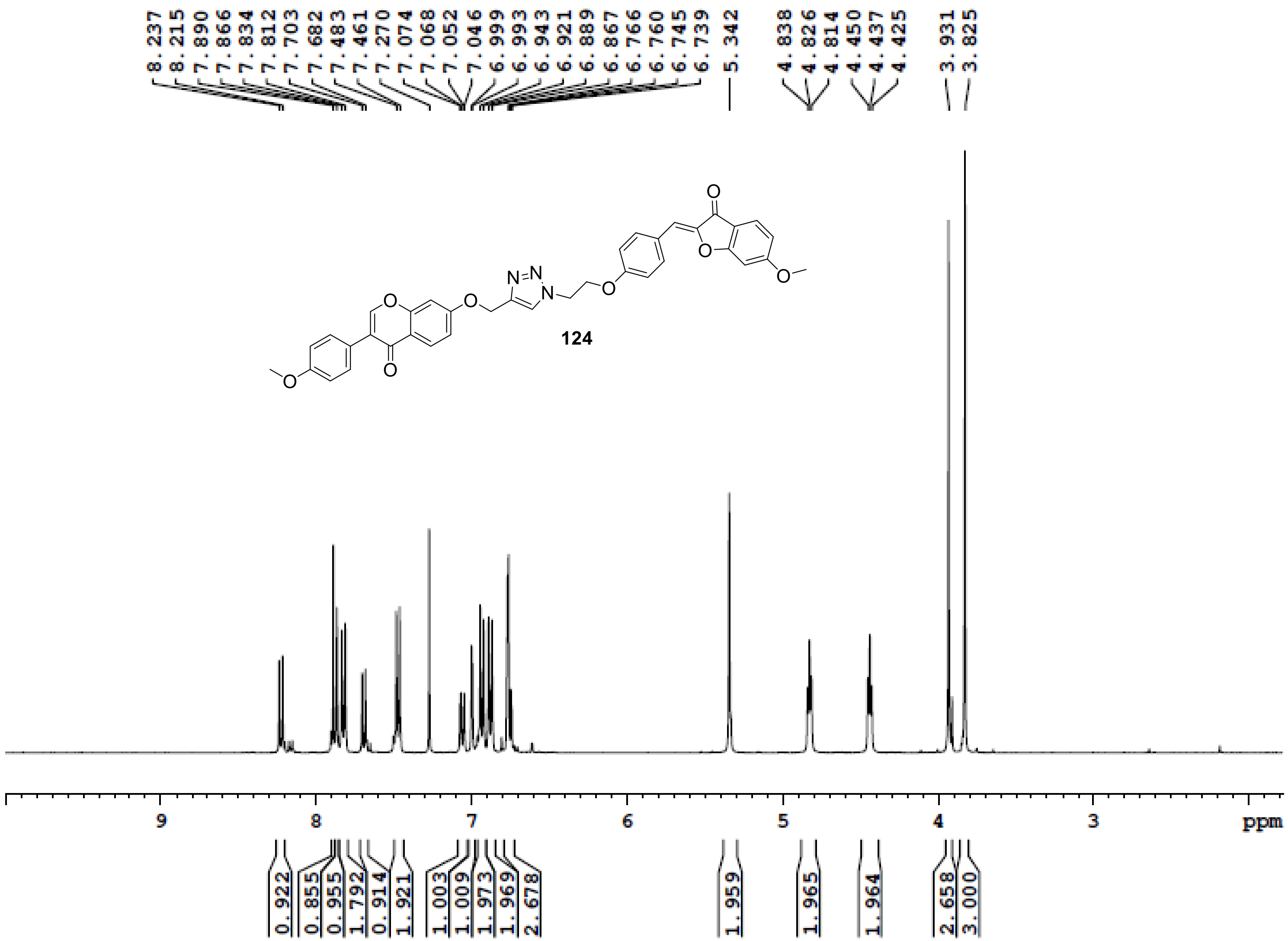
8.237
8.215
7.890
7.866
7.834
7.812
7.703
7.682
7.483
7.461
7.270
7.074
7.068
7.052
7.046
6.999
6.993
6.943
6.921
6.889
6.867
6.766
6.760
6.745
6.739
5.342
4.838
4.826
4.814
4.450
4.437
4.425
3.931
3.825
124
9
8
7
6
5
4
3
ppm
0.922
0.855
0.955
1.792
0.914
1.921
1.003
1.009
1.973
1.969
2.678
1.959
1.965
1.964
2.658
3.000

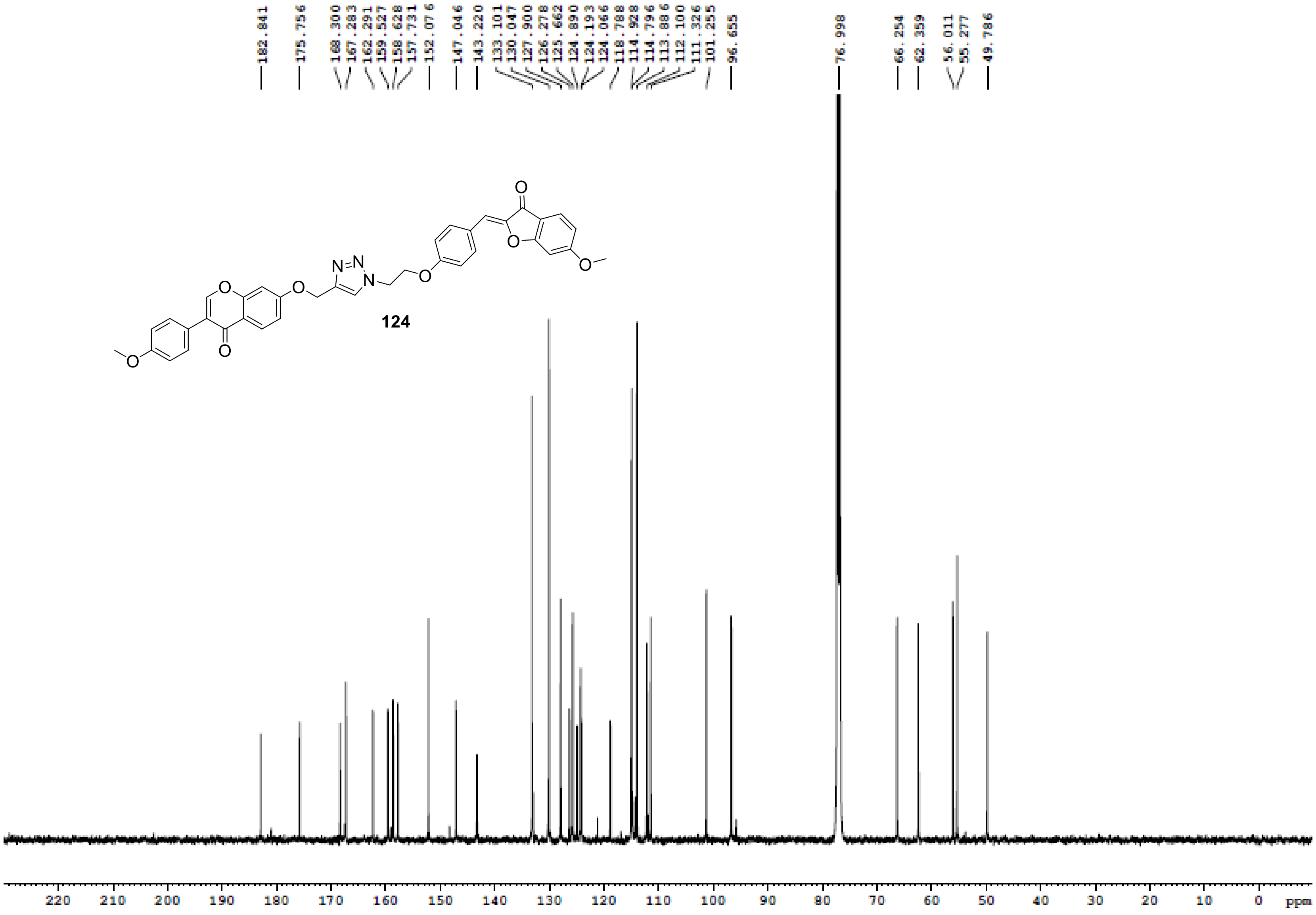
182.841
175.756
168.300
167.283
162.291
159.527
158.628
157.731
152.076
147.046
143.220
133.101
130.047
127.900
126.278
125.662
124.890
124.193
124.066
118.788
114.928
114.796
113.886
112.100
111.326
101.255
96.655
76.998
66.254
62.359
56.011
55.277
49.786
124
ppm

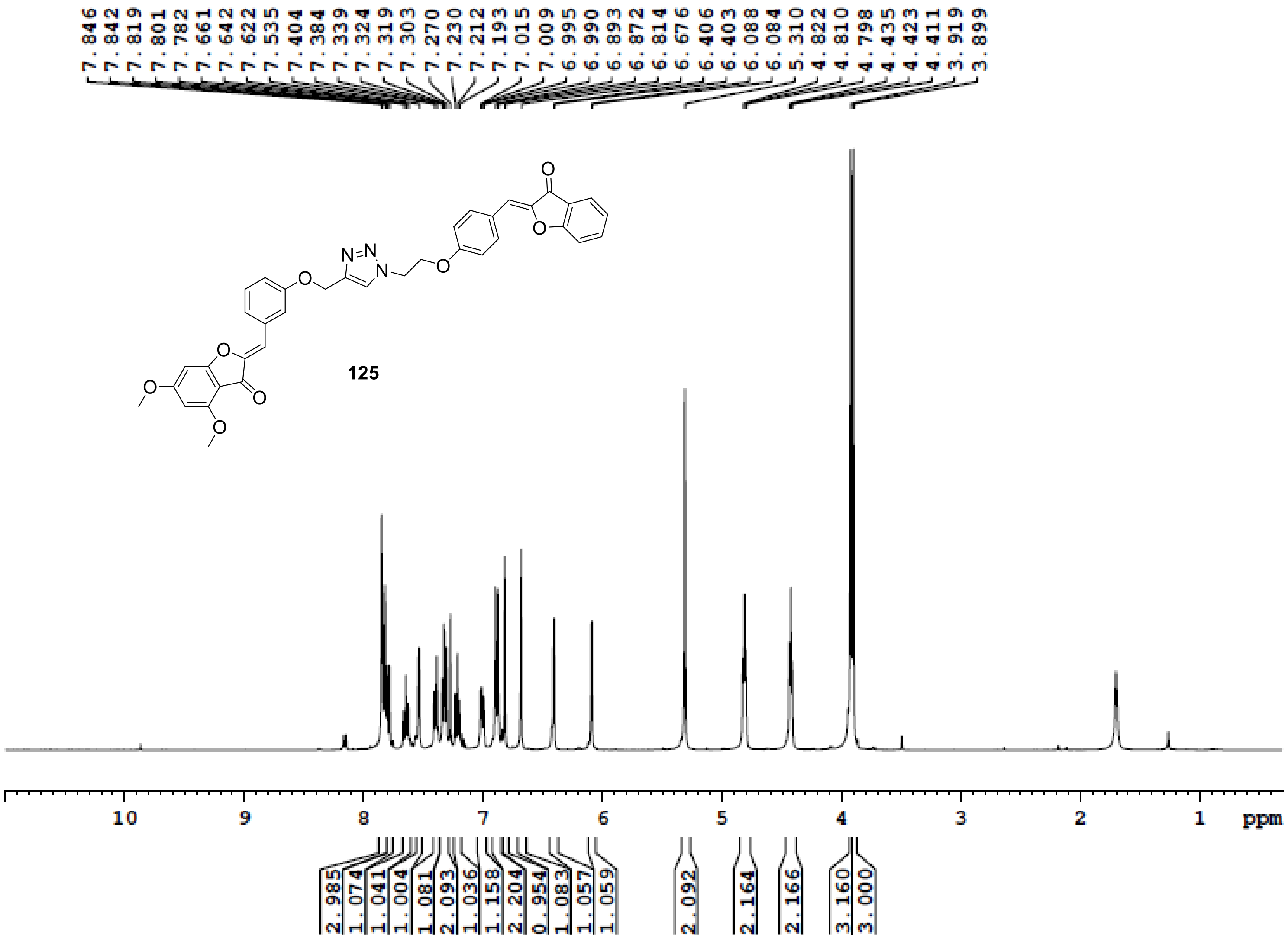
7.846
7.842
7.819
7.801
7.782
7.661
7.642
7.622
7.535
7.404
7.384
7.339
7.324
7.319
7.303
7.270
7.230
7.212
7.193
7.015
7.009
6.995
6.990
6.893
6.872
6.814
6.676
6.406
6.403
6.088
6.084
5.310
4.822
4.810
4.798
4.435
4.423
4.411
3.919
3.899
125
10
9
8
7
6
5
4
3
2
1
ppm
2.985
1.074
1.041
1.004
1.081
2.093
1.036
1.158
2.204
0.954
1.083
1.057
1.059
2.092
2.164
2.166
3.160
3.000

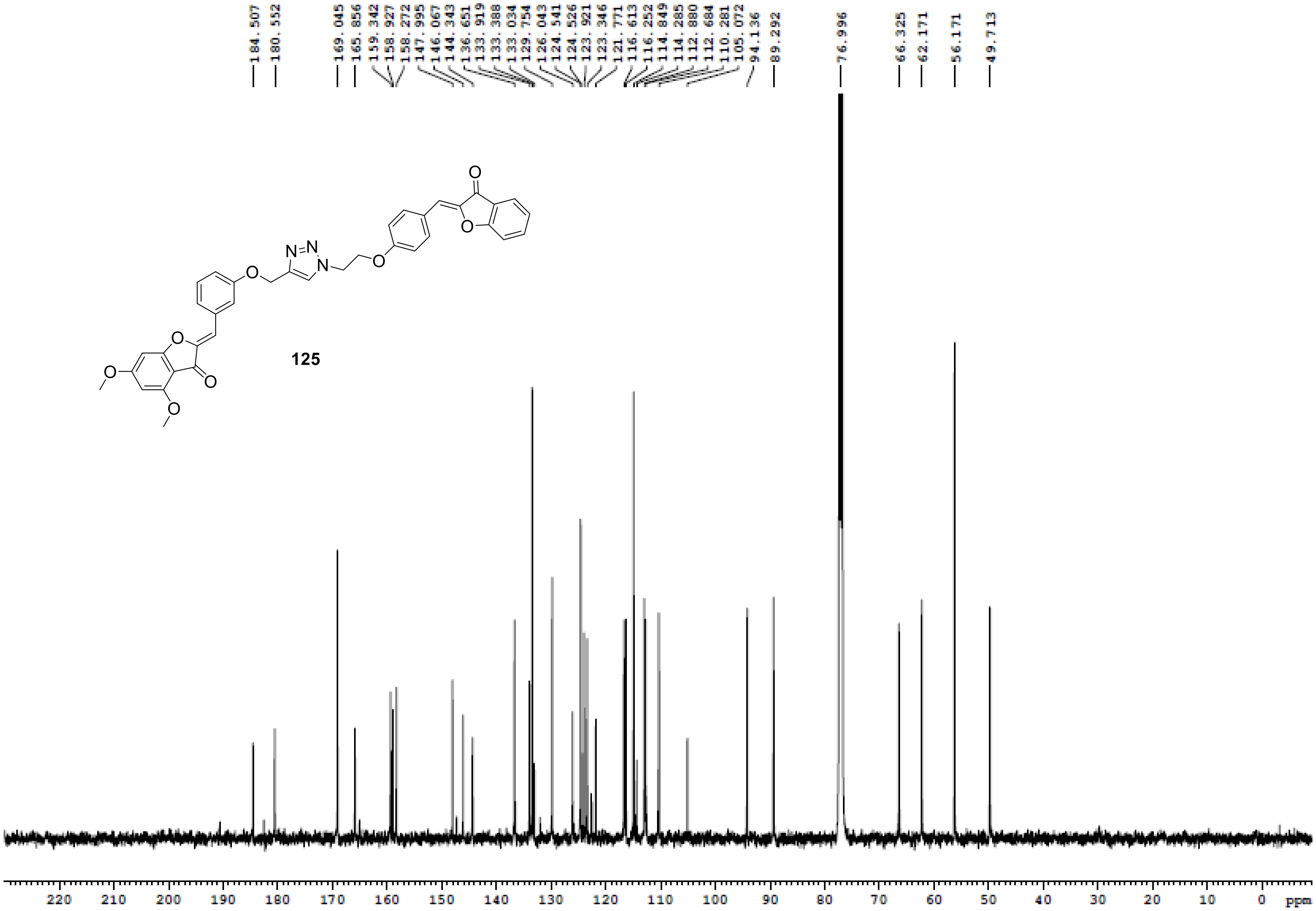
184.507
180.552
169.045
165.856
159.342
158.927
158.272
147.995
146.067
144.343
136.651
133.919
133.388
133.034
129.754
126.043
124.541
124.526
123.921
123.346
121.771
116.613
116.252
114.849
114.285
112.880
112.684
110.281
105.072
94.136
89.292
76.996
66.325
62.171
56.171
49.713
125
220 210 200 190 180 170 160 150 140 130 120 110 100 90 80 70 60 50 40 30 20 10 0 ppm

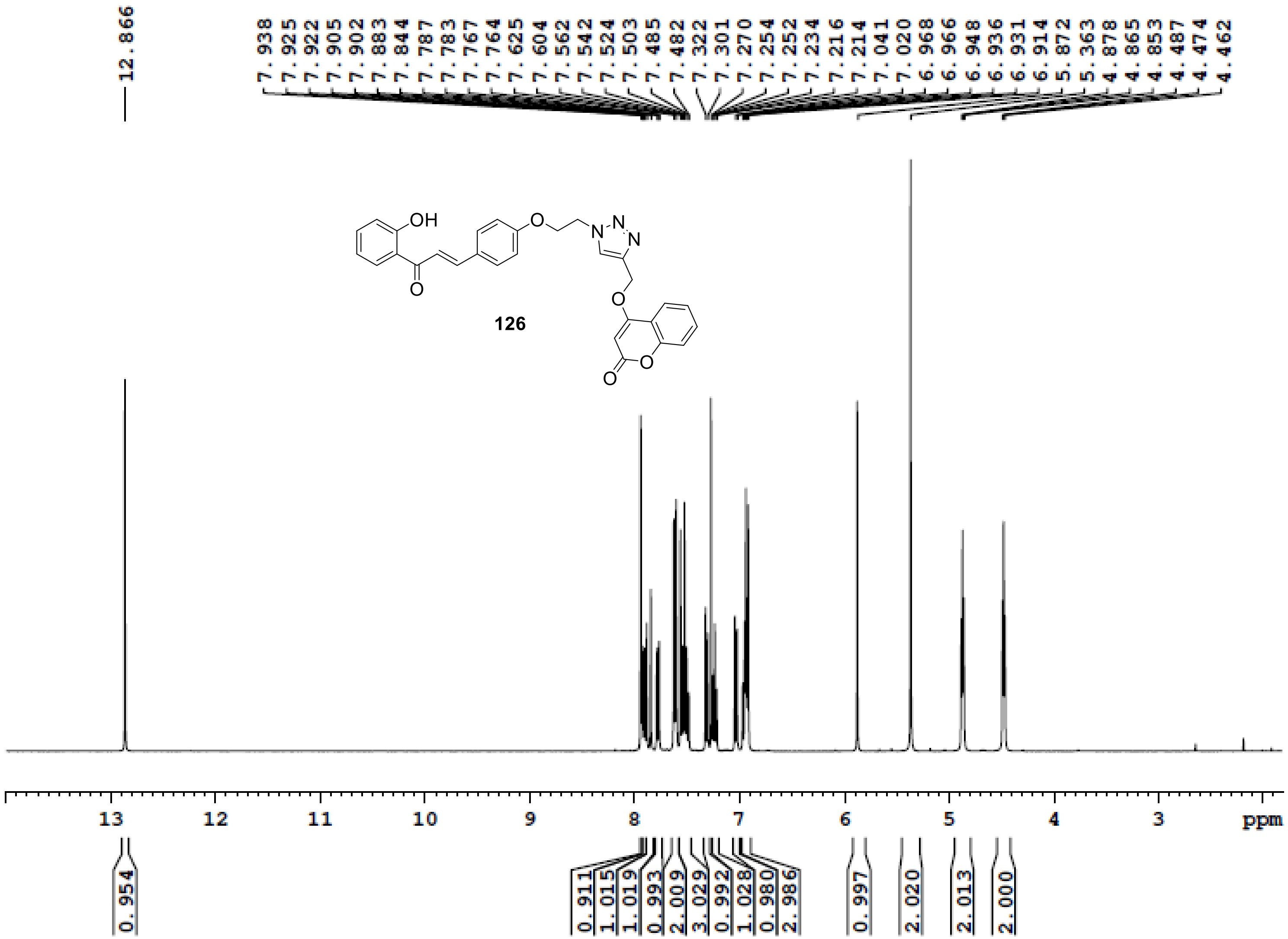

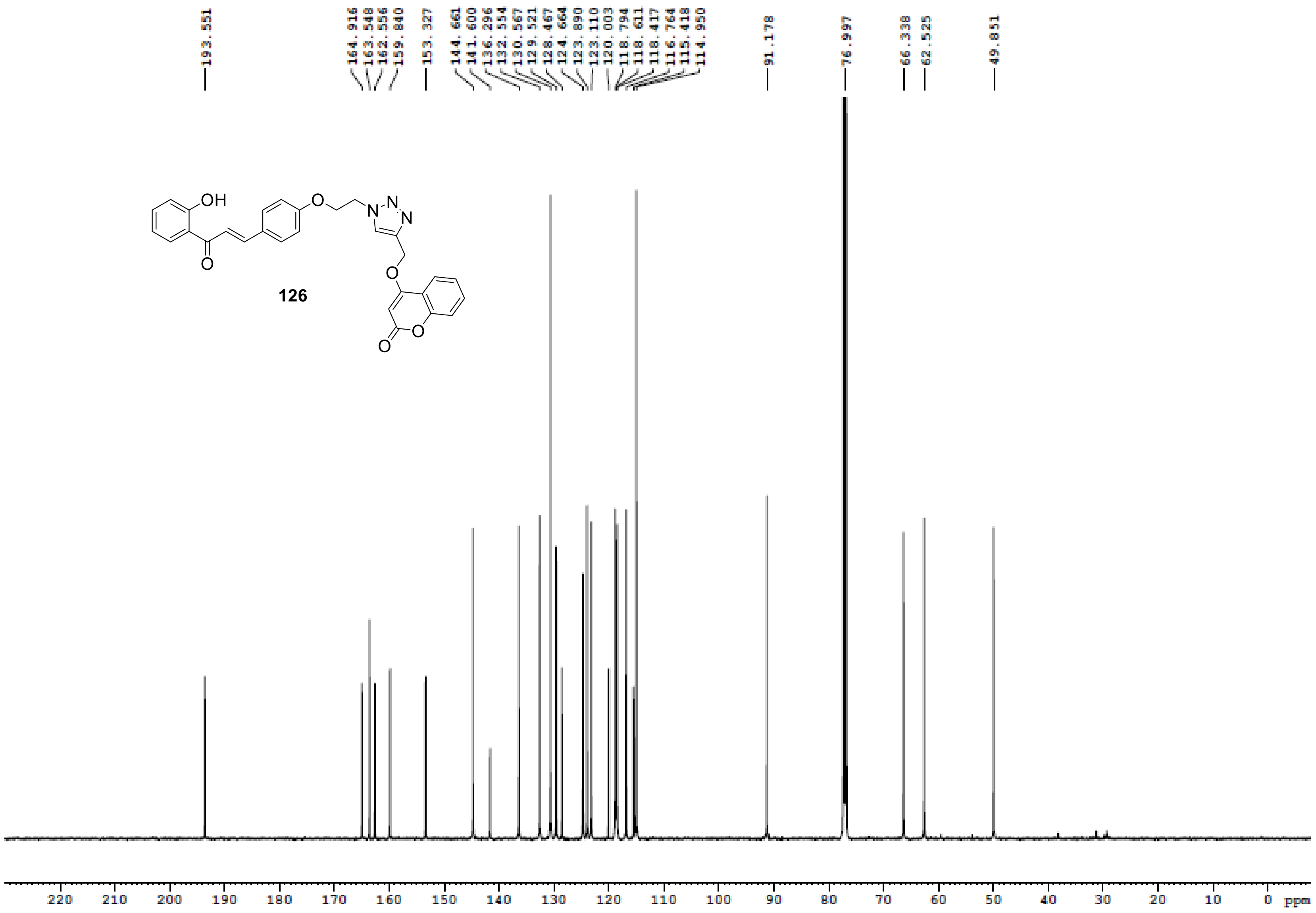
193.551
164.916
163.548
162.556
159.840
153.327
144.661
141.600
136.296
132.554
130.567
129.521
128.467
124.664
123.890
123.110
120.003
118.794
118.611
118.417
116.764
115.418
114.950
91.178
76.997
66.338
62.525
49.851
OH
O
N
N
N
O
O
O
126
220 210 200 190 180 170 160 150 140 130 120 110 100 90 80 70 60 50 40 30 20 10 0 ppm

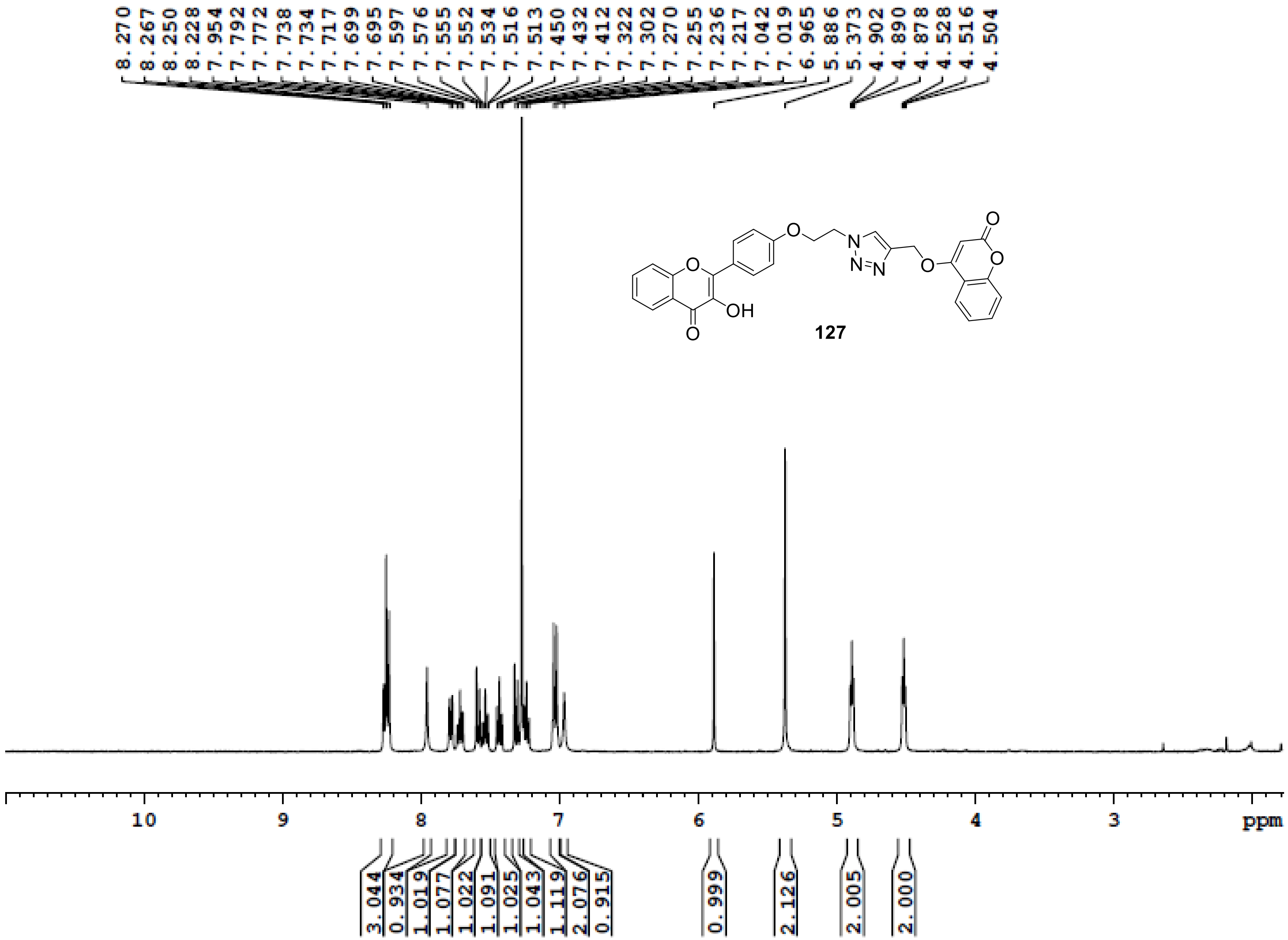
8.270
8.267
8.250
8.228
7.954
7.792
7.772
7.738
7.734
7.717
7.699
7.695
7.597
7.576
7.555
7.552
7.534
7.516
7.513
7.450
7.432
7.412
7.322
7.302
7.270
7.255
7.236
7.217
7.042
7.019
6.965
5.886
5.373
4.902
4.890
4.878
4.528
4.516
4.504
O
OH
O
O
N
N
N
O
O
O
127
10
9
8
7
6
5
4
3
ppm
3.044
0.934
1.019
1.077
1.022
1.091
1.025
1.043
1.119
2.076
0.915
0.999
2.126
2.005
2.000

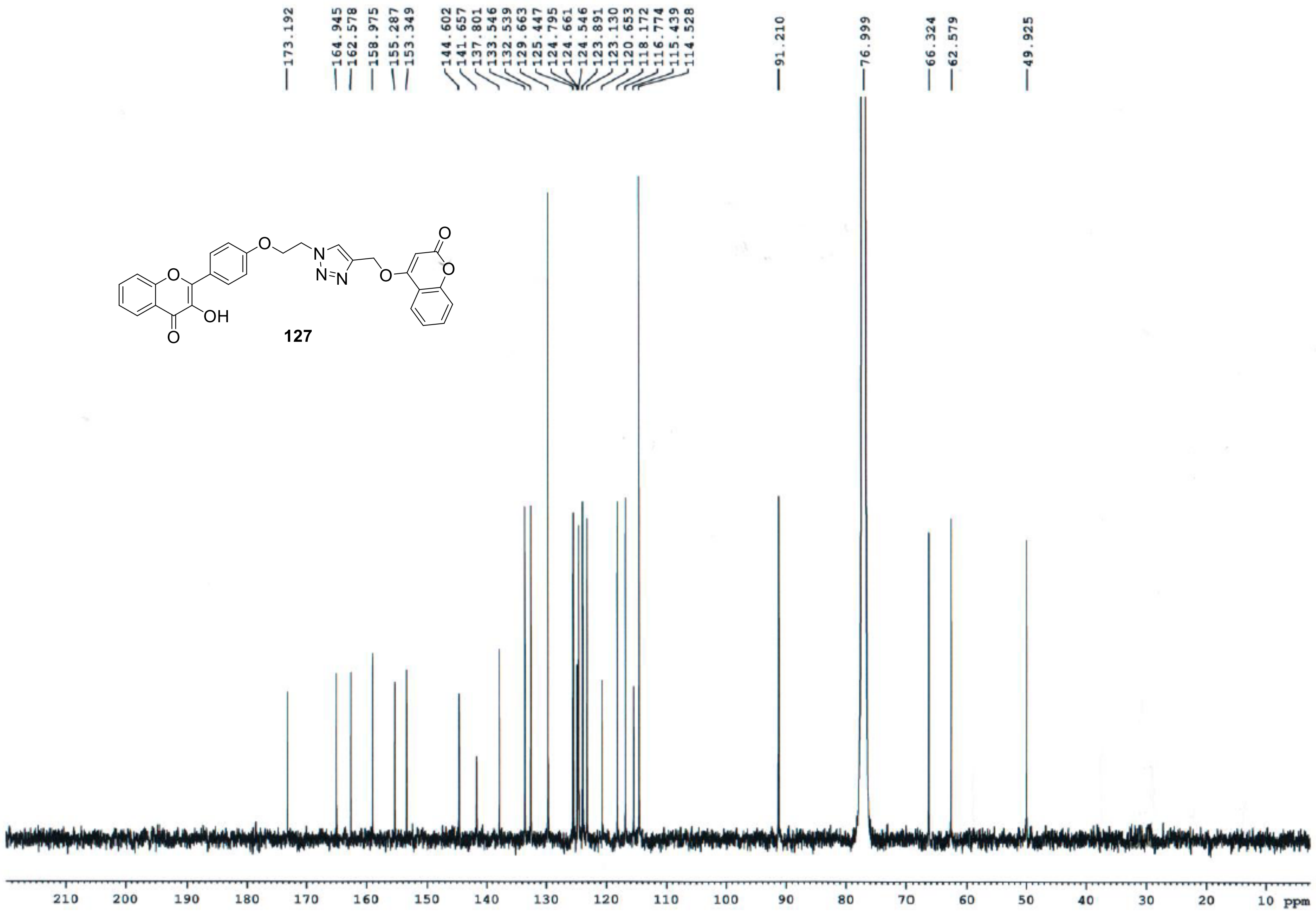
173.192
164.945
162.578
158.975
155.287
153.349
144.602
141.657
137.801
133.546
132.539
129.663
125.447
124.795
124.661
124.546
123.891
123.130
120.653
118.172
116.774
115.439
114.528
91.210
76.999
66.324
62.579
49.925
O
OH
N
N
N
127
210 200 190 180 170 160 150 140 130 120 110 100 90 80 70 60 50 40 30 20 10 ppm

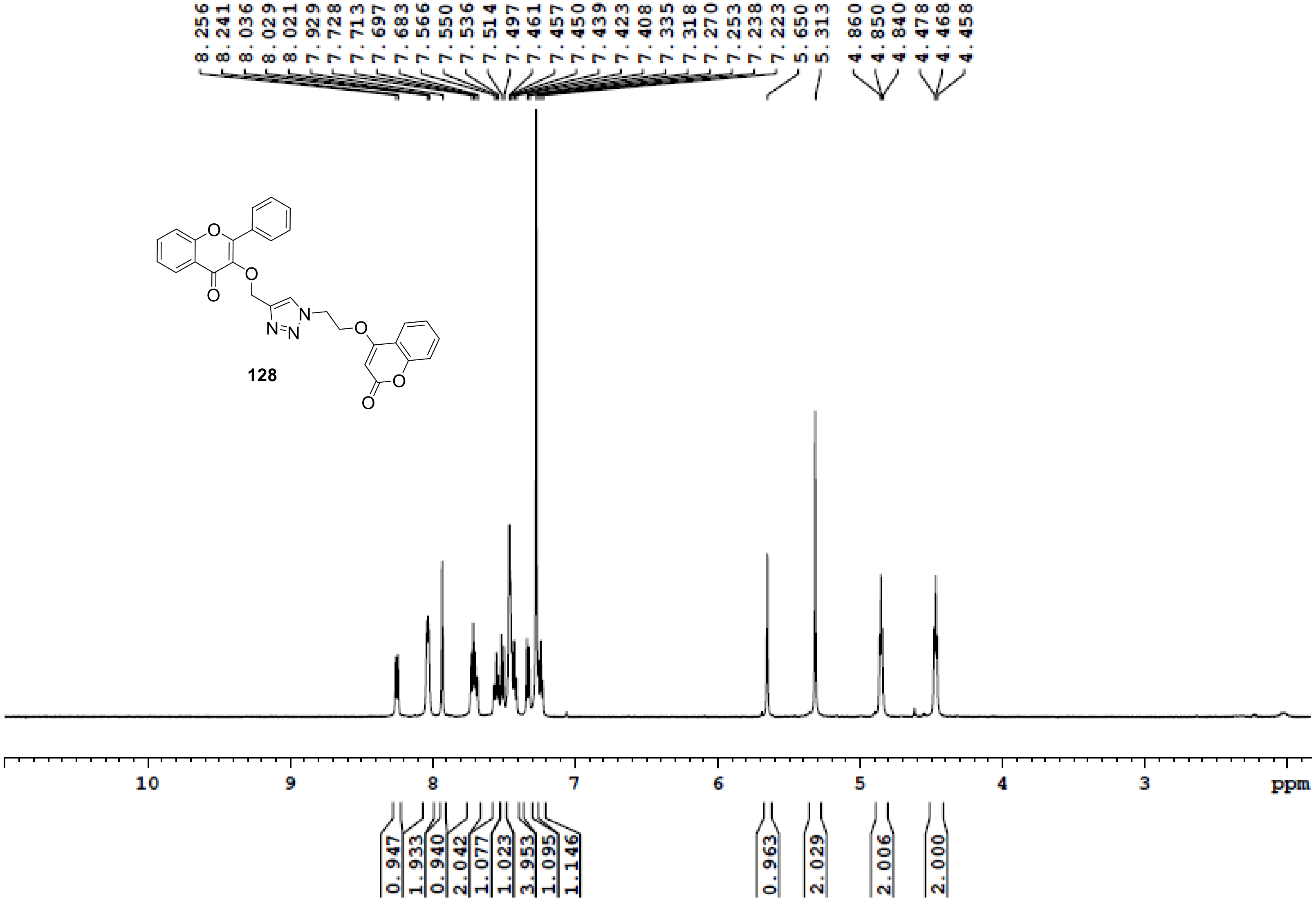
8.256
8.241
8.036
8.029
8.021
7.929
7.728
7.713
7.697
7.683
7.566
7.550
7.536
7.514
7.497
7.461
7.457
7.450
7.439
7.423
7.408
7.335
7.318
7.270
7.253
7.238
7.223
5.650
5.313
4.860
4.850
4.840
4.478
4.468
4.458
N
N
N
O
O
O
O
O
O
128
10
9
8
7
6
5
4
3
ppm
0.947
1.933
0.940
2.042
1.077
1.023
3.953
1.095
1.146
0.963
2.029
2.006
2.000

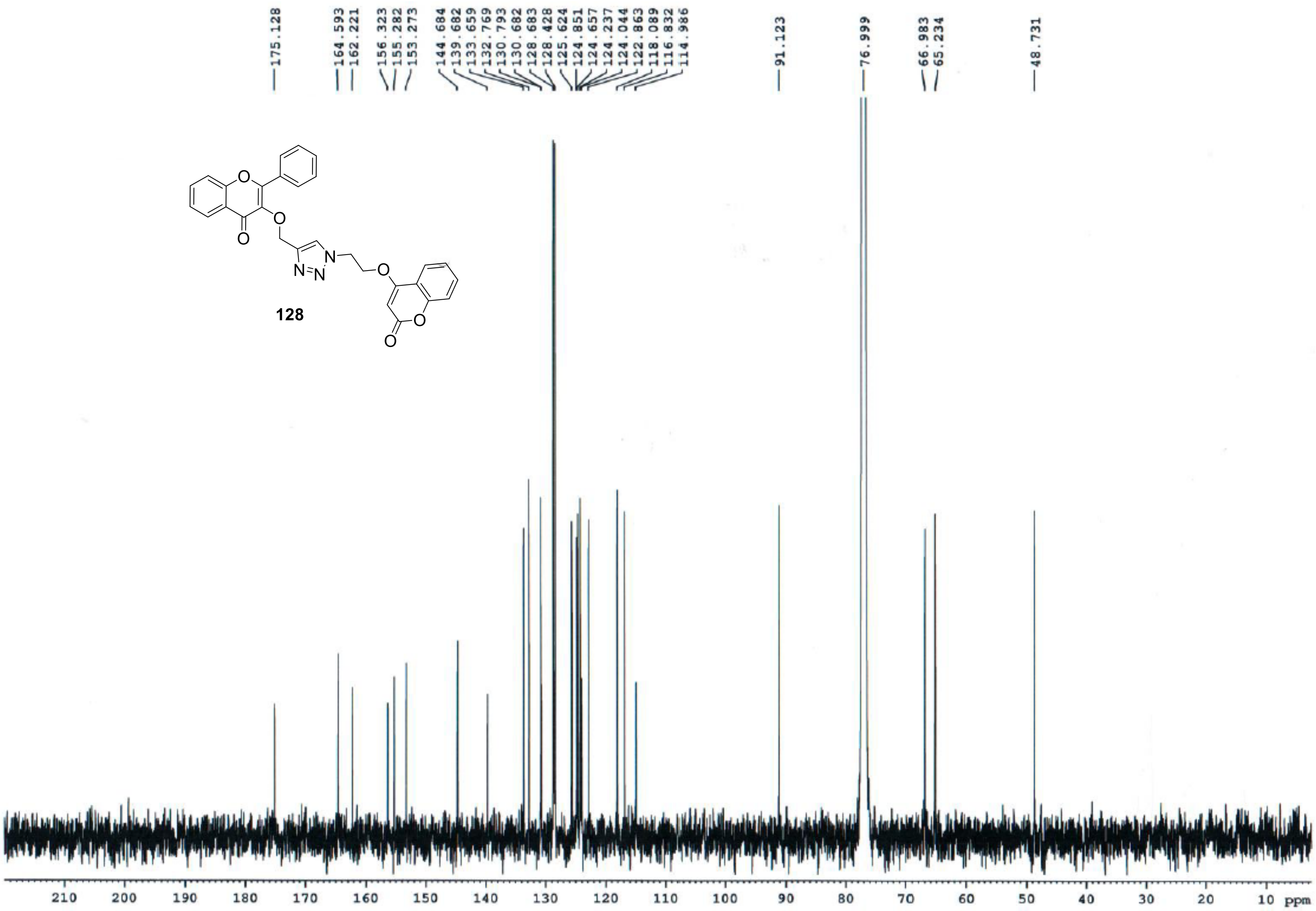
175.128
164.593
162.221
156.323
155.282
153.273
144.684
139.682
133.659
132.769
130.793
130.682
128.683
128.428
125.624
124.851
124.657
124.237
124.044
122.863
118.089
116.832
114.986
91.123
76.999
66.983
65.234
48.731
128
210 200 190 180 170 160 150 140 130 120 110 100 90 80 70 60 50 40 30 20 10 ppm

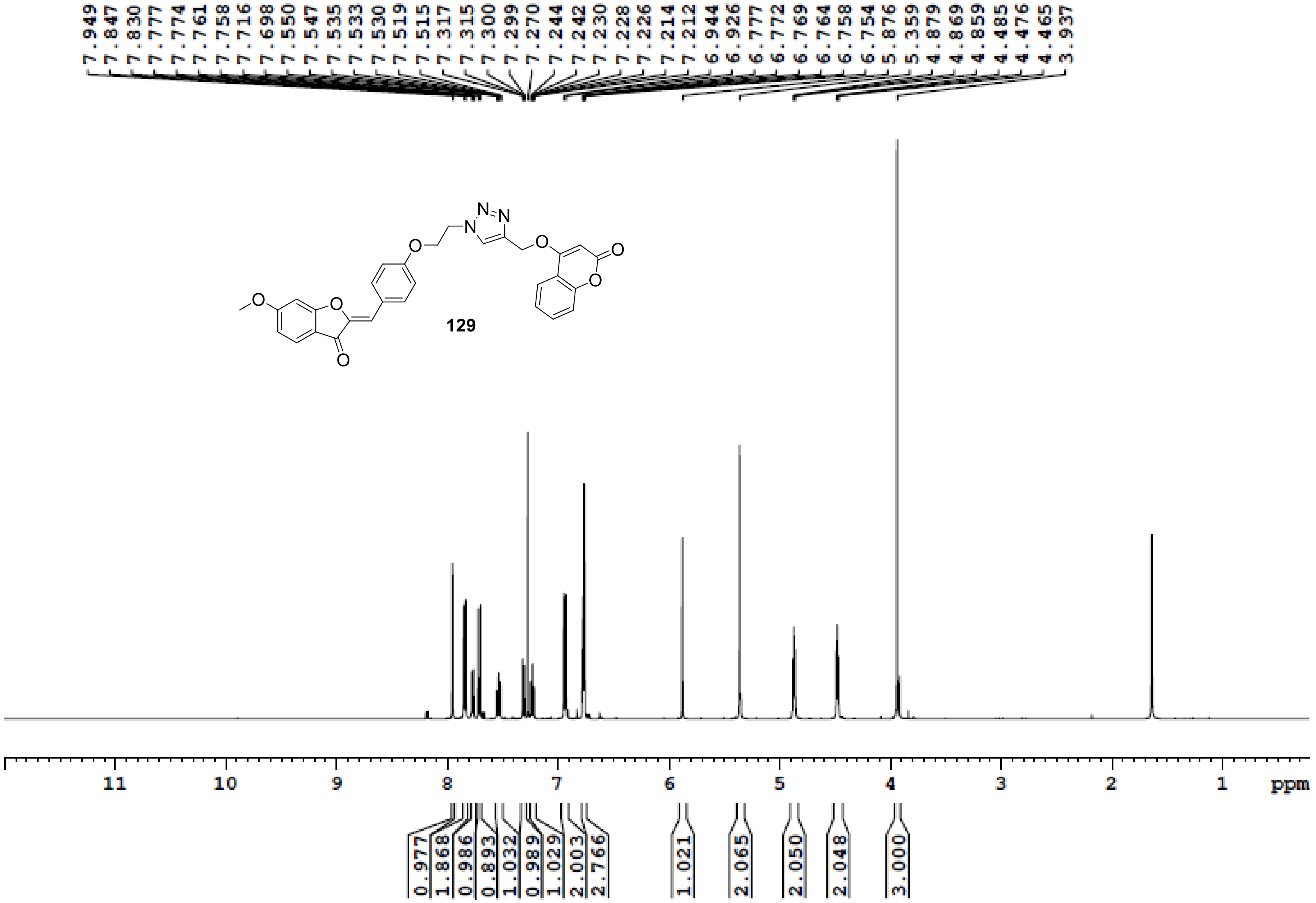

7.949
7.847
7.830
7.777
7.774
7.761
7.758
7.716
7.698
7.550
7.547
7.535
7.533
7.530
7.519
7.515
7.317
7.315
7.300
7.299
7.270
7.244
7.242
7.230
7.228
7.226
7.214
7.212
6.944
6.926
6.777
6.772
6.769
6.764
6.758
6.754
5.876
5.359
4.879
4.869
4.859
4.485
4.476
4.465
3.937
129
11 10 9 8 7 6 5 4 3 2 1 ppm
0.977
1.868
0.986
0.893
1.032
0.989
1.029
2.003
2.766
1.021
2.065
2.050
2.048
3.000

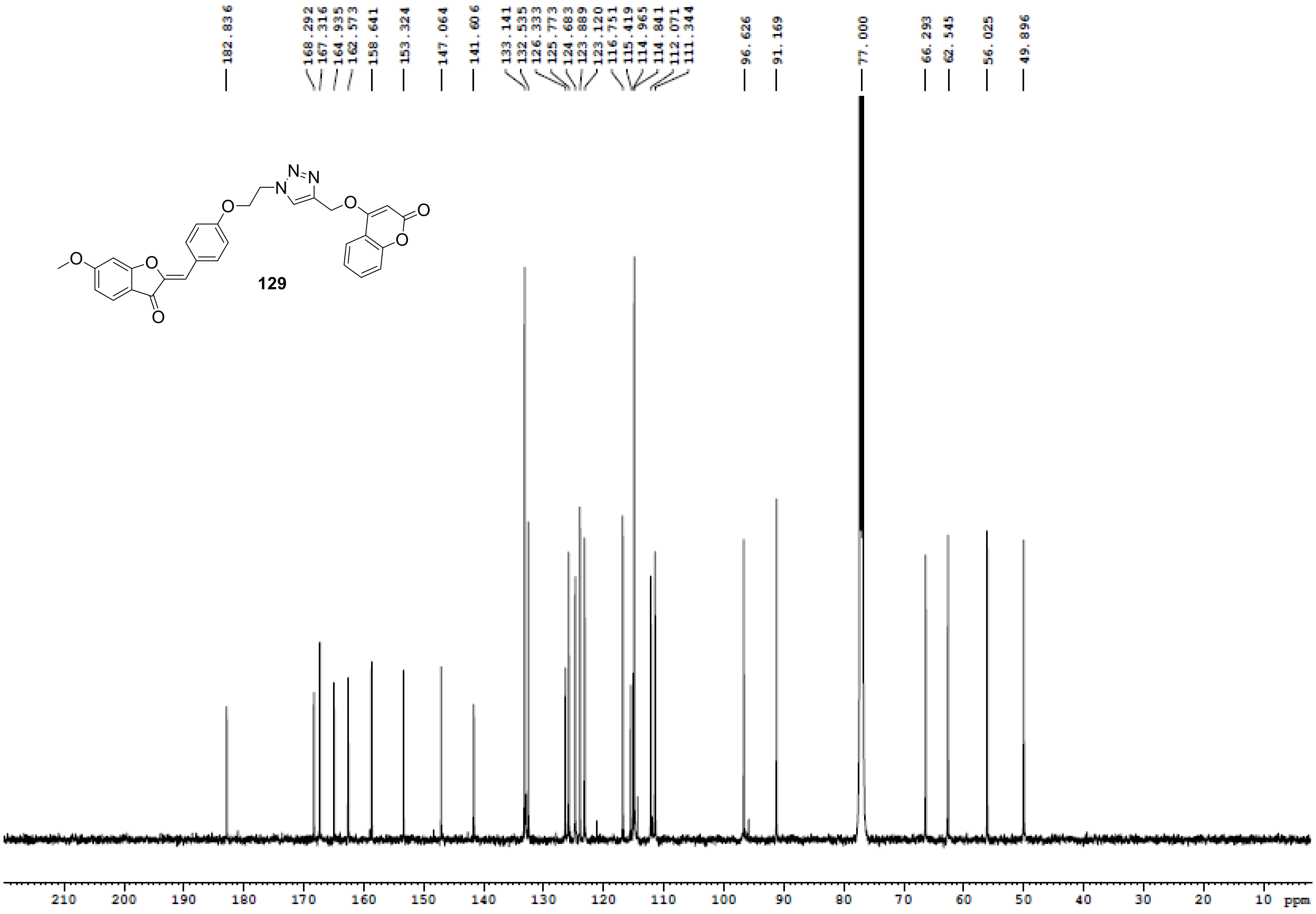
182.836
168.292
167.316
164.935
162.573
158.641
153.324
147.064
141.606
133.141
132.535
126.333
125.773
124.683
123.889
123.120
116.751
115.419
114.965
114.841
112.071
111.344
96.626
91.169
77.000
66.293
62.545
56.025
49.896
129
210 200 190 180 170 160 150 140 130 120 110 100 90 80 70 60 50 40 30 20 10 ppm

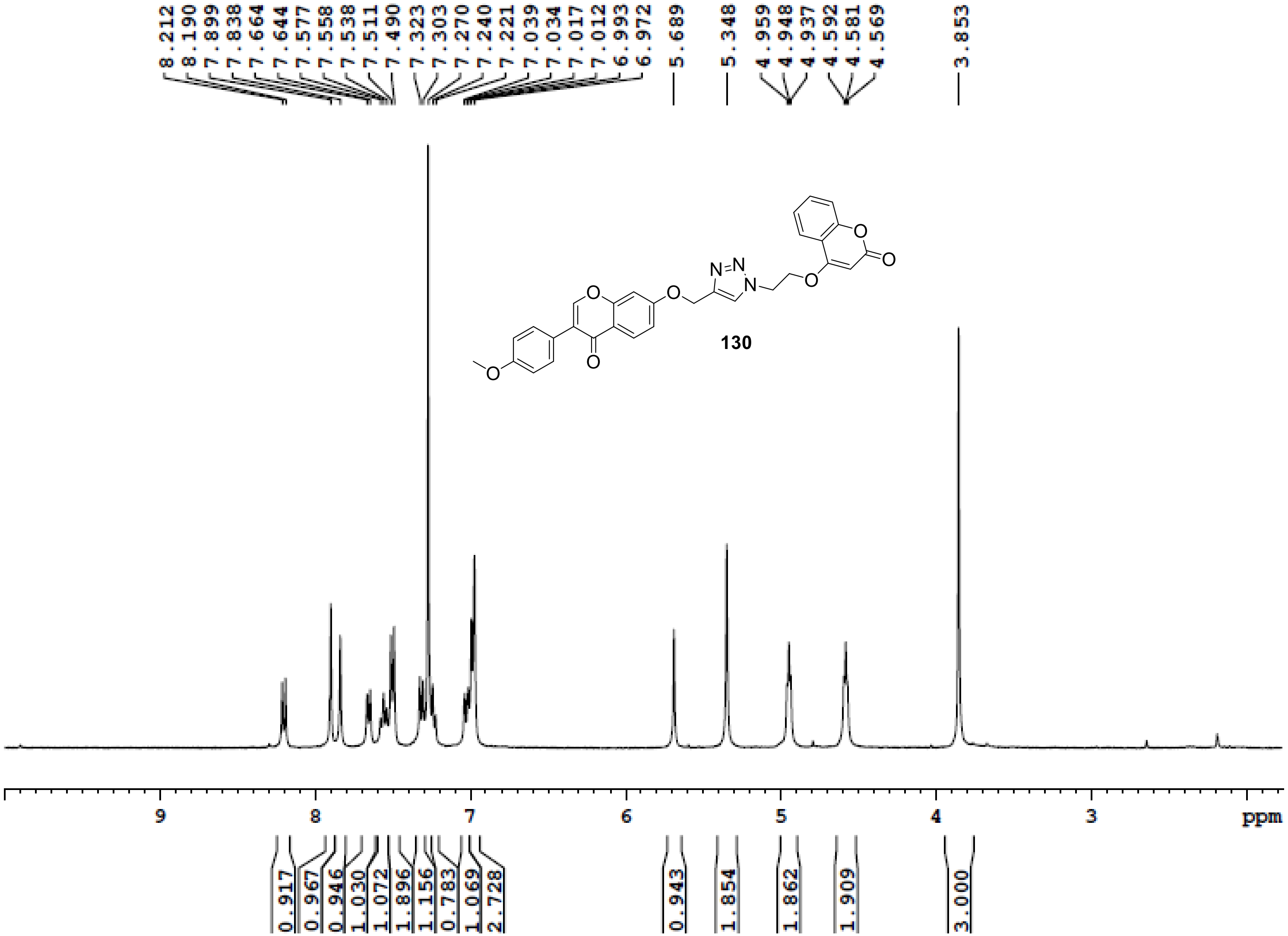
8.212 8.190 7.899 7.838 7.664 7.644 7.577 7.558 7.538 7.511 7.490 7.323 7.303 7.270 7.240 7.221 7.039 7.034 7.017 7.012 6.993 6.972
5.689
5.348
4.959 4.948 4.937
4.592 4.581 4.569
3.853
N=N
N
O
130
9
8
7
6
5
4
3
ppm
0.917 0.967 0.946 1.030 1.072 1.896 1.156 0.783 1.069 2.728
0.943
1.854
1.862
1.909
3.000

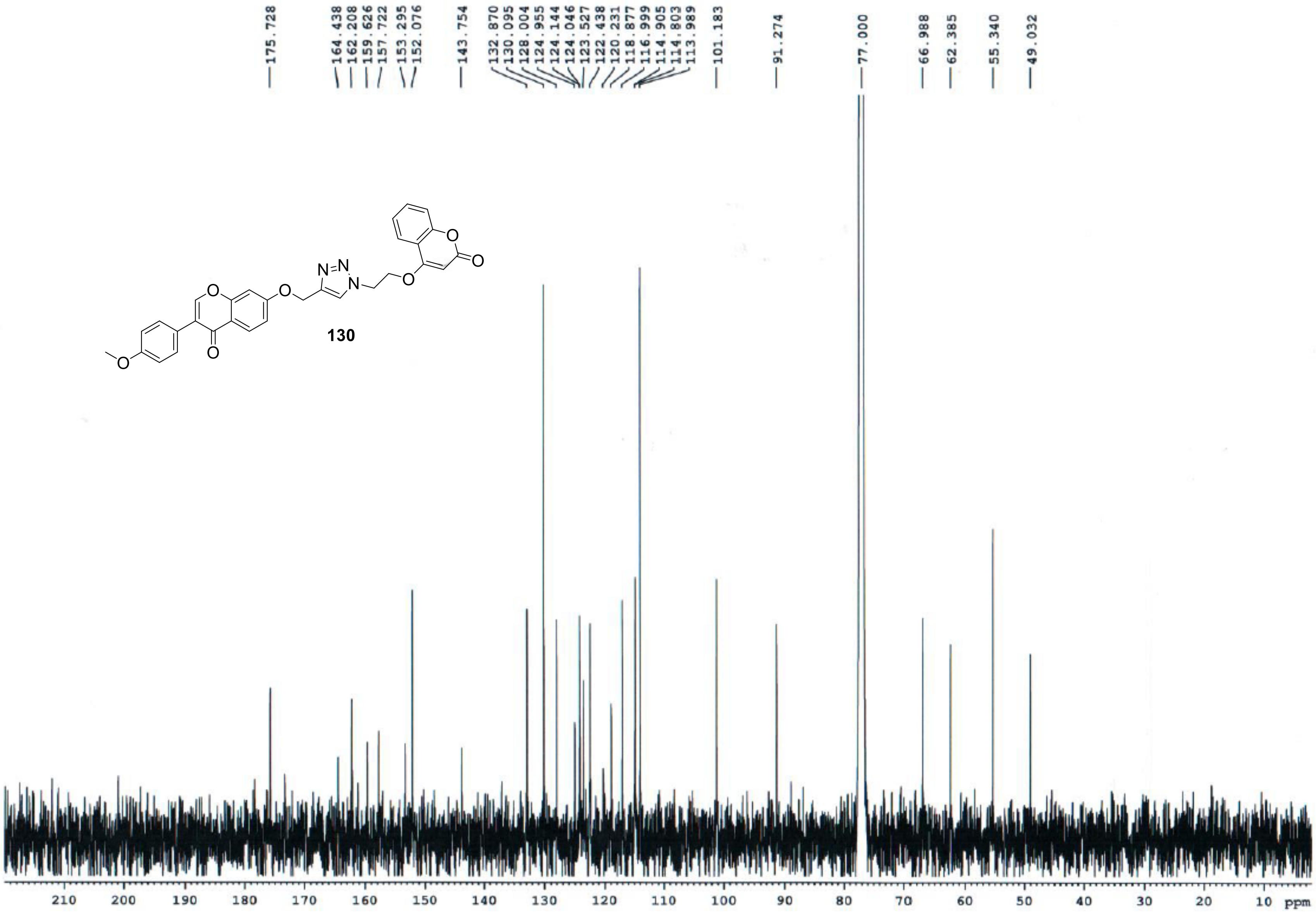
175.728
164.438
162.208
159.626
157.722
153.295
152.076
143.754
132.870
130.095
128.004
124.955
124.144
124.046
123.527
122.438
120.231
118.877
116.999
114.905
114.803
113.989
101.183
91.274
77.000
66.988
62.385
55.340
49.032
210 200 190 180 170 160 150 140 130 120 110 100 90 80 70 60 50 40 30 20 10 ppm

130

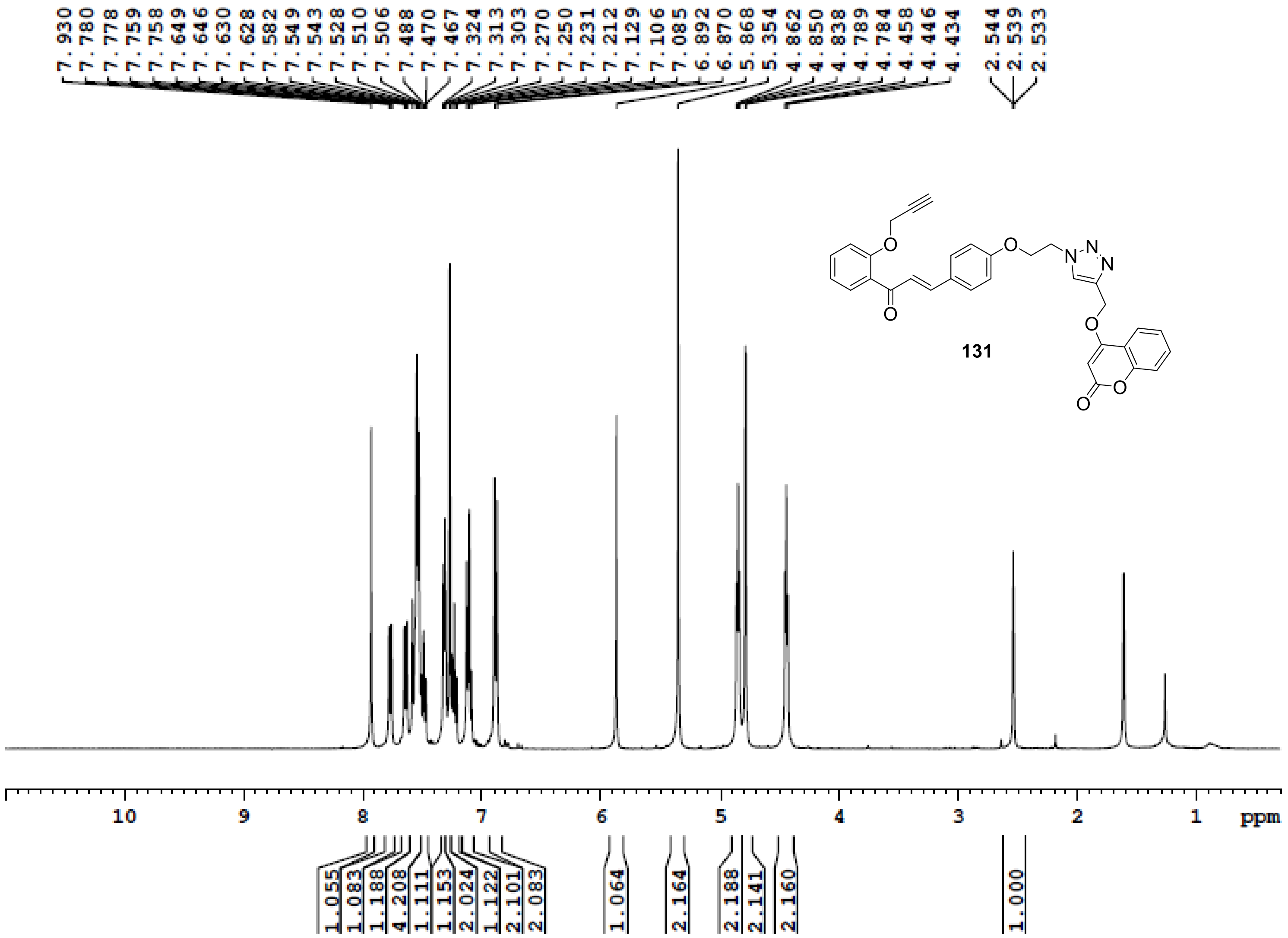
131
ppm

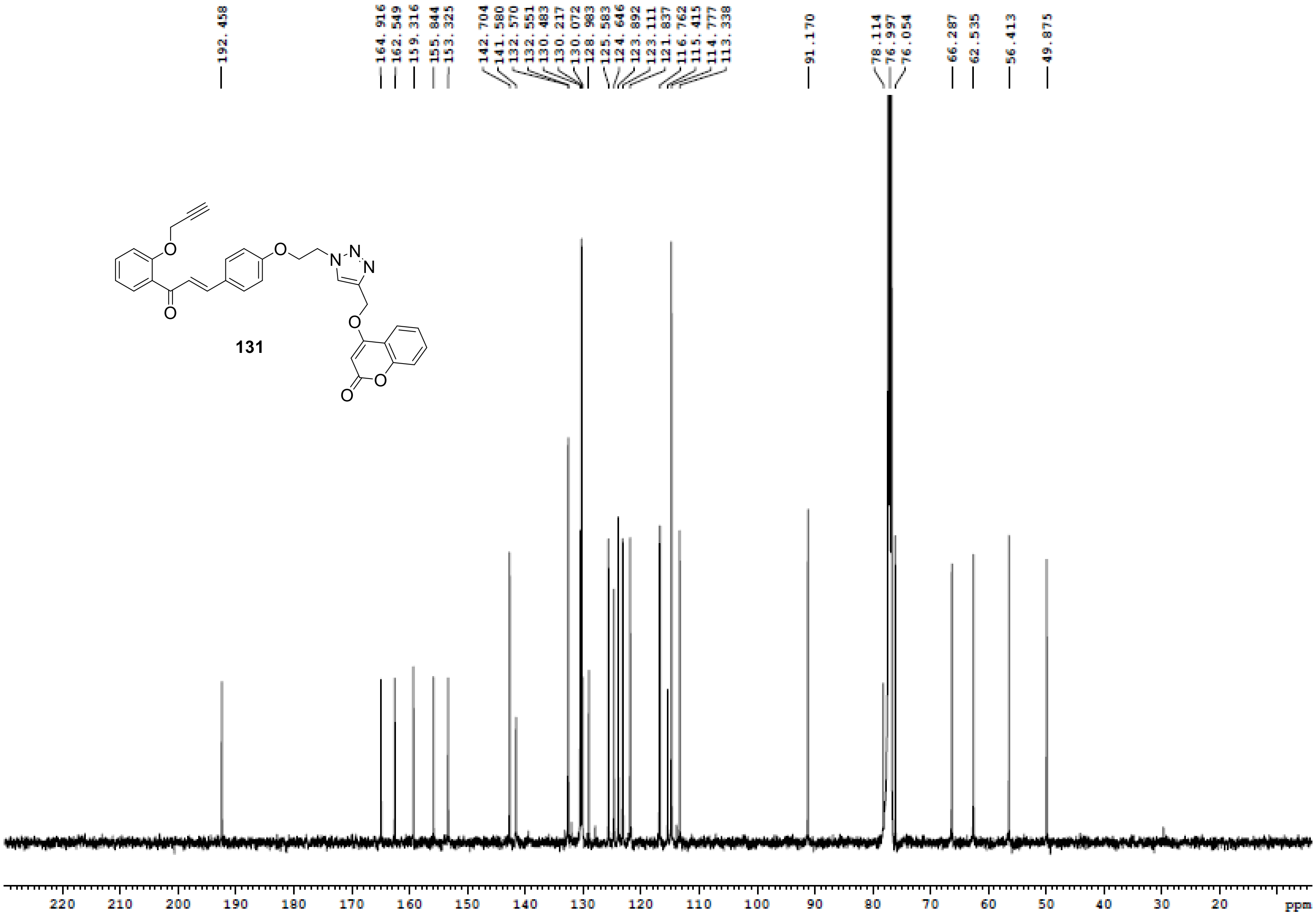

192.458
164.916
162.549
159.316
155.844
153.325
142.704
141.580
132.570
132.551
130.483
130.217
130.072
128.983
125.583
124.646
123.892
123.111
121.837
116.762
115.415
114.777
113.338
91.170
78.114
76.997
76.054
66.287
62.535
56.413
49.875
131
ppm

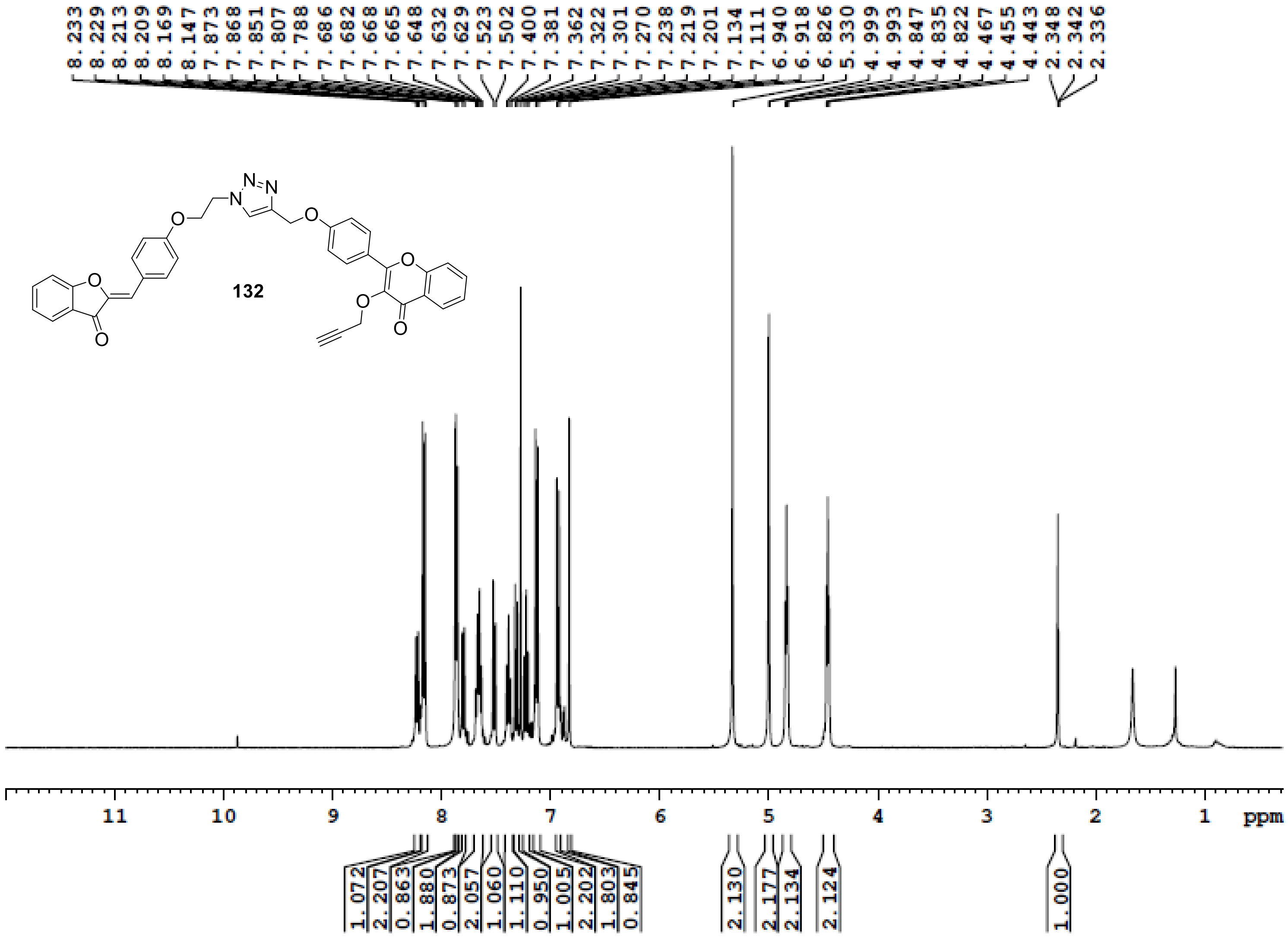
8.233
8.229
8.213
8.209
8.169
8.147
7.873
7.868
7.851
7.807
7.788
7.686
7.682
7.668
7.665
7.648
7.632
7.629
7.523
7.502
7.400
7.381
7.362
7.322
7.301
7.270
7.238
7.219
7.201
7.134
7.111
6.940
6.918
6.826
5.330
4.999
4.993
4.847
4.835
4.822
4.467
4.455
4.443
2.348
2.342
2.336
132
11
10
9
8
7
6
5
4
3
2
1
ppm
1.072
2.207
0.863
1.880
0.873
2.057
1.060
1.110
0.950
1.005
2.202
1.803
0.845
2.130
2.177
2.134
2.124
1.000

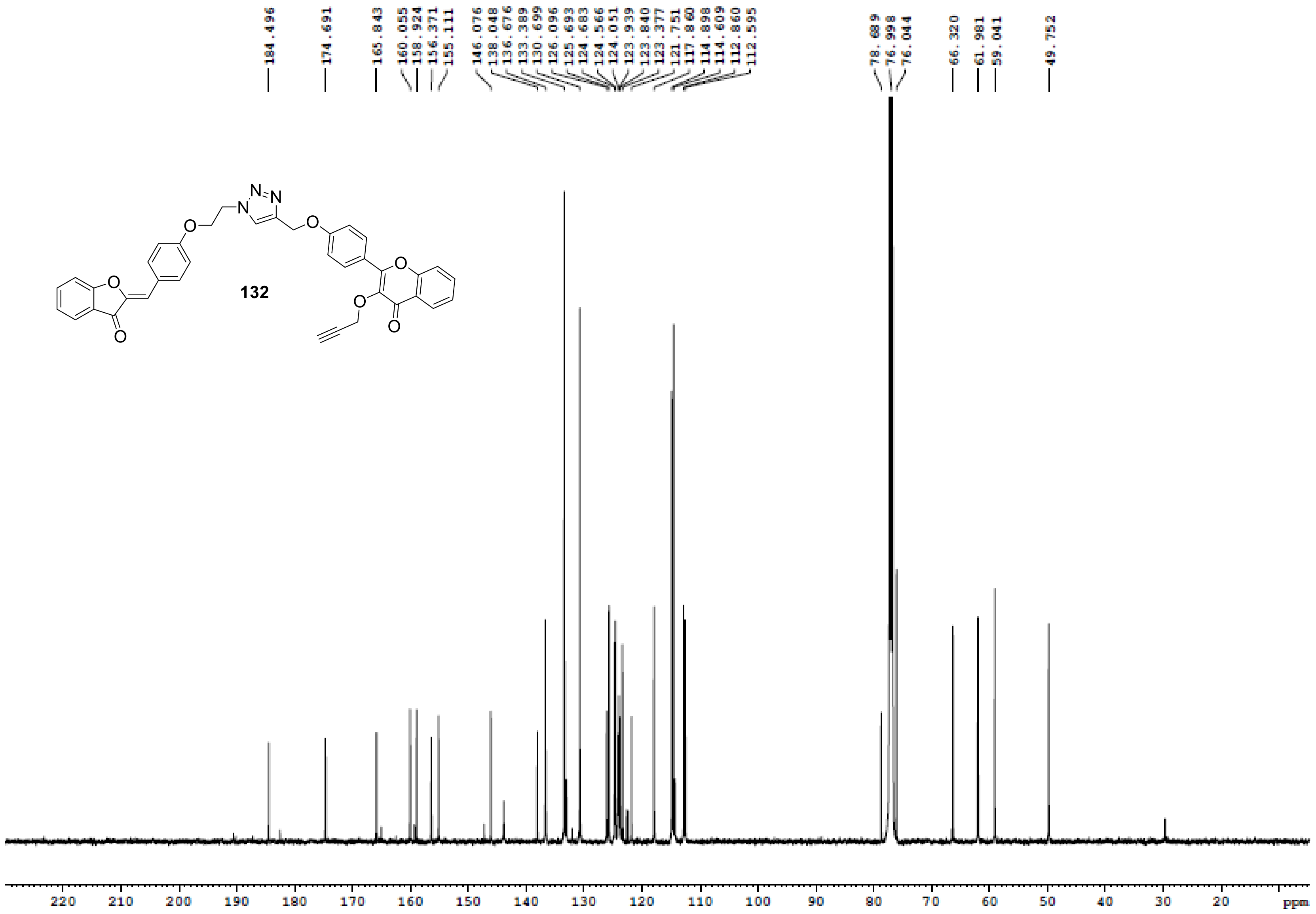
184.496
174.691
165.843
160.055
158.924
156.371
155.111
146.076
138.048
136.676
133.389
130.699
126.096
125.693
124.683
124.566
124.051
123.939
123.840
123.377
121.751
117.860
114.898
114.609
112.860
112.595
78.689
76.998
76.044
66.320
61.981
59.041
49.752
132
220 210 200 190 180 170 160 150 140 130 120 110 100 90 80 70 60 50 40 30 20 ppm

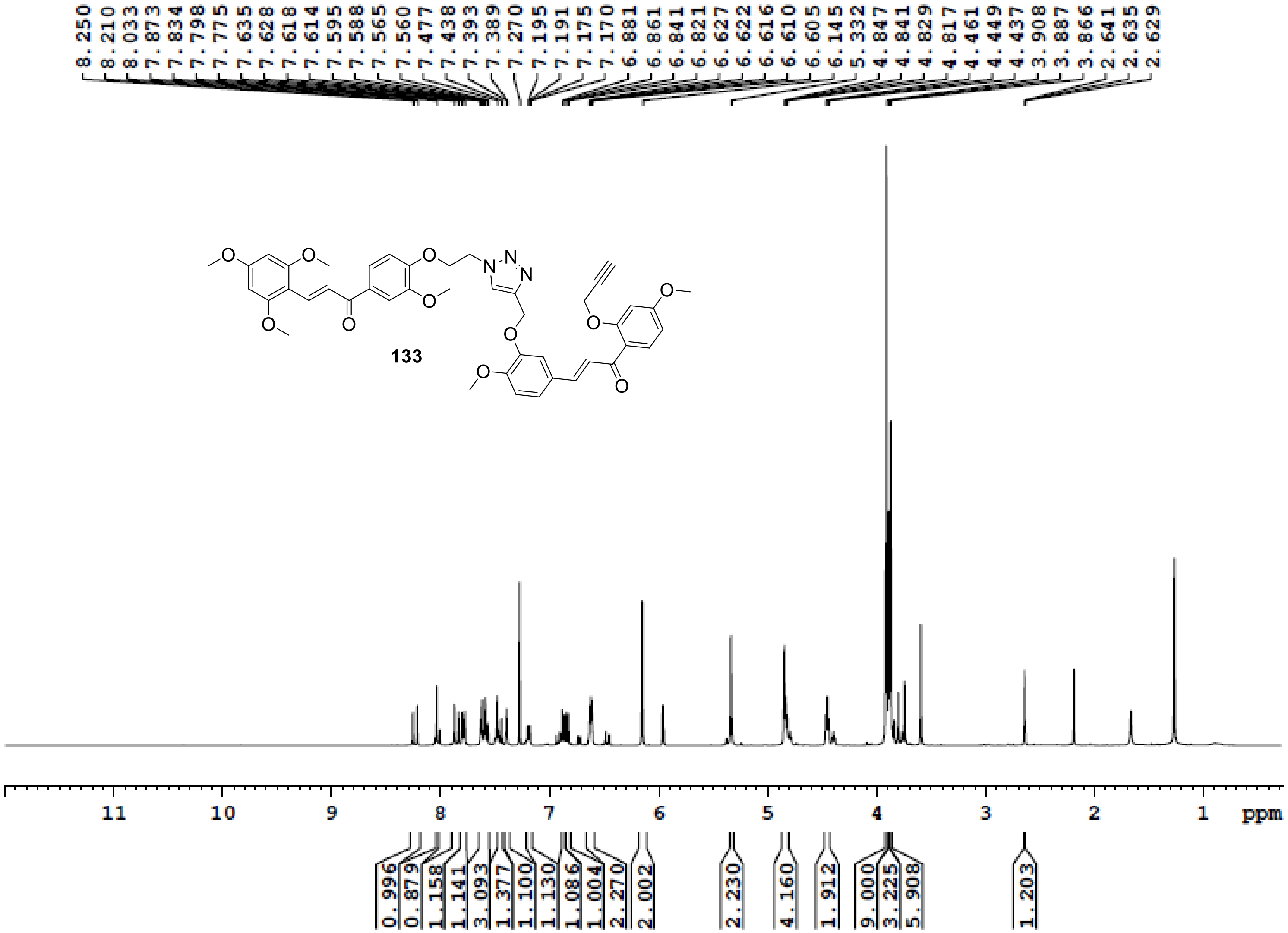
8.250 8.210 8.033 7.873 7.834 7.798 7.775 7.635 7.628 7.618 7.614 7.595 7.588 7.565 7.560 7.477 7.438 7.393 7.389 7.270 7.195 7.191 7.175 7.170 6.881 6.861 6.841 6.821 6.627 6.622 6.616 6.610 6.605 6.145 5.332 4.847 4.841 4.829 4.817 4.461 4.449 4.437 3.908 3.887 3.866 2.641 2.635 2.629
133
11 10 9 8 7 6 5 4 3 2 1 ppm
0.996 0.879 1.158 1.141 3.093 1.377 1.100 1.130 1.086 1.004 2.270 2.002 2.230 4.160 1.912 9.000 3.225 5.908 1.203

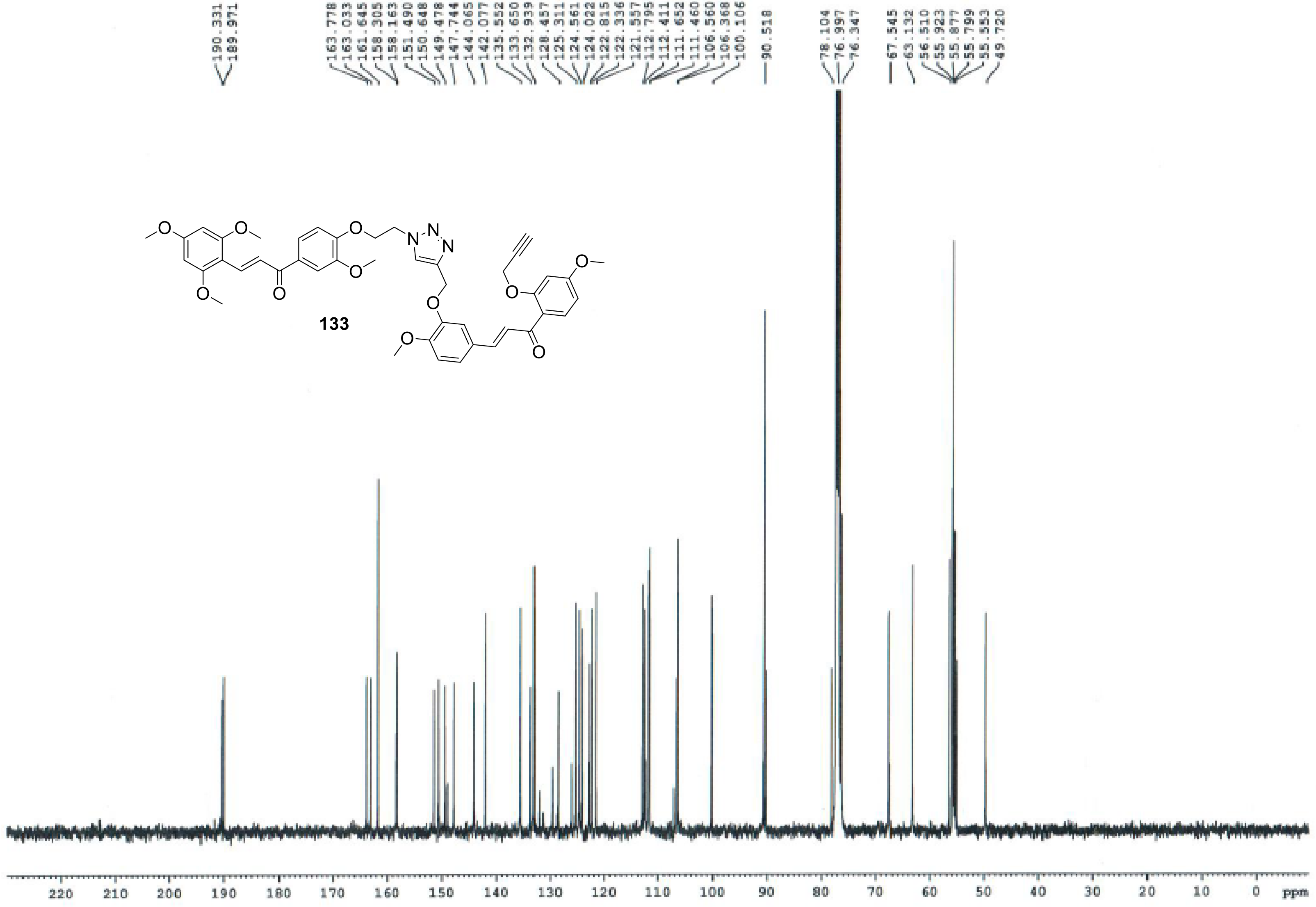
190.331
189.971
163.778
163.033
161.645
158.305
158.163
151.490
150.648
149.478
147.744
144.065
142.077
135.552
133.650
132.939
128.457
125.311
124.561
124.022
122.815
122.336
121.557
112.795
112.411
111.652
111.460
106.560
106.368
100.106
90.518
78.104
76.997
76.347
67.545
63.132
56.510
55.923
55.877
55.799
55.553
49.720
133
220 210 200 190 180 170 160 150 140 130 120 110 100 90 80 70 60 50 40 30 20 10 0 ppm

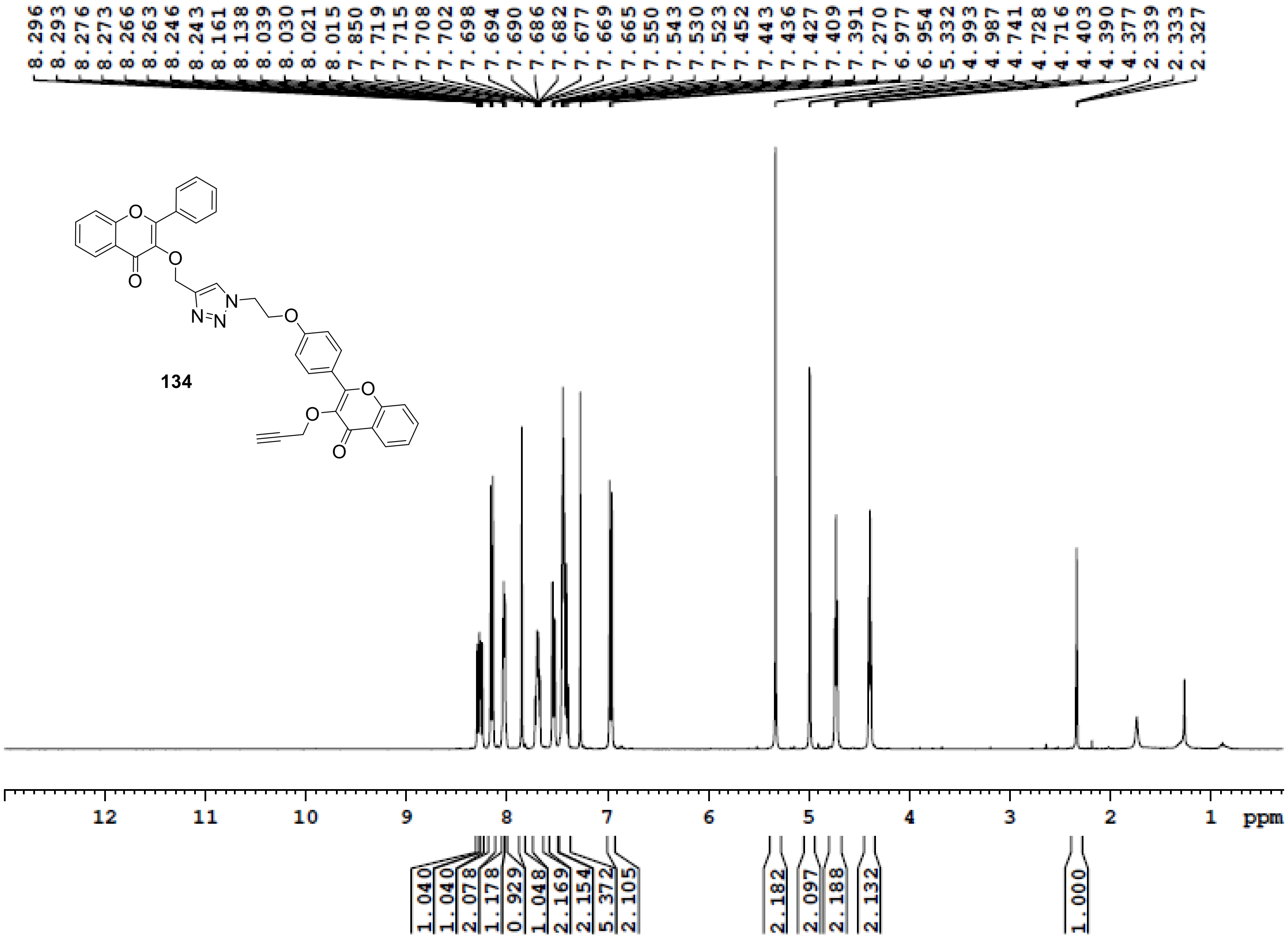
8.296 8.293 8.276 8.273 8.266 8.263 8.246 8.243 8.161 8.138 8.039 8.030 8.021 8.015 7.850 7.719 7.715 7.708 7.702 7.698 7.694 7.690 7.686 7.682 7.677 7.669 7.665 7.550 7.543 7.530 7.523 7.452 7.443 7.436 7.427 7.409 7.391 7.270 6.977 6.954 5.332 4.993 4.987 4.741 4.728 4.716 4.403 4.390 4.377 2.339 2.333 2.327
134
12 11 10 9 8 7 6 5 4 3 2 1 ppm
1.040 1.040 2.078 1.178 0.929 1.048 2.169 2.154 5.372 2.105 2.182 2.097 2.188 2.132 1.000

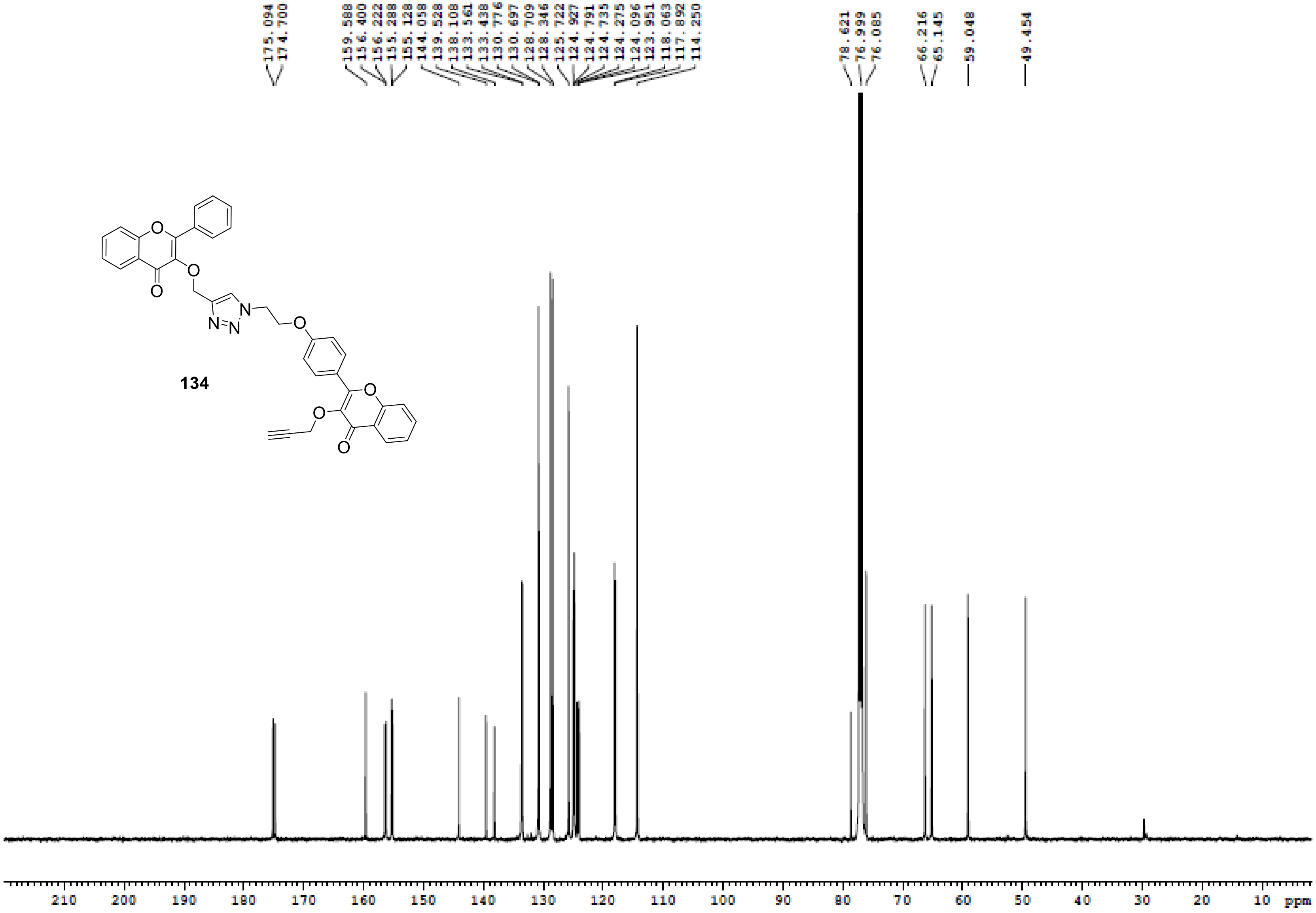
175.094
174.700
159.588
156.400
156.222
155.288
155.128
144.058
139.528
138.108
133.561
133.438
130.776
130.697
128.709
128.346
125.722
124.927
124.791
124.735
124.275
124.096
123.951
118.063
117.892
114.250
78.621
76.999
76.085
66.216
65.145
59.048
49.454
134
210 200 190 180 170 160 150 140 130 120 110 100 90 80 70 60 50 40 30 20 10 ppm

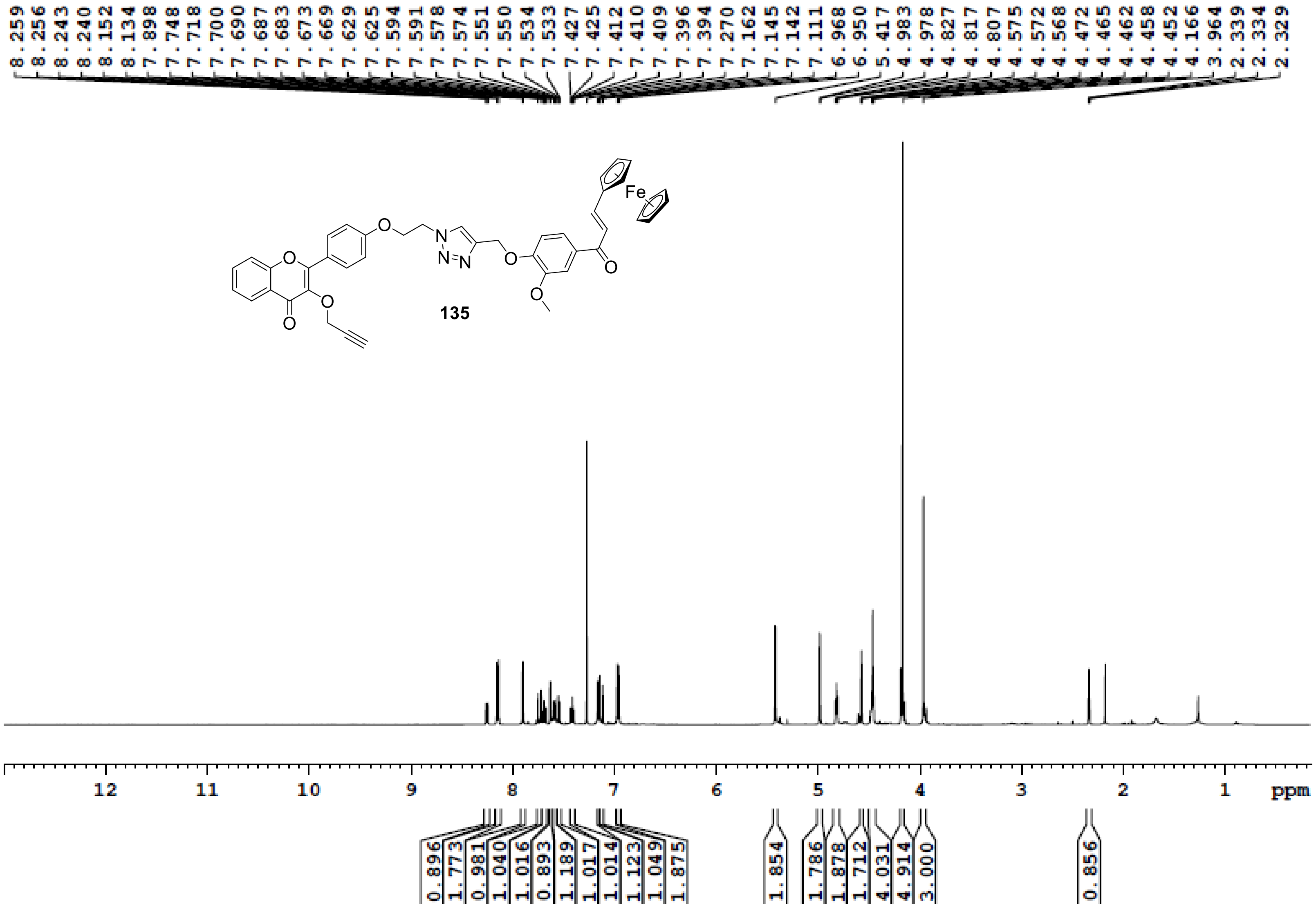
Fe
135
ppm

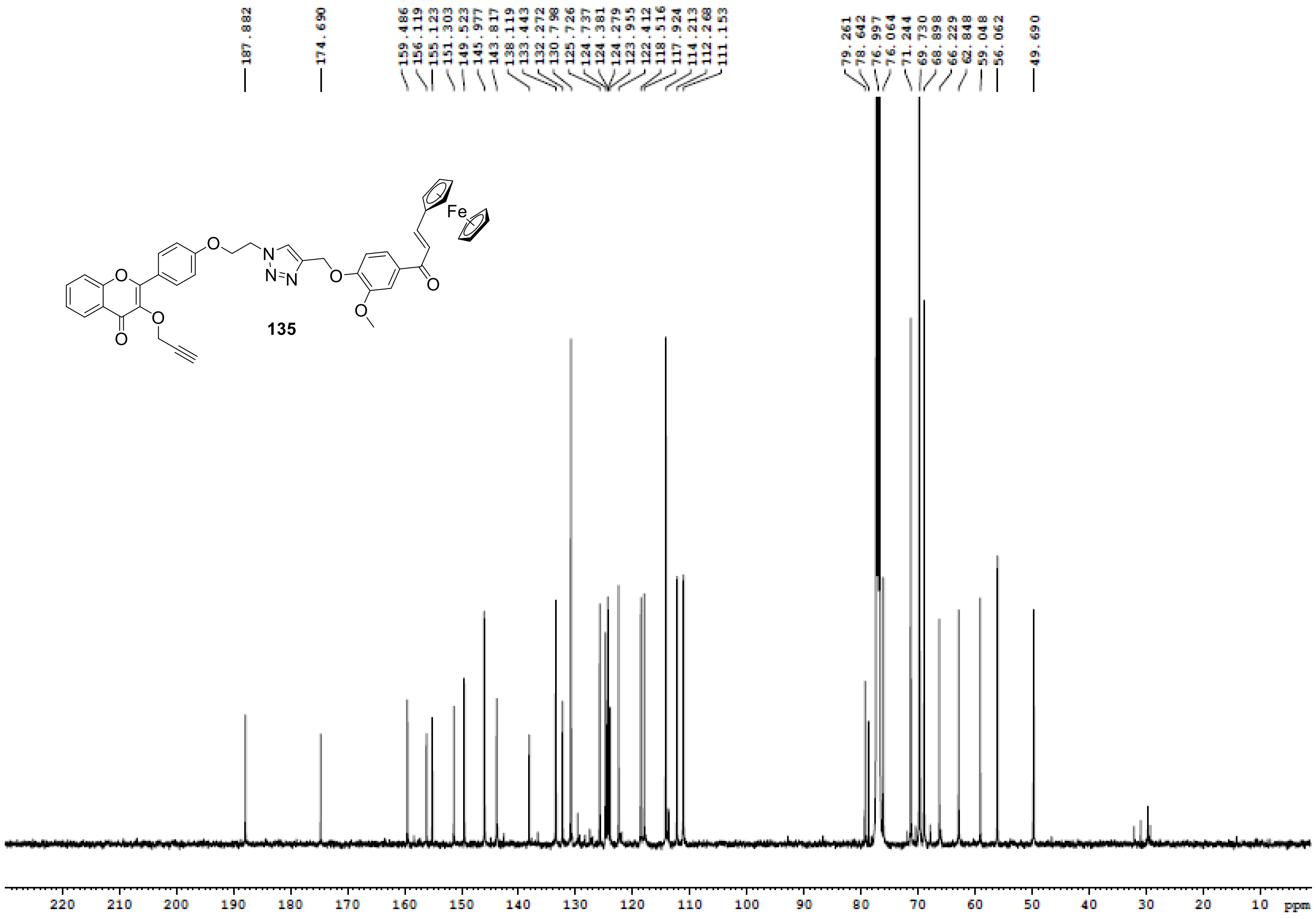
187.882
174.690
159.486
156.119
155.123
151.303
149.523
145.977
143.817
138.119
133.443
132.272
130.798
125.726
124.737
124.381
124.279
123.955
122.412
118.516
117.924
114.213
112.268
111.153
79.261
78.642
76.997
76.064
71.244
69.730
68.898
66.229
62.848
59.048
56.062
49.690
Fe
135
220
210
200
190
180
170
160
150
140
130
120
110
100
90
80
70
60
50
40
30
20
10
ppm

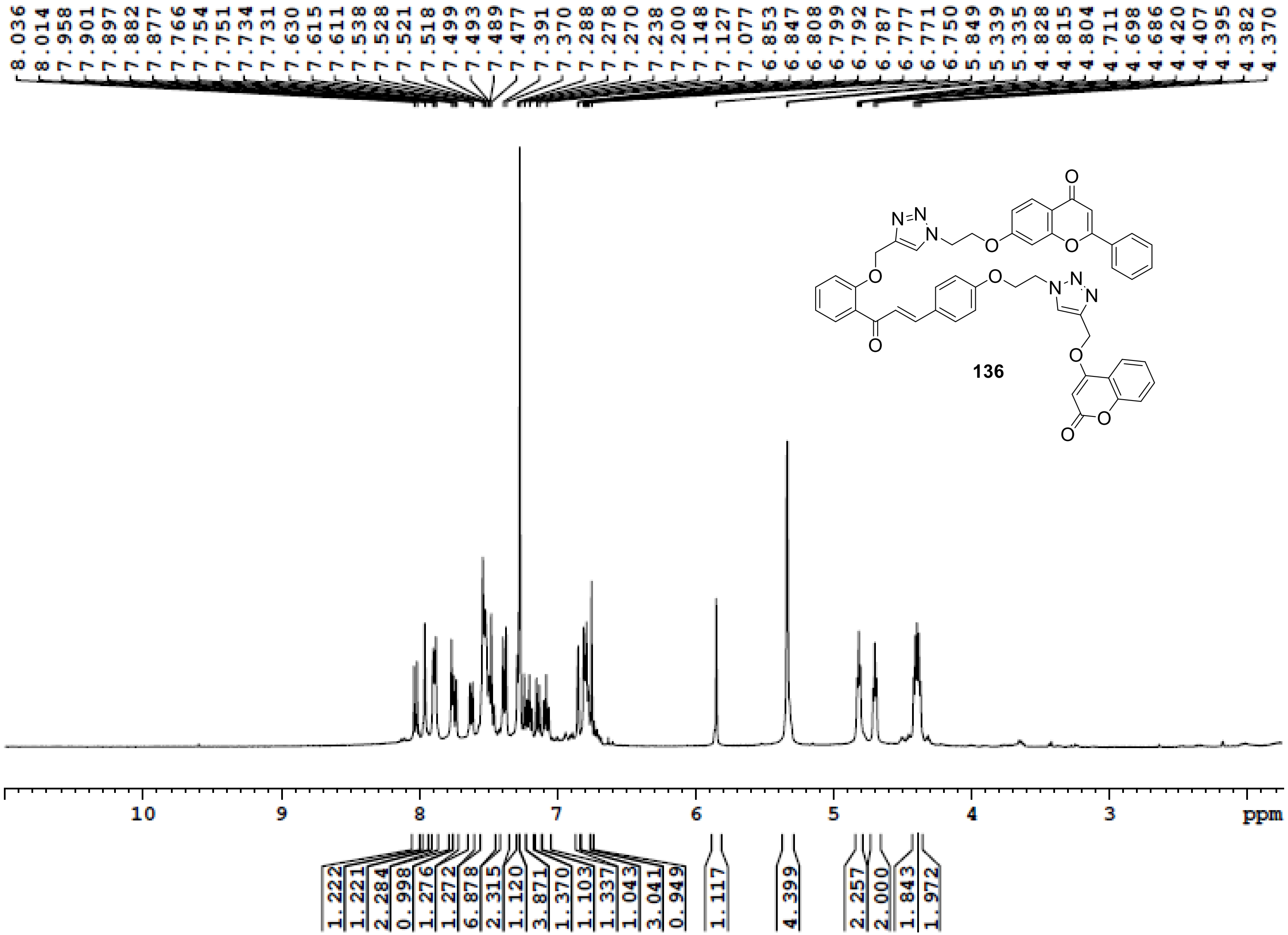
8.036
8.014
7.958
7.901
7.897
7.882
7.877
7.766
7.754
7.751
7.734
7.731
7.630
7.615
7.611
7.538
7.528
7.521
7.518
7.499
7.493
7.489
7.477
7.391
7.370
7.288
7.278
7.270
7.238
7.200
7.148
7.127
7.077
6.853
6.847
6.808
6.799
6.792
6.787
6.777
6.771
6.750
5.849
5.339
5.335
4.828
4.815
4.804
4.711
4.698
4.686
4.420
4.407
4.395
4.382
4.370
136
10
9
8
7
6
5
4
3
ppm
1.222
1.221
2.284
0.998
1.276
1.272
6.878
2.315
1.120
3.871
1.370
1.103
1.337
1.043
3.041
0.949
1.117
4.399
2.257
2.000
1.843
1.972

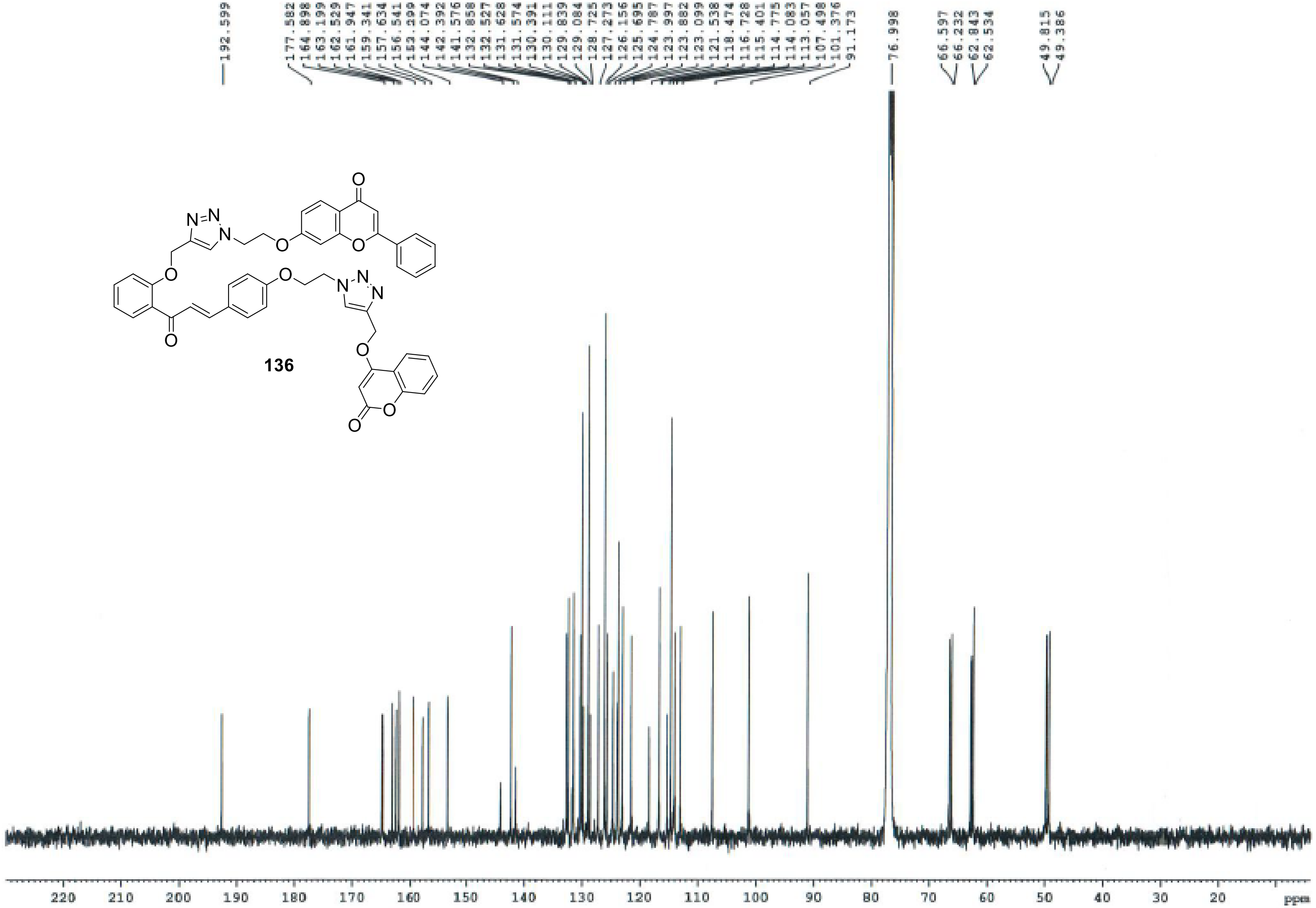
192.599
177.582
164.898
163.199
162.529
161.947
159.341
157.634
156.541
153.399
144.074
142.392
141.576
132.858
132.527
131.628
131.574
130.391
130.111
129.839
129.084
128.725
127.273
126.156
125.695
124.787
123.997
123.882
123.099
121.538
118.474
116.728
115.401
114.775
114.083
113.057
107.498
101.376
91.173
76.998
66.597
66.232
62.843
62.534
49.815
49.386
136
220 210 200 190 180 170 160 150 140 130 120 110 100 90 80 70 60 50 40 30 20
ppm

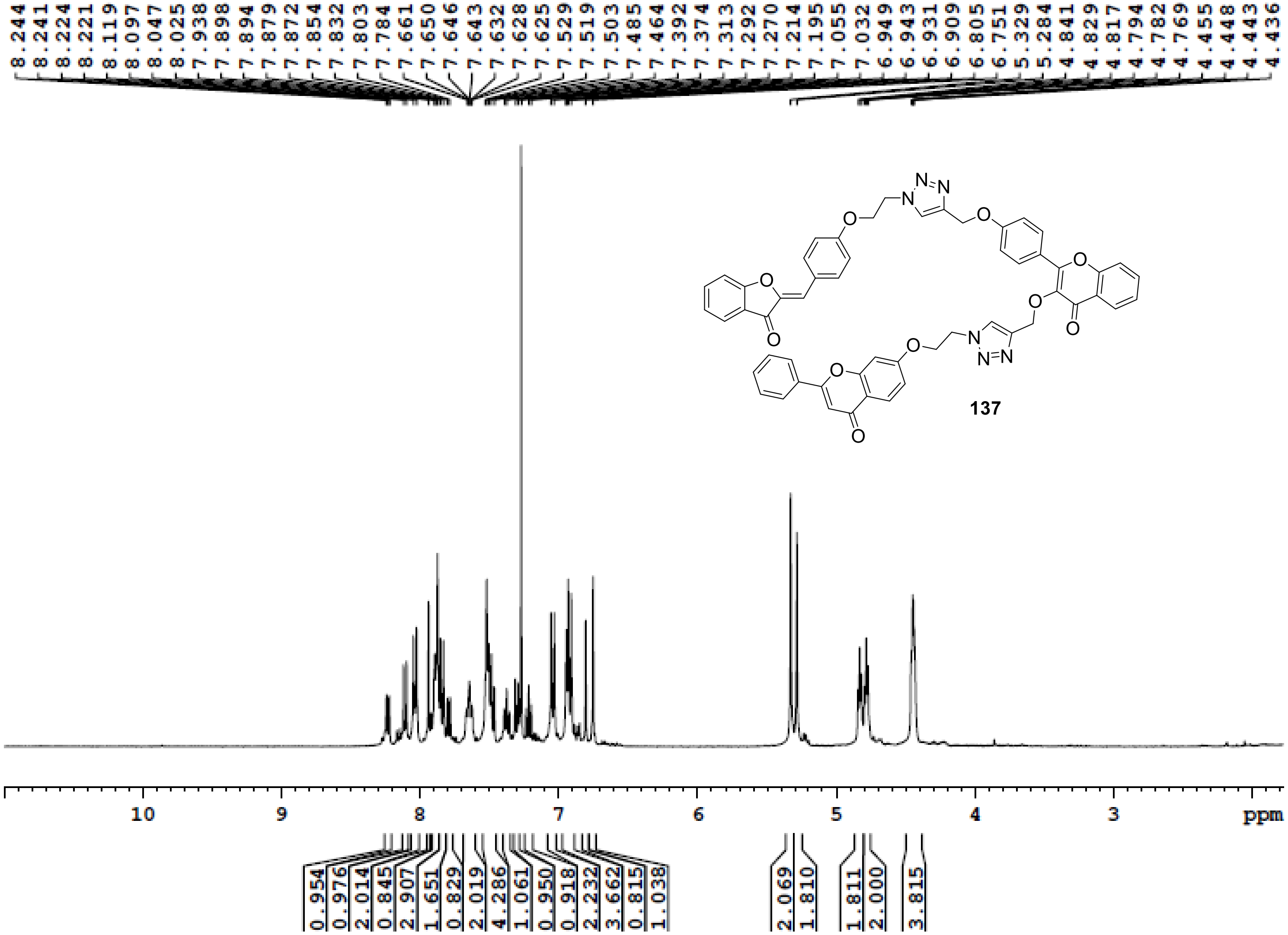
8.244 8.241 8.224 8.221 8.119 8.097 8.047 8.025 7.938 7.898 7.894 7.879 7.872 7.854 7.832 7.803 7.784 7.661 7.650 7.646 7.643 7.632 7.628 7.625 7.529 7.519 7.503 7.485 7.464 7.392 7.374 7.313 7.292 7.270 7.214 7.195 7.055 7.032 6.949 6.943 6.931 6.909 6.805 6.751 5.329 5.284 4.841 4.829 4.817 4.794 4.782 4.769 4.455 4.448 4.443 4.436
137
10
9
8
7
6
5
4
3
ppm
0.954 0.976 2.014 0.845 2.907 1.651 0.829 2.019 4.286 1.061 0.950 0.918 2.232 3.662 0.815 1.038
2.069 1.810
1.811 2.000
3.815

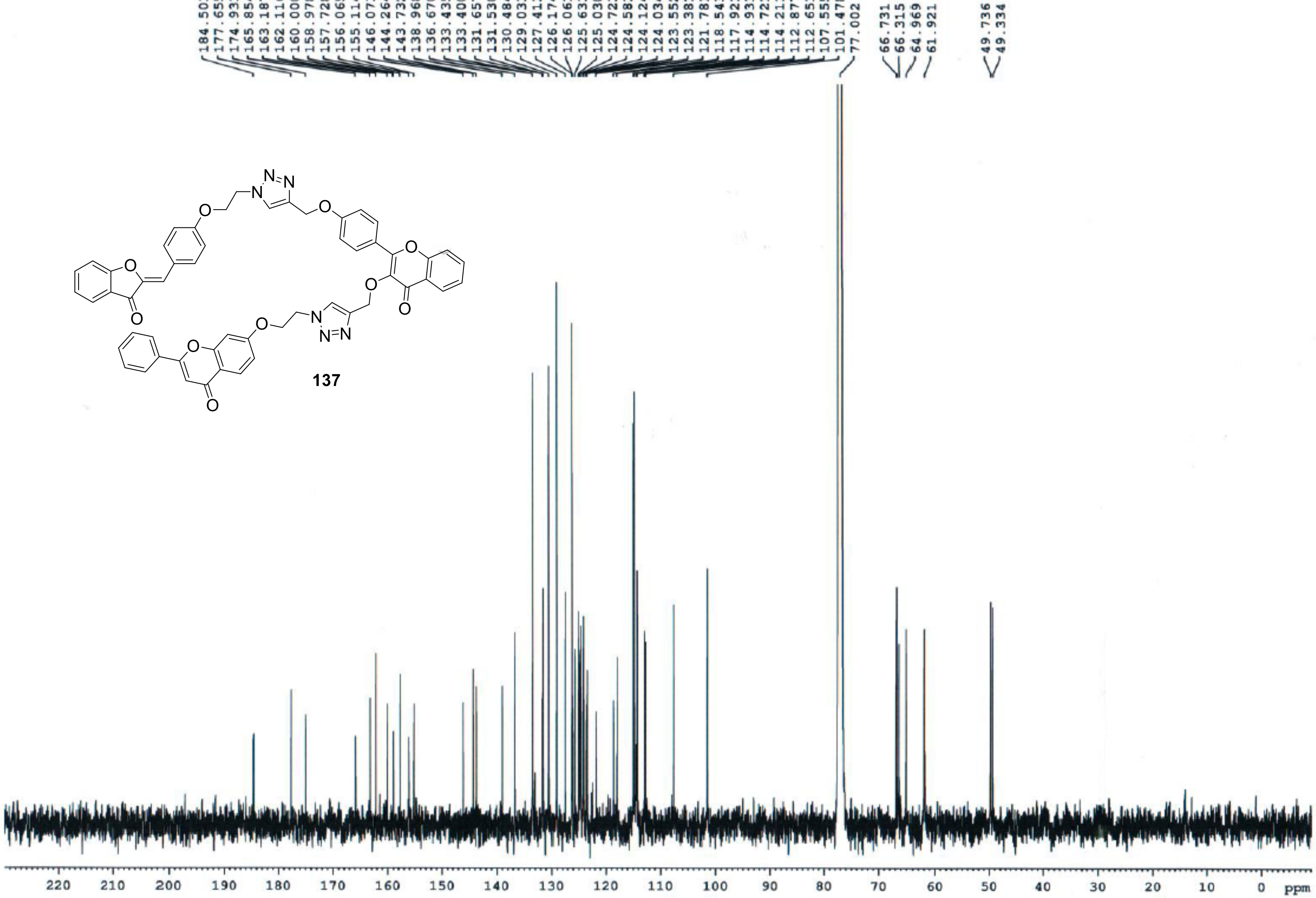
184.503
177.655
174.933
165.854
163.187
162.116
160.000
158.978
157.728
156.065
155.114
146.073
144.264
143.732
138.968
136.670
133.435
133.400
131.657
131.530
130.484
129.033
127.413
126.174
126.061
125.633
125.030
124.723
124.581
124.124
124.034
123.552
123.381
121.781
118.543
117.921
114.933
114.721
114.211
112.877
112.653
107.555
101.478
77.002
66.731
66.315
64.969
61.921
49.736
49.334
137
220 210 200 190 180 170 160 150 140 130 120 110 100 90 80 70 60 50 40 30 20 10 0 ppm

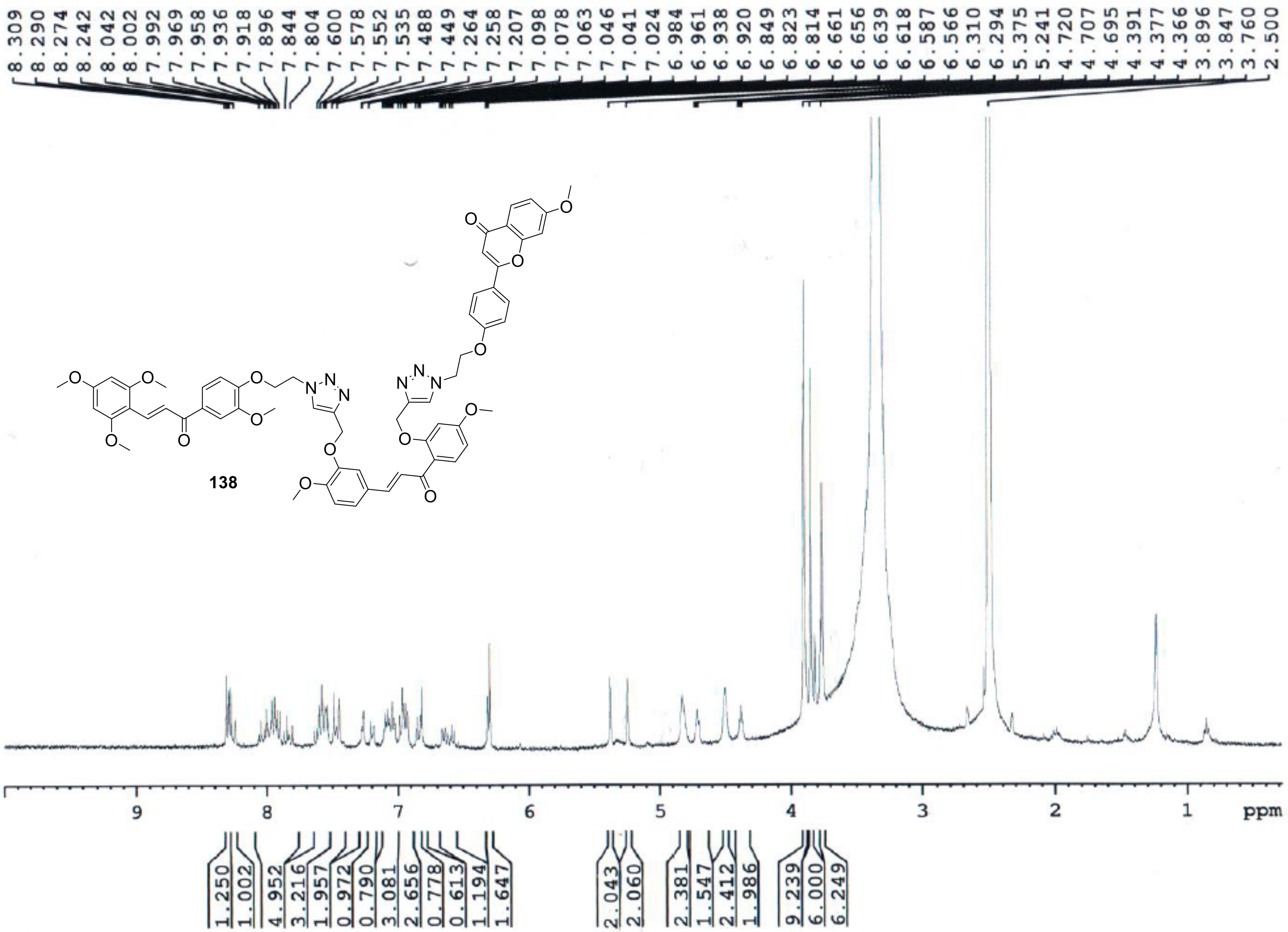
8.309
8.290
8.274
8.242
8.042
8.002
7.992
7.969
7.958
7.936
7.918
7.896
7.844
7.804
7.600
7.578
7.552
7.535
7.488
7.449
7.264
7.258
7.207
7.098
7.078
7.063
7.046
7.041
7.024
6.984
6.961
6.938
6.920
6.849
6.823
6.814
6.661
6.656
6.639
6.618
6.587
6.566
6.310
6.294
5.375
5.241
4.720
4.707
4.695
4.391
4.377
4.366
3.896
3.847
3.760
2.500
138
9
8
7
6
5
4
3
2
1
ppm
1.250
1.002
4.952
3.216
1.957
0.972
0.790
3.081
2.656
0.778
0.613
1.194
1.647
2.043
2.060
2.381
1.547
2.412
1.986
9.239
6.000
6.249

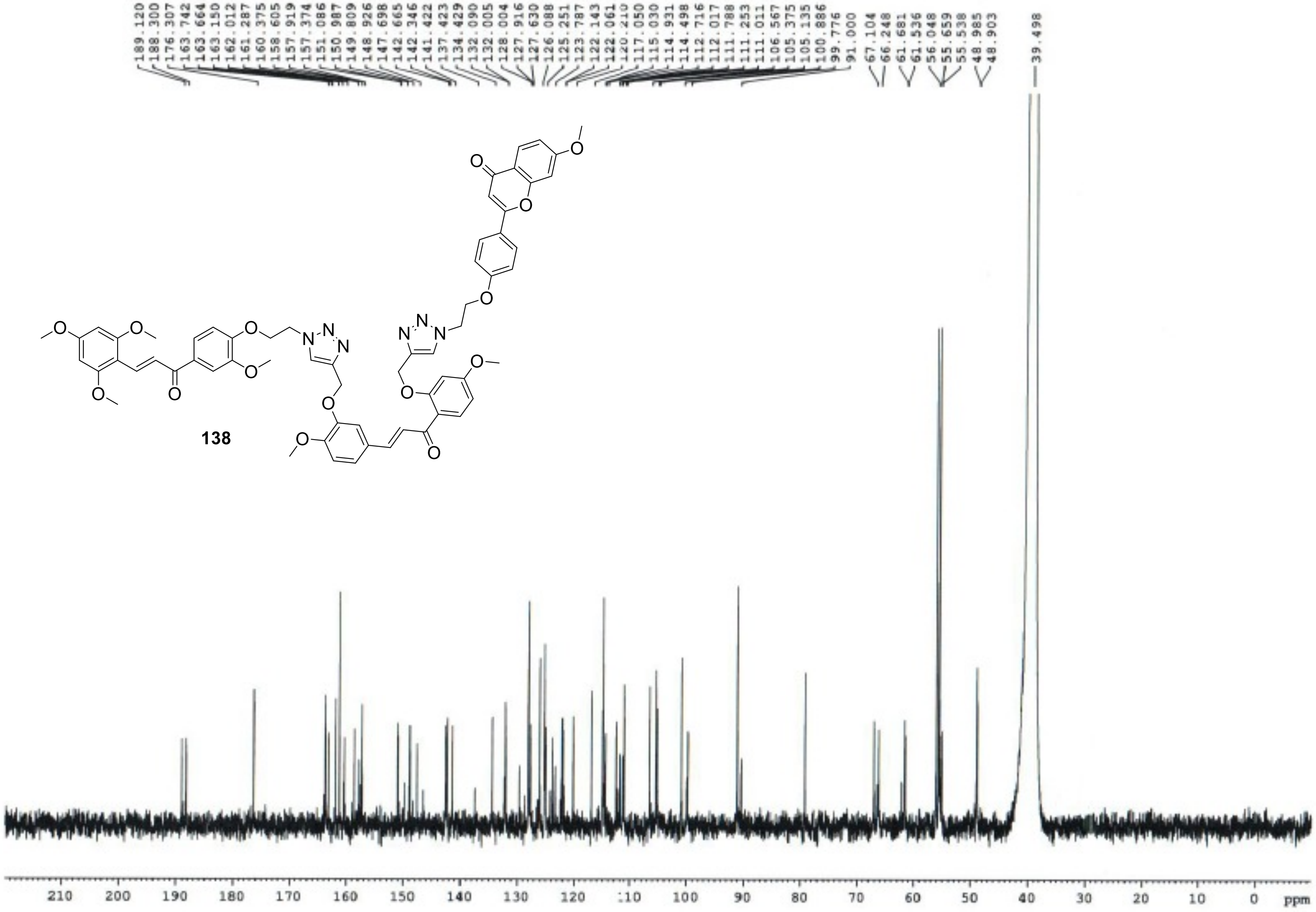
189.120
188.300
176.307
163.742
163.664
163.150
162.012
161.287
160.375
158.605
157.919
157.374
151.086
150.987
149.809
148.926
147.698
142.665
142.346
141.422
137.423
134.429
132.090
132.005
128.004
127.916
127.630
126.088
125.251
123.787
122.143
122.061
120.210
117.050
115.030
114.931
114.498
112.716
112.017
111.788
111.253
111.011
106.567
105.375
105.135
100.886
99.776
91.000
67.104
66.248
61.681
61.536
56.048
55.659
55.538
48.985
48.903
39.498
138
210 200 190 180 170 160 150 140 130 120 110 100 90 80 70 60 50 40 30 20 10 0 ppm

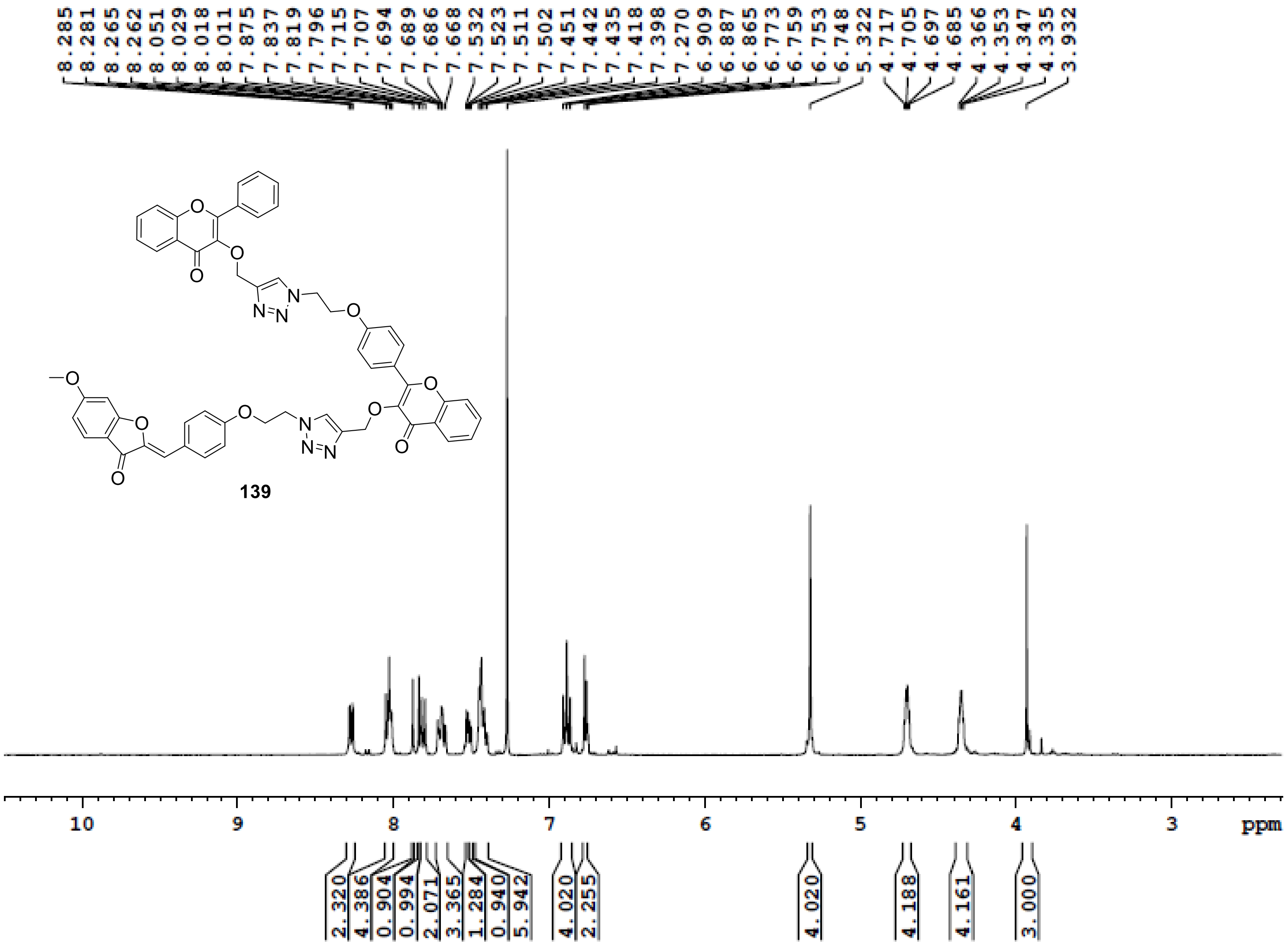
139
10
9
8
7
6
5
4
3
ppm

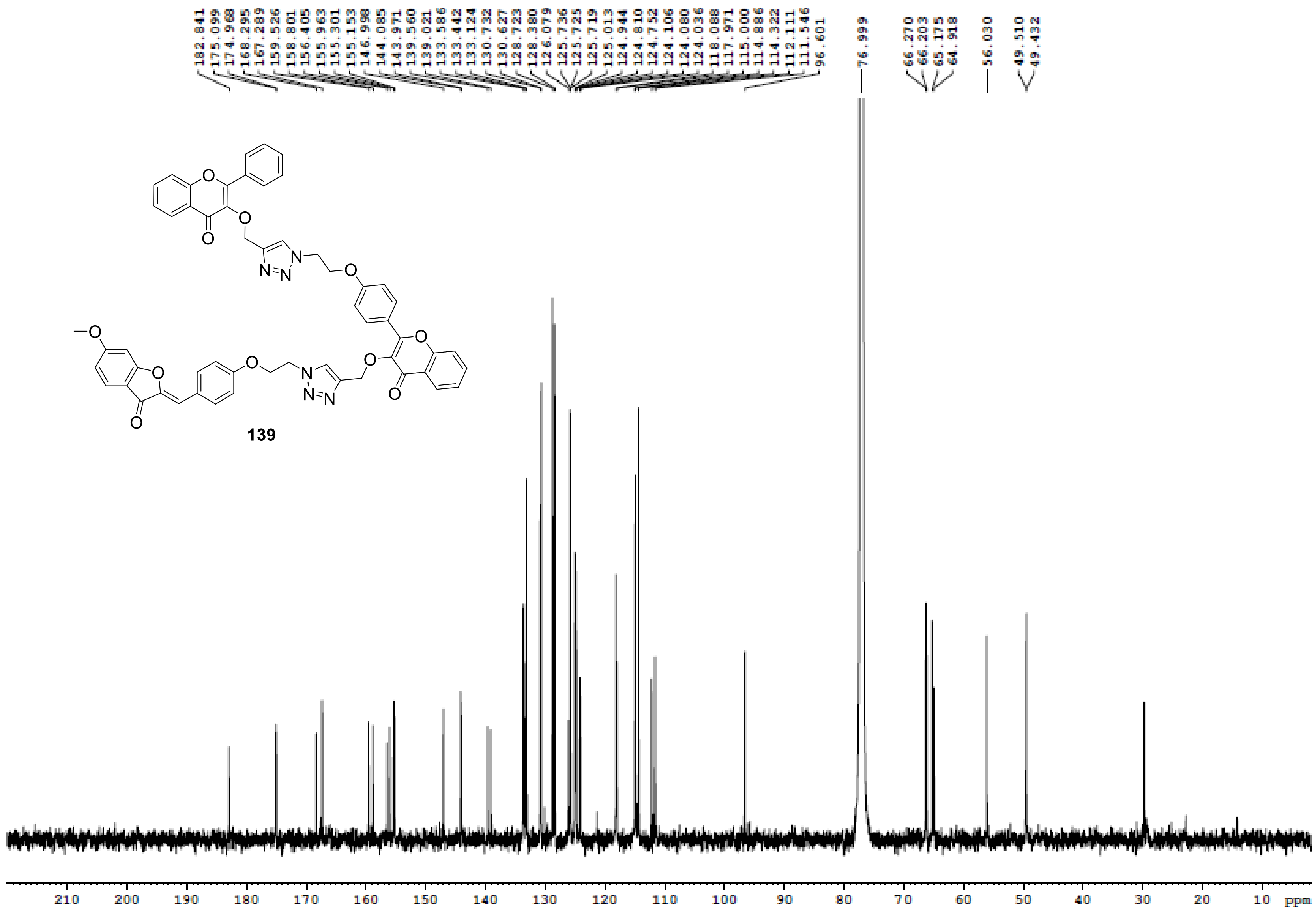
182.841
175.099
174.968
168.295
167.289
159.526
158.801
156.405
155.963
155.301
155.153
146.998
144.085
143.971
139.560
139.021
133.586
133.442
133.124
130.732
130.627
128.723
128.380
126.079
125.736
125.725
125.719
125.013
124.944
124.810
124.752
124.106
124.080
124.036
118.088
117.971
115.000
114.886
114.322
112.111
111.546
96.601
76.999
66.270
66.203
65.175
64.918
56.030
49.510
49.432
139
210 200 190 180 170 160 150 140 130 120 110 100 90 80 70 60 50 40 30 20 10 ppm

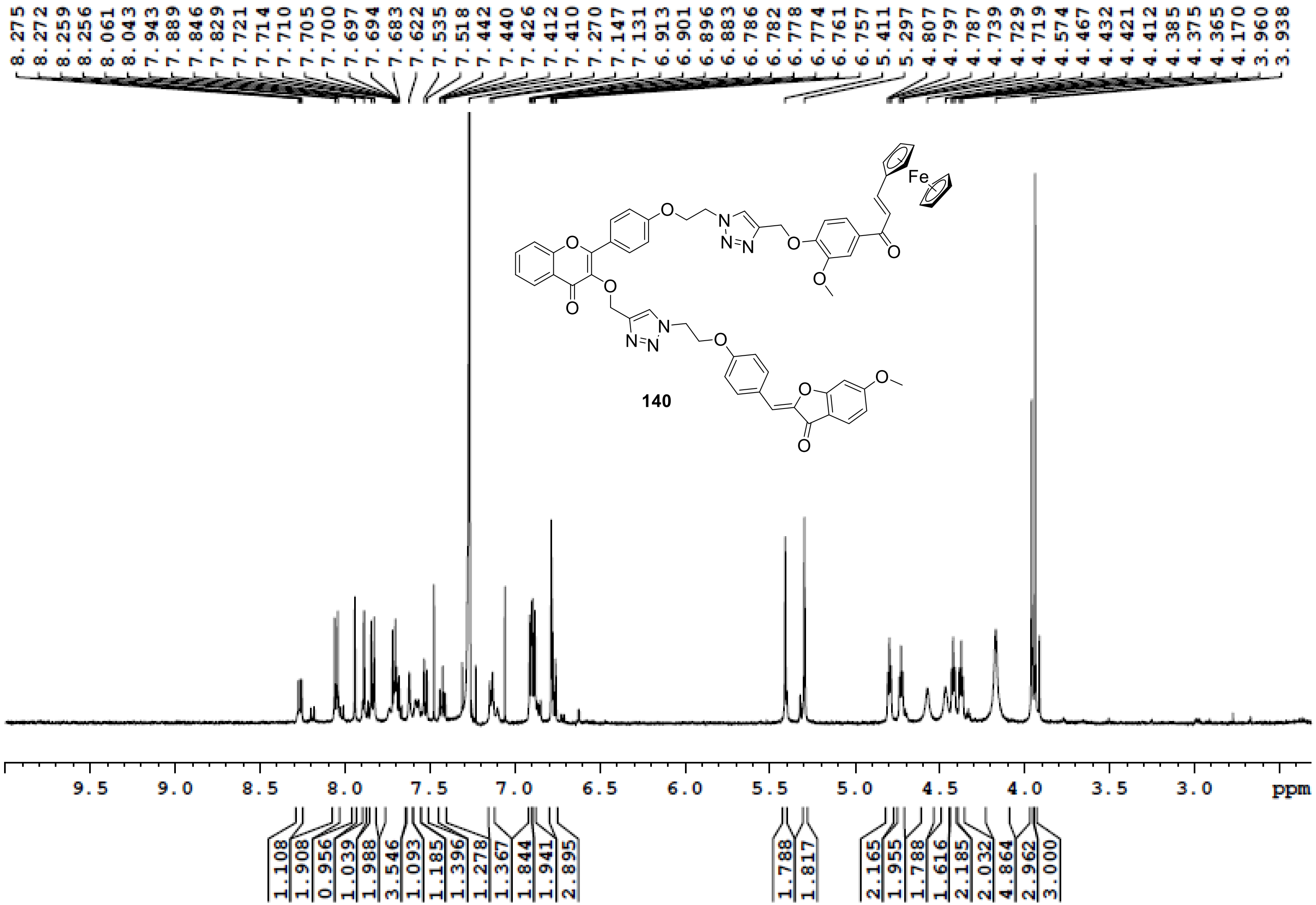
8.275 8.272 8.259 8.256 8.061 8.043 7.943 7.889 7.846 7.829 7.721 7.714 7.710 7.705 7.700 7.697 7.694 7.683 7.622 7.535 7.518 7.442 7.440 7.426 7.412 7.410 7.270 7.147 7.131 6.913 6.901 6.896 6.883 6.786 6.782 6.778 6.774 6.761 6.757 5.411 5.297 4.807 4.797 4.787 4.739 4.729 4.719 4.574 4.467 4.432 4.421 4.412 4.385 4.375 4.365 4.170 3.960 3.938
Fe
9.5 9.0 8.5 8.0 7.5 7.0 6.5 6.0 5.5 5.0 4.5 4.0 3.5 3.0 ppm
1.108 1.908 0.956 1.039 1.988 3.546 1.093 1.185 1.396 1.278 1.367 1.844 1.941 2.895 1.788 1.817 2.165 1.955 1.788 1.616 2.185 2.032 4.864 2.962 3.000

140

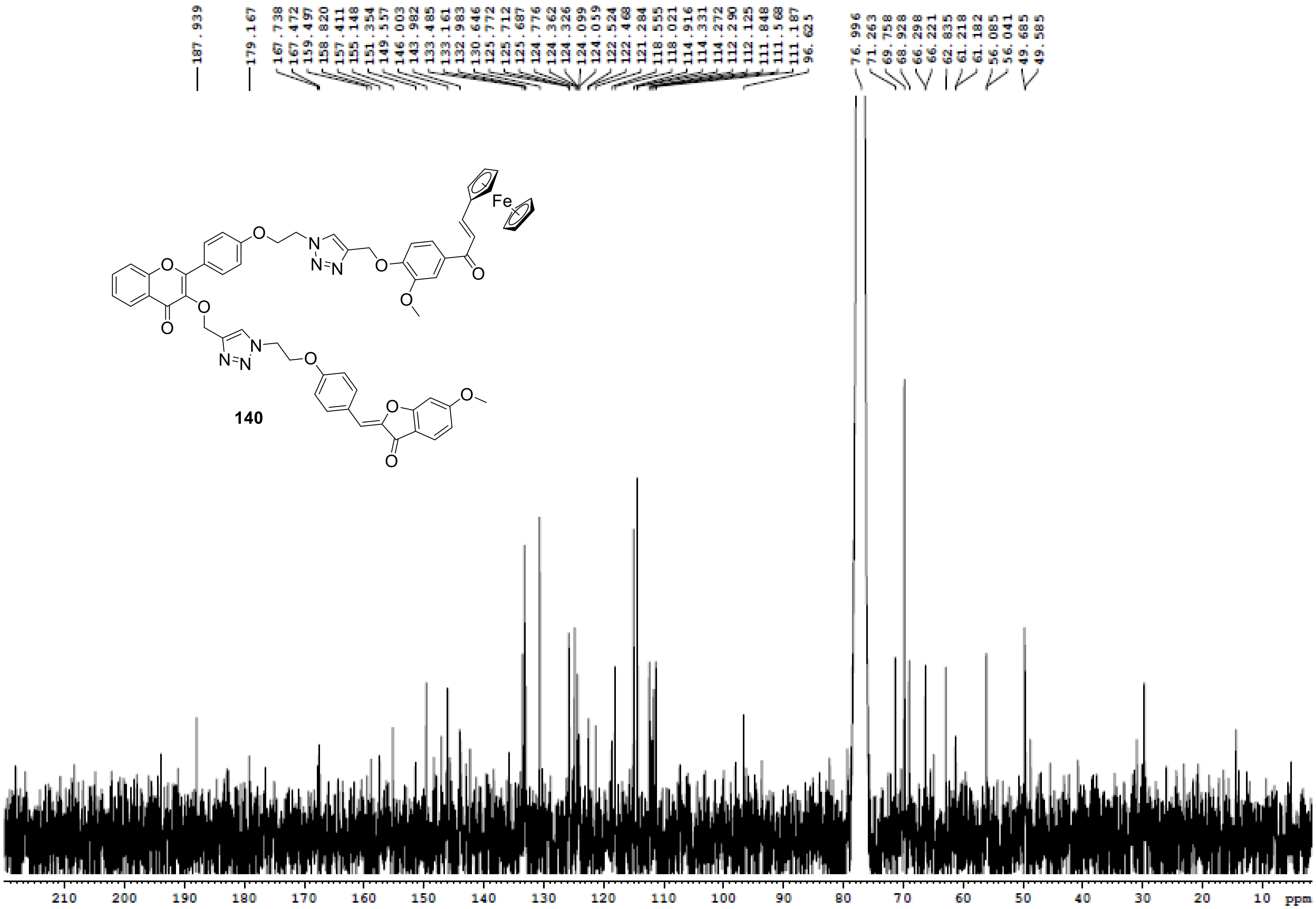
187.939
179.167
167.738
167.472
159.497
158.820
157.411
155.148
151.354
149.557
146.003
143.982
133.485
133.161
132.983
130.646
125.772
125.712
125.687
124.776
124.362
124.326
124.099
124.059
122.524
122.468
121.284
118.555
118.021
114.916
114.331
114.272
112.290
112.125
111.848
111.568
111.187
96.625
76.996
71.263
69.758
68.928
66.298
66.221
62.835
61.218
61.182
56.085
56.041
49.685
49.585
Fe
140
210 200 190 180 170 160 150 140 130 120 110 100 90 80 70 60 50 40 30 20 10 ppm
